# The Design and Synthesis of a New Series of 1,2,3-Triazole-Cored Structures Tethering Aryl Urea and Their Highly Selective Cytotoxicity toward HepG2

**DOI:** 10.3390/ph15050504

**Published:** 2022-04-20

**Authors:** Sittisak Oekchuae, Jitnapa Sirirak, Purin Charoensuksai, Pawaris Wongprayoon, Natthaya Chuaypen, Jutatip Boonsombat, Somsak Ruchirawat, Pisit Tangkijvanich, Apichart Suksamrarn, Panupun Limpachayaporn

**Affiliations:** 1 Department of Chemistry, Faculty of Science, Silpakorn University, Nakhon Pathom 73000, Thailand; oekchuae.s@gmail.com (S.O.); jitnapasirirak@gmail.com (J.S.); 2 Chulabhorn Research Institute, Bangkok 10210, Thailand; jutatip@cri.or.th (J.B.); somsak@cri.or.th (S.R.); 3 Department of Biopharmacy, Faculty of Pharmacy, Silpakorn University, Nakhon Pathom 73000, Thailand; charoensuksai_p@su.ac.th (P.C.); wongprayoon_p@su.ac.th (P.W.); 4 Center of Excellence in Hepatitis and Liver Cancer, Department of Biochemistry, Faculty of Medicine, Chulalongkorn University, Bangkok 10330, Thailand; natthaya.c@chula.ac.th (N.C.); pisittkvn@yahoo.com (P.T.); 5 Center of Excellence on Environmental Health and Toxicology (EHT), OPS, MHESI, Bangkok 10400, Thailand; 6 Program in Chemical Sciences, Chulabhorn Graduate Institute, Chulabhorn Royal Academy, Bangkok 10210, Thailand; 7 Department of Chemistry and Center of Excellence for Innovation in Chemistry, Faculty of Science, Ramkhamhaeng University, Bangkok 10240, Thailand; asuksamrarn@yahoo.com

**Keywords:** sorafenib analog, 1,2,3-triazole-containing drug, click reaction, selective anti-HepG2 agent, targeted cancer drug, hepatocellular carcinoma (HCC), drug discovery

## Abstract

Target cancer drug therapy is an alternative treatment for advanced hepatocellular carcinoma (HCC) patients. However, the treatment using approved targeted drugs has encountered a number of limitations, including the poor pharmacological properties of drugs, therapy efficiency, adverse effects, and drug resistance. As a consequence, the discovery and development of anti-HCC drug structures are therefore still in high demand. Herein, we designed and synthesized a new series of 1,2,3-triazole-cored structures incorporating aryl urea as anti-HepG2 agents. Forty-nine analogs were prepared via nucleophilic addition and copper-catalyzed azide-alkyne cycloaddition (CuAAC) with excellent yields. Significantly, almost all triazole-cored analogs exhibited less cytotoxicity toward normal cells, human embryonal lung fibroblast cell MRC-5, compared to Sorafenib and Doxorubicin. Among them, **2m’** and **2e** exhibited the highest selectivity indexes (SI = 14.7 and 12.2), which were ca. 4.4- and 3.7-fold superior to that of Sorafenib (SI = 3.30) and ca. 3.8- and 3.2-fold superior to that of Doxorubicin (SI = 3.83), respectively. Additionally, excellent inhibitory activity against hepatocellular carcinoma HepG2, comparable to Sorafenib, was still maintained. A cell-cycle analysis and apoptosis induction study suggested that **2m’** and **2e** likely share a similar mechanism of action to Sorafenib. Furthermore, compounds **2m’** and **2e** exhibit appropriate drug-likeness, analyzed by SwissADME. With their excellent anti-HepG2 activity, improved selectivity indexes, and appropriate druggability, the triazole-cored analogs **2m’** and **2e** are suggested to be promising candidates for development as targeted cancer agents and drugs used in combination therapy for the treatment of HCC.

## 1. Introduction

Cancer is a major public health problem in every country, as it is the leading cause of death worldwide and has an increasing incidence every year. The International Agency for Research on Cancer (IARC) reported 19,292,789 new cases of cancers and 9,958,133 deaths globally in 2020. Lung, colorectal, and liver cancers are the highest-ranked causes of death, with mortality rates of 81%, 48%, and 92%, respectively [1]. Various methods, including surgery, transplantation, radiation therapy, chemotherapy, and targeted drug therapy, have been used for the treatment of cancer [2,3], depending on the types and stages of cancers, as well as patient readiness [4,5,6,7]. In general, early-stage cancers can be cured by curative resection, radiation therapy, and transplantation, while chemotherapy and targeted drug therapy are the main options for the treatment of developed and advanced-stage cancers [8,9,10]. Targeted cancer drug therapy is an approach for the treatment of advanced cancers, which suppresses cancer cell growth mainly by the selective inhibition of enzymes and receptors in several signal transduction pathways [11]. Thus, the targeted therapy is generally effective with fewer adverse effects than chemotherapy [12,13]. Nevertheless, approved targeted cancer drugs can cause unexpected side effects and possess inappropriate pharmacological properties [14,15]. In addition, cancers can develop drug resistance, limiting the responsiveness of treatment in some patients [16,17]. Therefore, the discovery and structural developments of new, appropriate targeted cancer drugs with high safety profiles and drug-likeness must be intensively carried out.

Sorafenib (Nexavar^®^) is the first targeted cancer agent approved by the Food and Drug Administration of the United States (US FDA) for the treatment of advanced hepatocellular carcinoma (HCC) and later approved for the treatment of renal cell carcinoma (RCC) and thyroid cancer [18,19]. It suppresses cancer growth due to its multi-kinase inhibitory properties toward various receptor tyrosine kinases (RTKs) associated with cell proliferation, migration, differentiation, and angiogenesis [20]. In particular, Sorafenib is able to inhibit B-Raf [21] and VEGFR-2 [20,22,23,24,25,26], which are overexpressed in many cancers including HCC, RCC, thyroid carcinoma, non-small lung, breast, colorectal and ovarian cancers. However, cancer therapy using Sorafenib requires a high daily dose due to its poor bioavailability of 38–49% [19] and often causes unexpected adverse effects, e.g., cardiovascular reactions (hypertension), hand–foot skin reaction (HFSR), diarrhea, renal toxicity, fatigue, etc. [27]. Moreover, several recent studies reported drug resistance to Sorafenib treatment in cancer therapy [20,28].

**Figure 1:**
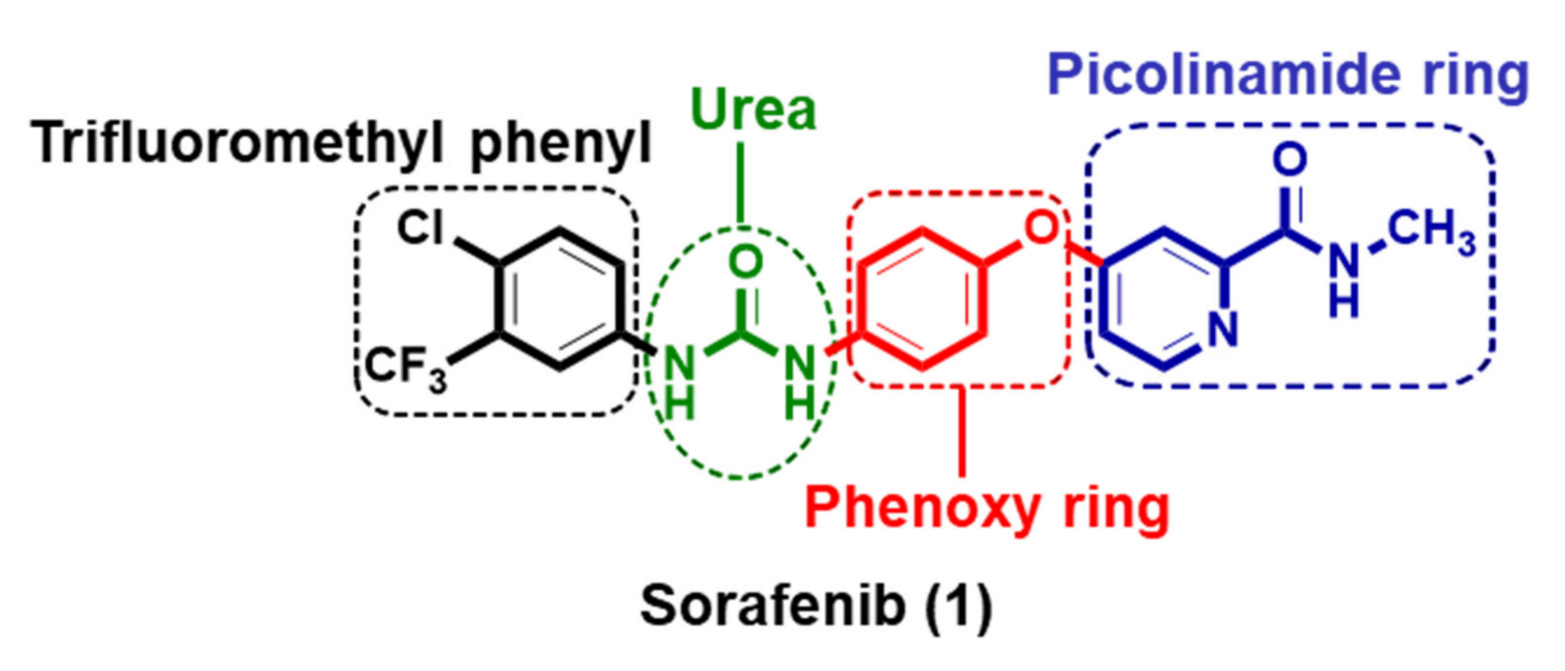
The chemical structure and the components of Sorafenib (**1**).

Although Sorafenib possessed some disadvantages, as aforementioned, the structure of Sorafenib (Figure 1) interacted well with important pockets in the active sites of RTKs, including B-Raf and VEGFR-2 [5,21,22,29,30,31]. Hence, it inspires organic and medicinal chemists to develop new targeted cancer agents by structural modification based on Sorafenib’s structure to enhance the therapeutic efficiency targeting overexpressed kinases [20,22,23,24,25,26,29,32,33,34,35,36], as well as to expand anti-tumor activities and improve the selectivity and pharmacological properties. Previous structural modifications of Sorafenib revealed that the presence of trifluoromethyl phenyl rings and the urea moiety of Sorafenib were essential for anti-cancer properties, since changing these moieties resulted in decreased activity [6,7,37,38,39]. On the other hand, the replacement of the phenoxy core [29,39,40,41,42] and picolinamide part [7,37,40,41,42] with various aromatic and heterocyclic rings maintained or enhanced the cytotoxicity and broadened inhibitory activities toward cancer cell lines.

**Figure 2:**
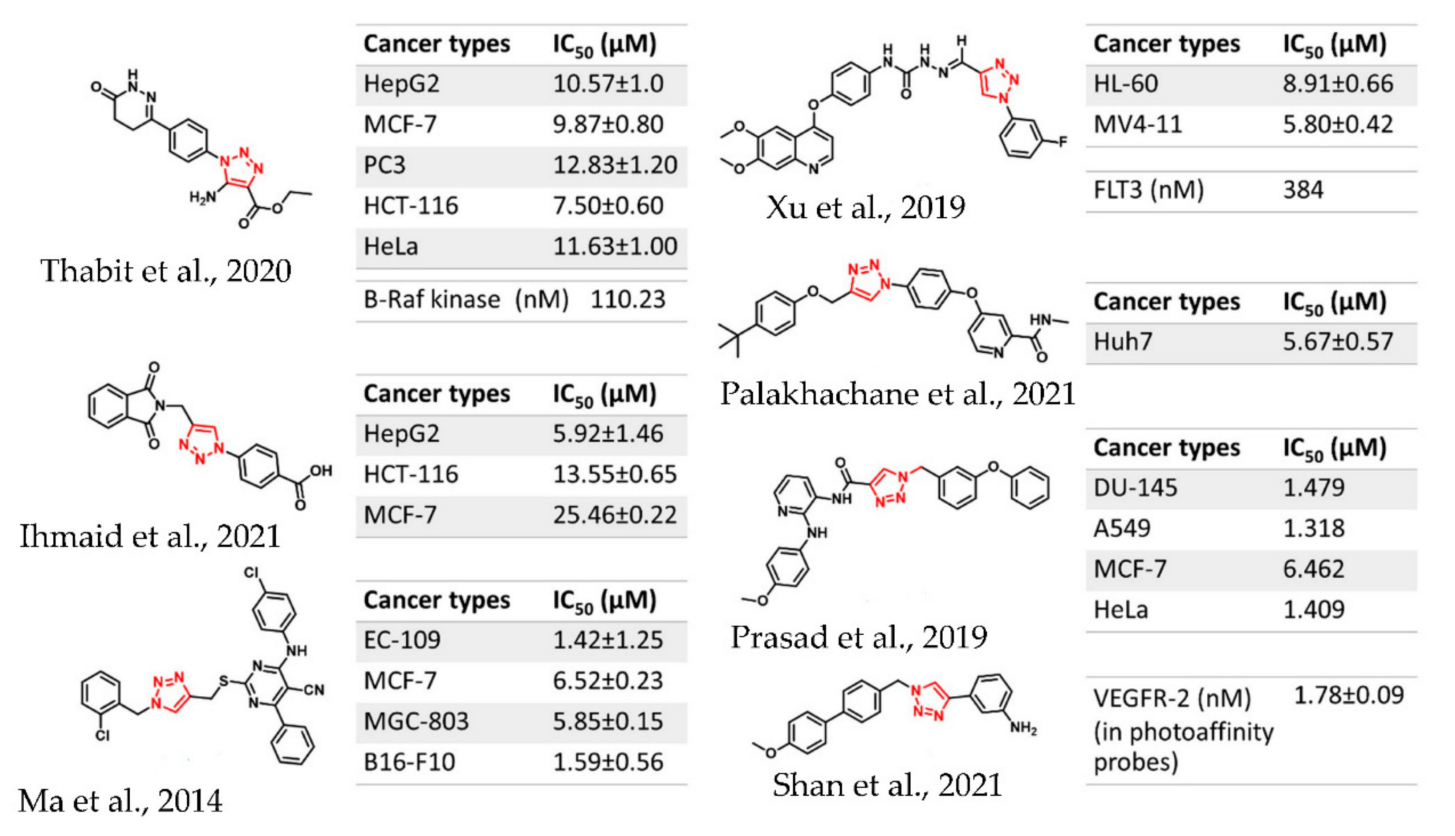
Some 1,2,3-triazole-containing analogs with their kinase-inhibitory and anti-cancer activities [4,31,49,50,51,52,53].

We have been interested in the structural development of Sorafenib using a 1,2,3-triazole ring as a component in the structure, since we envisioned that this aromatic heterocycle should interact well with the pocket of the targets by providing hydrogen bonds, π-π, and hydrophobic interactions. Furthermore, its structure is similar to amino acid histidine, leading to bio-compatibility [43,44], which has been proven to resist metabolism [4,45]. This structure also exists in a wide range of bioactive compounds including anti-cancer agents [4,37,46,47,48]. In recent years, several 1,2,3-triazole-incorporating compounds have been designed and synthesized, and they displayed good to excellent inhibitory activities toward a variety of cancer cell lines, as depicted in Figure 2 [4,31,49,50,51,52,53,54]. Recently, our group also demonstrated that the triazole-containing analogs obviously had inhibitory activity against hepatocellular carcinoma cell line Huh7 with a high selectivity index (SI). Thus, the 1,2,3-triazole structural feature had the potential to enhance the compound’s safety profile, while the anti-cancer activity could potentially be preserved [4].

**Figure 3:**
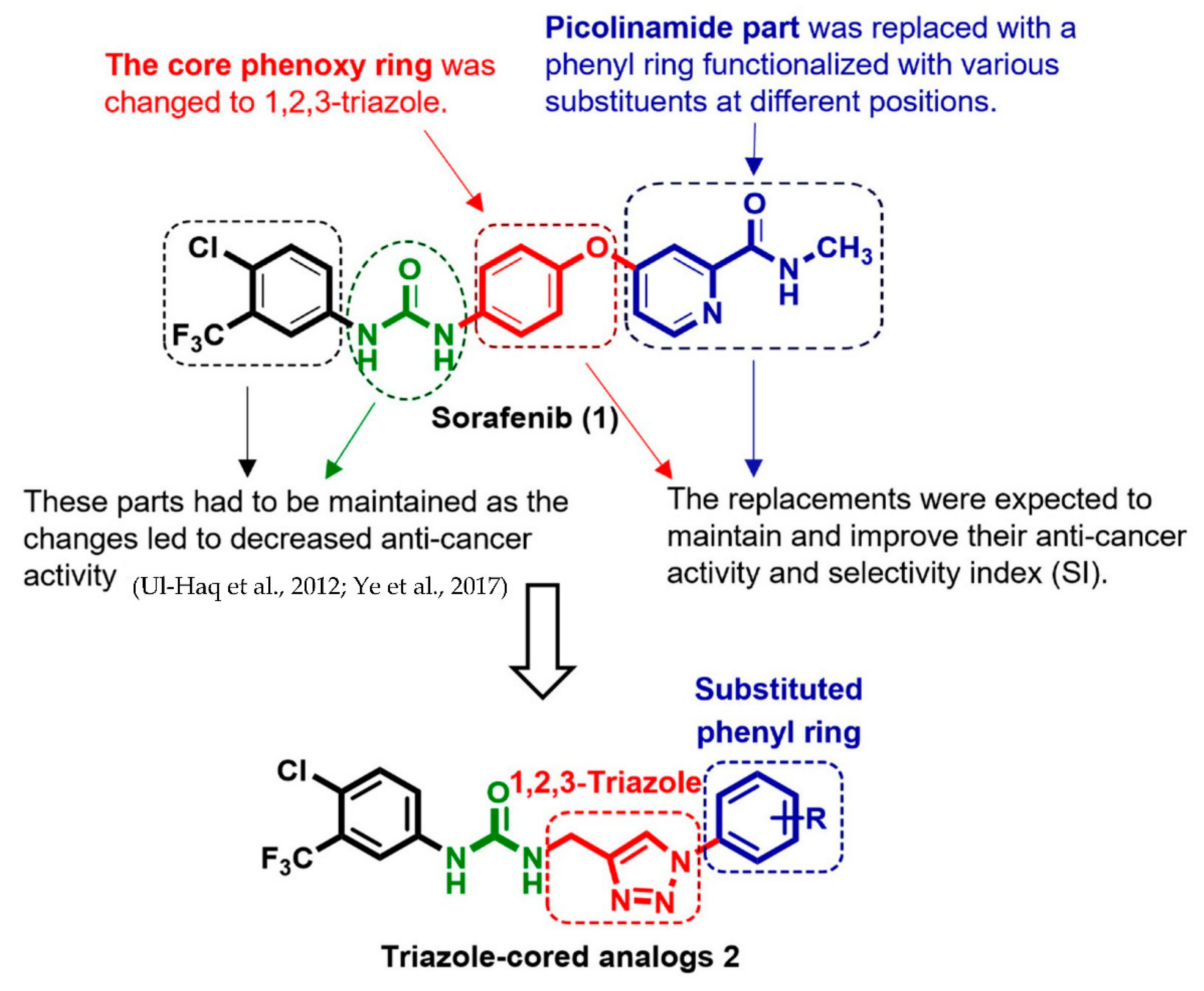
The structure of newly designed triazole-containing analogs **2** based on the modification of Sorafenib’s structure (**1**) [5,46].

**Scheme 1:**
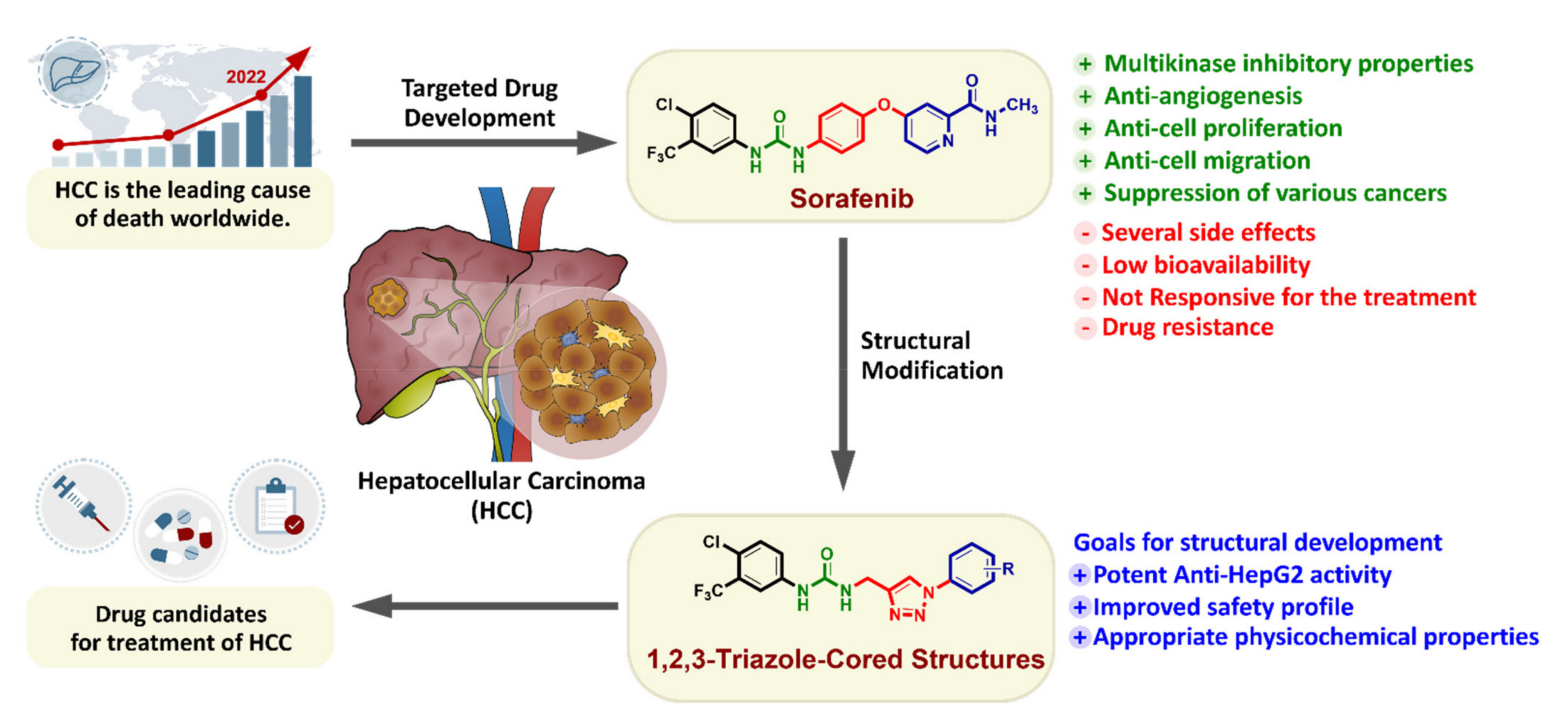
Background summary of this work.

Our previous work showed that replacing the aryl urea of Sorafenib with a 1,2,3-triazole ring resulted in a significantly reduced toxicity of the compounds, while removing an important aryl urea moiety drastically lowered the cytotoxicity toward hepatocellular carcinoma cell lines (HepG2 and Huh7) [4]. Based on this information, both the triazole part and the aryl urea moiety are retained in order to take these advantageous properties to our newly designed structures. Thus, in this new series of triazole-containing analogs **2** (Figure 3), compared to the Sorafenib structure, the phenoxy and picolinamide portion was replaced with a 1,2,3-triazole linked to a substituted phenyl moiety, while aryl urea was maintained to preserve the anti-cancer activities. Our designed molecules were expected to target kinases and/or other proteins and mechanisms relating to cancer cell growth. We have hypothesized that the substitution of the core phenoxy ring with the heterocycle could provide interaction with targets through π-π stacking and additional hydrogen bonding to the nitrogen atoms. Although the picolinamide part in the hinge region is highly conserved for the inhibition of kinases [55], it could tolerate substitution with heteroaromatics [7,37,38,41] or aromatics substituted with O- and N-containing functionalities, which provide important interactions, as picolinamide does in the hinge region, and they exhibit inhibitory activities toward kinases [56,57,58,59]. For example, the replacement of picolinamide with sulfonylphenyl [56], methoxyphenyl [57], cyanophenyl [58], and chlorophenyl [59] in the hinge region resulted in maintaining inhibitory activities toward cancer cell lines including HepG2, and kinases including VEGFR-2 and B-Raf. Therefore, the replacement of picolinamide moiety with the substituted phenyl ring could retain the π-π interaction. Moreover, O- and N-containing substituents on the phenyl ring—for example, OH, OMe, CN, COOH, NH_2_, NHAc, and CONHMe—together with interactions by 1,2,3-triazole, were expected to compensate for or mimic the interaction, which the picolinamide of Sorafenib does with targets including kinases. Other substituents, such as halogens, CF_3_, and various alkyl groups could be functionalized in the analogs to explore the opportunity to inhibit kinases and/or other proteins associated with cancer development. Expectedly, it could result in potent anti-cancer activities. The background summary of this work is illustrated in Scheme 1.

Based on our rationale, we aim to develop new structures containing 1,2,3-triazole for efficient anti-cancer agents with a high safety profile, using Sorafenib as a lead compound. We herein report the synthesis of a new series of 1,2,3-triazole-cored analogs tethering aryl urea and a substituted aromatic ring. The cytotoxicity of the synthesized compounds toward five cancer cell lines, including hepatocellular carcinoma HepG2, human lung carcinoma cells A549, Thai human cholangiocarcinoma HuCCA-1, T-cell acute lymphoblastic leukemia MOLT-3, and acute promyelocytic leukemia HL-60, and their structure–activity relationships (SARs) are also described. To reflect the safety profile of the analogs, the cytotoxicity toward human embryonal lung fibroblast cells MRC-5 and the selectivity index (SI) were evaluated. In addition, the cell-cycle arrest profile and apoptosis induction assay of the active analogs were studied, compared to Sorafenib. Finally, the drug-likeness was also analyzed.

## 2. Results and Discussion

### 2.1. Chemistry

**Scheme 2:**
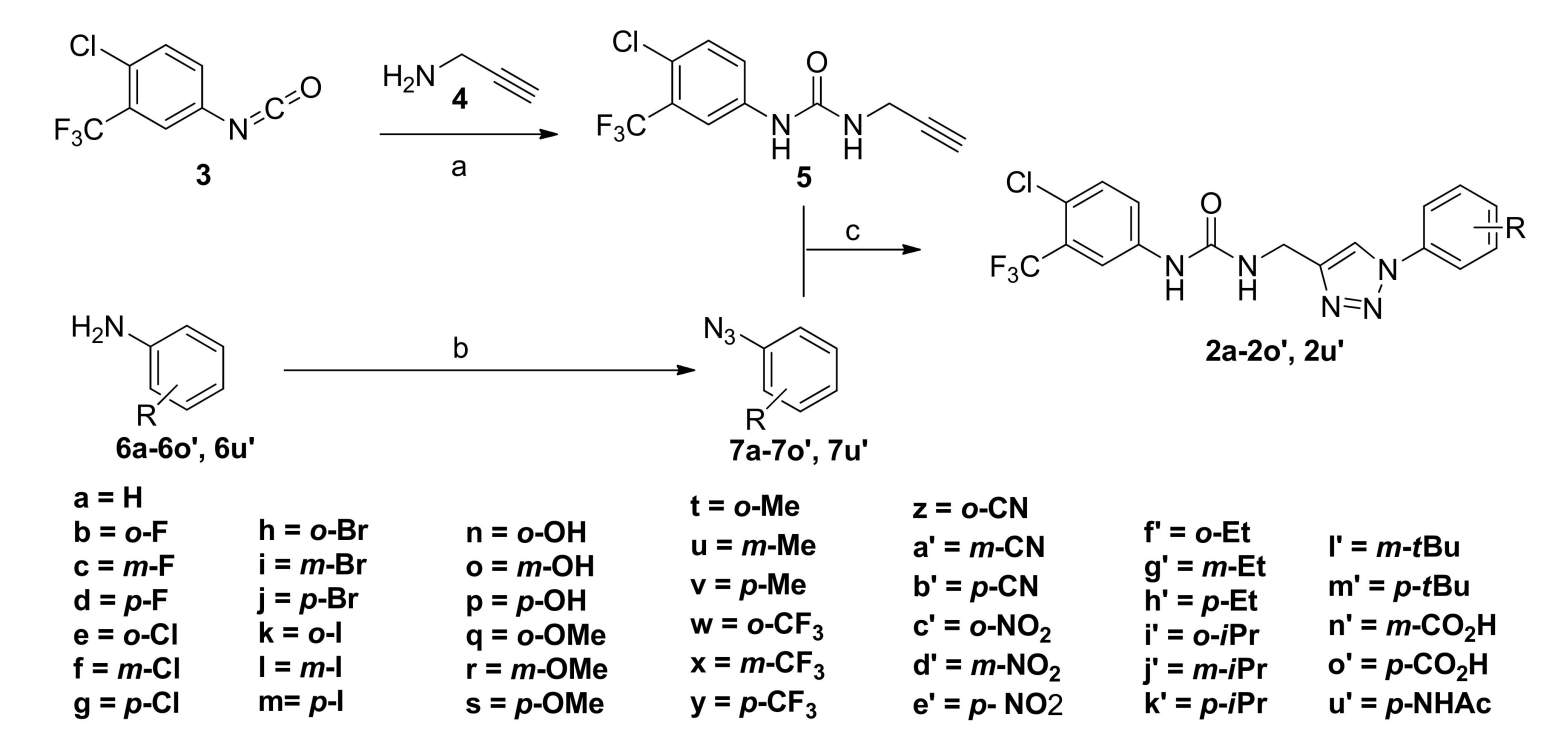
The synthesis route to triazole-cored analogs **2a**–**2o’**and **2u’.** Reagents and conditions: (a) dry CH_2_Cl_2_, rt, 6 h, 91%; (b) NaNO_2_ in 50% HCl at 0–5 °C, 30 min, then the addition of an aqueous solution of NaN_3_ and NaOAc at 0–5 °C, 30 min, 75%—quantitative yields; (c) CuSO_4_·5H_2_O (5 mol%), ascorbic acid (5 mol%), *n*-BuOH:H_2_O (1:1) at 65 °C, 2 h, 70%—quantitative yields.

**Scheme 3:**
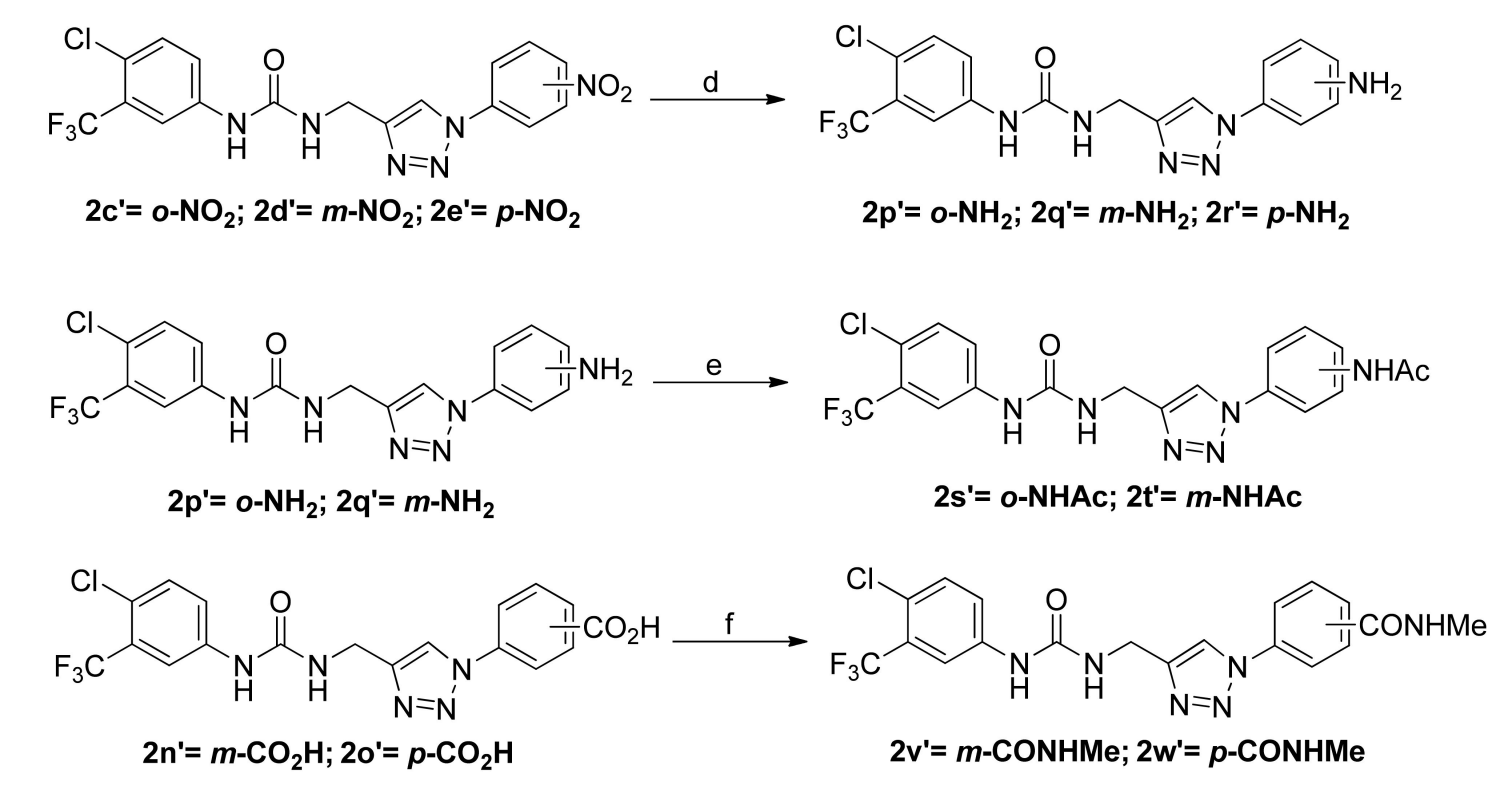
The functional group interconversions to 1,2,3-triazole derivatives **2p’**–**2t’** and **2v’**–**2w’**. Reagents and conditions: (d) SnCl_2_·2H_2_O, conc HCl, 0 °C to rt, then reflux at 65 °C, 3 h, 93–96%; (e) Ac_2_O, CH_2_Cl_2_:THF (1:1), rt, 10 min, 96–99%; (f) MeNH_2_·HCl, HATU, DIPEA, dry DMF, rt, 16 h, 72–77%.

The synthesis of forty-nine new triazole-cored analogs, **2a–2w’**, was accomplished in a few steps, as depicted in Scheme 2 and Scheme 3. Initially, 4-chloro-3-trifluoromethylphenyl isocyanate (**3**) underwent nucleophilic addition with propargyl amine (**4**) to obtain the ureido alkyne intermediate **5** in an excellent yield. In parallel, various substituted anilines **6a**–**6o’** and **6u’** were smoothly converted to the corresponding azides **7a**–**7o’** and **7u’** via diazotization using the procedure described previously [60]. Finally, the desired analogs **2a**–**2o’** and **2u’** were obtained by the construction of the 1,2,3-triazole through copper-catalyzed azide–alkyne cycloaddition (CuAAC) between alkyne **5** and azides **7a**–**7o’** and **7u’** with good to excellent yields.

Further functional group transformations were implemented to vary the substituents on the phenyl ring, as demonstrated in Scheme 3**.** The nitro analogs **2c’**–**2e’** were reduced using stannous (II) chloride under acidic conditions to give the corresponding anilines **2p’**–**2r’**. Subsequently, compounds **2p’** and **2q’** were acetylated to furnish the *N*-acetylated derivatives **2s’** and **2t’** in excellent yields. While the carboxylic acids **2n’** and **2o’** were coupled with methylamine using HATU to provide the amides **2v’** and **2w’** in good yields.

The precursors and the target analogs were characterized mainly by Nuclear Magnetic Resonance Spectroscopy (NMR) using ^1^H, ^13^C, ^19^F, and DEPT135 techniques, together with additional 2D NMR (COSY, HMQC, and HMBC) techniques in some cases. The formation of urea moiety was confirmed by the presence of two singlet peaks at δ = 6.31 and 8.48 ppm in the ^1^H NMR spectrum [61], while the generation of the azide intermediates **7a**–**7o’** and **7u’** was detected as a strong peak at approximately 2200–2000 cm^–1^ in infrared spectra (IR) [62]. After the coupling of alkyne **5** and azides **7**, a characteristic proton peak of the 1,2,3-triazole proton at approximately δ = 8.50–9.50 ppm [63] was observed, indicating the successful construction of the target triazole derivatives, which were confirmed complimentarily by High-Resolution Mass Spectrometry (HRMS).

### 2.2. Cytotoxicity toward Cancer Cell Lines

The cytotoxicity of the synthesized analogs toward HepG2, A549, HuCCA-1, MOLT-3, and HL-60 was evaluated compared to two positive controls, Sorafenib and Doxorubicin, by MTT or XTT assay [64]. The results revealed that the inhibitory effect of the synthesized triazole analogs was significantly more prominent toward HepG2 than the other cell lines tested. The inhibitory activities of the analogs against HepG2 are presented in Table 1, and the cytotoxicity toward other tested cancer cell lines is reported separately in the supporting information (Table S1).

The cytotoxicity screening of the analogs at the concentration of 25 µM toward HepG2 revealed that seven analogs, namely **2e**, **2f**, **2y**, **2i’**, **2k’**, **2l’**, and **2m’**, achieved cell viability percentages below 55%, indicating potent inhibitory activity. The cytotoxic activity of these compounds was then further explored to determine their half-maximal inhibitory activity (IC_50_). The results showed that **2e** (R = *o-*Cl), **2y** (R = *p-*CF_3_), **2i’** (R = *o-i*Pr), and **2m’** (R = *p-t*Bu) exhibited inhibitory activities with IC_50_ values of 5.02, 5.97, 5.40, and 5.57 µM, respectively, similar to that of Sorafenib (IC_50_ = 5.97 µM), whereas **2f** (R = *m-*Cl), **2l’** (R = *m-t*Bu), and **2k’** (R = *p-i*Pr) possessed moderate activity, with IC_50_ values of 9.81, 9.88, and 20.95 µM, respectively. However, all compounds were less active, when compared with the non-selective chemotherapy medication Doxorubicin (IC_50_ = 0.59 µM).

### 2.3. Structure–Activity Relationships (SARs)

The structure–activity relationships (SARs) suggested that the substituents on the phenyl ring connecting triazole had strong influences on anti-HepG2 activities. The analogs with a substituent capable of forming hydrogen bonds to the highly conserved hinge region of kinases, such as OH, OMe, CN, COOH, NH_2_, NHAc, and CONHAc, showed moderate to low anti-HepG2 activity at 25 µM. In contrast, the analogs with a hydrophobic group such as a methyl, ethyl, isopropyl, or *tert*-butyl group exhibited decreasing cell viability percentages in HepG2 cases, in accordance with the increase in the size of the substituent. Especially, **2i’** (R = *o*-*i*Pr) and **2m’** (R = *p*-*t*Bu) were capable of inhibiting HepG2 with similar IC_50_ values to that of Sorafenib, indicating that a bulky hydrophobic group might be an important group for the inhibition of HepG2. In spite of **2i’** (R = *o*-*i*Pr) and **2m’** (R = *p*-*t*Bu), it was found that compounds **2e** (R = *o*-Cl) and **2y** (R = *p*-CF_3_), which were substituted with an electron-withdrawing group, showed similar IC_50_ values against HepG2 to Sorafenib. The effect of the substituted position was not explicitly observed, nevertheless, the *o*- and *p*-substituted analogs tended to possess superior anti-HepG2 activities to *m*-substituted analogs, according to the structure and activity of the active analogs. Based on our results, it could be deduced that the analogs with a phenyl containing a functional group capable of forming a hydrogen bond, replacing picolinamide, might not be as suitable a mimic for picolinamide as we hypothesized.

### 2.4. Cytotoxicity toward MRC-5 Cells and Selectivity Index (SI)

Preliminarily, the safety property of the synthetic compounds was investigated by cytotoxicity assay against MRC-5 using MTT compared to Sorafenib and Doxorubicin. It was evident that almost all synthetic analogs showed IC_50_ values of more than 50 µM, which possessed approximately at least 2.5- and 22.1-fold less cytotoxic activity than Sorafenib (IC_50_ = 19.7 µM) and Doxorubicin (IC_50_ = 2.26 µM), respectively. The triazole analogs tended to show superior safety properties to the current approved therapeutic drugs. Thus, it could be implied that the presence of 1,2,3-triazole moiety can be one important factor in the compounds’ safety profile, which agrees with previous reports [4,45,65].

In addition to the promising safety property, some triazole-containing analogs exhibited an excellent selectivity index (SI) for HepG2, superior to Sorafenib and Doxorubicin. All active analogs, **2m’** (R = *p*-*t*Bu), **2e** (R = *o*-Cl), **2i’** (R = *o*-*i*Pr), and **2y** (R = *p*-CF_3_), which possessed similar inhibitory activities to Sorafenib, exhibited SI values of 14.7, 12.2, 10.1, and 9.81, respectively, which were up to 4.4-fold and 3.8-fold superior to those of Sorafenib (SI = 3.30) and Doxorubicin (SI = 3.83), respectively. The derivatives possessing SI ≥ 3.00 were considered safe and highly cancer-selective [64,66]. Moreover, the potent analogs showed significantly higher SIs than Sorafenib and Doxorubicin, implying that the synthetic analogs might be safer and, at this stage, more suitable for targeted HCC drug therapy.

Evidently, the synthetic triazole-cored analogs **2m’** (R = *p*-*t*Bu) and **2e** (R = *o*-Cl) were identified as active candidates toward HepG2 with the highest SIs. These compounds were further investigated for exploring the possible mechanisms of action underlying their cytotoxic effects by the analysis of cell-cycle arrest and apoptosis induction on HepG2 cells.

### 2.5. Cell-Cycle Analysis

**Figure 4:**
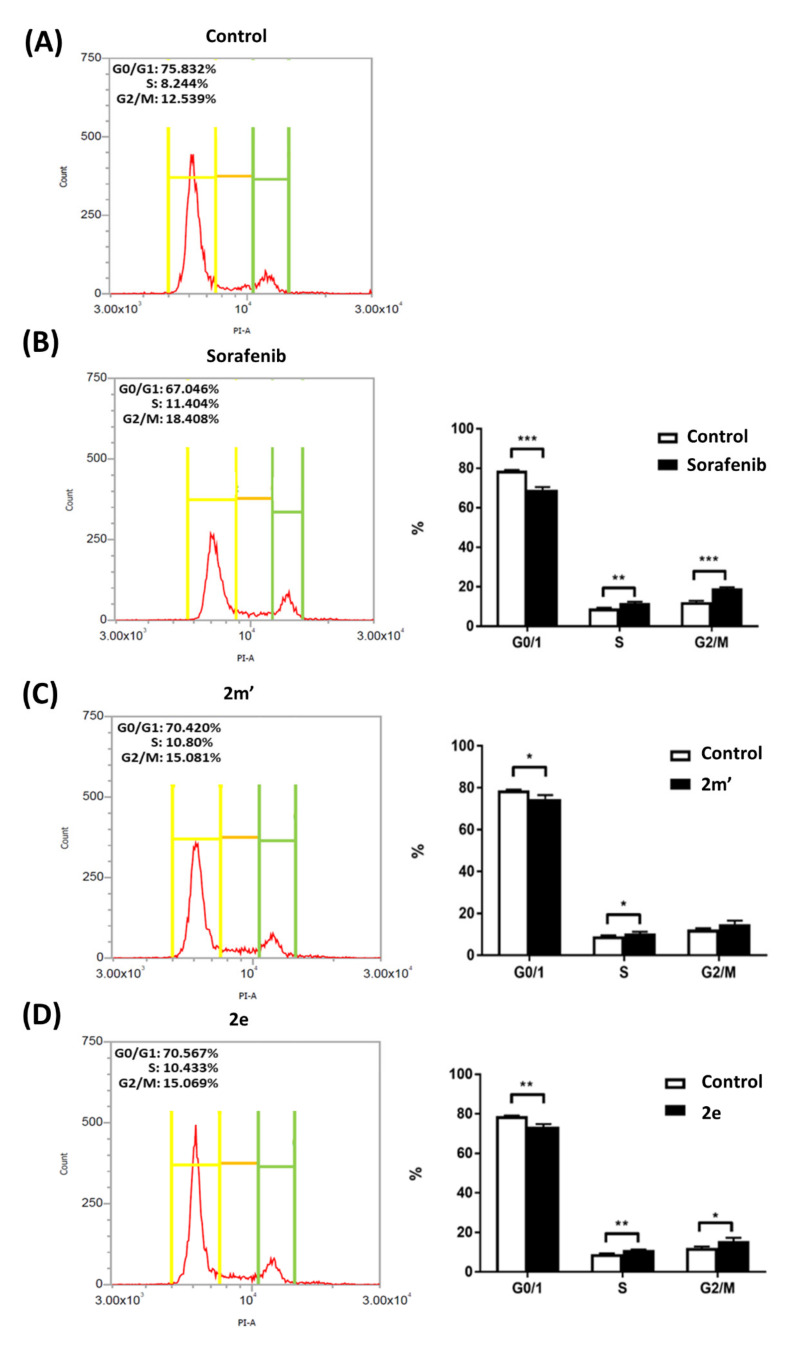
Cell-cycle distribution analysis of HepG2 cells treated with vehicle control (**A**), Sorafenib (**B**), compounds **2m’** (**C**), and **2e** (**D**) as described in Materials and Methods. * *p* < 0.05, ** *p* < 0.01, *** *p* < 0.001.

To further confirm the cytotoxic activity of **2m’** and **2e**, we sought to investigate the effect of these compounds on the cell-cycle distribution of HepG2 cancer cells. HepG2 treated with Sorafenib resulted in a decrease in cells in the G0/G1 phase and an increase in cells in the S and G2/M phases compared to the control (Figure 4A,B), suggesting that Sorafenib induced S and G2/M phase cell-cycle arrest. The effect observed was in line with a previous report by another group [67,68,69]. The selected compounds **2m’** and **2e** exhibited similar cell cycle profiles compared to Sorafenib (Figure 4C,D), indicating that the capability to induce S and G2/M phase cell-cycle arrest was recapitulated in these compounds. Given the similarity in chemical structure and biological activity of the synthesized compounds **2m’** and **2e** to their parent molecule Sorafenib, it is possible that these compounds share a similar mechanism of action.

### 2.6. Detection of Apoptosis

**Figure 5:**
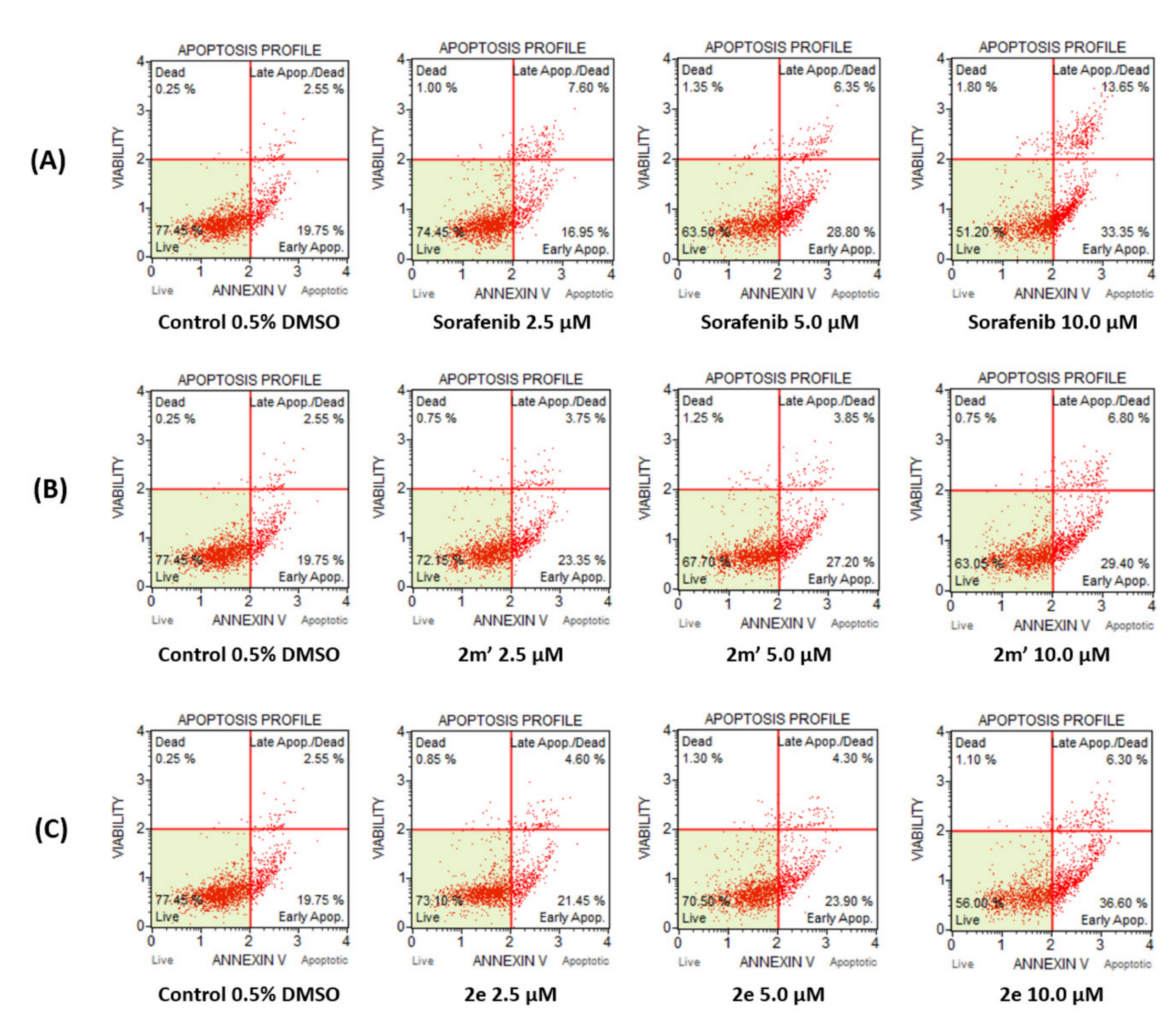
The detection of apoptosis, concentration-dependent for 48 h, by compounds (**A**) Sorafenib, (**B**) **2m’**, and (**C**) **2e** in HepG2 cells, compared with 0.5% DMSO as a control.

**Figure 6:**
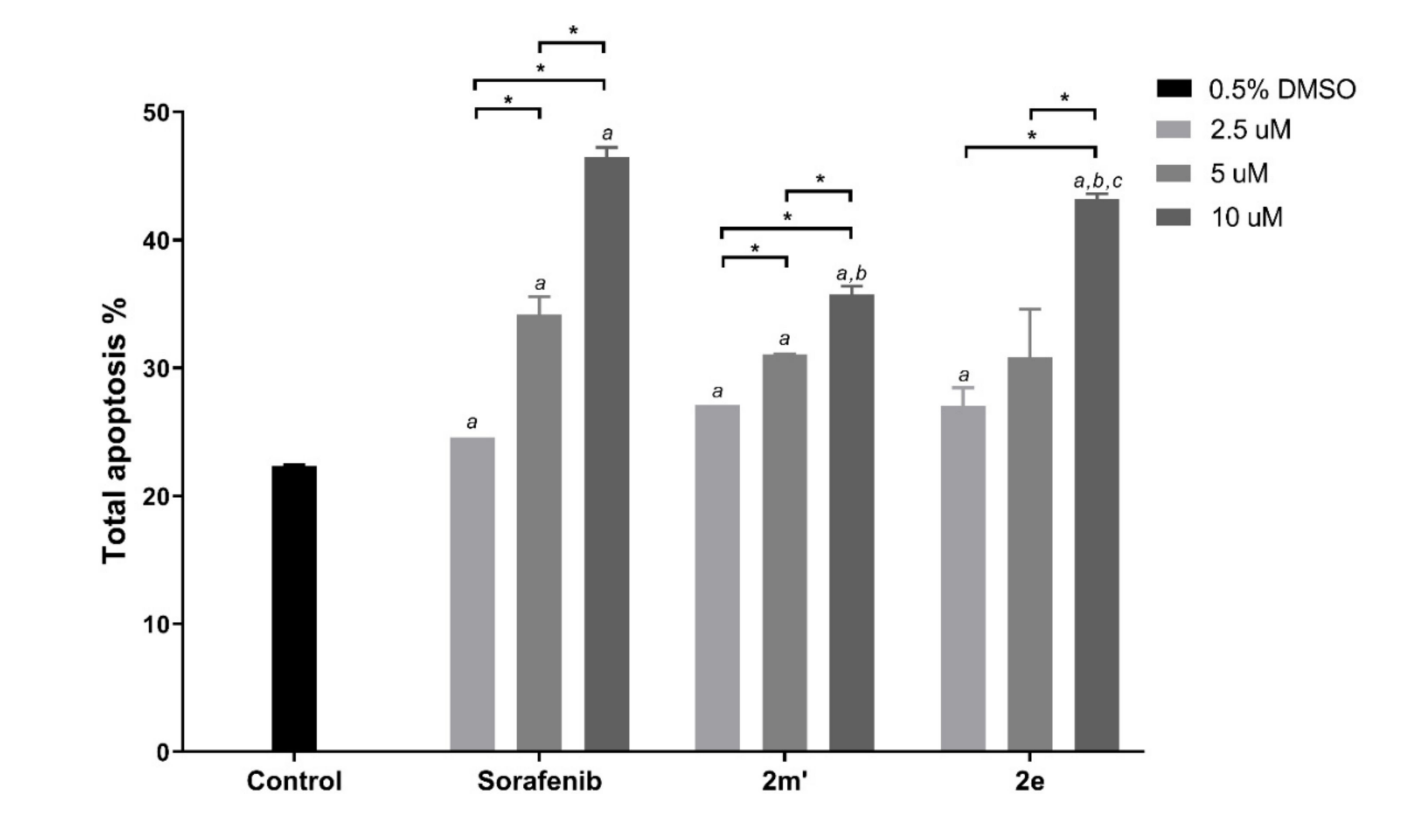
The comparison of the concentration-dependent total apoptosis of compounds **2m’** and **2e** at 48 h, compared with 0.5% DMSO as control and Sorafenib. Each treated group was compared using Student’s *t*-test (* *p* < 0.05 was considered statistically significant; *a* = compared with the control group, *b* = compared with the 10.0 µM Sorafenib group, *c* = compared with the 10.0 µM **2m’** group).

The induction of the apoptotic cell death of Sorafenib, **2m’**, and **2e** on HepG2 was quantified by Annexin V binding to phosphatidylserine (PS) on the outer cell surface using Muse’s Annexin V & Dead Cell Assay Kit. Based on annexin V reactivity and the intensity of the 7-AAD fluorescence, cells can be classified into four categories: dead, live, early apoptosis, and late apoptosis. HepG2 cell lines were treated for 48 h with 2.5 µM, 5.0 µM, and 10 μM of Sorafenib, **2m’**, and **2e**, and the effects on apoptosis are shown in Figure 5 and Figure 6. For the percentage of annexin V-PE-positive cells, the gradual increase in late stage apoptotic cells were 3.75%, 3.85%, and 6.80% for **2m’**, 4.60%, 4.30%, and 6.30% for **2e**, and 7.60, 6.35, and 13.65% for Sorafenib at 2.5 µM, 5.0 µM, and 10 µM, respectively, with a corresponding decrease in viable cells (Figure 5). Additionally, there was an increase in the total apoptotic cell population (early and late apoptosis) associated with higher doses of all three compounds. The average percentage of apoptotic cells after treatments at 2.5 µM, 5.0 µM, and 10.0 µM were 24.55 ± 0.28%, 35.15 ± 1.41%, and 46.45 ± 0.78% for Sorafenib; 27.10 ± 1.06%, 31.05 ± 0.04%, and 35.75 ± 0.67% for **2m’**; and 27.05 ± 1.41%, 30.85 ± 3.75%, and 43.20 ± 0.42% for **2e**. The basal apoptosis level in untreated cells was 22.35 ± 0.07%. Notably, there was a significant increase in total apoptosis at 10 µM for these three compounds with the highest percentage of apoptosis for Sorafenib. These results consistently indicated that **2m’**, **2e**, and especially Sorafenib exerted their cytotoxic effects through the induction of apoptosis, and compounds **2m’** and **2e** induced apoptosis in a dose-dependent manner similar to Sorafenib.

**Figure 7:**
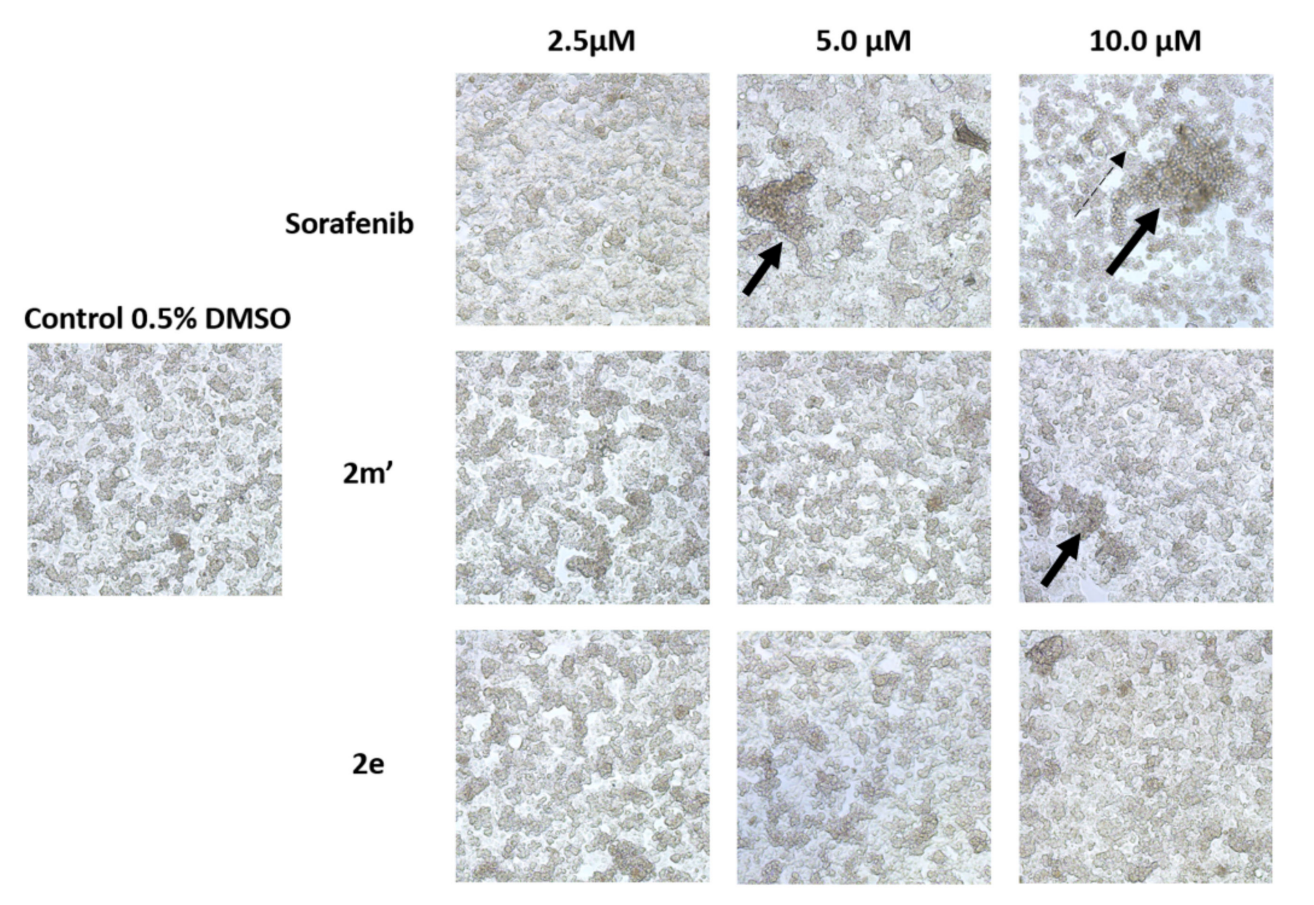
The cell morphology of HepG2 cells treated with different concentrations of compounds **2m’** and **2e** at 48 h, compared with 0.5% DMSO as a control and Sorafenib. The bold arrows show morphological changes with cell shrinkage and the dashed arrow shows cells smaller in size.

To observe the cell death of analogs and Sorafenib-treated hepatocellular carcinoma cells, the morphologies of HepG2 cells were compared to those of untreated control cells by using light microscopy. Notably, the cell morphology changes (shrink and smaller in size) were observed after treating HepG2 with Sorafenib at 5.0 µM and 10.0 µM, while analog **2e** maintained the morphology of intact cells after treatment at 48 h and the changes were observed when HepG2 was treated with **2m’** at 10.0 µM, as presented in Figure 7. These data suggest that the characteristics of cell apoptosis morphology changes, such as the shrinkage of the cells or the loss of cell volume, were observed in a dose-dependent manner after treatment with Sorafenib and **2m’**, which relate to the results of the cytotoxicity and apoptosis assays.

### 2.7. Physicochemical Properties and Lipinski’s Rule of Five

In order to evaluate the druggability of the potent triazole-containing analogs **2m’** and **2e** compared to Sorafenib, the physicochemical properties and analysis by Lipinski’s rule of five were conducted using the SwissADME website service [70]. All the calculated parameters are presented in Table 2. The results revealed that the synthetic compound **2m’** exhibited inferior physicochemical properties to Sorafenib. Although **2m’** had a similar molecular weight (MW), number of hydrogen bonds (nHBA and nHBD), and number of rotatable bonds (nRB) to Sorafenib, it exhibited inappropriate properties including higher lipophilicity (Clog P) and lower water solubility (Log S). Moreover, the violation of Lipinski’s rule was detected in the case of **2m’**, as its Clog P (4.74) was greater than or equal to the reported requirement (4.15) [71]. On the other hand, **2e** showed superior drug-likeness to **2m’** and Sorafenib. Analog **2e** possessed superior properties including lower molecular weight, more hydrophilicity, higher water solubility, and a smaller number of rotatable bonds, although **2e** had less hydrogen bonding positions (nHBA and nHBD) than Sorafenib. Additionally, compound **2e** possessed a topological polar surface area (TPSA) value of 71.84 Å^2^, which was slightly above the range attributed to most successful drugs (≤60–70 Å^2^) [72]. This value was much lower than that of Sorafenib (92.35 Å^2^), suggesting that **2e** tended to exhibit greater cell membrane permeability than Sorafenib [73]. According to the physicochemical parameters, **2e** exhibited appropriate druggability and conformed the Lipinski’s guidelines, thus tending to be a promising drug candidate for a targeted liver cancer agent.

## 3. Materials and Methods

### 3.1. Chemistry

#### 3.1.1. General

The 4-Chloro-3-trifluoromethylphenyl isocyanate (**3**) and propargyl amine (**4**) were purchased from Tokyo Chemical Industry (TCI). Commercial grade reagents and solvents were purchased from Tokyo Chemical Industry (TCI), Sigma-Aldrich, Arcos Organics, BDH chemicals, Riedel-de-Haen, Fluka, Carlo Erba, J.T. Baker, and RCI Labscan. They were analytical grade and used as received without purification. The solvent used in purifications was distilled prior to use. Silica gel F60 (Silicycle, Quebec City, Canada, 40–63 µm, 60 Å) was used for flash column chromatography, and Silica gel 60 F_254_ aluminum sheet (Merck) was used for preparative thin-layer chromatography (PTLC). All reactions and column chromatographic separations were monitored by TLC analysis using precoated silica gel 60 TLC sheets, visualized by 254 and 365 nm UV lamps. All products were characterized by nuclear magnetic resonance spectroscopy (NMR). The ^1^H NMR (300 MHz), ^13^C NMR (75 MHz), and ^19^F NMR (282 MHz) spectra were recorded on a Bruker AVANCE 300 using CDCl_3_, Acetone-*d_6_*, DMSO-*d_6_*, or MeOD*-d_4_* on 5 mm diameter tubes. The chemical shifts (δ) were reported in units of part per million (ppm). Coupling constants (*J*) were reported in Hertz (Hz) and refer to tetramethylsilane (TMS) as an internal standard (δ_H_ 0.00 ppm) or trifluoroacetic acid (CF_3_COOH) (δ_F_ -76.55 ppm) or residual signals of CDCl_3_ (δ_H_ 7.26 ppm, or δ_C_ 77.22 ppm), Acetone-*d_6_* (δ_H_ 2.05 ppm, or δ_C_ 29.84 and 206.26 ppm), DMSO-*d_6_* (δ_H_ 2.50 ppm, or δ_C_ 39.52 ppm), or MeOD*-d_4_* (δ_H_ 3.31 ppm, or δ_C_ 49.00 ppm). The signals were reported as follows: chemical shifts, multiplicity, and coupling constant. The multiplicities were given as s = singlet, d = doublet, t = triplet, q = quartet, dd = doublet of doublet, dt = doublet of triplet, td = triplet of doublet, tt = triplet of triplet, ddd = doublet of doublet of doublet, dddd = doublet of doublet of doublet of doublet, m = multiplet, br = broad. High-Resolution Mass Spectrometry (HRMS) was performed using an ESI and APCI ionization technique on a Bruker Daltonics MicroTOF spectrometer. Melting points were measured on a Büchi Melting Point B-545 apparatus.

#### 3.1.2. The Procedure for the Preparation of Ureido Alkyne **5**

The solution of 4-chloro-3-(trifluoromethyl)phenyl isocyanate (**3**) (2.79 g, 12.59 mmol, 1.05 eq) and propargyl amine (**4**) (767.9 µL, 11.90 mmol, 1.00 eq) in dry dichloromethane (0.15 M, 80.0 mL) was stirred at room temperature for 1 h. The reaction mixture was evaporated under reduced pressure to give a 1-(4-chloro-3-(trifluoromethyl)phenyl)-3-(prop-2-yn-1-yl)urea (**5**) (3.17 g, 91% yield) as a white solid which was used without further purification.

*1-(4-chloro-3-(trifluoromethyl)phenyl)-3-(prop-2-yn-1-yl)urea (**5**)*. mp = 122–123 °C; ^1^H NMR (300 MHz, acetone-*d_6_*) δ 3.07 (s, 1H), 4.02 (dd, 2H, *J* = 5.6 and 2.4 Hz), 6.31 (brs, 1H), 7.48 (d, 1H, *J* = 8.7 Hz), 7.68 (dd, 1H, *J* = 8.7 and 2.0 Hz), 8.08 (d, 1H, *J* = 2.2 Hz) and 8.48 (brs, 1H) ppm.; ^13^C NMR (75 MHz, acetone-*d_6_*) δ 155.4, 140.7, 132.6, 128.4 (q, J = 30.8 Hz), 123.9 (q, J = 270.8 Hz), 123.8, 123.4, 117.8 (q, *J* = 6.0 Hz), 81.8, 71.9 and 29.8 ppm.; ^19^F NMR (282 MHz, CDCl3) δ −63.59 (s, 3F) ppm.; HRMS (ESI+): *m/z* = 299.0169 [M+Na]^+^; calcd 299.0175 for [(C_11_H_8_ClF_3_N_2_O)+Na]^+^.

#### 3.1.3. General Procedure for the Preparation of Phenyl Azide **7a**-**7o’** and **7u’**

Phenyl azide **7a**-**7o’** and **7u’** were prepared according to the procedure described previously [60]. A stirred solution of aniline derivatives **6a**-**6o’** and **6u’** (1.00 eq) in 50% HCl (0.74 M) at 0 °C was treated dropwise with an aqueous solution of sodium nitrite (1.50 M, 3.00 eq) while maintaining the temperature of the reaction mixture below 0 °C. The reaction mixture was stirred at 0 °C for 30 min, then an aqueous solution of sodium azide (1.50 M, 2.00 eq) was added, followed by an aqueous solution of sodium acetate (1.50 M, 2.00 eq) at below 0 °C. After stirring for 30 min, the reaction mixture was diluted with water (80 mL) and extracted with dichloromethane (3 × 50 mL). The combined organic phase was washed with an aqueous sodium hydrogen carbonate solution (20 mL) dried over anhydrous sodium sulfate and filtered. The solvent was removed under reduced pressure to obtain phenyl azides **7a** to **7e’**, **7g’**, **7h’**, **7j’** to **7o’**, and **7u’**, which were used without purification. The crude products of phenyl azides **7f’** and **7i’** were further purified by column chromatography (silica gel, hexane). All phenyl azides are known [74,75,76,77,78,79,80,81,82,83,84,85,86,87,88]. The characteristics and spectroscopic data are given in the supporting information.

#### 3.1.4. General Procedure for the Preparation of Sorafenib Derivatives **2a**-**2o’** and **2u’**

Triazole-cored derivatives **2a**-**2o’** and **2u’** were prepared according to the procedure described previously [89]. To a stirred suspension of phenyl azides **2a**-**2o’** and **2u’** (0.70 mmol, 1.30 eq) and ureido alkyne 5 (150 mg, 0.54 mmol, 1.00 eq) in the mixture solvent of *n-*butanol and water (1:1; 0.14 M, 4.0 mL) and ascorbic acid (5 mg, 0.03 mmol, 0.05 eq), an aqueoeus 1M copper (II) sulfate pentahydrate solution (27 µL, 0.03 mmol, 0.05 eq) was added. The reaction mixture was stirred at 65 °C for 2 h, and then was diluted with water (5 mL), followed by a 10% *v/v* ammonium hydroxide solution (2.5 mL). After that, the resulting solution was extracted with ethyl acetate (3 × 50 mL). The combined organic phase was washed with brine (20 mL), dried over anhydrous sodium sulfate, and filtered. The solvent was removed under reduced pressure and the crude product was purified by column chromatography (silica gel, ethyl acetate in hexane) to obtain the products **2a**-**2o’** and **2u’**.

*1-(4-chloro-3-(trifluoromethyl)phenyl)-3-((1-phenyl-1H-1,2,3-triazol-4-yl)methyl)urea (**2a**)*. The crude residue was pre-absorbed on silica gel and purified by flash silica gel column chromatography using a mixture of 50% ethyl acetate in hexane as an eluent to provide (209 mg, 98% yield) the compound **2a** as an orange-yellow solid; mp = 168–169 °C; ^1^H NMR (300 MHz, DMSO*-d_6_*) δ 4.45 (d, 2H, *J* = 3.0 Hz), 6.91 (brt, 1H, *J* = 5.7 Hz), 7.48 (tt, 1H, *J* = 6.6 and 1.2 Hz), 7.55 (d, 1H, *J* = 8.8 Hz), 7.57–7.64 (m, 3H), 7.89 (dd, 2H, *J* = 8.3 and 1.2 Hz), 8.09 (d, 1H, *J* = 2.2 Hz), 8.67 (s, 1H) and 9.13 (s, 1H) ppm.; ^13^C NMR(75 MHz, DMSO*-d_6_*) δ 155.0, 146.7, 140.1, 136.8, 132.0, 130.1 (x2), 128.8, 126.8 (q, *J* = 30.8 Hz), 123.0 (q, *J* = 271.5 Hz), 122.7, 121.8 (q, *J* = 1.5 Hz), 121.1, 120.2 (2C), 116.5 (q, *J* = 6.0 Hz) and 35.0 ppm.; ^19^F NMR (282 MHz, DMSO*-d_6_*) δ −63.41 (s, 3F) ppm.; HRMS (ESI+): *m/z* = 396.0838 [M+H]^+^; calcd 396.0839 for [(C_17_H_13_ClF_3_N_5_O)+H]^+^.

*1-(4-chloro-3-(trifluoromethyl)phenyl)-3-((1-(2-fluorophenyl)-1H-1,2,3-triazol-4-yl)methyl)urea (**2b**).* The crude residue was recrystallized using acetone as an solvent to provide (174 mg, 78% yield) the compound **2b** as a white solid; mp = 168–169 °C; ^1^H NMR (300 MHz, DMSO*-d_6_*) δ 4.44 (d, 2H, *J* = 5.6 Hz), 6.92 (brt, 1H, *J* = 5.7 Hz), 7.34–7.61 (m, 5H), 7.77 (td, 1H, *J* = 7.8 and 1.6 Hz), 8.00 (d, 1H *J* = 2.2 Hz), 8.35 (d, 1H, *J* = 2.1 Hz) and 9.12 (s, 1H) ppm.; ^13^C NMR (75 MHz, DMSO*-d_6_*) δ 154.9, 153.8 (d, *J* = 248.3 Hz), 146.1, 140.0, 131.9, 131.2 (d, *J* = 7.5 Hz), 126.7 (q, *J* = 30.8 Hz), 125.9, 125.6 (d, *J* = 3.8 Hz), 124.8 (d, *J* = 10.5 Hz), 124.3 (d, *J* = 4.5 Hz), 122.9 (q, *J* = 271.5 Hz), 122.5, 121.6 (q, *J* = 2.3 Hz), 117.4 (d, *J* = 19.5 Hz), 116.3 (q, *J* = 6.0 Hz) and 34.8 ppm.; ^19^F NMR (282 MHz, DMSO*-d_6_*) δ −63.31 (s, 3F) and -125.54 (s, 1F) ppm.; HRMS (ESI+): *m/z* = 414.0734 [M+H]^+^; calcd 414.0745 for [(C_17_H_13_ClF_4_N_5_O)+H]^+^.

*1-(4-chloro-3-(trifluoromethyl)phenyl)-3-((1-(3-fluorophenyl)-1H-1,2,3-triazol-4-yl)methyl)urea (**2c**)*. The crude residue was pre-absorbed on silica gel and purified by flash silica gel column chromatography using a mixture of 60% ethyl acetate in hexane as an eluent to provide (206 mg, 92% yield) the compound **2c** as a white solid; mp = 182–183 °C; ^1^H NMR (300 MHz, DMSO*-d_6_*) δ 4.44 (d, 2H, *J* = 5.5 Hz), 6.93 (brt, 1H, *J* = 5.6 Hz), 7.33 (tdd, 1H, *J* = 8.6, 2.5 and 0.81 Hz), 7.54 (d, 1H, *J* = 8.8 Hz), 7.59 (dd, 1H, *J* = 8.9 and 2.3 Hz), 7.63 (ddd, 1H, *J* = 8.3, 8.3 and 6.3 Hz), 7.80 (ddd, 1H, *J* = 8.1, 2.0 and 0.81 Hz), 7.85 (dt, 1H, *J* = 10.1 and 2.2 Hz), 8.10 (d, 1H, *J* = 2.3 Hz), 8.72 (s, 1H), 9.16 (s, 1H) ppm.; ^13^C NMR (75 MHz, DMSO*-d_6_*) δ 162.5 (d, *J* = 243.8 Hz), 154.8, 146.8, 140.0, 137.9 (d, *J* = 10.5 Hz), 131.9, 131.8 (d, *J* = 8.3 Hz), 126.6 (q, *J* = 30.0 Hz), 122.9 (q, *J* = 271.5 Hz), 122.5, 121.6, 121.1, 116.3 (q, *J* = 6.0 Hz), 115.9 (d, *J* = 3.0 Hz), 115.3 (d, *J* = 20.3 Hz), 107.5 (d, *J* = 26.3 Hz) and 34.8 ppm.; ^19^F NMR (282 MHz, DMSO*-d_6_*) δ −63.12 (s, 3F) and -112.29 (s, 1F) ppm.; HRMS (ESI+): *m/z* = 414.0743 [M+H]^+^; calcd 414.0745 for [(C_17_H_13_ClF_4_N_5_O)+H]^+^.

*1-(4-chloro-3-(trifluoromethyl)phenyl)-3-((1-(4-fluorophenyl)-1H-1,2,3-triazol-4-yl)methyl)urea (**2d**)*. The crude residue was pre-absorbed on silica gel and purified by flash silica gel column chromatography using a mixture of 60% ethyl acetate in hexane as an eluent to provide (179 mg, 80% yield) the compound **2d** as a white solid; mp = 189–190 °C; ^1^H NMR (300 MHz, DMSO*-d_6_*) δ 4.45 (d, 2H, *J* = 5.6 Hz), 6.91 (brt, 1H, *J* = 5.6 Hz), 7.42 (t, 2H, *J* = 8.8 Hz), 7.53 (d, 1H, *J* = 8.8 Hz), 7.58 (dd, 1H, *J* = 8.8 and 2.3 Hz), 7.94 (dd, 2H, *J* = 13.8 and 9.1 Hz), 8.09 (d, 1H, *J* = 2.3 Hz), 8.65 (s, 1H) and 9.13 (s, 1H) ppm.; ^13^C NMR (75 MHz, DMSO*-d_6_*) δ 161.6 (d, *J* = 244.5 Hz), 154.8, 146.6, 140.0, 133.2 (d, *J* = 3.0 Hz), 131.8, 126.6 (q, *J* = 30.8 Hz), 122.9 (q, *J* = 271.5 Hz), 122.4, 122.3 (2C, d, *J* = 9.0 Hz), 121.5 (q, *J* = 1.5 Hz), 121.2, 116.5 (2C, d, *J* = 22.5 Hz), 116.2 (q, *J* = 5.3 Hz) and 34.84 ppm.; ^19^F NMR (282 MHz, DMSO*-d_6_*) δ −63.53 (s, 3F) and -115.40 (s, 1F) ppm.; HRMS (ESI+): *m/z* = 414.0733 [M+H]^+^; calcd 414.0745 for [(C_17_H_13_ClF_4_N_5_O)+H]^+^.

*1-(4-chloro-3-(trifluoromethyl)phenyl)-3-((1-(2-chlorophenyl)-1H-1,2,3-triazol-4-yl)methyl)urea (**2e**)*. The crude residue was pre-absorbed on silica gel and purified by flash silica gel column chromatography using a mixture of 40% ethyl acetate in hexane as an eluent to provide (211 mg, 91% yield) the compound **2e** as a beige-colored solid; mp = 147–148 °C; ^1^H NMR (300 MHz, DMSO*-d_6_*) δ 4.47 (d, 2H, *J* = 5.4 Hz), 6.93 (brt, 1H, *J* = 5.6 Hz), 7.51–7.61 (m, 4H), 7.65 (td, 1H, *J* = 7.5 and 2.1 Hz), 7.76 (dd, 1H, *J* = 7.5 and 1.8 Hz), 8.08 (d, 1H, *J* = 2.3 Hz), 8.40 (s, 1H) and 9.14 (s, 1H) ppm.; ^13^C NMR (75 MHz, DMSO*-d_6_*) δ 154.9, 145.5, 140.0, 134.6, 131.9, 131.6, 130.6, 128.5 (2C), 128.4, 126.7 (q, *J* = 30.0 Hz), 124.9, 122.9 (q, *J* = 271.5 Hz), 122.5, 121.6 (q, *J* = 2.3 Hz), 116.3 (q, *J* = 5.3 Hz) and 34.8 ppm.; ^19^F NMR (282 MHz, DMSO*-d_6_*) δ −63.17 (s, 3F) ppm.; HRMS (ESI+): *m/z* = 430.0429 [M+H]^+^; calcd 430.0449 for [(C_17_H_13_Cl_2_F_3_N_5_O)+H]^+^.

*1-(4-chloro-3-(trifluoromethyl)phenyl)-3-((1-(3-chlorophenyl)-1H-1,2,3-triazol-4-yl)methyl)urea (**2f**)*. The crude residue was pre-absorbed on silica gel and purified by flash silica gel column chromatography using a mixture of 40% ethyl acetate in hexane as an eluent to provide (200 mg, 86% yield) the compound **2f** as a beige-colored solid; mp = 163–164 °C; ^1^H NMR (300 MHz, DMSO*-d_6_*) δ 4.45 (d, 2H, *J* = 5.5 Hz), 6.93 (brt, 1H, *J* = 5.6 Hz), 7.51–7.60 (m, 3H), 7.61 (t, 1H, *J* = 8.1 Hz), 7.92 (ddd, 1H, *J* = 7.8, 2.1 and 1.2 Hz), 8.05 (t, 1H, *J* = 2.1 Hz), 8.10 (d, 1H, *J* = 2.1 Hz), 8.76 (s, 1H) and 9.15 (s, 1H) ppm.; ^13^C NMR (75 MHz, DMSO*-d_6_*) δ 154.8, 146.8, 140.0, 137.7, 134.2, 131.8, 131.6, 128.3, 126.6 (q, *J* = 30.0 Hz), 122.9 (q, *J* = 273.0 Hz), 122.4, 121.6 (q, *J* = 2.3 Hz), 121.1, 119.7, 118.5, 116.2 (q, *J* = 6.0 Hz) and 34.9 ppm.; ^19^F NMR (282 MHz, DMSO*-d_6_*) δ −63.06 (s, 3F) ppm.; HRMS (ESI+): *m/z* = 430.0397 [M+H]^+^; calcd 430.0449 for [(C_17_H_13_Cl_2_F_3_N_5_O)+H]^+^.

1*-(4-chloro-3-(trifluoromethyl)phenyl)-3-((1-(4-chlorophenyl)-1H-1,2,3-triazol-4-yl)methyl)urea (**2g**)*. The crude residue was pre-absorbed on silica gel and purified by flash silica gel column chromatography using a mixture of 50% ethyl acetate in hexane as an eluent to provide (230 mg, 99% yield) the compound **2g** as a white solid; mp = 203–204 °C; ^1^H NMR (300 MHz, DMSO*-d_6_*) δ 4.44 (d, 2H, *J* = 5.5 Hz), 6.93 (brt, 1H, *J* = 5.5 Hz), 7.55 (d, 1H, *J* = 8.8 Hz), 7.58 (dd, 1H, *J* = 8.9 and 2.2 Hz), 7.66 (d, 2H, *J* = 8.9 Hz), 7.95 (d, 2H, *J* = 8.9 Hz), 8.10 (d, 1H, *J* = 2.0 Hz), 8.70 (s, 1H) and 9.16 (s, 1H) ppm.; ^13^C NMR (75 MHz, DMSO*-d_6_*) δ 154.8, 146.7, 140.0, 135.5, 132.8, 131.8, 129.8 (2C), 126.6 (q, *J* = 30.0 Hz), 122.9 (q, *J* = 271.5 Hz), 122.4, 121.6 (2C), 121.6 (q, *J* = 1.5 Hz), 121.0, 116.3 (q, *J* = 6.0 Hz) and 34.8 ppm.; ^19^F NMR (282 MHz, DMSO*-d_6_*) δ −63.10 (s, 3F) ppm.; HRMS (ESI+): *m/z* = 430.0445 [M+H]^+^; calcd 430.0449 for [(C_17_H_13_Cl_2_F_3_N_5_O)+H]^+^.

*1-((1-(2-bromophenyl)-1H-1,2,3-triazol-4-yl)methyl)-3-(4-chloro-3-(trifluoromethyl)phenyl)urea (**2h**)*. The crude residue was pre-absorbed on silica gel and purified by flash silica gel column chromatography using a mixture of 50% ethyl acetate in hexane as an eluent to provide (231 mg, 90% yield) the compound **2h** as a beige-colored crystal solid; mp = 144–145 °C; ^1^H NMR (300 MHz, DMSO*-d_6_*) δ 4.47 (d, 2H, *J* = 5.3 Hz), 6.92 (brt, 1H, *J* = 5.4 Hz), 7.50–7.64 (m, 5H), 7.90 (brd, 1H, *J* = 7.5 Hz), 8.08 (d, 1H, *J* = 2.1 Hz), 8.37 (s, 1H) and 9.14 (s, 1H) ppm.; ^13^C NMR (75 MHz, DMSO*-d_6_*) δ 155.3, 145.8, 140.1, 136.7, 134.1, 132.3 (2C), 129.4, 129.1, 127.1 (q, *J* = 30.0 Hz), 125.3, 123.3 (q, *J* = 270.8 Hz), 122.9, 122.0 (q, *J* = 1.5 Hz), 119.2, 116.7 (q, *J* = 5.3 Hz) and 35.2 ppm.; ^19^F NMR (282 MHz, DMSO*-d_6_*) δ −63.07 (s, 3F) ppm.; HRMS (ESI+): *m/z* = 473.9886 [M+H]^+^; calcd 473.9944 for [(C_17_H_13_BrClF_3_N_5_O)+H]^+^.

*1-((1-(3-bromophenyl)-1H-1,2,3-triazol-4-yl)methyl)-3-(4-chloro-3-(trifluoromethyl)phenyl)urea (**2i**)*. The crude residue was pre-absorbed on silica gel and purified by flash silica gel column chromatography using a mixture of 50% ethyl acetate in hexane as an eluent to provide (256 mg, 100% yield) the compound **2i** as a white solid; mp = 160–161 °C; ^1^H NMR (300 MHz, DMSO*-d_6_*) δ 4.45 (d, 2H, *J* = 5.4 Hz), 6.92 (brt, 1H, *J* = 5.4 Hz), 7.53 (t, 1H, *J* = 8.1 Hz), 7.55 (d, 1H, *J* = 8.7 Hz), 7.59 (dd, 1H, *J* = 9.0 and 2.4 Hz), 7.68 (brdd, 1H, *J* = 8.1 and 0.9 Hz), 7.95 (brdd, 1H, *J* = 8.1 and 1.2Hz), 8.10 (d, 1H, *J* = 2.1 Hz), 8.17 (t, 1H, *J* = 1.8 Hz), 8.76 (s, 1H) and 9.15 (s, 1H) ppm.; ^13^C NMR (75 MHz, DMSO*-d_6_*) δ 154.8, 146.8, 140.0, 137.8, 131.8, 131.7 (2C), 131.2, 126.6 (q, *J* = 30.0 Hz), 122.9 (q, *J* = 271.5 Hz), 122.5 (2C), 121.6 (q, *J* = 2.3 Hz), 121.1, 118.9, 116.4 (q, *J* = 6.8 Hz) and 34.8 ppm.; ^19^F NMR (282 MHz, DMSO*-d_6_*) δ −63.07 (s, 3F) ppm.; HRMS (ESI+): *m/z* = 473.9881 [M+H]^+^; calcd 473.9944 for [(C_17_H_13_BrClF_3_N_5_O)+H]^+^.

*1-((1-(4-bromophenyl)-1H-1,2,3-triazol-4-yl me-thyl)-3-(4-chloro-3-(trifluoromethyl)phenyl)urea (**2j**)*. The crude residue was pre-absorbed on silica gel and purified by flash silica gel column chromatography using a mixture of 50% ethyl acetate in hexane as an eluent to provide (223 mg, 87% yield) the compound **2j** as a white solid; mp = 206–207 °C; ^1^H NMR (300 MHz, DMSO*-d_6_*) δ 4.44 (d, 2H, *J* = 5.5 Hz), 6.93 (brt, 1H, *J* = 5.6 Hz), 7.54 (d, 1H, *J* = 8.8 Hz), 7.58 (dd, 1H, *J* = 8.9 and 2.3 Hz), 7.78 (d, 2H, *J* = 9.0 Hz), 7.88 (d, 2H, *J* = 9.0 Hz), 8.09 (d, 1H, *J* = 2.2 Hz), 8.70 (s, 1H) and 9.16 (s, 1H) ppm.; ^13^C NMR (75 MHz, DMSO*-d_6_*) δ 154.8, 146.8, 140.0, 135.9, 132.7 (2C), 131.8, 126.6 (q, *J* = 30.8 Hz), 122.9 (q, *J* = 271.5 Hz), 122.4, 121.9 (2C), 121.5, 121.2, 121.0, 116.3 (q, *J* = 5.3 Hz) and 34.8 ppm.; ^19^F NMR (282 MHz, DMSO*-d_6_*) δ −63.13 (s, 3F) ppm.; HRMS (ESI+): *m/z* = 473.9939 [M+H]^+^; calcd 473.9944 for [(C_17_H_13_BrClF_3_N_5_O)+H]^+^.

*1-(4-chloro-3-(trifluoromethyl)phenyl)-3-((1-(2-iodophenyl)-1H-1,2,3-triazol-4-yl)methyl)urea (**2k**).* The crude residue was pre-absorbed on silica gel and purified by flash silica gel column chromatography using a mixture of 50% ethyl acetate in hexane as an eluent to provide (197 mg, 70% yield) the compound **2k** as a light-yellow solid; mp = 165–166 °C; ^1^H NMR (300 MHz, DMSO*-d_6_*) δ 4.47 (d, 2H, *J* = 5.3 Hz), 6.93 (brt, 1H, *J* = 5.3 Hz), 7.35 (td, 1H, *J* = 7.7 and 1.5 Hz), 7.46–7.65 (m, 4H), 8.08 (d, 1H, *J* = 6.5 Hz), 8.09 (d, 1H, *J* = 2.5 Hz), 8.32 (s, 1H) and 9.16 (s, 1H) ppm.; ^13^C NMR (75 MHz, DMSO*-d_6_*) δ 154.7, 145.3, 140.0, 139.8, 139.7, 131.8, 131.7, 129.3, 127.9, 126.6 (q, *J* = 30.8 Hz), 124.5, 122.8 (q, *J* = 270.8 Hz), 122.4, 121.6, 116.3 (q, *J* = 6.0 Hz), 95.4 and 34.8 ppm.; ^19^F NMR (282 MHz, DMSO*-d_6_*) δ −63.05 (s, 3F) ppm.; HRMS (ESI+): *m/z* = 521.9738 [M+H]^+^; calcd 521.9805 for [(C_17_H_12_ClF_3_IN_5_O)+H]^+^.

*1-(4-chloro-3-(trifluoromethyl)phenyl)-3-((1-(3-iodophenyl)-1H-1,2,3-triazol-4-yl)methyl)urea (**2l**)*. The crude residue was pre-absorbed on silica gel and purified by flash silica gel column chromatography using a mixture of 40% ethyl acetate in hexane as an eluent to provide (259 mg, 92% yield) the compound **2l** as a beige-color solid; mp = 164–165 °C; ^1^H NMR (300 MHz, DMSO*-d_6_*) δ 4.44 (d, 2H, *J* = 5.4 Hz), 6.92 (brt, 1H, *J* = 5.4 Hz), 7.36 (dd, 1H, *J* = 8.1 and 7.8 Hz), 7.54 (d, 1H, *J* = 9.0 Hz), 7.59 (dd, 1H, *J* = 9.0 and 2.4 Hz), 7.83 (ddd, 1H, *J* = 7.8, 1.5 and 0.9 Hz), 7.95 (ddd, 1H, *J* = 8.1, 2.1 and 0.6 Hz), 8.10 (d, 1H, *J* = 2.4 Hz), 8.28 (dd, 1H, *J* = 2.1 and 1.5 Hz), 8.71 (s, 1H) and 9.14 (s, 1H) ppm.; ^13^C NMR (75 MHz, DMSO*-d_6_*) δ 154.8, 146.8, 140.0, 137.6, 137.2, 131.9, 131.7, 128.0, 126.6 (q, *J* = 30.0 Hz), 122.9 (q, *J* = 271.5 Hz), 122.5, 121.6 (q, *J* = 2.3 Hz), 121.1, 119.3, 116.3 (q, *J* = 6.0 Hz), 95.4 and 34.8 ppm.; ^19^F NMR (282 MHz, DMSO*-d_6_*) δ −63.07 (s, 3F) ppm.; HRMS (ESI+): *m/z* = 521.9737 [M+H]^+^; calcd 521.9805 for [(C_17_H_12_ClF_3_IN_5_O)+H]^+^.

*1-(4-chloro-3-(trifluoromethyl)phenyl)-3-((1-(4-iodophenyl)-1H-1,2,3-triazol-4-yl)methyl)urea (**2m**)*. The crude residue was pre-absorbed on silica gel and purified by flash silica gel column chromatography using a mixture of 40% ethyl acetate in hexane as an eluent to provide (251 mg, 89% yield) the compound **2m** as a yellow solid; mp = 195–196 °C; ^1^H NMR (300 MHz, DMSO*-d_6_*) δ 4.44 (d, 2H, *J* = 5.5 Hz), 6.92 (brt, 1H, *J* = 5.5 Hz), 7.54 (d, 1H, *J* = 8.8 Hz), 7.58 (dd, 1H, *J* = 8.9 and 2.1 Hz), 7.73 (d, 2H, *J* = 8.7 Hz), 7.94 (d, 2H, *J* = 8.7 Hz), 8.09 (d, 1H, *J* = 2.0 Hz), 8.68 (s, 1H) and 9.15 (s, 1H) ppm.; ^13^C NMR (75 MHz, DMSO*-d_6_*) δ 154.9, 146.8, 140.0, 138.6 (2C), 136.4, 131.9, 126.6 (q, *J* = 30.0 Hz), 122.9 (q, *J* = 271.5 Hz), 122.5, 121.9 (2C), 121.6 (q, *J* = 2.3 Hz), 120.9, 116.3 (q, *J* = 5.3 Hz), 94.1 and 34.9 ppm.; ^19^F NMR (282 MHz, DMSO*-d_6_*) δ −63.26 (s, 3F) ppm.; HRMS (ESI+): *m/z* = 521.9803 [M+H]^+^; calcd 521.9805 for [(C_17_H_12_ClF_3_IN_5_O)+H]^+^.

*1-(4-chloro-3-(trifluoromethyl)phenyl)-3-((1-(2-hydroxyphenyl)-1H-1,2,3-triazol-4-yl)methyl)urea (**2n**).* The crude residue was pre-absorbed on silica gel and purified by flash sil-ica gel column chromatography using a mixture of 50% ethyl acetate in hexane as an el-uent to provide (220 mg, 99% yield) the compound **2n** as a brown-red solid; mp = 202–203 °C; ^1^H NMR (300 MHz, DMSO*-d_6_*) δ 4.44 (d, 2H, *J* = 5.4 Hz), 6.90 (brt, 1H, *J* = 5.5 Hz), 6.98 (td, 1H, *J* = 8.0 and 1.0 Hz), 7.10 (dd, 1H, *J* = 8.2 and 0.9 Hz), 7.33 (td, 1H, *J* = 8.3 and 1.6 Hz), 7.54 (d, 1H, *J* = 8.9 Hz), 7.58 (dd, 2H, *J* = 7.9 and 1.6 Hz), 8.07 (d, 1H, *J* = 2.2 Hz), 8.33 (s, 1H), 9.12 (s, 1H) and 10.53 (brs, 1H) ppm.; ^13^C NMR (75 MHz, DMSO*-d_6_*) δ 154.9, 149.5, 145.1, 140.0, 131.9, 130.0, 126.7 (q, *J* = 30.0 Hz), 125.1, 124.6, 124.2, 122.9 (q, *J* = 271.5 Hz), 122.4, 121.6 (q, *J* = 1.5 Hz), 119.6, 117.1, 116.3 (q, *J* = 6.0 Hz) and 34.9 ppm.; ^19^F NMR (282 MHz, DMSO*-d_6_*) δ −63.28 (s, 3F) ppm.; HRMS (ESI+): *m/z* = 412.0777 [M+H]^+^; calcd 412.0788 for [(C_17_H_13_ClF_3_N_5_O_2_)+H]^+^.

*1-(4-chloro-3-(trifluoromethyl)phenyl)-3-((1-(3-hydroxyphenyl)-1H-1,2,3-triazol-4-yl)methyl)urea (**2o**).* The crude residue was pre-absorbed on silica gel and purified by flash sil-ica gel column chromatography using a mixture of 50% ethyl acetate in hexane as an el-uent to provide (220 mg, 99% yield) the compound **2o** as a white solid; mp = 231–232 °C; ^1^H NMR (300 MHz, DMSO*-d_6_*) δ 4.43 (d, 2H, *J* = 5.4 Hz), 6.86 (ddd, 1H, *J* = 8.8, 2.2 and 1.1 Hz), 6.90 (brt, 1H, *J* = 5.6 Hz), 7.24–7.35 (m, 2H), 7.36 (t, 1H, *J* = 8.5 Hz), 7.54 (d, 1H, *J* = 8.8 Hz), 7.59 (dd, 1H, *J* = 8.9 and 2.3 Hz), 8.09 (d, 1H, *J* = 2.2 Hz), 8.59 (s, 1H), 9.13 (s, 1H) and 10.00 (brs, 1H) ppm.; ^13^C NMR (75 MHz, DMSO*-d_6_*) δ 158.5, 154.8, 146.4, 140.0, 137.7, 131.9, 130.8, 126.7 (q, *J* = 30.8 Hz), 122.9 (q, *J* = 271.5 Hz), 122.5, 121.6 (q, *J* = 2.3 Hz), 120.9, 116.3 (q, *J* = 6.0 Hz), 115.6, 110.4, 107.0 and 34.9 ppm.; ^19^F NMR (282 MHz, DMSO*-d_6_*) δ −63.23 (s, 3F) ppm.; HRMS (ESI+): *m/z* = 412.0783 [M+H]^+^; calcd 412.0788 for [(C_17_H_13_ClF_3_N_5_O_2_)+H]^+^.

*1-(4-chloro-3-(trifluoromethyl)phenyl)-3-((1-(4-hydroxyphenyl)-1H-1,2,3-triazol-4-yl)methyl)urea (**2p**)*. The crude residue was pre-absorbed on silica gel and purified by flash sil-ica gel column chromatography using a mixture of 50% ethyl acetate in hexane as an el-uent to provide (189 mg, 85% yield) the compound **2p** as a light-brown solid; mp = 139–140 °C; ^1^H NMR (300 MHz, DMSO*-d_6_*) δ 4.43 (d, 2H, *J* = 5.5 Hz), 6.88 (brt, 1H, *J* = 5.6 Hz), 6.92 (d, 2H, *J* = 8.9 Hz), 7.52 (d, 1H, *J* = 8.8 Hz), 7.58 (dd, 1H, *J* = 8.9 and 2.3 Hz), 7.65 (d, 2H, *J* = 8.9 Hz), 8.09 (d, 1H, *J* = 2.3 Hz), 8.46 (s, 1H), 9.11 (s, 1H) and 9.93 (s, 1H) ppm.; ^13^C NMR (75 MHz, DMSO*-d_6_*) δ 158.1, 155.3, 146.5, 140.5, 132.3, 129.3, 127.1 (q, *J* = 30.8 Hz), 123.3 (q, *J* = 271.5 Hz), 122.9, 122.3 (2C), 122.0 (q, *J* = 1.5 Hz), 121.3, 116.7 (q, *J* = 6.0 Hz), 116.5 (2C) and 35.3 ppm.; ^19^F NMR (282 MHz, DMSO*-d_6_*) δ −63.47 (s, 3F) ppm.; HRMS (ESI+): *m/z* = 412.0772 [M+H]^+^; calcd 412.0788 for [(C_17_H_13_ClF_3_N_5_O_2_)+H]^+^.

*1-(4-chloro-3-(trifluoromethyl)phenyl)-3-((1-(2-methoxyphenyl)-1H-1,2,3-triazol-4-yl)methyl)urea (**2q**)*. The crude residue was pre-absorbed on silica gel and purified by flash sil-ica gel column chromatography using a mixture of 40% ethyl acetate in hexane as an el-uent to provide (202 mg, 88% yield) the compound **2q** as a white solid; mp = 176–177 °C; ^1^H NMR (300 MHz, DMSO*-d_6_*) δ 3.84 (s, 3H), 4.44 (d, 2H, *J* = 5.3 Hz), 6.90 (brt, 1H, *J* = 5.4 Hz), 7.13 (td, 1H, *J* = 7.8 and 1.2 Hz), 7.31 (dd, 1H, *J* = 8.4 and 1.2 Hz), 7.48–7.62 (m, 4H), 8.08 (d, 1H, *J* = 2.3 Hz), 8.29 (s, 1H) and 9.12 (s, 1H) ppm.; ^13^C NMR (75 MHz, DMSO*-d_6_*) δ 154.8, 151.6, 145.1, 140.0, 131.9, 130.6, 126.6 (q, *J* = 30.0 Hz), 125.8, 125.7, 124.6, 122.9 (q, *J* = 270.8 Hz), 122.4, 121.6 (q, *J* = 1.5 Hz), 120.9, 116.3 (q, *J* = 6.0 Hz), 113.0, 56.1 and 34.8 ppm.; ^19^F NMR (282 MHz, DMSO*-d_6_*) δ −63.08 (s, 3F) ppm.; HRMS (ESI+): *m/z* = 426.0937 [M+H]^+^; calcd 426.0945 for [(C_18_H_15_ClF_3_N_5_O_2_)+H]^+^.

*1-(4-chloro-3-(trifluoromethyl)phenyl)-3-((1-(3-methoxyphenyl)-1H-1,2,3-triazol-4-yl)methyl)urea (**2r**)*. The crude residue was pre-absorbed on silica gel and purified by flash sil-ica gel column chromatography using a mixture of 50% ethyl acetate in hexane as an el-uent to provide (221 mg, 96% yield) the compound **2r** as a beige-colored solid; mp = 164–165 °C; ^1^H NMR (300 MHz, DMSO*-d_6_*) δ 3.84 (s, 3H), 4.44 (d, 2H, *J* = 5.4 Hz), 6.93 (brt, 1H, *J* = 5.4 Hz), 7.03 (ddd, 1H, *J* = 3.0, 2.4 and 0.6 Hz), 7.43–7.51 (m, 3H), 7.54 (d, 1H, *J* = 8.7 Hz), 7.59 (dd, 1H, *J* = 8.7 and 2.1 Hz), 8.10 (d, 1H, *J* = 2.3 Hz), 8.70 (s, 1H) and 9.15 (s, 1H) ppm.; ^13^C NMR (75 MHz, DMSO*-d_6_*) δ 160.2, 154.8, 146.5, 140.0, 137.8, 131.9, 130.8, 126.6 (q, *J* = 30.0 Hz), 122.9 (q, *J* = 270.8 Hz), 122.4, 121.6 (q, *J* = 1.5 Hz), 121.1, 116.3 (q, *J* = 5.3 Hz), 114.3, 112.0, 105.6, 55.6 and 34.9 ppm.; ^19^F NMR (282 MHz, DMSO*-d_6_*) δ −63.08 (s, 3F) ppm.; HRMS (ESI+): *m/z* = 426.0936 [M+H]^+^; calcd 426.0945 for [(C_18_H_15_ClF_3_N_5_O_2_)+H]^+^.

*1-(4-chloro-3-(trifluoromethyl)phenyl)-3-((1-(4-methoxyphenyl)-1H-1,2,3-triazol-4-yl)methyl)urea (**2s**).* The crude residue was pre-absorbed on silica gel and purified by flash sil-ica gel column chromatography using a mixture of 50% ethyl acetate in hexane as an el-uent to provide (205 mg, 89% yield) the compound **2s** as a light-orange solid; mp = 156–157 °C; ^1^H NMR (300 MHz, DMSO*-d_6_*) δ 3.82 (s, 3H), 4.43 (d, 2H, *J* = 5.3 Hz), 6.90 (brt, 1H, *J* = 5.4 Hz), 7.11 (d, 2H, *J* = 9.0 Hz), 7.53 (d, 1H, *J* = 9.0 Hz), 7.59 (dd, 1H, *J* = 9.0 and 2.4 Hz), 7.79 (d, 2H, *J* = 9.0 Hz), 8.09 (d, 1H, *J* = 2.4 Hz), 8.55 (s, 1H) and 9.13 (s, 1H) ppm.; ^13^C NMR (75 MHz, DMSO*-d_6_*) δ 159.2, 154.9, 146.3, 140.0, 131.9, 130.2, 126.7 (q, *J* = 30.0 Hz), 122.9 (q, *J* = 271.5 Hz), 122.5, 121.7 (3C), 120.9, 116.3 (q, *J* = 5.3 Hz), 114.9 (2C), 55.6 and 34.9 ppm.; ^19^F NMR (282 MHz, DMSO*-d_6_*) δ −63.08 (s, 3F) ppm.; HRMS (ESI+): *m/z* = 426.0934 [M+H]^+^; calcd 426.0945 for [(C_18_H_15_ClF_3_N_5_O_2_)+H]^+^.

*1-(4-chloro-3-(trifluoromethyl)phenyl)-3-((1-(o-tolyl)-1H-1,2,3-triazol-4-yl)methyl)urea (**2t**)*. The crude residue was pre-absorbed on silica gel and purified by flash silica gel column chromatography using a mixture of 50% ethyl acetate in hexane as an eluent to provide (182 mg, 82% yield) the compound **2t** as a light-brown solid; mp = 166–167 °C; ^1^H NMR (300 MHz, DMSO*-d_6_*) δ 2.14 (s, 3H), 4.46 (d, 2H, *J* = 5.3 Hz), 6.91 (brt, 1H, *J* = 5.5 Hz), 7.37–7.42 (m, 2H), 7.43–7.49 (m, 2H), 7.54 (d, 1H, *J* = 8.7 Hz), 7.59 (dd, 1H, *J* = 8.7 and 2.4 Hz), 8.09 (d, 1H, *J* = 2.3 Hz), 8.32 (s, 1H) and 9.13 (s, 1H) ppm.; ^13^C NMR (75 MHz, DMSO*-d_6_*) δ 154.9, 145.4, 140.0, 136.4, 133.0, 131.9, 131.4, 129.8, 127.0, 126.7 (q, *J* = 30.8 Hz), 126.0, 124.3, 122.9 (q, *J* = 271.5 Hz), 122.5, 121.6 (q, *J* = 2.3 Hz), 116.3 (q, *J* = 6.0 Hz), 34.9 and 17.5 ppm.; ^19^F NMR (282 MHz, DMSO*-d_6_*) δ −63.18 (s, 3F) ppm.; HRMS (ESI+): *m/z* = 410.0933 [M+H]^+^; calcd 410.0995 for [(C_18_H_15_ClF_3_N_5_O)+H]^+^.

*1-(4-chloro-3-(trifluoromethyl)phenyl)-3-((1-(m-tolyl)-1H-1,2,3-triazol-4-yl)methyl)urea (**2u**).* The crude residue was pre-absorbed on silica gel and purified by flash silica gel column chromatography using a mixture of 40% ethyl acetate in hexane as an eluent to provide (197 mg, 89% yield) the compound **2u** as a white solid; mp = 188–189 °C; ^1^H NMR (300 MHz, DMSO*-d_6_*) δ 2.40 (s, 3H), 4.44 (d, 2H, *J* = 5.4 Hz), 6.91 (brt, 1H, *J* = 5.4 Hz), 7.28 (brd, 1H, *J* = 7.5 Hz), 7.45 (t, 1H, *J* = 7.8 Hz), 7.54 (d, 1H, *J* = 8.7 Hz), 7.59 (dd, 1H, *J* = 9.0 and 2.4 Hz), 7.68 (brd, 1H, *J* = 8.1 Hz), 7.73 (brs, 1H), 8.09 (d, 1H, *J* = 2.2 Hz), 8.62 (s, 1H) and 9.14 (s, 1H) ppm.; ^13^C NMR (75 MHz, DMSO*-d_6_*) δ 155.9, 147.4, 141.0, 140.6, 137.6, 132.8, 130.6, 130.1, 127.6 (q, *J* = 30.8 Hz), 123.8 (q, *J* = 270.8 Hz), 123.4, 122.5 (q, *J* = 2.3 Hz), 121.8, 121.3, 118.0, 117.3 (q, *J* = 6.0 Hz), 35.8 and 21.8 ppm.; ^19^F NMR (282 MHz, DMSO*-d_6_*) δ −63.17 (s, 3F); HRMS (ESI+): *m/z* = 410.0933 [M+H]^+^; calcd 410.0995 for [(C_18_H_15_ClF_3_N_5_O)+H]^+^.

*1-(4-chloro-3-(trifluoromethyl)phenyl)-3-((1-(p-tolyl)-1H-1,2,3-triazol-4-yl)methyl)urea (**2v**)*. The crude residue was pre-absorbed on silica gel and purified by flash silica gel column chromatography using a mixture of 60% ethyl acetate in hexane as an eluent to provide (199 mg, 90% yield) the compound **2v** as a white solid; mp = 182–183 °C; ^1^H NMR (300 MHz, DMSO*-d_6_*) δ 2.38 (s, 3H), 4.44 (d, 2H, *J* = 5.5 Hz), 6.92 (brt, 1H, *J* = 5.6 Hz), 7.38 (d, 2H, *J* = 8.2 Hz), 7.55 (d, 1H, *J* = 8.8 Hz), 7.59 (dd, 1H, *J* = 8.9 and 2.3 Hz), 7.77 (d, 2H, *J* = 8.5 Hz), 8.10 (d, 1H, *J* = 2.2 Hz), 8.61 (s, 1H) and 9.15 (s, 1H) ppm.; ^13^C NMR (75 MHz, DMSO*-d_6_*) δ 154.8, 146.4, 140.0, 138.2, 134.5, 131.9, 130.2 (2C), 126.6 (q, *J* = 30.0 Hz), 122.9 (q, *J* = 271.5 Hz), 122.4, 121.6 (q, *J* = 2.3 Hz), 120.8, 119.9 (2C), 116.2 (q, *J* = 5.3 Hz), 34.9 and 20.5 ppm.; ^19^F NMR (282 MHz, DMSO*-d_6_*) δ −63.31 (s, 3F) ppm.; HRMS (ESI+): *m/z* = 410.0996 [M+H]^+^; calcd 410.0995 for [(C_18_H_15_ClF_3_N_5_O)+H]^+^.

*1-(4-chloro-3-(trifluoromethyl)phenyl)-3-((1-(2-(trifluoromethyl)phenyl)-1H-1,2,3-triazol-4-yl)methyl)urea (**2w**)*. The crude residue was pre-absorbed on silica gel and purified by flash silica gel column chromatography using a mixture of 50% ethyl acetate in hexane as an eluent to provide (248 mg, 99% yield) the compound **2w** as a white solid; mp = 113–114 °C; ^1^H NMR (300 MHz, DMSO*-d_6_*) δ 4.45 (d, 2H, *J* = 5.4 Hz), 6.91 (brt, 1H, *J* = 5.4 Hz), 7.54 (d, 1H, *J* = 9.2 Hz), 7.58 (dd, 1H, *J* = 9.2 and 2.2 Hz), 7.67 (brd, 1H, *J* = 7.7 Hz), 7.84 (brt, 1H, *J* = 7.6 Hz), 7.91 (brt, 1H, *J* = 7.4 Hz), 8.02 (brd, 1H, *J* = 7.4 Hz), 8.07 (brs, 1H), 8.35 (s, 1H) and 9.15 (s, 1H) ppm.; ^13^C NMR (75 MHz, DMSO*-d_6_*) δ 155.0, 145.6, 140.0, 134.4 (q, *J* = 1.5 Hz), 134.0, 131.9, 131.1, 129.3, 127.5 (q, *J* = 5.3 Hz), 126.8 (q, *J* = 30.8 Hz), 125.5, 124.9 (q, *J* = 3.0 Hz), 123.0 (q, *J* = 271.5 Hz), 122.9 (q, *J* = 272.3 Hz), 122.5, 121.7 (q, *J* = 1.5 Hz), 116.4 (q, *J* = 5.3 Hz) and 34.8 ppm.; ^19^F NMR (282 MHz, DMSO*-d_6_*) δ −60.05 (s, 3F) and -63.42 (s, 3F) ppm.; HRMS (ESI+): *m/z* = 464.0716 [M+H]^+^; calcd 464.0713 for [(C_18_H_12_ClF_6_N_5_O)+H]^+^.

*1-(4-chloro-3-(trifluoromethyl)phenyl)-3-((1-(3-(trifluoromethyl)phenyl)-1H-1,2,3-triazol-4-yl)methyl)urea (**2x**)*. The crude residue was pre-absorbed on silica gel and purified by flash silica gel column chromatography using a mixture of 40% ethyl acetate in hexane as an eluent to provide (245 mg, 98% yield) the compound **2x** as a white crystal solid; mp = 152–153 °C; ^1^H NMR (300 MHz, DMSO*-d_6_*) δ 4.47 (d, 2H, *J* = 5.5 Hz), 6.94 (brt, 1H, *J* = 5.6 Hz), 7.54 (d, 1H, *J* = 8,8 Hz), 7.59 (dd, 1H, *J* = 8.9 and 2.3 Hz), 7.77–7.89 (m, 2H), 8.10 (d, 1H, *J* = 2.2 Hz), 8.21–8.33 (m, 2H), 8.86 (s, 1H) and 9.17 (s, 1H) ppm.; ^13^C NMR (75 MHz, DMSO*-d_6_*) δ 154.9, 147.0, 140.0, 137.2, 131.9, 131.3, 130.6 (q, *J* = 32.3 Hz), 126.7 (q, *J* = 30.0 Hz), 125.1 (q, *J* = 3.8 Hz), 123.9, 123.6 (q, *J* = 270.8 Hz), 122.9 (q, *J* = 271.5 Hz), 122.5, 121.6 (q, *J* = 1.5 Hz), 121.3, 116.6 (q, *J* = 3.8 Hz), 116.3 (q, *J* = 5.3 Hz) and 34.9 ppm.; ^19^F NMR (282 MHz, DMSO*-d_6_*) δ −63.01 (s, 3F) and -63.23 (s, 3F) ppm.; HRMS (ESI+): *m/z* = 464.0704 [M+H]^+^; calcd 464.0713 for [(C_18_H_12_ClF_6_N_5_O)+H]^+^.

*1-(4-chloro-3-(trifluoromethyl)phenyl)-3-((1-(4-(trifluoromethyl)phenyl)-1H-1,2,3-triazol-4-yl)methyl)urea (**2y**)*. The crude residue was pre-absorbed on silica gel and purified by flash silica gel column chromatography using a mixture of 40% ethyl acetate in hexane as an eluent to provide (245 mg, 98% yield) the compound **2y** as a white solid; mp = 199–200 °C; ^1^H NMR (300 MHz, DMSO*-d_6_*) δ 4.46 (d, 2H, *J* = 5.5 Hz), 6.94 (brt, 1H, *J* = 5.6 Hz), 7.54 (d, 1H, *J* = 8.8 Hz), 7.59 (dd, 1H, *J* = 8.9 and 2.3 Hz), 7.97 (d, 2H, *J* = 8.6 Hz), 8.10 (d, 1H, *J* = 2.2 Hz), 8.20 (d, 2H, *J* = 8.5 Hz), 8.82 (s, 1H) and 9.17 (s, 1H) ppm.; ^13^C NMR (75 MHz, DMSO*-d_6_*) δ 154.9, 147.1, 140.0, 139.5, 131.8, 128.6 (q, *J* = 32.3 Hz), 127.2 (2C, q, *J* = 3.8 Hz), 126.7 (q, *J* = 30.0 Hz), 123.9 (q, *J* = 270.8 Hz), 122.9 (q, *J* = 271.5 Hz), 122.5, 121.6 (q, *J* = 1.5 Hz), 121.2, 120.4 (2C), 116.3 (q, *J* = 5.3 Hz) and 34.9 ppm.; ^19^F NMR (282 MHz, DMSO*-d_6_*) δ −62.74 (s, 3F) and −63.19 (s, 3F) ppm.; HRMS (ESI+): *m/z* = 464.0717 [M+H]^+^; calcd 464.0713 for [(C_18_H_12_ClF_6_N_5_O)+H]^+^.

*1-(4-chloro-3-(trifluoromethyl)phenyl)-3-((1-(2-cyanophenyl)-1H-1,2,3-triazol-4-yl)methyl)urea (**2z**)*. The crude residue was pre-absorbed on silica gel and purified by flash silica gel column chromatography using a mixture of 60% ethyl acetate in hexane as an eluent to provide (216 mg, 95% yield) the compound **2z** as a light-yellow solid; mp = 162–163 °C; ^1^H NMR (300 MHz, DMSO*-d_6_*) δ 4.49 (d, 2H, *J* = 5.5 Hz), 6.97 (brt, 1H, *J* = 5.6 Hz), 7.54 (d, 1H, *J* = 8.8 Hz), 7.59 (dd, 1H, *J* = 8.9 and 2.3 Hz), 7.75 (td, 1H, *J* = 7.6 and 1.2 Hz), 7.85 (dd, 1H, *J* = 8.0 and 1.0 Hz), 7.94 (td, 1H, *J* = 8.1 and 1.4 Hz), 8.10 (d, 1H, *J* = 2.3 Hz), 8.12 (dd, 1H, *J* = 7.5 and 1.3 Hz), 8.61 (s, 1H) and 9.17 (s, 1H) ppm.; ^13^C NMR (75 MHz, DMSO*-d_6_*) δ 154.9, 146.5, 140.0, 137.9, 134.8, 124.7, 131.8, 130.1, 126.6 (q, *J* = 30.0 Hz), 125.7, 123.7, 122.9 (q, *J* = 271.5 Hz), 122.5, 121.6 (q, *J* = 1.5 Hz), 116.3 (q, *J* = 6.0 Hz), 115.9, 107.0 and 34.8 ppm.; ^19^F NMR (282 MHz, DMSO*-d_6_*) δ −62.08 (s, 3F) ppm.; HRMS (ESI+): *m/z* = 421.0735 [M+H]^+^; calcd 421.0791 for [(C_18_H_13_ClF_3_N_6_O)+H]^+^.

*1-(4-chloro-3-(trifluoromethyl)phenyl)-3-((1-(3-cyanophenyl)-1H-1,2,3-triazol-4-yl)methyl)urea (**2a******’****)*. The crude residue was pre-absorbed on silica gel and purified by flash silica gel column chromatography using a mixture of 60% ethyl acetate in hexane as an eluent to provide (220 mg, 97% yield) the compound **2a’** as a beige-colored crystal solid; mp = 212–213 °C; ^1^H NMR (300 MHz, DMSO*-d_6_*) δ 4.46 (d, 2H, *J* = 5.5 Hz), 6.95 (brt, 1H, *J* = 5.7 Hz), 7.54 (d, 1H, *J* = 8.8 Hz), 7.58 (dd, 1H, *J* = 8.8 and 2.3 Hz), 7.79 (t, 1H, *J* = 8.0 Hz), 7.94 (tt, 1H, *J* = 7.7 and 1.1 Hz), 8.09 (d, 1H, *J* = 2.3 Hz), 8.28 (ddd, 1H, *J* = 8.3, 2.2 and 1.0 Hz), 8.45 (brt, 1H, *J* = 1.7 Hz), 8.79 (s, 1H) and 9.18 (s, 1H) ppm.; ^13^C NMR (75 MHz, DMSO*-d_6_*) δ 155.8, 148.1, 141.0, 138.0, 133.0, 132.8, 132.2, 127.6 (q, *J* = 30.0 Hz), 125.5, 124.2, 123.8 (q, *J* = 271.5 Hz), 123.4, 122.5 (q, *J* = 1.5 Hz), 122.1, 118.8, 117.2 (q, *J* = 5.3 Hz), 113.8 and 35.8 ppm.; ^19^F NMR (282 MHz, DMSO*-d_6_*) δ −62.14 (s, 3F) ppm.; HRMS (ESI+): *m/z* = 421.0725 [M+H]^+^; calcd 421.0791 for [(C_18_H_13_ClF_3_N_6_O)+H]^+^.

*1-(4-chloro-3-(trifluoromethyl)phenyl)-3-((1-(4-cyanophenyl)-1H-1,2,3-triazol-4-yl)methyl)urea (**2b’**)*. The crude residue was pre-absorbed on silica gel and purified by flash silica gel column chromatography using a mixture of 50% ethyl acetate in hexane as an eluent to provide (220 mg, 97% yield) the compound **2b’** as a light-yellow crystal solid; mp = 223–224 °C; ^1^H NMR (300 MHz, DMSO*-d_6_*) δ 4.47 (d, 2H, *J* = 5.6 Hz), 6.93 (brt, 1H, *J* = 5.6 Hz), 7.50 (d, 1H, *J* = 8.8 Hz), 7.58 (dd, 1H, *J* = 8.8 and 2.3 Hz), 8.05 (d, 2H, *J* = 8.8 Hz), 8.08 (d, 1H, *J* = 2.4 Hz), 8.14 (d, 2H, *J* = 8.8 Hz), 8.82 (s, 1H) and 9.15 (s, 1H) ppm.; ^13^C NMR (75 MHz, DMSO*-d_6_*) δ 154.9, 147.2, 140.0, 139.6, 134.2 (2C), 131.8, 126.6 (q, *J* = 30.0 Hz), 122.9 (q, *J* = 271.5 Hz), 122.4, 121.6 (q, *J* = 2.3 Hz), 121.1, 120.3 (2C), 118.1, 116.3 (q, *J* = 6.8 Hz), 110.9 and 34.8 ppm.; ^19^F NMR (282 MHz, DMSO*-d_6_*) δ −63.19 (s, 3F) ppm.; HRMS (ESI+): *m/z* = 421.0783 [M+H]^+^; calcd 421.0791 for [(C_18_H_13_ClF_3_N_6_O)+H]^+^.

*1-(4-chloro-3-(trifluoromethyl)phenyl)-3-((1-(2-nitrophenyl)-1H-1,2,3-triazol-4-yl)methyl)urea (**2c’**)*. The crude residue was pre-absorbed on silica gel and purified by flash silica gel column chromatography using a mixture of 60% ethyl acetate in hexane as an eluent to provide (212 mg, 89% yield) the compound **2c’** as a brown solid; mp = 159–160 °C; ^1^H NMR (300 MHz, DMSO*-d_6_*) δ 4.47 (d, 2H, *J* = 5.5 Hz), 6.94 (brt, 1H, *J* = 5.5 Hz), 7.55 (d, 1H, *J* = 9.0 Hz), 7.59 (dd, 1H, *J* = 9.0 and 2.4 Hz), 7.78–7.87 (m, 2H), 7.94 (ddd, 1H, *J* = 9.6, 8.1 and 1.2 Hz), 8.10 (d, 1H, *J* = 2.4 Hz), 8.20 (dd, 1H, *J* = 8.1 and 1.5 Hz), 8.55 (s, 1H) and 9.13 (s, 1H) ppm.; ^13^C NMR (75 MHz, DMSO*-d_6_*) δ 154.8, 146.3, 144.1, 140.0, 134.3, 131.9, 131.0, 129.2, 127.4, 126.6 (q, *J* = 30.0 Hz), 125.5, 124.0, 122.9 (q, *J* = 271.5 Hz), 122.5, 121.6 (q, *J* = 1.5 Hz), 116.3 (q, *J* = 6.0 Hz) and 34.7 ppm.; ^19^F NMR (282 MHz, DMSO*-d_6_*) δ −63.11 (s, 3F) ppm.; HRMS (ESI+): *m/z* = 441.0632 [M+H]^+^; calcd 441.0690 for [(C_17_H_13_ClF_3_N_6_O_3_)+H]^+^.

*1-(4-chloro-3-(trifluoromethyl)phenyl)-3-((1-(3-nitrophenyl)-1H-1,2,3-triazol-4-yl)methyl)urea (**2d’**)*. The crude residue was pre-absorbed on silica gel and purified by flash silica gel column chromatography using a mixture of 60% ethyl acetate in hexane as an eluent to provide (226 mg, 95% yield) the compound **2d’** as a yellow crystal solid; mp = 189–190 °C; ^1^H NMR (300 MHz, DMSO*-d_6_*) δ 4.47 (d, 2H, *J* = 5.5 Hz), 6.95 (brt, 1H, *J* = 5.7 Hz), 7.54 (d, 1H, *J* = 8.7 Hz), 7.59 (dd, 1H, *J* = 9.0 and 2.4 Hz), 7.88 (dd, 1H, *J* = 8.4 and 8.1 Hz), 8.10 (d, 1H, *J* = 2.4 Hz), 8.31 (ddd, 1H, *J* = 8.1, 2.1 and 0.9 Hz), 8.41 (ddd, 1H, *J* = 8.4, 2.4 and 0.9 Hz), 8.73 (t, 1H, *J* = 2.1 Hz), 8.92 (s, 1H) and 9.18 (s, 1H) ppm.; ^13^C NMR (75 MHz, DMSO*-d_6_*) δ 154.8, 148.5, 147.1, 140.0, 137.2, 131.8, 131.5, 126.6 (q, *J* = 30.0 Hz), 125.9, 122.9, 122.8 (q, *J* = 271.5 Hz), 122.4, 121.5 (q, *J* = 1.5 Hz), 121.4, 116.2 (q, *J* = 5.3 Hz), 114.6 and 34.8 ppm.; ^19^F NMR (282 MHz, DMSO*-d_6_*) δ −63.06 (s, 3F) ppm.; HRMS (ESI+): *m/z* = 441.0630 [M+H]^+^; calcd 441.0690 for [(C_17_H_13_ClF_3_N_6_O_3_)+H]^+^.

*1-(4-chloro-3-(trifluoromethyl)phenyl)-3-((1-(4-nitrophenyl)-1H-1,2,3-triazol-4-yl)methyl)urea (**2e’**)*. The crude residue was pre-absorbed on silica gel and purified by flash silica gel column chromatography using a mixture of 60% ethyl acetate in hexane as an eluent to provide (219 mg, 92% yield) the compound **2e’** as a light-yellow solid; mp = 176–177 °C; ^1^H NMR (300 MHz, DMSO*-d_6_*) δ 4.47 (d, 2H, *J* = 5.6 Hz), 6.96 (brt, 1H, *J* = 5.6 Hz), 7.54 (d, 1H, *J* = 8.9 Hz), 7.59 (dd, 1H, *J* = 8.9 and 2.3 Hz), 8.10 (d, 1H, *J* = 2.3 Hz), 8.23 (d, 2H, *J* = 9.2 Hz), 8.43 (d, 2H, *J* = 9.2 Hz), 8.89 (s, 1H) and 9.19 (s, 1H) ppm.; ^13^C NMR (75 MHz, DMSO*-d_6_*) δ 154.9, 147.4, 146.6, 140.9, 140.0, 131.9, 126.6 (q, *J* = 30.8 Hz), 125.5 (2C), 122.9 (q, *J* = 271.5 Hz), 122.5, 121.6 (q, *J* = 1.5 Hz), 121.4, 120.5 (2C), 116.3 (q, *J* = 6.0 Hz) and 34.8 ppm.; ^19^F NMR (282 MHz, DMSO*-d_6_*) δ −63.14 (s, 3F) ppm.; HRMS (ESI+): *m/z* = 441.0691 [M+H]^+^; calcd 441.0690 for [(C_17_H_13_ClF_3_N_6_O_3_)+H]^+^.

*1-(4-chloro-3-(trifluoromethyl)phenyl)-3-((1-(2-ethylphenyl)-1H-1,2,3-triazol-4-yl)methyl)urea (**2f’**)*. The crude residue was pre-absorbed on silica gel and purified by flash silica gel column chromatography using a mixture of 40% ethyl acetate in hexane as an eluent to provide (215 mg, 94% yield) the compound **2f’** as a white solid; mp = 153–154 °C; ^1^H NMR (300 MHz, DMSO*-d_6_*) δ 1.01 (t, 3H, *J* = 7.5 Hz), 2.42 (q, 2H, *J* = 7.5 Hz), 4.46 (d, 2H, *J* = 5.2 Hz), 6.90 (brt, 1H, *J* = 5.3 Hz), 7.35 (dd, 1H, *J* = 7.8 and 2.1 Hz), 7.40 (td, 1H, *J* = 7.8 and 2.1 Hz), 7.46–7.53 (m, 2H), 7.54 (d, 1H, *J* = 8.4 Hz), 7.59 (dd, 1H, *J* = 9.0 and 2.4 Hz), 8.09 (d, 1H, *J* = 2.2 Hz), 8.29 (s, 1H) and 9.13 (s, 1H) ppm.; ^13^C NMR (75 MHz, DMSO*-d_6_*) δ 154.8, 145.3, 140.0, 139.3, 135.8, 131.8, 130.1, 129.8, 126.9, 126.5 (q, *J* = 30.0 Hz), 126.3, 124.6, 122.9 (q, *J* = 270.8 Hz), 122.4, 121.5 (q, *J* = 1.5 Hz), 116.3 (q, *J* = 6.0 Hz), 34.9, 23.7 and 14.8 ppm.; ^19^F NMR (282 MHz, DMSO*-d_6_*) δ −63.07 (s, 3F) ppm.; HRMS (ESI+): *m/z* = 424.1126 [M+H]^+^; calcd 424.1152 for [(C_19_H_17_ClF_3_N_5_O)+H]^+^.

*1-(4-chloro-3-(trifluoromethyl)phenyl)-3-((1-(3-ethylphenyl)-1H-1,2,3-triazol-4-yl)methyl)urea (**2g’**)*. The crude residue was pre-absorbed on silica gel and purified by flash silica gel column chromatography using a mixture of 40% ethyl acetate in hexane as an eluent to provide (224 mg, 98% yield) the compound **2g’** as a beige-colored solid; mp = 148–149 °C; ^1^H NMR (300 MHz, DMSO*-d_6_*) δ 1.22 (t, 3H, *J* = 7.6 Hz), 2.70 (q, 2H, *J* = 7.6 Hz), 4.44 (d, 2H, *J* = 5.3 Hz), 6.92 (brt, 1H, *J* = 5.5 Hz), 7.32 (brd, 1H, *J* = 7.5 Hz), 7.48 (t, 1H, *J* = 7.8 Hz), 7.54 (d, 1H, *J* = 8.7 Hz), 7.59 (dd, 1H, *J* = 8.7 and 2.4 Hz), 7.70 (brd, 1H, *J* = 7.70 Hz), 7.75 (brs, 1H), 8.10 (d, 1H, *J* = 2.3 Hz), 8.65 (s, 1H) and 9.14 (s, 1H) ppm.; ^13^C NMR (75 MHz, DMSO*-d_6_*) δ 154.8, 146.5, 145.9, 140.0, 136.7, 131.8, 129.7, 128.0, 126.6 (q, *J* = 30.8 Hz), 122.9 (q, *J* = 271.5 Hz), 122.4, 121.6 (q, *J* = 1.5 Hz), 120.9, 119.3, 117.3, 116.3 (q, *J* = 6.0 Hz), 34.9, 28.0 and 15.4 ppm.; ^19^F NMR (282 MHz, DMSO*-d_6_*) δ −63.06 (s, 3F) ppm.; HRMS (ESI+): *m/z* = 424.1122 [M+H]^+^; calcd 424.1152 for [(C_17_H_13_ClF_3_N_6_O_3_)+H]^+^.

*1-(4-chloro-3-(trifluoromethyl)phenyl)-3-((1-(4-ethylphenyl)-1H-1,2,3-triazol-4-yl)methyl)urea (**2h’**).* The crude residue was pre-absorbed on silica gel and purified by flash silica gel column chromatography using a mixture of 40% ethyl acetate in hexane as an eluent to provide (229 mg, 100% yield) the compound **2h’** as a white solid; mp = 169–170 °C; ^1^H NMR (300 MHz, DMSO*-d_6_*) δ 1.21 (t, 3H, *J* = 7.6 Hz), 2.67 (q, 2H, *J* = 7.6 Hz), 4.44 (d, 2H, *J* = 5.3 Hz), 6.91 (brt, 1H, *J* = 5.4 Hz), 7.40 (d, 2H, *J* = 8.4 Hz), 7.54 (d, 1H, *J* = 8.7 Hz), 7.59 (dd, 1H, *J* = 8.7 and 2.4 Hz), 7.79 (d, 2H, *J* = 8.4 Hz), 8.09 (d, 1H, *J* = 2.3 Hz), 8.61 (s, 1H) and 9.13 (s, 1H) ppm.; ^13^C NMR (75 MHz, DMSO*-d_6_*) δ 154.8, 146.4, 144.4, 140.0, 134.6, 131.8, 129.0 (2C), 126.6 (q, *J* = 30.8 Hz), 122.9 (q, *J* = 271.5 Hz), 122.4, 121.6 (q, *J* = 2.3 Hz), 120.8, 120.0 (2C), 116.3 (q, *J* = 6.0 Hz), 34.9, 27.7 and 15.4 ppm.; ^19^F NMR (282 MHz, DMSO*-d_6_*) δ −63.06 (s, 3F) ppm.; HRMS (ESI+): *m/z* = 424.1123 [M+H]^+^; calcd 424.1152 for [(C_17_H_13_ClF_3_N_6_O_3_)+H]^+^.

*1-(4-chloro-3-(trifluoromethyl)phenyl)-3-((1-(2-isopropylphenyl)-1H-1,2,3-triazol-4-yl)methyl)urea (**2i’**)*. The crude residue was pre-absorbed on silica gel and purified by flash silica gel column chromatography using a mixture of 40% ethyl acetate in hexane as an eluent to provide (187 mg, 79% yield) the compound **2i’** as a white solid; mp = 172–173 °C; ^1^H NMR (300 MHz, DMSO*-d_6_*) δ 1.11 (d, 6H, *J* = 6.9 Hz), 2.56 (septet, 1H, *J* = 6.9 Hz), 4.46 (d, 2H, *J* = 5.3 Hz), 6.93 (brt, 1H, *J* = 5.4 Hz), 7.31 (dd, 1H, *J* = 7.2 and 0.9 Hz), 7.39 (ddd, 1H, *J* = 8.7, 7.8 and 3.0 Hz), 7.50–7.62 (m, 4H), 8.09 (d, 1H, *J* = 2.1 Hz), 8.28 (s, 1H) and 9.13 (s, 1H) ppm.; ^13^C NMR (75 MHz, DMSO*-d_6_*) δ 154.8, 145.3, 144.2, 140.0, 135.0, 131.8, 130.4, 126.8, 126.7, 126.6 (q, *J* = 3.0 Hz), 126.5, 124.9, 122.9 (q, *J* = 271.5 Hz), 122.4, 121.6 (q, *J* = 1.5 Hz), 116.2 (q, *J* = 6.0 Hz), 34.9, 27.5 and 23.5 (2C) ppm.; ^19^F NMR (282 MHz, DMSO*-d_6_*) δ −63.07 (s, 3F) ppm.; HRMS (ESI+): *m/z* = 438.1285 [M+H]^+^; calcd 438.1308 for [(C_20_H_19_ClF_3_N_5_O)+H]^+^.

*1-(4-chloro-3-(trifluoromethyl)phenyl)-3-((1-(3-isopropylphenyl)-1H-1,2,3-triazol-4-yl)methyl)urea (**2j’**)*. The crude residue was pre-absorbed on silica gel and purified by flash silica gel column chromatography using a mixture of 40% ethyl acetate in hexane as an eluent to provide (208 mg, 88% yield) the compound **2j’** as a light-yellow solid; mp = 162–163 °C; ^1^H NMR (300 MHz, DMSO*-d_6_*) δ 1.24 (d, 6H, *J* = 6.9 Hz), 2.99 (septet, 1H, *J* = 6.9 Hz), 4.45 (d, 2H, *J* = 5.4 Hz), 6.92 (brt, 1H, *J* = 5.5 Hz), 7.35 (brd, 1H, *J* = 7.8 Hz), 7.48 (t, 1H, *J* = 7.8 Hz), 7.54 (d, 1H, *J* = 8.7 Hz), 7.59 (dd, 1H, *J* = 8.7 and 2.1 Hz), 7.69 (ddd, 1H, *J* = 7.8, 2.1 and 1.2 Hz), 7.75 (brt, 1H, *J* = 1.8 Hz), 8.09 (d, 1H, *J* = 2.3 Hz), 8.67 (s, 1H) and 9.14 (s, 1H) ppm.; ^13^C NMR (75 MHz, DMSO*-d_6_*) δ 154.8, 150.6, 146.4, 140.0, 136.8, 131.8, 129.8, 126.6 (q, *J* = 30.0 Hz), 126.5, 122.9 (q, *J* = 270.8 Hz), 122.4, 121.6 (q, *J* = 1.5 Hz), 121.0, 118.0, 117.5, 116.3 (q, *J* = 6.0 Hz), 34.9, 34.4 and 23.6 (2C) ppm.; ^19^F NMR (282 MHz, DMSO*-d_6_*) δ −63.07 (s, 3F) ppm.; HRMS (ESI+): *m/z* = 438.1289 [M+H]^+^; calcd 438.1308 for [(C_20_H_19_ClF_3_N_5_O)+H]^+^.

*1-(4-chloro-3-(trifluoromethyl)phenyl)-3-((1-(4-isopropylphenyl)-1H-1,2,3-triazol-4-yl)methyl)urea (**2k’**).* The crude residue was pre-absorbed on silica gel and purified by flash silica gel column chromatography using a mixture of 40% ethyl acetate in hexane as an eluent to provide (220 mg, 93% yield) the compound **2k’** as a beige-colored solid; mp = 156–157 °C; ^1^H NMR (300 MHz, DMSO*-d_6_*) δ 1.23 (d, 6H, *J* = 6.9 Hz), 2.97 (septet, 1H, *J* = 6.9 Hz), 4.44 (d, 2H, *J* = 5.3 Hz), 6.91 (brt, 1H, *J* = 5.4 Hz), 7.44 (d, 2H, *J* = 8.7 Hz), 7.54 (d, 1H, *J* = 8.7 Hz), 7.59 (dd, 1H, *J* = 8.7 and 2.4 Hz), 7.79 (d, 2H, *J* = 8.4 Hz), 8.09 (d, 1H, *J* = 2.3 Hz), 8.61 (s, 1H) and 9.13 (s, 1H) ppm.; ^13^C NMR (75 MHz, DMSO*-d_6_*) δ 154.8, 150.0, 146.4, 140.1, 134.7, 131.9, 127.6 (2C), 126.7 (q, *J* = 30.8 Hz), 122.9 (q, *J* = 271.5 Hz), 122.4, 121.6 (q, *J* = 1.5 Hz), 120.9, 120.1 (2C), 116.3 (q, *J* = 5.3 Hz), 34.9, 33.1 and 23.7 (2C) ppm.; ^19^F NMR (282 MHz, DMSO*-d_6_*) δ −63.06 (s, 3F) ppm.; HRMS (ESI+): *m/z* = 438.1303 [M+H]^+^; calcd 438.1308 for [(C_20_H_19_ClF_3_N_5_O)+H]^+^.

*1-((1-(3-(tert-butyl)phenyl)-1H-1,2,3-triazol-4-yl)methyl)-3-(4-chloro-3-(trifluoromethyl)phenyl)urea (**2l’**).* The crude residue was pre-absorbed on silica gel and purified by flash silica gel column chromatography using a mixture of 30% ethyl acetate in hexane as an eluent to provide (207 mg, 85% yield) the compound **2l’** as a white solid; mp = 187–188 °C; ^1^H NMR (300 MHz, DMSO*-d_6_*) δ 1.33 (s, 9H), 4.45 (d, 2H, *J* = 5.4 Hz), 6.91 (brt, 1H, *J* = 5.5 Hz), 7.48–7.51 (m, 2H), 7.54 (d, 1H, *J* = 8.7 Hz), 7.59 (dd, 1H, *J* = 8.7 and 2.4 Hz), 7.65–7.73 (m, 1H), 7.84 (brs, 1H), 8.10 (d, 1H, *J* = 2.1 Hz), 8.71 (s, 1H) and 9.14 (s, 1H) ppm.; ^13^C NMR (75 MHz, DMSO*-d_6_*) δ 154.8, 152.8, 146.4, 140.0, 136.6, 131.8, 129.5, 126.6 (q, *J* = 30.0 Hz), 125.4, 122.9 (q, *J* = 270.8 Hz), 122.4, 121.6 (q, *J* = 1.5 Hz), 121.1, 117.4, 117.0, 116.3 (q, *J* = 5.3 Hz), 34.9, 34.7 and 30.6 (3C) ppm.; ^19^F NMR (282 MHz, DMSO*-d_6_*) δ −63.06 (s, 3F) ppm.; HRMS (ESI+): *m/z* = 452.1412 [M+H]^+^; calcd 452.1465 for [(C_21_H_22_ClF_3_N_5_O)+H]^+^.

*1-((1-(4-(tert-butyl)phenyl)-1H-1,2,3-triazol-4-yl)methyl)-3-(4-chloro-3-(trifluoromethyl)phenyl)urea (**2m’**)*. The crude residue was pre-absorbed on silica gel and purified by flash silica gel column chromatography using a mixture of 40% ethyl acetate in hexane as an eluent to provide (212 mg, 87% yield) the compound **2m’** as a white crystal solid; mp = 94–95 °C; ^1^H NMR (300 MHz, DMSO*-d_6_*) δ 1.31 (s, 9H), 4.44 (d, 2H, *J* = 5.3 Hz), 6.91 (brt, 1H, *J* = 5.5 Hz), 7.50–7.63 (overlapped, 4H), 7.79 (d, 2H, *J* = 8.7 Hz), 8.09 (d, 1H, *J* = 2.1 Hz), 8.61 (s, 1H) and 9.14 (s, 1H) ppm.; ^13^C NMR (75 MHz, DMSO*-d_6_*) δ 154.9, 151.3, 146.4, 140.0, 134.4, 131.9, 126.7 (q, *J* = 30.0 Hz), 126.6 (2C), 122.9 (q, *J* = 271.5 Hz), 122.5, 121.6 (q, *J* = 2.3 Hz), 120.9, 119.8 (2C), 116.3 (q, *J* = 6.0 Hz), 34.9, 34.5 and 31.0 (3C) ppm.; ^19^F NMR (282 MHz, DMSO*-d_6_*) δ −63.15 (s, 3F) ppm.; HRMS (ESI+): *m/z* = 452.1469 [M+H]^+^; calcd 452.1465 for [(C_20_H_19_ClF_3_N_5_O)+H]^+^.

*3-(4-((3-(4-chloro-3-(trifluoromethyl)phenyl)ureido)methyl)-1H-1,2,3-triazol-1-yl)benzoic acid (**2n’**).* The crude residue was pre-absorbed on silica gel and purified by flash silica gel column chromatography using a mixture of 1% formic acid/50% ethyl acetate in hexane as an eluent to provide (214 mg, 90% yield) the compound **2n’** as a light-brown solid; mp = 246–247 °C; ^1^H NMR (300 MHz, DMSO*-d_6_*) δ 4.46 (d, 2H, *J* = 5.4 Hz), 6.95 (brt, 1H, *J* = 5.5 Hz), 7.54 (d, 1H, *J* = 8.7 Hz), 7.59 (dd, 1H, *J* = 8.7 and 2.1 Hz), 7.72 (dd, 1H, *J* = 8.1, 7.8 Hz), 8.02 (brd, 1H, *J* = 8.1 Hz), 8.09 (d, 1H, *J* = 2.1 Hz), 8.16 (brdd, 1H, *J* = 7.8 and 1.5 Hz), 8.40 (brt, 1H, *J* = 1.5 Hz), 8.80 (s, 1H) and 9.18 (s, 1H) ppm.; ^13^C NMR (75 MHz, DMSO*-d_6_*) δ 166.4, 154.9, 146.9, 140.1, 136.9, 132.6, 131.9, 130.4, 129.1, 126.6 (q, *J* = 30.8 Hz), 124.1, 122.9 (q, *J* = 270.8 Hz), 122.4, 121.5 (q, *J* = 2.3 Hz), 121.2, 120.4, 116.3 (q, *J* = 5.3 Hz) and 34.9 ppm.; ^19^F NMR (282 MHz, DMSO*-d_6_*) δ −63.42 (s, 3F) ppm.; HRMS (ESI+): *m/z* = 440.0688 [M+H]^+^; calcd 440.0737 for [(C_18_H_14_ClF_3_N_5_O_3_)+H]^+^.

*4-(4-((3-(4-chloro-3-(trifluoromethyl)phenyl)ureido)methyl)-1H-1,2,3-triazol-1-yl)benzoic acid (**2o’**)*. The crude residue was pre-absorbed on silica gel and purified by flash silica gel column chromatography using a mixture of 1% formic acid/60% ethyl acetate in hexane as an eluent to provide (223 mg, 94% yield) the compound **2o’** as a white solid; mp = 253–254 °C; ^1^H NMR (300 MHz, DMSO*-d_6_*) δ 4.45 (d, 2H, *J* = 5.5 Hz), 6.94 (brt, 1H, *J* = 5.6 Hz), 7.54 (d, 1H, *J* = 8.8 Hz), 7.59 (dd, 1H, *J* = 8.9 and 2.3 Hz), 8.05 (d, 2H, *J* = 8.9 Hz), 8.09 (d, 1H, *J* = 2.5 Hz), 8.12 (d, 2H, *J* = 8.9 Hz), 8.78 (s, 1H) and 9.17 (s, 1H) ppm.; ^13^C NMR (75 MHz, DMSO*-d_6_*) δ 166.5, 154.9, 147.0, 140.0, 139.6, 131.9, 131.1 (2C), 130.6, 126.7 (q, *J* = 30.0 Hz), 122.7 (q, *J* = 271.5 Hz), 122.5, 121.7 (q, *J* = 2.3 Hz), 121.1, 119.7 (2C), 116.3 and 34.9 ppm.; ^19^F NMR (282 MHz, DMSO*-d_6_*) δ −63.17 (s, 3F) ppm.; HRMS (ESI+): *m/z* = 440.0728 [M+H]^+^; calcd 440.0737 for [(C_18_H_14_ClF_3_N_5_O_3_)+H]^+^.

*N-(4-(4-((3-(4-chloro-3-(trifluoromethyl)phenyl)ureido)methyl)-1H-1,2,3-triazol-1-yl)phenyl)acetamide (**2u’**).* The crude residue was recrystallized in acetone as an solvent to provide (242 mg, 99% yield) of the compound **2u’** as a light-brown solid; mp = 238–239 °C; ^1^H NMR (300 MHz, DMSO*-d_6_*) δ 2.07 (s, 3H), 4.44 (d, 2H, *J* = 5.5 Hz), 6.92 (brt, 1H, *J* = 5.6 Hz), 7.54 (d, 1H, *J* = 8.8 Hz), 7.59 (dd, 1H, *J* = 8.7 and 2.2 Hz), 7.75 (d, 2H, *J* = 9.2 Hz), 7.81 (d, 2H, *J* = 9.2 Hz), 8.09 (d, 1H, *J* = 2.2 Hz), 8.55 (s, 1H), 9.14 (s, 1H) and 10.18 (s, 1H) ppm.; ^13^C NMR (75 MHz, DMSO*-d_6_*) δ 168.7, 154.9, 146.4, 140.0, 139.6, 131.9, 131.7, 126.7 (q, *J* = 30.8 Hz), 122.9 (q, *J* = 271.5 Hz), 121.5, 121.6 (q, *J* = 1.5 Hz), 120.8, 120.6 (2C), 119.7 (2C), 116.3 (q, *J* = 5.3 Hz), 34.9 and 24.0 ppm.; ^19^F NMR (282 MHz, DMSO*-d_6_*) δ −63.82 (s, 3F) ppm.; HRMS (ESI+): *m/z* = 453.1048 [M+H]^+^; calcd 453.1054 for [(C_19_H_17_ClF_3_N_6_O_2_)+H]^+^.

#### 3.1.5. General Procedure for the Preparation of Sorafenib Derivatives **2p’**–**2r’**

Triazole-cored derivatives **2p’**–**2r’** were prepared according to the procedure described previously [90]. A stirred solution of tin (II) chloride dihydrate (208 mg, 0.92 mmol, 4.00 eq) in conc. HCl (1.0 mL) was stirred at 0 °C for 5 min and then the nitrobenzene **2c’**, **2d’**, or **2e’** (100 mg, 0.23 mmol, 1.00 eq) was added. The reaction mixture was stirred at 65 °C for 3 h. The resulting solution was then cooled to room temperature, diluted with water (30 mL), basified to pH 8 by using an aqueous sodium hydrogen carbonate solution, and extracted with ethyl acetate (3 × 50 mL). The combined organic phase was dried over anhydrous sodium sulfate and filtered. The solvent was removed under reduced pressure and the crude product was purified by column chromatography (silica gel, ethyl acetate in hexane) to obtain compound **2p’**, **2q’**, or **2r’**.

*1-((1-(2-aminophenyl)-1H-1,2,3-triazol-4-yl)methyl)-3-(4-chloro-3-(trifluoromethyl)phenyl)urea (**2p’**).* The crude residue was pre-absorbed on silica gel and purified by flash sil-ica gel column chromatography using a mixture of 50% ethyl acetate in hexane as an el-uent to provide (88 mg, 93% yield) the compound **2p’** as a light-brown-red crystal solid; mp = 107–108 °C; ^1^H NMR (300 MHz, DMSO*-d_6_*) δ 4.46 (d, 2H, *J* = 5.3 Hz), 6.70 (td, 1H, *J* = 7.5 and 1.2 Hz), 6.89 (brt, 1H, *J* = 5.4 Hz), 6.94 (dd, 1H, *J* = 8.1 and 1.2 Hz), 7.21 (td, 1H, *J* = 7.5 and 1.5 Hz), 7.22 (dd, 1H, *J* = 8.1 and 1.2 Hz), 7.54 (d, 1H, *J* = 8.7 Hz), 7.59, (dd, 1H, *J* = 9.0 and 2.4 Hz), 8.09 (d, 1H, *J* = 2.3 Hz), 8.26 (s, 1H) and 9.15 (s, 1H) ppm.; ^13^C NMR (75 MHz, DMSO*-d_6_*) δ 154.8, 145.2, 142.0, 140.0, 131.9, 129.9, 126.6 (q, *J* = 30.0 Hz), 125.2, 123.6, 122.9 (q, *J* = 271.5 Hz), 122.4, 122.2, 121.5, 116.9, 116.4, 116.2 (q, *J* = 5.3 Hz) and 27.9 ppm.; ^19^F NMR (282 MHz, DMSO*-d_6_*) δ −63.07 (s, 3F) ppm.; HRMS (ESI+): *m/z* = 411.0884 [M+H]^+^; calcd 411.0948 for [(C_17_H_14_ClF_3_N_6_O)+H]^+^.

*1-((1-(3-aminophenyl)-1H-1,2,3-triazol-4-yl)methyl)-3-(4-chloro-3-(trifluoromethyl)phenyl)urea (**2q’**).* The crude residue was pre-absorbed on silica gel and purified by flash sil-ica gel column chromatography using a mixture of 50% ethyl acetate in hexane as an el-uent to provide (91 mg, 96% yield) the compound **2q’** as a brown-red solid; mp = 108–109 °C; ^1^H NMR (300 MHz, DMSO*-d_6_*) δ 4.43 (d, 2H, *J* = 5.5 Hz), 6.70 (dd, 1H, *J* = 8.1 and 1.2 Hz), 6.95 (brt, 1H, *J* = 5.4 Hz0, 6.99 (dd, 1H, *J* = 7.8 and 1.2 Hz), 7.15 (t, 1H, *J* = 2.1 Hz), 7.22 (t, 1H, *J* = 8.1 Hz), 7.54 (d, 1H, *J* = 9.0 Hz), 7.59 (dd, 1H, *J* = 9.0 and 2.4 Hz), 8.09 (d, 1H, *J* = 2.3 Hz), 8.57 (s, 1H) and 9.21 (s, 1H) ppm.; ^13^C NMR (75 MHz, DMSO*-d_6_*) δ 154.8, 148.7, 146.2, 140.0, 137.5, 131.8, 130.2, 126.6 (q, *J* = 30.0 Hz), 122.9 (q, *J* = 271.5 Hz), 122.4, 121.5, 120.7, 116.2 (q, *J* = 5.3 Hz), 114.6, 107.8, 105.6 and 34.9 ppm.; ^19^F NMR (282 MHz, DMSO*-d_6_*) δ −63.55 (s, 3F) ppm.; HRMS (ESI+): *m/z* = 411.0888 [M+H]^+^; calcd 411.0948 for [(C_17_H_14_ClF_3_N_6_O)+H]^+^.

*1-((1-(4-aminophenyl)-1H-1,2,3-triazol-4-yl)methyl)-3-(4-chloro-3-(trifluoromethyl)phenyl)urea (**2r’**).* The crude residue was pre-absorbed on silica gel and purified by flash sili-ca gel column chromatography using a mixture of 60% ethyl acetate in hexane as an elu-ent to provide (89 mg, 94% yield) the compound **2r’** as a brown solid; mp = 209–210 °C; ^1^H NMR (300 MHz, DMSO*-d_6_*) δ 4.41 (d, 2H, *J* = 5.5 Hz), 6.73 (d, 2H, *J* = 8.8 Hz), 6.90 (brt, 1H, *J* = 5.6 Hz), 7.50 (d, 2H, *J* = 8.8 Hz), 7.54 (d, 1H, *J* = 8.8 Hz), 7.59 (dd, 1H, *J* = 8.9 and 2.3 Hz), 8.10 (d, 1H, *J* = 2.2 Hz), 8.39 (s, 1H) and 9.14 (s, 1H) ppm.; ^13^C NMR (75 MHz, DMSO*-d_6_*) δ 154.8, 147.7, 145.9, 140.0, 131.9, 127.0, 126.6 (q, *J* = 30.0 Hz), 122.9 (q. *J* = 271.5 Hz), 122.5, 121.5 (3C), 120.6, 116.2 (q, *J* = 5.3 Hz), 114.9 (2C) and 34.9 ppm.; ^19^F NMR (282 MHz, DMSO*-d_6_*) δ −63.94 (s, 3F) ppm.; HRMS (ESI+): *m/z* = 411.0941 [M+H]^+^; calcd 411.0948 for [(C_17_H_14_ClF_3_N_6_O)+H]^+^.

#### 3.1.6. General Procedure for the Preparation of Sorafenib Derivatives **2s’** and **2t’**

A solution of compound **2p’** or **2q’** (100 mg, 0.24 mmol, 1.00 eq) and acetic anhydride (28 µL, 0.29 mmol, 1.20 eq) in the mixture of dichloromethane and tetrahydrofuran (1:1; 0.24 M, 1.0 mL) was stirred at room temperature for 10 min. After that, the reaction solution was diluted with water (30 mL) and extracted with ethyl acetate (3 × 30 mL). The combined organic phase was dried over anhydrous sodium sulfate and filtered. The solvent was removed under reduced pressure and the crude product was purified by column chromatography (silica gel, ethyl acetate in hexane) to obtain compound **2s’** or **2t’**.

*N-(2-(4-((3-(4-chloro-3-(trifluoromethyl)phenyl)ureido)methyl)-1H-1,2,3-triazol-1-yl)phenyl)acetamide (**2s’**).* The crude residue was pre-absorbed on silica gel and purified by flash silica gel column chromatography using a mixture of pure ethyl acetate as an eluent to provide (108 mg, 99% yield) the compound **2s’** as a beige-colored solid; mp = 189–190 °C; ^1^H NMR (300 MHz, DMSO*-d_6_*) δ 1.89 (s, 3H), 4.44 (d, 2H, *J* = 5.4 Hz), 6.91 (brt, 1H, *J* = 5.6 Hz), 7.39 (td, 1H, *J* = 7.5 and 1.2 Hz), 7.47–7.61 (m, 4H), 7.63 (dd, 1H, *J* = 8.1 and 1.2 Hz), 8.11 (d, 1H, *J* = 1.8 Hz), 8.21 (s, 1H), 9.17 (s, 1H) and 9.60 (brs, 1H) ppm.; ^13^C NMR (75 MHz, DMSO*-d_6_*) δ 168.8, 154.8, 145.5, 140.0, 131.8, 131.5, 131.3, 129.6, 127.4, 126.6 (q, *J* = 20.8 Hz), 126.4, 125.7, 123.7, 122.9 (q, *J* = 271.5 Hz), 122.4, 121.5, 116.2 (q, *J* = 6.0 Hz), 34.8 and 22.9 ppm.; ^19^F NMR (282 MHz, DMSO*-d_6_*) δ −63.10 (s, 3F) ppm.; HRMS (ESI+): *m/z* = 453.1000 [M+H]^+^; calcd 453.1054 for [(C_19_H_17_ClF_3_N_6_O_2_)+H]^+^.

*N-(3-(4-((3-(4-chloro-3-(trifluoromethyl)phenyl)ureido)methyl)-1H-1,2,3-triazol-1-yl)phenyl)acetamide (**2t’**).* The crude residue was pre-absorbed on silica gel and purified by flash silica gel column chromatography using a mixture of pure ethyl acetate as an eluent to provide (104 mg, 96% yield) the compound **2t’** as a light-brown solid; mp = 241–242 °C; ^1^H NMR (300 MHz, DMSO*-d_6_*) δ 2.08 (s, 3H), 4.45 (d, 2H, *J* = 5.3 Hz), 6.92 (brt, 1H, *J* = 5.1 Hz), 7.41–7.65 (m, 5H), 8.09 (d, 1H, *J* = 2.0 Hz), 8.27 (s, 1H), 8.57 (s, 1H), 9.13 (s, 1H) and 10.23 (brs, 1H) ppm.; ^13^C NMR (75 MHz, DMSO*-d_6_*) δ 168.8, 154.8, 146.5, 140.6, 140.0, 136.9, 131.8, 130.2, 126.6 (q, *J* = 30.0 Hz), 122.9 (q, *J* = 271.5 Hz), 122.4, 121.6 (q, *J* = 1.5 Hz), 120.9, 118.7, 116.3 (q, *J* = 6.0 Hz), 114.4, 110.4, 34.9 and 24.1 ppm.; ^19^F NMR (282 MHz, DMSO*-d_6_*) δ −62.86 (s, 3F) ppm.; HRMS (ESI+): *m/z* = 453.1054 [M+H]^+^; calcd 453.1054 for [(C_19_H_17_ClF_3_N_6_O_2_)+H]^+^.

#### 3.1.7. General Procedure for the Preparation of Sorafenib Derivatives **2v’** and **2w’**

Triazole-cored derivatives **2v’** and **2w’** were prepared according to the procedure described previously [91]. The solution of compound **2n’**or **2o’** (100 mg, 0.23 mmol, 1.00 eq) and 1-[bis(dimethylamino)methylene]-1H-1,2,3-triazolo [4,5-b]pyridinium-3-oxide hex-afluorophosphate (HATU) (95 mg, 0.25 mmol, 1.10 eq) in dry dimethylformamide (0.11 M, 2.1 mL) was stirred at room temperature for 20 min. After that, methylamine hydrochloride (18 µL, 0.46 mmol, 2.00 eq) and *N*,*N-*diisopropylethylamine (DIPEA) (120 µL, 0.69 mmol, 3.00 eq) were added. The reaction mixture was stirred at room temperature for 18 h. Then, the reaction solution was diluted with water (100 mL) followed by a saturated aqueous sodium chloride solution (5.0 mL) and then extracted with ethyl acetate (3 × 30 mL). The combined organic phase was repeatedly washed with a saturated aqueous sodium chloride solution (2 × 30 mL), dried over anhydrous sodium sulfate, and filtered. The solvent was removed under reduced pressure and the crude product was purified by column chromatography (silica gel, ethyl acetate in hexane) to obtain compound **2v’** or **2w’**.

*3-(4-((3-(4-chloro-3-(trifluoromethyl)phenyl)ureido)methyl)-1H-1,2,3-triazol-1-yl)-N-methylbenzamide (**2v’**).* The crude residue was pre-absorbed on silica gel and purified by flash silica gel column chromatography using a mixture of 80% ethyl acetate in hexane as an eluent to provide (80 mg, 77% yield) the compound **2v’** as a white solid; mp = 191–192 °C; ^1^H NMR (300 MHz, DMSO*-d_6_*) δ 2.81 (d, 3H, *J* = 4.5 Hz), 4.46 (d, 2H, *J* = 5.3 Hz), 6.96 (brt, 1H, *J* = 5.4 Hz), 7.54 (d, 1H, *J* = 8.8 Hz), 7.59 (dd, 1H, *J* = 8.9 and 2.3 Hz), 7.67 (t, 1H, *J* = 7.9 Hz), 7.91 (brd, 1H, *J* = 7.9 Hz), 8.05 (brdd, 1H, *J* = 8.0 and 1.3 Hz), 8.10 (d, 1H, *J* = 2.2 Hz), 8.33 (t, 1H, *J* = 1.7 Hz), 8.66 (brd, 1H, *J* = 4.4 Hz), 8.71 (s, 1H) and 9.18 (s, 1H) ppm.; ^13^C NMR (75 MHz, DMSO*-d_6_*) δ 165.5, 154.9, 146.8, 140.0, 136.7, 136.1, 131.9, 130.1, 127.1, 126.6 (q, *J* = 30.8 Hz), 122.9 (q, *J* = 271.5 Hz), 122.5, 122.4, 121.6, 121.1, 118.6, 116.3 (q, *J* = 6.0 Hz), 34.9 and 26.4 ppm.; ^19^F NMR (282 MHz, DMSO*-d_6_*) δ −62.99 (s, 3F) ppm.; HRMS (ESI+): *m/z* = 453.1038 [M+H]^+^; calcd 453.1054 for [(C_19_H_16_ClF_3_N_6_O_2_)+H]^+^.

*4-(4-((3-(4-chloro-3-(trifluoromethyl)phenyl)ureido)methyl)-1H-1,2,3-triazol-1-yl)-N-methylbenzamide (**2w’**)*. The crude residue was pre-absorbed on silica gel and purified by flash silica gel column chromatography using a mixture of 80% ethyl acetate in hexane as an eluent to provide (75 mg, 72% yield) the compound **2w’** as a white solid; mp = 243–244 °C; ^1^H NMR (300 MHz, DMSO*-d_6_*) δ 2.81 (d, 3H, *J* = 4.4 Hz), 4.46 (d, 2H, *J* = 5.3 Hz), 6.94 (brt, 1H, *J* = 5.2 Hz), 7.54 (d, 1H, *J* = 9.0 Hz), 7.59 (dd, 1H, *J* = 9.0 and 2.1 Hz), 8.02 (s, 4H), 8.10 (d, 1H, *J* = 2.1 Hz), 8.58 (brd, 1H, *J* = 4.4 Hz), 8.75 (s, 1H) and 9.16 (s, 1H) ppm.; ^13^C NMR (75 MHz, DMSO*-d_6_*) δ 165.5, 154.8, 146.8, 140.0, 138.3, 134.2, 131.8, 128.8 (2C), 126.6 (q, *J* = 30.8 Hz), 122.9 (q, *J* = 217.5 Hz), 122 4, 121.5 (q, *J* = 1.5 Hz), 121.0, 119.5 (2C), 116.2 (q, *J* = 5.3 Hz), 30.8 and 26.3 ppm.; ^19^F NMR (282 MHz, DMSO*-d_6_*) δ −63.08 (s, 3F) ppm.; HRMS (ESI+): *m/z* = 453.1016 [M+H]^+^; calcd 453.1054 for [(C_19_H_16_ClF_3_N_6_O_2_)+H]^+^.

### 3.2. Cytotoxicity

The HepG2 cell line was provided by Dr. Praneet Opanasopit. The A549, MOLT-3, HL-60, and MRC-5 cell lines were obtained from the American Type Culture Collection (ATCC, Manassas, VA, USA). The HuCCA-1 cell line was obtained from the Immunology lab, Siriraj Hospital, Bangkok, Thailand.

Cells were seeded at a density of 2 × 104 cells/well for A549, HuCCA-1, and MRC-5, and 8 × 104 cells/well for HepG2, onto a 96-well plate in the corresponding cell culture medium (100 mL for adherent cells) and maintained at 37 °C with 95% humidity and 5% CO2 for 24 h to allow attachment. For A549 and HuCCA-1, cells were grown in Ham’S/F12 (Hyclone Laboratories) medium containing 2 mM L-glutamine (Sigma, St. Louis, MO, USA) supplement with 100 U/mL penicillin–streptomycin (Sigma) and 10% fetal bovine serum (JR Scientific, Inc. Woodland, CA, USA). For HepG2, the cells were maintained in DMEM (Gibco, Waltham, MA, USA) supplemented with 10% FBS (Gibco), 1% non-essential amino acids, 1% GlutaMAX (Gibco), 100 units/mL penicillin, and 100 µg/mL streptomycin. For MRC-5, cells were grown in DMEM (Hyclone Laboratories) medium containing a supplement with 100 U/mL penicillin-streptomycin (Sigma) and 10% fetal bovine serum (JR Scientific, Inc. Woodland, CA, USA). Afterward, 100 µL of media containing serial 2-fold diluted compounds were added to each well to a final concentration of 100–3.125 µM. DMSO was used as vehicle control. Cells were exposed for 48 h for A549, HuCCA-1, and MRC-5 cells and for 72 h for HepG2 cells to the synthetic compounds and positive controls (Sorafenib and Doxorubicin) and cell viability was determined by MTT assay. Briefly, cells were washed with phosphate buffer saline (PBS) solution then incubated with 1 mg/mL thiazolyl blue tetrazolium bromide (Sigma-Aldrich, St. Louis, MO, USA) for 4 h. Afterward, the supernatant was discarded and 100 µL of DMSO was added to each well and mixed to dissolve the formazan crystals. The absorbance at 570 nm was measured using a microplate reader (Packard Bioscience). All experiments were performed in triplicate. Data were expressed as the IC50 ± SD. The IC50 was calculated by a non-linear regression analysis using the scientific statistic software GraphPad Prism version 7 (GraphPad Software Inc., La Jolla, CA, USA) [92,93,94]. The cytotoxicity investigation of the synthetic compounds toward MOLT-3 and HL-60 cells was conducted by XTT assay [95]. MOLT-3 and HL-60 cells were seeded at a density of 5 × 104 cells/well onto a 96-well plate and incubated for 24 h. Cells were exposed for 48 h. Plates were incubated for 4 h after the addition of a 50 µL mixture of 1 mg/mL of XTT solution (5 mL) and 0.383 mg/mL of PMS (100 µL). The absorbance of the orange formazan compounds formed was measured at wavelengths of 492 nm and 690 nm.

### 3.3. Selectivity Index (SI)

The Selectivity Index (SI) was calculated from the IC50 toward normal cell (MRC-5) to the IC50 toward HepG2. The SI values were ≥3.00, indicating high cancer selectivity [64,66].

(1) $$\mathrm{Selectivity}\;\mathrm{Index}\;(\mathrm{SI})=\frac{Cytotoxicity\;towards\;MRC-5}{Cytotoxicity\;towards\;HepG2}$$

### 3.4. Cell-Cycle Analysis

HepG2 cells were seeded into 6-well plates at a density of 2 × 10^5^ cells/well and incubated at 37 °C in the presence of 5% CO_2_ for 24 h to allow attachment. Afterward, cells were treated with complete media containing Sorafenib, compounds **2m’** and **2e** at 2 µM, or DMSO as vehicle control for 72 h. The concentration of DMSO was kept at 0.5% *v/v* for all conditions. Cells were washed with PBS before they were harvested; 1 × 10^5^ Cells were collected and fixed with 70% ice-cold ethanol, and then washed twice with ice-cold PBS. Afterward, the cells were treated with 100 g/mL of DNase-free RNase A (Sigma-Aldrich, St. Louis, MO, USA) in PBS containing 0.1% *v/v* Triton-X 100 (Sigma-Aldrich, St. Louis, MO, USA) for 5 min at room temperature and then stained with 20 g/mL propidium iodide (Life Technologies, Carlsbad, CA, USA) in PBS containing 0.1% *v/v* Triton-X 100 for 15 min at room temperature while protected from light. Cell-cycle distribution was then analyzed with a flow cytometer (Attune NxT, Thermo Fischer Scientific, Waltham, MA, USA). Data were analyzed with Attune NxT Software (Thermo Fischer Scientific, Waltham, MA, USA). All experiments were performed in triplicate. Student’s *t*-test was used for statistical analysis; *p* < 0.05 was considered statistically significant [96].

### 3.5. Detection of Apoptosis

The detection of apoptosis was performed by the Muse^®^ Annexin V & Dead Cell Kit according to the manufacturer’s protocol (Millipore, Billerica, MA, USA). HepG2 cells at 5 × 10^5^ cells/mL in a completed DMEM medium were seeded into 24-well plates and incubated at 37 °C in the presence of 5% CO_2_ for 24 h. The medium was removed from the plates, followed by treatment with Sorafenib, **2m’**, and **2e** at concentrations of 1.25 µM, 2.5 µM, 5.0 µM, and 10.0 µM for 48 h. After incubation, the cells were washed with 300 µL PBS/well and removed from plates by Trypsinization (300 µL of trypsin/well). An amount of 100 µL of HepG2 cells in suspension and 100 µL of Muse Annexin V & Dead Cell reagent were added to a 1.5 mL tube. The apoptosis was measured using the Muse cell analyzer and Muse analysis software (Millipore, Billerica, MA, USA). Cells were classified into four groups: live (Annexin V− and 7-AAD−), early apoptosis (Annexin V+ and 7-AAD−), late apoptosis (Annexin V+ and 7-AAD+), and dead or necrotic (Annexin V− and 7-AAD+). The apoptosis experiment was performed in duplicate.

### 3.6. Physicochemical Property Methodology

All drug-likeness properties and Lipinski’s rule of five were obtained by using SwissADME website services [70].

## 4. Conclusions

A new series of 1,2,3-triazole-cored analogs, in which the core phenoxy ring and picolinamide ring of Sorafenib were replaced with 1,2,3-triazole linking a substituted phenyl ring, were synthesized successfully via nucleophilic addition and 1,3-dipolar cycloaddition and evaluated for their in vitro anti-cancer activity against five different cancer cell lines. The synthetic triazole-cored analogs exhibited inhibitory activities toward HepG2 dominantly over other cancer cell lines. Analogs **2m’** (R = *p*-*t*Bu) and **2e** (R = *o*-Cl) exhibited similar anti-HepG2 properties to that of Sorafenib, but were less active than a chemotherapy drug, Doxorubicin. In addition, **2m’** (R = *p*-*t*Bu) and **2e** (R = *o*-Cl) showed 4.4- and 3.7-fold superior SIs to that of Sorafenib and were 3.8- and 3.2-fold superior to that of Doxorubicin. Disappointingly, the analogs with a functional group capable of forming hydrogen bonds in the hinge region did not show potent anti-HepG2 activity, as expected. For cell-cycle analysis on HepG2, both compounds caused an increased number of cells in the S and G2/M phases, similar to those of Sorafenib, suggesting that they could share a similar mechanism of action. The induction of apoptotic cell death was observed in the treated HepG2 with **2m’** and **2e** in a dose-dependent manner at 48 h, similar to that of the Sorafenib case. The cytotoxic effect of the candidates might be due to the inhibition of kinases and/or other proteins involving apoptosis cell death and/or even affecting other mechanisms of action. Further investigations should be carried out.

Evidently, the replacement of the core phenoxy and picolinamide ring of Sorafenib with a 1,2,3-triazole ring linking an *ortho*- and *para*-substituted phenyl ring with electron-withdrawing and bulky alkyl groups could still maintain anti-HepG2 activity. In addition, the presence of 1,2,3-triazole was proven to promote the compounds’ SI, which agrees with a previous report [4]. Therefore, the 1,2,3-triazole linking a substituted benzene was an interesting structural feature for selective anti-HepG2 agents with a high safety profile. Furthermore, these two compounds (**2m’** and **2e**) displayed good physicochemical profiles, especially compound **2e**, which possessed several parameters superior to those of Sorafenib. Therefore, this study identified promising candidates for further development as targeted HCC drugs and drugs used in combination therapy with other anti-HCC drugs in the clinic.

## Figures and Tables

**Table 1:** In vitro cytotoxicity toward HepG2 and MRC-5 of the synthesized triazole derivatives, indicated as cell viability percentage at 25 µM and half-maximal inhibitory concentration (IC_50_) at a µM scale. The selectivity index (SI) was calculated.

Compound	R	Cytotoxicity			SI
		HepG2		MRC-5	
		Cell Viability at 25 µM	IC_50_ (µM)	IC_50_ (µM)	
**2a**	H	83%	-	>100	-
**2b**	*o*-F	79%	-	73.5 ± 9.14	-
**2c**	*m*-F	60%	-	61.6 ± 3.63	-
**2d**	*p*-F	58%	-	59.3 ± 1.96	-
**2e**	*o*-Cl	53%	5.02 ± 2.07	61.4 ± 3.51	12.2
**2f**	*m*-Cl	52%	9.81 ± 5.02	63.8 ± 3.53	6.50
**2g**	*p*-Cl	72%	-	14.6 ± 4.17	-
**2h**	*o*-Br	61%	-	52.9 ± 3.10	-
**2i**	*m*-Br	81%	-	53.0 ± 3.31	-
**2j**	*p*-Br	75%	-	60.7 ± 1.88	-
**2k**	*o*-I	60%	-	49.1 ± 2.95	-
**2l**	*m*-I	88%	-	49.2 ± 2.13	-
**2m**	*p*-I	63%	-	49.9 ± 6.61	-
**2n**	*o*-OH	75%	-	>100	-
**2o**	*m*-OH	89%	-	>100	-
**2p**	*p*-OH	66%	-	69.6 ± 5.66	-
**2q**	*o*-OMe	74%	-	68.0 ± 5.14	-
**2r**	*m*-OMe	76%	-	76.1 ± 9.18	-
**2s**	*p*-OMe	64%	-	21.2 ± 2.51	-
**2t**	*o*-Me	72%	-	61.7 ± 3.22	-
**2u**	*m*-Me	69%	-	>100	-
**2v**	*p*-Me	91%	-	99.9 ± 0.90	-
**2w**	*o*-CF_3_	58%	-	58.8 ± 0.39	-
**2x**	*m*-CF_3_	81%	-	63.2 ± 1.57	-
**2y**	*p*-CF_3_	39%	5.97 ± 2.14	58.6 ± 3.51	9.81
**2z**	*o*-CN	66%	-	59.7 ± 6.63	-
**2a’**	*m*-CN	59%	-	99.3 ± 2.99	-
**2b’**	*p*-CN	113%	-	64.1 ± 0.36	-
**2c’**	*o*-NO_2_	78%	-	60.6 ± 5.26	-
**2d’**	*m*-NO_2_	62%	-	58.4 ± 5.69	-
**2e’**	*p*-NO_2_	70%	-	66.1 ± 4.50	-
**2f’**	*o*-Et	60%	-	57.7 ± 1.82	-
**2g’**	*m*-Et	79%	-	94.7 ± 5.24	-
**2h’**	*p*-Et	63%	-	75.4 ± 10.43	-
**2i’**	*o*-*i*Pr	29%	5.40 ± 0.35	54.5 ± 7.03	10.1
**2j’**	*m*-*i*Pr	62%	-	73.3 ± 15.60	-
**2k’**	*p*-*i*Pr	50%	20.95 ± 4.17	65.9 ± 10.21	3.15
**2l’**	*m*-*t*Bu	46%	9.88 ± 3.03	68.2 ± 11.91	6.91
**2m’**	*p*-*t*Bu	44%	5.57 ± 0.91	81.6 ± 5.07	14.7
**2n’**	*m*-CO_2_H	96%	-	>100	-
**2o’**	*p*-CO_2_H	81%	-	>100	-
**2p’**	*o*-NH_2_	65%	-	>100	-
**2q’**	*m*-NH_2_	57%	-	62.3 ± 3.38	-
**2r’**	*p*-NH_2_	79%	-	>100	-
**2s’**	*o*-NHAc	60%	-	53.6 ± 2.54	-
**2t’**	*m*-NHAc	93%	-	>100	-
**2u’**	*p-*NHAc	76%	-	>100	-
**2v’**	*m*-CONHMe	74%	-	>100	-
**2w’**	*p*-CONHMe	61%	-	62.2 ± 7.88	-
Doxorubicin		-	0.59 ± 0.10	2.3 ± 0.22	3.83
Sorafenib (**1**)		-	5.97 ± 0.71	19.7 ± 1.68	3.30

**Table 2:** The physicochemical properties of active compounds **2m’** and **2e** compared with Sorafenib.

Compounds	^a^MW	^b^TPSA	^c^CLog P	^d^Log S	^e^nHBA	^f^nHBD	^g^nVio	^h^nRB
**2m’**	451.87	71.84	4.74	−6.32	6	2	1	8
**2e**	430.21	71.84	4.01	−5.23	6	2	0	7
Sorafenib	464.82	92.35	4.11	−5.71	7	3	0	9

^a^MW = molecular weight; ^b^TPSA = Topological Polar Surface Area (Å^2^); ^c^CLog P = Consensus Log P_o/w_ (lipophilicity parameter); ^d^Log S = water solubility parameter, Log S scale insoluble < −10 < poorly < −6 < moderately < −4 < soluble < −2 very < 0 <highly (topological method implemented from Ali, J. et al. **2012**, *J. Chem. Inf. Model*.); ^e^nHBA = Number of Hydrogen Bond Acceptors; ^f^nHBD = Number of Hydrogen Bond Donors; ^g^nVio = number of violations to Lipinski’s rule of five (drug-likeness); ^h^nRB = number of rotatable bonds.

## Data Availability

Data is contained within the article and Supplementary Material.

## Supplementary Materials

The following supporting information can be downloaded at https://www.mdpi.com/article/10.3390/ph15050504/s1: Characteristics and spectroscopic data of alkynes **5** and various azidobenzenes **7**; Table S1: The cytotoxicity of Sorafenib derivatives toward various human lung carcinoma cell lines A549, Thai human cholangiocarcinoma cells HuCCA-1, T-cell acute lymphoblastic leukemia MOLT-3, and acute promyelocytic leukemia HL-60. The inhibitory activities are indicated as IC_50_ at the micromolar scale (µM). The calculated selectivity indices (SI) are reported in square brackets; References for azides; NMR spectra.
