# Supplementary material for: The Design and Synthesis of a New Series of 1,2,3-Triazole-Cored Structures Tethering Aryl Urea and Their Highly Selective Cytotoxicity toward HepG2
Source: Pharmaceuticals (Basel). 2022 Apr 20;15(5):504. doi: 10.3390/ph15050504 (PMC9147274; doi:10.3390/ph15050504)

Supporting Information

The Design and Synthesis of a New Series of 1,2,3-Triazole-Cored Structures Tethering Aryl Urea and Their Highly Selective Cytotoxicity toward HepG2

Sittisak Oekchuae[a,b], Jitnapa Sirirak[a], Purin Charoensuksai[c], Pawaris Wongprayoon[c], Natthaya Chuaypen[d], Jutatip Boonsombat[b,e], Somsak Ruchirawat[b,e,f], Pisit Tangkijvanich[d], Apichart Suksamrarn[g], and Panupun Limpachayaporn[a,]*

[a] *Department of Chemistry, Faculty of Science, Silpakorn University, Nakhon Pathom 73000, Thailand*

[b] *Chulabhorn Research Institute, Bangkok 10210, Thailand*

[c] *Department of Biopharmacy and Bioactives from Natural Resources Research Collaboration for Excellence in Pharmaceutical Sciences, Faculty of Pharmacy, Silpakorn University, Nakhon Pathom, 73000, Thailand*

[d] *Center of Excellence in Hepatitis and Liver Cancer, Department of Biochemistry, Faculty of Medicine, Chulalongkorn University, Bangkok 10330, Thailand*

[e] *Center of Excellence on Environmental Health and Toxicology (EHT), OPS, MHESI, Bangkok 10400, Thailand*

[f] *Program in Chemical Sciences, Chulabhorn Graduate Institute, Chulabhorn Royal Academy, Bangkok 10210, Thailand*

[g] *Department of Chemistry and Center of Excellence for Innovation in Chemistry, Faculty of Science, Ramkhamhaeng University, Bangkok 10240, Thailand*

*Corresponding author: Panupun Limpachayaporn

Mailing address: Department of Chemistry, Faculty of Science, Silpakorn University, Nakhon Pathom 73000, Thailand

E-mail: limpachayaporn_p@su.ac.th or panupun.lim@gmail.com; Tel.: +66-34-255797; Fax: +66-34-271356

Table of Contents

1. Characteristics and spectroscopic data of alkyne **5** and various azidobenzenes **7**............ S-3

2. Biological activities toward A549, HuCCA-1, MOLT-3, and HL-60 cancer cell lines .. S-19

3. Reference for azides.. S-23

4. NMR Spectra S-25

1. Characteristics and spectroscopic data of alkyne 5 and various azidobenzenes 7

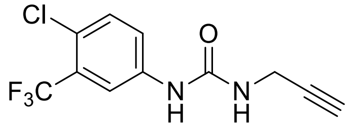

*1-(4-chloro-3-(trifluoromethyl)phenyl)-3-(prop-2-yn-1-yl)urea (**5**).* mp = 122-123 ºC; ^{1}H NMR (300 MHz, acetone-d_6) δ 3.07 (s, 1H), 4.02 (dd, 2H, *J* = 5.6 and 2.4 Hz), 6.31 (brs, 1H), 7.48 (d, 1H, *J* = 8.7 Hz), 7.68 (dd, 1H, *J* = 8.7 and 2.0 Hz), 8.08 (d, 1H, *J* = 2.2 Hz) and 8.48 (brs, 1H) ppm.; ^{13}C NMR (75 MHz, acetone-d_6) δ 155.4, 140.7, 132.6, 128.4 (q, *J* = 30.8 Hz), 123.9 (q, *J* = 270.8 Hz), 123.8, 123.4, 117.8 (q, *J* = 6.0 Hz), 81.8, 71.9 and 29.8 ppm.; ^{19}F NMR (282 MHz, $CDCl_3$) δ -63.59 (s, 3F) ppm.; HRMS (ESI+): *m/z* = 299.0169 $[M+Na]^+$; calcd 299.0175 for $[(C_{11}H_8ClF_3N_2O)+Na]^+$.

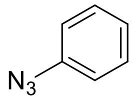

*azidobenzene (**7a**).*[74-75] Following the procedure B using aniline (548 µL, 6.00 mmol, 1.00 eq), sodium nitrite (1.50 M; 1.24 g, 18.00 mmol, 3.00 eq), sodium azide (1.50 M; 780 mg, 12.00 mmol, 2.00 eq) and sodium acetate (1.50 M; 984 mg, 12.00 mmol, 2.00 eq) in 50% HCl (0.74 M, 8.1 mL) at 0 ºC for 1 h to give compound **7a** as a black oil (715 mg, 100% yield) which was used without purification. IR (neat) 2125, 2094, 1594, 1491, 1293, and 1282 cm^{-1}; ^{1}H NMR (300 MHz, $CDCl_3$) δ 6.94 (brdd, 2H, *J* = 8.7 and 1.2 Hz), 7.06 (brtd, 1H, *J* = 8.4 and 1.2 Hz) and 7.26 (brt, 2H, *J* = 8.1 Hz) ppm.; ^{13}C NMR (75 MHz, $CDCl_3$) δ 140.1, 129.8 (2C), 124.9 and 119.1 (2C) ppm.

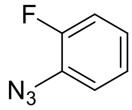

*1-azido-2-fluorobenzene (**7b**).*[74-75] Following the procedure B using 2-fluoroaniline (579 µL, 6.00 mmol, 1.00 eq), sodium nitrite (1.50 M; 1.24 g, 18.00 mmol, 3.00 eq), sodium azide (1.50 M; 780 mg, 12.00 mmol, 2.00 eq) and sodium acetate (1.50 M; 984 mg, 12.00 mmol, 2.00 eq) in 50% HCl (0.74 M, 8.1 mL) at 0 ºC for 1 h to give compound **7b** as a brown oil (816 mg, 99% yield) which was used without purification. IR (neat) 2117, 2105, 1493, 1314,1297, 1228, and 1096 cm^{-1}; ^{1}H NMR (300 MHz, DMSO-d_6) δ 7.10-7.26 (m, 4H) ppm.; ^{13}C NMR (75 MHz, DMSO-d_6) δ 154.3 (d, *J* = 245.3 Hz), 127.5 (d, *J* = 10.5 Hz), 126.7 (d, *J* = 7.50 Hz),

125.9 (d, J = 3.8 Hz), 121.6 and 117.1 (d, J = 18.0 Hz); ^{19}F NMR (282 MHz, DMSO-d_6) δ -128.75 (s, 1F) ppm.

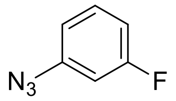

*1-azido-3-fluorobenzene (7**c**)*. [75] Following the procedure B using 3-fluoroaniline (577 μL, 6.00 mmol, 1.00 eq), sodium nitrite (1.50 M; 1.24 g, 18.00 mmol, 3.00 eq), sodium azide (1.50 M; 780 mg, 12.00 mmol, 2.00 eq) and sodium acetate (1.50 M; 984 mg, 12.00 mmol, 2.00 eq) in 50% HCl (0.74 M, 8.1 mL) at 0 °C for 1 h to give compound **7c** as a deep brown oil (790 mg, 96% yield) which was used without purification. IR (neat) 2109, 1589, 1485, 1296, and 1209 cm^{-1}; ^{1}H NMR (300 MHz, $CDCl_3$) δ 6.73 (dt, 1H, J = 9.6 and 2.4 Hz), 6.83 (dd, 1H, J = 8.1 and 2.1 Hz), 6.85 (dddd, 1H, J = 11.1, 9.0, 2.4 and 0.6 Hz) and 7.30 (ddd, 1H, J = 14.7, 8.1 and 6.3 Hz) ppm.; ^{13}C NMR (75 MHz, $CDCl_3$) δ 163.5 (d, J = 246.0 Hz), 141.9 (d, J = 9.8 Hz), 142.0 (d, J = 9.8 Hz), 114.7 (d, J = 3.0 Hz), 111.9 (d, J = 21.0 Hz) and 106.8 (d J = 25.5 Hz); ^{19}F NMR (282 MHz, DMSO-d_6) δ -113.00 (s, 1F) ppm.

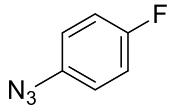

*1-azido-4-fluorobenzene (7**d**)*. [75] Following the procedure B using 4-fluoroaniline (568 μL, 6.00 mmol, 1.00 eq), sodium nitrite (1.50 M; 1.24 g, 18.00 mmol, 3.00 eq), sodium azide (1.50 M; 780 mg, 12.00 mmol, 2.00 eq) and sodium acetate (1.50 M; 984 mg, 12.00 mmol, 2.00 eq) in 50% HCl (0.74 M, 8.1 mL) at 0 °C for 1 h to give compound **7d** as a brown black oil (814 mg, 99% yield) which was used without purification. IR (neat) 2107, 2067, 1498, 1302, 1295, 1225, and 1097 cm^{-1}; ^{1}H NMR (300 MHz, $CDCl_3$) δ 6.94-7.10 (m, 4H) ppm.; ^{13}C NMR (75 MHz, $CDCl_3$) δ 160.0 (d, J = 242.7 Hz), 135.8, (d, J = 2.9 Hz), 120.3 (2C, d, J = 8.2 Hz) and 116.6 (2C, d, J = 23.1 Hz); ^{19}F NMR (282 MHz, $CDCl_3$) δ -118.46 (s, 1F) ppm.

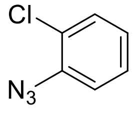

*1-azido-2-chlorobenzene (7**e**)*. [76] Following the procedure B using 2-chloroaniline (933 μL, 6.00 mmol, 1.00 eq), sodium nitrite (1.50 M; 1.24 g, 18.00 mmol, 3.00 eq), sodium azide (1.50 M; 780 mg, 12.00 mmol, 2.00 eq) and sodium acetate (1.50 M; 984 mg, 12.00 mmol, 2.00 eq) in 50% HCl (0.74 M, 8.1 mL) at 0 °C for 1 h to give compound **7e** as a brown oil (875 mg,

95% yield) which was used without purification. IR (neat) 2132, 2101, 1585, 1476, 1442, 1302, 1131, and 1051 cm^{-1}; ^{1}H NMR (300 MHz, $CDCl_3$) δ 7.07 (td, 1H, J = 7.8 and 1.5 Hz), 7.17 (dd, 1H, J = 8.1 and 1.5 Hz), 7.29 (td, 1H, J = 8.1 and 1.5 Hz) and 7.37 (dd, 1H, J = 8.1 and 1.5 Hz) ppm.; ^{13}C NMR (75 MHz, $CDCl_3$) δ 137.2, 130.7, 127.9, 125.6, 125.0 and 119.7 ppm.

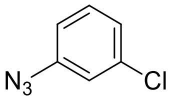

*1-azido-3-chlorobenzene (7**f**).* [77] Following the procedure B using 3-chloroaniline (933 μL, 6.00 mmol, 1.00 eq), sodium nitrite (1.50 M; 1.24 g, 18.00 mmol, 3.00 eq), sodium azide (1.50 M; 780 mg, 12.00 mmol, 2.00 eq) and sodium acetate (1.50 M; 984 mg, 12.00 mmol, 2.00 eq) in 50% HCl (0.74 M, 8.1 mL) at 0 °C for 1 h to give compound **7f** as a light brown oil (875 mg, 95% yield) which was used without purification. IR (neat) 2098, 1590, 1579, 1475, 1283, 1257, and 1140 cm^{-1}; ^{1}H NMR (300 MHz, $CDCl_3$) δ 6.90 (ddd, 1H, J = 8.1, 2.1 and 0.9 Hz), 7.00 (t, 1H, J = 2.1 Hz), 7.10 (ddd, 1H, J = 8.1, 2.1 and 0.9 Hz) and 7.26 (t, 1H, J = 8.1 Hz) ppm.; ^{13}C NMR (75 MHz, $CDCl_3$) δ 141.5, 135.5, 130.6, 125.0, 119.0 and 117.2 ppm.

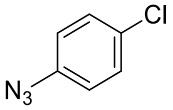

*1-azido-4-chlorobenzene (7**g**).* [74, 77] Following the procedure B using 4-chloroaniline (535 μL, 6.00 mmol, 1.00 eq), sodium nitrite (1.50 M; 1.24 g, 18.00 mmol, 3.00 eq), sodium azide (1.50 M; 780 mg, 12.00 mmol, 2.00 eq) and sodium acetate (1.50 M; 984 mg, 12.00 mmol, 2.00 eq) in 50% HCl (0.74 M, 8.1 mL) at 0 °C for 1 h to give compound **7g** as a light brown oil (912 mg, 99% yield) which was used without purification. IR (neat) 2126, 2089, 1485, 1293, 1269, 1129, 1091, and 1012 cm^{-1}; ^{1}H NMR (300 MHz, $CDCl_3$) δ 6.92 (d, 2H, J = 8.7 Hz) and 7.23 (d, 2H, J = 9.0 Hz) ppm.; ^{13}C NMR (75 MHz, $CDCl_3$) δ 138.7, 130.2, 129.82 (2C) and 120.2 (2C) ppm.

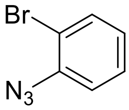

*1-azido-2-bromobenzene (7**h**).* [78] Following the procedure B using 2-bromoaniline (653 μL, 6.00 mmol, 1.00 eq), sodium nitrite (1.50 M; 1.24 g, 18.00 mmol, 3.00 eq), sodium azide (1.50 M; 780 mg, 12.00 mmol, 2.00 eq) and sodium acetate (1.50 M; 984 mg, 12.00 mmol, 2.00 eq)

in 50% HCl (0.74 M, 8.1 mL) at 0 °C for 1 h to give compound **7h** as a deep brown oil (1.19 g, 100% yield) which was used without purification. IR (neat) 2134, 2119, 2096, 1471, 1438, 1310, 1295, and 1029 cm^{-1}; ^1H NMR (300 MHz, $CDCl_3$) δ 6.99 (td, 1H, *J* = 7.9 and 1.5 Hz), 7.15 (dd, 1H, *J* = 8.0 and 1.5 Hz), 7.33 (td, 1H, *J* = 8.0 and 1.4 Hz) and 7.53 (dd, 1H, *J* = 8.0 and 1.4 Hz) ppm.; ^{13}C NMR (75 MHz, $CDCl_3$) δ 138.5, 133.7, 128.5, 125.9, 119.4 and 113.9 ppm.

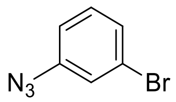

*1-azido-3-bromobenzene (7**i**).* [79] Following the procedure B using 3-bromoaniline (653 μL, 6.00 mmol, 1.00 eq), sodium nitrite (1.50 M; 1.24 g, 18.00 mmol, 3.00 eq), sodium azide (1.50 M; 780 mg, 12.00 mmol, 2.00 eq) and sodium acetate (1.50 M; 984 mg, 12.00 mmol, 2.00 eq) in 50% HCl (0.74 M, 8.1 mL) at 0 °C for 1 h to give compound **7i** as a deep brown oil (1.19 g, 100% yield) which was used without purification. IR (neat) 2133, 2098, 1587, 1585, 1470, 1306, 1283, 1257, and 1067 cm^{-1}; ^1H NMR (300 MHz, $CDCl_3$) δ 6.94 (ddd, 1H, *J* = 7.8, 2.2 and 1.3 Hz), 7.16 (t, 1H, *J* = 1.9 Hz), 7.19 (t, 1H, *J* = 7.8 Hz) and 7.25 (dt, 1H, *J* = 8.0 and 1.3 Hz) ppm.; ^{13}C NMR (75 MHz, $CDCl_3$) δ 141.3, 130.6, 127.8, 123.2, 122.0 and 117.5 ppm.

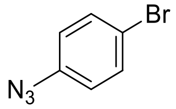

*1-azido-4-bromobenzene (7**j**).* [78] Following the procedure B using 4-bromoaniline (1.03 g, 6.00 mmol, 1.00 eq), sodium nitrite (1.50 M; 1.24 g, 18.00 mmol, 3.00 eq), sodium azide (1.50 M; 780 mg, 12.00 mmol, 2.00 eq) and sodium acetate (1.50 M; 984 mg, 12.00 mmol, 2.00 eq) in 50% HCl (0.74 M, 8.1 mL) at 0 °C for 1 h to give compound **7j** as a brown oil (1.19 g, 100% yield) which was used without purification. IR (neat) 2125, 2087, 1481, 1291, 1270, 1072, and 1009 cm^{-1}; ^1H NMR (300 MHz, $CDCl_3$) δ 6.95 (d, 2H, *J* = 8.7 Hz) and 7.41 (d, 2H, *J* = 9.0 Hz) ppm.; ^{13}C NMR (75 MHz, $CDCl_3$) δ 139.2, 132.7 (2C), 120.6 (2C) and 117.7 ppm.

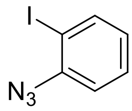

*1-azido-2-iodobenzene (7**k**).* [80] Following the procedure B using 2-iodoaniline (1.31 g, 6.00 mmol, 1.00 eq), sodium nitrite (1.50 M; 1.24 g, 18.00 mmol, 3.00 eq), sodium azide (1.50 M;

780 mg, 12.00 mmol, 2.00 eq) and sodium acetate (1.50 M; 984 mg, 12.00 mmol, 2.00 eq) in 50% HCl (0.74 M, 8.1 mL) at 0 °C for 1 h to give compound **7k** as a black brown oil (1.41 g, 96% yield) which was used without purification. IR (neat) 2127, 2109, 2090, 1578, 1464, 1434, 1303, 1288, and 1017 cm^{-1}; ^{1}H NMR (300 MHz, $CDCl_3$) δ 6.86 (td, 1H, J = 7.8 and 1.5 Hz), 7.13 (dd, 1H, J = 8.1 and 1.5 Hz), 7.38 (td, 1H, J = 7.5 and 1.5 Hz) and 7.78 (dd, 1H, J = 7.9 and 1.5 Hz) ppm.; ^{13}C NMR (75 MHz, $CDCl_3$) δ 140.8, 139.3, 128.9, 125.6, 117.8 and 87.7 ppm.

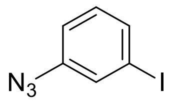

*1-azido-3-iodobenzene (7**l**).* [79] Following the procedure B using 3-iodoaniline (722 µL, 6.00 mmol, 1.00 eq), sodium nitrite (1.50 M; 1.24 g, 18.00 mmol, 3.00 eq), sodium azide (1.50 M; 780 mg, 12.00 mmol, 2.00 eq) and sodium acetate (1.50 M; 984 mg, 12.00 mmol, 2.00 eq) in 50% HCl (0.74 M, 8.1 mL) at 0 °C for 1 h to give compound **7l** as a black brown oil (1.46 g, 99% yield) which was used without purification. IR (neat) 2128, 2096, 1580, 1566, 1467, 1416, 1303, 1281, 1256, and 1058 cm^{-1}; ^{1}H NMR (300 MHz, $CDCl_3$) δ 6.98 (ddd, 1H, J = 8.1, 2.1 and 1.2 Hz), 7.06 (dd, 1H, J = 8.1 and 7.5 Hz), 7.37 (dd, 1H, J = 2.1 and 1.5 Hz) and 7.46 (dt, 1H, J = 7.5 and 1.5 Hz) ppm.; ^{13}C NMR (75 MHz, $CDCl_3$) δ 140.8, 133.5, 130.6, 127.6, 118.0 and 94.91 ppm.

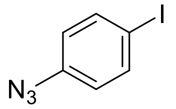

*1-azido-4-iodobenzene (7**m**).* [81] Following the procedure B using 4-iodoaniline (500 mg g, 2.28 mmol, 1.00 eq), sodium nitrite (1.50 M; 472 mg, 6.84 mmol, 3.00 eq), sodium azide (1.50 M; 297 mg, 4.56 mmol, 2.00 eq) and sodium acetate (1.50 M; 374 mg, 4.56 mmol, 2.00 eq) in 50% HCl (0.74 M, 3.1 mL) at 0 °C for 1 h to give compound **7m** as a black brown oil (555 mg, 99% yield) which was used without purification. IR (neat) 2118, 2080, 1477, 1289, 1267, 1131, 1112, and 1002 cm^{-1}; ^{1}H NMR (300 MHz, $CDCl_3$) δ 6.74 (d, 2H, J = 8.7 Hz) and 7.60 (d, 2H, J = 8.7 Hz) ppm.; ^{13}C NMR (75 MHz, $CDCl_3$) δ 139.8, 138.6 (2C), 120.9 (2C) and 88.3 ppm.

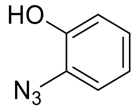

*2-azidophenol (7**n**)*. [77] Following the procedure B using 2-aminophenol (1000 mg, 9.16 mmol, 1.00 eq), sodium nitrite (1.50 M; 1.90 g, 27.49 mmol, 3.00 eq), sodium azide (1.50 M; 1.19 g, 18.32 mmol, 2.00 eq) and sodium acetate (1.50 M; 1.50 g, 18.32 mmol, 2.00 eq) in 50% HCl (0.74 M, 12.4 mL) at 0 ºC for 1 h to give compound **7n** as a black brown oil (1.24 g, 100% yield) which was used without purification. IR (neat) 3410, 2119, 2103, 1494, 1293, 1246, 1207, and 1142 cm^{-1}; ^{1}H NMR (300 MHz, $CDCl_3$) δ 5.41 (brs, 1H), 6.88 (m, 2H) and 6.99 (m, 2H) ppm.; ^{13}C NMR (75 MHz, $CDCl_3$) δ 147.3, 126.0 (2C), 121.3, 118.5 and 116.2 ppm.

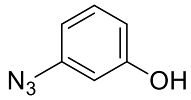

*3-azidophenol (7**o**)*. [82] Following the procedure B using 3-aminophenol (1000 mg, 9.16 mmol, 1.00 eq), sodium nitrite (1.50 M; 1.90 g, 27.49 mmol, 3.00 eq), sodium azide (1.50 M; 1.19 g, 18.32 mmol, 2.00 eq) and sodium acetate (1.50 M; 1.50 g, 18.32 mmol, 2.00 eq) in 50% HCl (0.74 M, 12.4 mL) at 0 ºC for 1 h to give compound **7o** as a black brown oil (1.24 g, 100% yield) which was used without purification. IR (neat) 3335, 1591, 1487, 1456, 1300, 1211, 1163, and 1114 cm^{-1}; ^{1}H NMR (300 MHz, $CDCl_3$) δ 5.14 (s, 1H), 6.49 (t, 1H, *J* = 2.2 Hz), 6.61 (dt, 2H, *J* = 8.1 and 2.3 Hz) and 7.18 (t, 1H, *J* = 8.1 Hz) ppm.; ^{13}C NMR (75 MHz, $CDCl_3$) δ 156.7, 141.4, 130.8, 112.2, 111.5 and 106.3 ppm.

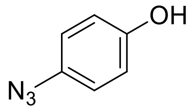

*4-azidophenol (7**p**)*. [81] Following the procedure B using 4-aminophenol (1000 mg, 9.16 mmol, 1.00 eq), sodium nitrite (1.50 M; 1.90 g, 27.49 mmol, 3.00 eq), sodium azide (1.50 M; 1.19 g, 18.32 mmol, 2.00 eq) and sodium acetate (1.50 M; 1.50 g, 18.32 mmol, 2.00 eq) in 50% HCl (0.74 M, 12.4 mL) at 0 ºC for 1 h to give compound **7p** as a black brown oil (1.24 g, 100% yield) which was used without purification. IR (neat) 3291, 2108, 1505, 1444, 1303, 1230, and 1104 cm^{-1}; ^{1}H NMR (300 MHz, DMSO-d_6) δ 6.81 (d, 2H, *J* = 9.0 Hz), 6.89 (d, 2H, *J* = 9.0 Hz) and 9.57 (brs, 1H) ppm.; ^{13}C NMR (75 MHz, DMSO-d_6) δ 155.2, 129.9, 120.2 (2C) and 116.8 (2C) ppm.

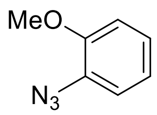

*1-azido-2-methoxybenzene (**7q**).* [82] Following the procedure B using 2-methoxyaniline (678 μL, 6.00 mmol, 1.00 eq), sodium nitrite (1.50 M; 1.24 g, 18.00 mmol, 3.00 eq), sodium azide (1.50 M; 780 mg, 12.00 mmol, 2.00 eq) and sodium acetate (1.50 M; 984 mg, 12.00 mmol, 2.00 eq) in 50% HCl (0.74 M, 8.1 mL) at 0 °C for 1 h to give compound **7q** as a black brown oil (886 mg, 99% yield) which was used without purification. IR (neat) 2942, 2839, 2110, 1494, 1456, 1300, 1242, 1101, and 1027 cm^{-1}; ^{1}H NMR (300 MHz, $CDCl_3$) δ 3.86 (s, 3H), 6.89 (ddd, 1H, *J* = 8.1, 7.8 and 1.5 Hz), 6.93 (dd, 1H, *J* = 7.2 and 1.5 Hz), 7.00 (dd, 1H, *J* = 7.8 and 1.8 Hz) and 7.09 (ddd, 1H, *J* = 8.1, 7.2 and 1.8 Hz) ppm.; ^{13}C NMR (75 MHz, $CDCl_3$) δ 151.7, 127.6, 125.1, 120.6, 119.9, 111.4 and 55.0 ppm.

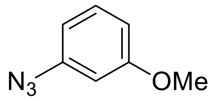

*1-azido-3-methoxybenzene (**7r**).* [82] Following the procedure B using 3-methoxyaniline (672 μL, 6.00 mmol, 1.00 eq), sodium nitrite (1.50 M; 1.24 g, 18.00 mmol, 3.00 eq), sodium azide (1.50 M; 780 mg, 12.00 mmol, 2.00 eq) and sodium acetate (1.50 M; 984 mg, 12.00 mmol, 2.00 eq) in 50% HCl (0.74 M, 8.1 mL) at 0 °C for 1 h to give compound **7r** as a black oil (886 mg, 99% yield) which was used without purification. IR (neat) 2984, 2837, 2103, 1600, 1587, 1488, 1297, 1285, 1239, 1226, 1110, and 1038 cm^{-1}; ^{1}H NMR (300 MHz, $CDCl_3$) δ 3.79 (s, 3H), 6.54 (dd, 1H, *J* = 2.4 and 2.1 Hz), 6.64 (ddd, 1H, *J* = 8.1, 2.1 and 0.9 Hz), 6.68 (ddd, 1H, *J* = 8.1, 2.4 and 0.9 Hz) and 7.25 (t, 1H, *J* = 8.1 Hz) ppm.; ^{13}C NMR (75 MHz, $CDCl_3$) δ 160.7, 141.1, 130.2, 111.0, 110.4, 104.8 and 54.9 ppm.

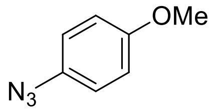

*1-azido-4-methoxybenzene (**7s**).* [82] Following the procedure B using 4-methoxyaniline (739 mg, 6.00 mmol, 1.00 eq), sodium nitrite (1.50 M; 1.24 g, 18.00 mmol, 3.00 eq), sodium azide (1.50 M; 780 mg, 12.00 mmol, 2.00 eq) and sodium acetate (1.50 M; 984 mg, 12.00 mmol, 2.00 eq) in 50% HCl (0.74 M, 8.1 mL) at 0 °C for 1 h to give compound **7s** as a black oil (877 mg, 98% yield) which was used without purification. IR (neat) 2958, 2838, 2100, 1501, 1284, 1240, 1181, 1180 and 1032 cm^{-1}; ^{1}H NMR (300 MHz, $CDCl_3$) δ 3.79 (s, 3H), 6.88 (d, 2H, *J* =

9.3 Hz) and 6.95 (d, 2H, J = 9.0 Hz) ppm.; ^{13}C NMR (75 MHz, $CDCl_3$) δ 156.9, 132.0, 119.7 (2C), 114.8 (2C) and 55.0 ppm.

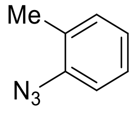

1-azido-2-methylbenzene (7t). [77] Following the procedure B using *o*-toluidine (643 µL, 6.00 mmol, 1.00 eq), sodium nitrite (1.50 M; 1.24 g, 18.00 mmol, 3.00 eq), sodium azide (1.50 M; 780 mg, 12.00 mmol, 2.00 eq) and sodium acetate (1.50 M; 984 mg, 12.00 mmol, 2.00 eq) in 50% HCl (0.74 M, 8.1 mL) at 0 °C for 1 h to give compound **7t** as a black oil (799 mg, 100% yield) which was used without purification. IR (neat) 2925, 2123, 2103, 1489, 1283, and 1099 cm^{-1}; ^{1}H NMR (300 MHz, $CDCl_3$) δ 2.18 (s, 3H), 7.00 (td, 1H, J = 7.2 and 1.2 Hz), 7.06 (brd, 1H, J = 7.8 Hz), 7.12 (brd, 1H, J = 7.2 Hz) and 7.19 (td, 1H, J = 8.1 and 1.2 Hz) ppm.; ^{13}C NMR (75 MHz, $CDCl_3$) δ 138.4, 131.1, 129.6, 127.1, 124.6, 117.9 and 17.2 ppm.

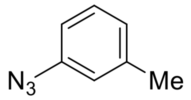

*1-azido-3-methylbenzene (7**u**).* [79] Following the procedure B using *m*-toluidine (650 µL, 6.00 mmol, 1.00 eq), sodium nitrite (1.50 M; 1.24 g, 18.00 mmol, 3.00 eq), sodium azide (1.50 M; 780 mg, 12.00 mmol, 2.00 eq) and sodium acetate (1.50 M; 984 mg, 12.00 mmol, 2.00 eq) in 50% HCl (0.74 M, 8.1 mL) at 0 °C for 1 h to give compound **7u** as a black oil (799 mg, 100% yield) which was used without purification. IR (neat) 2919, 2850, 2109, 1593, 1571, 1424, 1299, 1116, and 1024 cm^{-1}; ^{1}H NMR (300 MHz, $CDCl_3$) δ 2.34 (s, 3H), 6.80-6.85 (m, 2H), 6.94 (brd, 1H, J = 7.8 Hz) and 7.22 (dd, 1H, J = 8.7 and 7.8 Hz) ppm.; ^{13}C NMR (75 MHz, $CDCl_3$) δ 139.9, 138.8, 129.5, 125.8, 119.6, 116.1 and 21.3 ppm.

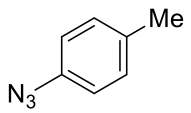

*1-azido-4-methylbenzene (7**v**).* [74] Following the procedure B using *p*-toluidine (643 mg, 6.00 mmol, 1.00 eq), sodium nitrite (1.50 M; 1.24 g, 18.00 mmol, 3.00 eq), sodium azide (1.50 M; 780 mg, 12.00 mmol, 2.00 eq) and sodium acetate (1.50 M; 984 mg, 12.00 mmol, 2.00 eq) in 50% HCl (0.74 M, 8.1 mL) at 0 °C for 1 h to give compound **7v** as a brown oil (799 mg, 100% yield) which was used without purification. IR (neat) 3031, 2926, 2139, 2099, 2052, 1505, 1295, 1281, 1129, and 1117 cm^{-1}; ^{1}H NMR (300 MHz, $CDCl_3$) δ 2.30 (s, 3H), 6.89 (d, 2H, J

= 8.4 Hz) and 7.12 (d, 2H, *J* = 8.6 Hz) ppm.; ^{13}C NMR (75 MHz, $CDCl_3$) δ 137.2, 134.6, 130.3 (2C), 118.9 (2C) and 20.8 ppm.

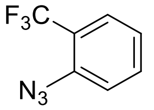

*1- azido- 2- (trifluoromethyl) benzene (7**w**).* [83] Following the procedure B using 2-(trifluoromethyl)aniline (780 µL, 6.21 mmol, 1.00 eq), sodium nitrite (1.50 M; 1.28 g, 18.62 mmol, 3.00 eq), sodium azide (1.50 M; 807 mg, 12.42 mmol, 2.00 eq) and sodium acetate (1.50 M; 1.02 g, 12.42 mmol, 2.00 eq) in 50% HCl (0.74 M, 8.4 mL) at 0 °C for 1 h to give compound **7w** as a yellow oil (1.16 g, 100% yield) which was used without purification. IR (neat) 2126, 2099, 1493, 1313, 1292, 1291, 1113, and 1039 cm^{-1}; ^{1}H NMR (300 MHz, DMSO-d_6) (ppm): 7.35 (t, 1H, *J* = 7.8 Hz), 7.55 (d, 1H, *J* = 8.0 Hz), 7.71 (d, 1H, *J* = 7.9 Hz) and 7.73 (t, 1H, *J* = 7.7 Hz) ppm.; ^{13}C NMR (75 MHz, DMSO-d_6) δ 137.9 (q, *J* = 0.8 Hz), 134.1, 127.1 (q, *J* = 5.3 Hz), 125.0, 123.1 (q, *J* = 270.8 Hz), 120.6 and 119.3 (q, *J* = 30.8 Hz) ppm.; ^{19}F NMR (282 MHz, DMSO-d_6) δ -62.04 (s, 3F) ppm.

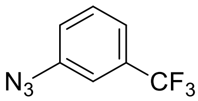

*1- azido- 3- (trifluoromethyl) benzene (7**x**)* . [79] Following the procedure B using 3-(trifluoromethyl)aniline (775 µL, 6.21 mmol, 1.00 eq), sodium nitrite (1.50 M; 1.28 g, 18.62 mmol, 3.00 eq), sodium azide (1.50 M; 807 mg, 12.42 mmol, 2.00 eq) and sodium acetate (1.50 M; 1.02 g, 12.42 mmol, 2.00 eq) in 50% HCl (0.74 M, 8.4 mL) at 0 °C for 1 h to give compound **7x** as a yellow oil (1.16 g, 100% yield) which was used without purification. IR (neat) 2106, 1453, 1323, 1289, 1278, 1169, 1124, 1109, and 1065 cm^{-1}; ^{1}H NMR (300 MHz, DMSO-d_6) (ppm): 7.35 (brs, 1H), 7.43 (ddd, 1H, *J* = 8.0, 1.3 and 0.6 Hz), 7.51 (dt, 1H, *J* = 7.8 and 0.7 Hz) and 7.63 (t, 1H, *J* = 7.9 Hz) ppm.; ^{13}C NMR (75 MHz, DMSO-d_6) δ 140.7, 131.1, 130.7 (q, *J* = 32.3 Hz), 123.6 (q, *J* = 270.8 Hz), 123.1, 121.5 (q, *J* = 4.5 Hz) and 115.8 (q, *J* = 3.8 Hz) ppm.; ^{19}F NMR (282 MHz, DMSO-d_6) δ -62.36 (s, 3F) ppm.

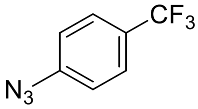

*1- azido- 4- (trifluoromethyl) benzene (7**y**)* . [77] Following the procedure B using 4-(trifluoromethyl)aniline (1.00 g, 6.21 mmol, 1.00 eq), sodium nitrite (1.50 M; 1.28 g, 18.62

mmol, 3.00 eq), sodium azide (1.50 M; 807 mg, 12.42 mmol, 2.00 eq) and sodium acetate (1.50 M; 1.02 g, 12.42 mmol, 2.00 eq) in 50% HCl (0.74 M, 8.4 mL) at 0 °C for 1 h to give compound **7y** as a yellow oil (1.16 g, 100% yield) which was used without purification. IR (neat) 2125, 2100, 1614, 1325, 1318, 1284, 1164, 1120, 1108, 1063, and 1015 cm^{-1}; ^{1}H NMR (300 MHz, DMSO-d_6) (ppm): 7.29 (d, 2H, J = 8.4 Hz) and 7.72 (d, 2H, J = 8.4 Hz) ppm.; ^{13}C NMR (75 MHz, DMSO-d_6) δ 143.7, 127.0 (2C, q, J = 3.8 Hz0, 125.4 (q, J = 32.3 Hz), 124.1 (q, J = 270.0 Hz) and 119.8 (2C) ppm.; ^{19}F NMR (282 MHz, DMSO-d_6) δ -62.35 (s, 3F) ppm.

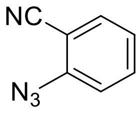

2-azidobenzonitrile (7z). [77] Following the procedure B using 2-aminobenzonitrile (709 mg, 6.00 mmol, 1.00 eq), sodium nitrite (1.50 M; 1.24 g, 18.00 mmol, 3.00 eq), sodium azide (1.50 M; 780 mg, 12.00 mmol, 2.00 eq) and sodium acetate (1.50 M; 984 mg, 12.00 mmol, 2.00 eq) in 50% HCl (0.74 M, 8.1 mL) at 0 °C for 1 h to give compound **7z** as a brown solid (865 mg, 100% yield) which was used without purification. mp = 47-48 °C; IR (neat) 2225, 2140, 2113, 1593, 1575, 1488, 1446, 1308, 1305, 1292, and 1167 cm^{-1}; ^{1}H NMR (300 MHz, $CDCl_3$) δ 7.23 (td, 1H, J = 7.8 and 0.9 Hz), 7.27 (dd, 1H, J = 8.7 and 0.9 Hz), 7.61 (dd, 1H, J = 7.5 and 1.2 Hz) and 7.63 (td, 1H, J = 7.5 and 1.5 Hz) ppm.; ^{13}C NMR (75 MHz, $CDCl_3$) δ 143.3, 134.2, 133.9, 125.1, 118.9, 115.6 and 104.0 ppm.

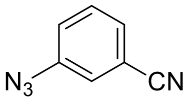

3-azidobenzonitrile (7a'). [79] Following the procedure B using 3-aminobenzonitrile (709 mg, 6.00 mmol, 1.00 eq), sodium nitrite (1.50 M; 1.24 g, 18.00 mmol, 3.00 eq), sodium azide (1.50 M; 780 mg, 12.00 mmol, 2.00 eq) and sodium acetate (1.50 M; 984 mg, 12.00 mmol, 2.00 eq) in 50% HCl (0.74 M, 8.1 mL) at 0 °C for 1 h to give compound **7a'** as a brown solid (822 mg, 95% yield) which was used without purification. mp = 52-53 °C; IR (neat) 2230, 2116, 1599, 1577, 1486, 1433, 1301, 1277, and 1112 cm^{-1}; ^{1}H NMR (300 MHz, $CDCl_3$) δ 7.24-7.29 (m, 2H), 7.43 (dt, 1H, J = 7.5 and 1.5 Hz) and 7.48 (td, 1H, J = 7.5 and 1.5 Hz) ppm.; ^{13}C NMR (75 MHz, $CDCl_3$) δ 141.5, 130.7, 128.4, 123.4, 122.4, 117.9 and 113.9 ppm.

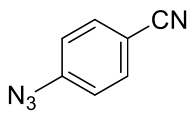

*4-azidobenzonitrile (7**b’**).* [84] Following the procedure B using 4-aminobenzonitrile (709 mg, 6.00 mmol, 1.00 eq), sodium nitrite (1.50 M; 1.24 g, 18.00 mmol, 3.00 eq), sodium azide (1.50 M; 780 mg, 12.00 mmol, 2.00 eq) and sodium acetate (1.50 M; 984 mg, 12.00 mmol, 2.00 eq) in 50% HCl (0.74 M, 8.1 mL) at 0 ºC for 1 h to give compound **7b’** as a brown solid (856 mg, 99% yield) which was used without purification. mp = 58-59 ºC; IR (neat) 2221, 2154, 2112, 1597, 1505, 1417, 1309, 1281, 1176, and 1126 cm^{-1}; ^{1}H NMR (300 MHz, $CDCl_3$) δ 7.11 (d, 2H, J = 9.0 Hz) and 7.65 (d, 2H, J = 9.0 Hz) ppm.; ^{13}C NMR (75 MHz, $CDCl_3$) δ 144.9, 133.9 (2C), 119.8 (2C), 118.4 and 108.3 ppm.

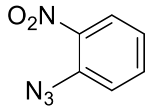

*1-azido-2-nitrobenzene (7**c’**).* [74] Following the procedure B using 2-nitroaniline (829 mg, 6.00 mmol, 1.00 eq), sodium nitrite (1.50 M; 1.24 g, 18.00 mmol, 3.00 eq), sodium azide (1.50 M; 780 mg, 12.00 mmol, 2.00 eq) and sodium acetate (1.50 M; 984 mg, 12.00 mmol, 2.00 eq) in 50% HCl (0.74 M, 8.1 mL) at 0 ºC for 1 h to give compound **7c’** as a light brown solid (975 mg, 99% yield) which was used without purification. mp = 40-41 ºC; IR (neat) 2120, 2093, 1603, 1580, 1520, 1479, 1342, 1291, and 1166 cm^{-1}; ^{1}H NMR (300 MHz, $CDCl_3$) δ 7.27 (ddd, 1H, J = 8.4, 7.5 and 1.2 Hz), 7.35 (dd, 1H, J = 8.4 and 1.2 Hz), 7.64 (ddd, 1H, J = 8.1, 7.5 and 1.5 Hz) and 7.93 (dd, 1H, J = 8.1 and 1.5 Hz) ppm.; ^{13}C NMR (75 MHz, $CDCl_3$) δ 140.8, 134.7, 134.2, 126.0, 125.1 and 120.9 ppm.

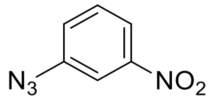

*1-azido-3-nitrobenzene (7**d’**).* [74] Following the procedure B using 3-nitroaniline (829 mg, 6.00 mmol, 1.00 eq), sodium nitrite (1.50 M; 1.24 g, 18.00 mmol, 3.00 eq), sodium azide (1.50 M; 780 mg, 12.00 mmol, 2.00 eq) and sodium acetate (1.50 M; 984 mg, 12.00 mmol, 2.00 eq) in 50% HCl (0.74 M, 8.1 mL) at 0 ºC for 1 h to give compound **7d’** as a light brown solid (975 mg, 99% yield) which was used without purification. mp = 51-52 ºC; IR (neat) 2120, 2104, 1517, 1476, 1351, 1304, 1271, and 1143 cm^{-1}; ^{1}H NMR (300 MHz, $CDCl_3$) δ 7.35 (ddd, 1H, J = 8.1, 2.1 and 0.9 Hz), 7.55 (t, 1H, J = 8.1 Hz), 7.89 (t, 1H, J = 2.1 Hz) and 8.00 (ddd, 1H, J

= 8.1, 2.1 and 0.9 Hz) ppm.; ^{13}C NMR (75 MHz, $CDCl_3$) δ 149.2, 142.0, 130.7, 125.0, 119.7 and 114.1 ppm.

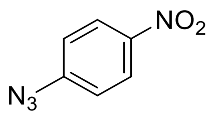

*1-azido-4-nitrobenzene (7**e'**).* [77] Following the procedure B using 4-nitroaniline (829 mg, 6.00 mmol, 1.00 eq), sodium nitrite (1.50 M; 1.24 g, 18.00 mmol, 3.00 eq), sodium azide (1.50 M; 780 mg, 12.00 mmol, 2.00 eq) and sodium acetate (1.50 M; 984 mg, 12.00 mmol, 2.00 eq) in 50% HCl (0.74 M, 8.1 mL) at 0 °C for 1 h to give compound **7e'** as a light brown solid (985 mg, 100% yield) which was used without purification. mp = 72-73 ºC; IR (neat) 2121, 2085, 1590, 1514, 1490, 1340, 1287, 1180, and 1107 cm^{-1}; ^{1}H NMR (300 MHz, $CDCl_3$) δ 7.14 (d, 2H, J = 9.3 Hz) and 8.24 (d, 2H, J = 9.3 Hz) ppm.; ^{13}C NMR (75 MHz, $CDCl_3$) δ 146.9, 144.7, 125.9 (2C) and 119.4 (2C) ppm.

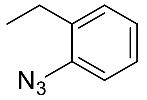

*1-azido-2-ethylbenzene (7**f'**).* [85] Following the procedure B using 2-ethylaniline (750 µL, 6.00 mmol, 1.00 eq), sodium nitrite (1.50 M; 1.24 g, 18.00 mmol, 3.00 eq), sodium azide (1.50 M; 780 mg, 12.00 mmol, 2.00 eq) and sodium acetate (1.50 M; 984 mg, 12.00 mmol, 2.00 eq) in 50% HCl (0.74 M, 8.1 mL) at 0 °C for 1 h to give the crude product which was purified by column chromatography (silica gel, pure hexane) to obtain the product **7f'** as a yellow oil (706 mg, 80% yield). IR (neat) 2971, 2120, 2118, 1582, 1489, 1452, 1284, 1151, and 1104 cm^{-1}; ^{1}H NMR (300 MHz, $CDCl_3$) δ 1.17 (t, 3H, J = 7.5 Hz), 2.59 (q, 2H, J = 7.5 Hz), 7.06 (td, 1H, J = 7.5 and 1.2 Hz), 7.11 (dd, 1H, J = 7.8 and 1.2 Hz), 7.17 (ddd, 1H, J = 7.5, 1.2 and 0.6 Hz) and 7.22 (ddd, 1H, J = 8.7, 7.8 and 1.5 Hz) ppm.; ^{13}C NMR (75 MHz, $CDCl_3$) δ 137.8, 135.7, 129.6, 127.1, 124.8, 118.0, 24.3 and 14.6 ppm.

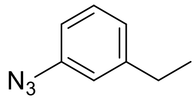

*1-azido-3-ethylbenzene (7**g'**).* [85] Following the procedure B using 3-ethylaniline (750 µL, 6.00 mmol, 1.00 eq), sodium nitrite (1.50 M; 1.24 g, 18.00 mmol, 3.00 eq), sodium azide (1.50 M; 780 mg, 12.00 mmol, 2.00 eq) and sodium acetate (1.50 M; 984 mg, 12.00 mmol, 2.00 eq) in 50% HCl (0.74 M, 8.1 mL) at 0 °C for 1 h to give compound **7g'** as a black oil (865 mg,

98% yield) which was used without purification. IR (neat) 2968, 2106, 1606, 1585, 1489, 1447, 1287, and 1203 cm^{-1}; ^{1}H NMR (300 MHz, $CDCl_3$) δ 1.22 (t, 3H, *J* = 7.6 Hz), 2.63 (q, 2H, *J* = 7.6 Hz), 6.81-6.87 (m, 2H), 6.97 (brd, 1H, *J* = 7.4 Hz) and 7.24 (dd, 1H, *J* = 7.8 and 7.6 Hz) ppm.; ^{13}C NMR (75 MHz, $CDCl_3$) δ 146.3, 140.1, 129.8, 124.7, 118.6, 116.5, 28.9 and 15.4 ppm.

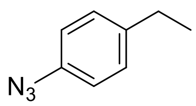

*1-azido-4-ethylbenzene (7**h'**).* [86] Following the procedure B using 4-ethylaniline (750 µL, 6.00 mmol, 1.00 eq), sodium nitrite (1.50 M; 1.24 g, 18.00 mmol, 3.00 eq), sodium azide (1.50 M; 780 mg, 12.00 mmol, 2.00 eq) and sodium acetate (1.50 M; 984 mg, 12.00 mmol, 2.00 eq) in 50% HCl (0.74 M, 8.1 mL) at 0 °C for 1 h to give compound **7h'** as a brown oil (883 mg, 100% yield) which was used without purification. IR (neat) 2968, 2133, 2094, 1506, 1288, and 1130 cm^{-1}; ^{1}H NMR (300 MHz, $CDCl_3$) δ 1.21 (t, 3H, *J* = 7.6 Hz), 2.62 (q, 2H, *J* = 7.6 Hz), 6.93 (d, 2H, *J* = 8.5 Hz) and 7.16 (d, 2H, *J* = 8.6 Hz) ppm.; ^{13}C NMR (75 MHz, $CDCl_3$) δ 141.1, 137.6, 129.3 (2C), 119.1 (2C), 28.5 and 15.7 ppm.

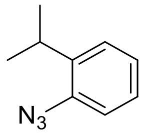

*1-azido-2-isopropylbenzene (7**i'**).* [78] Following the procedure B using 2-isopropylaniline (836 µL, 6.00 mmol, 1.00 eq), sodium nitrite (1.50 M; 1.24 g, 18.00 mmol, 3.00 eq), sodium azide (1.50 M; 780 mg, 12.00 mmol, 2.00 eq) and sodium acetate (1.50 M; 984 mg, 12.00 mmol, 2.00 eq) in 50% HCl (0.74 M, 8.1 mL) at 0 °C for 1 h to give the crude product which was purified by column chromatography (silica gel, pure hexane) to obtain the pure product **7i'** as a colorless oil (745 mg, 75% yield). IR (neat) 2964, 2122, 2118, 1488, 1446, 1291, 1077, and 1037 cm^{-1}; ^{1}H NMR (300 MHz, $CDCl_3$) δ 1.20 (d, 6H, *J* = 6.9 Hz), 3.20 (septet, 1H, *J* = 6.9 Hz), 7.10 (td, 1H, *J* = 7.2 and 1.5 Hz), 7.11 (dd, 1H, *J* = 7.2 and 1.5 Hz), 7.21 (dd, 1H, *J* = 7.2 and 1.5 Hz) and 7.22-7.27 (m, 1H) ppm.; ^{13}C NMR (75 MHz, $CDCl_3$) δ 140.1, 137.3, 127.0, 126.7, 125.0, 118.1, 28.0 and 22.9 (2C) ppm.

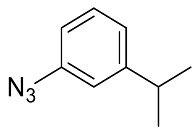

*1-azido-3-isopropylbenzene (**7j'**)*. [77] Following the procedure B using 3-isopropylaniline (845 µL, 6.00 mmol, 1.00 eq), sodium nitrite (1.50 M; 1.24 g, 18.00 mmol, 3.00 eq), sodium azide (1.50 M; 780 mg, 12.00 mmol, 2.00 eq) and sodium acetate (1.50 M; 984 mg, 12.00 mmol, 2.00 eq) in 50% HCl (0.74 M, 8.1 mL) at 0 ºC for 1 h to give compound **7j'** as a black oil (890 mg, 92% yield) which was used without purification. IR (neat) 2963, 2137, 2100, 1605, 1604, 1480, 1443, and 1290 cm^{-1}; ^{1}H NMR (300 MHz, $CDCl_3$) δ 1.24 (d, 6H, J = 6.9 Hz), 2.69 (septet, 1H, J = 6.9 Hz), 6.85 (ddd, 1H, J = 7.8, 2.4 and 0.9 Hz), 6.87 (t, 1H, J = 2.1 Hz), 7.00 (brd, 1H, J = 7.8 Hz) and 7.26 (td, 1H, J = 7.8 and 0.3 Hz) ppm.; ^{13}C NMR (75 MHz, $CDCl_3$) δ 150.8, 139.9, 129.6, 123.1, 117.1, 116.4, 34.1 and 23.7 (2C) ppm.

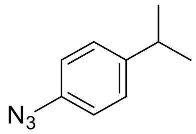

*1-azido-4-isopropylbenzene (**7k'**)*. [87] Following the procedure B using 4-isopropylaniline (854 µL, 6.00 mmol, 1.00 eq), sodium nitrite (1.50 M; 1.24 g, 18.00 mmol, 3.00 eq), sodium azide (1.50 M; 780 mg, 12.00 mmol, 2.00 eq) and sodium acetate (1.50 M; 984 mg, 12.00 mmol, 2.00 eq) in 50% HCl (0.74 M, 8.1 mL) at 0 ºC for 1 h to give compound **7k'** as a brown oil (967 mg, 100% yield) which was used without purification. IR (neat) 2962, 2130, 2090, 1606, 1506, 1292, 1285, 1130, and 1055 cm^{-1}; ^{1}H NMR (300 MHz, $CDCl_3$) δ 1.23 (d, 6H, J = 6.9 Hz), 2.88 (septet, 1H, J = 6.9 Hz), 6.95 (d, 2H, J = 8.4 Hz) and 7.20 (d, 2H, J = 8.1 Hz) ppm.; ^{13}C NMR (75 MHz, $CDCl_3$) δ 145.5, 137.5, 127.7 (2C), 118.9 (2C), 33.6, 23.9 (2C) ppm.

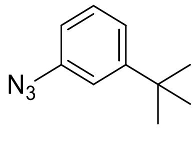

*1-azido-3-(tert-butyl)benzene (**7l'**)*. [79] Following the procedure B using 3-(*tert*-butyl)aniline (211 µL, 1.34 mmol, 1.00 eq), sodium nitrite (1.50 M; 277 mg, 4.02 mmol, 3.00 eq), sodium azide (1.50 M; 174 mg, 2.68 mmol, 2.00 eq) and sodium acetate (1.50 M; 220 mg, 2.68 mmol, 2.00 eq) in 50% HCl (0.74 M, 1.8 mL) at 0 ºC for 1 h to give compound **7l'** as a yellow oil (225 mg, 96% yield) which was used without purification. IR (neat) 2966, 2116, 2096, 1604,

1600, 1483, 1365, 1294, 1274, and 1144 cm^{-1}; ^{1}H NMR (300 MHz, $CDCl_3$) δ 1.31 (s, 9H), 6.86 (ddd, 1H, J = 7.8, 2.1 and 1.2 Hz), 7.02 (t, 1H, J = 2.1 Hz), 7.16 (ddd, 1H, J = 7.8, 1.8 and 1.2 Hz) and 7.28 (t, 1H, J = 7.8 Hz) ppm.; ^{13}C NMR (75 MHz, $CDCl_3$) δ 153.2, 139.7, 129.4, 122.1, 116.3, 116.0, 34.8 and 31.2 (3C) ppm.

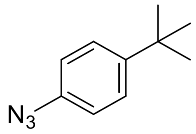

*1-azido-4-(tert-butyl)benzene (***7m'***)*. [77] Following the procedure B using 4-(*tert*-butyl)aniline (211 μL, 1.34 mmol, 1.00 eq), sodium nitrite (1.50 M; 277 mg, 4.02 mmol, 3.00 eq), sodium azide (1.50 M; 174 mg, 2.68 mmol, 2.00 eq) and sodium acetate (1.50 M; 220 mg, 2.68 mmol, 2.00 eq) in 50% HCl (0.74 M, 1.8 mL) at 0 ºC for 1 h to give compound **7m'** as a black brown oil (232 mg, 99% yield) which was used without purification. IR (neat) 2964, 2121, 2086, 1508, 1364, 1295, 1282, and 1137 cm^{-1}; ^{1}H NMR (300 MHz, $CDCl_3$) δ 1.29 (s, 9H), 6.94 (d, 2H, J = 9.0 Hz) and 7.34 (d, 2H, J = 8.7 Hz) ppm.; ^{13}C NMR (75 MHz, $CDCl_3$) δ 148.0, 137.1, 126.7 (2C), 118.6 (2C), 34.4 and 31.3 (3C) ppm.

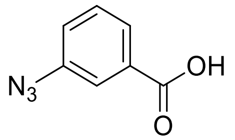

*3-azidobenzoic acid (***7n'***)*. [77] Following the procedure B using 3-aminobenzoic acid (823 mg, 6.00 mmol, 1.00 eq), sodium nitrite (1.50 M; 1.24 g, 18.00 mmol, 3.00 eq), sodium azide (1.50 M; 780 mg, 12.00 mmol, 2.00 eq) and sodium acetate (1.50 M; 984 mg, 12.00 mmol, 2.00 eq) in 50% HCl (0.74 M, 8.1 mL) at 0 ºC for 1 h to give compound **7n'** as a light-yellow solid (969 mg, 99% yield) which was used without purification. mp = 147-148 ºC; IR (neat) 2551, 2116, 1680, 1580, 1452, 1417, 1302, 1262, and 1154 cm^{-1}; ^{1}H NMR (300 MHz, DMSO-d_6) δ 7.36 (ddd, 1H, J = 8.1, 2.4 and 0.9 Hz), 7.53 (dd, 1H, J = 8.1 and 7.8 Hz), 7.55-7.58 (m, 1H) and 7.75 (dt, 1H, J = 7.8 and 1.2 Hz) ppm.; ^{13}C NMR (75 MHz, DMSO-d_6) δ 166.5, 139.9, 132.6, 130.3, 125.9, 123.5 and 119.4 ppm.

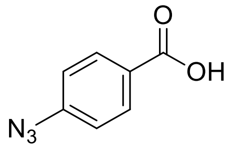

*4-azidobenzoic acid (7**o'**)*. [77] Following the procedure B using 4-aminobenzoic acid (823 mg, 6.00 mmol, 1.00 eq), sodium nitrite (1.50 M; 1.24 g, 18.00 mmol, 3.00 eq), sodium azide (1.50 M; 780 mg, 12.00 mmol, 2.00 eq) and sodium acetate (1.50 M; 984 mg, 12.00 mmol, 2.00 eq) in 50% HCl (0.74 M, 8.1 mL) at 0 ºC for 1 h to give compound **7o'** as a brown solid (979 mg, 100% yield) which was used without purification. mp = 177-178 ºC; IR (neat) 2544, 2101, 1668, 1600, 1507, 1424, 1317, 1283, 1177, and 1121 cm^{-1}; ^{1}H NMR (300 MHz, MeOD-d_4) δ 7.14 (d, 2H, J = 8.8 Hz) and 8.03 (d, 2H, J = 8.8 Hz) ppm.; ^{13}C NMR (75 MHz, MeOD-d_4) δ 169.2, 146.4, 132.8 (2C), 128.7 and 120.1 (2C) ppm.

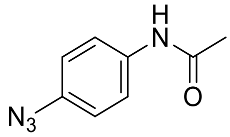

*N-(4-azidophenyl)acetamide (7**u'**)*. [88] Following the procedure B using 4'-aminoacetanilide (100 mg, 0.67 mmol, 1.00 eq), sodium nitrite (1.50 M; 139 mg, 2.01 mmol, 3.00 eq), sodium azide (1.50 M; 87 mg, 1.34 mmol, 2.00 eq) and sodium acetate (1.50 M; 110 mg, 1.34 mmol, 2.00 eq) in 50% HCl (0.74 M, 0.9 mL) at 0 ºC for 1 h to give compound **7u'** as a brown solid (117 mg, 99% yield) which was used without purification. mp = 115-116 ºC; IR (neat) 3255, 3073, 2112, 2079, 1665, 1605, 1551, 1504, 1407, 1291, and 1284 cm^{-1}; ^{1}H NMR (300 MHz, DMSO-d_6) δ 2.03 (s, 3H), 7.05 (d, 2H, J = 8.9 Hz), 7.62 (d, 2H, J = 8.9 Hz) and 10.01 (brs, 1H) ppm.; ^{13}C NMR (75 MHz, DMSO-d_6) δ 168.3, 136.7, 133.6, 120.5 (2C), 119.4 (2C) and 23.9 ppm.

2. Biological activities toward A549, HuCCA-1, MOLT-3, and HL-60 cancer cell lines

The investigation of the biological activity of triazole derivatives in HepG2 cancer cells, we found the active compounds by screening of cytotoxicity at a concentration of 25 μM. It was obtained that the %cell viability and chose the target compounds with a cut off less than 55% (%inhibition at 45%) to investigate the IC_{50} values. However, the cytotoxicity toward A549, HuCCA-1, MOLT-3, and HL-60 cancer cell lines was displayed as the IC_{50} values as shown in **Table S1**.

Table S1 The cytotoxicity of triazole-cored derivatives toward various human lung carcinoma cell lines A549, Thai human cholangiocarcinoma cells HuCCA-1, *T*-cell acute lymphoblastic leukemia MOLT-3, and acute promyelocytic leukemia HL-60. The inhibitory activities were indicated as IC_{50} in micromolar scale (μM). The calculated selectivity indices (SI) were reported in square brackets.

Compound	R	Cancer Cell lines, IC_{50} (μM), [SI]			
		A549	HuCCA-1	MOLT-3	HL-60
***Doxorubicin**	-	0.47±0.02 [4.81]	1.21±0.04 [1.87]	0.01±0.00 [226]	0.12±0.02 [18.83]
****Sorafenib**	-	19.65±0.01 [1.00]	33.19±1.45 [0.59]	ND	5.35±0.38 [3.69]
**2a**	H	>100	>100	>100	>100
**2b**	*o*-F	77.34±1.76 [0.95]	69.15±19.17 [1.06]	25.28±1.38 [2.91]	40.65±4.64 [1.81]
**2c**	*m*-F	69.39±2.44 [0.89]	44.91±3.84 [1.37]	18.37±0.36 [3.35]	34.10±11.31 [1.81]
**2d**	*p*-F	57.38±8.36 [1.03]	55.35±6.49 [1.07]	20.54±1.23 [2.89]	35.02±9.04 [1.69]
**2e**	*o*-Cl	ND	ND	ND	52.09±13.88 [1.18]
**2f**	*m*-Cl	ND	ND	ND	37.51±2.23 [1.70]
**2g**	*p*-Cl	82.98±2.70 [0.18]	33.65±1.83 [0.43]	>100	>100
**2h**	*o*-Br	ND	ND	ND	37.71±3.88 [1.40]

**2i**	*m*-Br	ND	ND	ND	35.75±0.34 [1.48]
**2j**	*p*-Br	33.83±2.29 [1.79]	10.85±0.36 [5.59]	53.38±24.33 [1.14]	76.05±22.64 [0.80]
**2k**	*o*-I	ND	ND	ND	37.44±2.36 [1.31]
**2l**	*m*-I	ND	ND	ND	33.24±0.58 [1.48]
**2m**	*p*-I	ND	ND	29.92±1.51 [1.67]	49.76±7.19 [1.00]
**2n**	*o*-OH	ND	ND	39.42±3.52 [>3.08]	51.12±1.77 [>2.38]
**2o**	*m*-OH	ND	ND	28.29±4.51 [>4.29]	39.22±2.72 [>3.10]
**2p**	*p*-OH	85.85±7.24 [0.81]	87.43±0.79 [0.80]	22.51±1.85 [3.09]	32.93±7.77 [2.11]
**2q**	*o*-OMe	ND	ND	ND	42.39±2.18 [1.60]
**2r**	*m*-OMe	ND	ND	ND	42.81±1.95 [1.78]
**2s**	*p*-OMe	ND	ND	ND	>100
**2t**	*o*-Me	ND	ND	ND	45.00±4.03 [1.37]
**2u**	*m*-Me	ND	ND	ND	>100
**2v**	*p*-Me	ND	ND	91.12±1.66 [1.10]	>100
**2w**	*o*-CF_3	ND	ND	30.64±0.80 [1.92]	34.50±0.73 [1.70]
**2x**	*m*-CF_3	ND	ND	27.77±4.56 [2.28]	28.79±7.46 [2.19]
**2y**	*p*-CF_3	ND	ND	18.13±0.45 [3.23]	19.41±0.50 [3.02]
**2z**	*o*-CN	ND	ND	ND	56.37±10.91 [1.06]
**2a’**	*m*-CN	ND	ND	ND	33.84±7.18 [2.93]
**2b’**	*p*-CN	80.14±9.24 [0.80]	74.13±1.88 [0.86]	17.56±0.59 [3.65]	32.84±9.55 [1.95]

**2c'**	*o*-NO_2	ND	ND	ND	46.03±5.51 [1.32]
**2d'**	*m*-NO_2	ND	ND	ND	37.39±1.95 [1.56]
**2e'**	*p*-NO_2	63.88±4.59 [1.03]	42.24±2.75 [1.56]	14.75±0.43 [4.48]	22.52±3.39 [2.93]
**2f'**	*o*-Ethyl	ND	ND	ND	36.31±1.51 [1.59]
**2g'**	*m*-Ethyl	ND	ND	ND	40.75±0.61 [2.32]
**2h'**	*p*-Ethyl	ND	ND	ND	>100
**2i'**	*o*-isopropyl	ND	ND	ND	17.77±1.62 [3.07]
**2j'**	*m*-isopropyl	ND	ND	ND	29.28±7.33 [2.50]
**2k'**	*p*-isopropyl	ND	ND	ND	>100
**2l'**	*m*-*t*ert-Butyl	ND	ND	ND	21.55±2.68 [3.17]
**2m'**	*p*-*t*ert-Butyl	39.61±0.12 [2.06]	35.08±12.67 [2.33]	11.18±1.15 [7.30]	18.04±2.20 [4.52]
**2n'**	*m*-CO_2H	ND	ND	ND	>100
**2o'**	*p*-CO_2H	>100	>100	76.50±2.70 [>1.49]	>100
**2p'**	*o*-NH_2	ND	ND	ND	48.61±9.18 [>2.50]
**2q'**	*m*-NH_2	ND	ND	ND	42.67±3.41 [1.46]
**2r'**	*p*-NH_2	ND	ND	33.89±1.48 [>3.59]	42.07±0.68 [>2.89]
**2s'**	*o*-NHAc	ND	ND	ND	34.98±0.99 [1.53]
**2t'**	*m*- NHAc	94.17±6.18 [>1.17]	92.69±3.11 [>1.19]	25.20±1.33 [>4.38]	35.38±9.28 [>3.12]
**2u'**	*p*- NHAc	>100	11.28±4.95 [>9.79]	34.63±9.23 [>3.19]	75.84±23.92 [>1.46]
**2v'**	*m*-CONHMe	ND	ND	ND	42.11±2.61 [>2.62]

**2w'**	*p*-CONHMe	ND	ND	ND	36.46±1.57 [1.71]

*Doxorubicin = Doxorubicin hydrochloride; **Sorafenib = Sorafenib tosylate; ND = Not Determined; Selectivity Index (SI) = (IC_{50} for MRC-5)/(IC_{50} for each cancer cell); [blue color] indicates SI value ≥ 3.00, highly cancer-selective.

3. Reference

74. P. Iniyavan, G. L. Balaji, S. Sarveswari, V. Vijayakumar, CuO nanoparticles: synthesis and application as an efficient reusable catalyst for the preparation of xanthene substituted 1, 2, 3-triazoles via click chemistry, Tetrahedron Lett. 56(35) (2015) 5002-5009, https://doi.org/10.1016/j.tetlet.2015.07.016.
75. I. Birkenfelder, J. Gurke, L. Grubert, S. Hecht, B. M. Schmidt, Click Chemistry Derived Pyridazines: Electron-Deficient Building Blocks with Defined Conformation and Packing Structure, Chem Asian J. 12(24) (2017) 3156-3161, https://doi.org/10.1002/asia.201701277.
76. M. Liu, Y. Hou, W. Yin, S. Zhou, P. Qian, Z. Guo, L. Zu, Y. Zhao, Discovery of a novel 6, 7-disubstituted-4-(2-fluorophenoxy) quinolines bearing 1, 2, 3-triazole-4-carboxamide moiety as potent c-Met kinase inhibitors, Eur. J. Med. Chem. 119 (2016) 96-108, https://doi.org/10.1016/j.ejmech.2016.04.035.
77. J. Cui, L. A. Hu, W. Shi, G. Cui, X. Zhang, Q. W. Zhang, Design, synthesis and anti-platelet aggregation activity study of ginkgolide-1, 2, 3-triazole derivatives, Molecules 24(11) (2019) 2156, https://doi.org/10.3390/molecules24112156.
78. S. W. Kwok, J. R. Fotsing, R. J. Fraser, V. O. Rodionov, V. V. Fokin, Transition-metal-free catalytic synthesis of 1, 5-diaryl-1, 2, 3-triazoles, Org. Lett. 12(19) (2010) 4217-4219, https://doi.org/10.1021/ol101568d.
79. H. C. Bertrand, M. Schaap, L. Baird, N. D. Georgakopoulos, A. Fowkes, C. Thiollier, H. Kachi, A. T. Dinkova-Kostova, G. Wells, Design, synthesis, and evaluation of triazole derivatives that induce Nrf2 dependent gene products and inhibit the Keap1–Nrf2 protein–protein interaction, J. Med. Chem. 58(18) (2015) 7186-7194, https://doi.org/10.1021/acs.jmedchem.5b00602.
80. Z. Zhang, F. Xiao, B. Huang, J. Hu, B. Fu, Z. Zhang, Cyclization of alkyne–azide with isonitrile/CO via self-relay rhodium catalysis, Org. Lett. 18(5) (2016) 908-911, https://doi.org/10.1021/acs.orglett.5b03570.
81. L. Ren, N. Jiao, PdCl 2 catalyzed efficient assembly of organic azides, CO, and alcohols under mild conditions: a direct approach to synthesize carbamates, ChemComm 50(28) (2014) 3706-3709, https://doi.org/10.1039/C4CC00538D.
82. M. Kurumi, K. Sasaki, H. Takata, T. Nakayama, Synthesis and chemiluminescent activity of pyridazino [4, 5-b] indole-1, 4 (2H, 3H)-diones, Heterocycles 53(12) (2000) 2809-2819, https://doi.org/10.3987/COM-00-9049.
83. S. Zhou, H. Liao, M. Liu, G. Feng, B. Fu, R. Li, M. Cheng, Y. Zhao, P. Gong, Discovery andw biological evaluation of novel 6, 7-disubstituted-4-(2-fluorophenoxy) quinoline derivatives possessing 1, 2, 3-triazole-4-carboxamide moiety as c-Met kinase inhibitors, Bioorg. Med. Chem. 22(22) (2014) 6438-6452, https://doi.org/10.1016/j.bmc.2014.09.037.
84. K. V. Kutonova, M. E. Trusova, P. S. Postnikov, V. D. Filimonov, J. Parello, A simple and effective synthesis of aryl azides via arenediazonium tosylates, Synthesis 45(19) (2013) 2706-2710, https://doi.org/10.1055/s-0033-1339648.

85. D. Kanabar, P. Farrales, A. Kabir, D. Juang, M. Gnanmony, J. Almasri, N. Torrents, S. Shukla, V. Gupta, V. V. Dukhande, A. D'Souza, A. Muth, Optimizing the aryl-triazole of cjoc42 for enhanced gankyrin binding and anti-cancer activity, Bioorg. Med. Chem. Lett. 30(17) (2020) 127372, https://doi.org/10.1016/j.bmcl.2020.127372.
86. M. Hu, J. Li, S. Q. Yao, In situ "click" assembly of small molecule matrix metalloprotease inhibitors containing zinc-chelating groups, Org. Lett. 10(24) (2008) 5529-5531, https://doi.org/10.1021/ol802286g.
87. F. Sebest, L. Casarrubios, H. S. Rzepa, A. J. White, S. Diez-Gonzalez, Thermal azide–alkene cycloaddition reactions: straightforward multi-gram access to Δ 2-1, 2, 3-triazolines in deep eutectic solvents, Green Chem. 20(17) (2018) 4023-4035, https://doi.org/10.1039/C8GC01797B.
88. N. T. Pokhodylo, V. S. Matiichuk, N. D. Obushak, Synthesis and transformations of 1-(azidophenyl)-1H-tetrazoles. Russ. J. Org. Chem. 46(4) (2010) 556-560, https://doi.org/10.1134/S1070428010040196.

4. NMR Spectra

^{1}H NMR of compound **5** (300 MHz, Acetone-*d*$_6$)

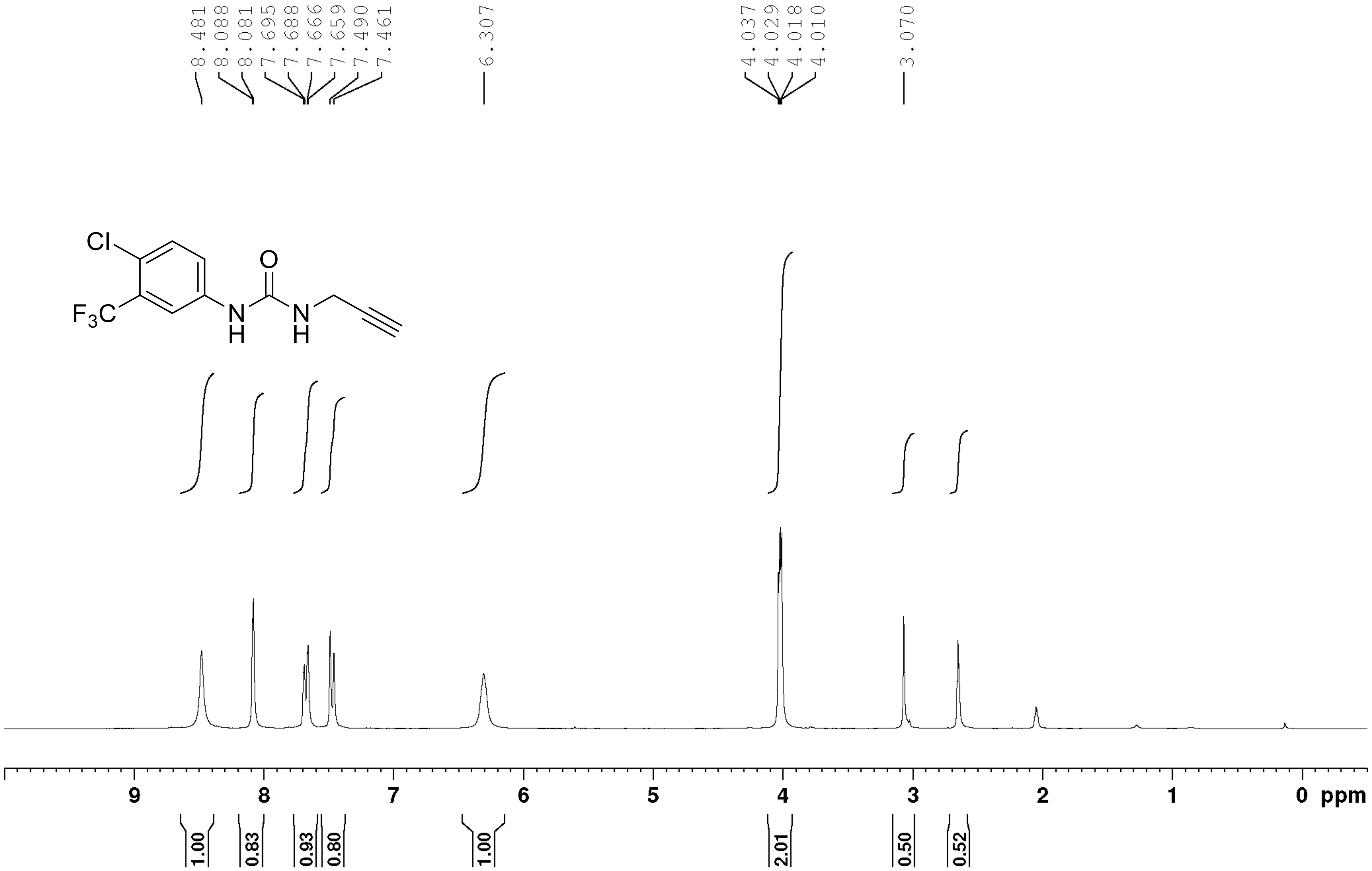

^{13}C NMR of compound **5** (75 MHz, Acetone-d_6)

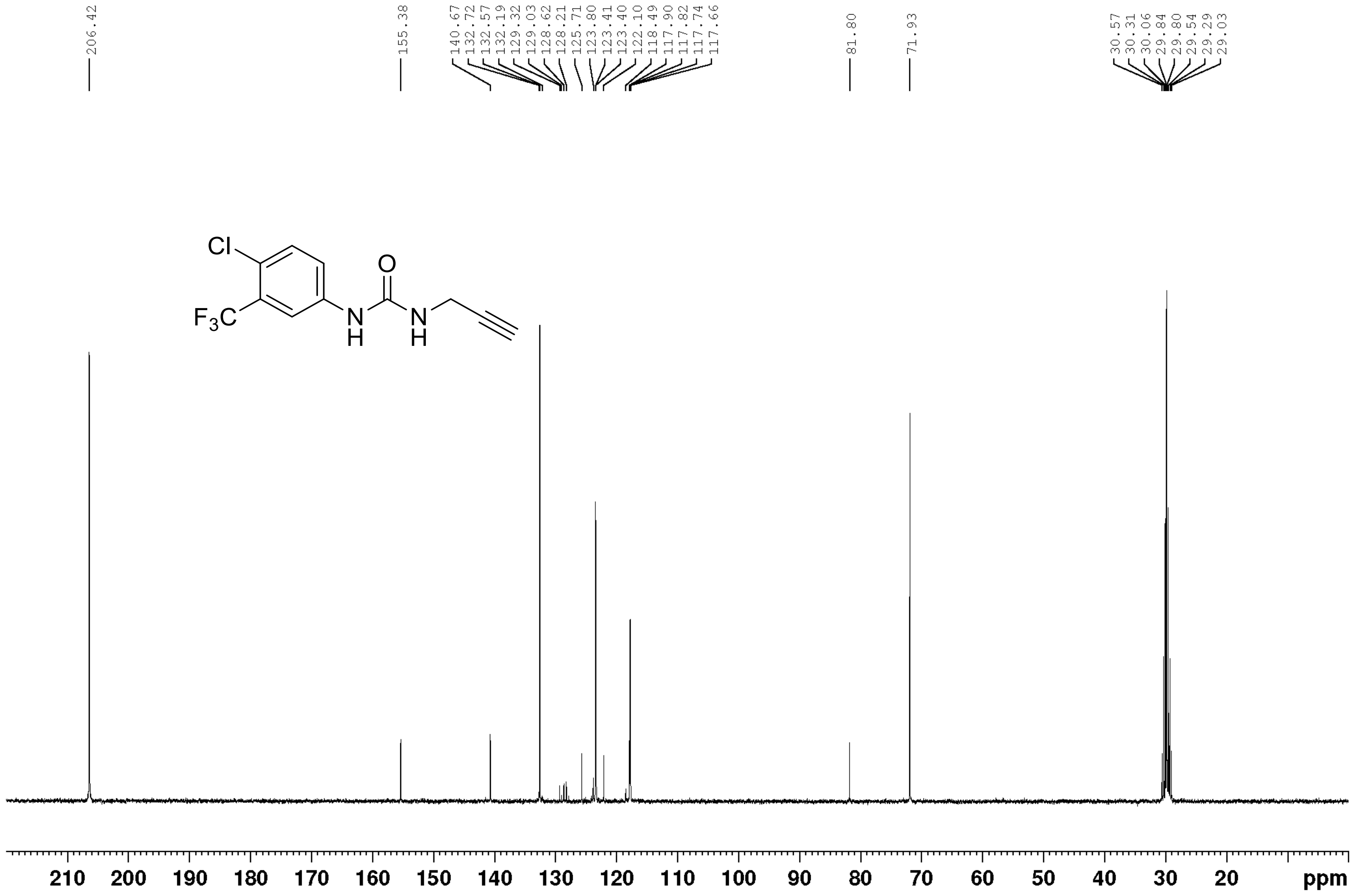

^{19}F NMR of compound **5** (282 MHz, $CDCl_3$)

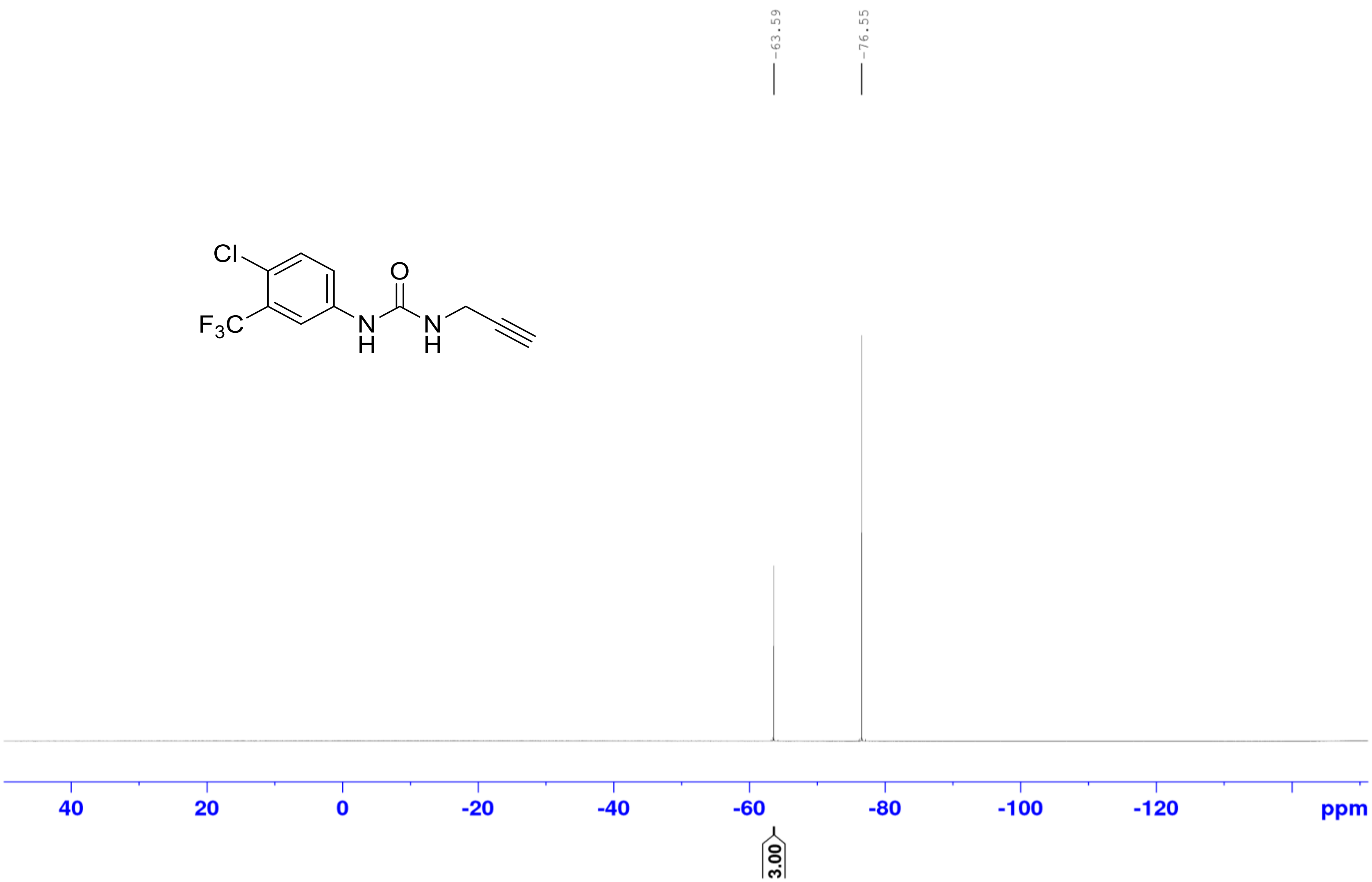

^{1}H NMR of compound **7a** (300 MHz, $CDCl_3$)

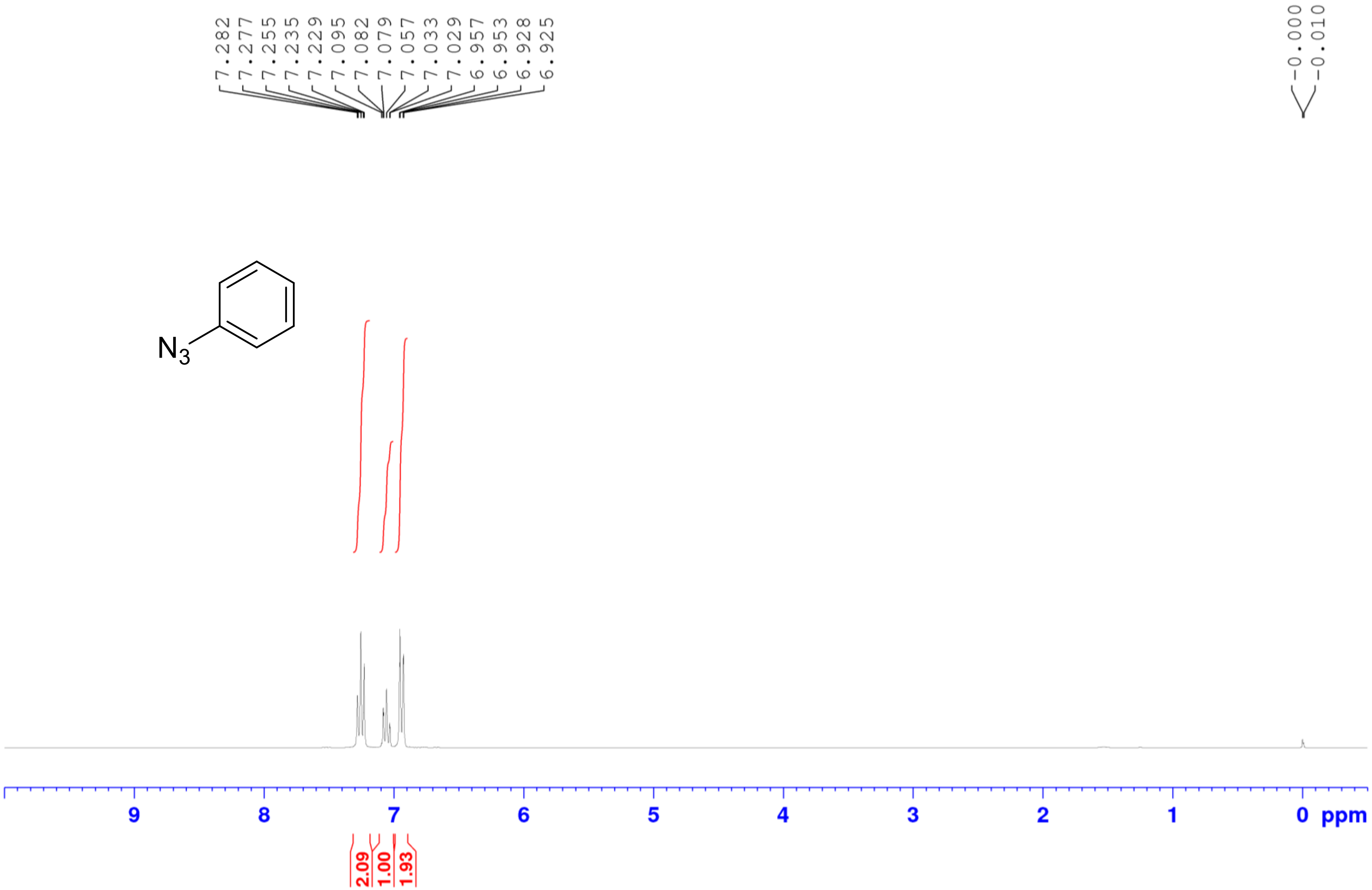

^{13}C NMR of compound **7a** (75 MHz, $CDCl_3$)

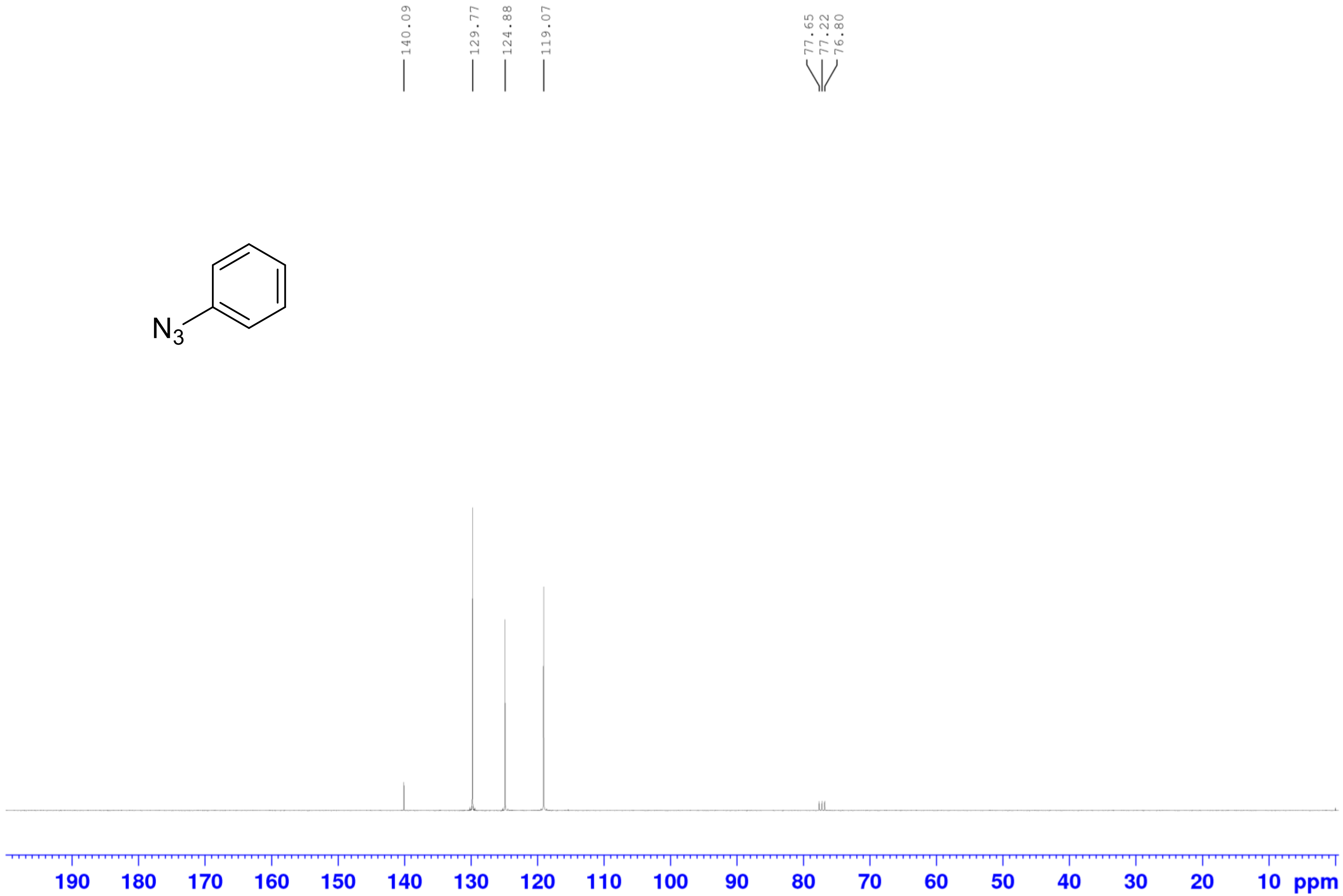

^{1}H NMR of compound **7b** (300 MHz, $CDCl_3$)

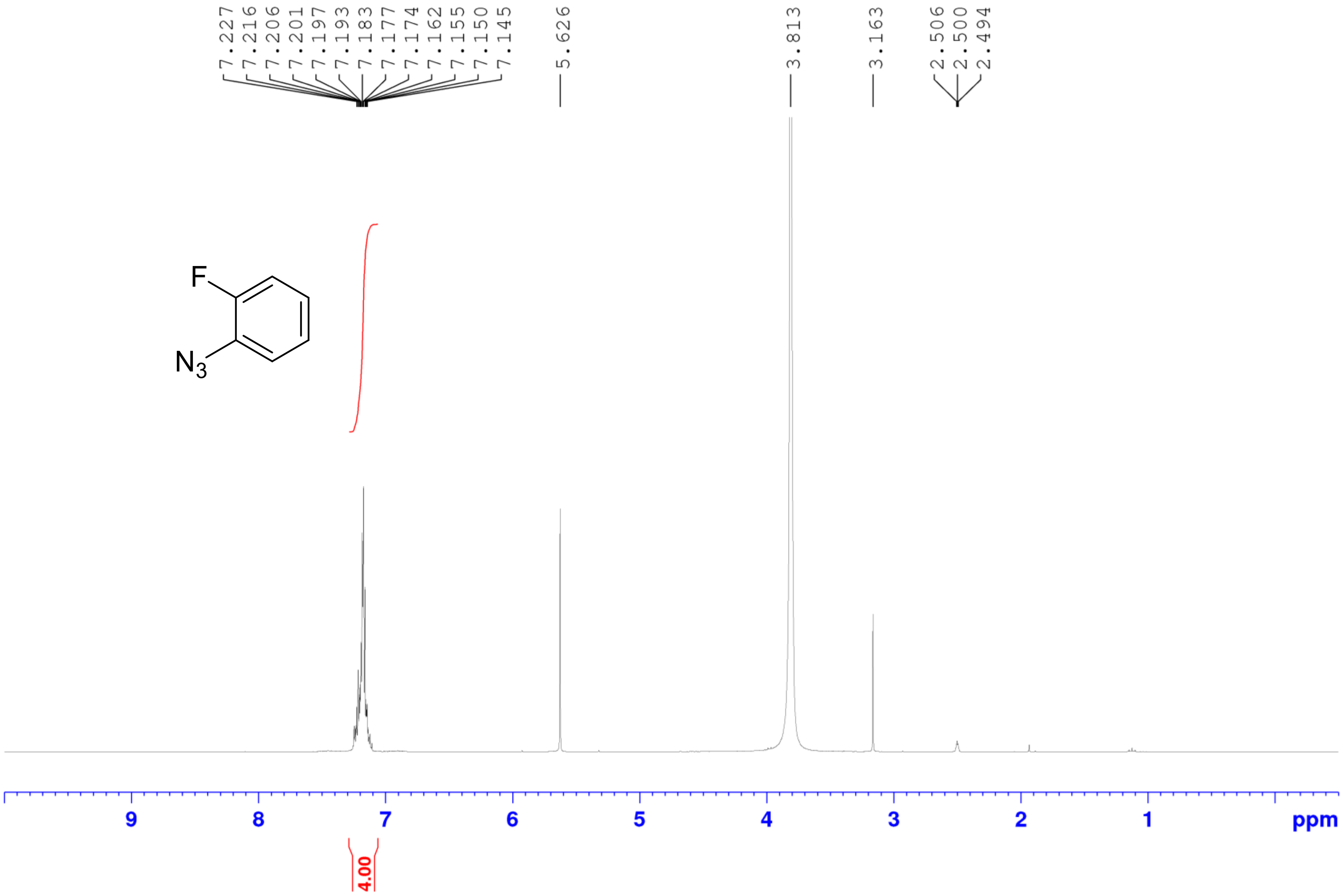

^{13}C NMR of compound **7b** (75 MHz, $CDCl_3$)

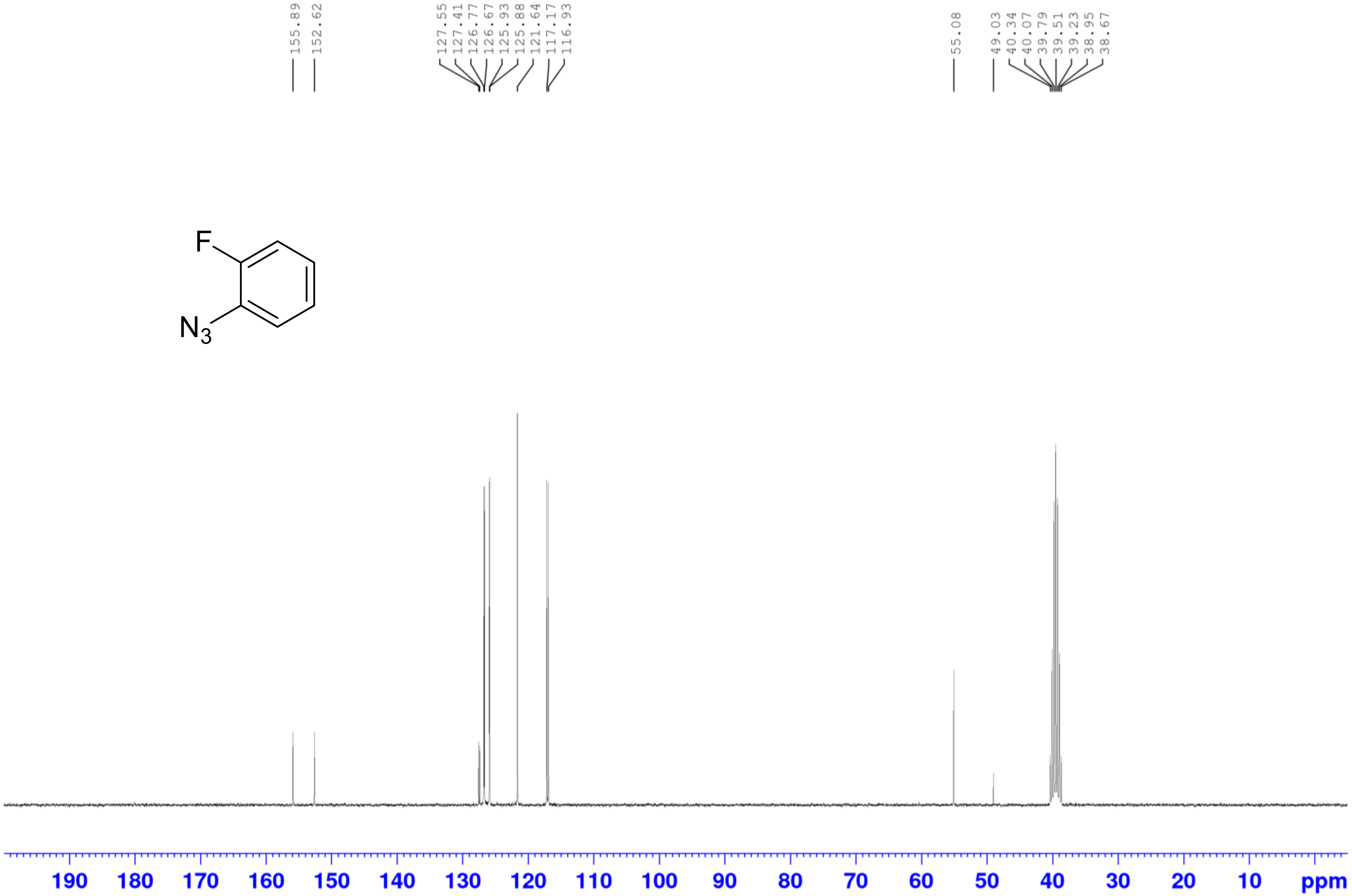

^{16}F NMR of compound **7b** (282 MHz, $CDCl_3$)

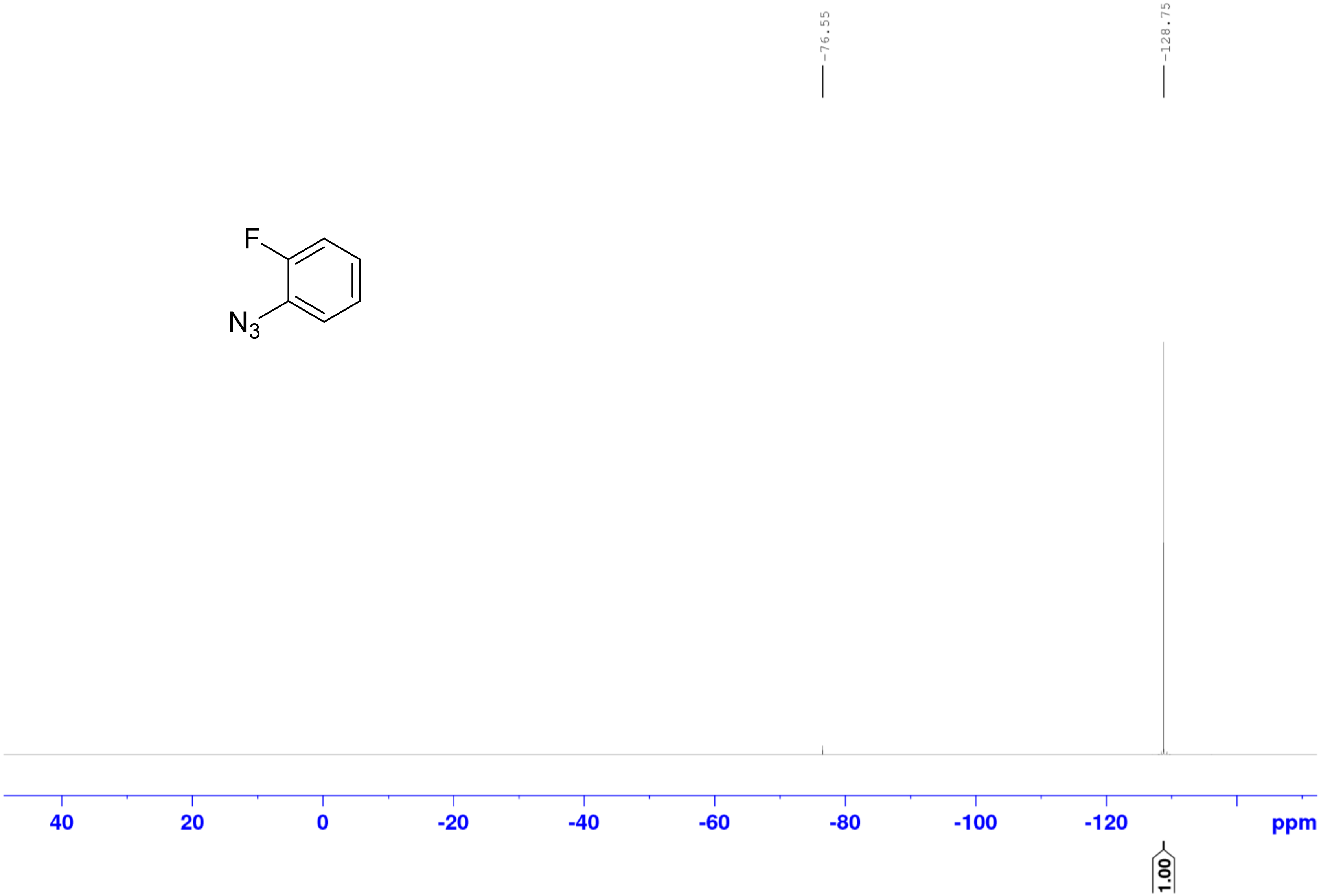

^{1}H NMR of compound **7c** (300 MHz, $CDCl_3$)

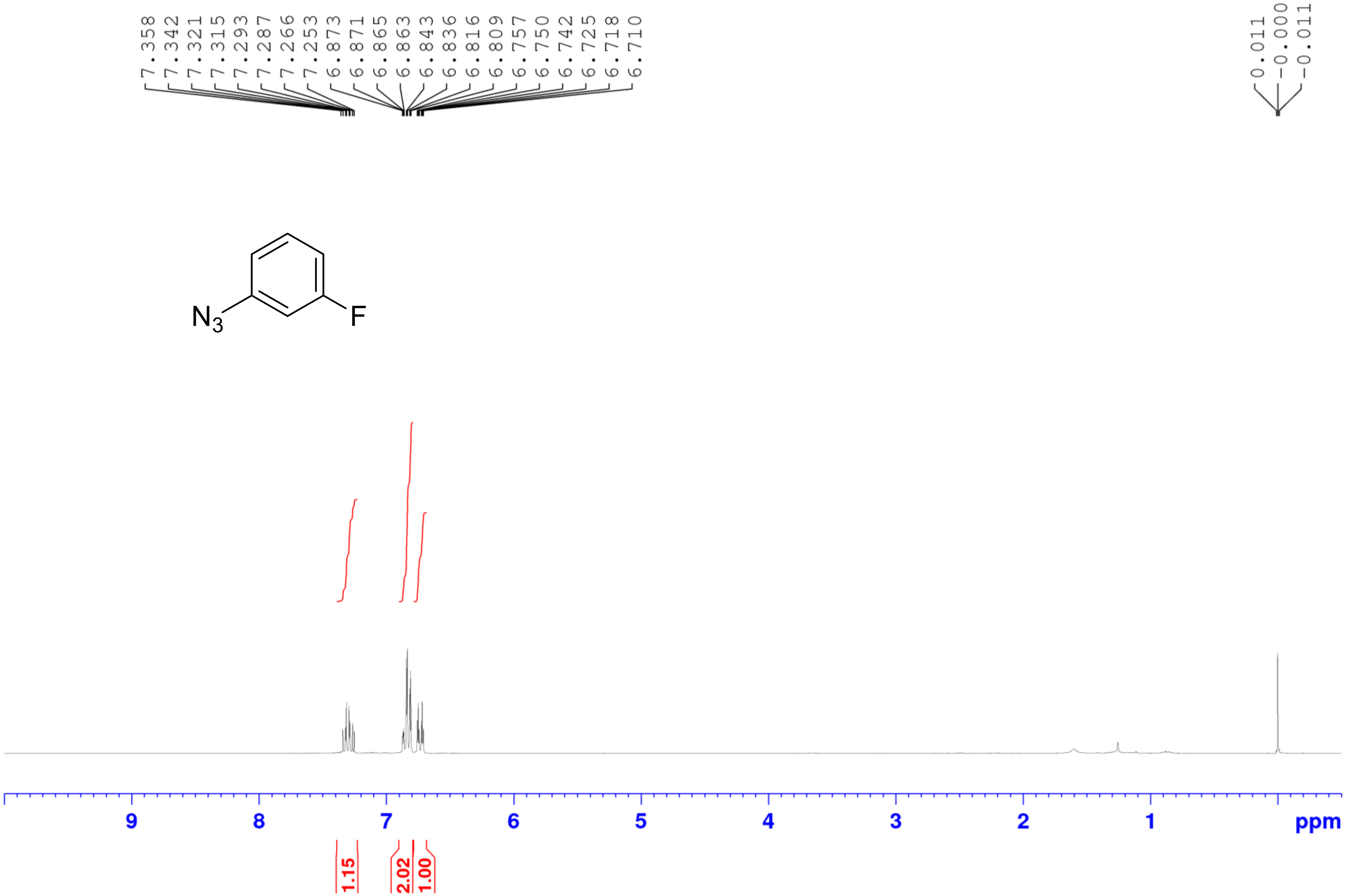

^{13}C NMR of compound **7c** (75 MHz, $CDCl_3$)

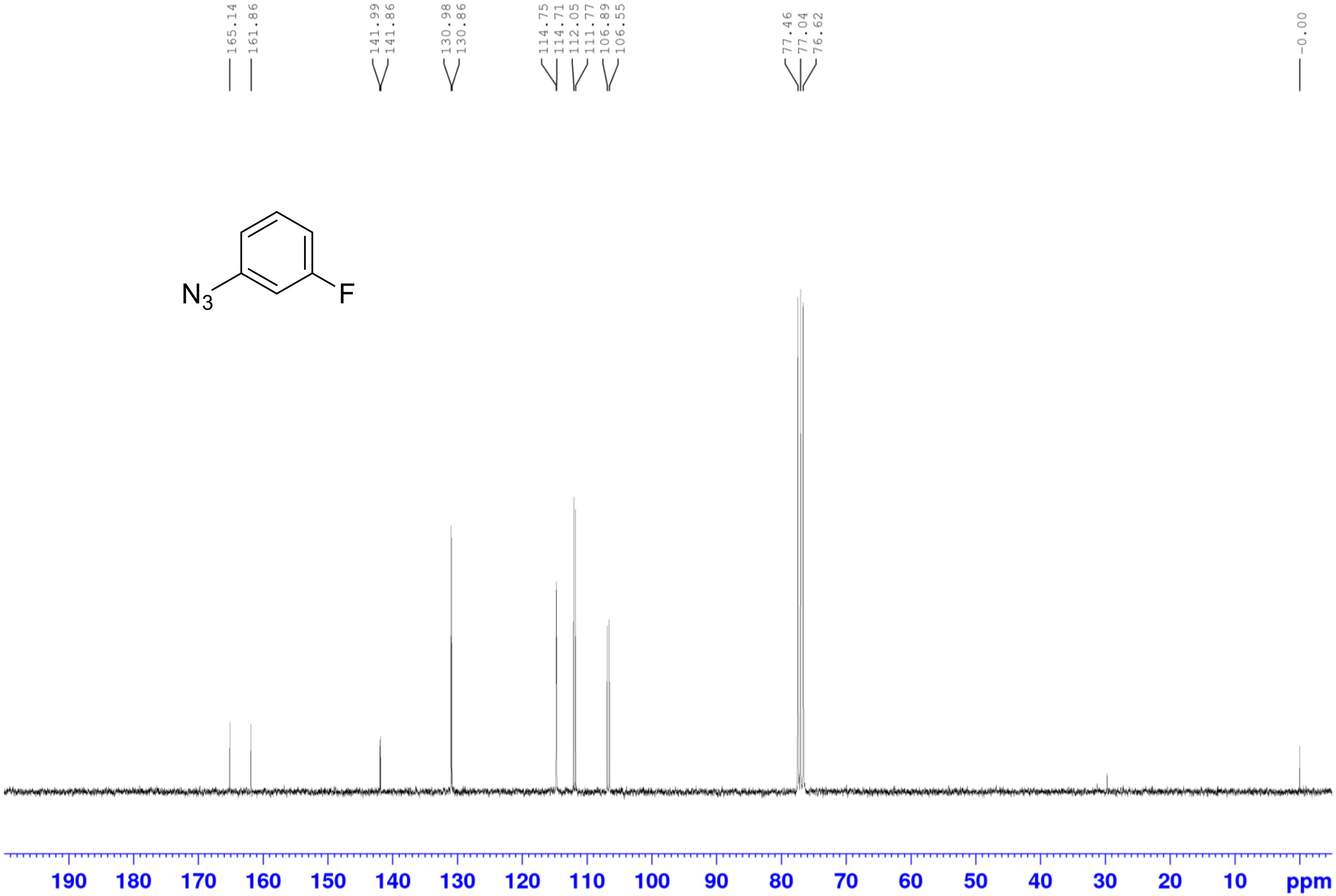

^{19}F NMR of compound **7c** (282 MHz, DMSO-*d*$_6$)

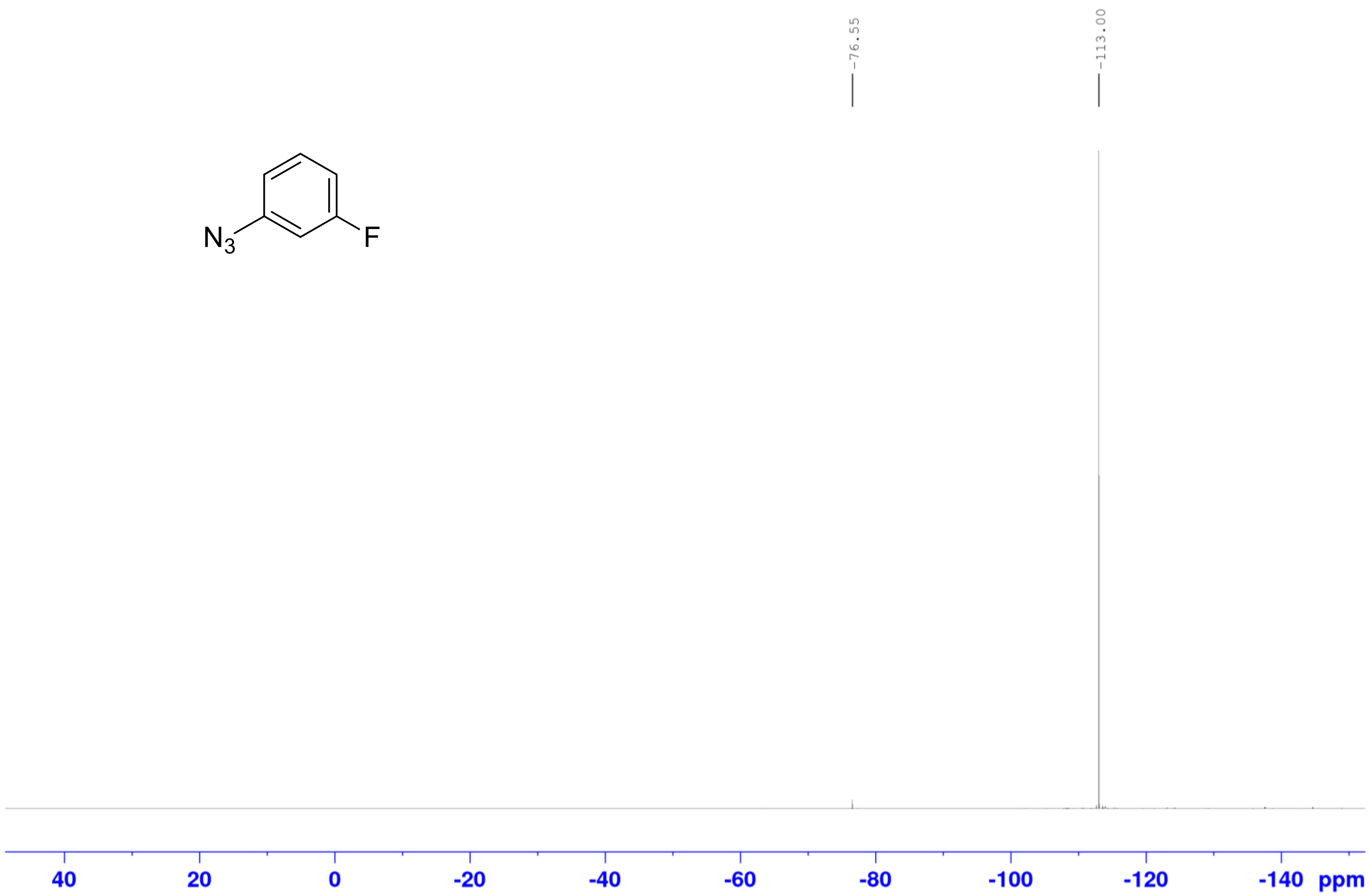

^{1}H NMR of compound **7d** (300 MHz, $CDCl_3$)

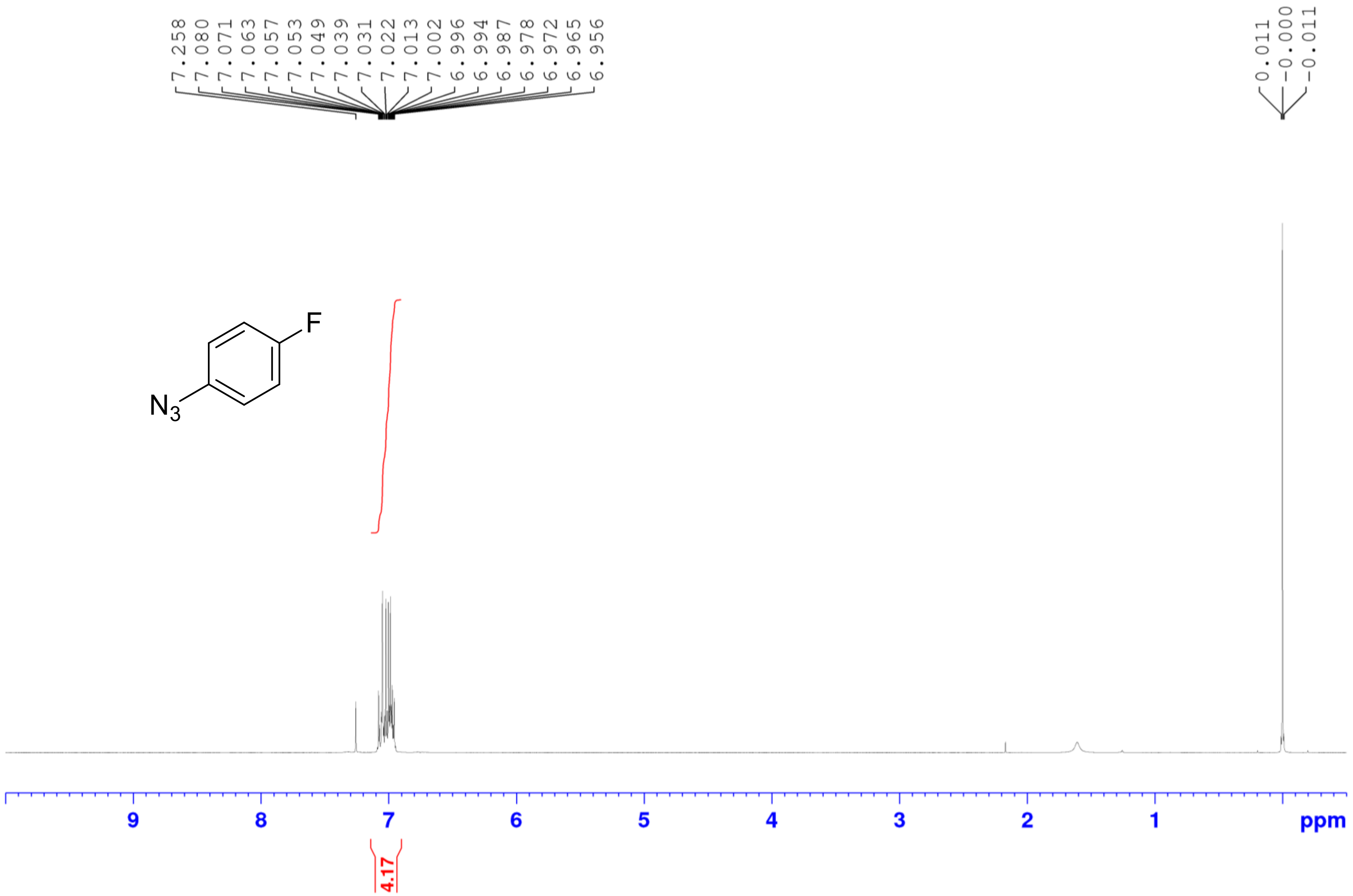

^{13}C NMR of compound **7d** (75 MHz, $CDCl_3$)

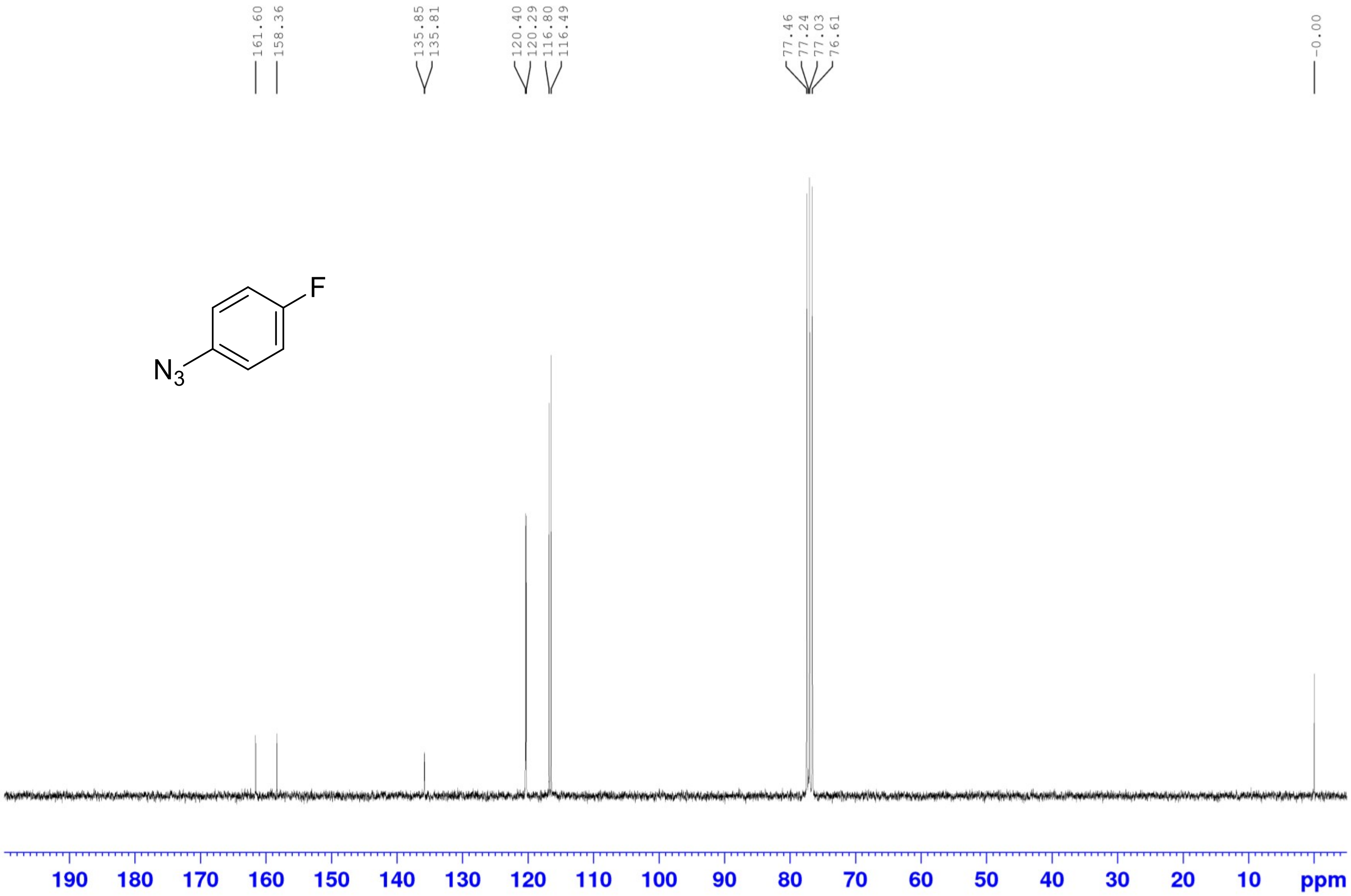

^{19}F NMR of compound **7d** (282 MHz, $CDCl_3$)

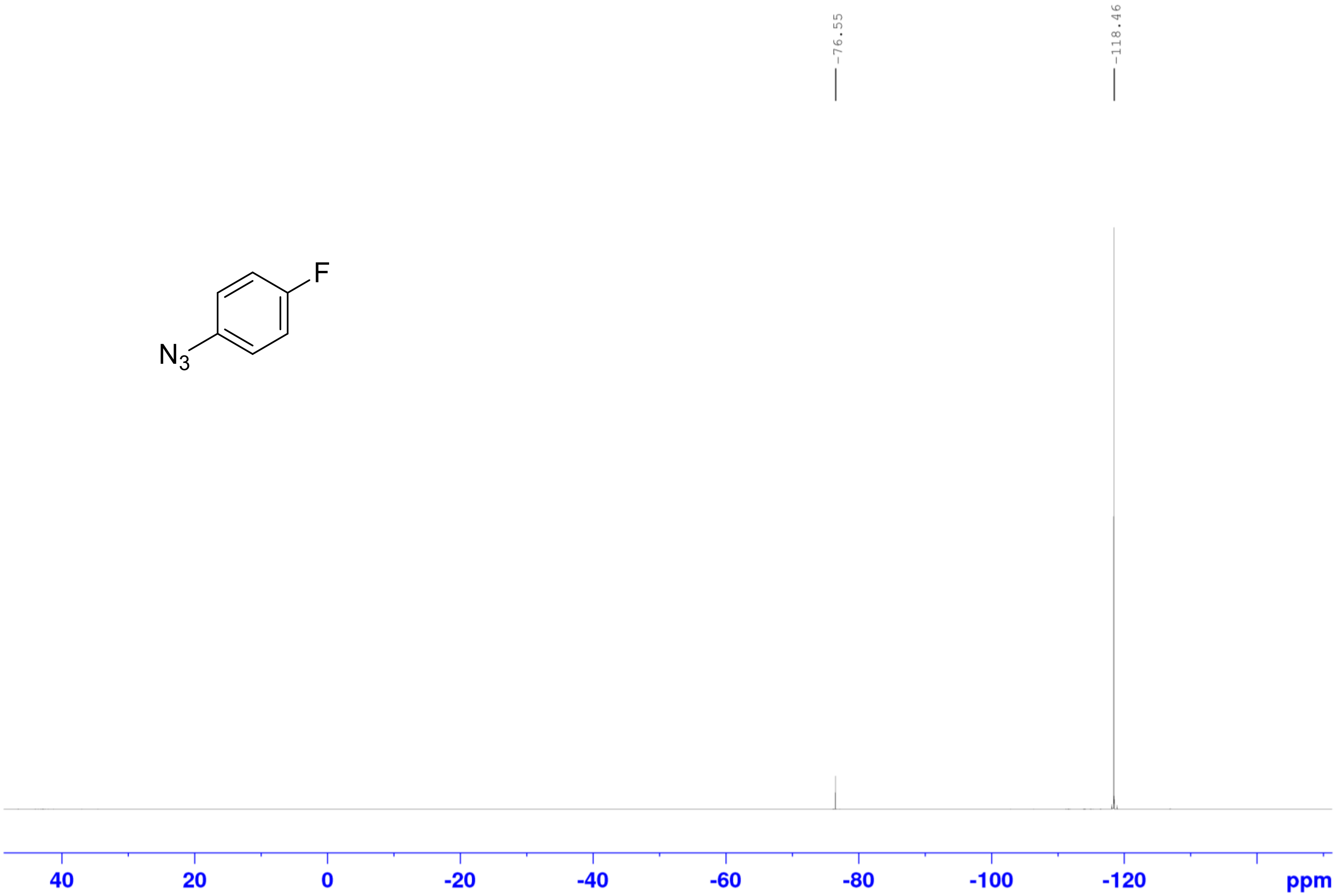

^{1}H NMR of compound **7e** (300 MHz, $CDCl_3$)

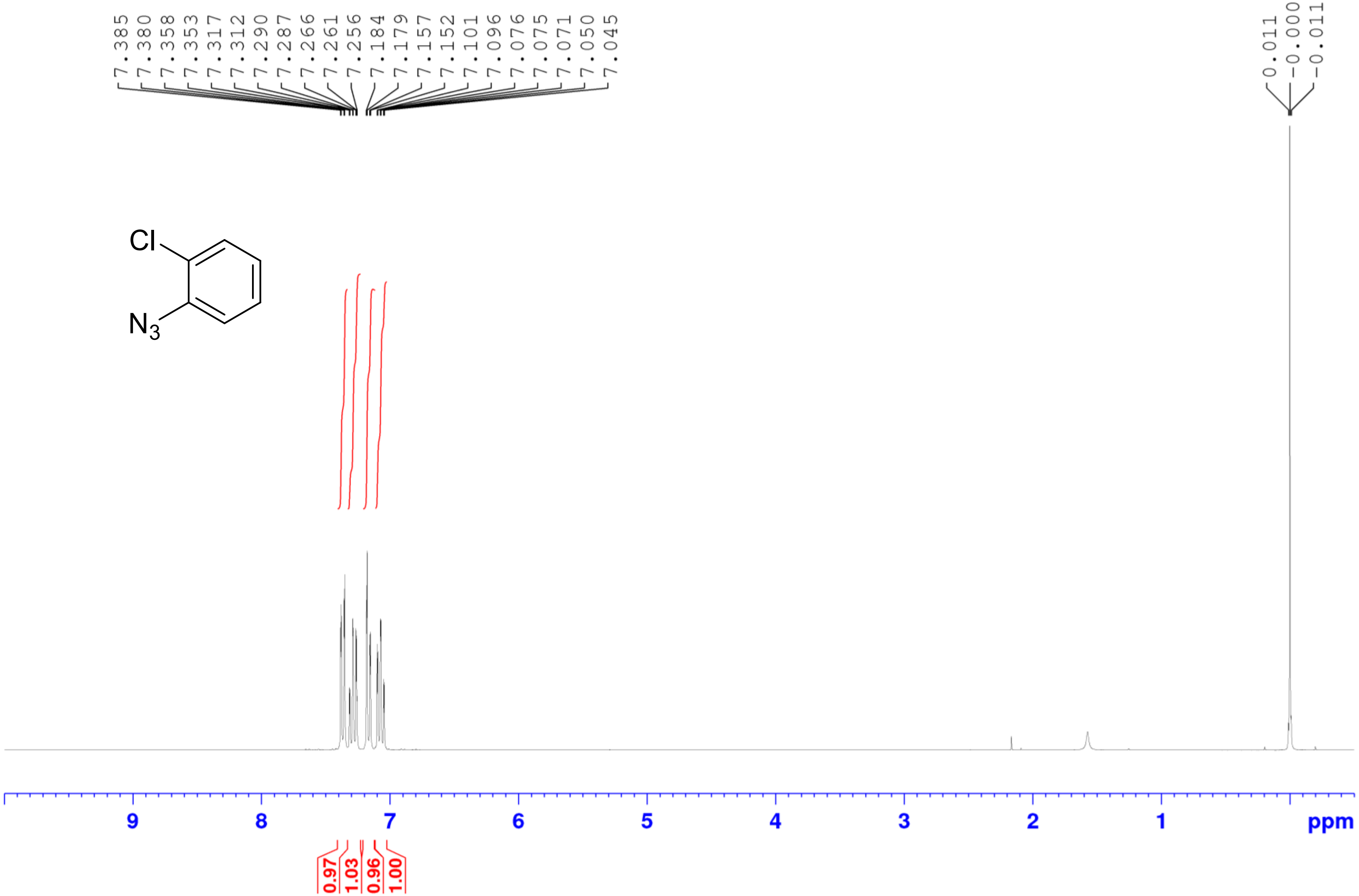

^{13}C NMR of compound **7e** (75 MHz, $CDCl_3$)

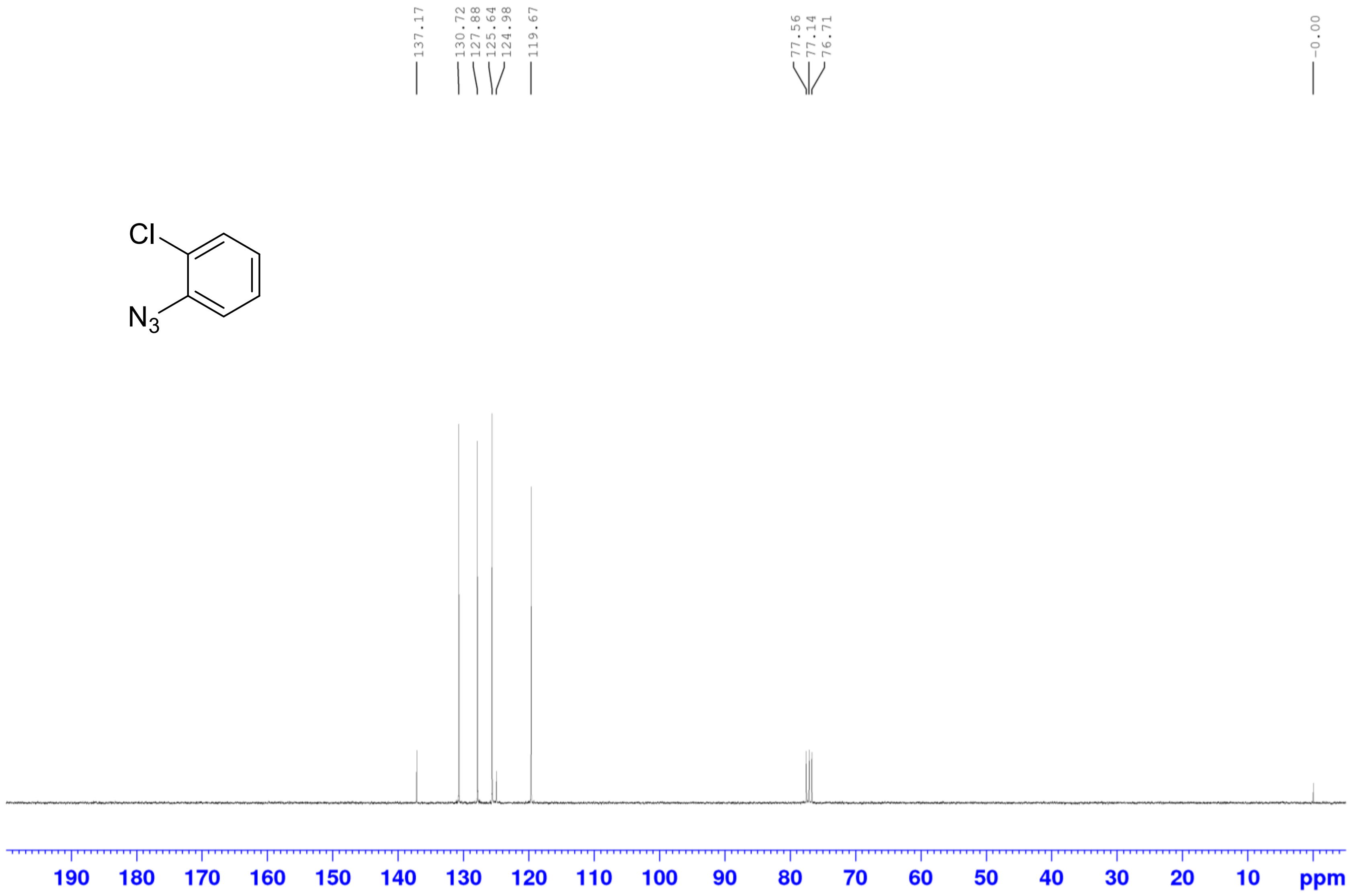

^{1}H NMR of compound **7f** (300 MHz, $CDCl_3$)

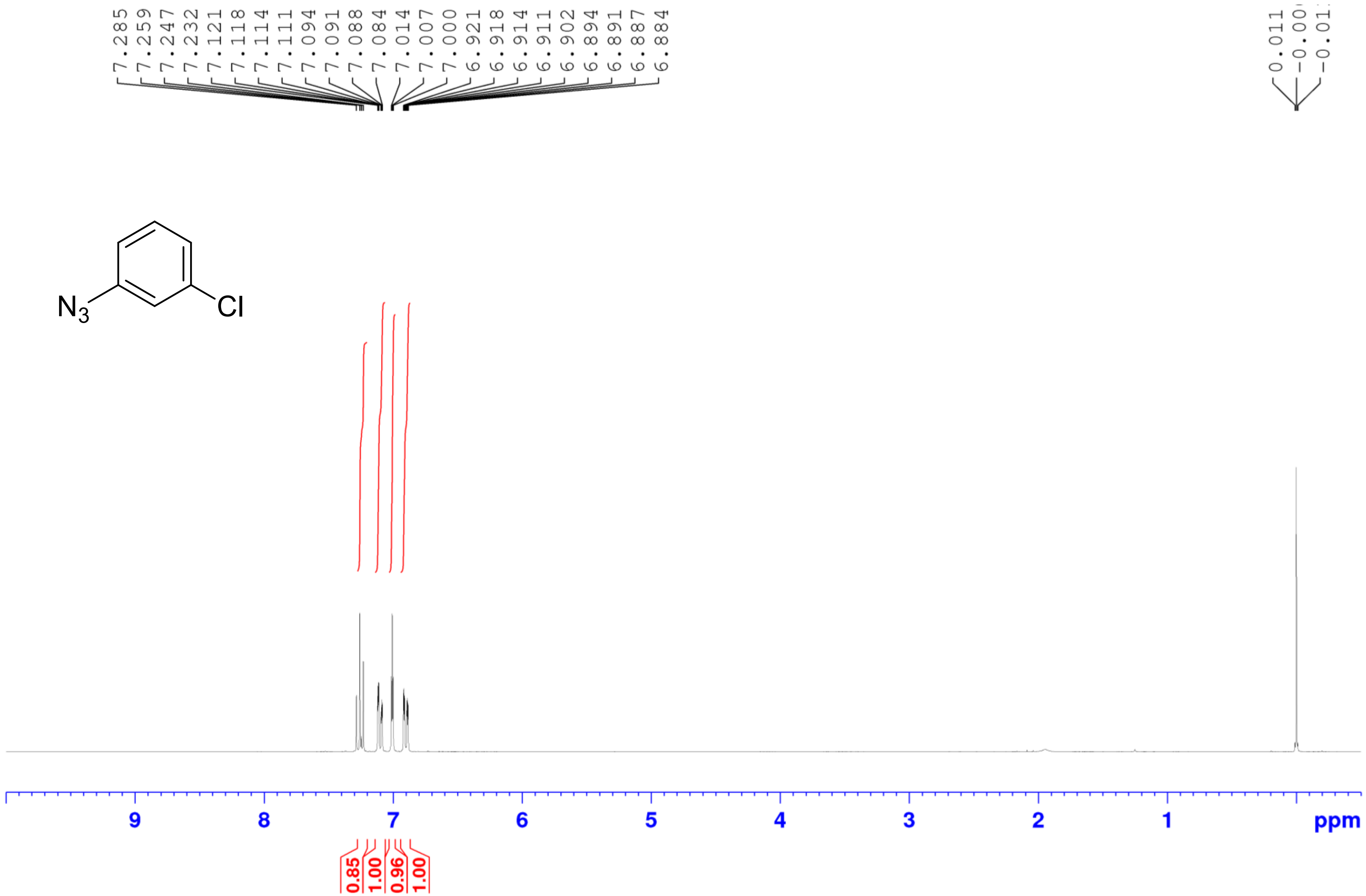

^{13}C NMR of compound **7f** (75 MHz, $CDCl_3$)

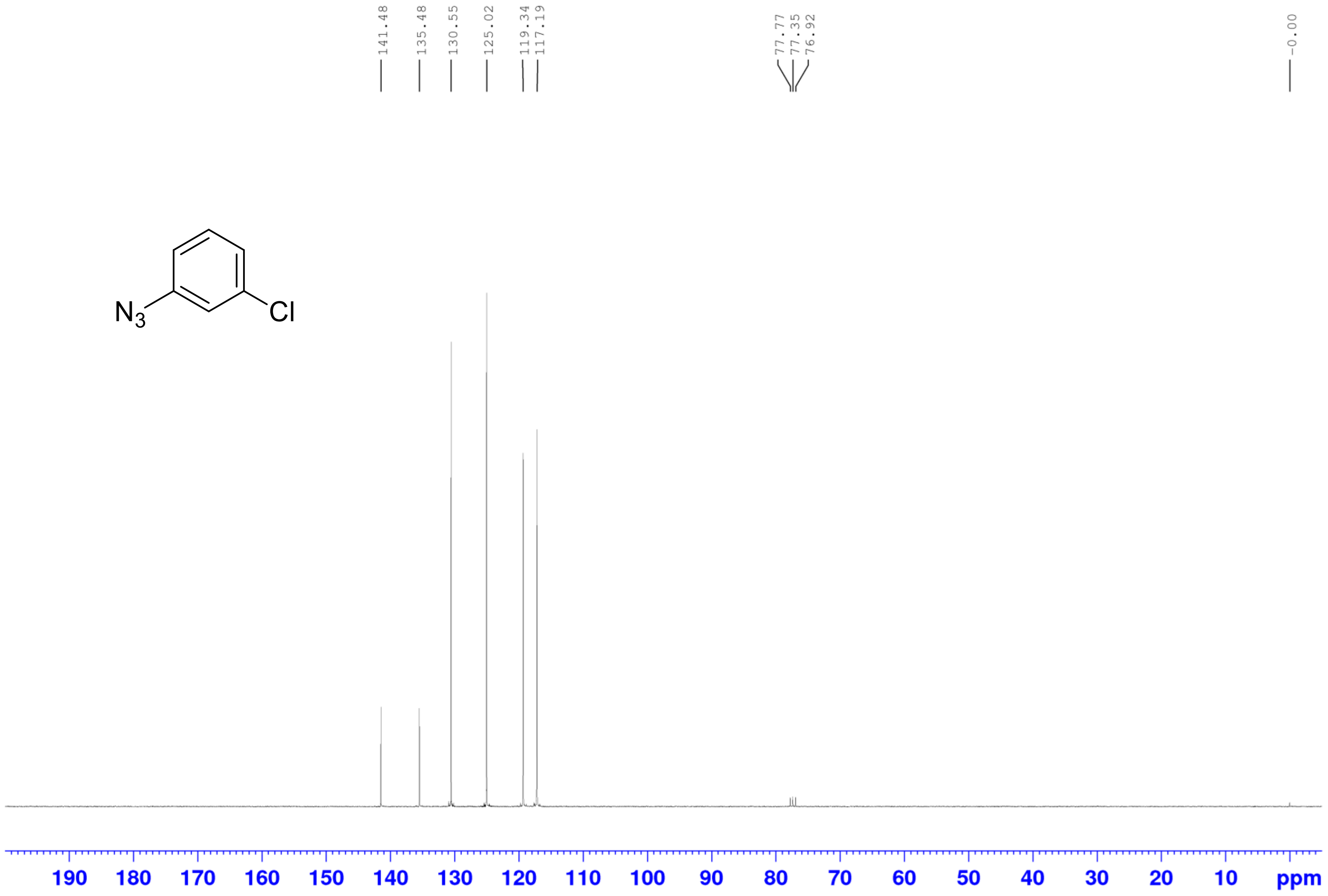

^{1}H NMR of compound **7g** (300 MHz, $CDCl_3$)

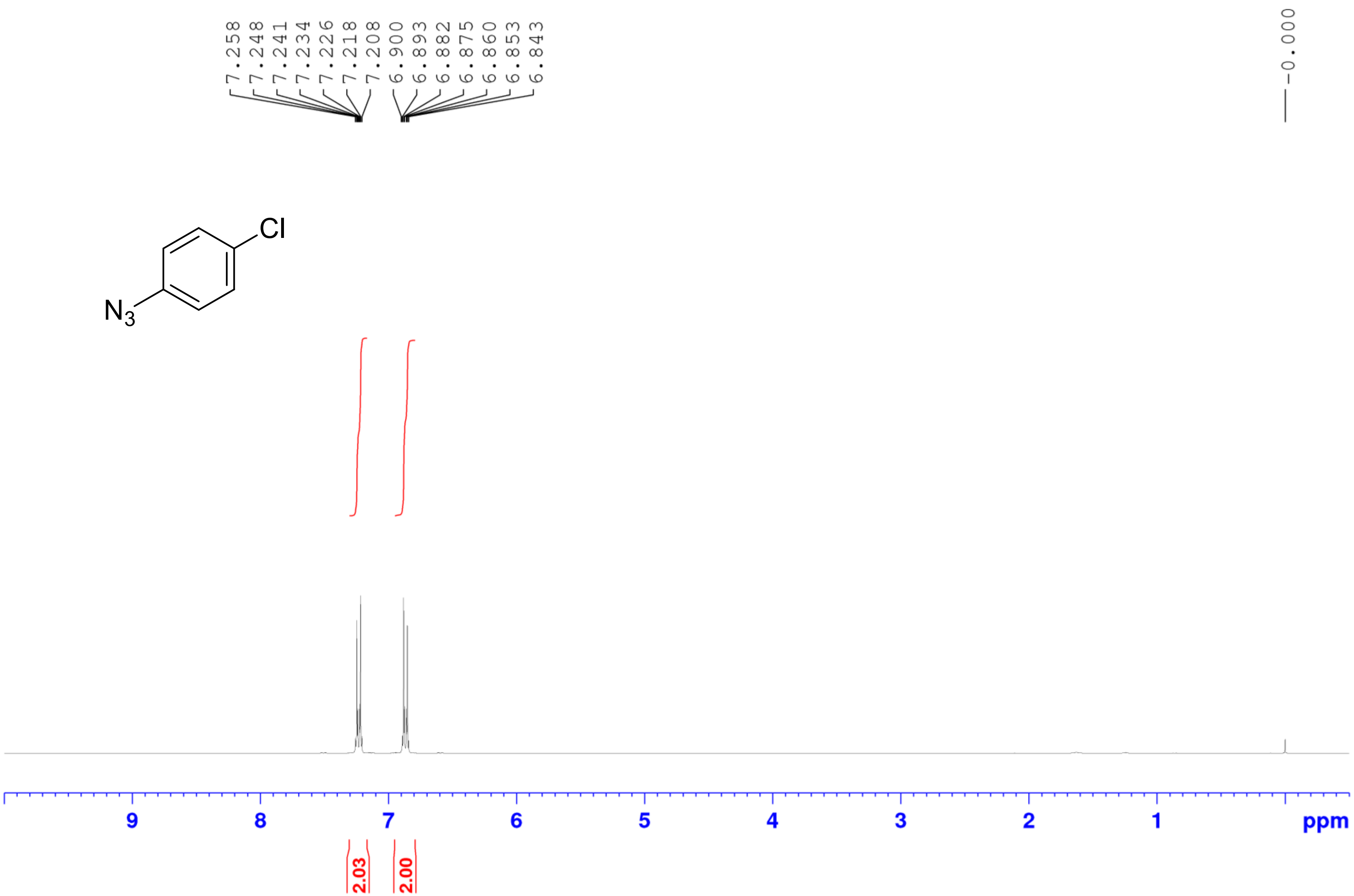

^{13}C NMR of compound **7g** (75 MHz, $CDCl_3$)

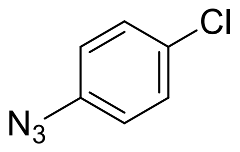

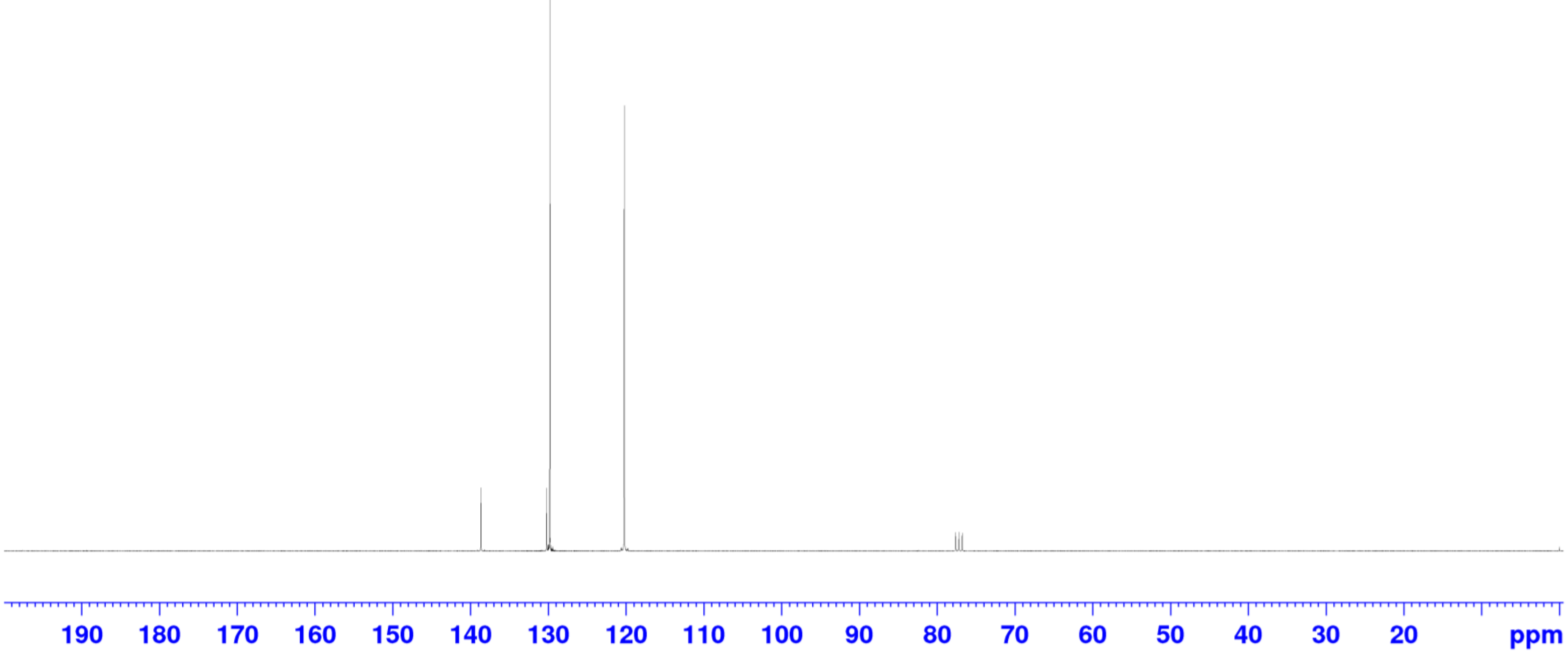

^{1}H NMR of compound **7h** (300 MHz, $CDCl_3$)

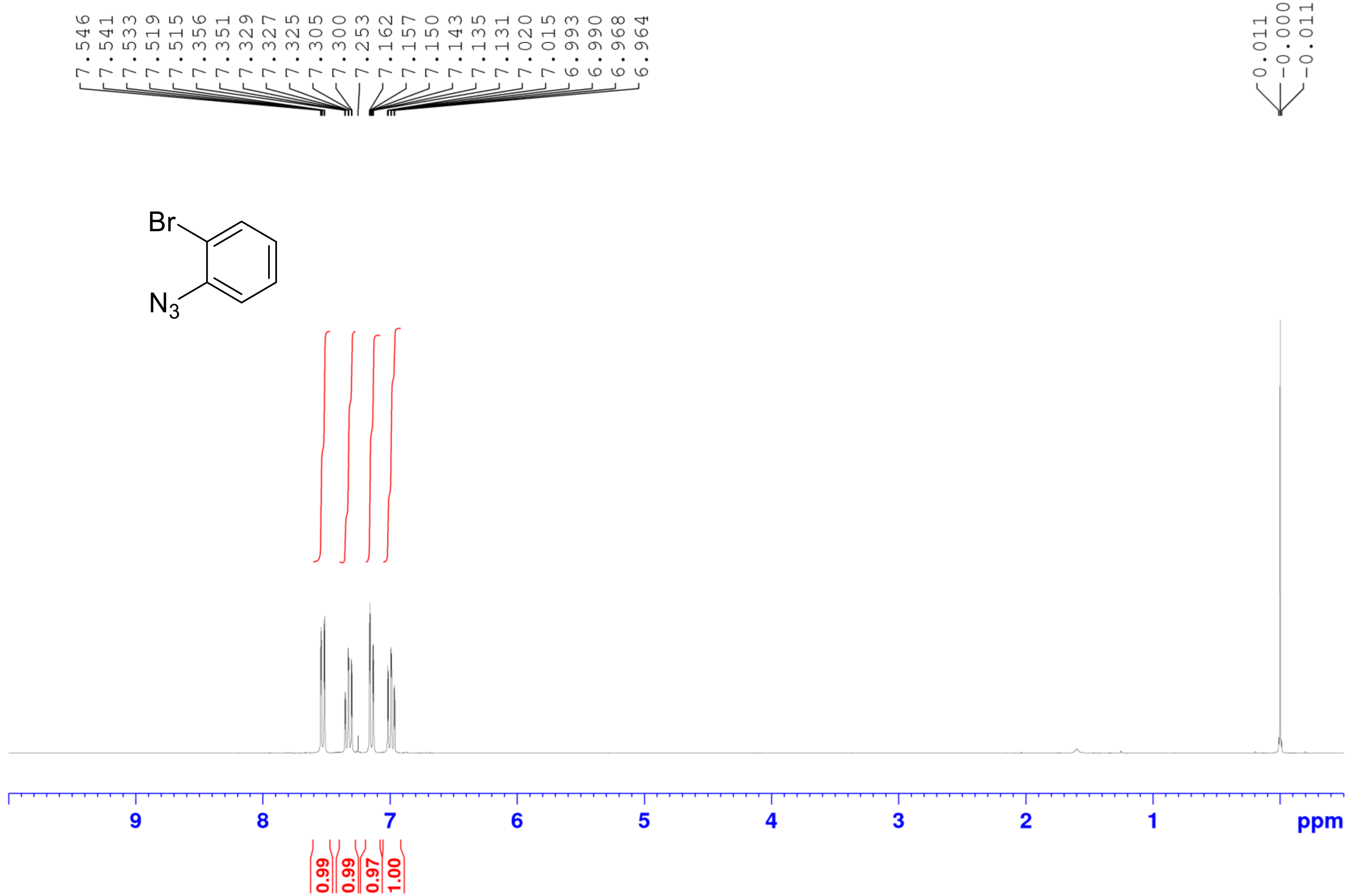

^{13}C NMR of compound **7h** (75 MHz, $CDCl_3$)

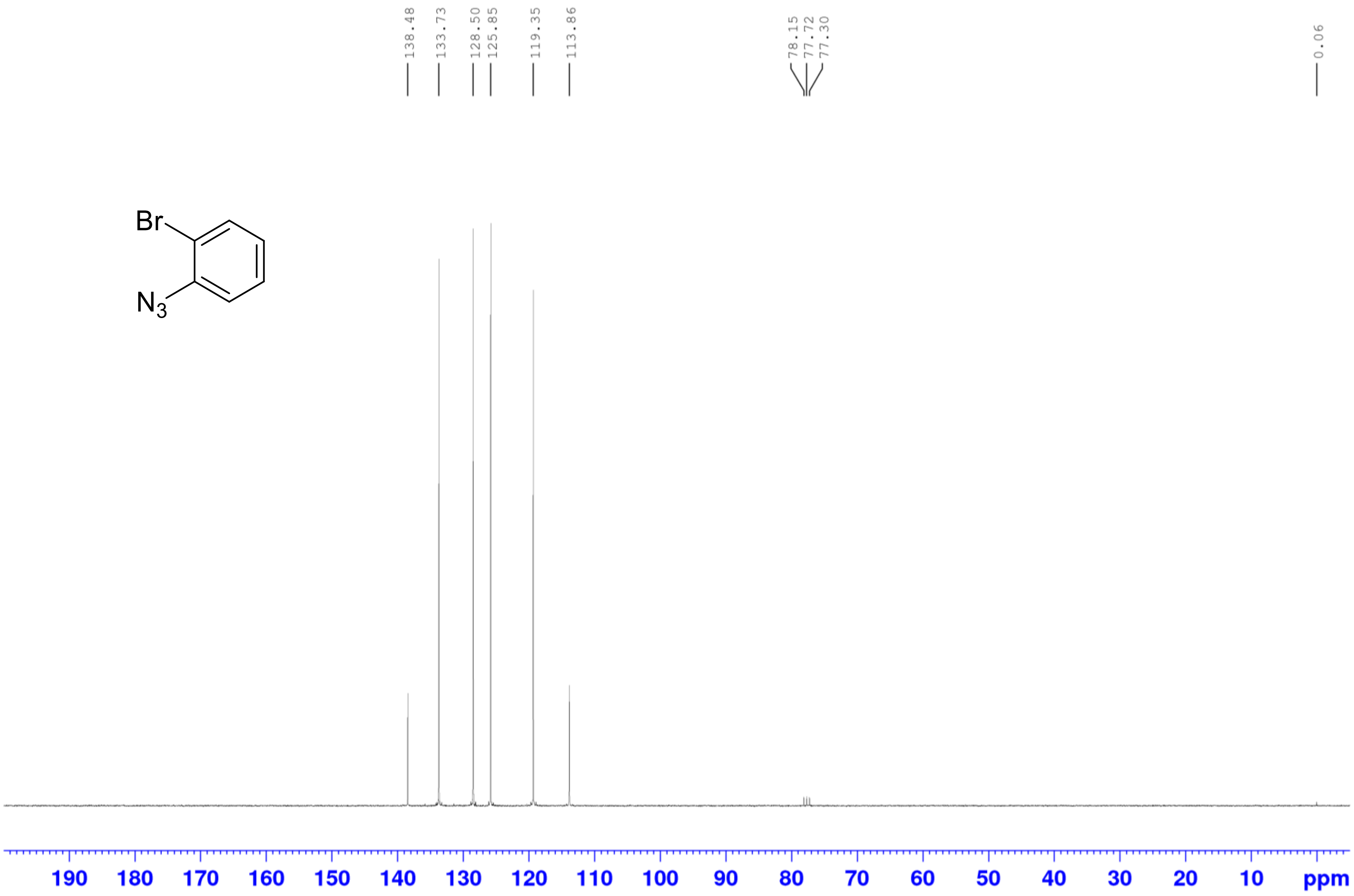

^{1}H NMR of compound **7i** (300 MHz, $CDCl_3$)

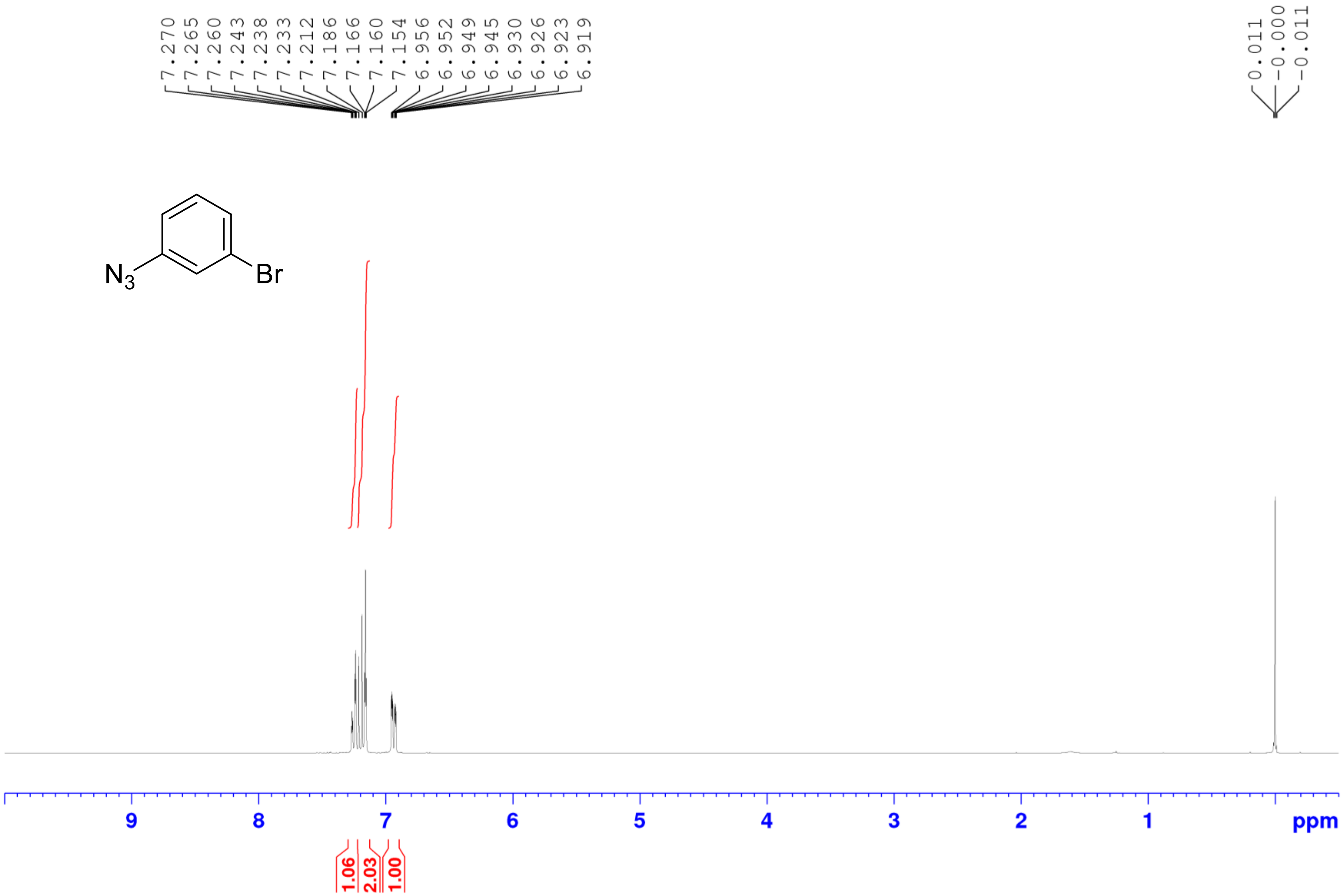

^{13}C NMR of compound **7i** (75 MHz, $CDCl_3$)

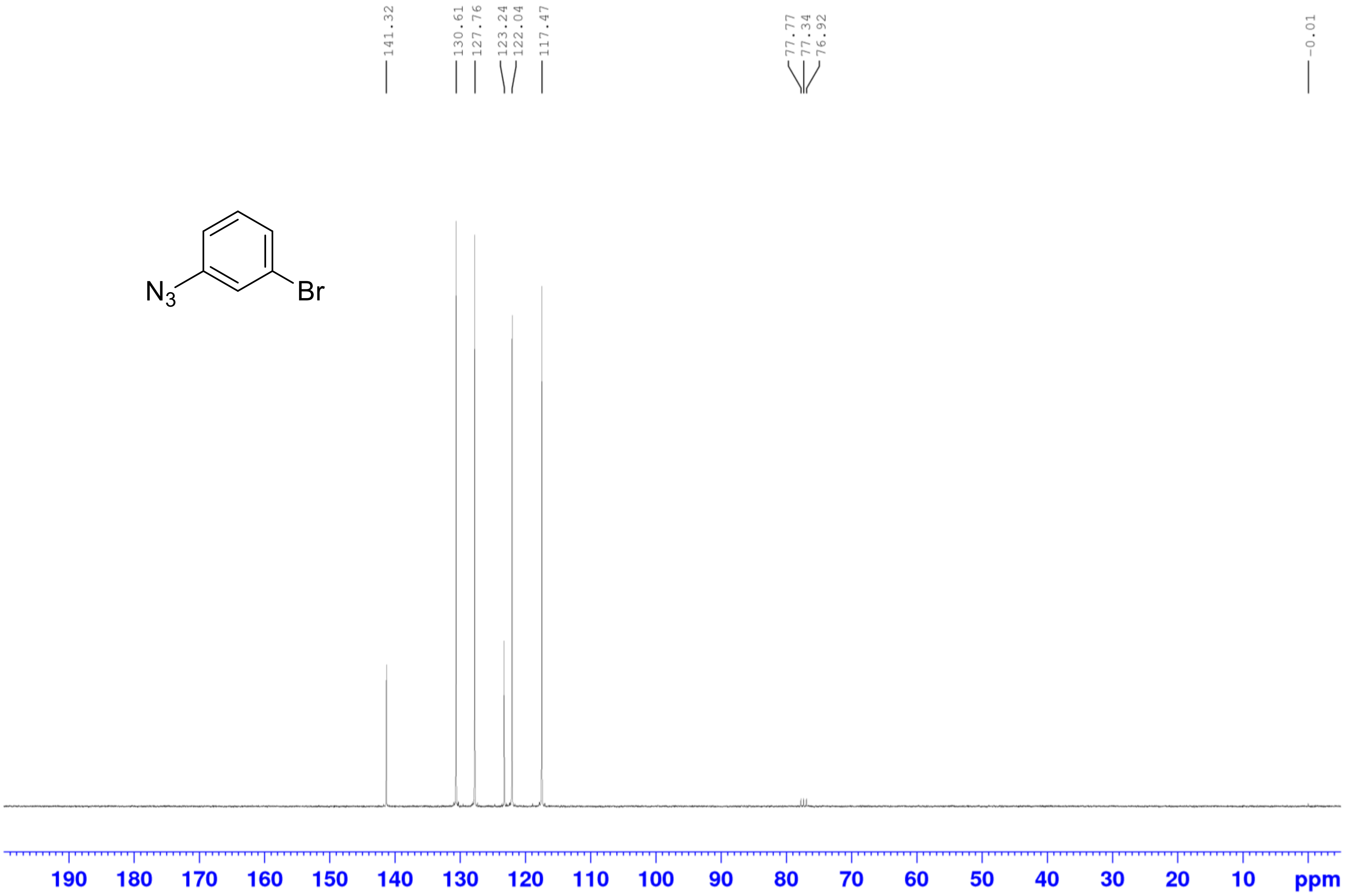

^{1}H NMR of compound **7j** (300 MHz, $CDCl_3$)

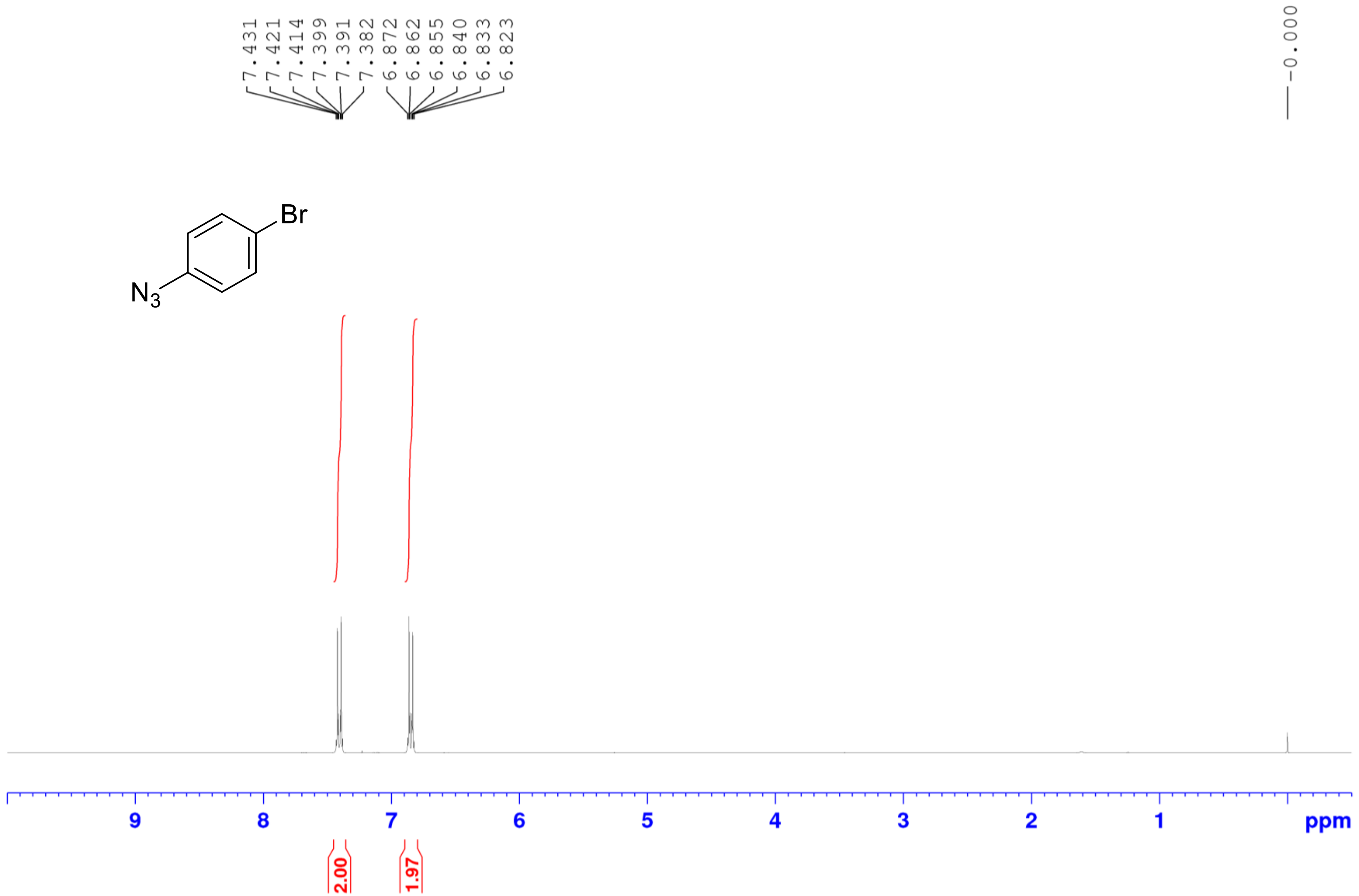

^{13}C NMR of compound **7j** (75 MHz, $CDCl_3$)

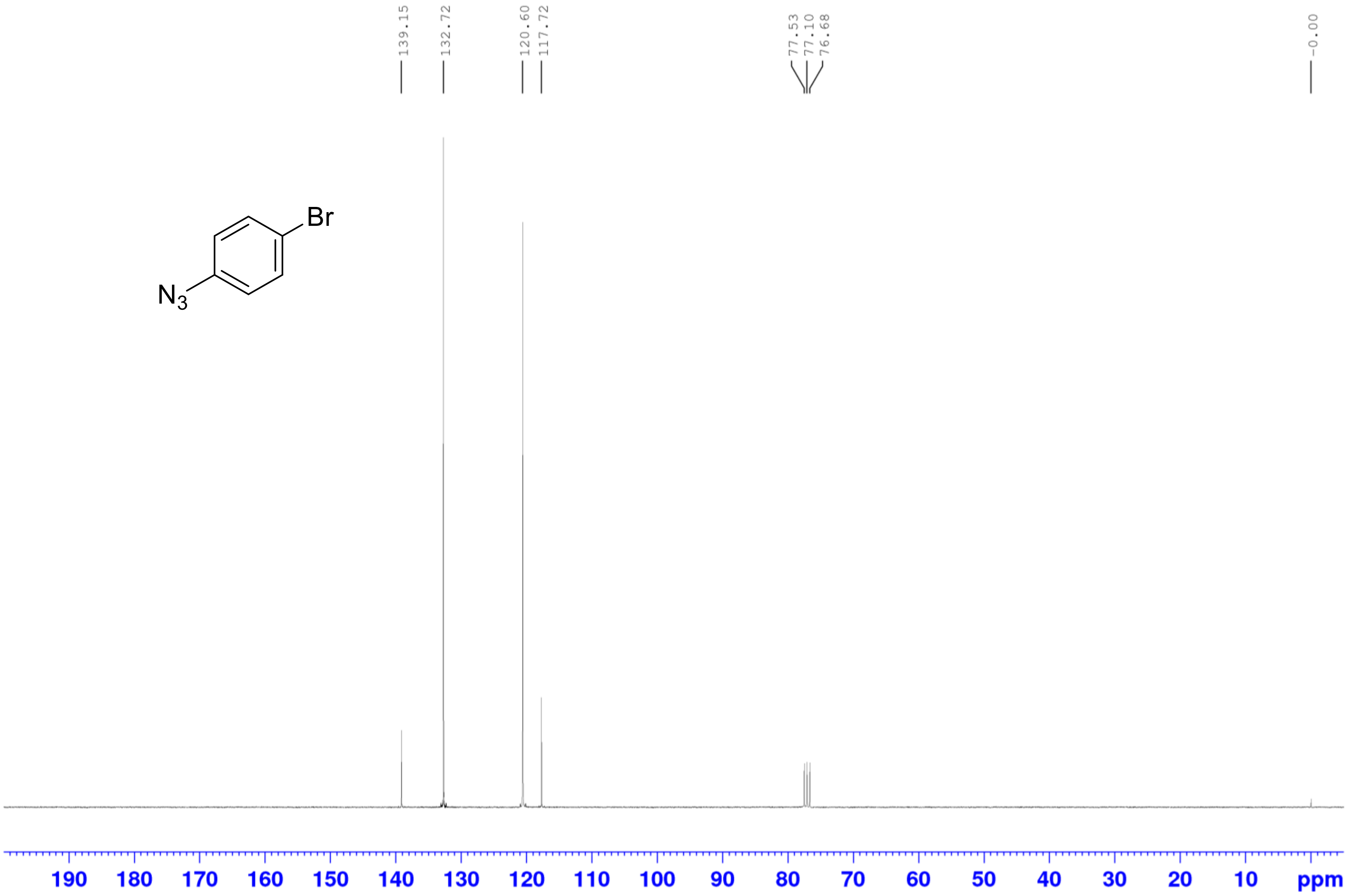

^{1}H NMR of compound **7k** (300 MHz, $CDCl_3$)

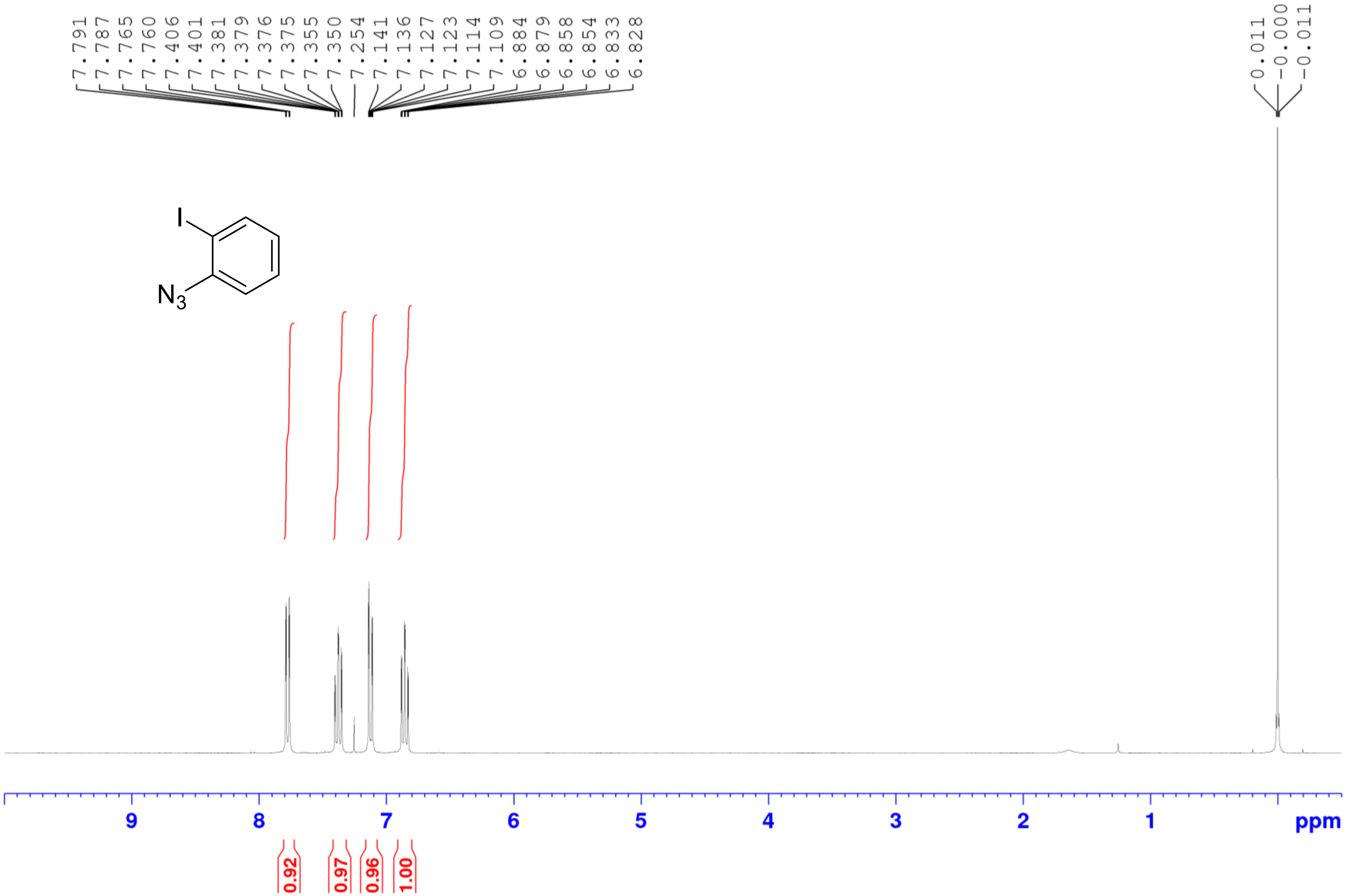

^{13}C NMR of compound **7k** (75 MHz, $CDCl_3$)

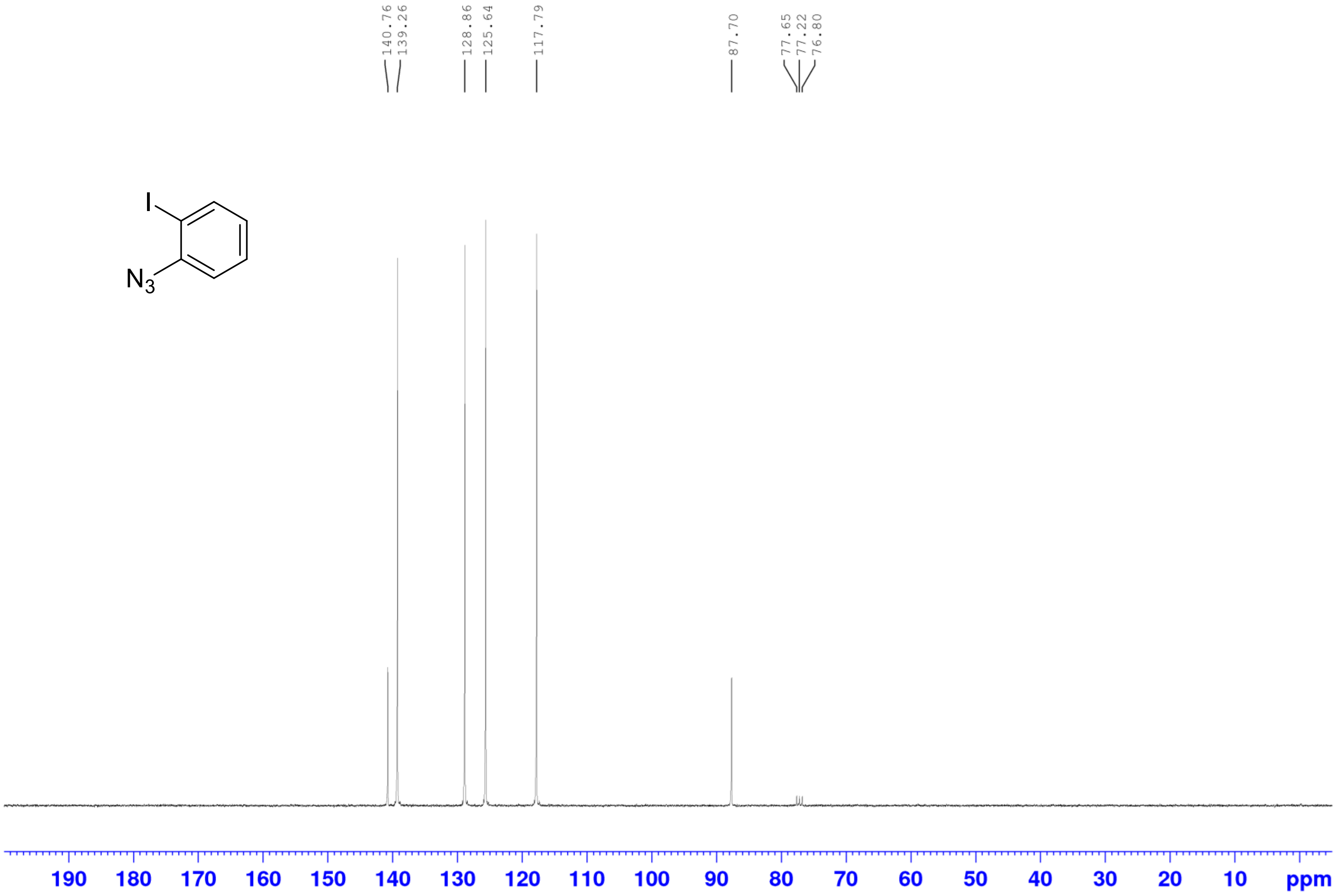

^{1}H NMR of compound **7l** (300 MHz, $CDCl_3$)

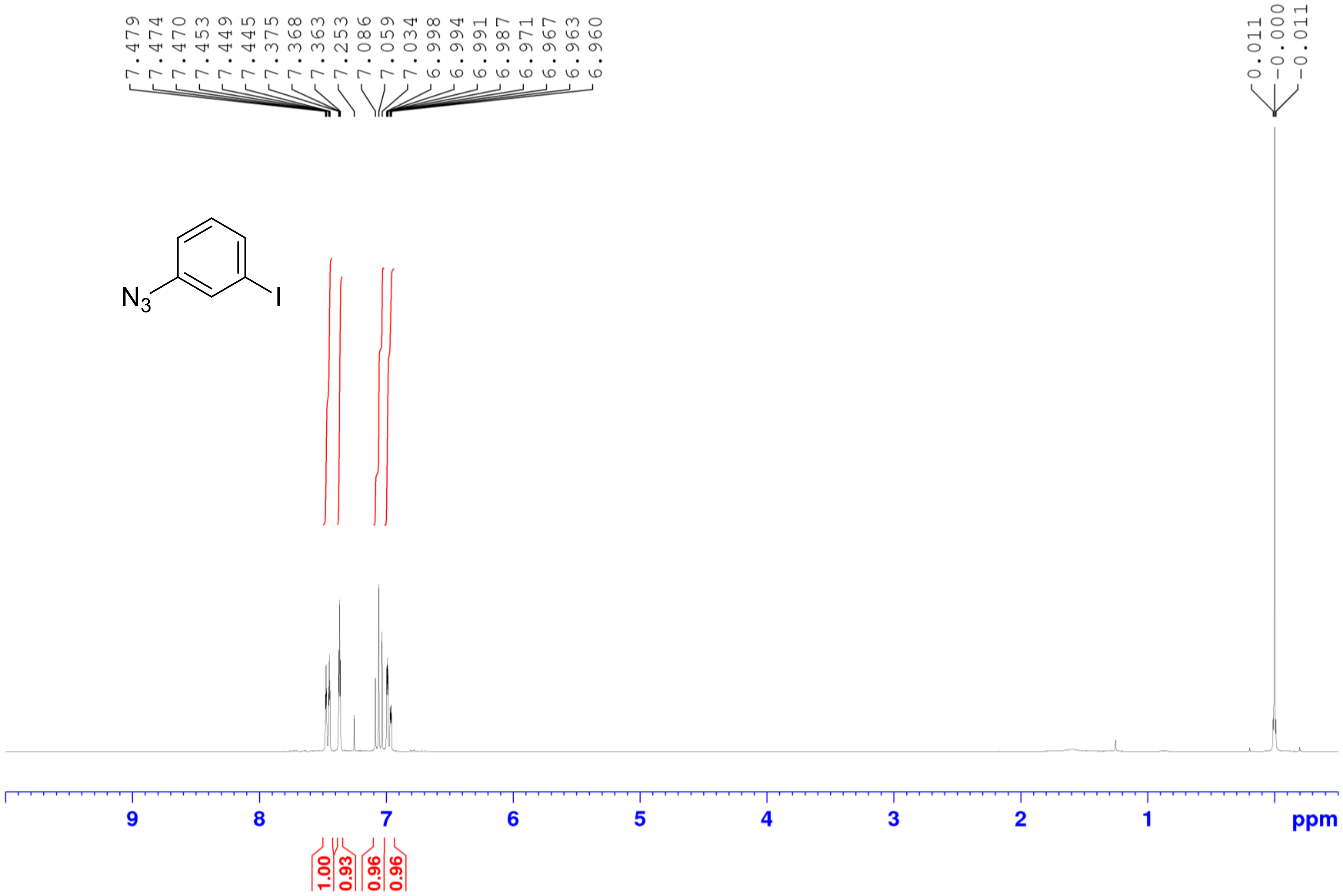

^{13}C NMR of compound **7l** (75 MHz, $CDCl_3$)

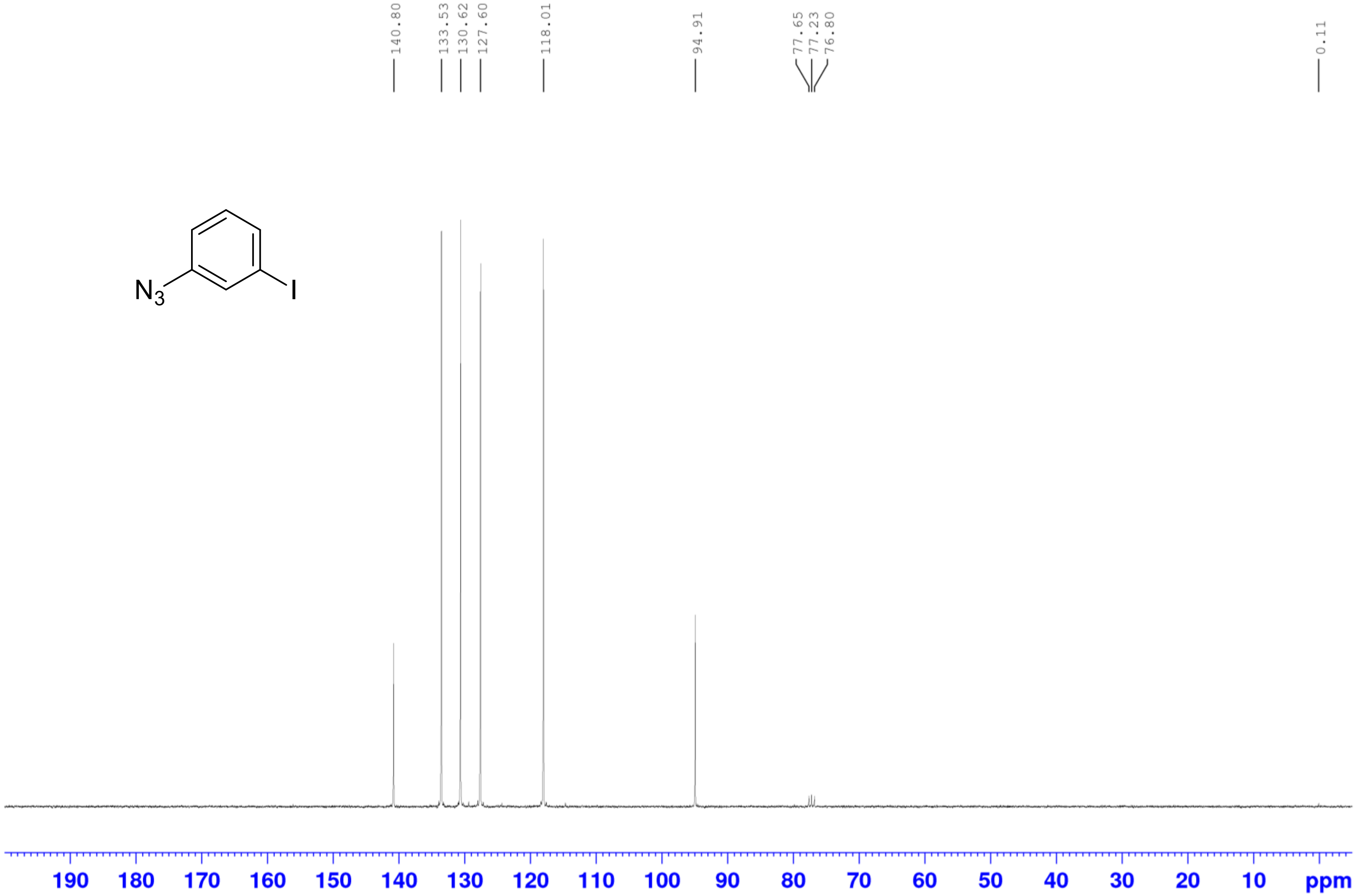

^{1}H NMR of compound **7m** (300 MHz, $CDCl_3$)

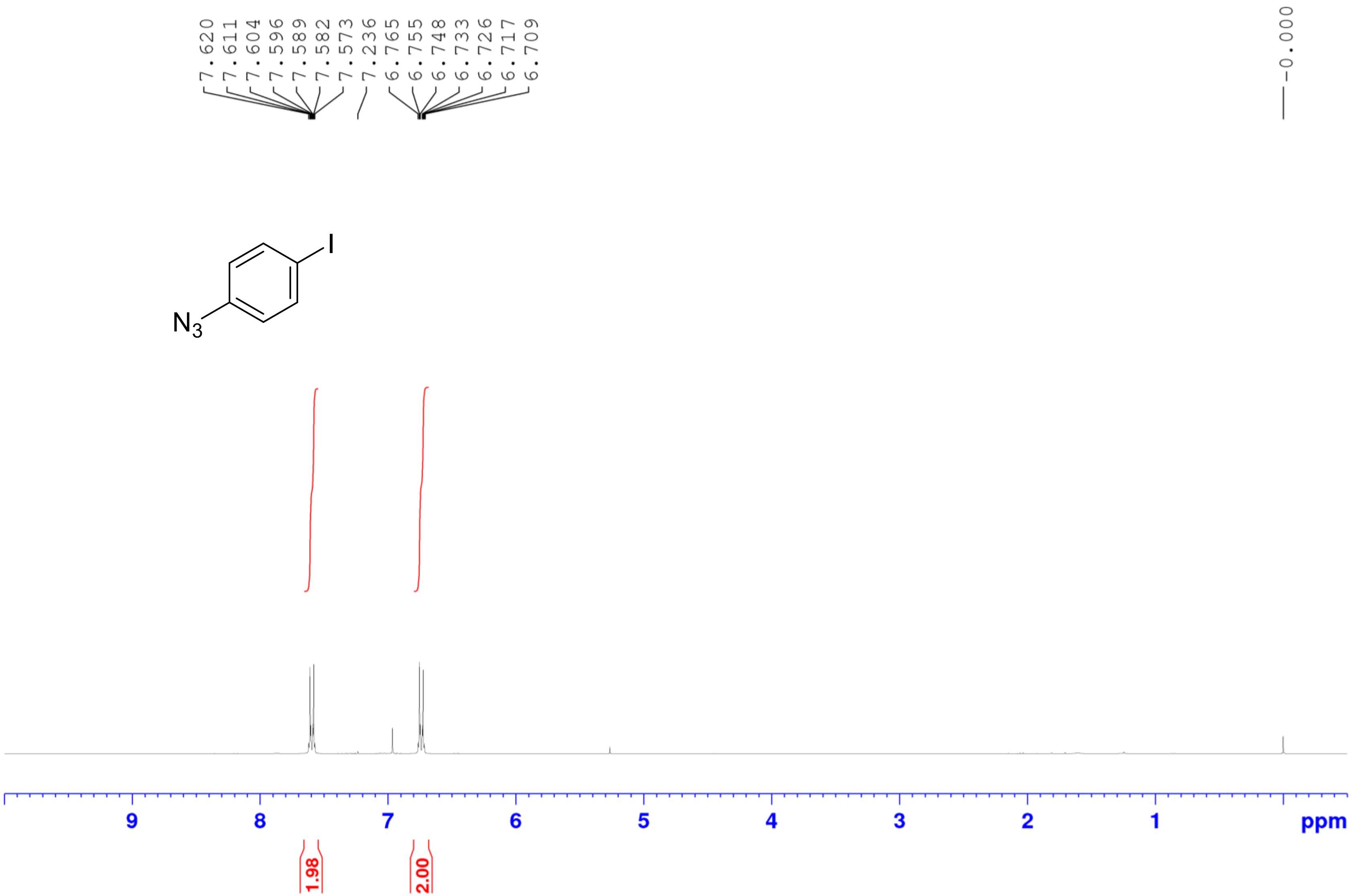

^{13}C NMR of compound **7m** (75 MHz, $CDCl_3$)

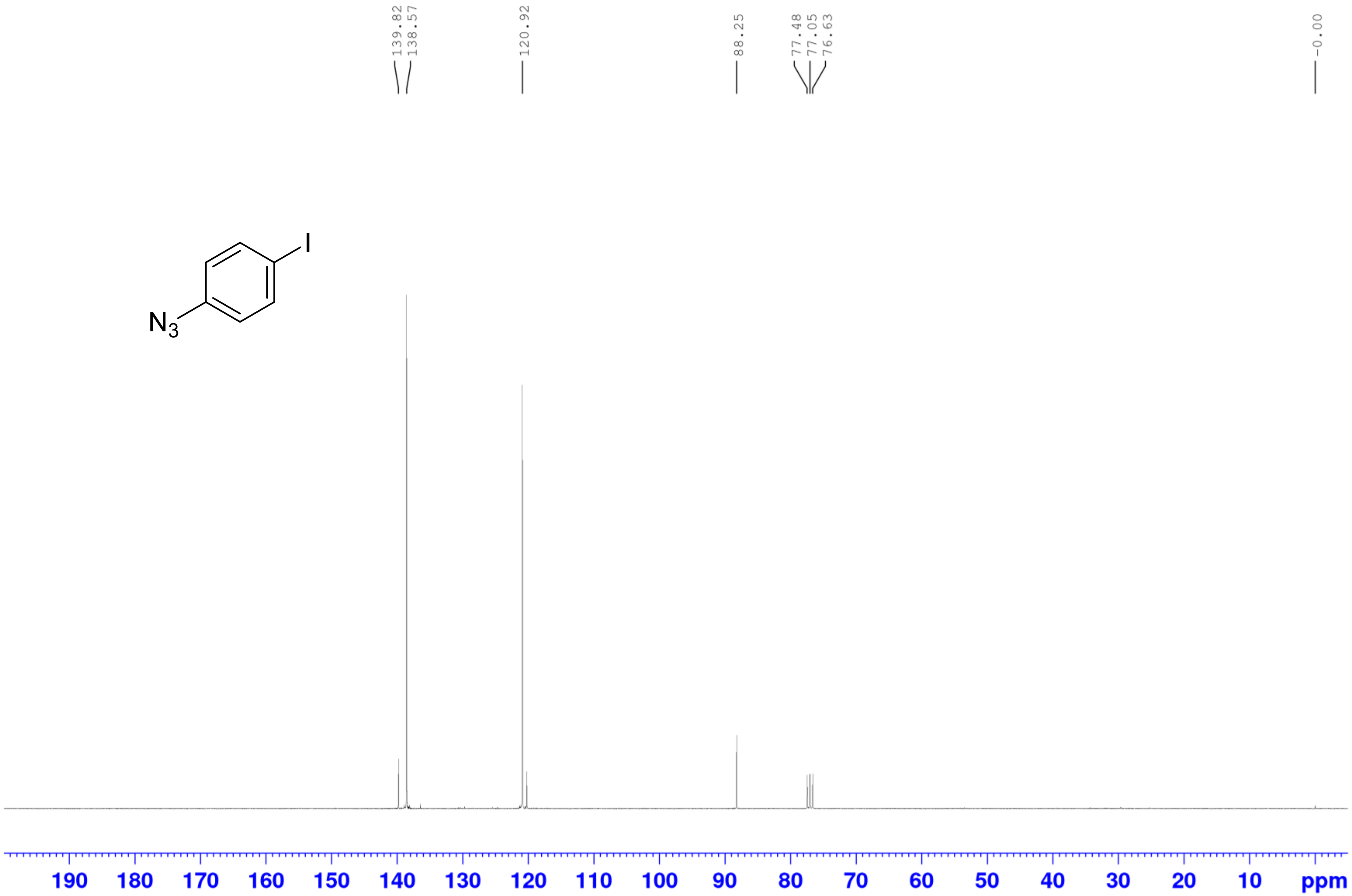

^{1}H NMR of compound **7n** (300 MHz, $CDCl_3$)

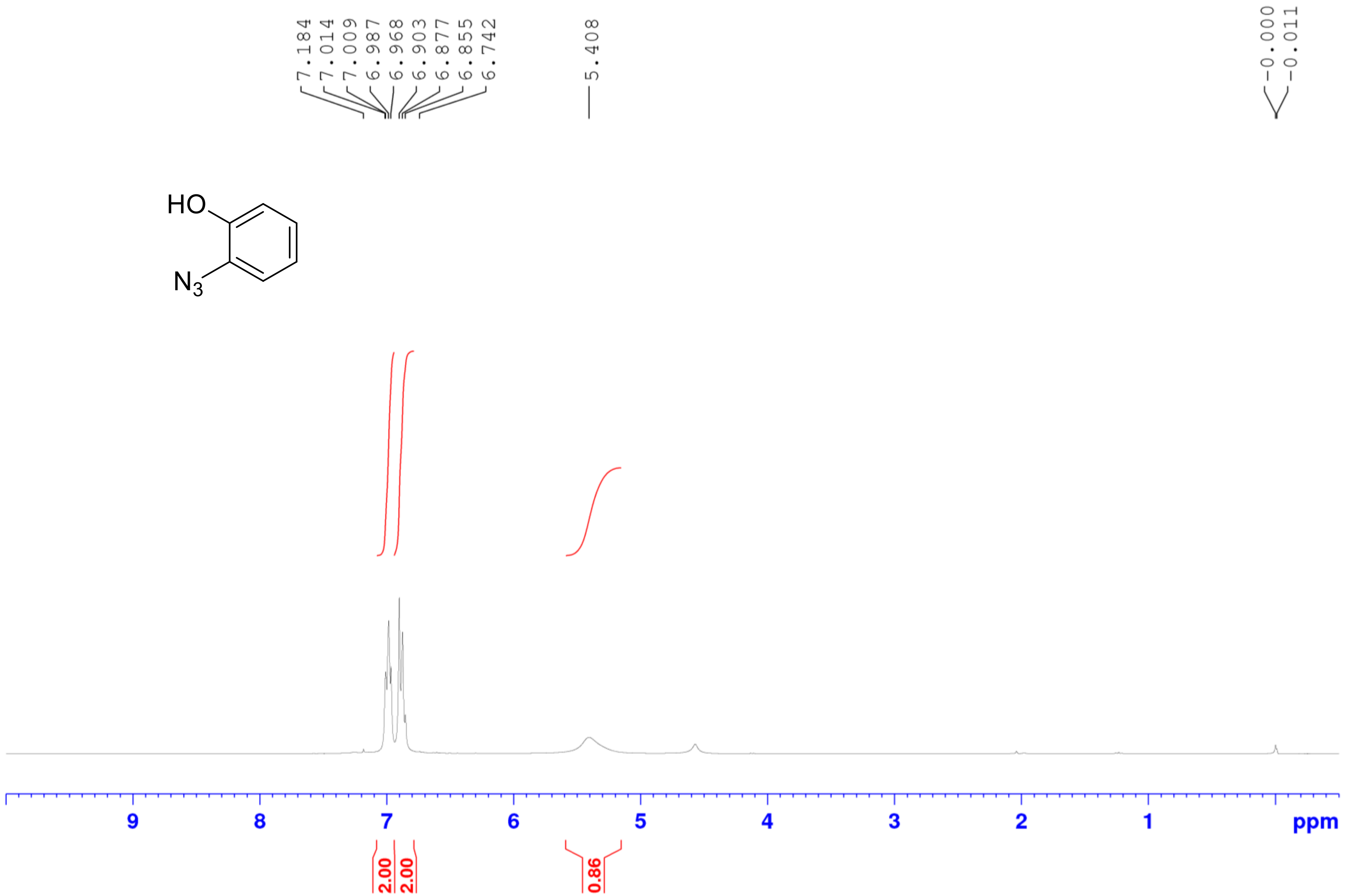

^{13}C NMR of compound **7n** (75 MHz, $CDCl_3$)

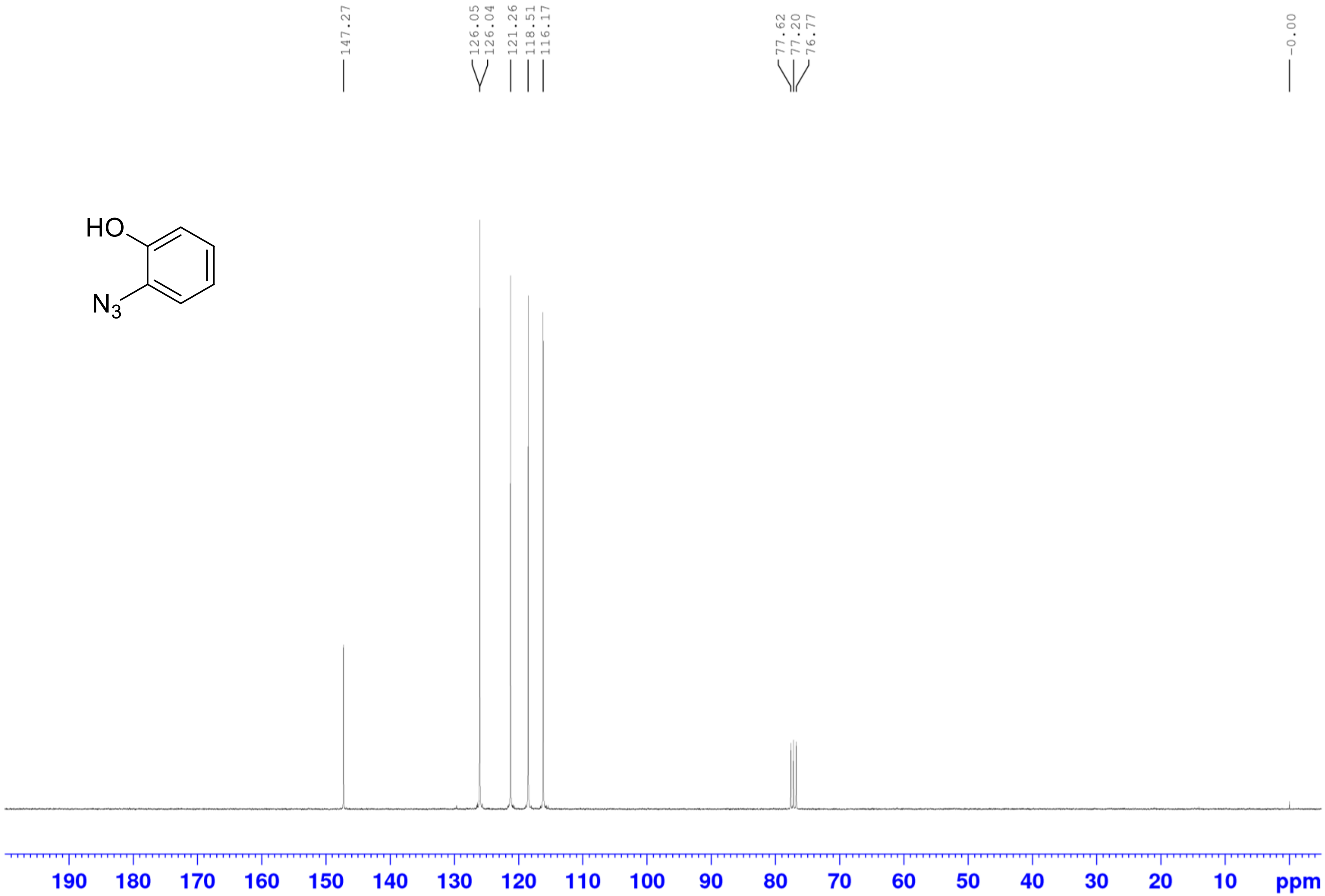

^{1}H NMR of compound **7o** (300 MHz, $CDCl_3$)

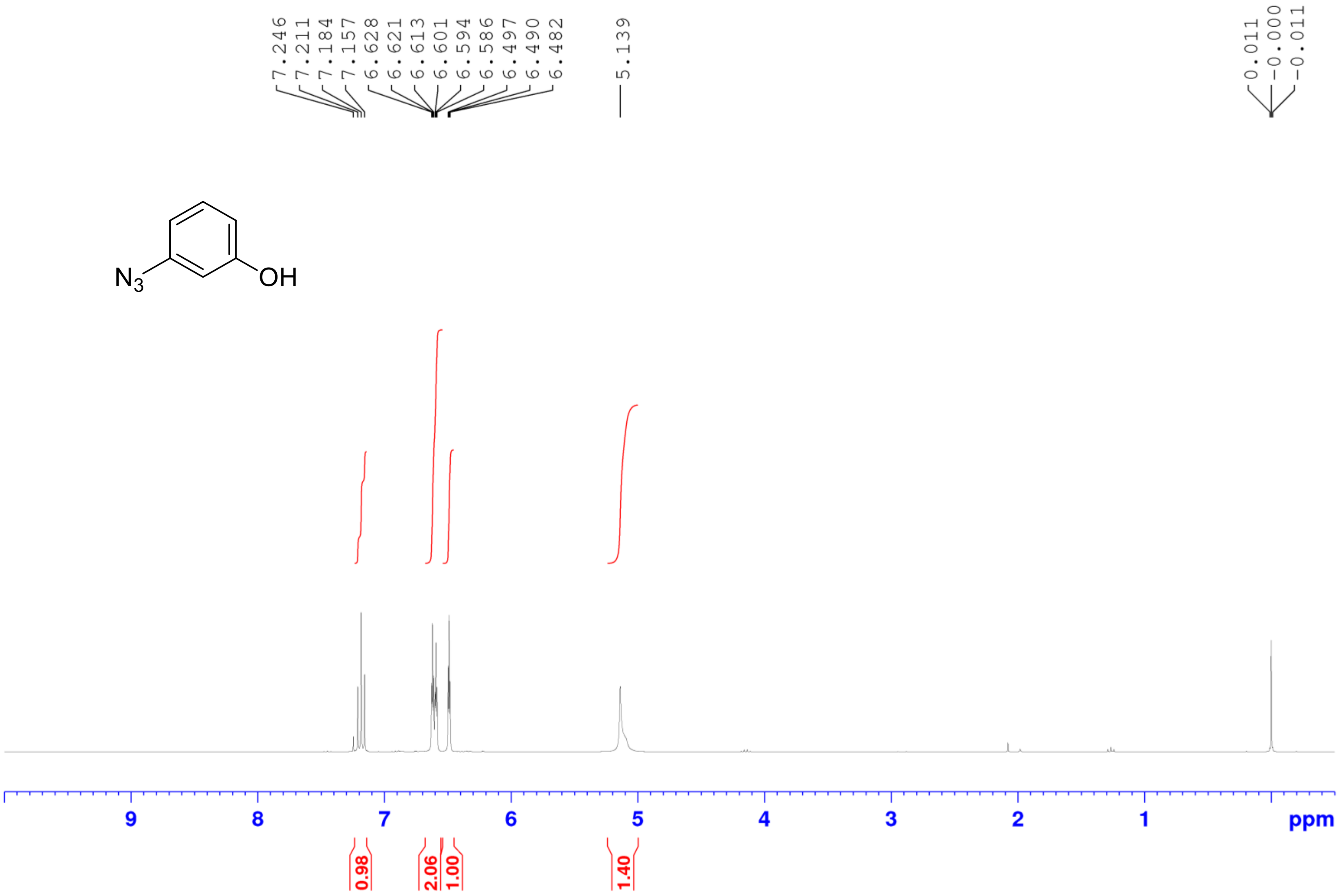

^{13}C NMR of compound **7o** (75 MHz, $CDCl_3$)

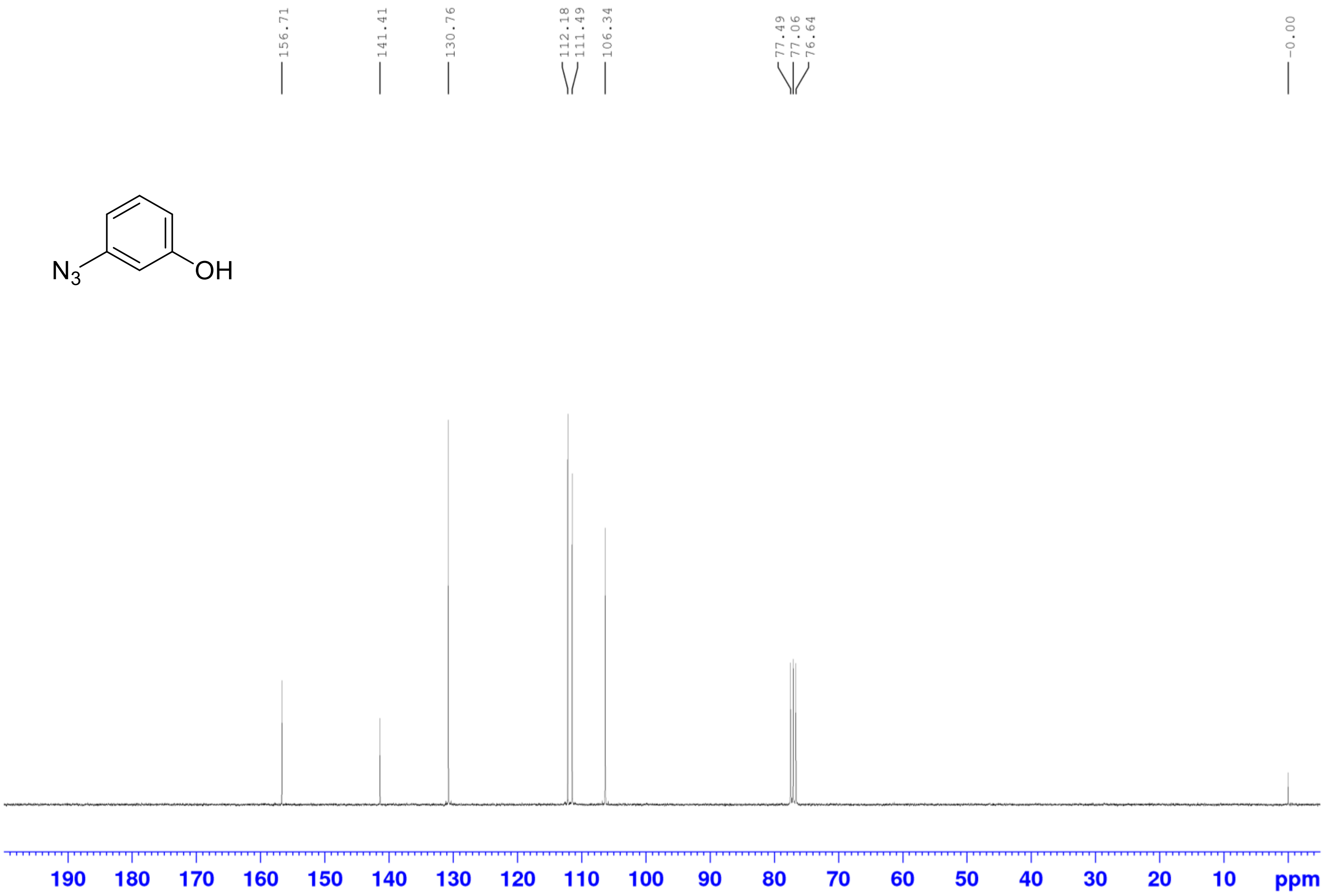

^{1}H NMR of compound **7p** (300 MHz, DMSO-*d6*)

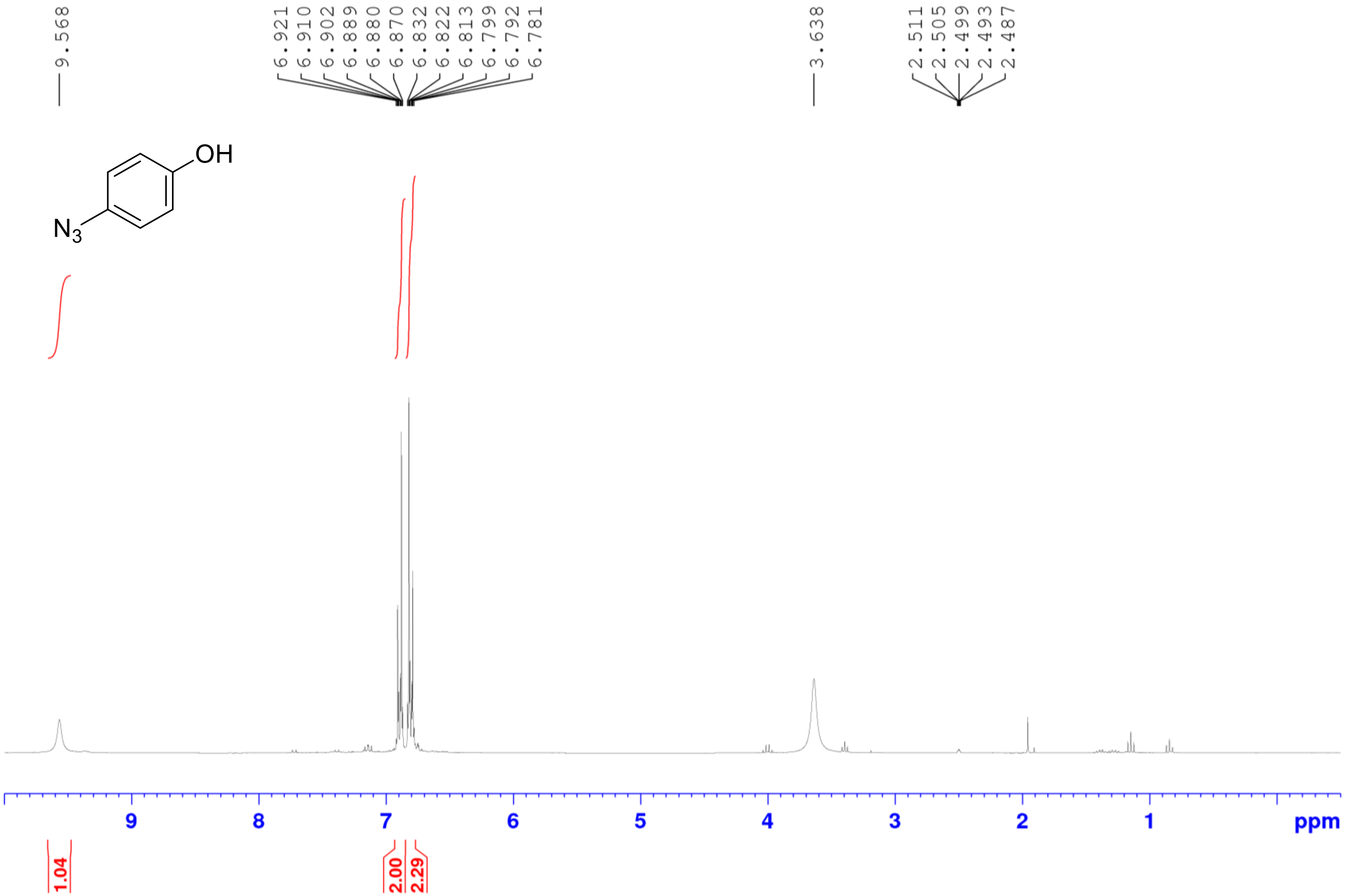

^{13}C NMR of compound **7p** (75 MHz, DMSO-d_6)

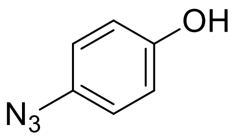

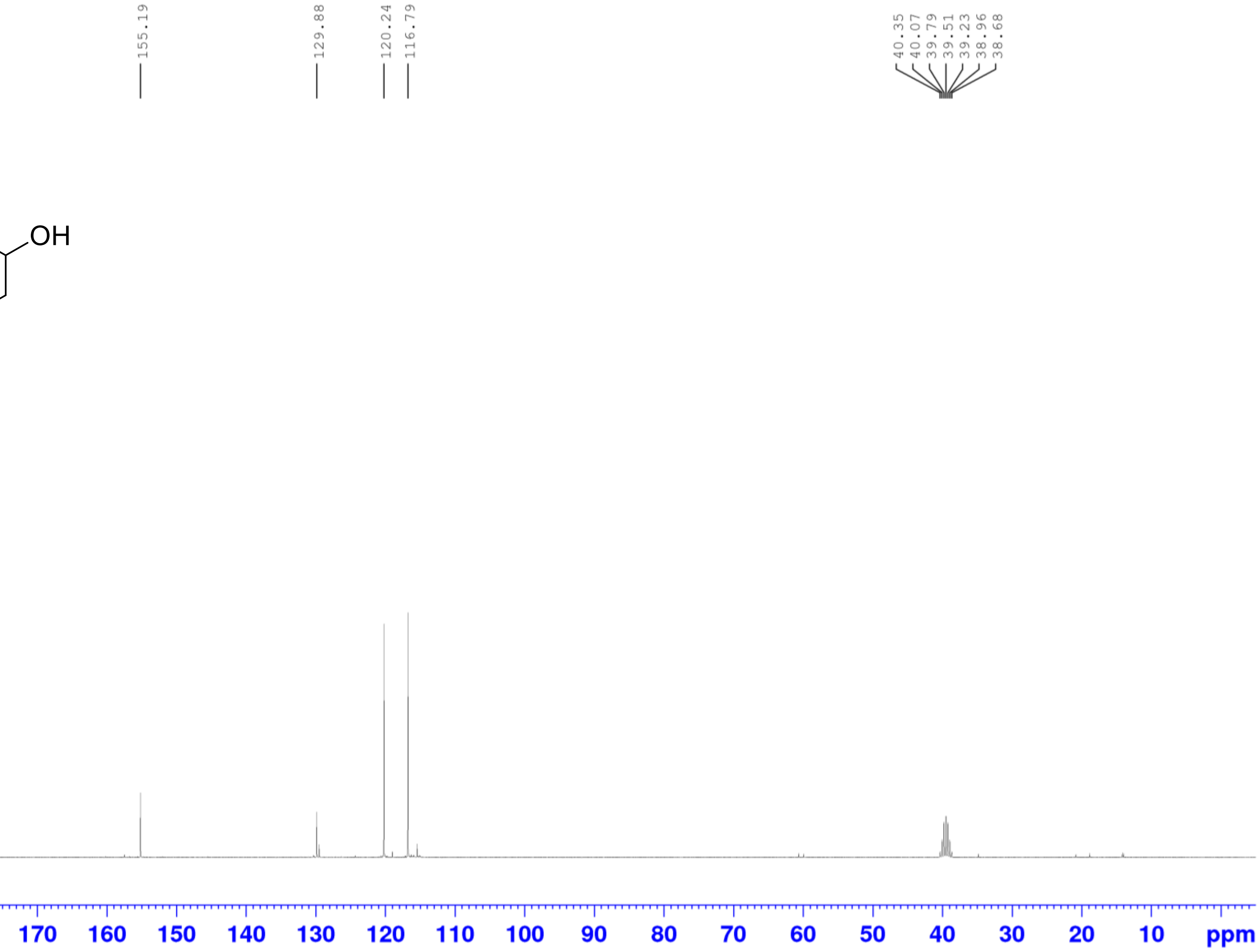

^{1}H NMR of compound **7q** (300 MHz, $CDCl_3$)

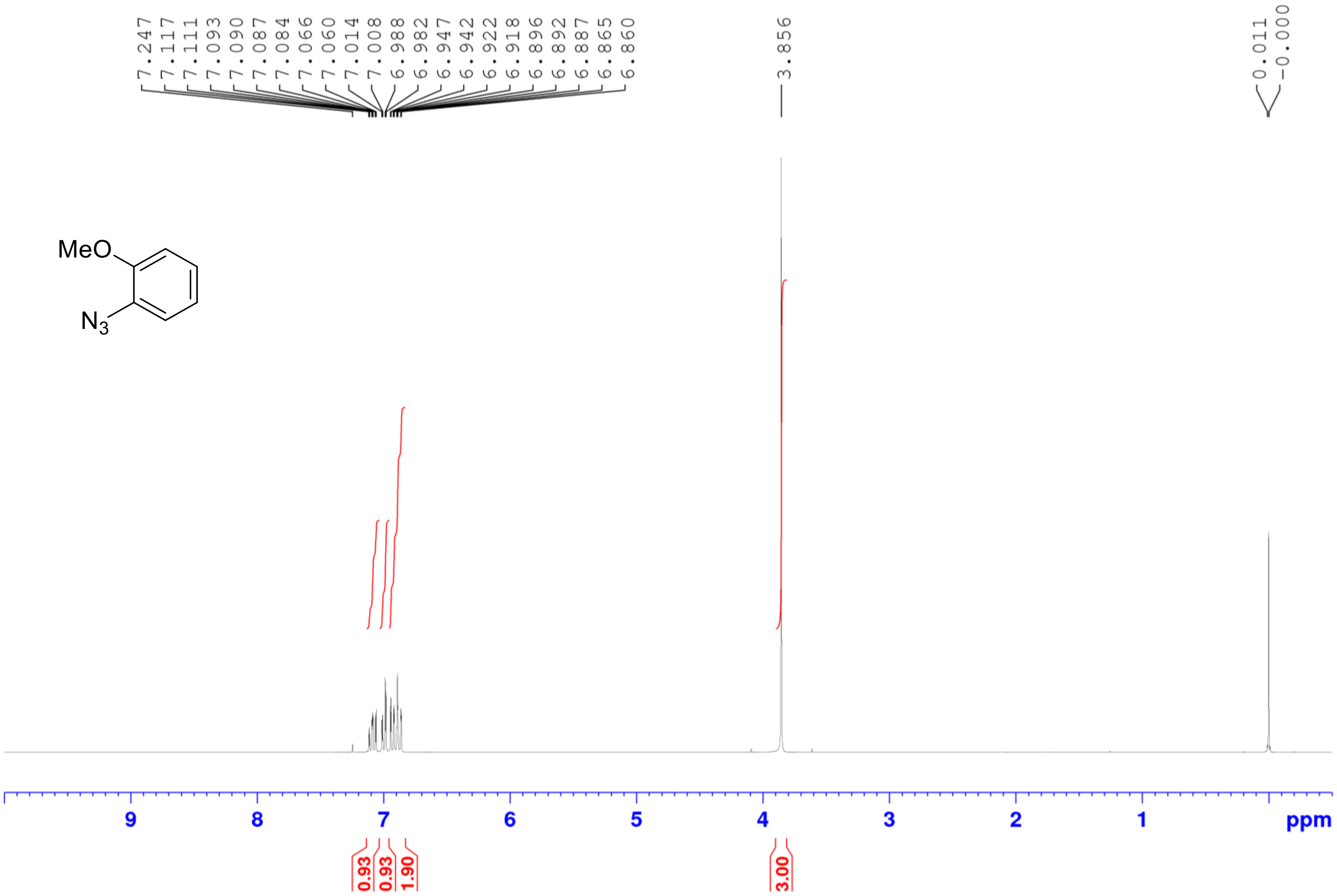

^{13}C NMR of compound **7q** (75 MHz, $CDCl_3$)

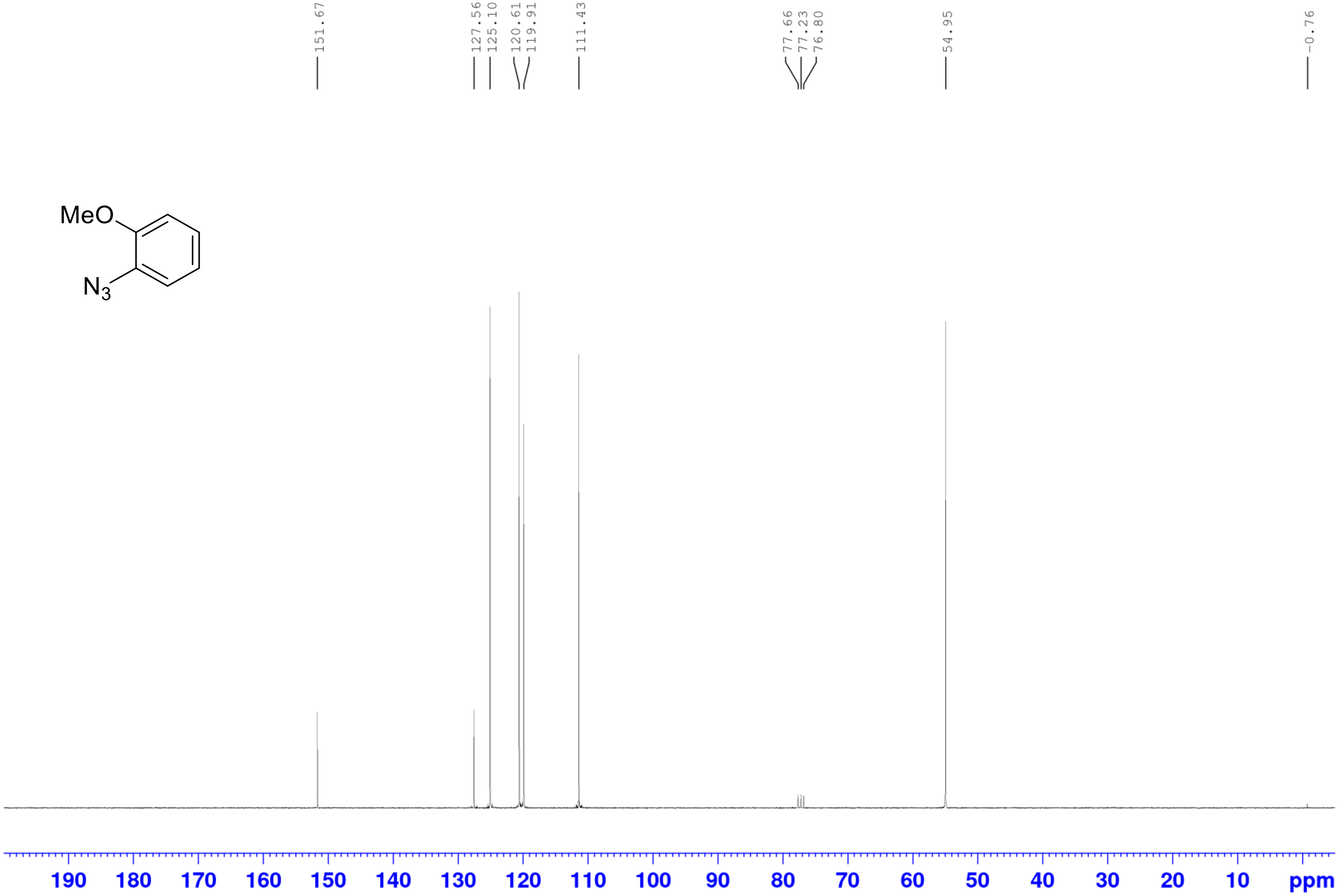

^{1}H NMR of compound **7r** (300 MHz, $CDCl_3$)

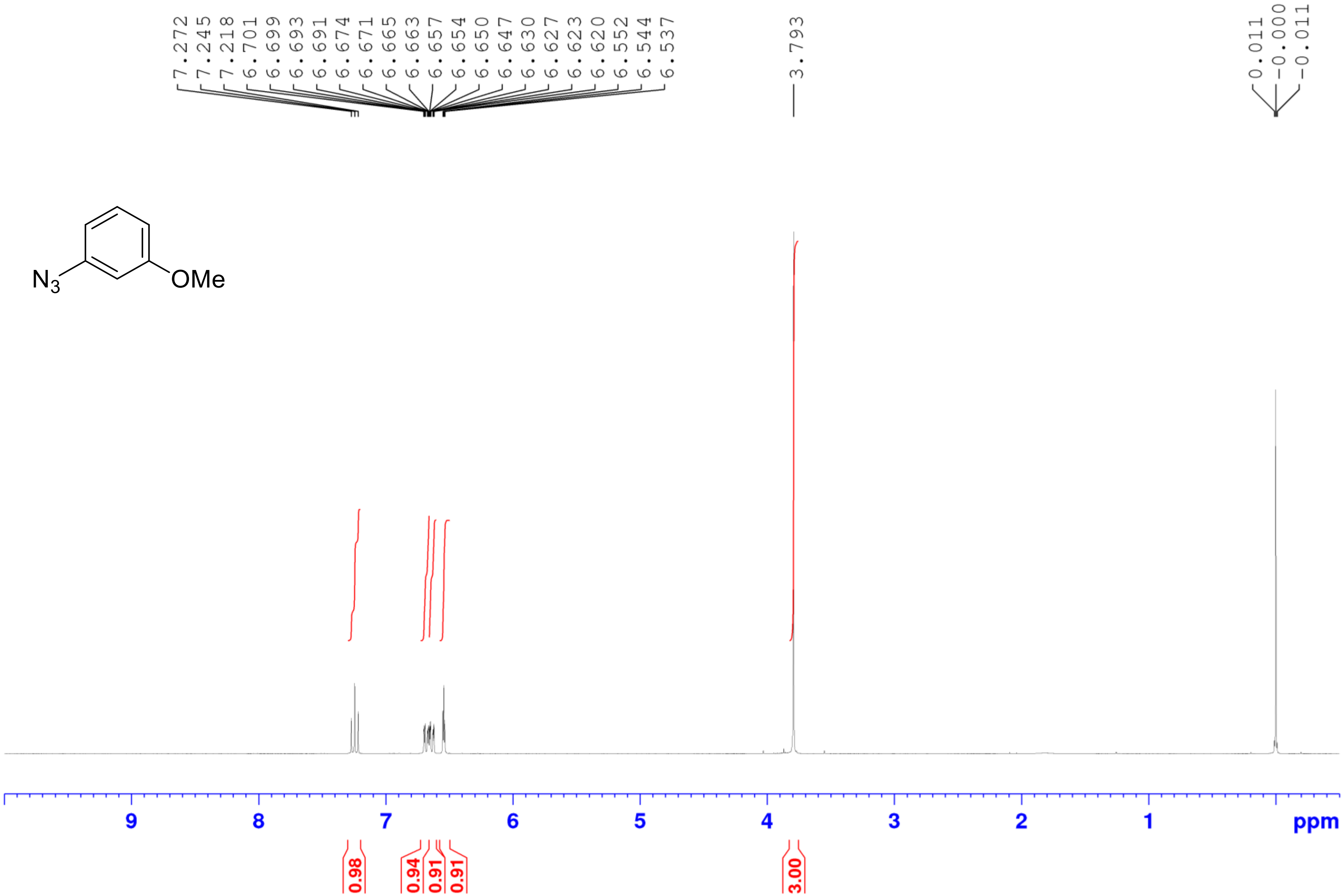

^{13}C NMR of compound **7r** (75 MHz, $CDCl_3$)

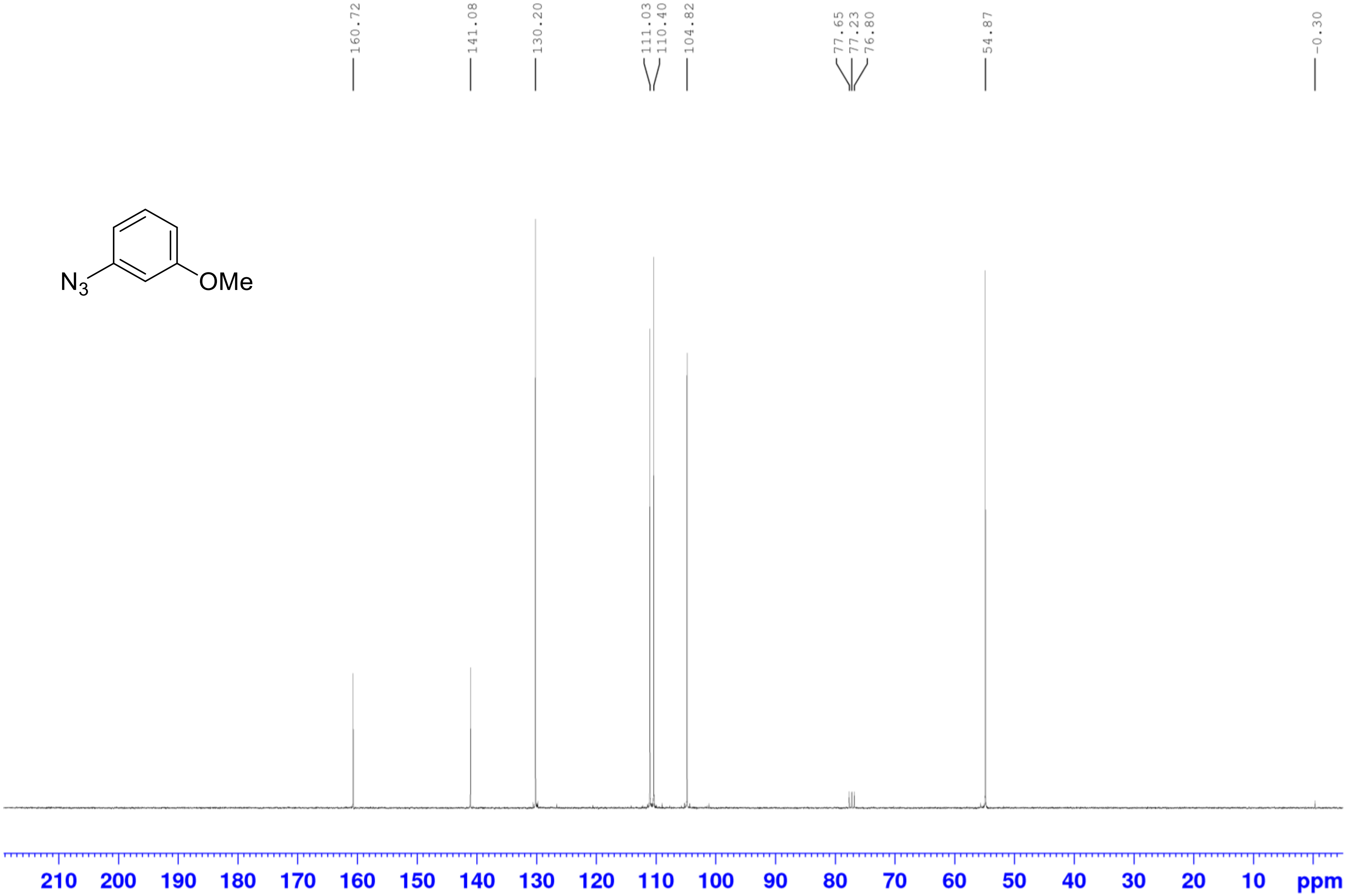

^{1}H NMR of compound **7s** (300 MHz, $CDCl_3$)

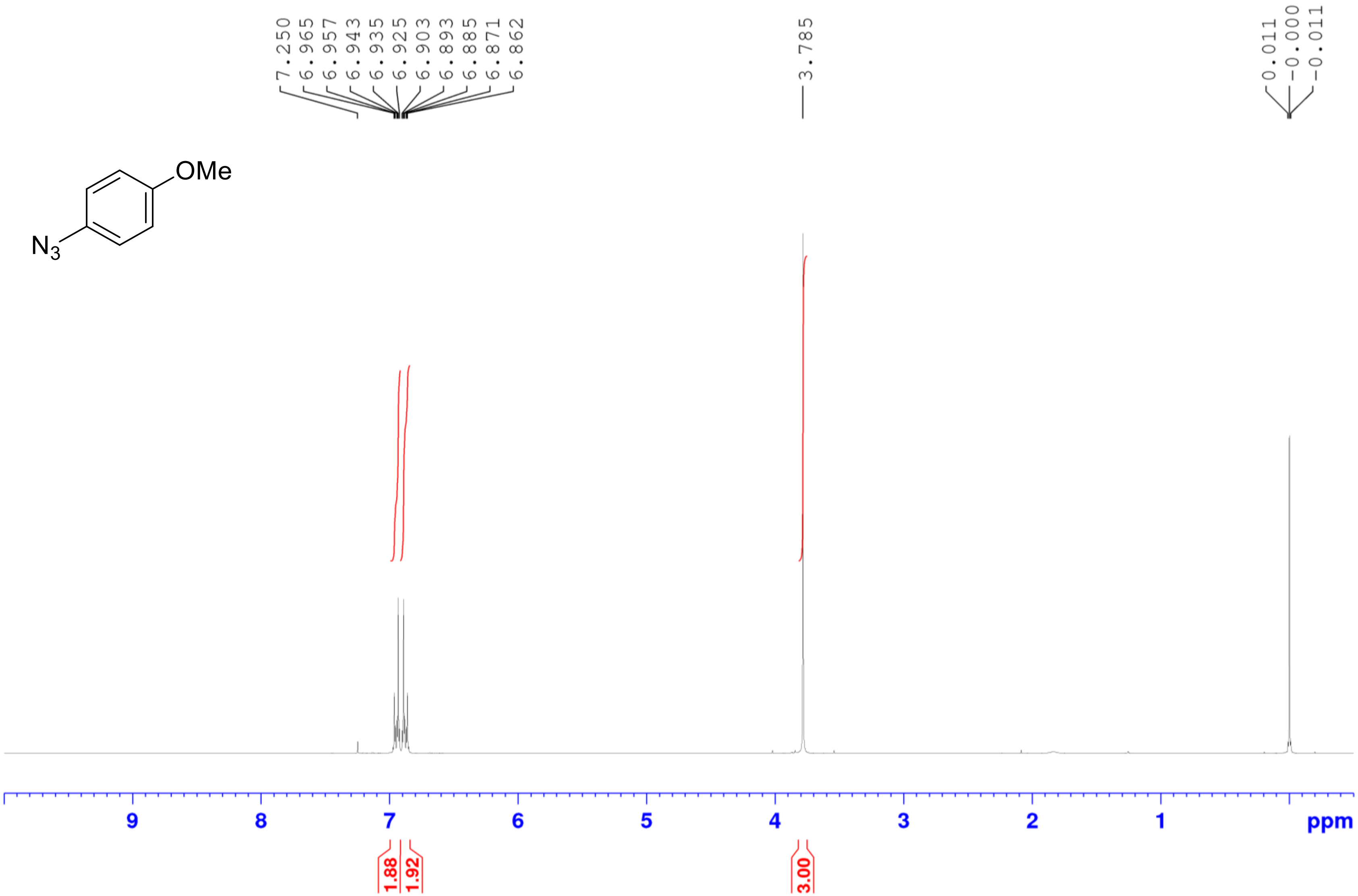

^{13}C NMR of compound **7s** (75 MHz, $CDCl_3$)

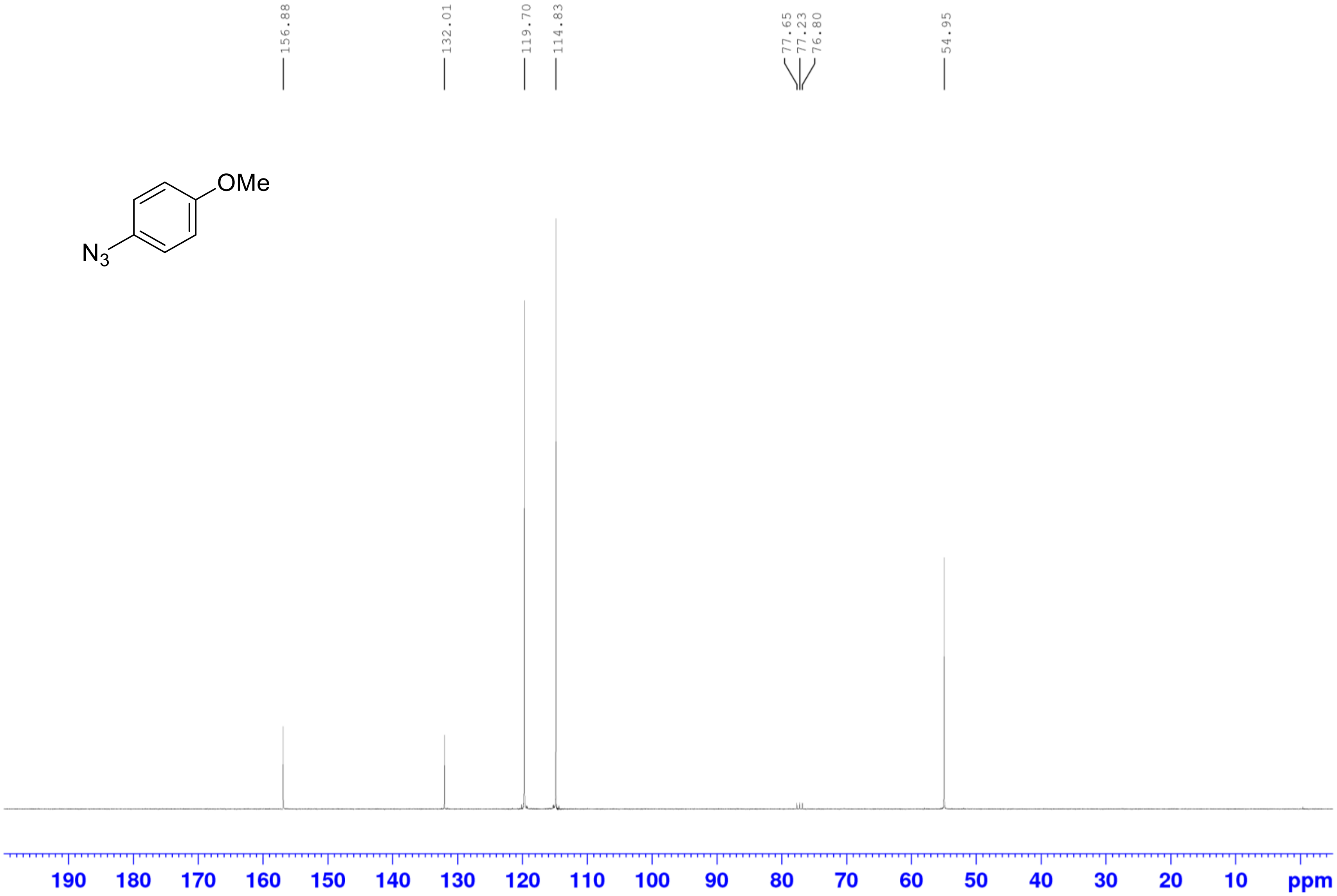

^{1}H NMR of compound **7t** (300 MHz, $CDCl_3$)

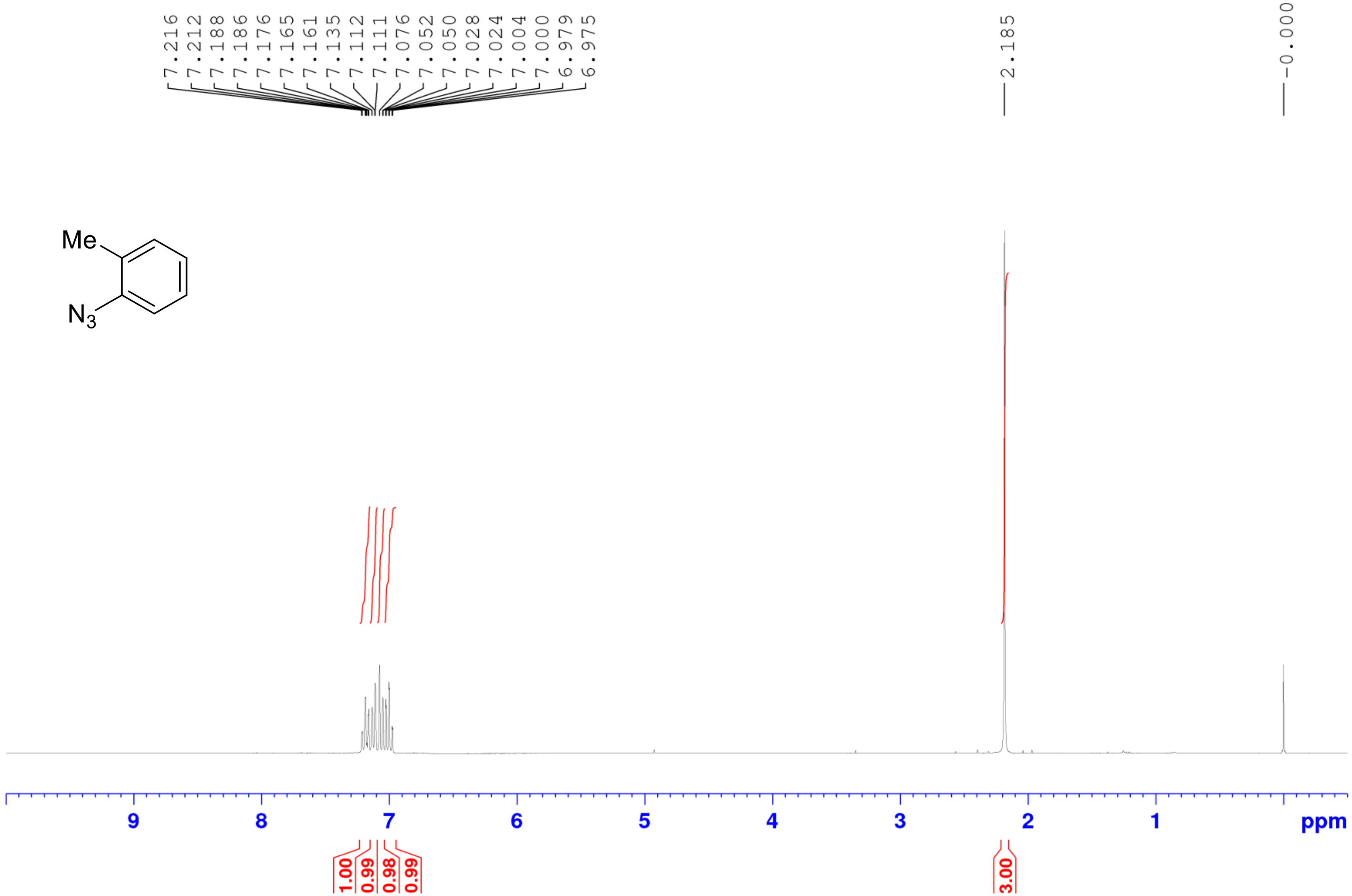

^{13}C NMR of compound **7t** (75 MHz, $CDCl_3$)

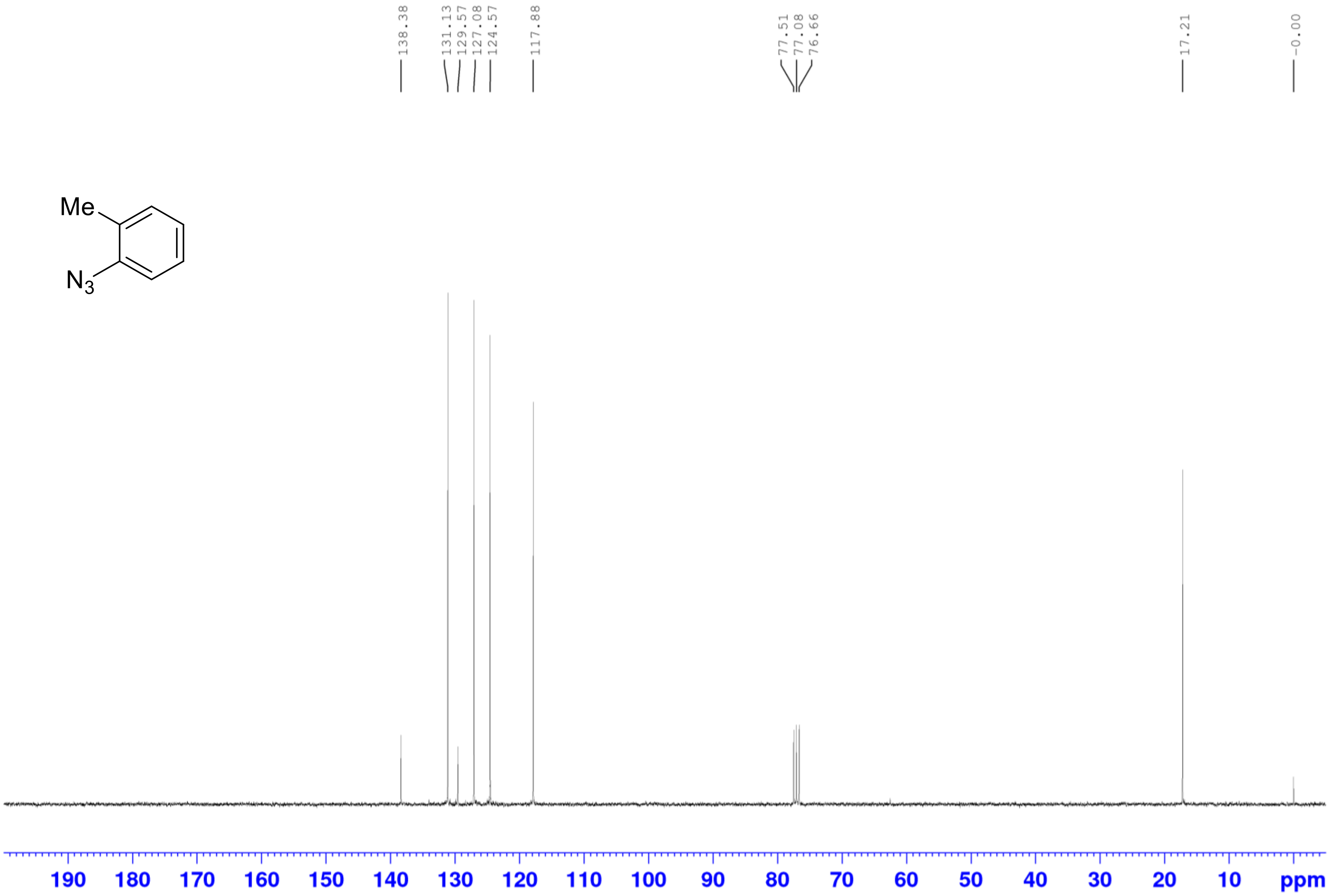

^{1}H NMR of compound **7u** (300 MHz, $CDCl_3$)

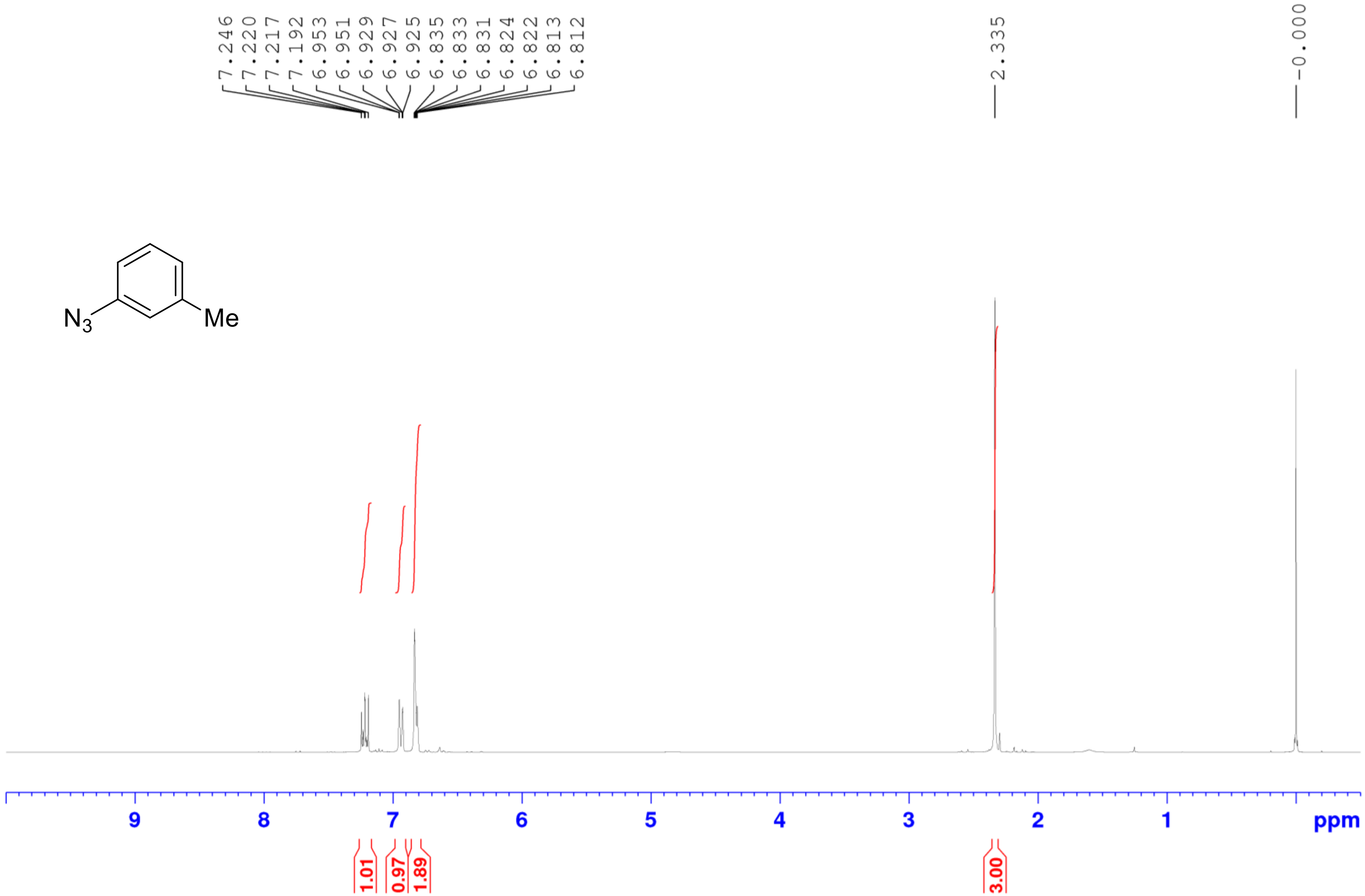

^{13}C NMR of compound **7u** (75 MHz, $CDCl_3$)

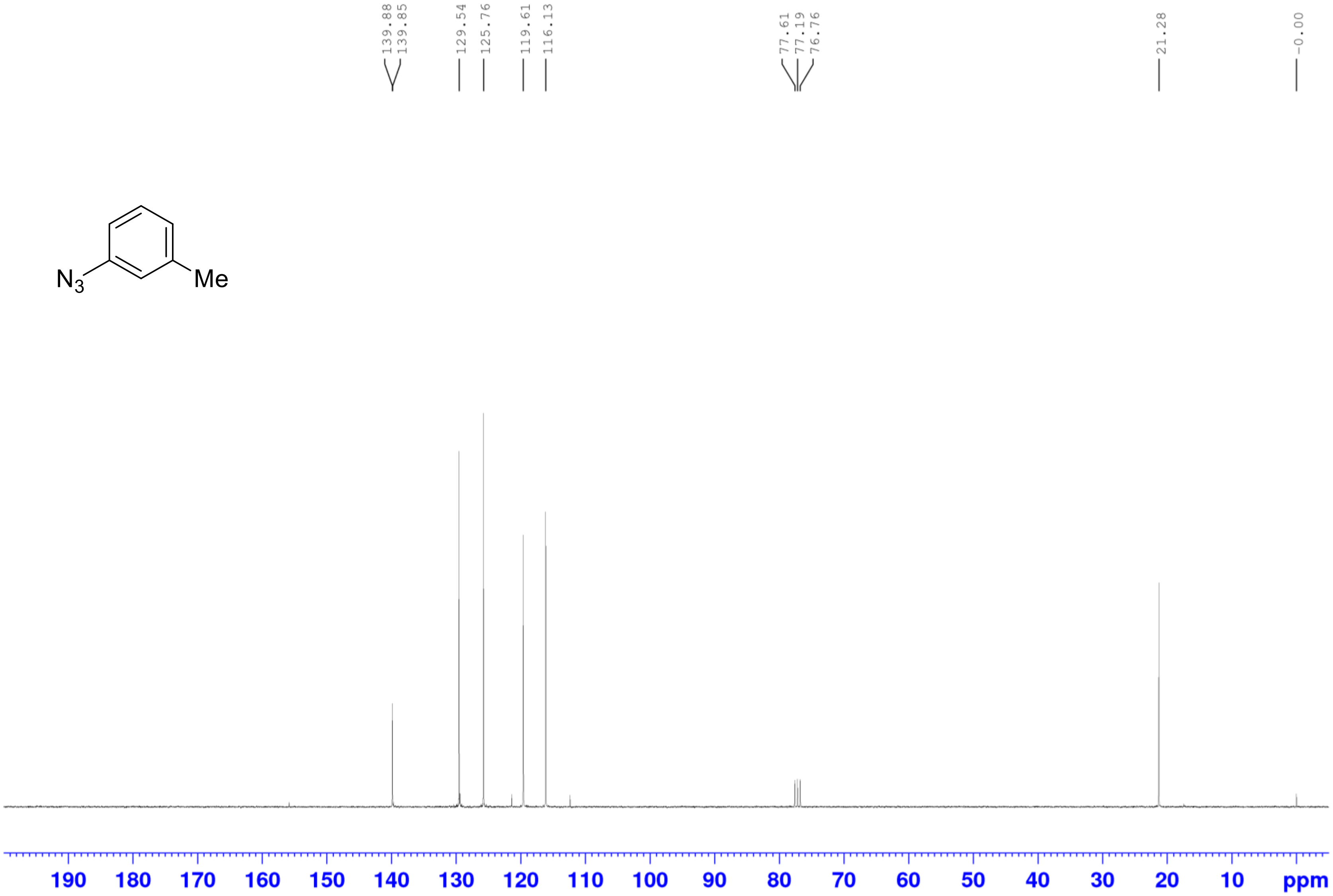

^{1}H NMR of compound **7v** (300 MHz, $CDCl_3$)

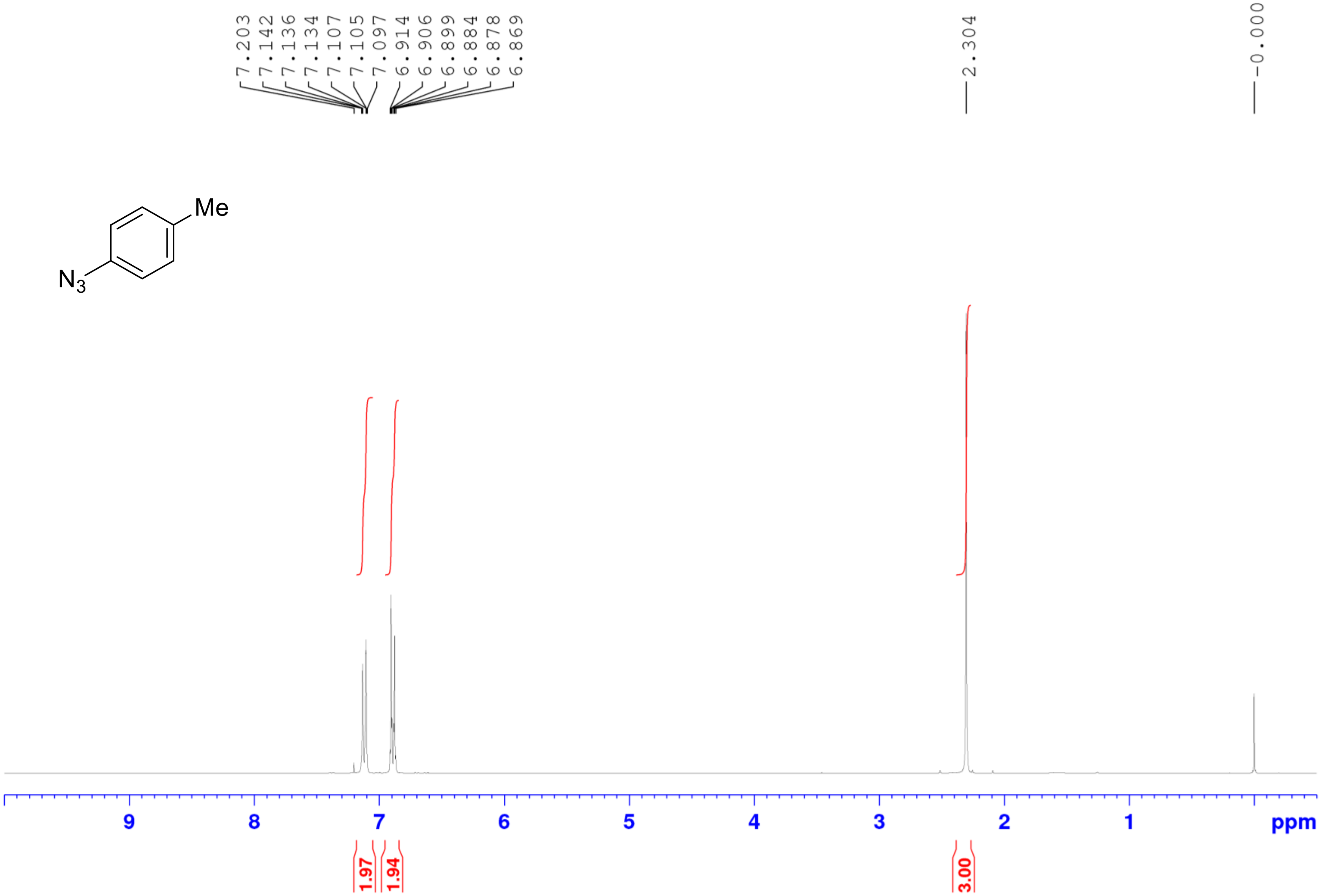

^{13}C NMR of compound **7v** (75 MHz, $CDCl_3$)

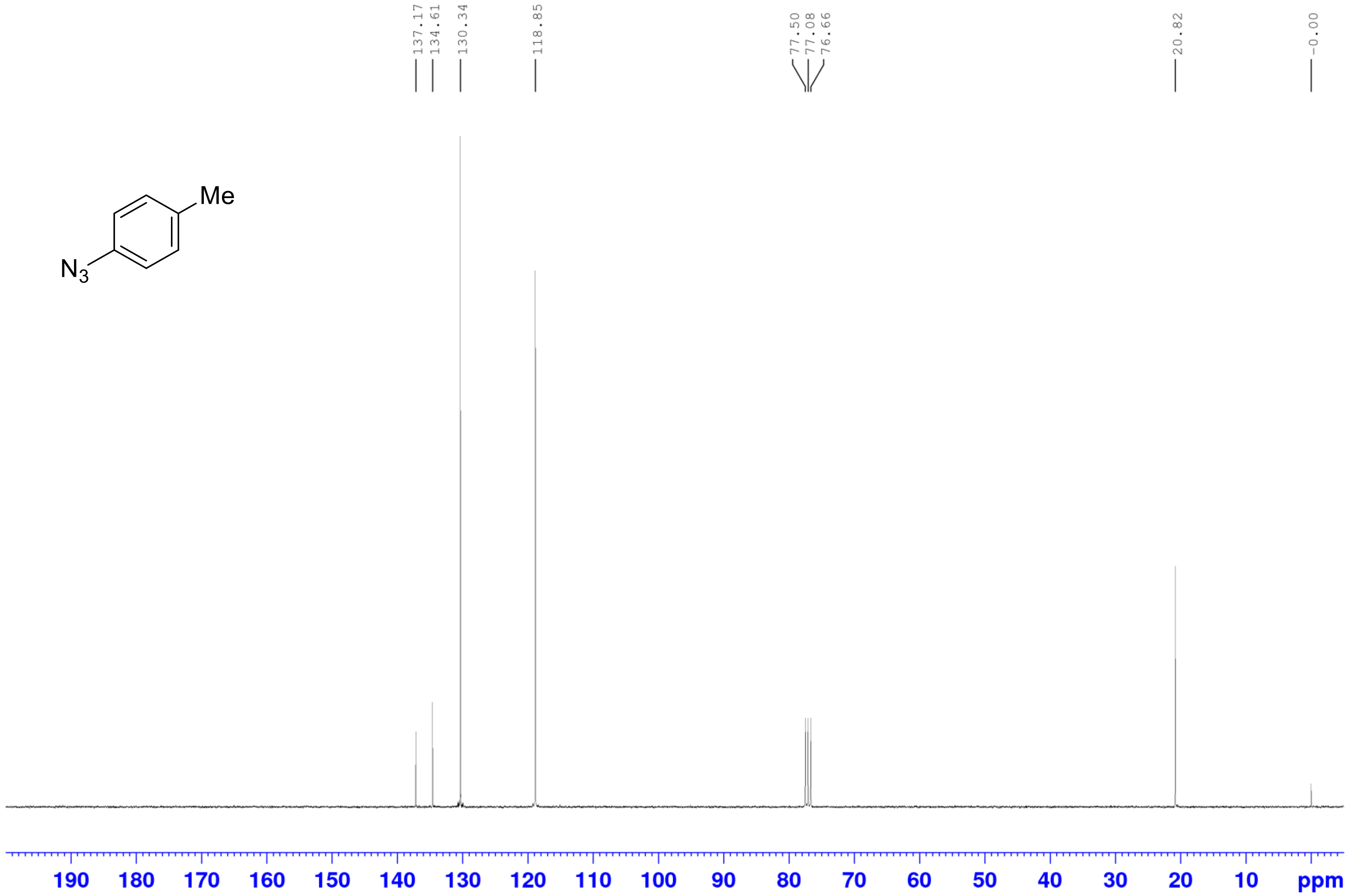

^{1}H NMR of compound **7w** (300 MHz, DMSO-d_6)

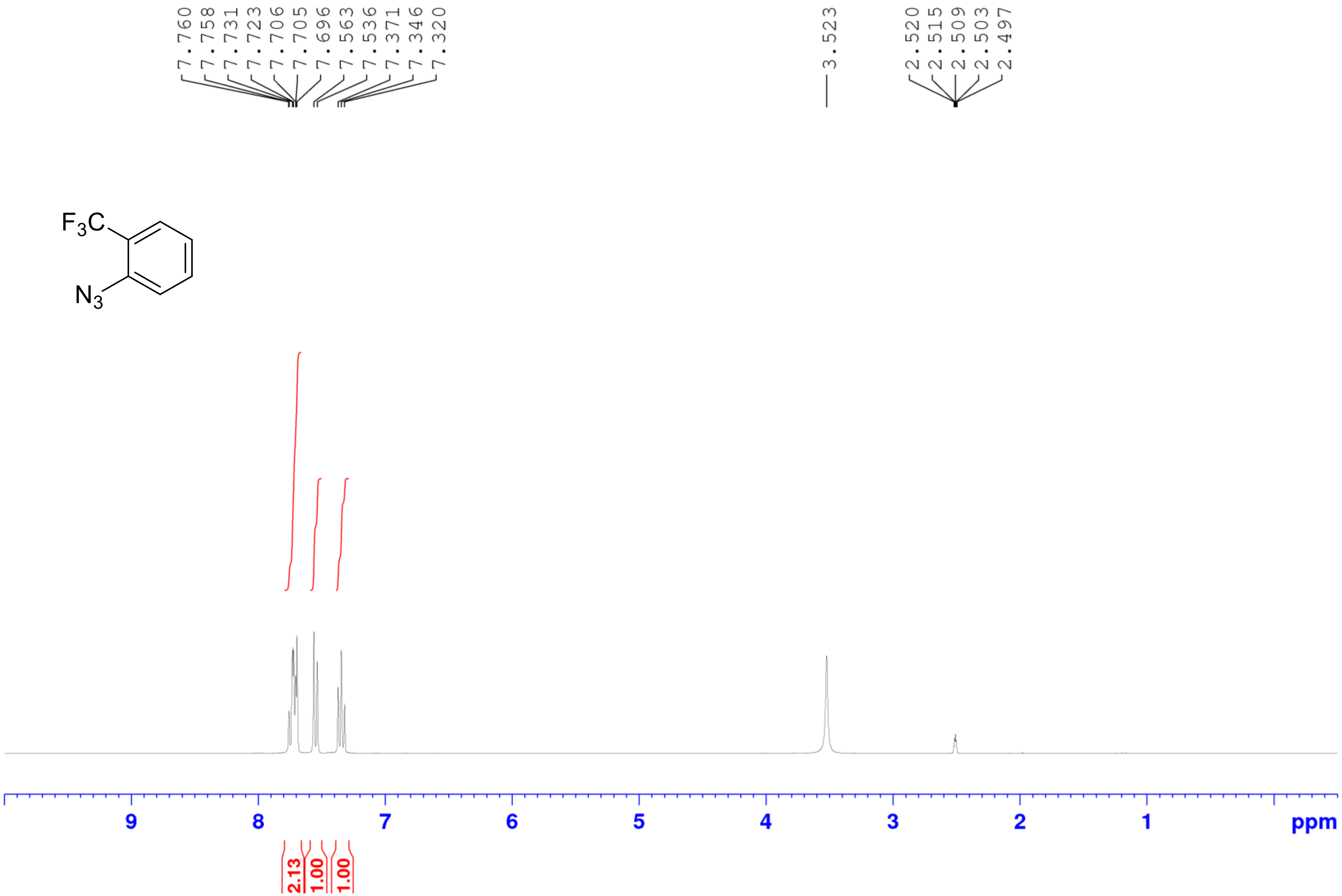

^{13}C NMR of compound **7w** (75 MHz, DMSO-d_6)

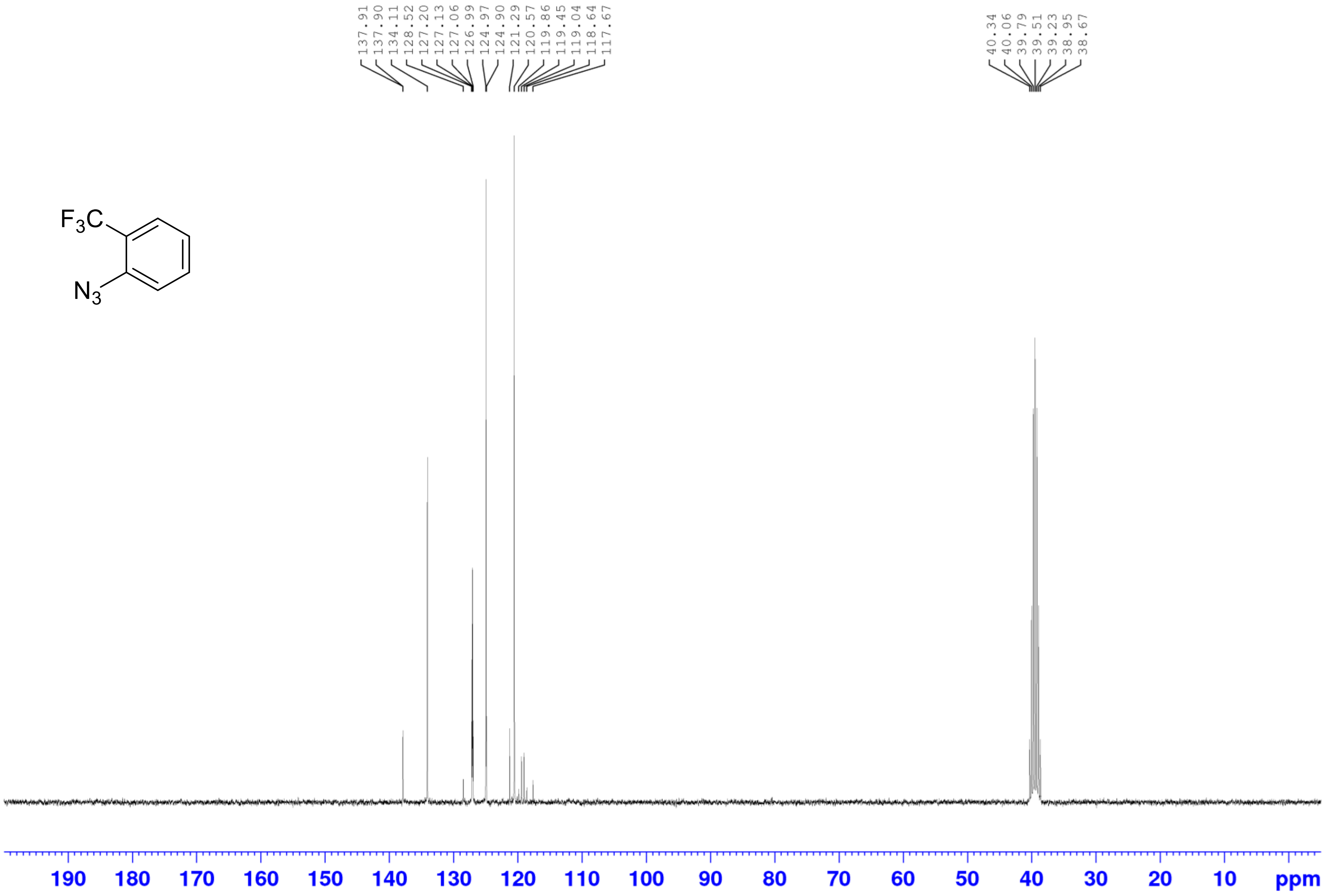

^{19}F NMR of compound **7w** (282 MHz, DMSO-*d*$_6$)

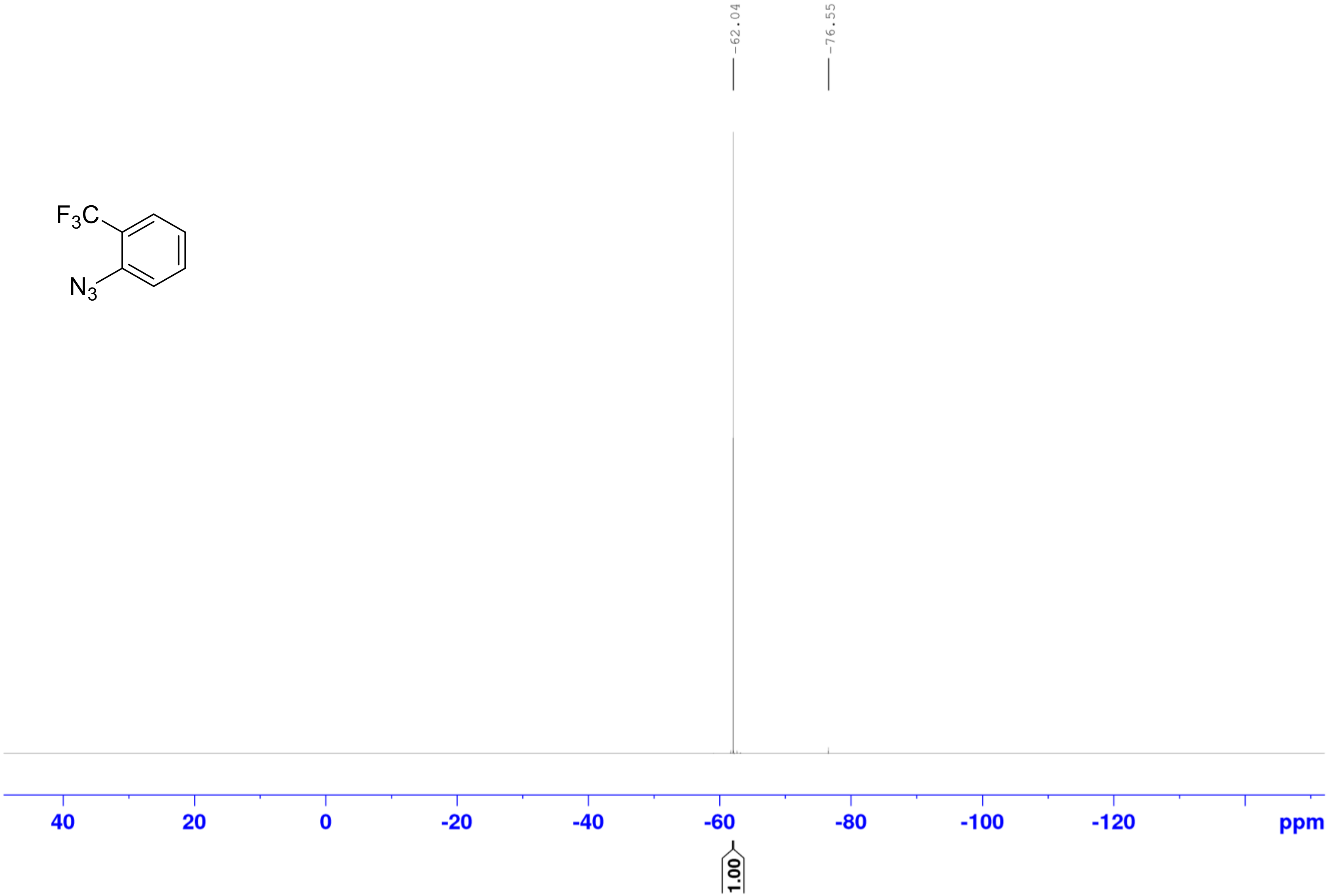

^{1}H NMR of compound **7x** (300 MHz, DMSO-d_6)

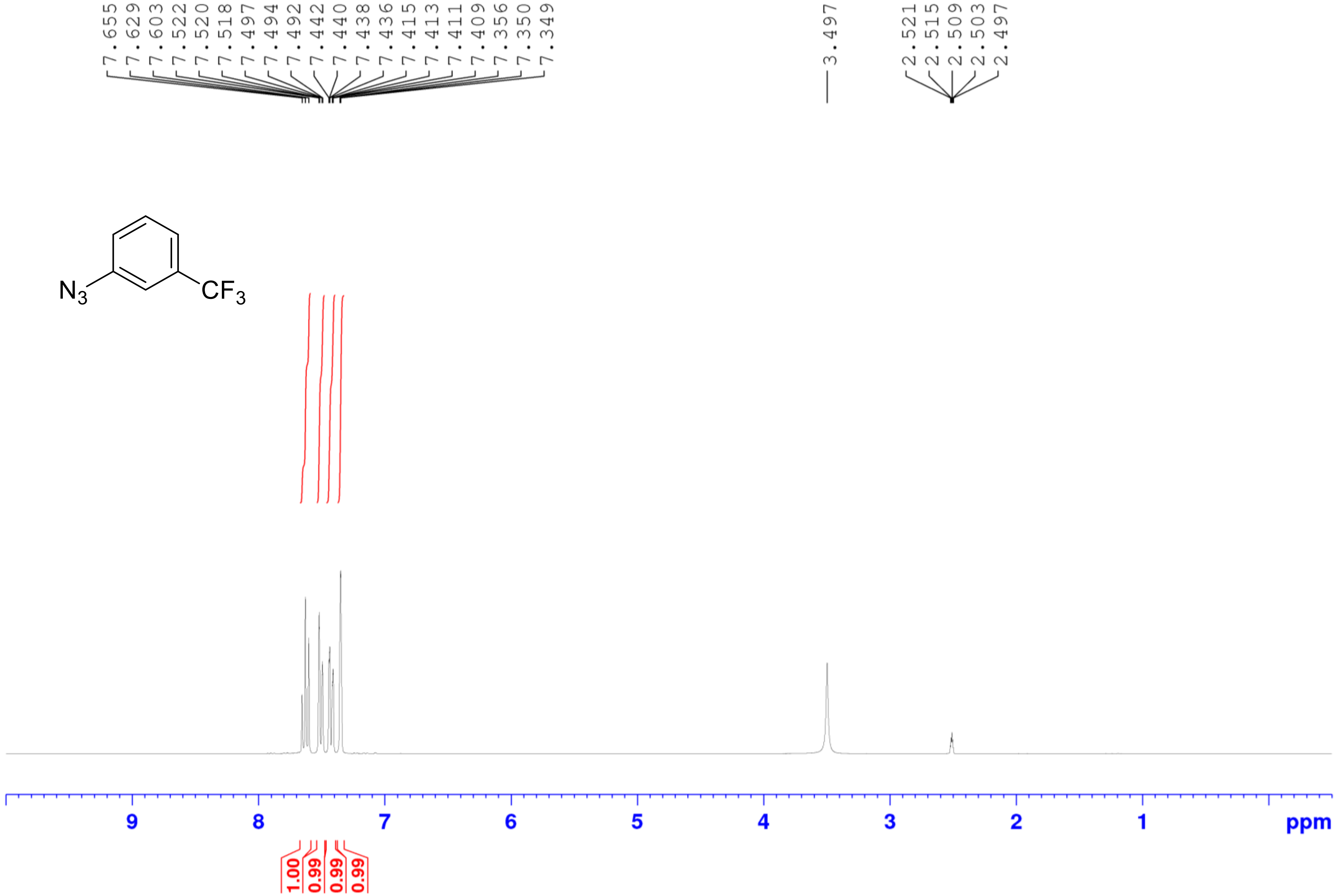

^{13}C NMR of compound **7x** (75 MHz, DMSO-d_6)

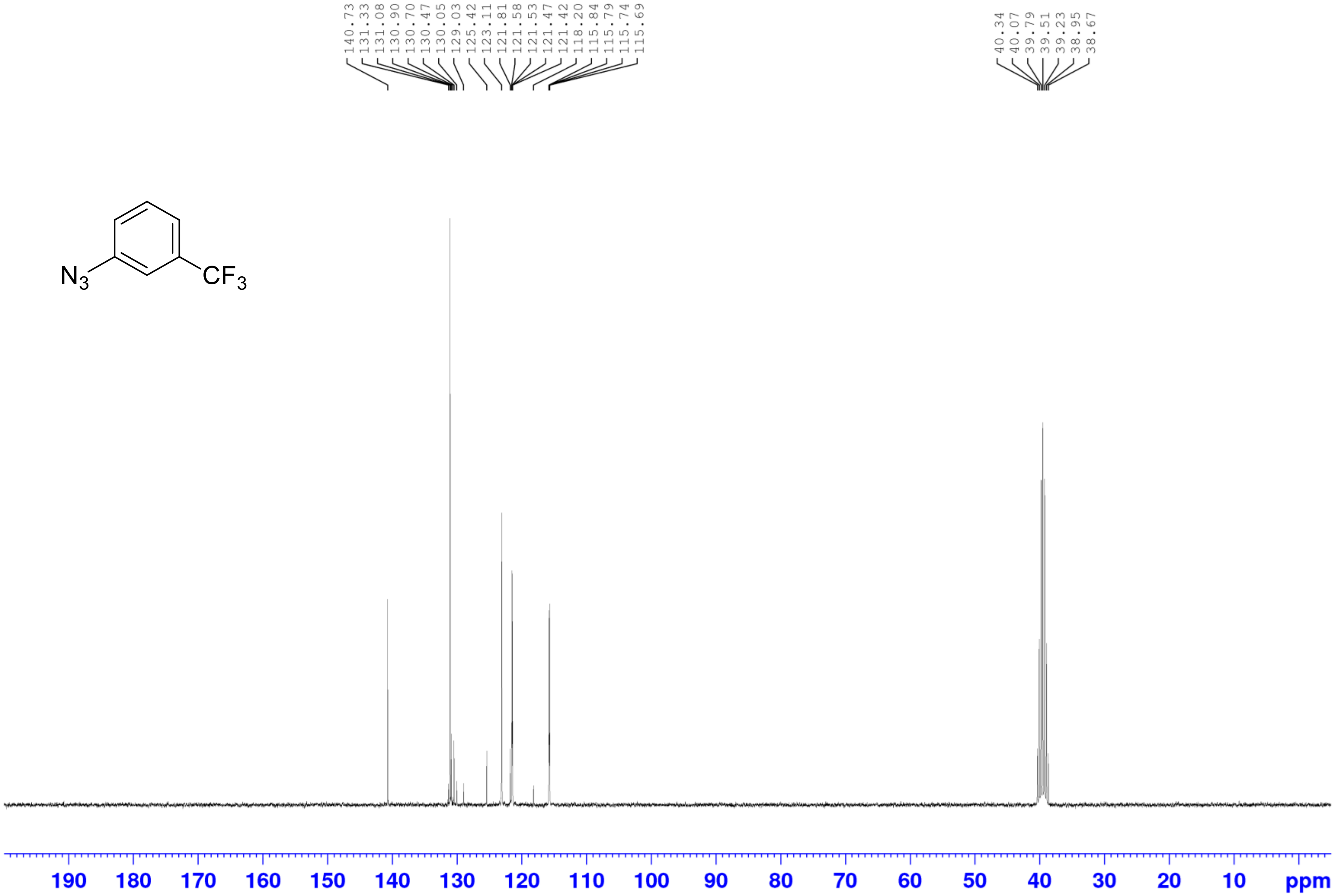

^{19}F NMR of compound **7x** (282 MHz, DMSO-d_6)

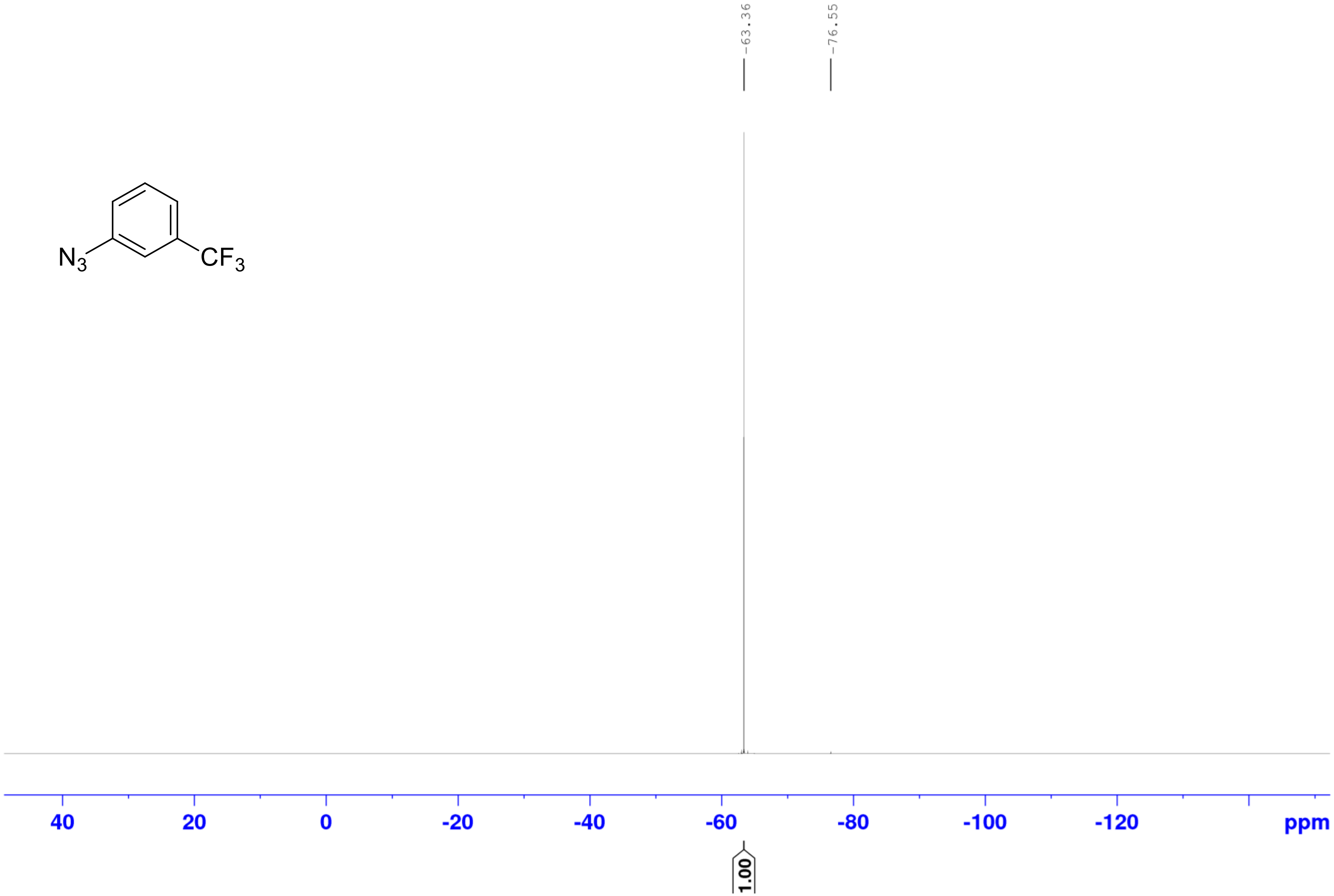

^{1}H NMR of compound **7y** (300 MHz, DMSO-*d6*)

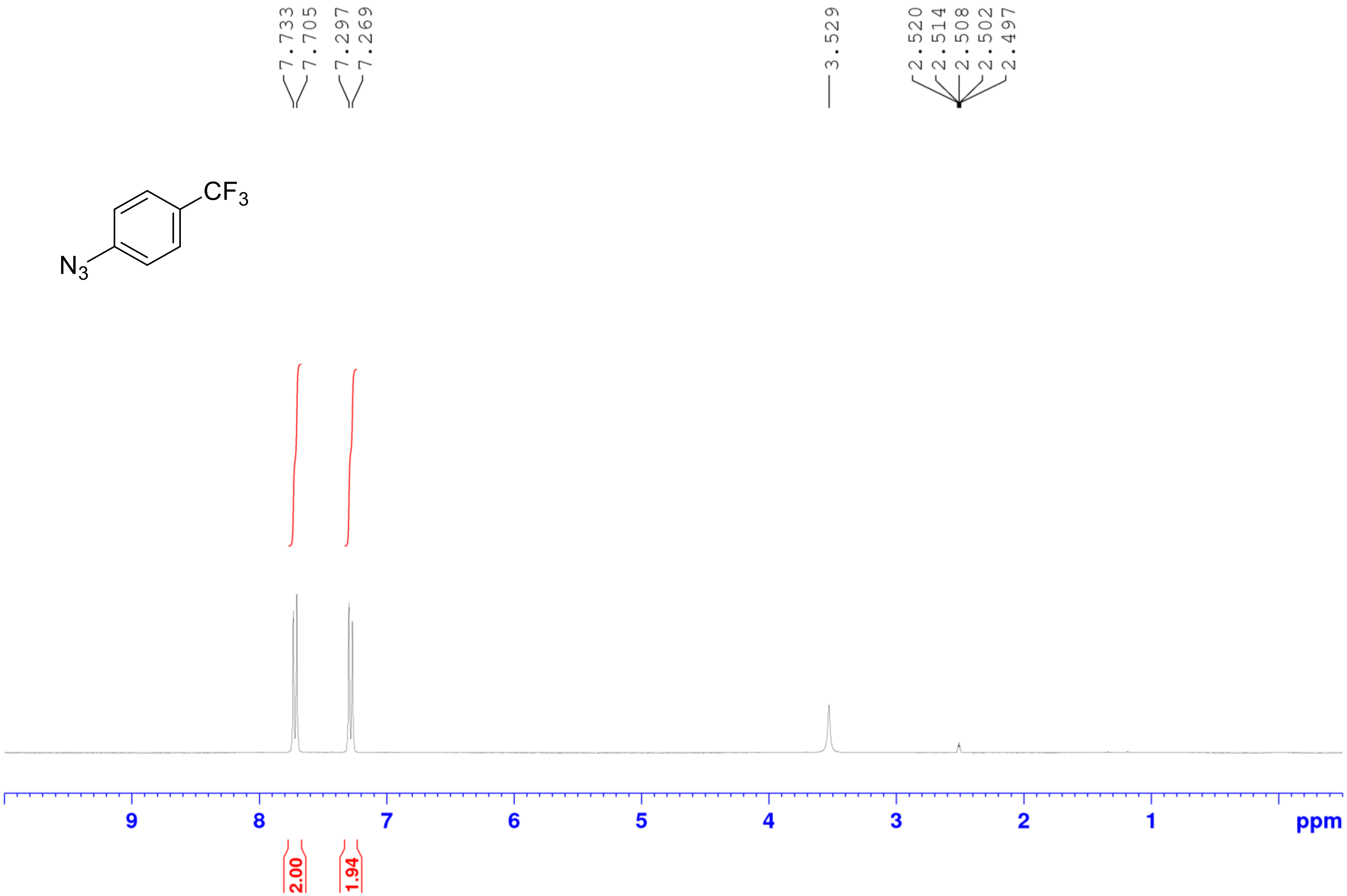

^{13}C NMR of compound **7y** (75 MHz, DMSO-d_6)

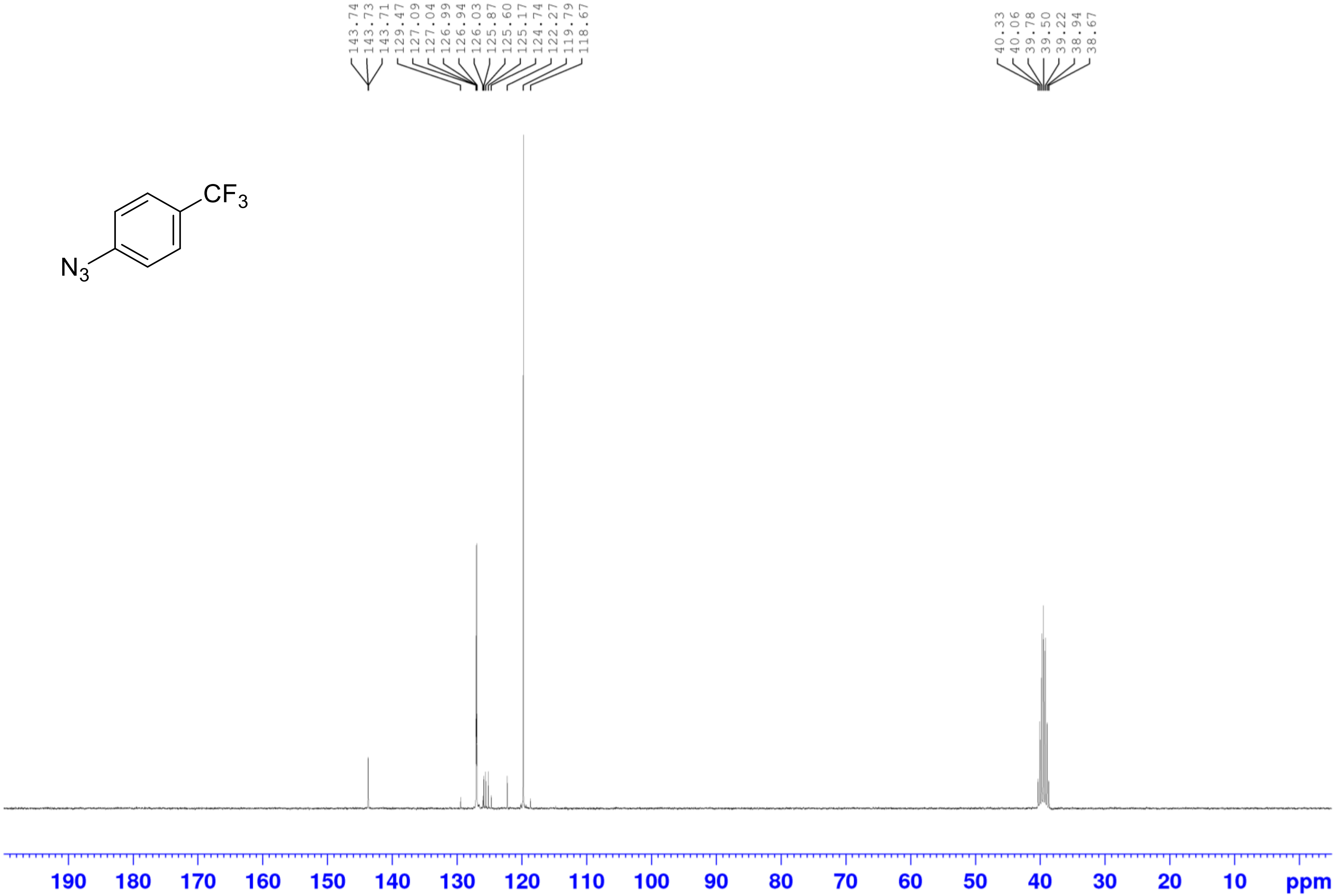

^{19}F NMR of compound **7y** (282 MHz, DMSO-d_6)

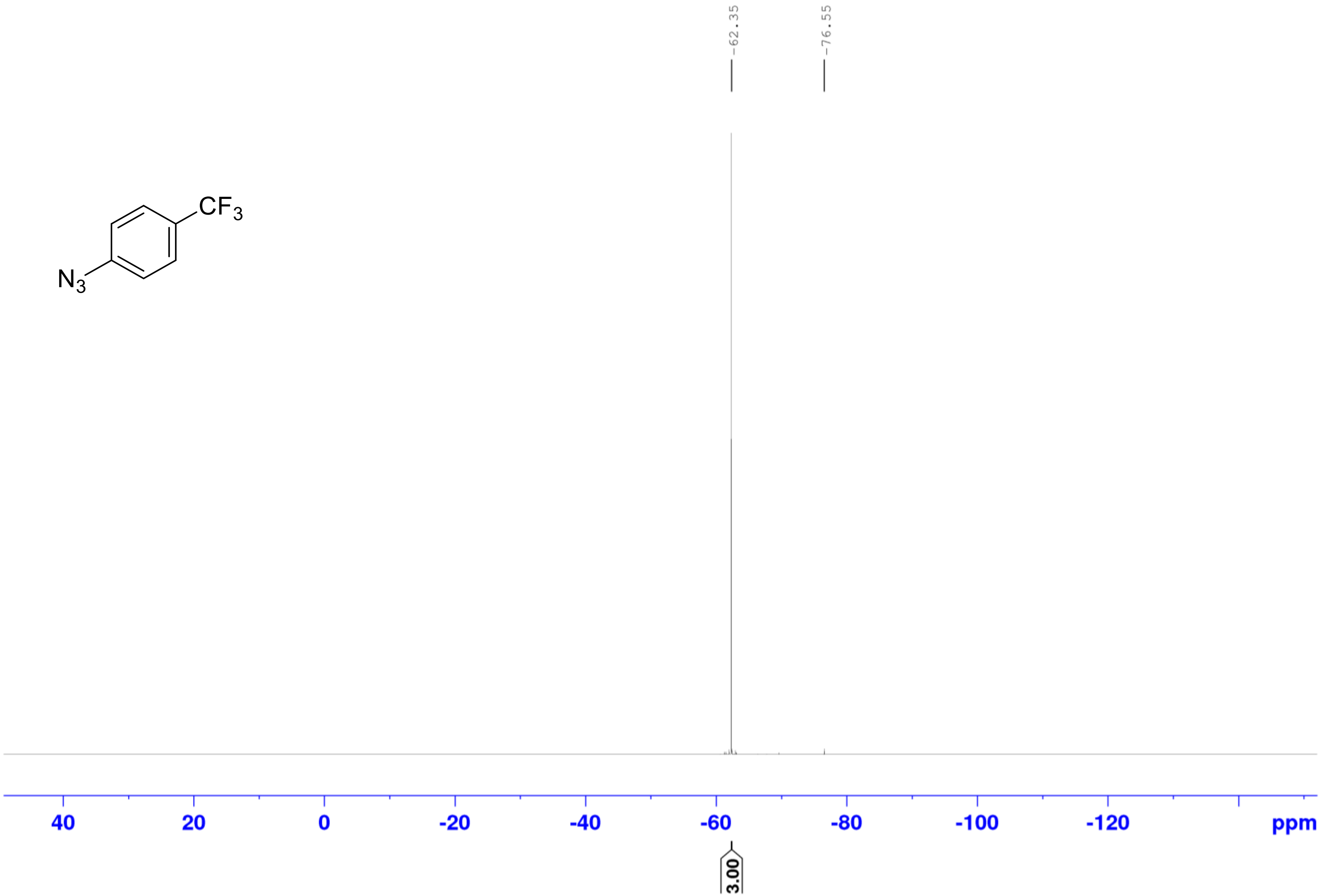

^{1}H NMR of compound **7z** (300 MHz, $CDCl_3$)

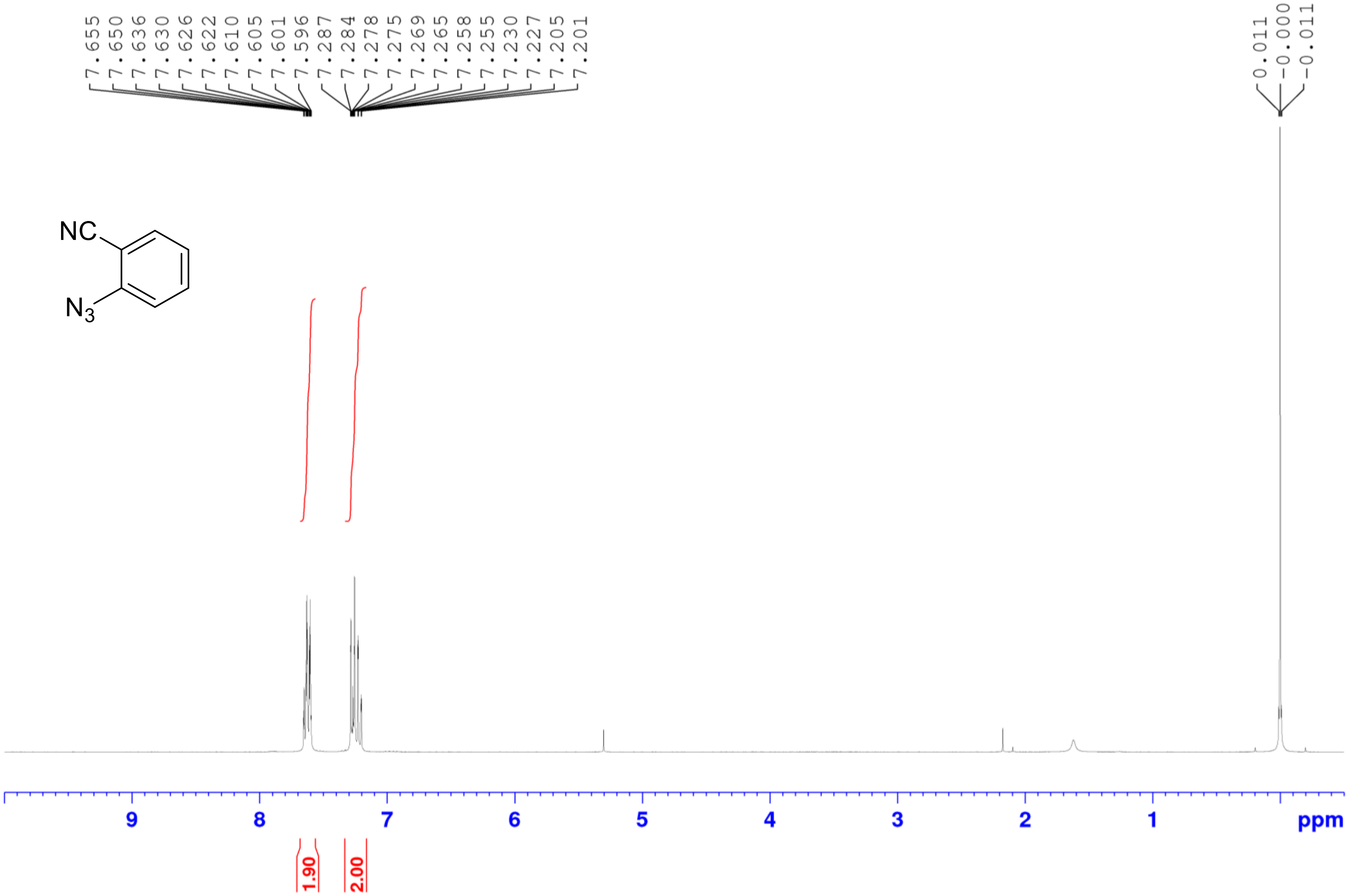

^{13}C NMR of compound **7z** (75 MHz, $CDCl_3$)

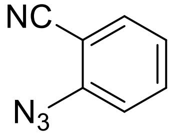

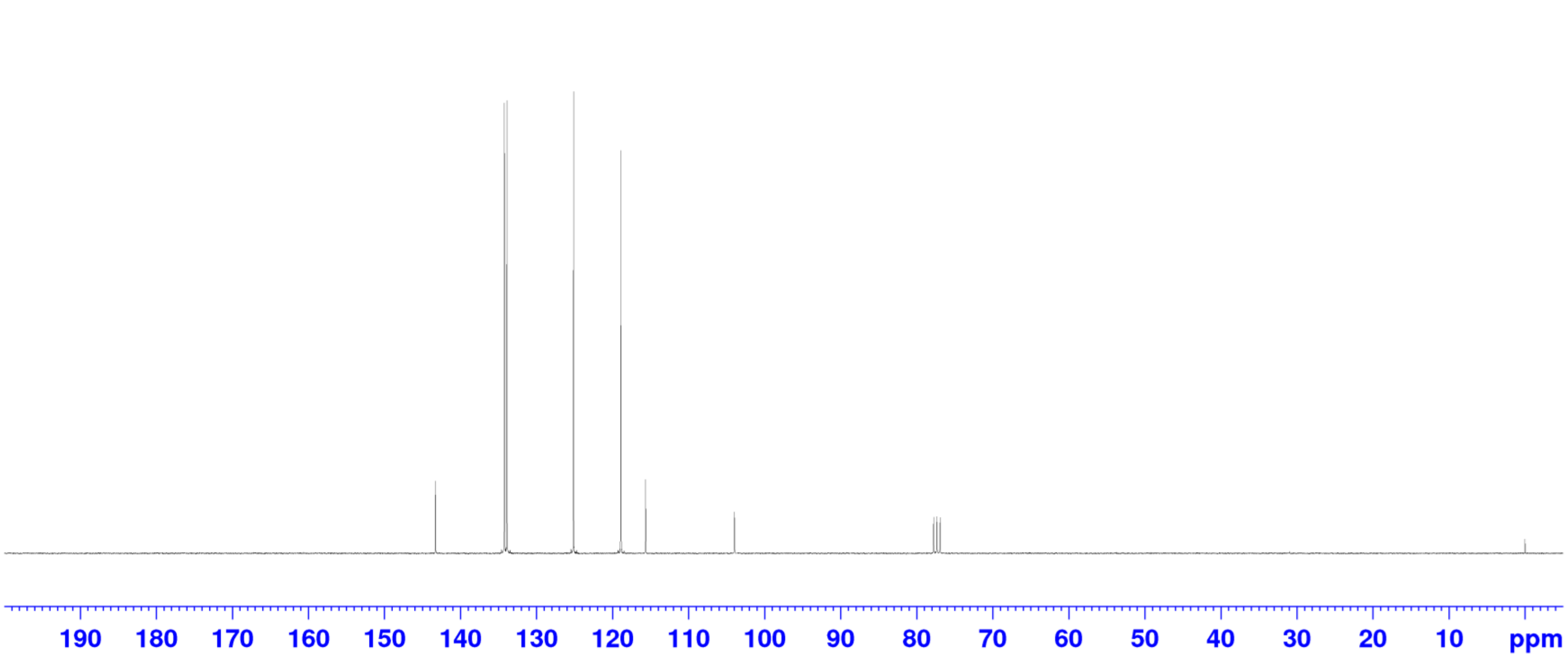

^{1}H NMR of compound **7a'** (300 MHz, $CDCl_3$)

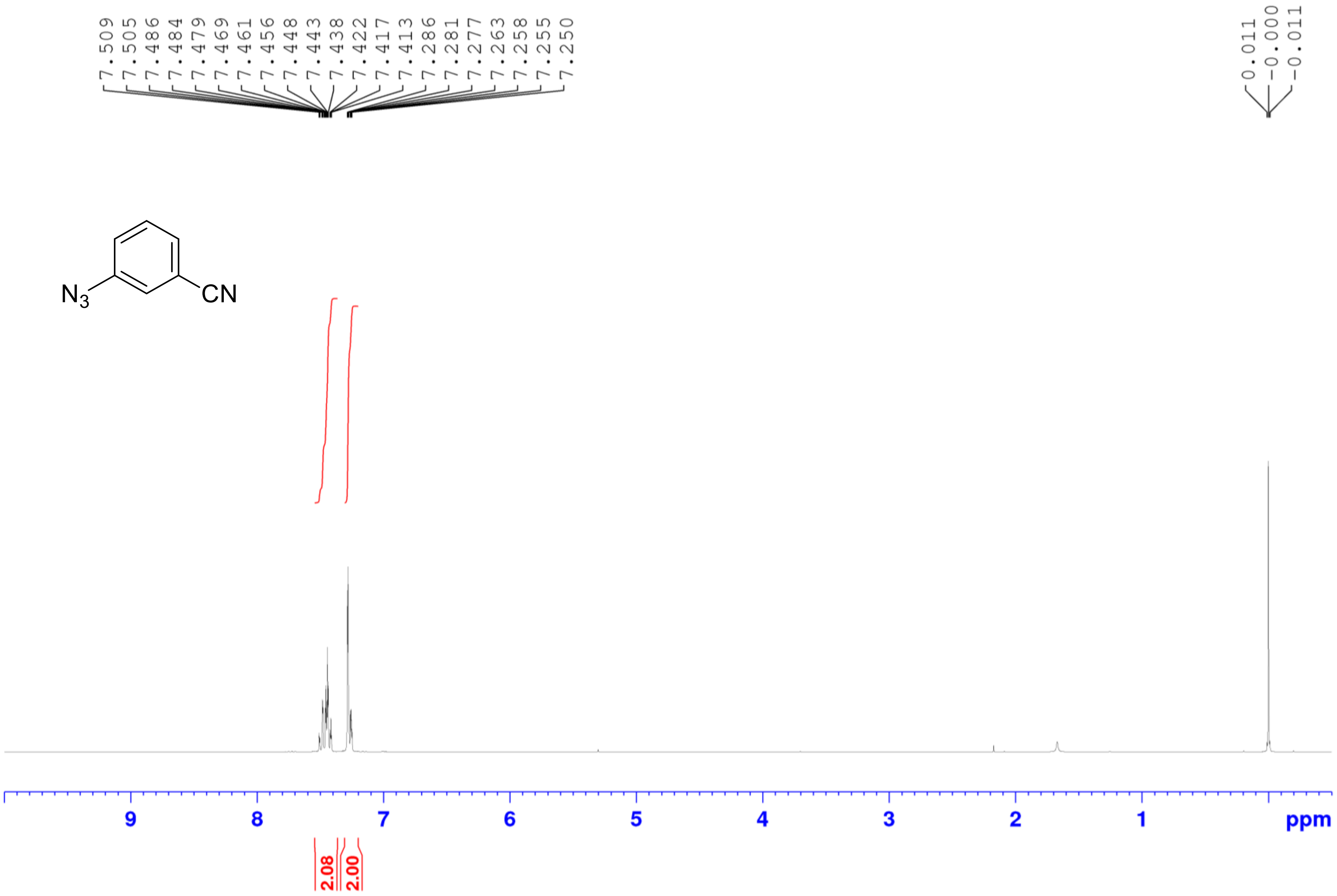

^{13}C NMR of compound **7a'** (75 MHz, $CDCl_3$)

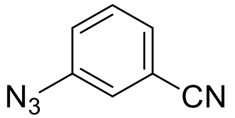

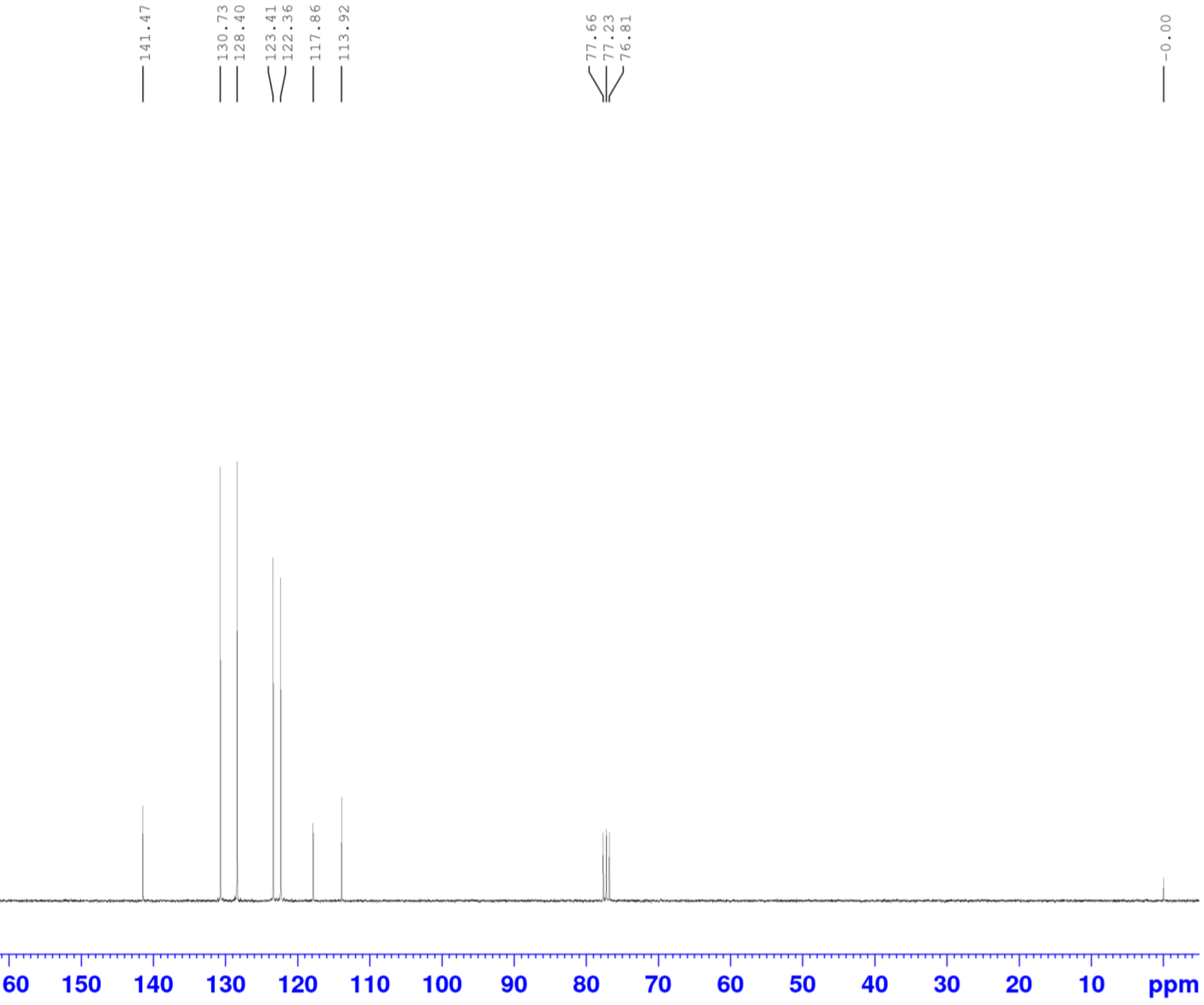

^{1}H NMR of compound **7b'** (300 MHz, $CDCl_3$)

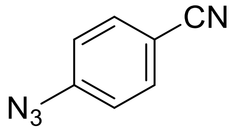

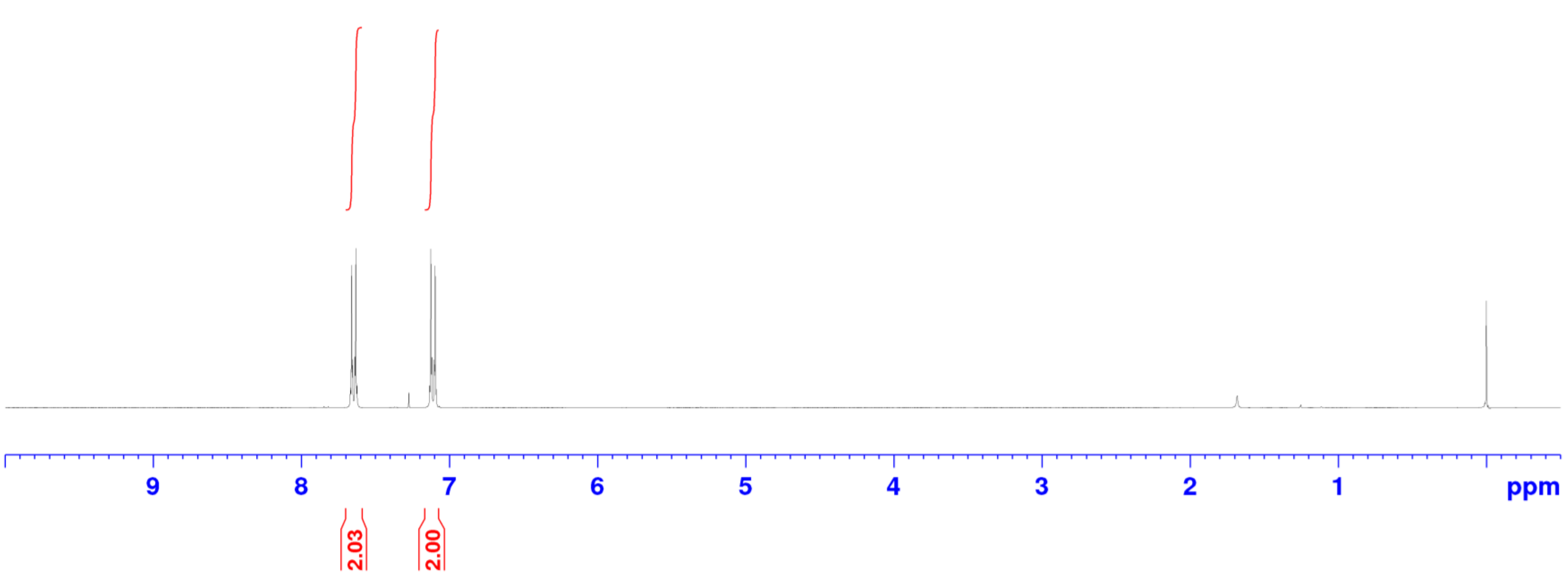

^{13}C NMR of compound **7b’** (75 MHz, $CDCl_3$)

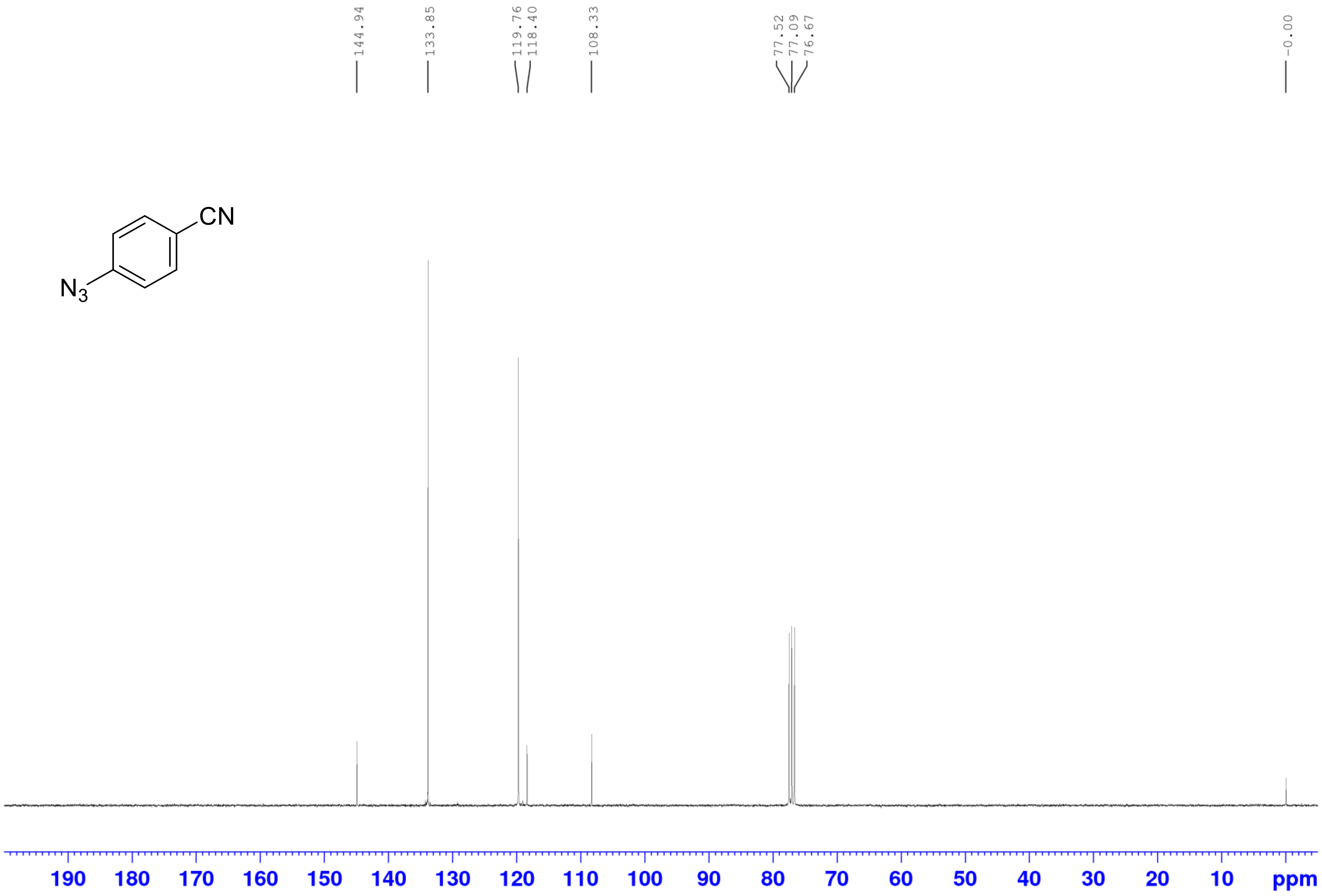

^{1}H NMR of compound **7c'** (300 MHz, $CDCl_3$)

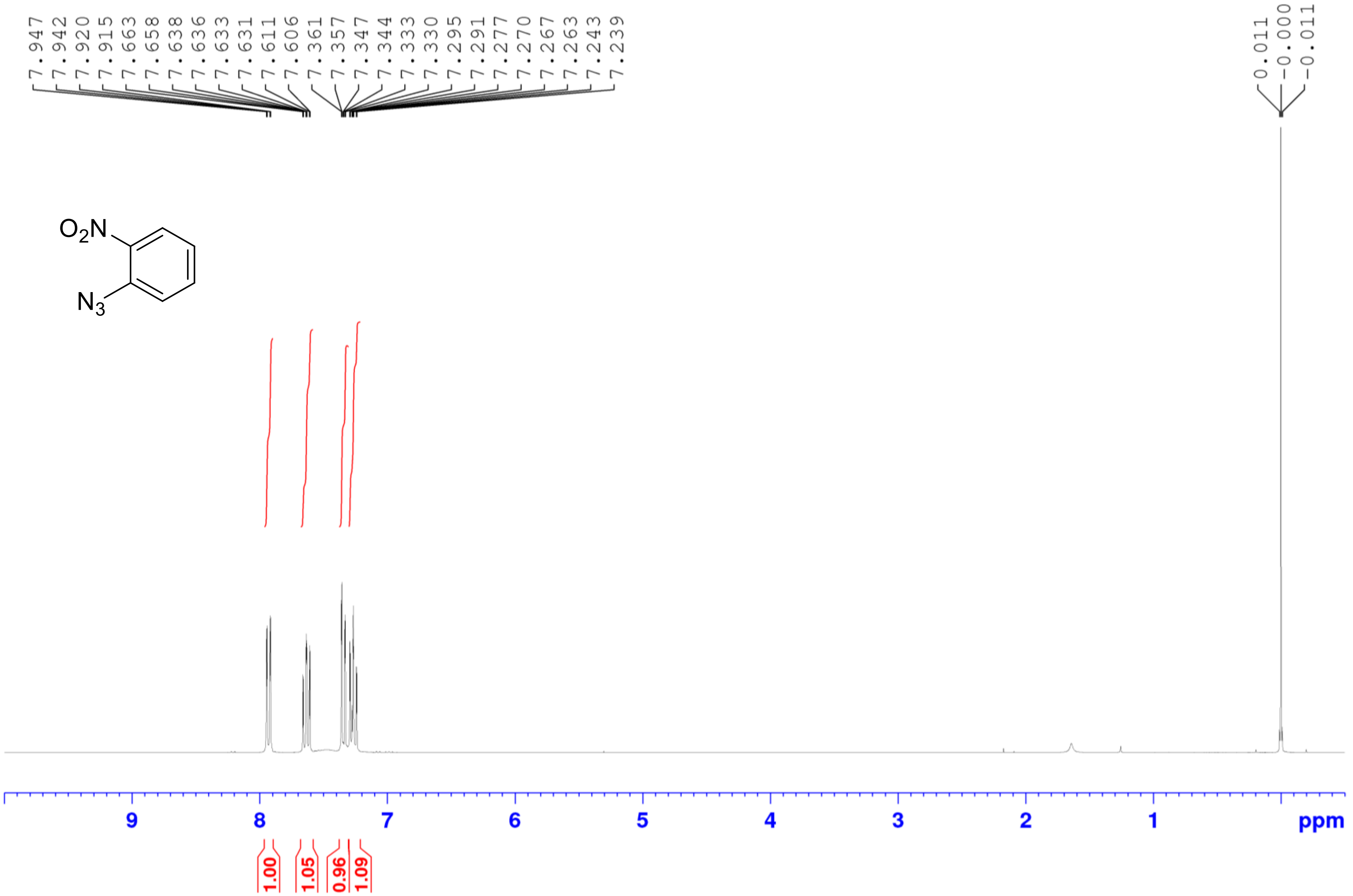

^{13}C NMR of compound **7c’** (75 MHz, $CDCl_3$)

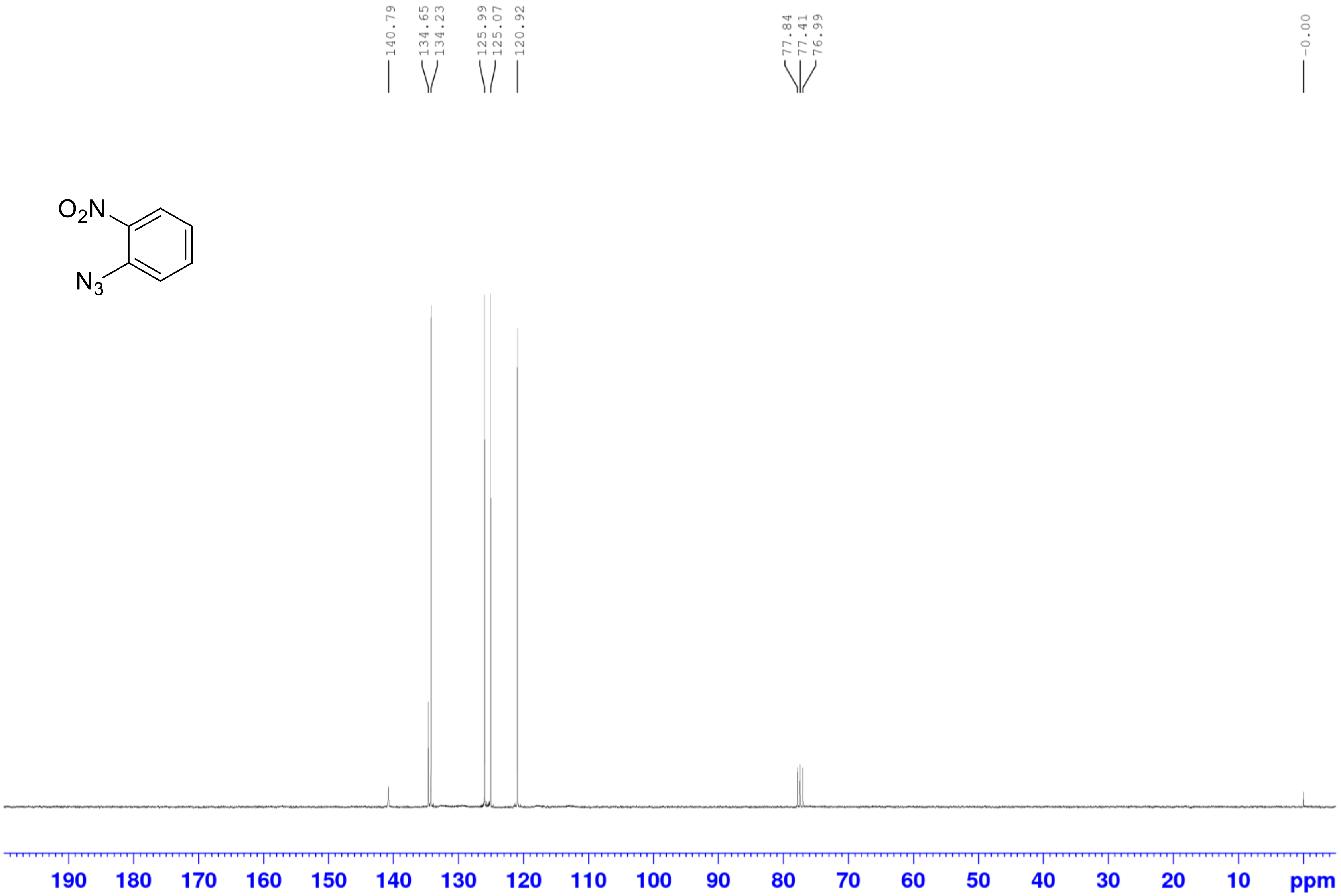

^{1}H NMR of compound **7d'** (300 MHz, $CDCl_3$)

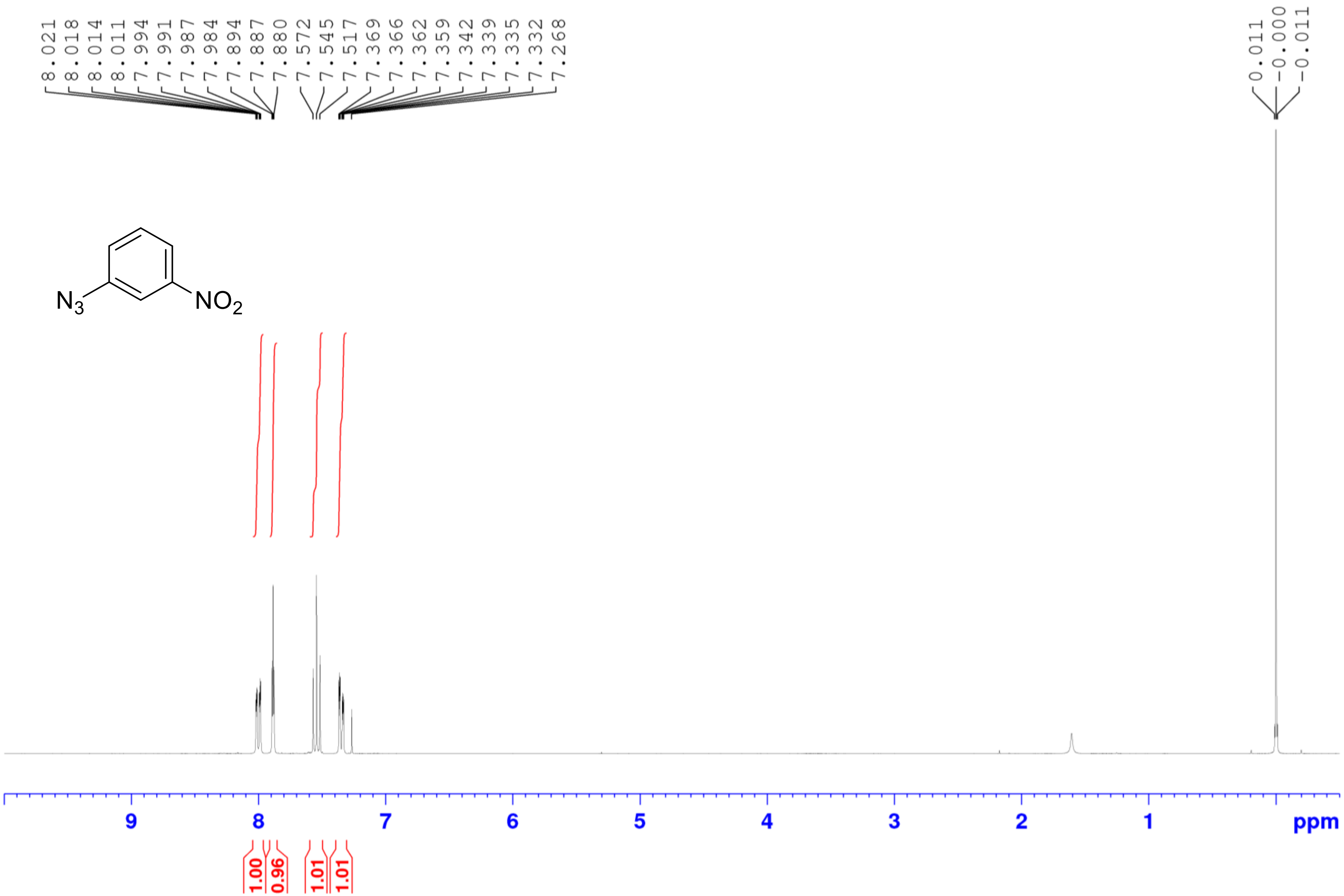

^{13}C NMR of compound **7d’** (75 MHz, $CDCl_3$)

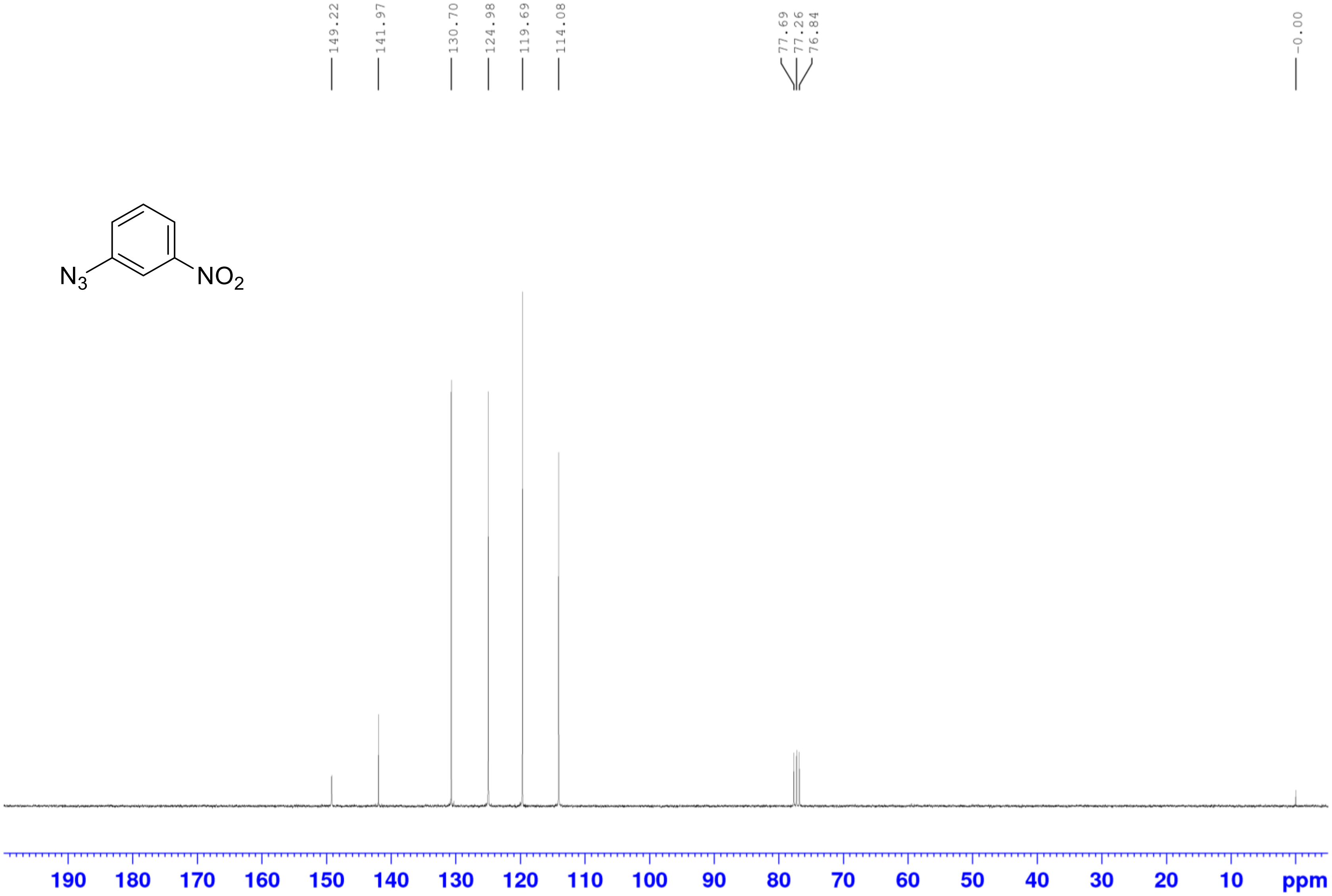

^{1}H NMR of compound **7e'** (300 MHz, $CDCl_3$)

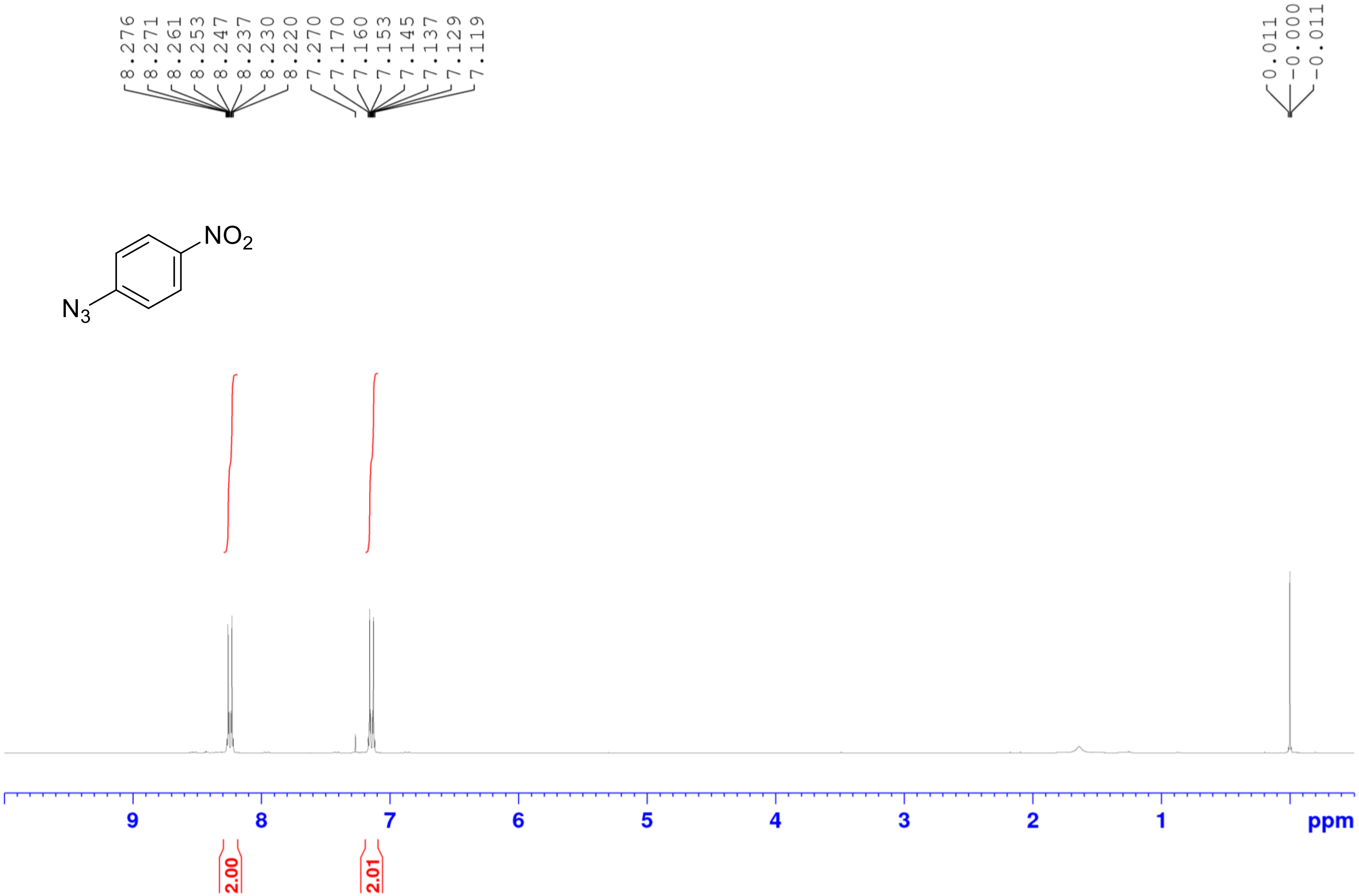

^{13}C NMR of compound **7e’** (75 MHz, $CDCl_3$)

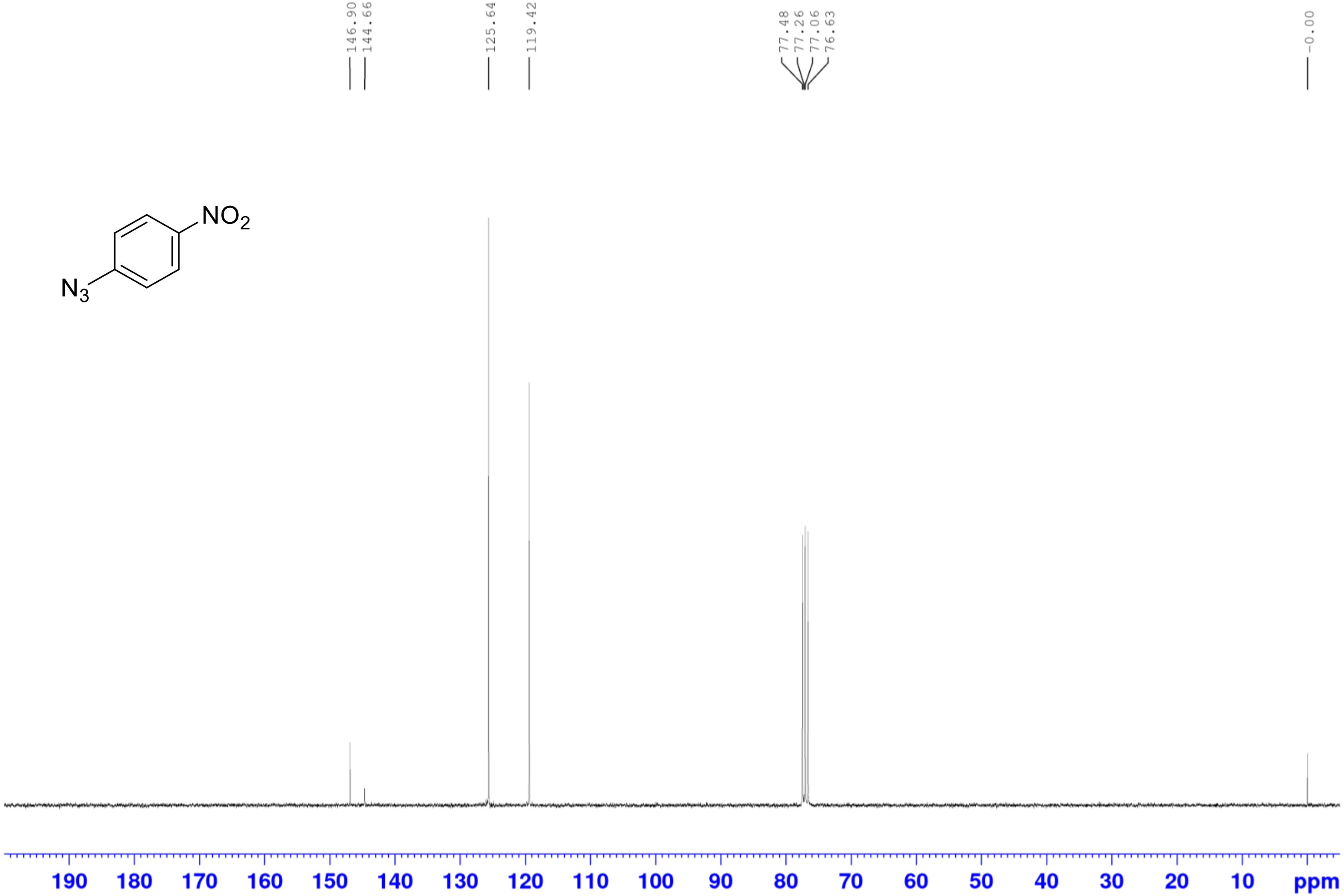

^{1}H NMR of compound **7f’** (300 MHz, $CDCl_3$)

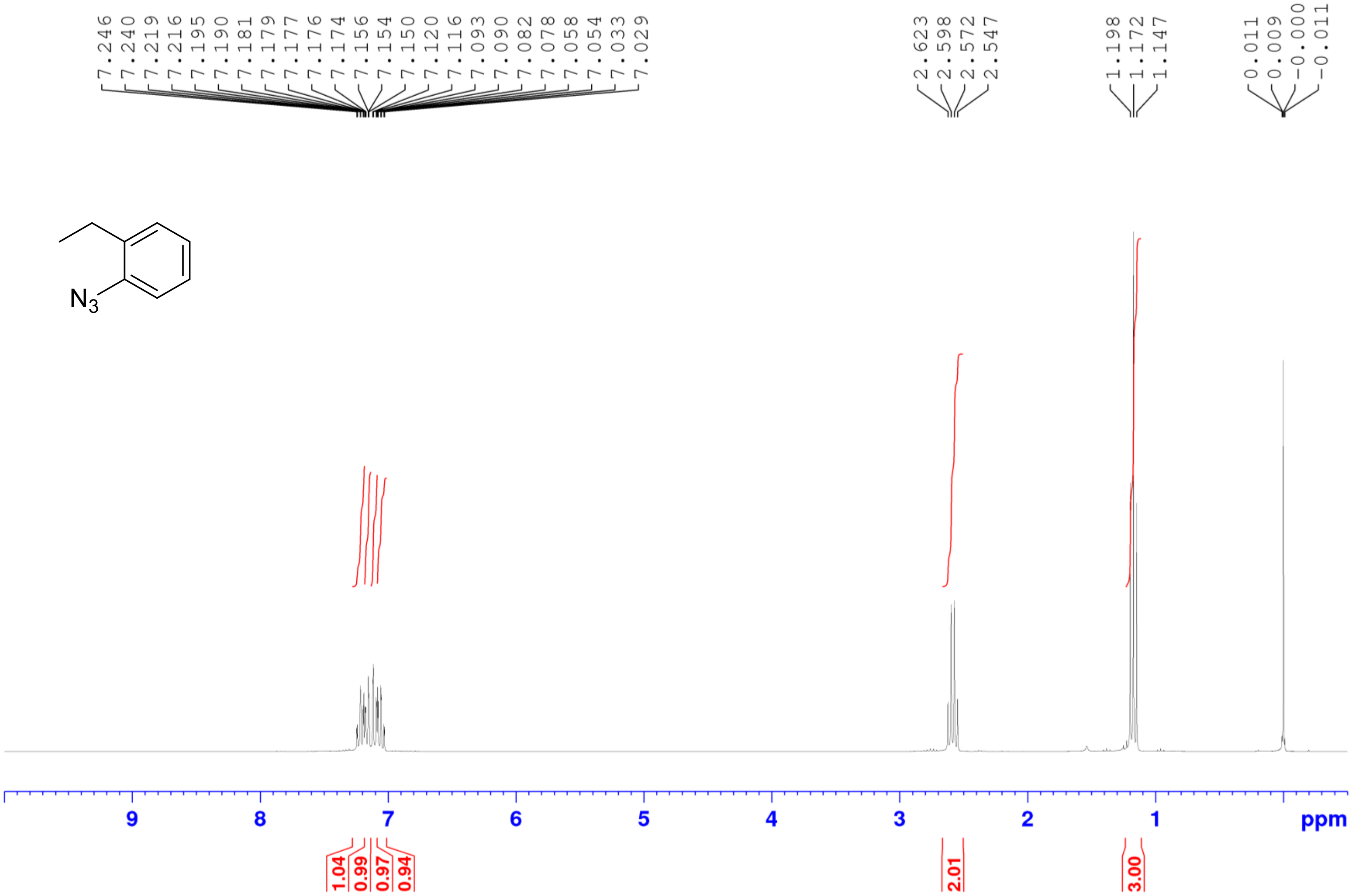

^{13}C NMR of compound **7f'** (75 MHz, $CDCl_3$)

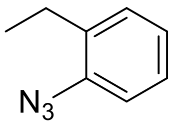

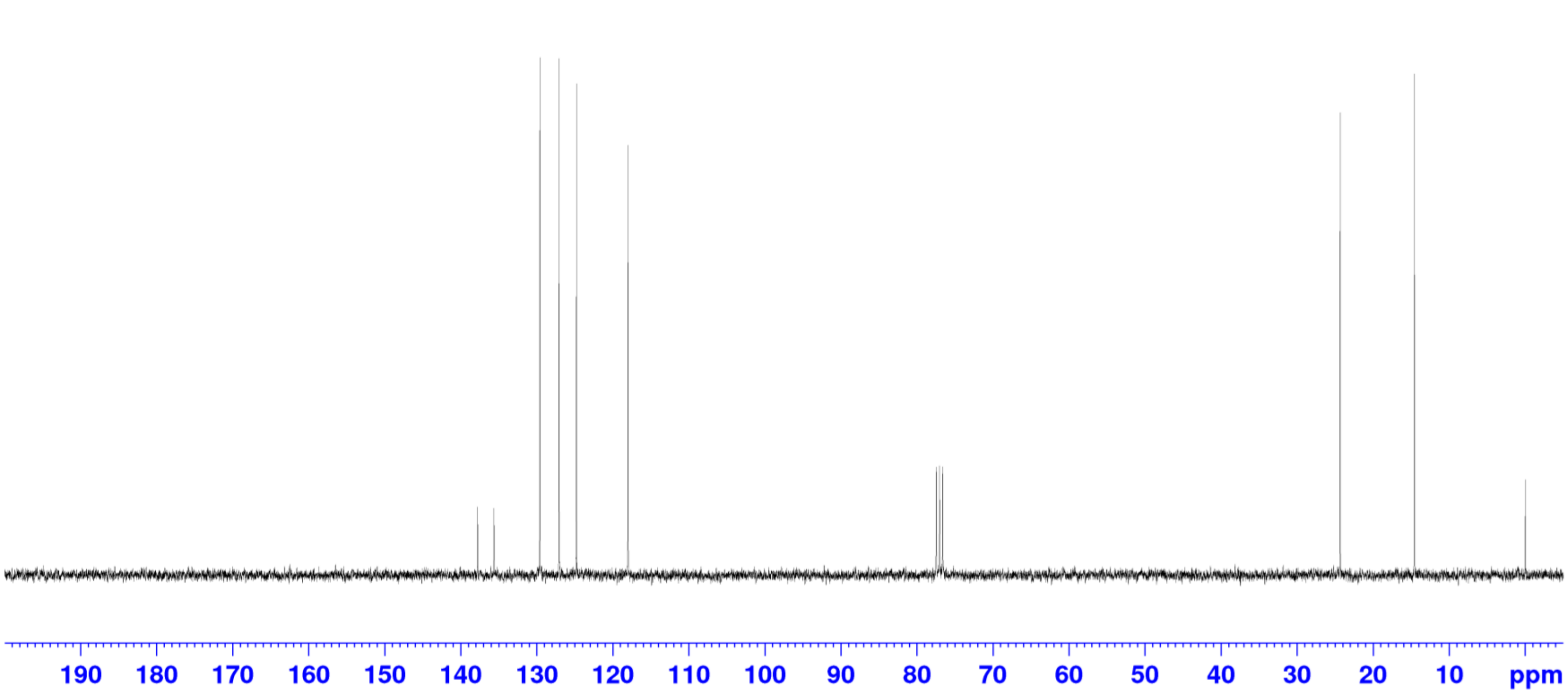

^{1}H NMR of compound **7g’** (300 MHz, $CDCl_3$)

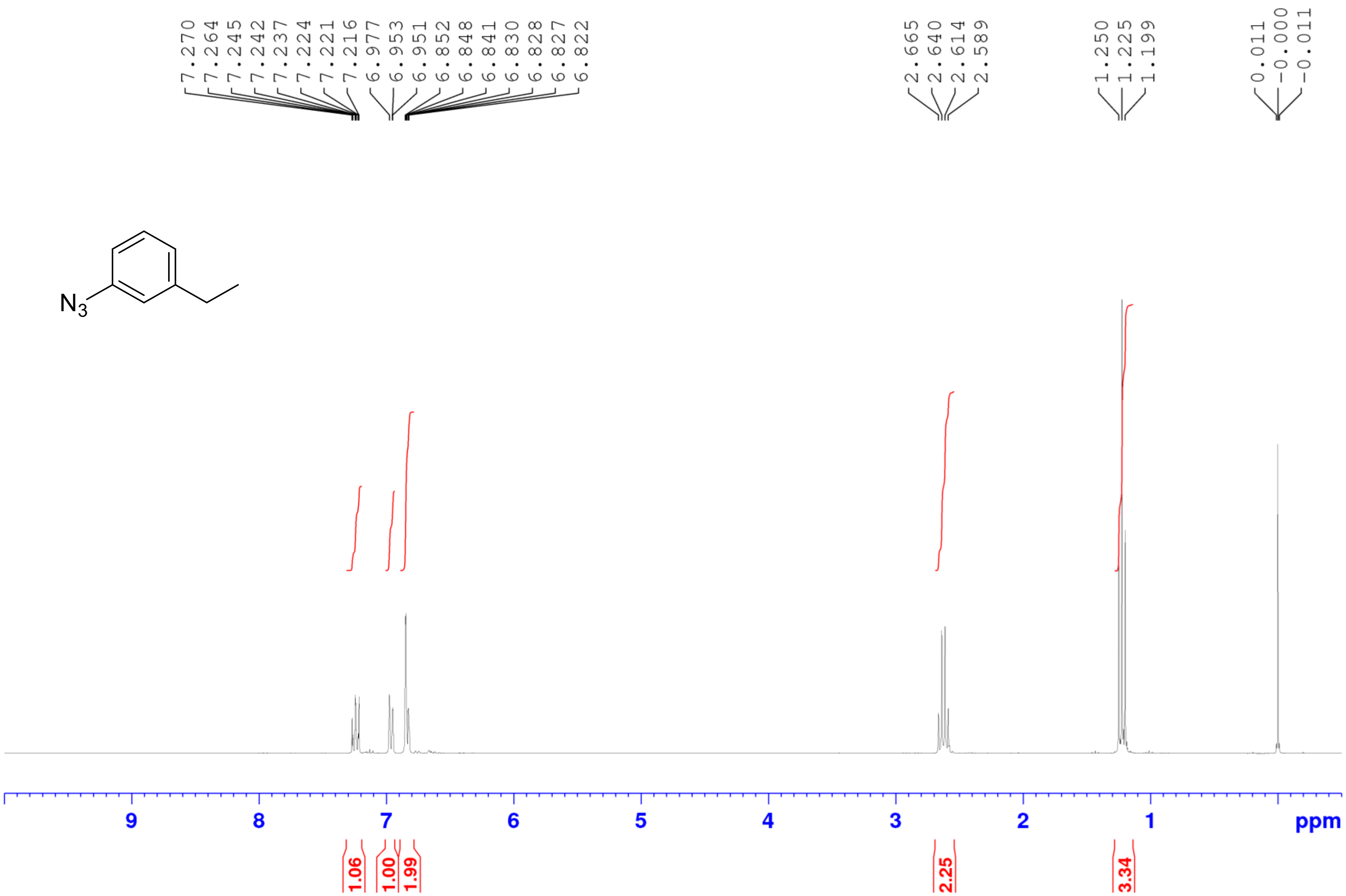

^{13}C NMR of compound **7g’** (75 MHz, $CDCl_3$)

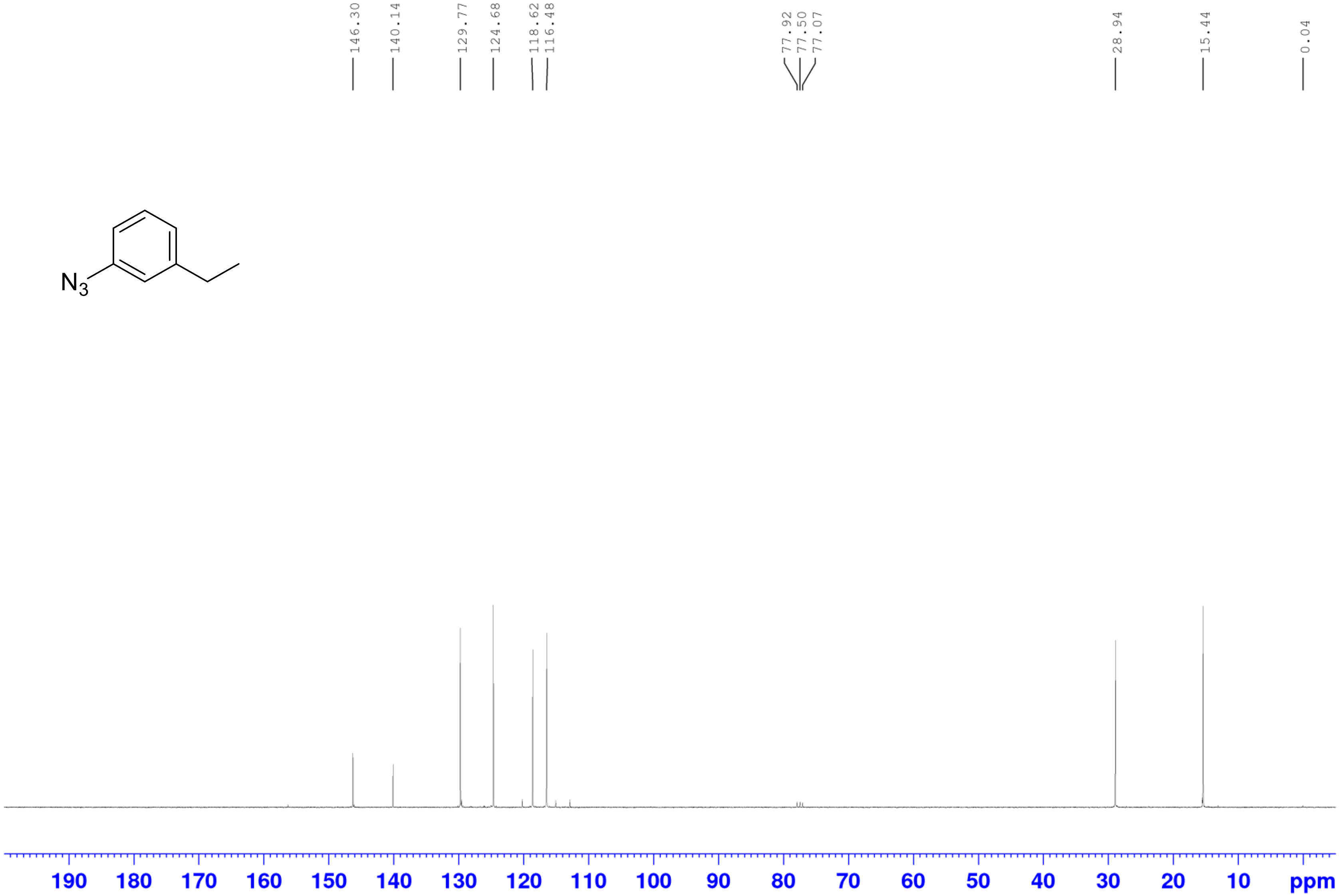

^{1}H NMR of compound **7h’** (300 MHz, $CDCl_3$)

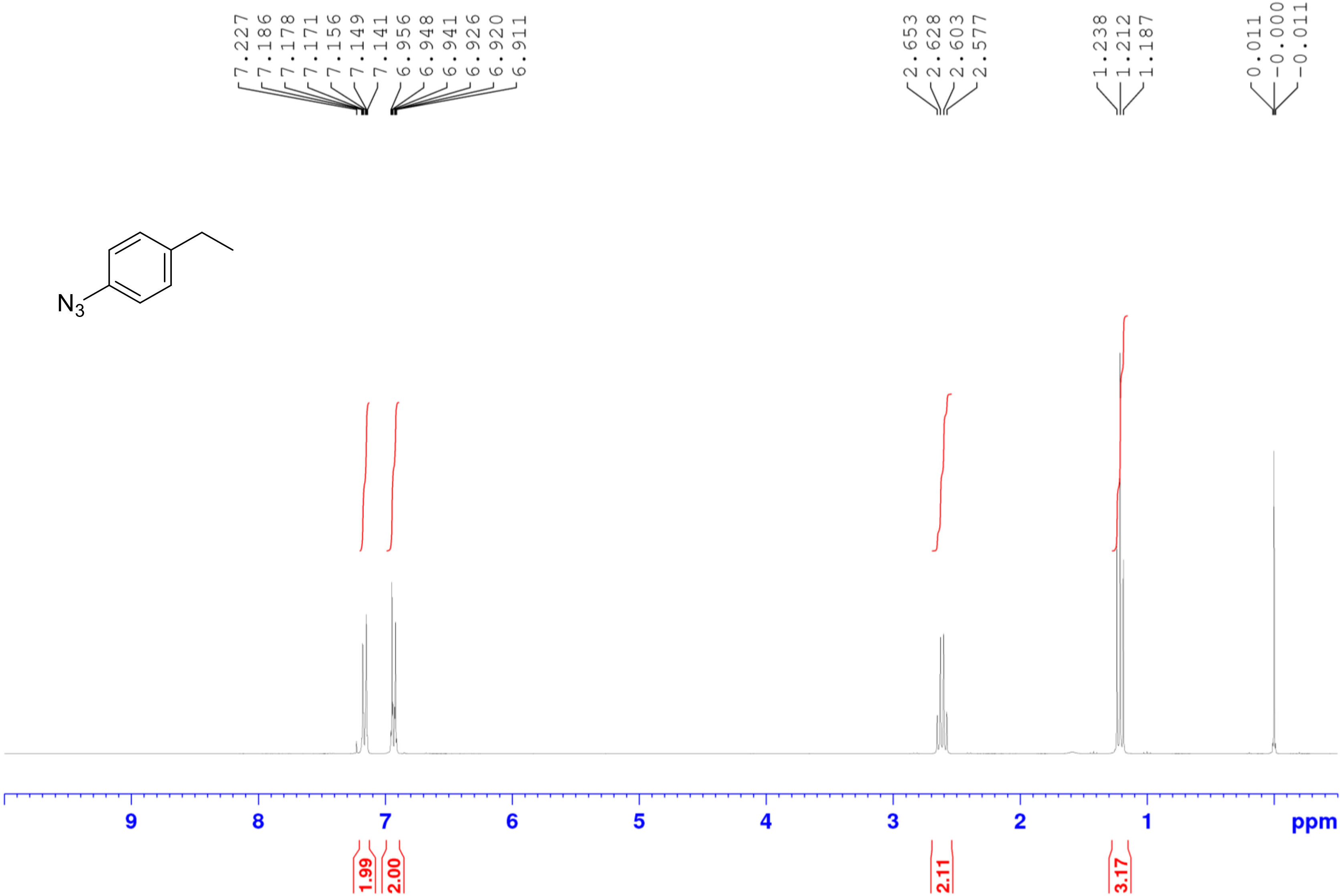

^{13}C NMR of compound **7h'** (75 MHz, $CDCl_3$)

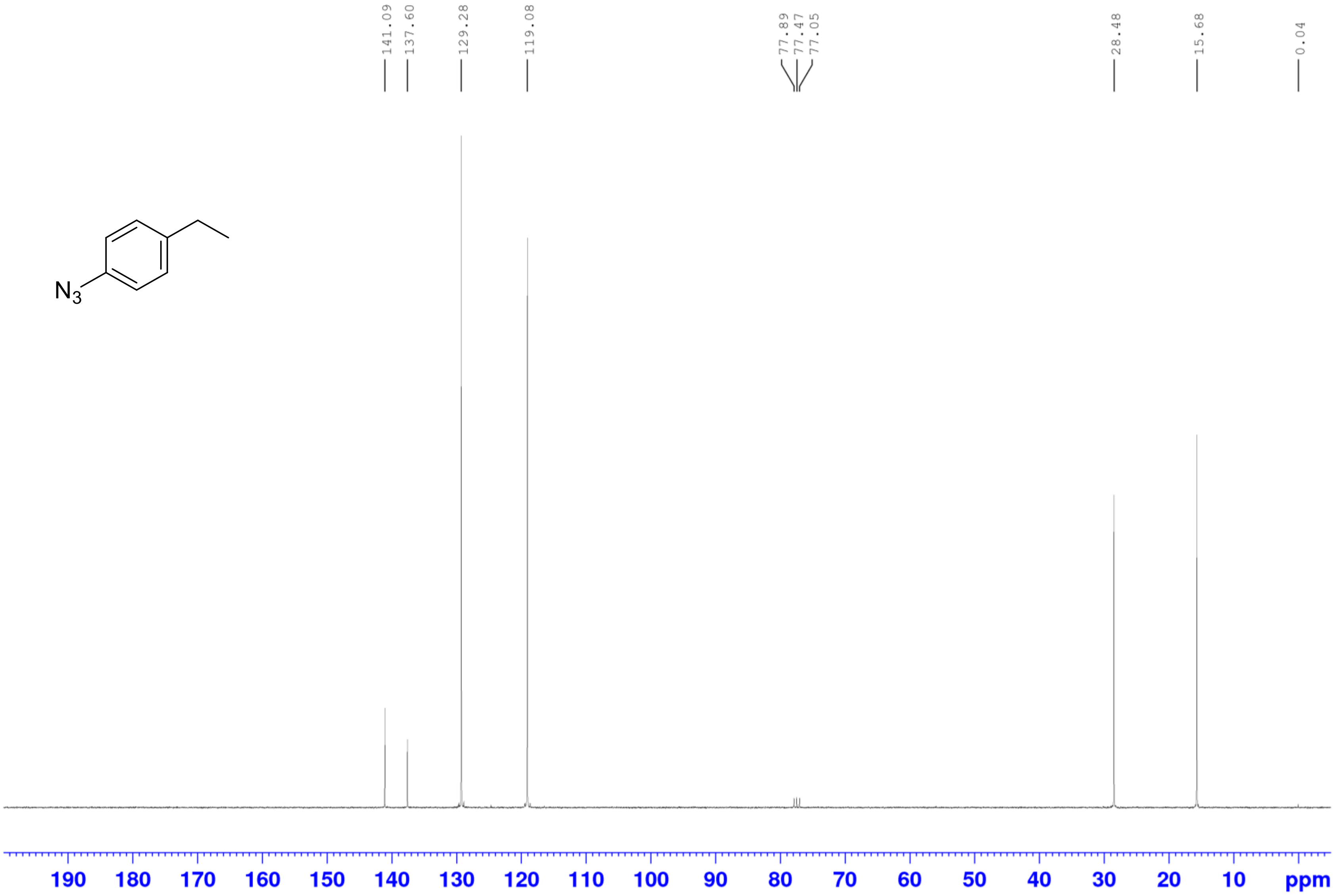

^{1}H NMR of compound **7i'** (300 MHz, $CDCl_3$)

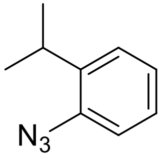

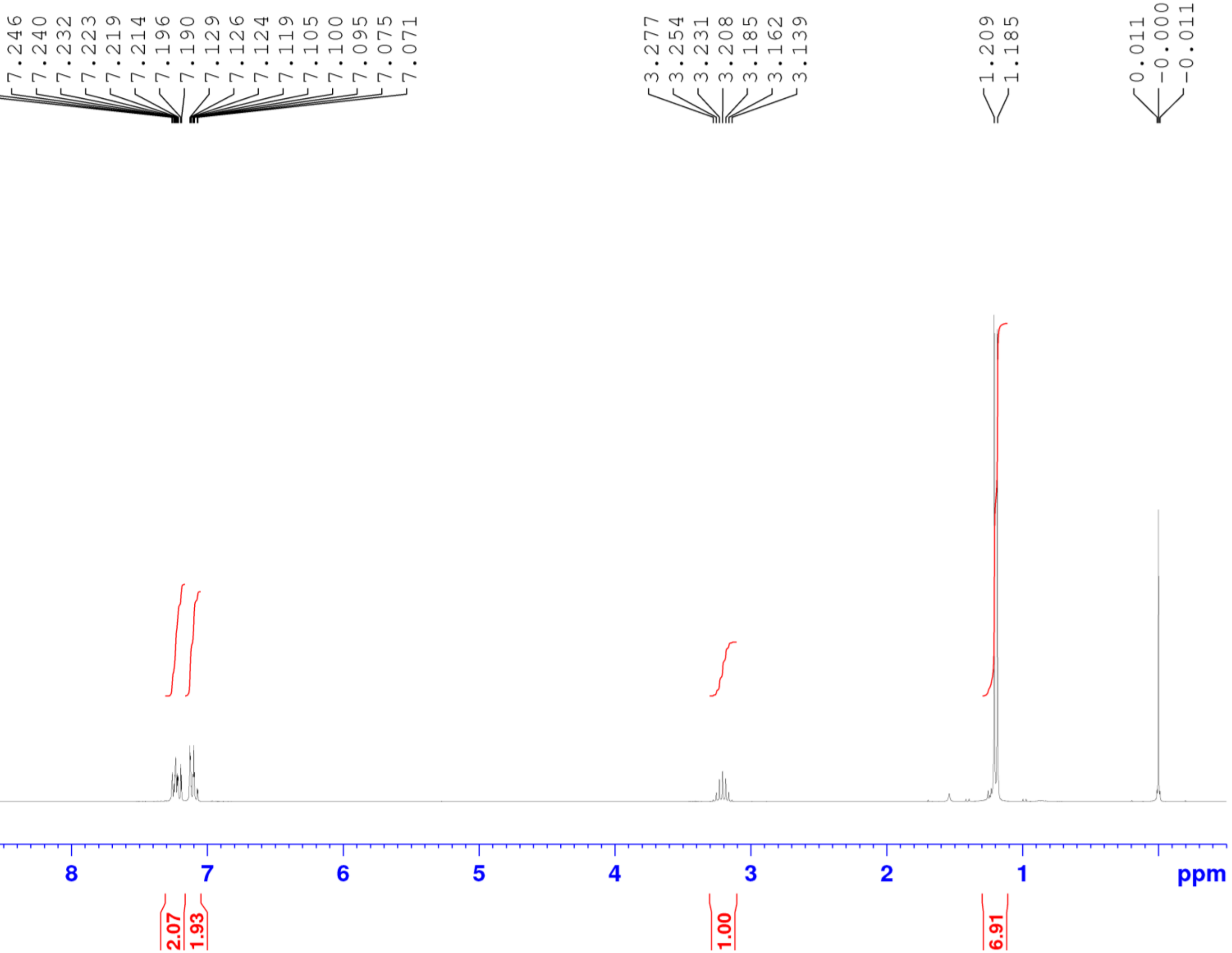

^{13}C NMR of compound **7i'** (75 MHz, $CDCl_3$)

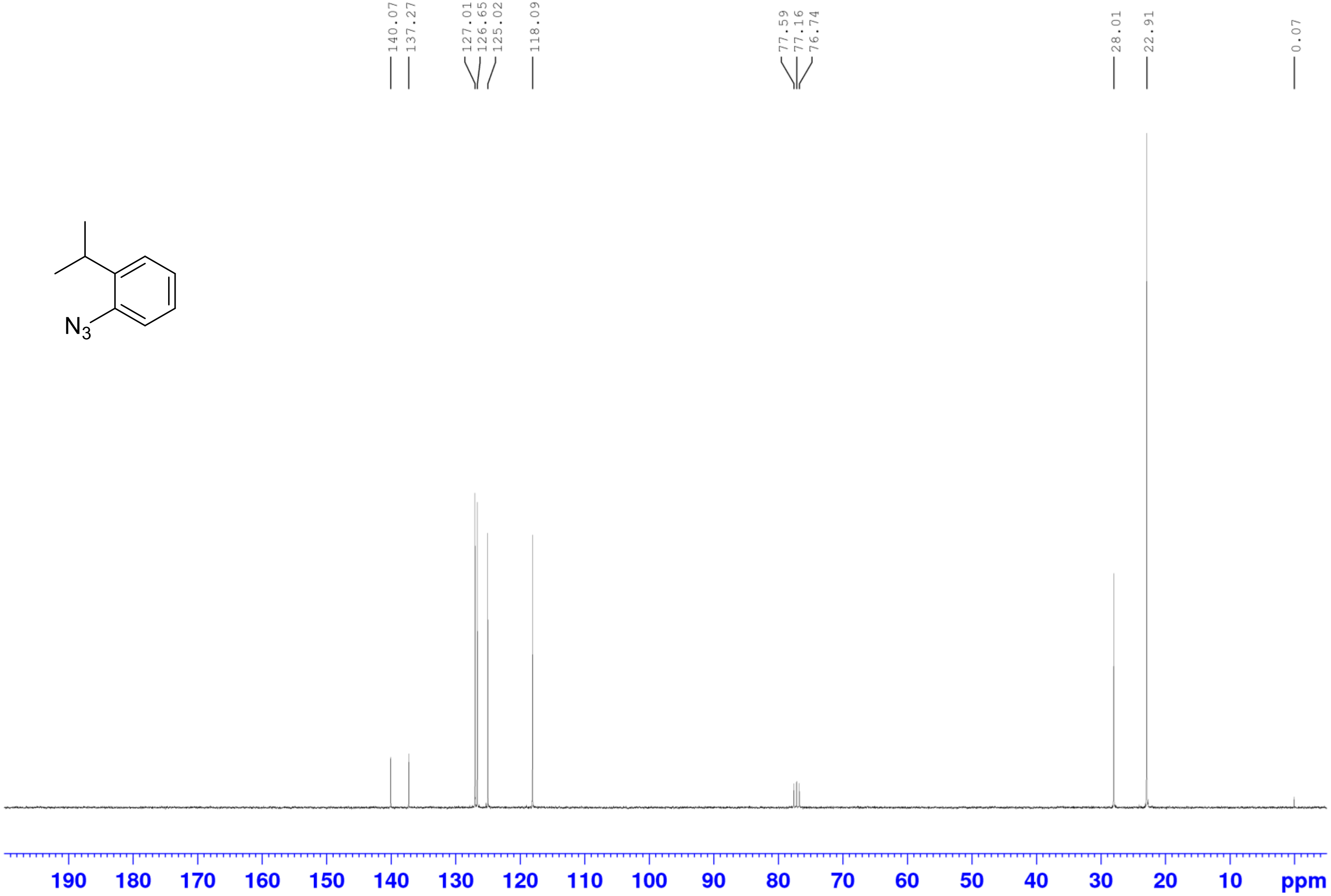

^{1}H NMR of compound **7j'** (300 MHz, $CDCl_3$)

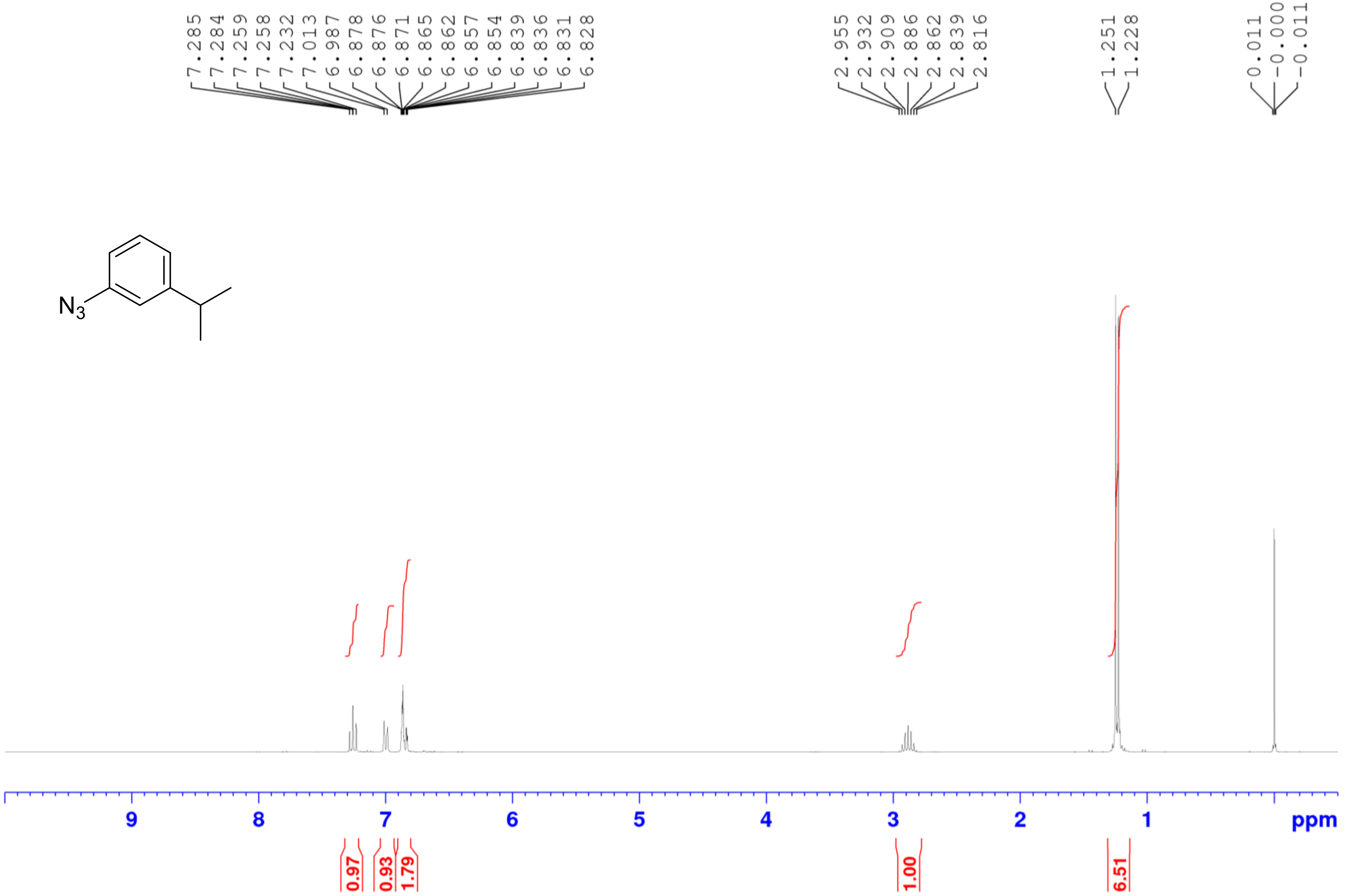

^{13}C NMR of compound **7j'** (75 MHz, $CDCl_3$)

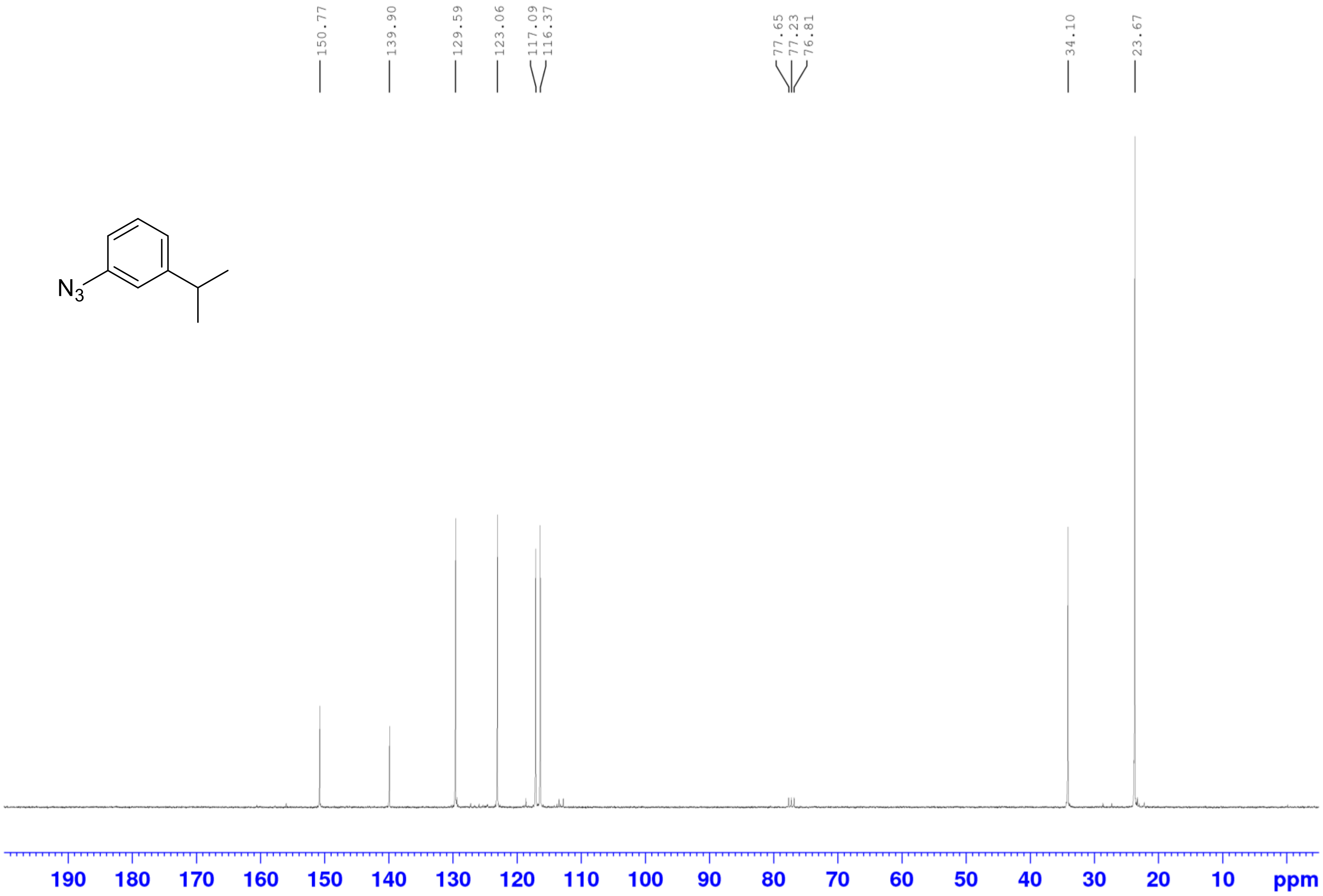

^{1}H NMR of compound **7k'** (300 MHz, $CDCl_3$)

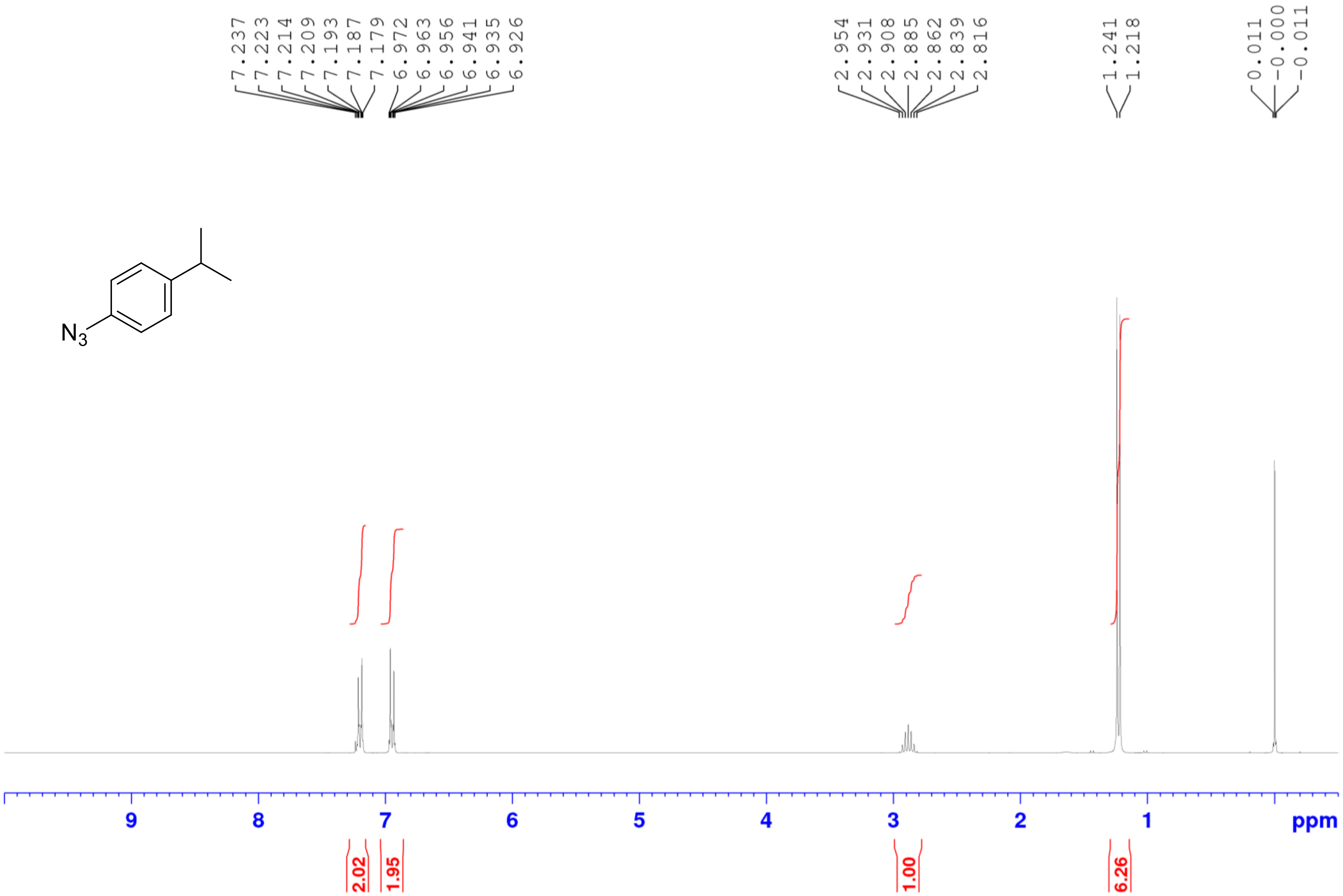

^{13}C NMR of compound **7k'** (75 MHz, $CDCl_3$)

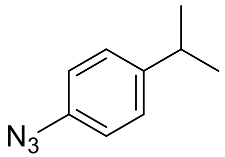

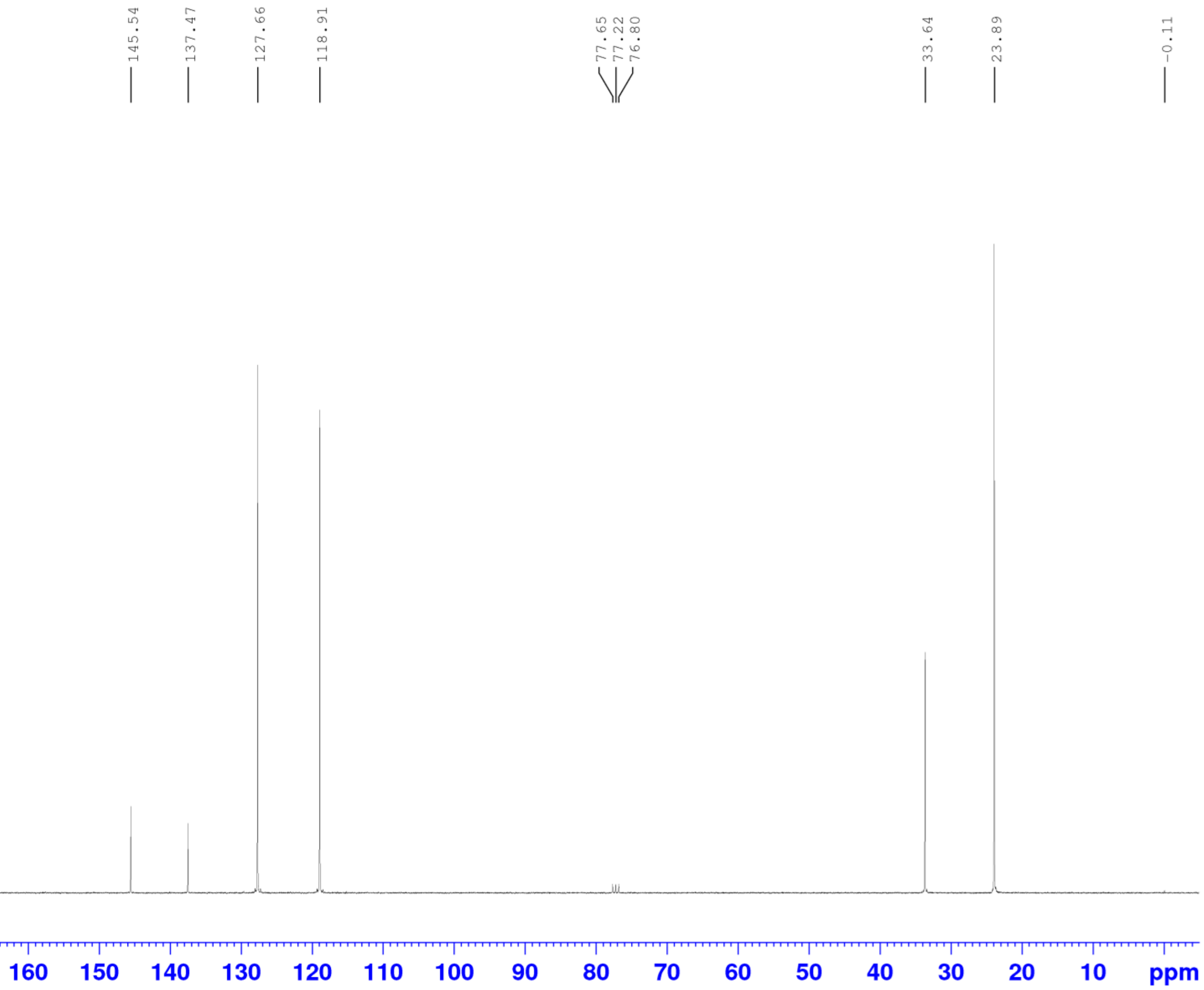

^{1}H NMR of compound **7l’** (300 MHz, $CDCl_3$)

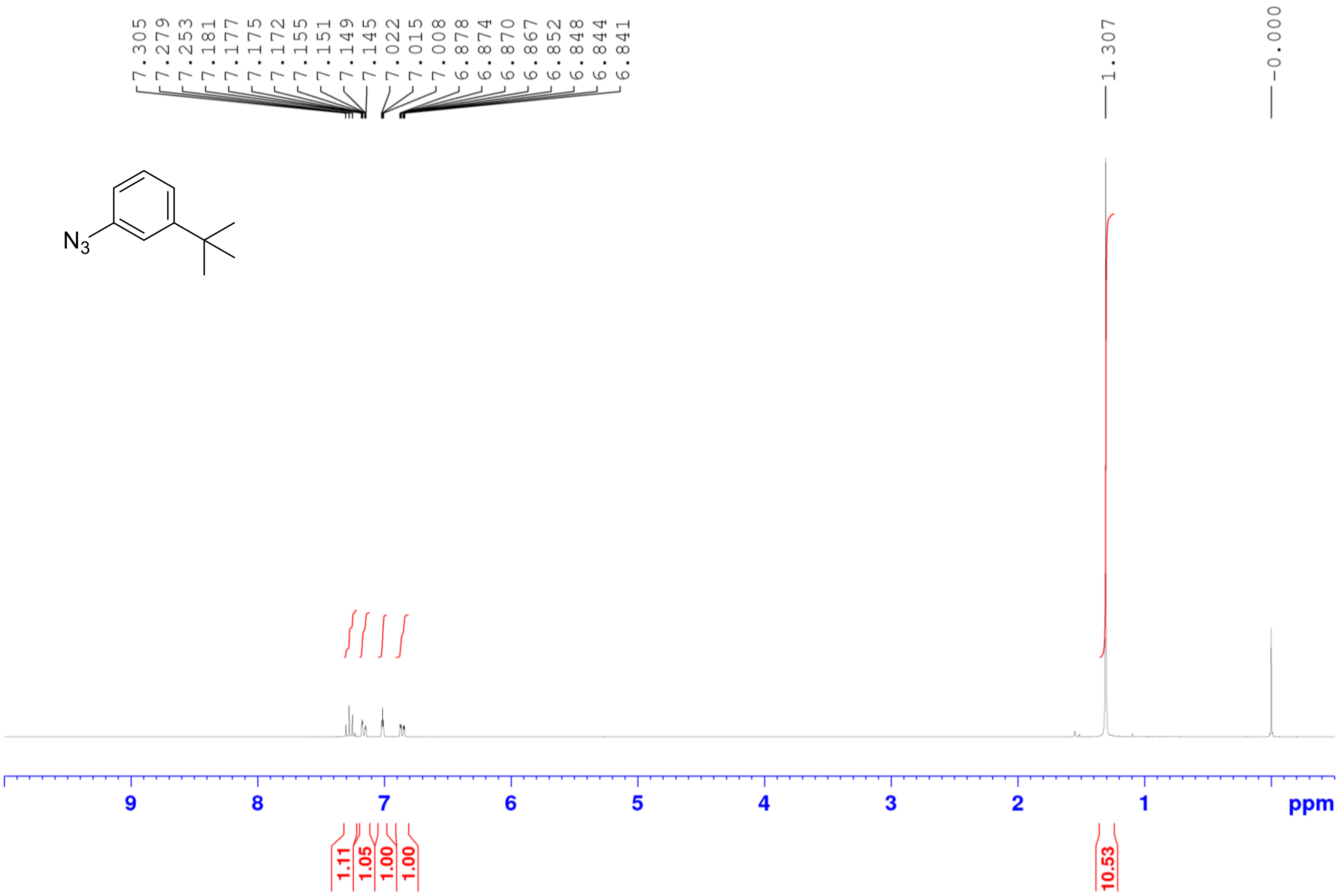

^{13}C NMR of compound **7l’** (75 MHz, $CDCl_3$)

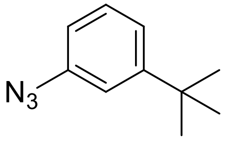

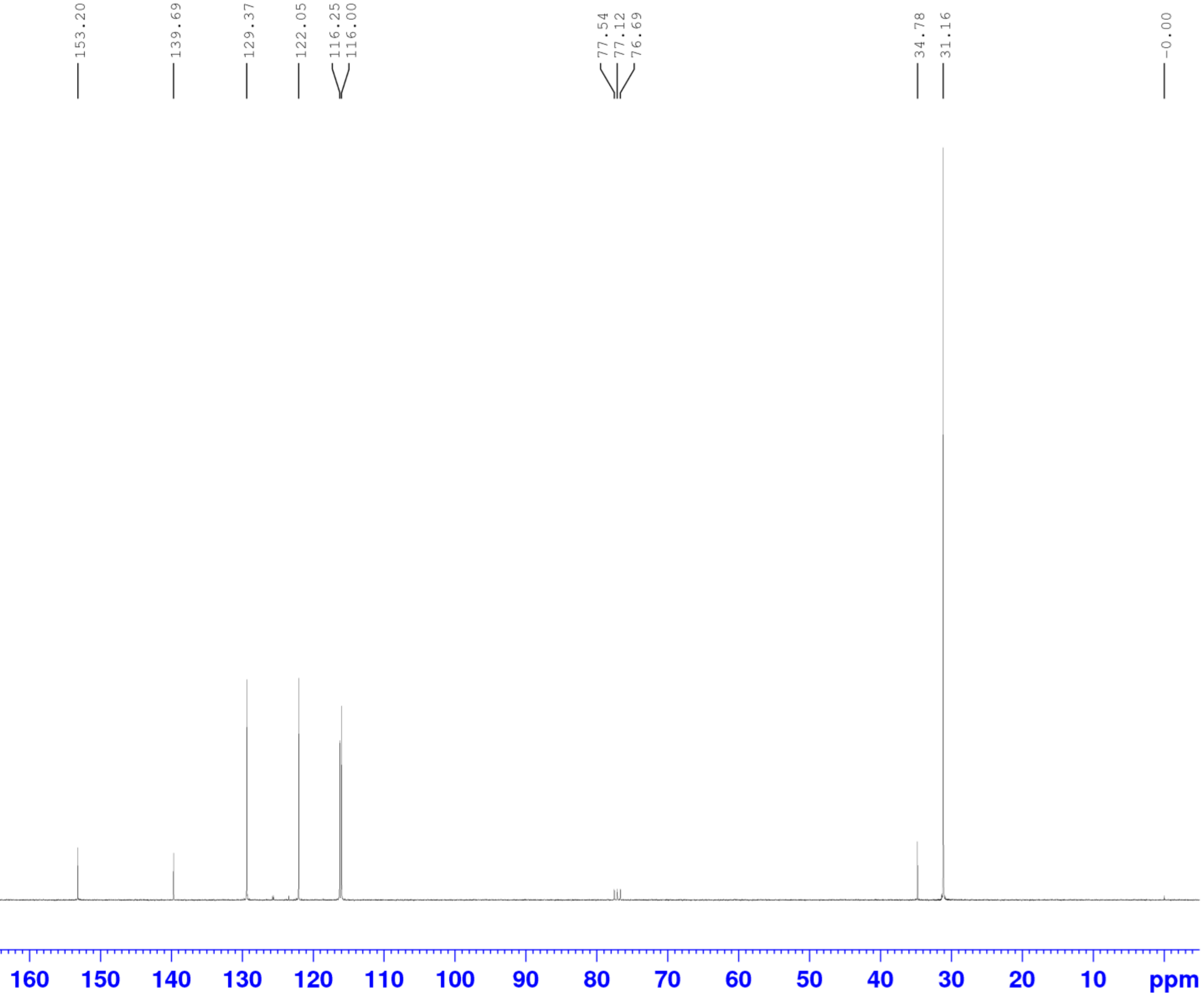

^{1}H NMR of compound **7m'** (300 MHz, $CDCl_3$)

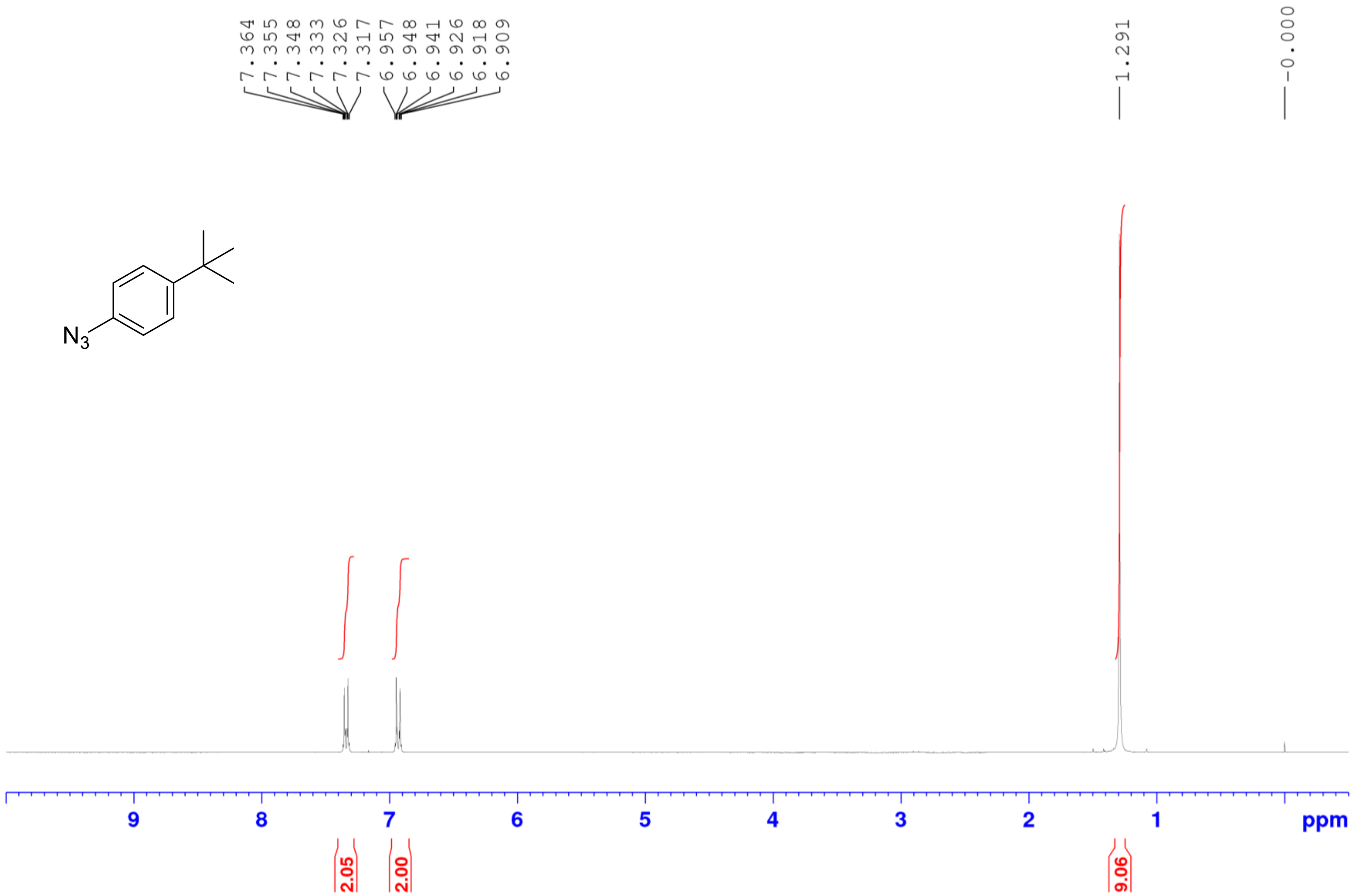

^{13}C NMR of compound **7m'** (75 MHz, $CDCl_3$)

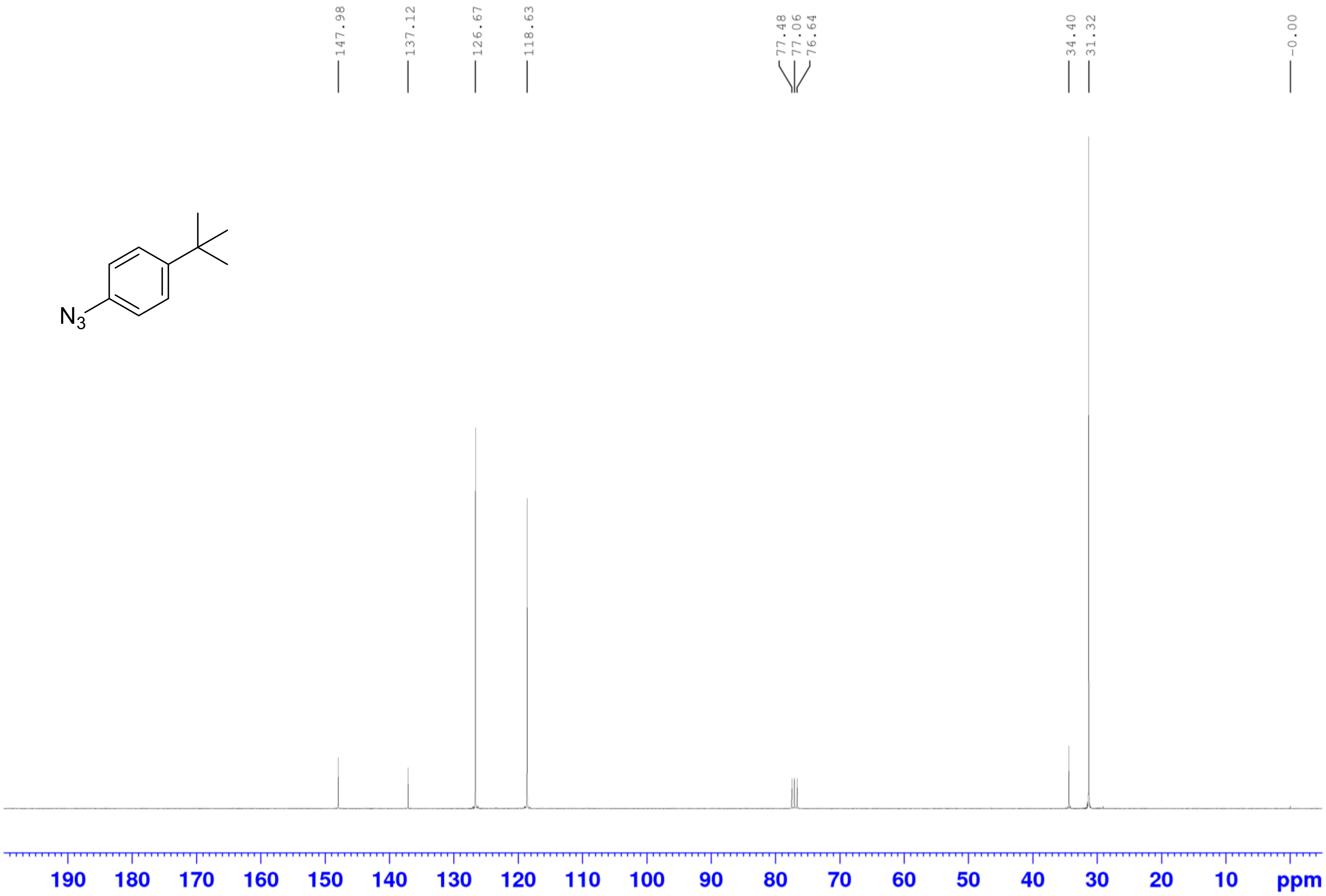

^{1}H NMR of compound **7n'** (300 MHz, DMSO-d_6)

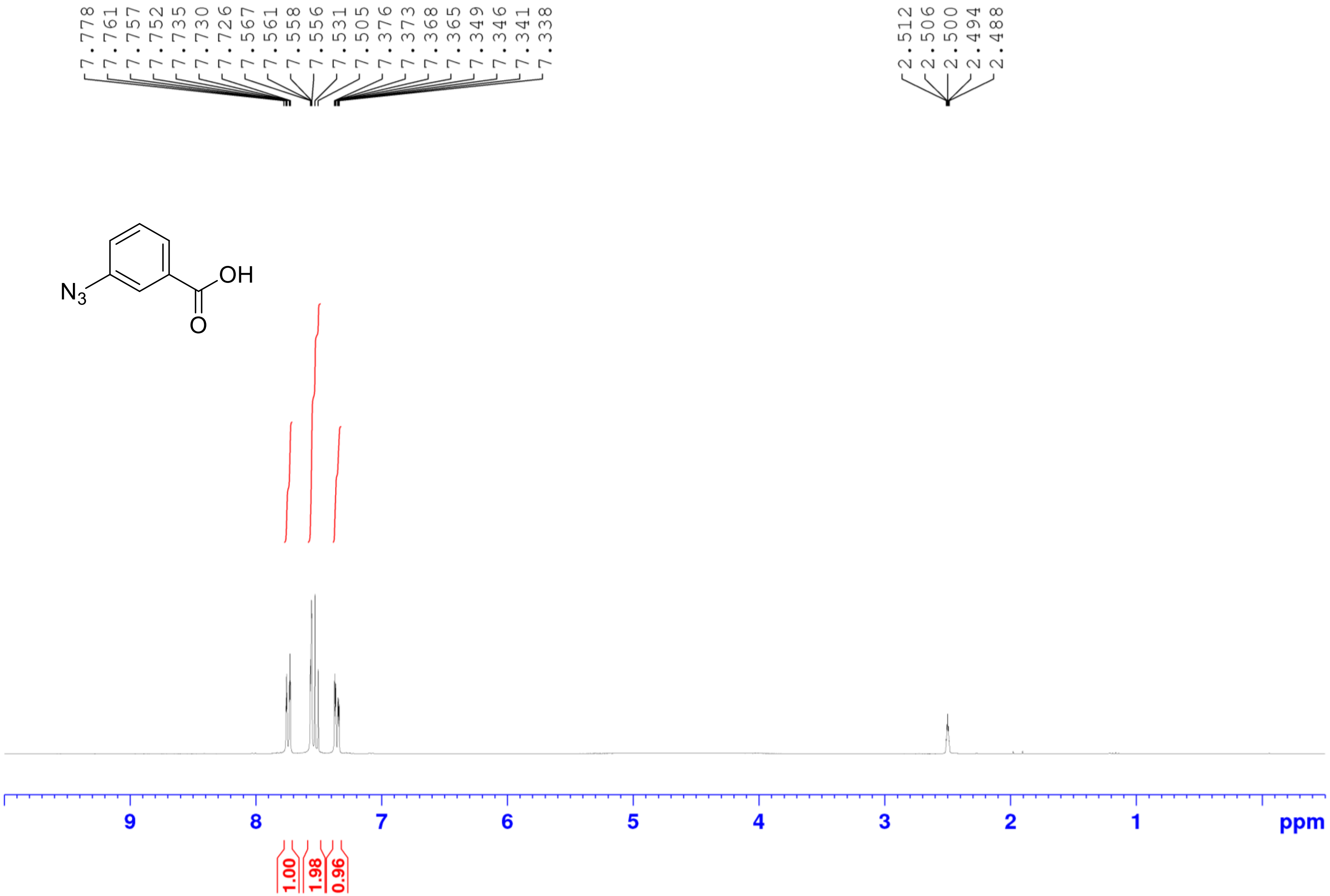

^{13}C NMR of compound **7n'** (75 MHz, DMSO-d_6)

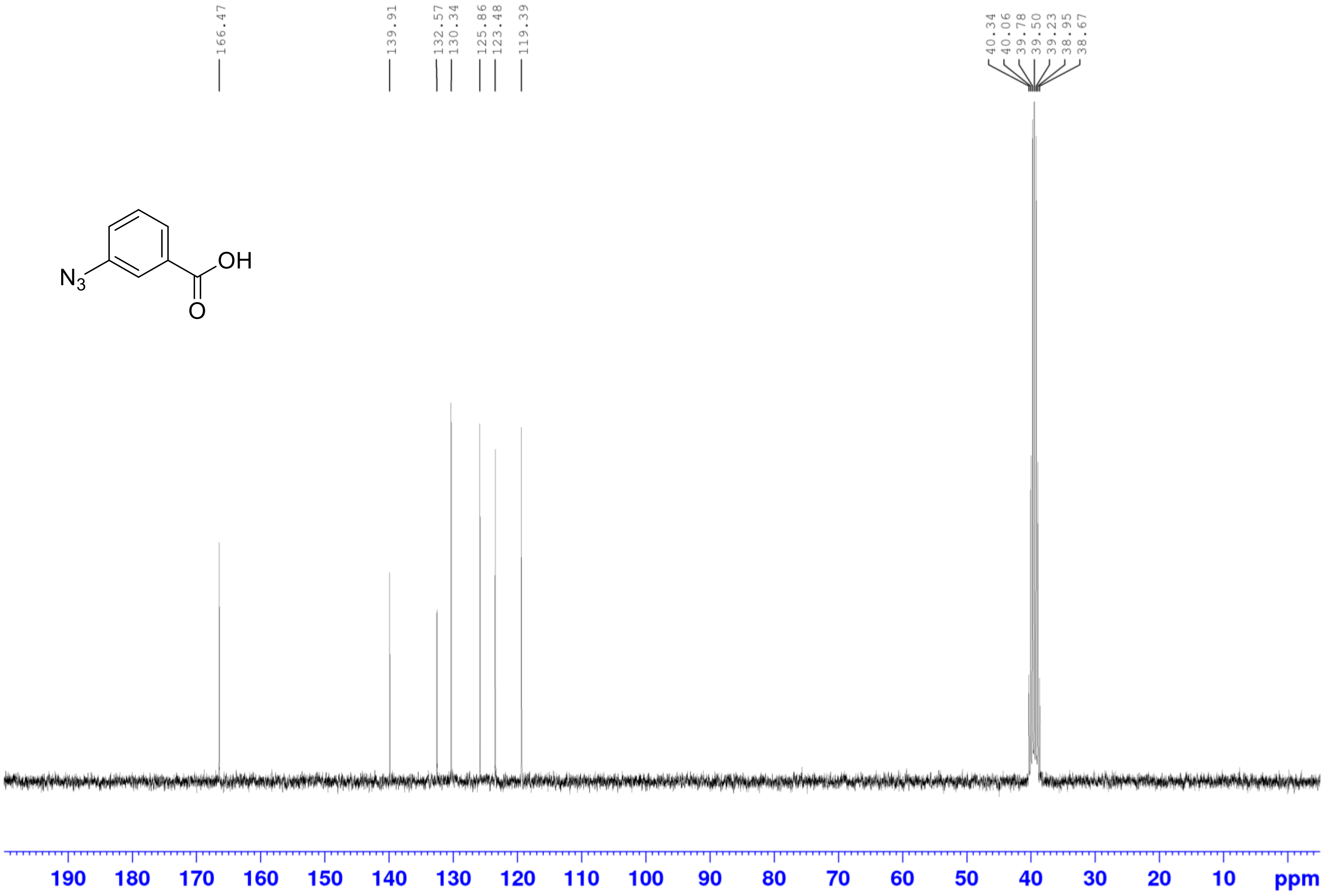

^{1}H NMR of compound **7o'** (300 MHz, MeOD-d_4)

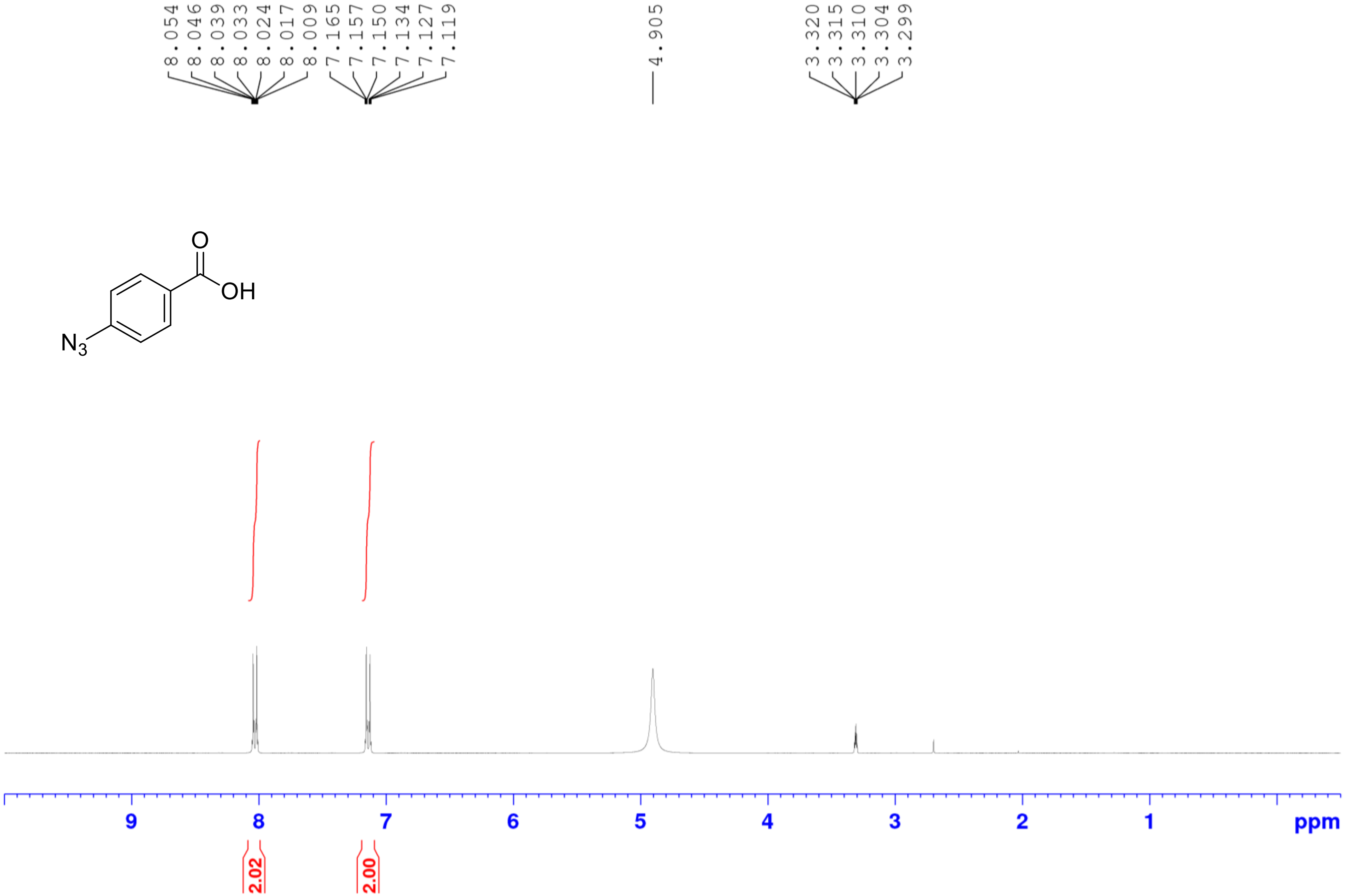

^{13}C NMR of compound **7o'** (75 MHz, MeOD-d_4)

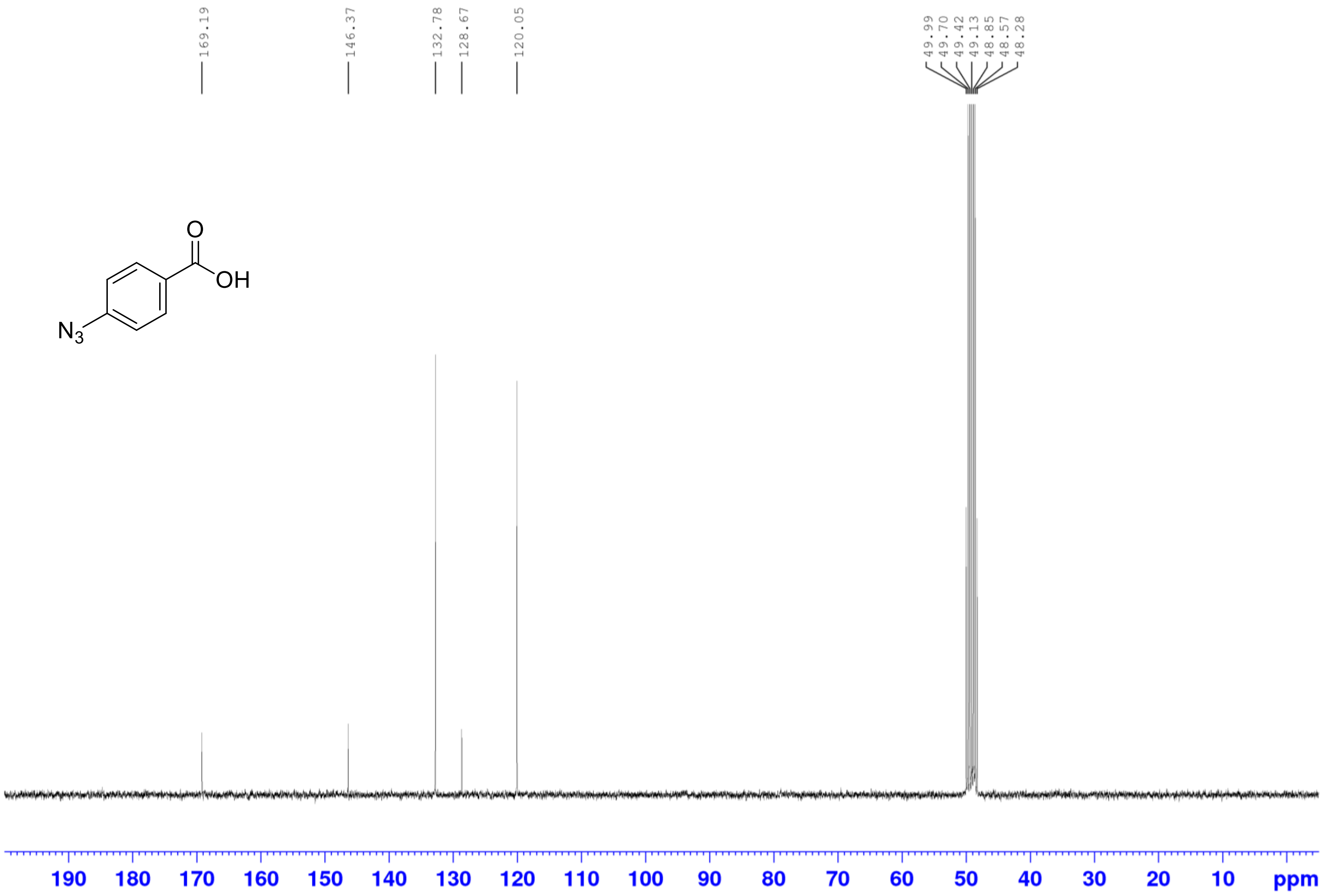

^{1}H NMR of compound **7u’** (300 MHz, DMSO-d_6)

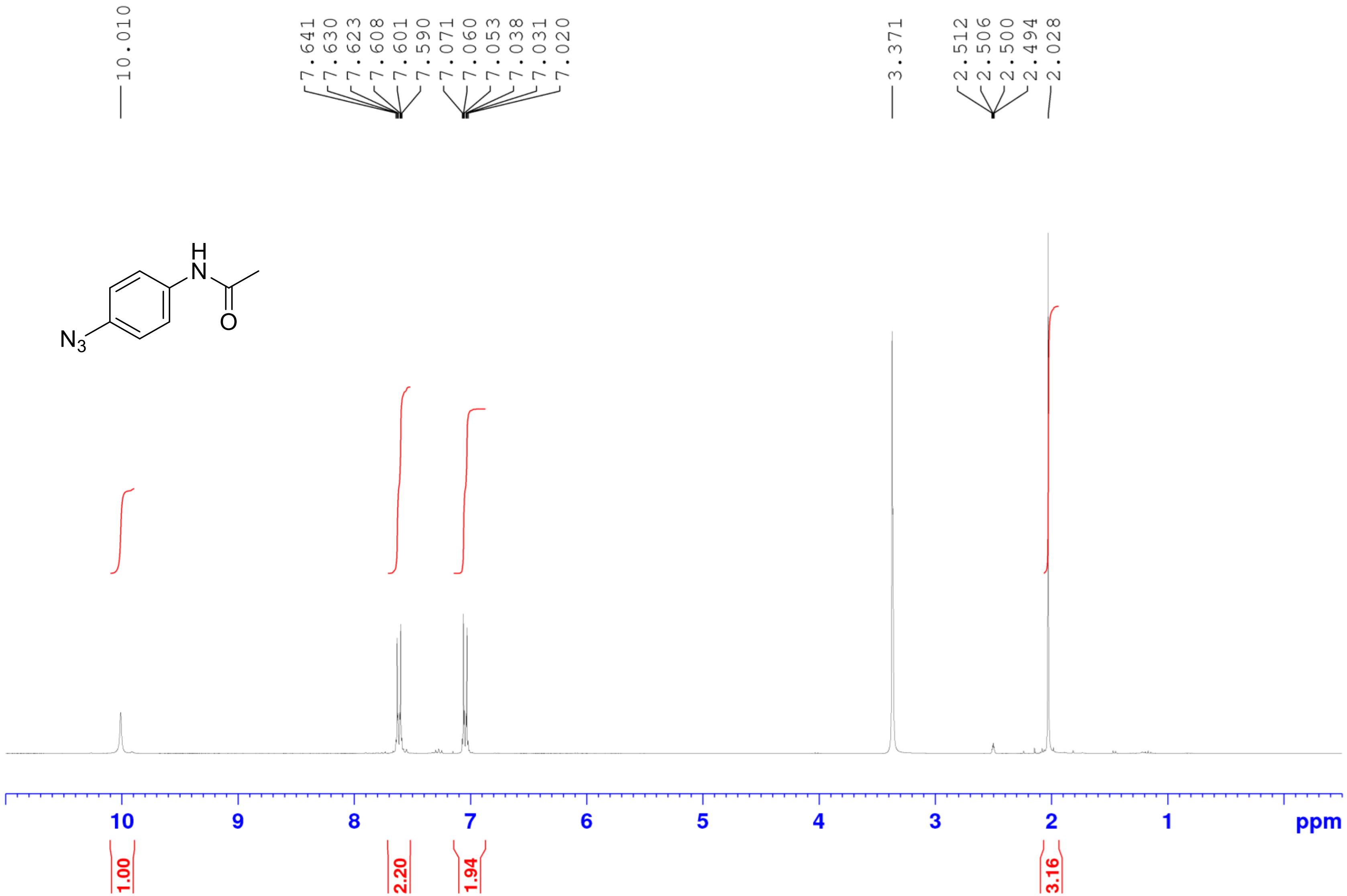

^{13}C NMR of compound **7u’** (75 MHz, DMSO-d_6)

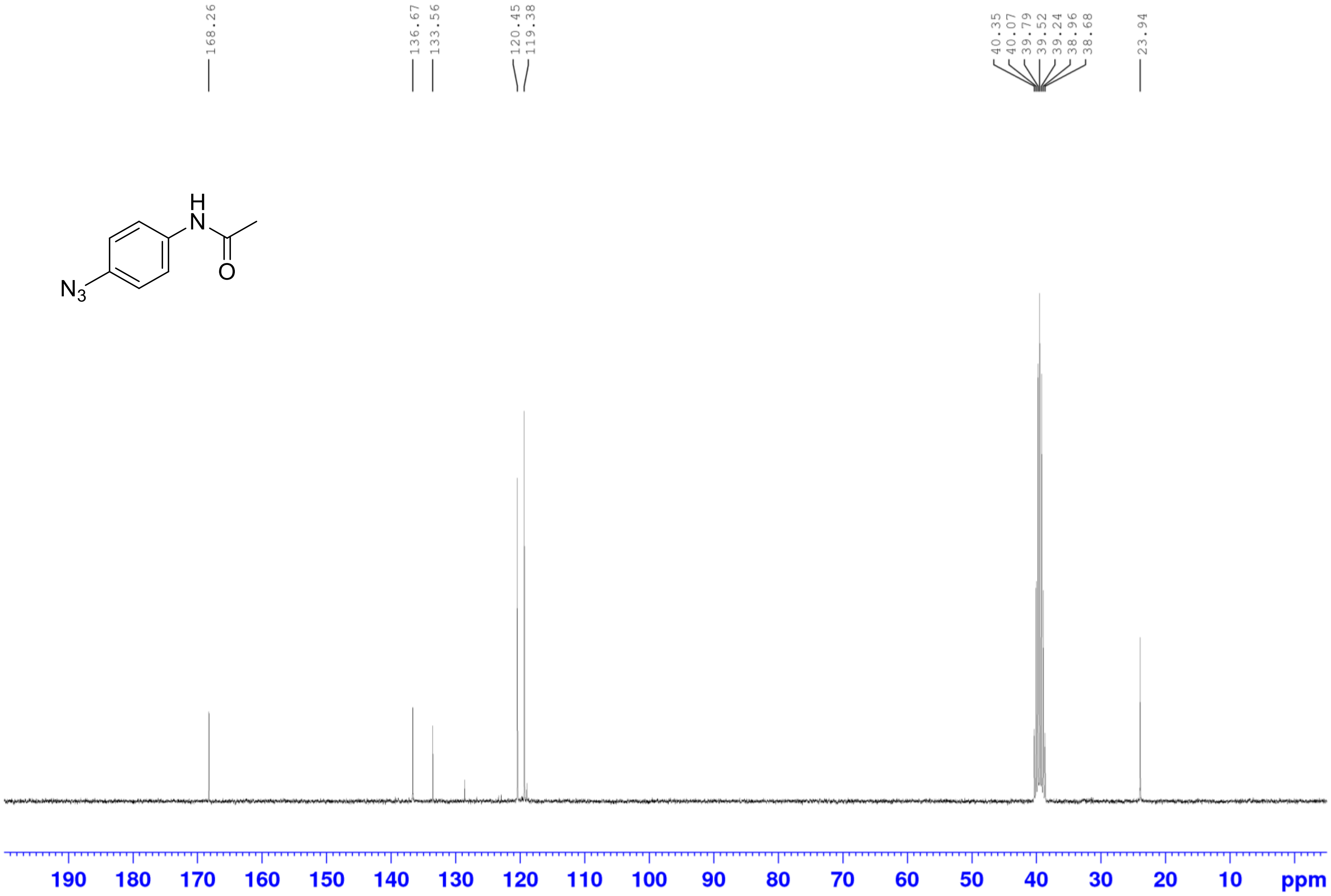

^{1}H NMR of compound **2a** (300 MHz, DMSO-d_6)

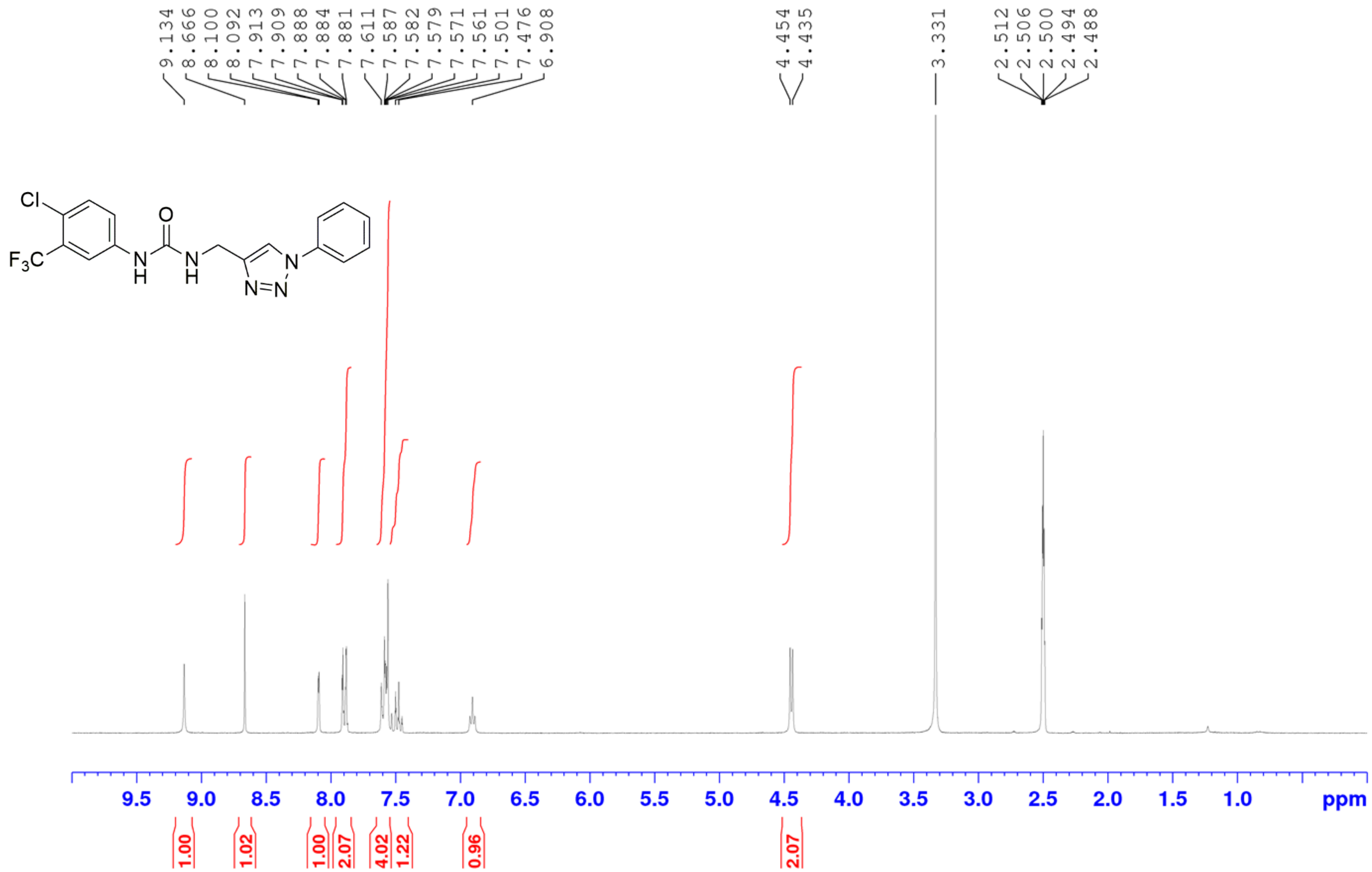

^{13}C NMR of compound **2a** (75 MHz, DMSO-d_6)

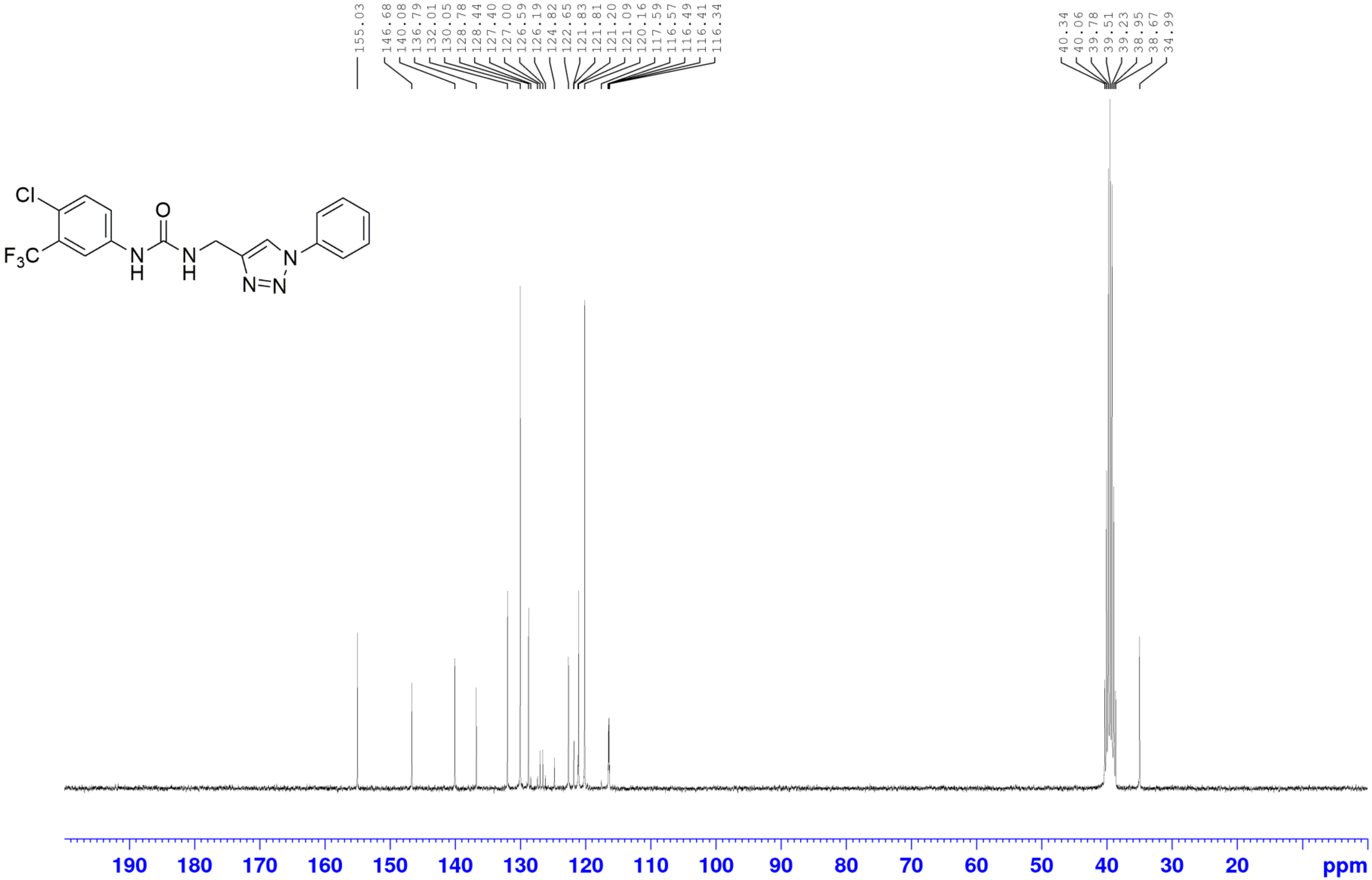

^{19}F NMR of compound **2a** (282 MHz, DMSO-d_6)

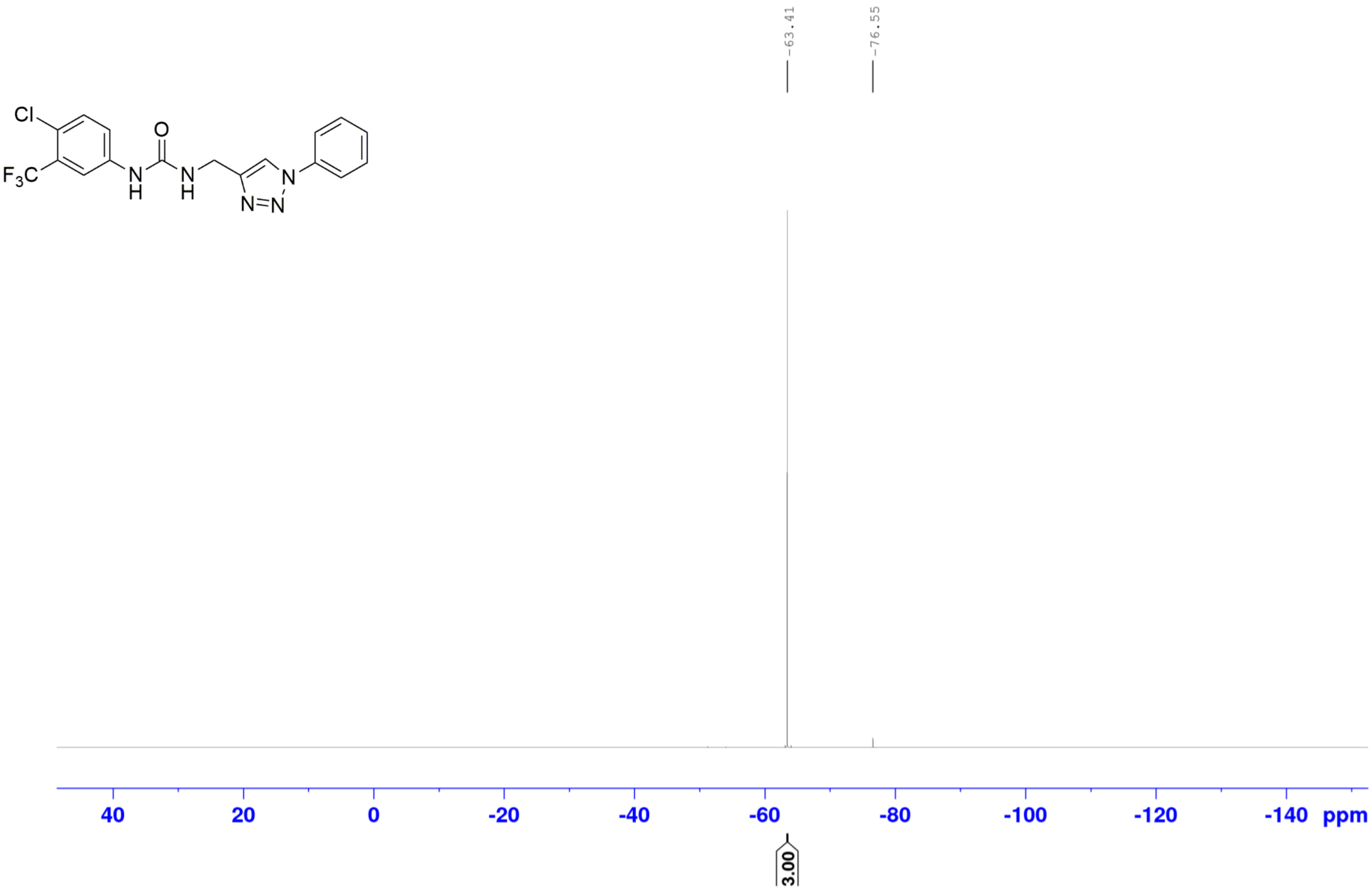

^{1}H NMR of compound **2b** (300 MHz, DMSO-d_6)

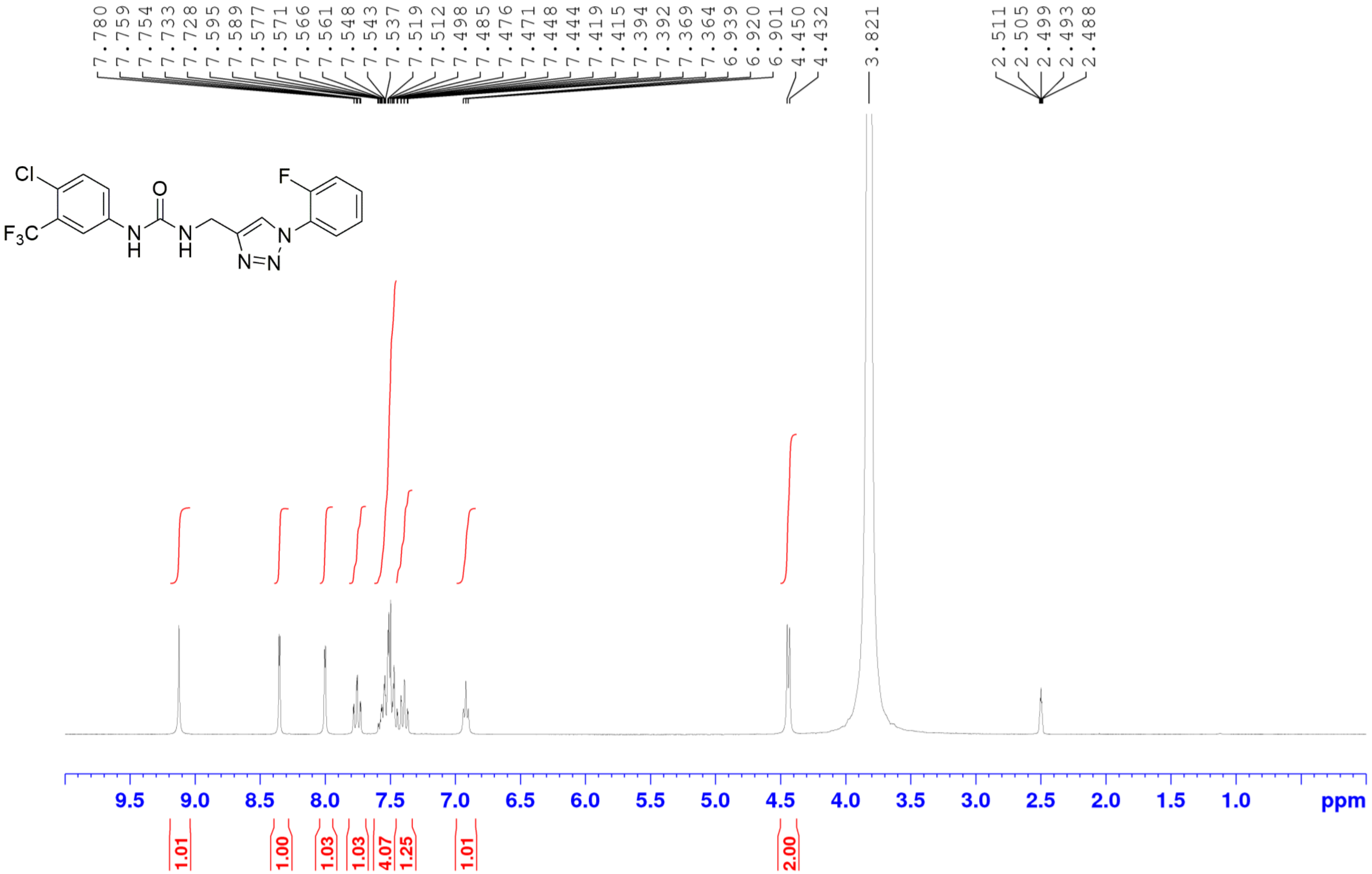

^{13}C NMR of compound **2b** (75 MHz, DMSO-d_6)

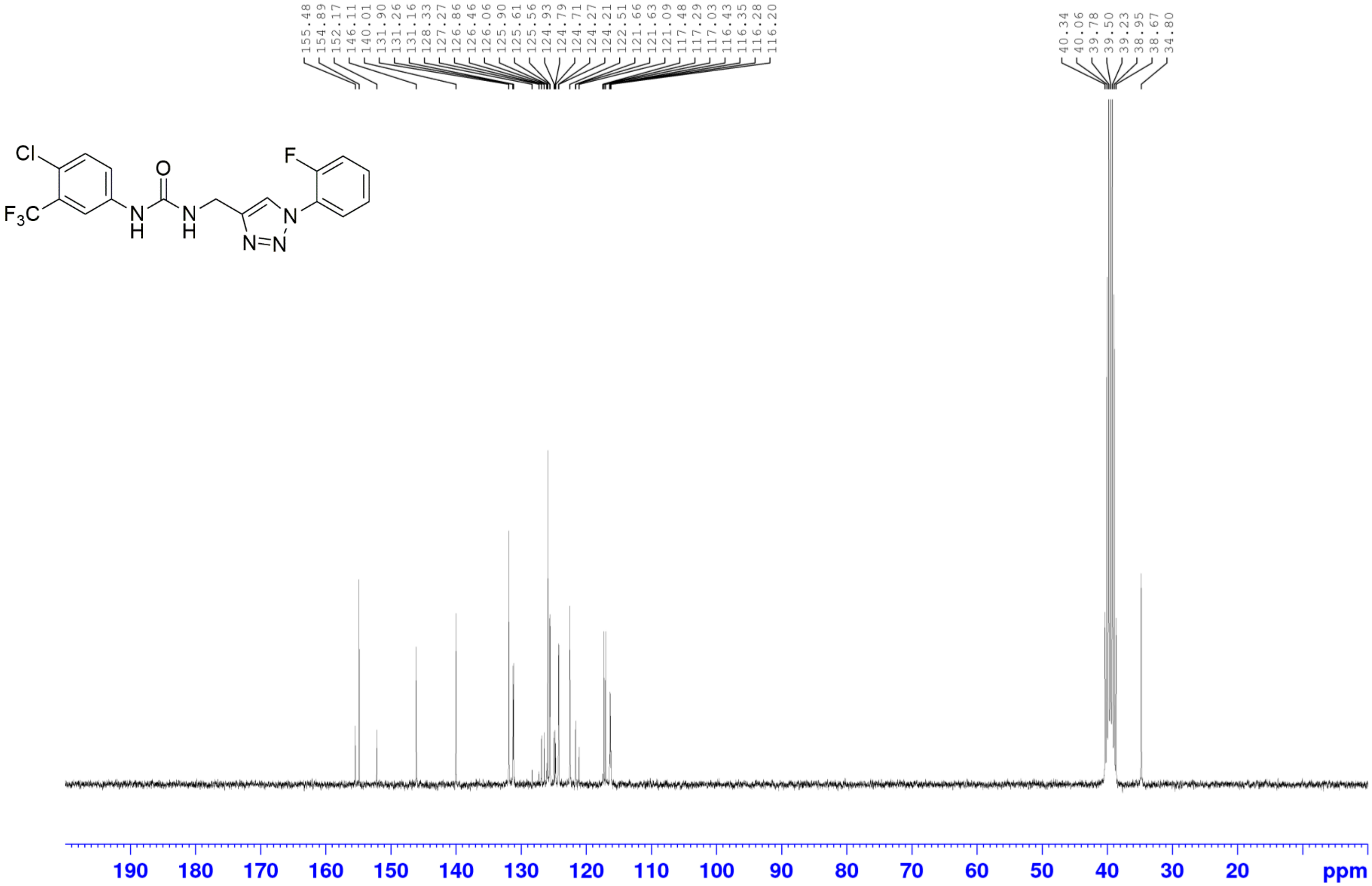

^{19}F NMR of compound **2b** (282 MHz, DMSO-*d*6)

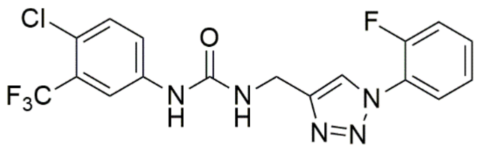

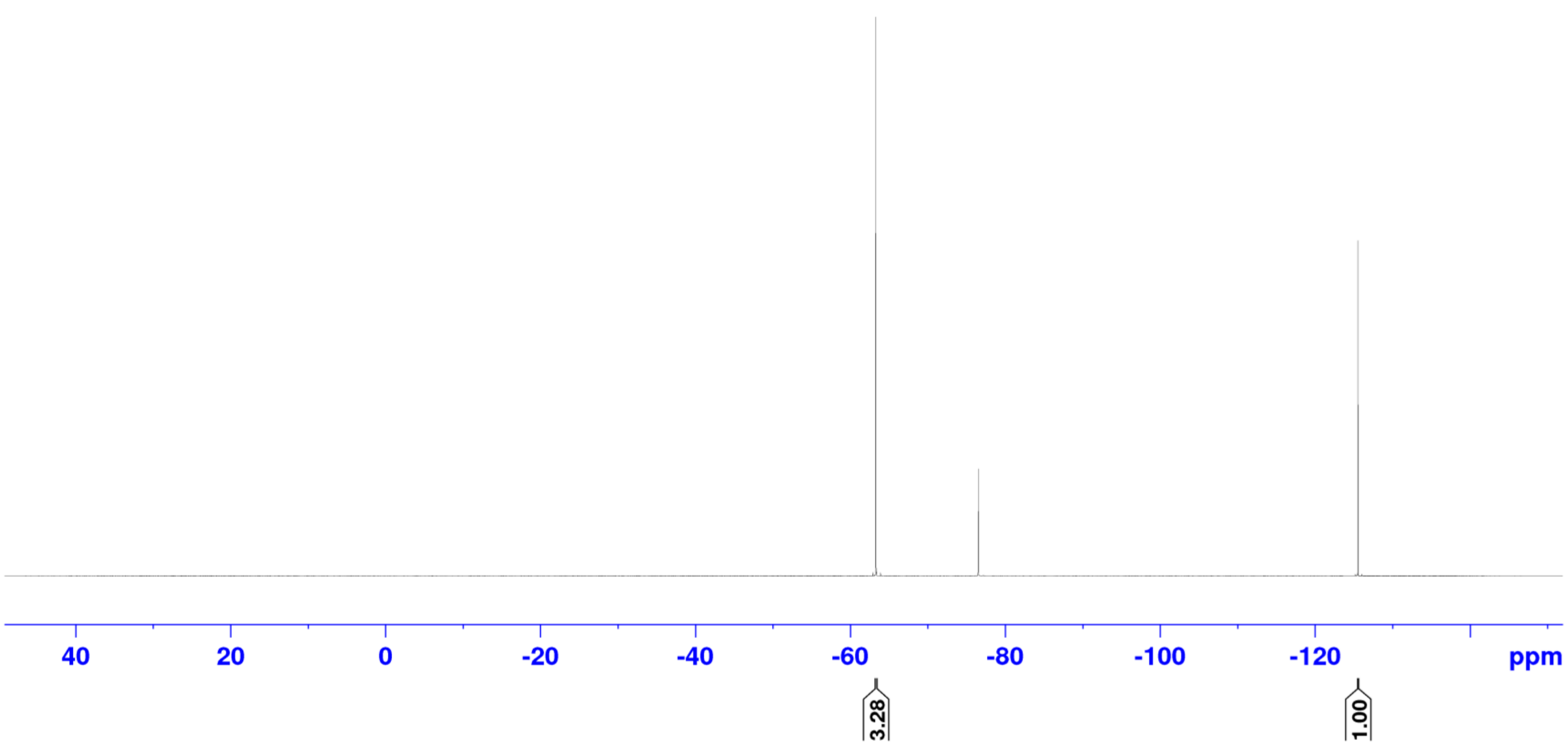

^{1}H NMR of compound **2c** (300 MHz, DMSO-d_6)

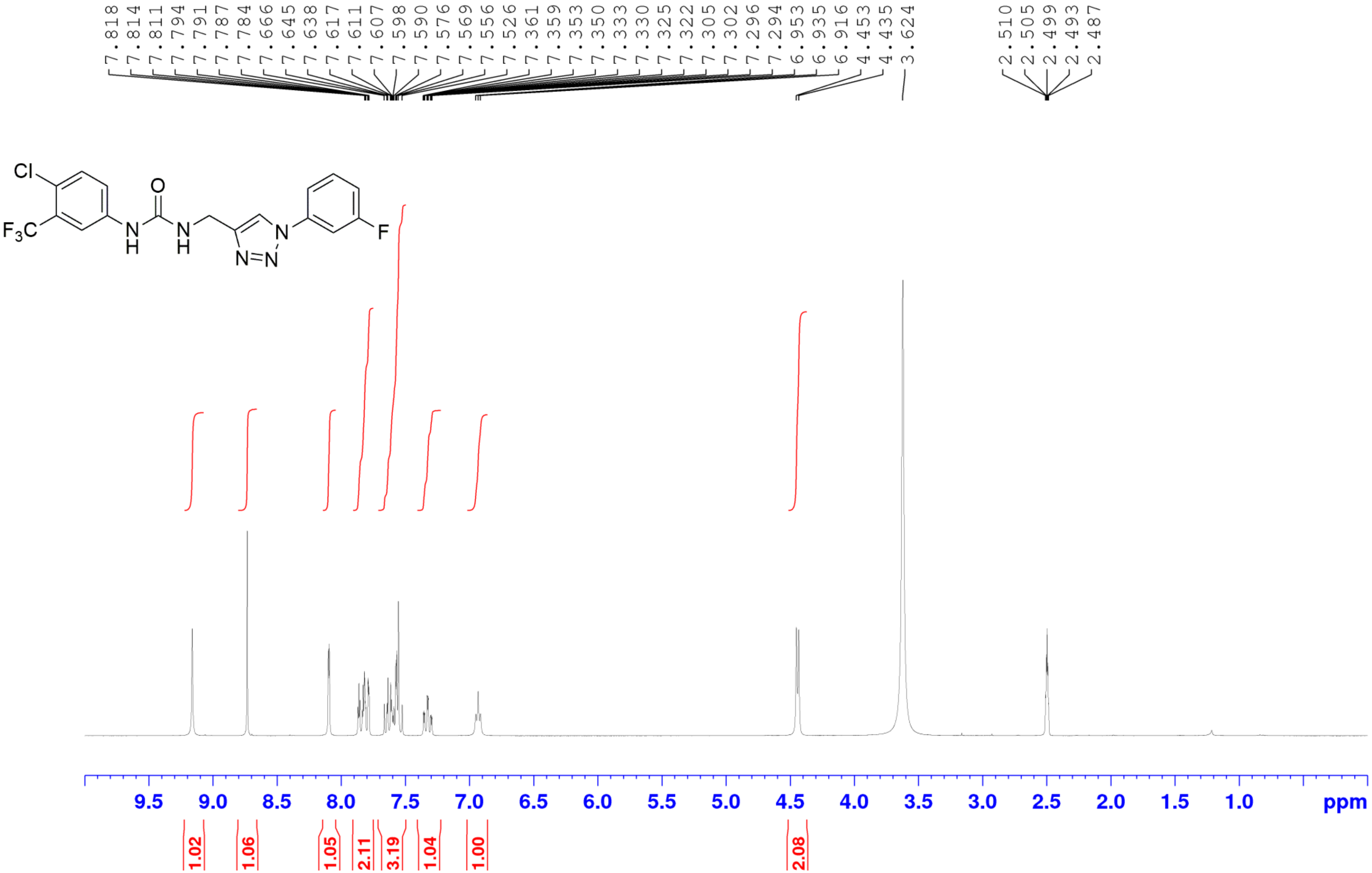

^{13}C NMR of compound **2c** (75 MHz, DMSO-d_6)

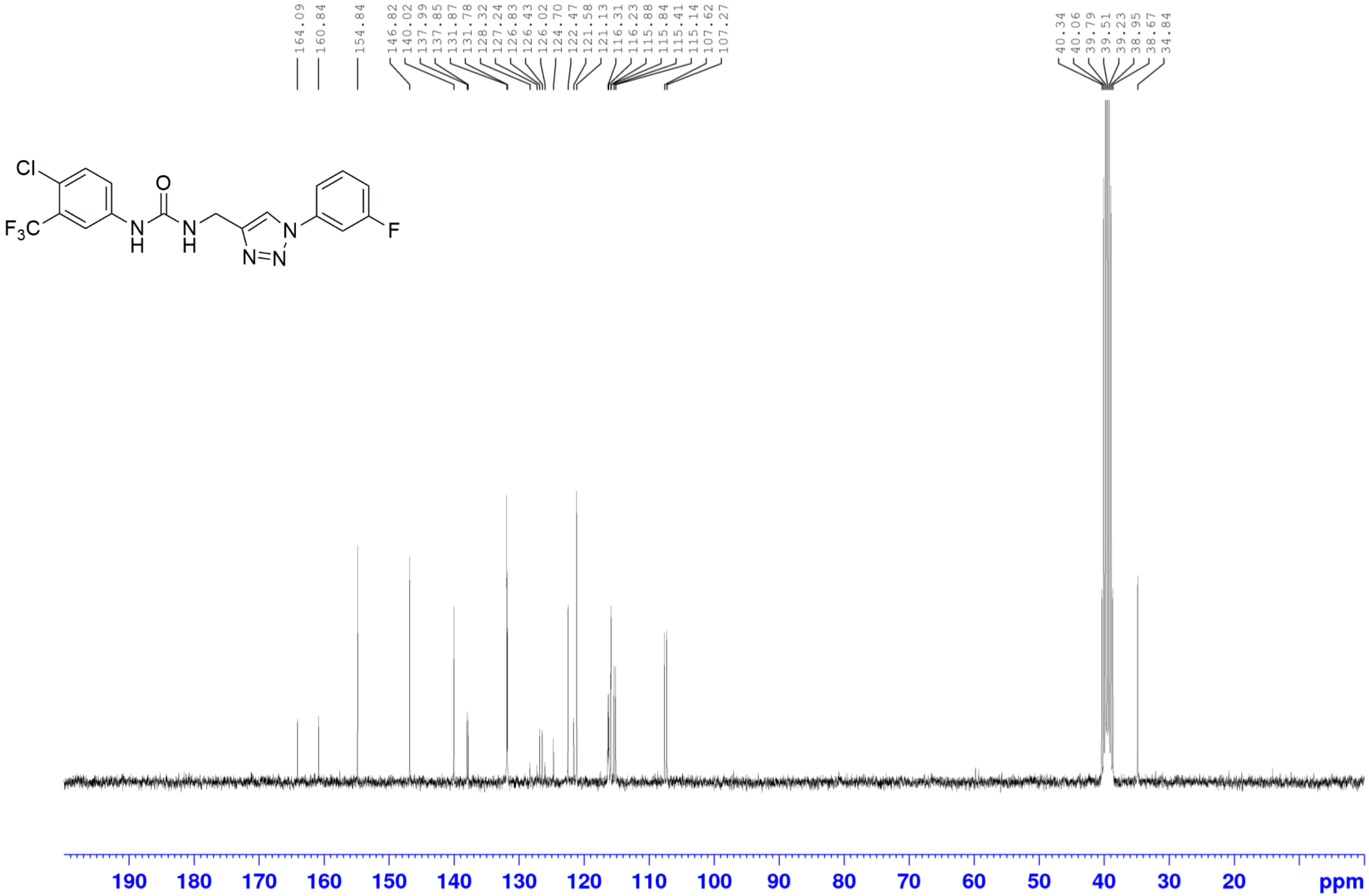

^{19}F NMR of compound **2c** (282 MHz, DMSO-d_6)

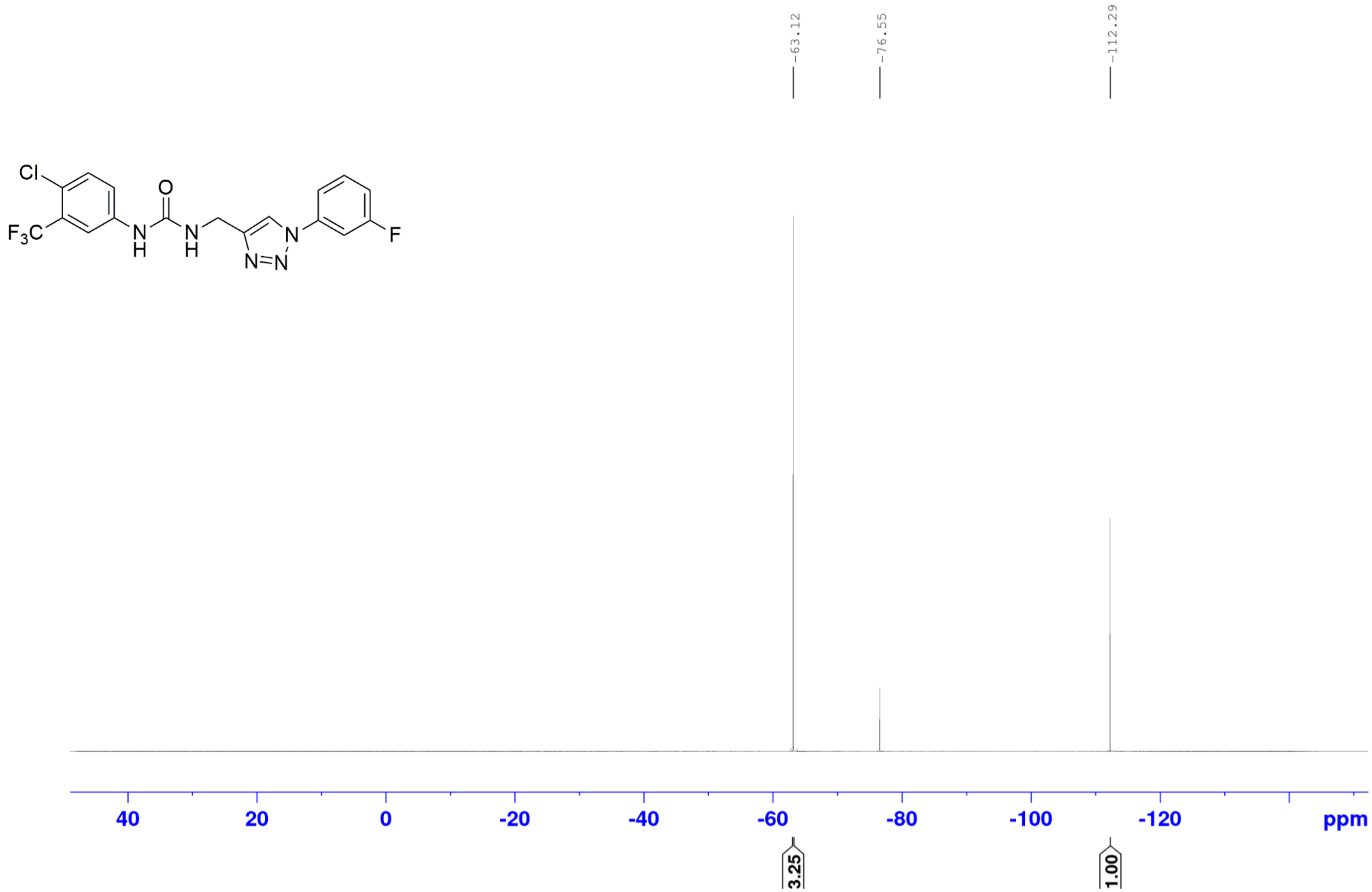

^{1}H NMR of compound **2d** (300 MHz, DMSO-d_6)

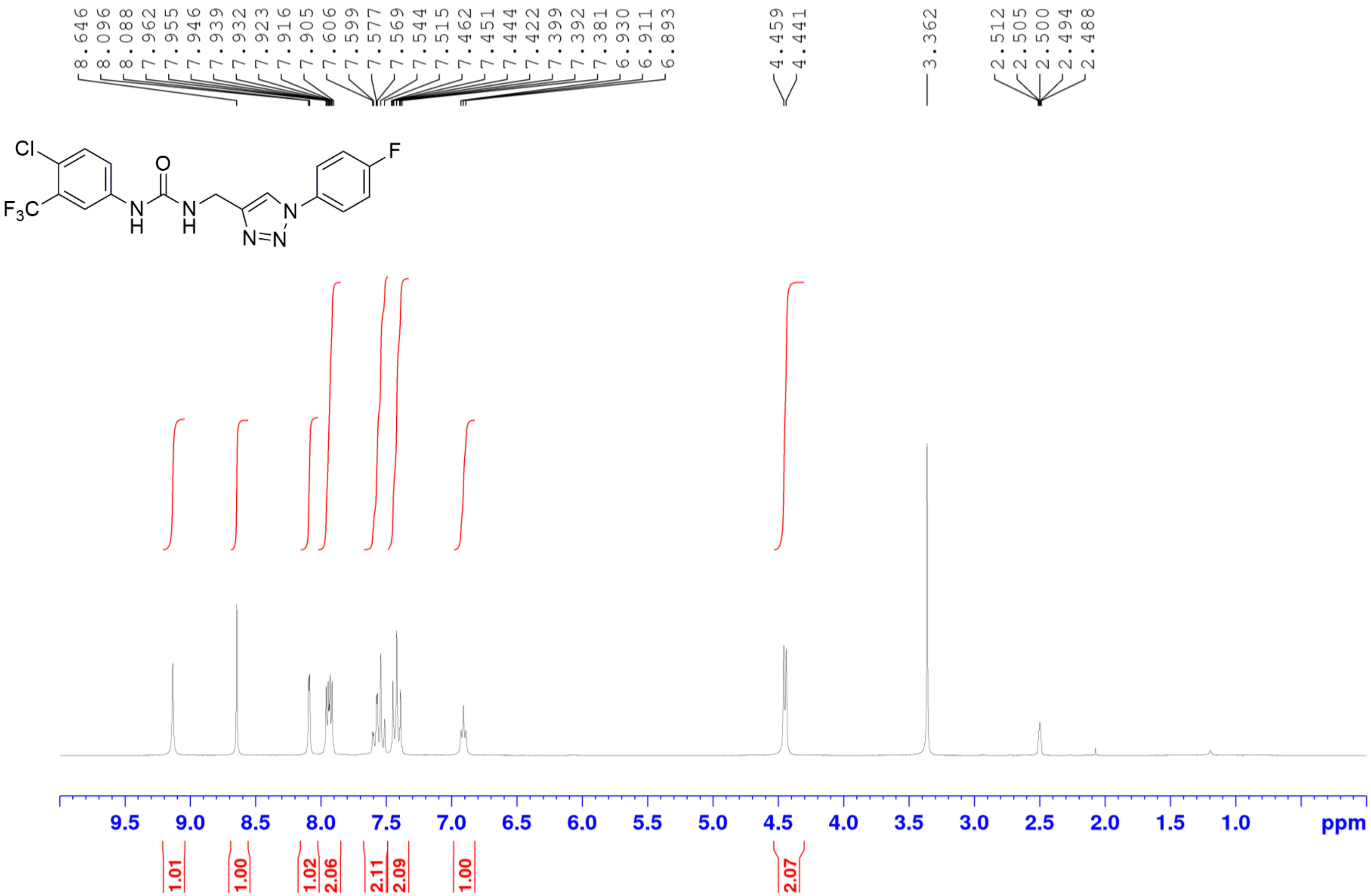

^{13}C NMR of compound **2d** (75 MHz, DMSO-d_6)

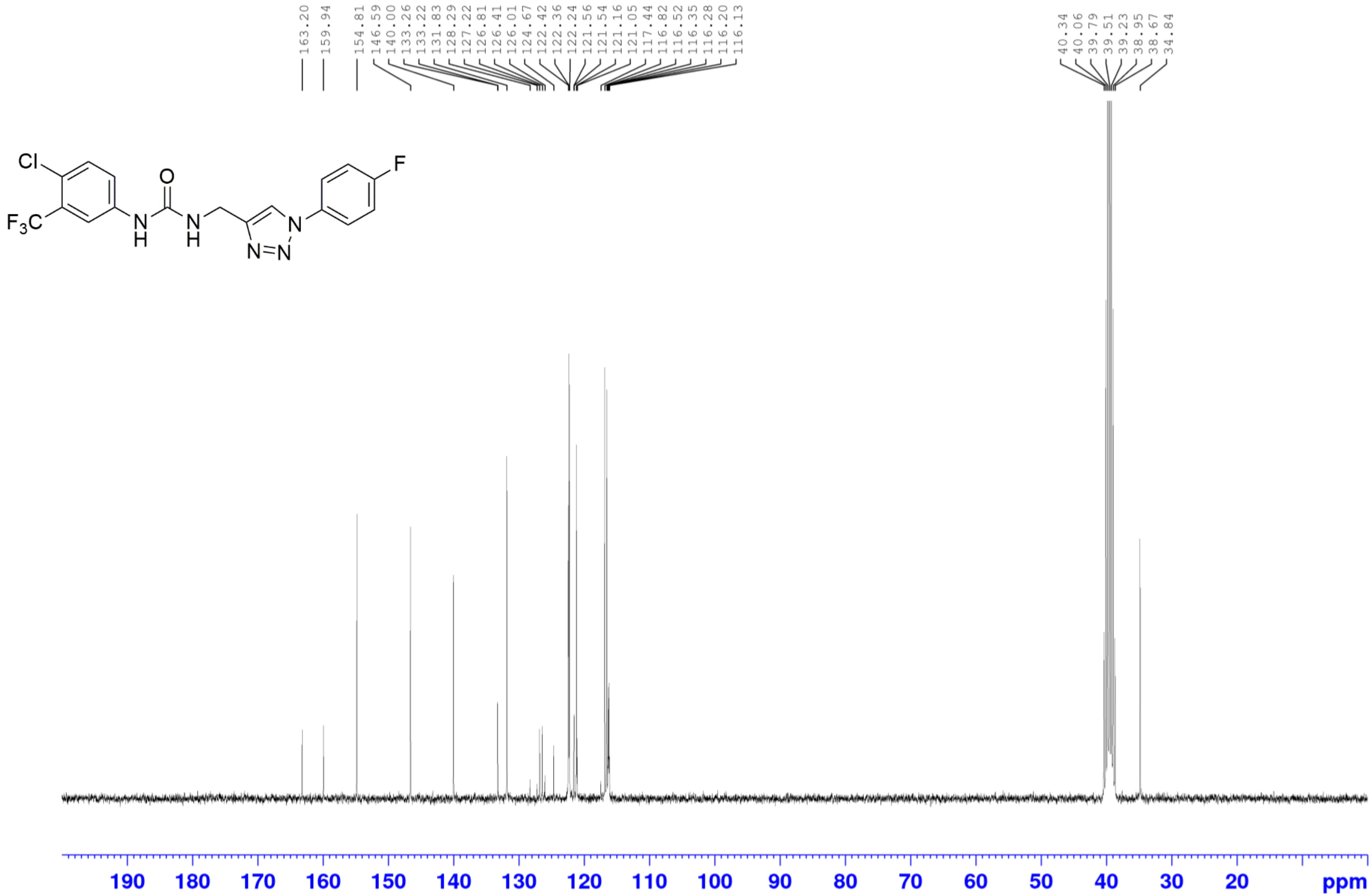

^{19}F NMR of compound **2d** (282 MHz, DMSO-d_6)

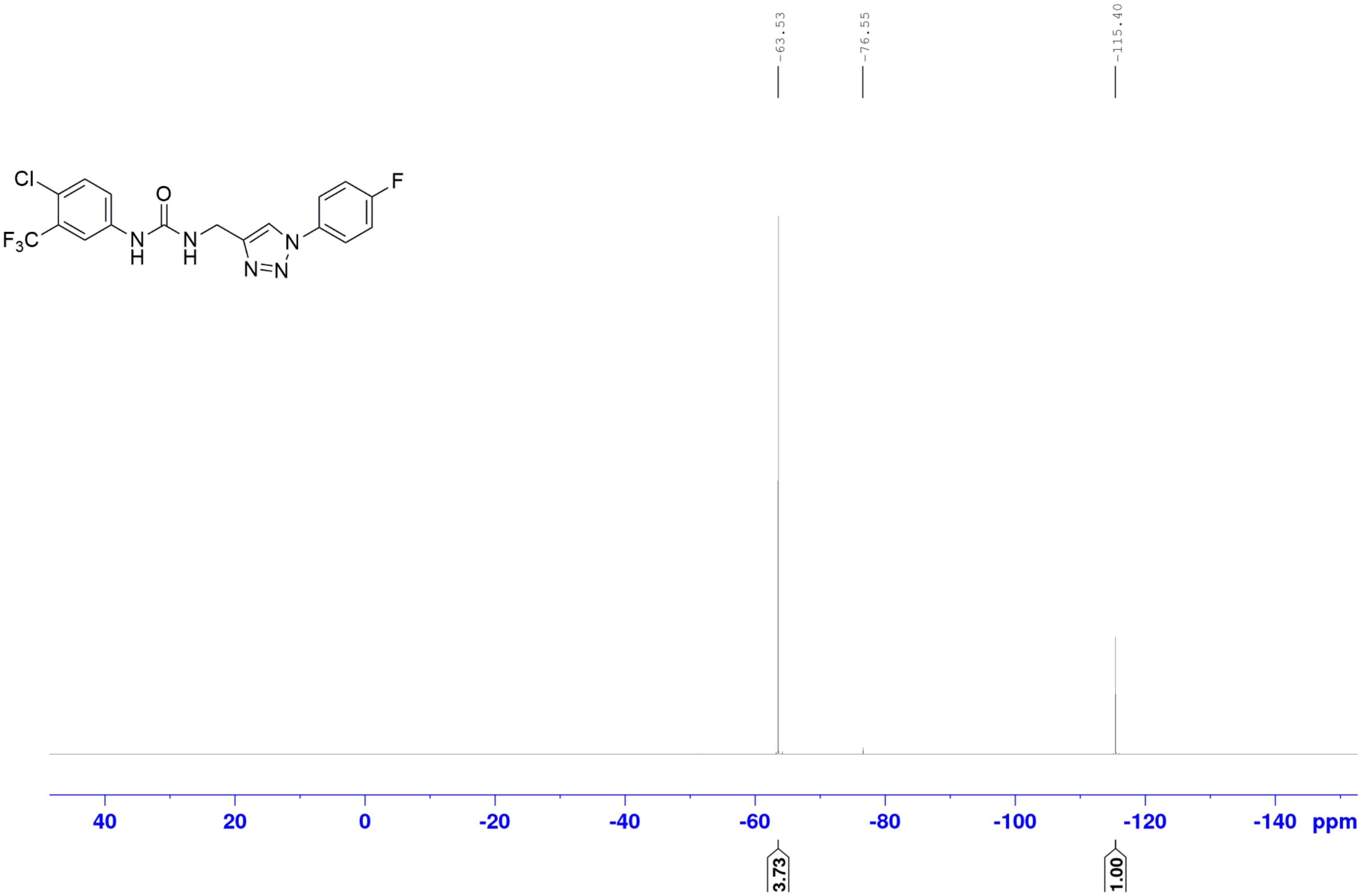

^{1}H NMR of compound **2e** (300 MHz, DMSO-d_6)

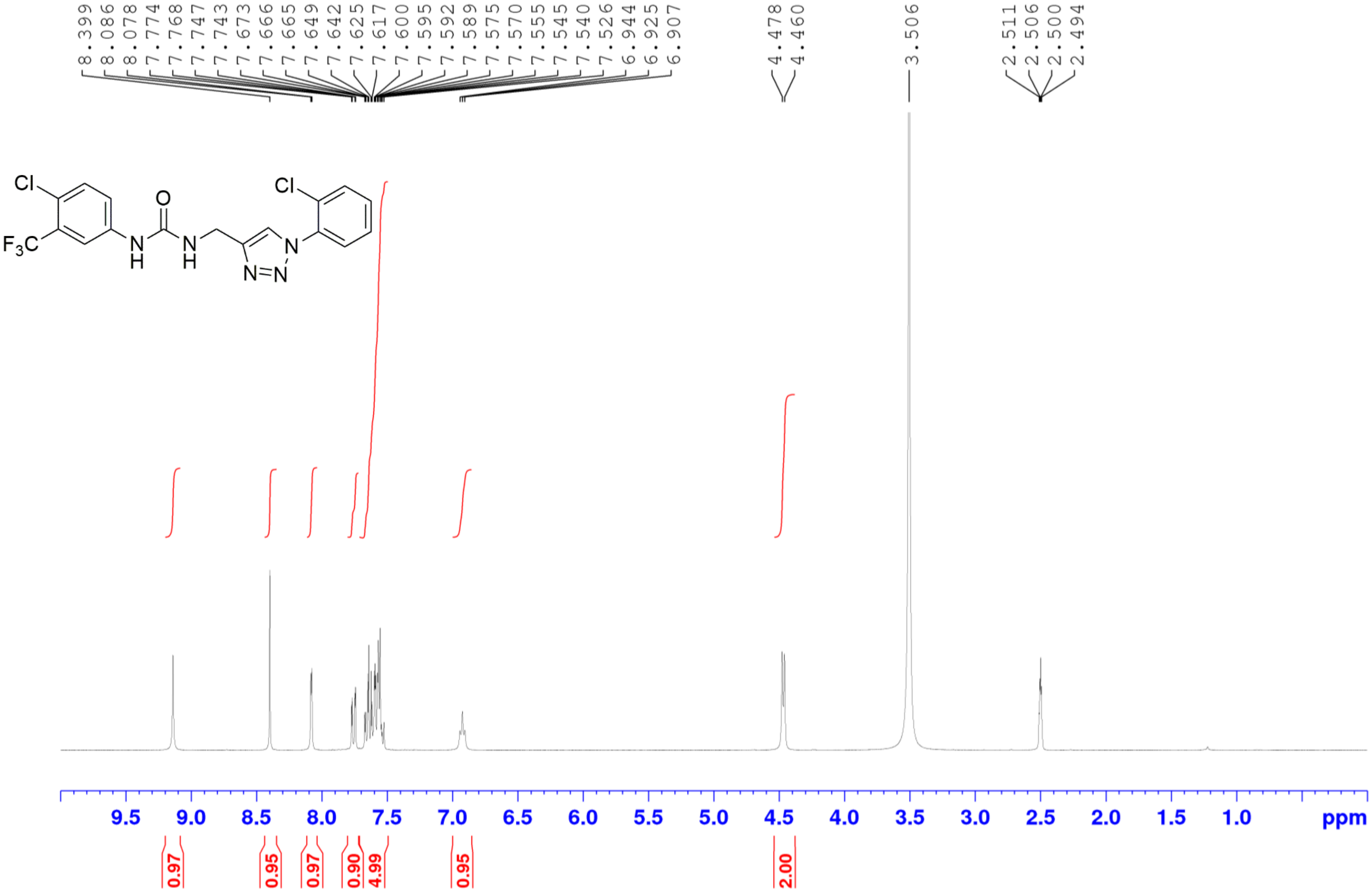

^{13}C NMR of compound **2e** (75 MHz, DMSO-d_6)

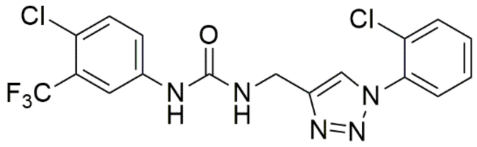

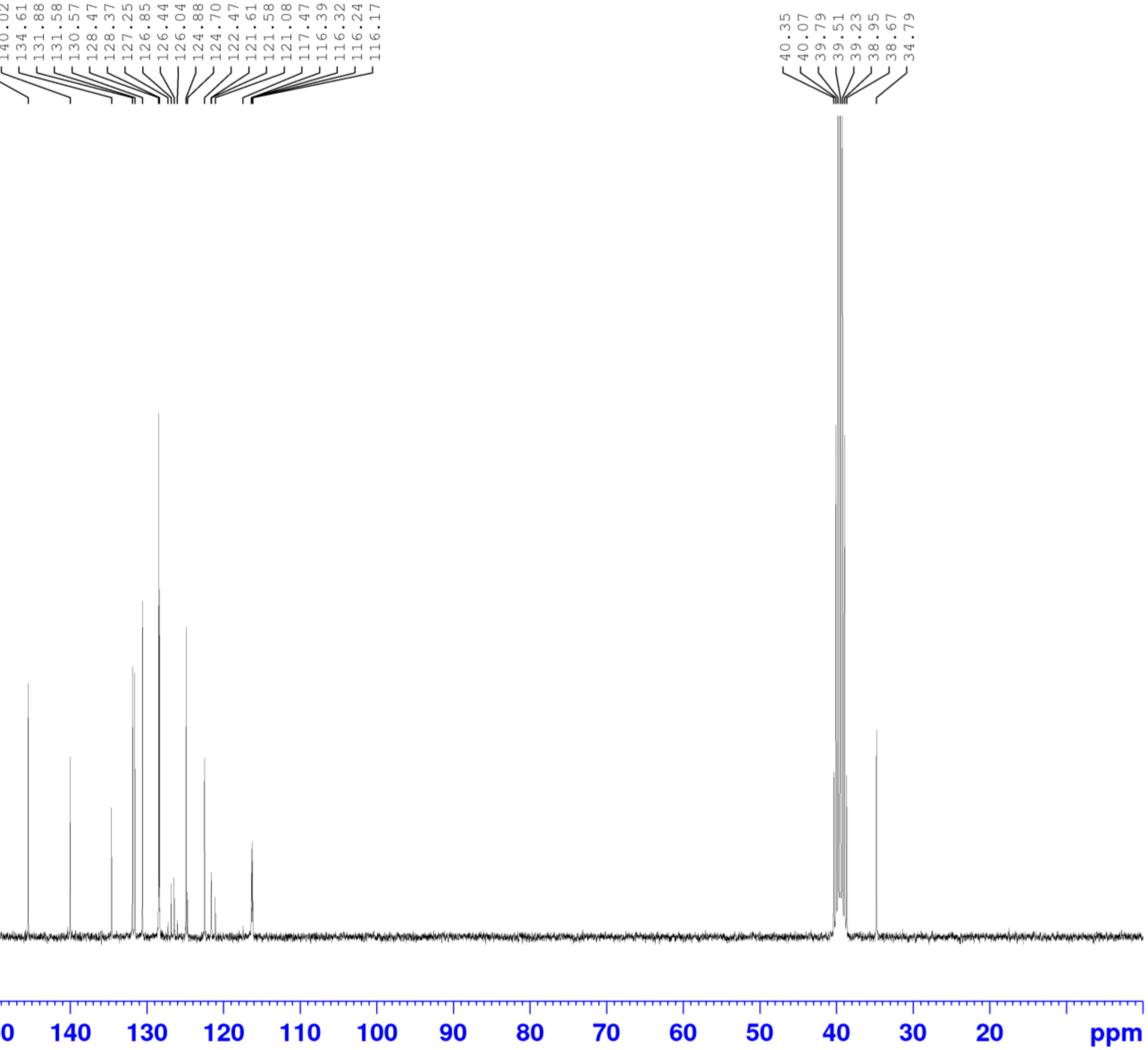

^{19}F NMR of compound **2e** (282 MHz, DMSO-d_6)

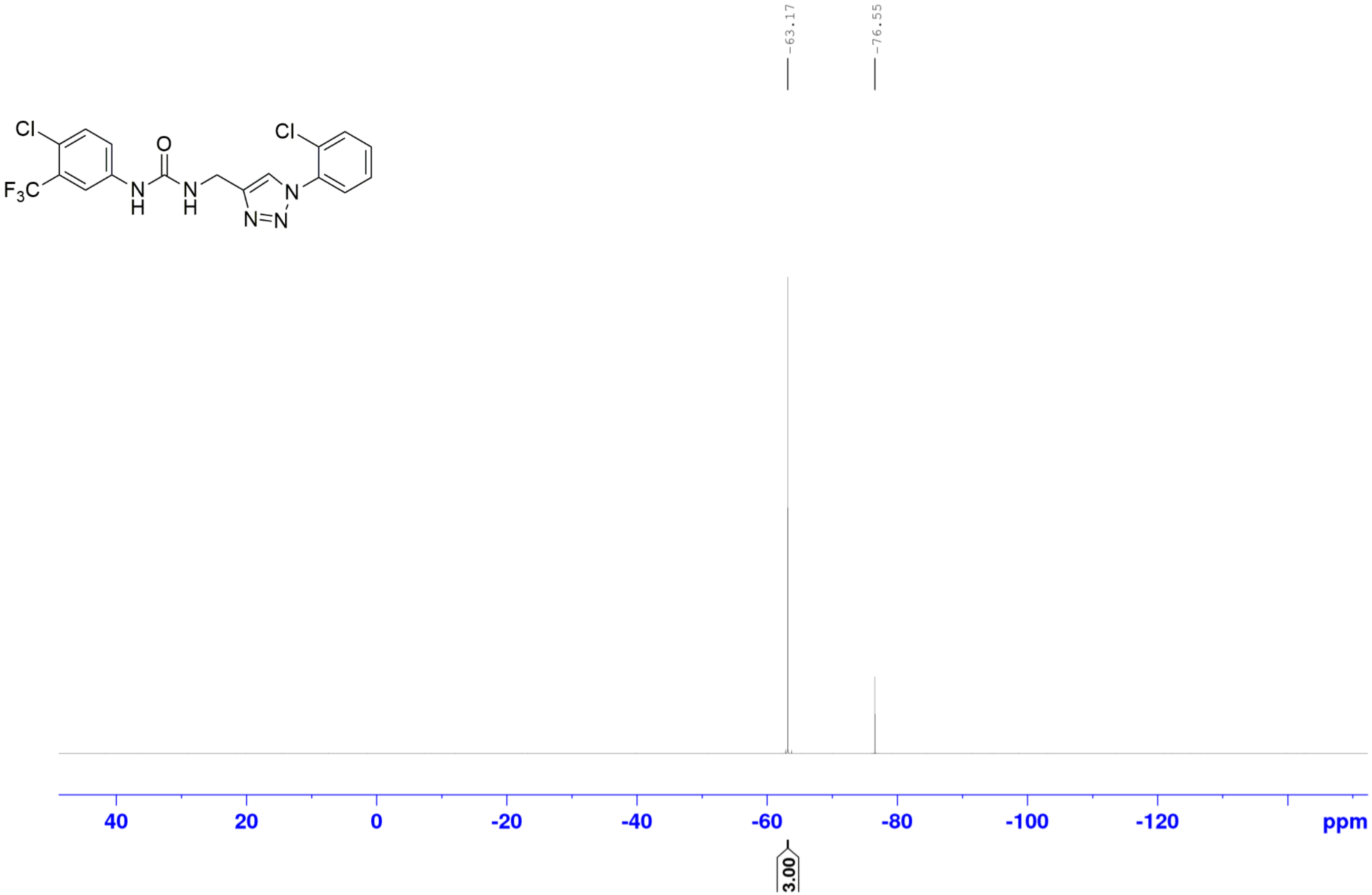

^{1}H NMR of compound **2f** (300 MHz, DMSO-d_6)

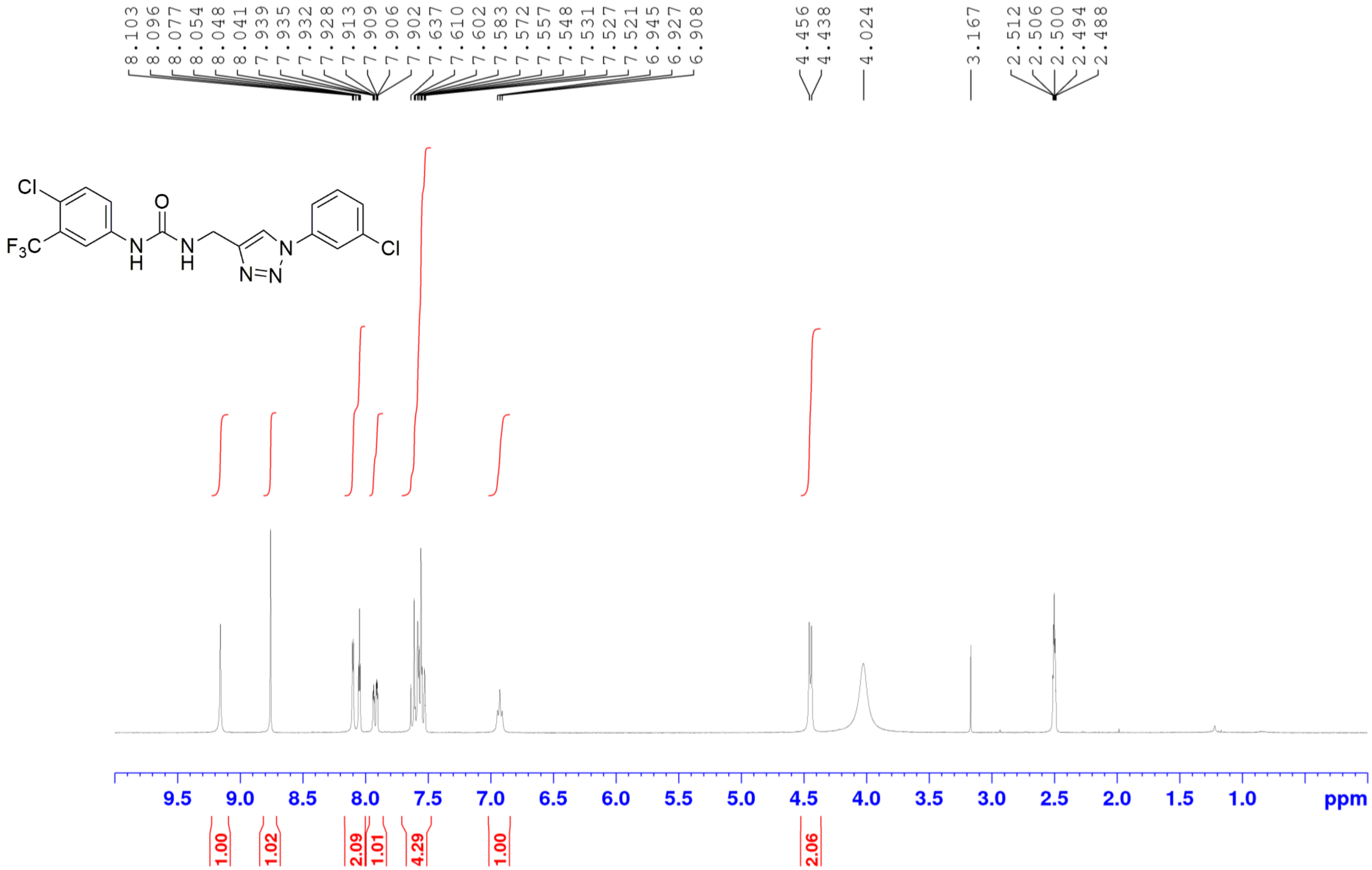

^{13}C NMR of compound **2f** (75 MHz, DMSO-*d*6)

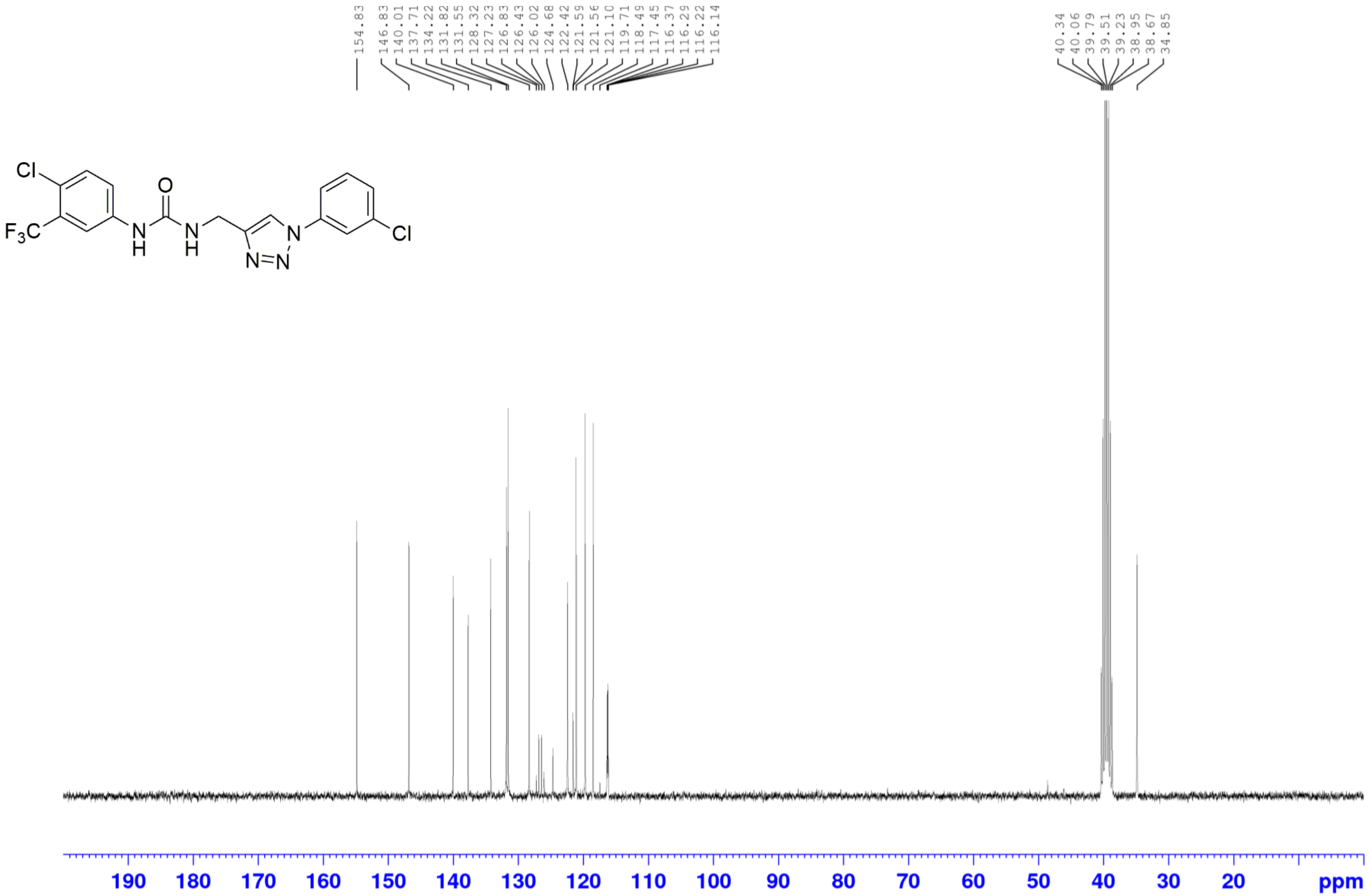

^{19}F NMR of compound **2f** (282 MHz, DMSO-d_6)

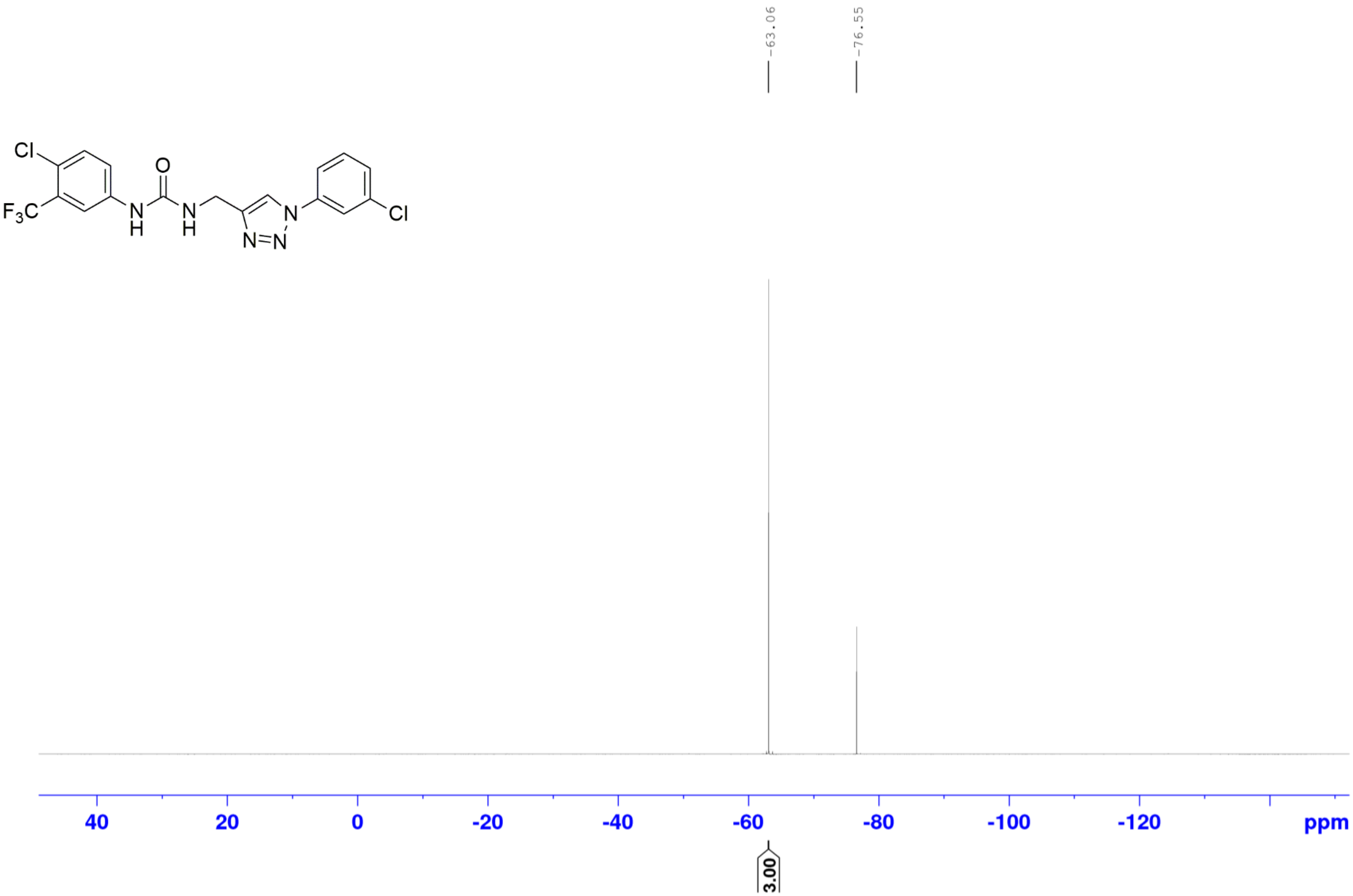

^{1}H NMR of compound **2g** (300 MHz, DMSO-d_6)

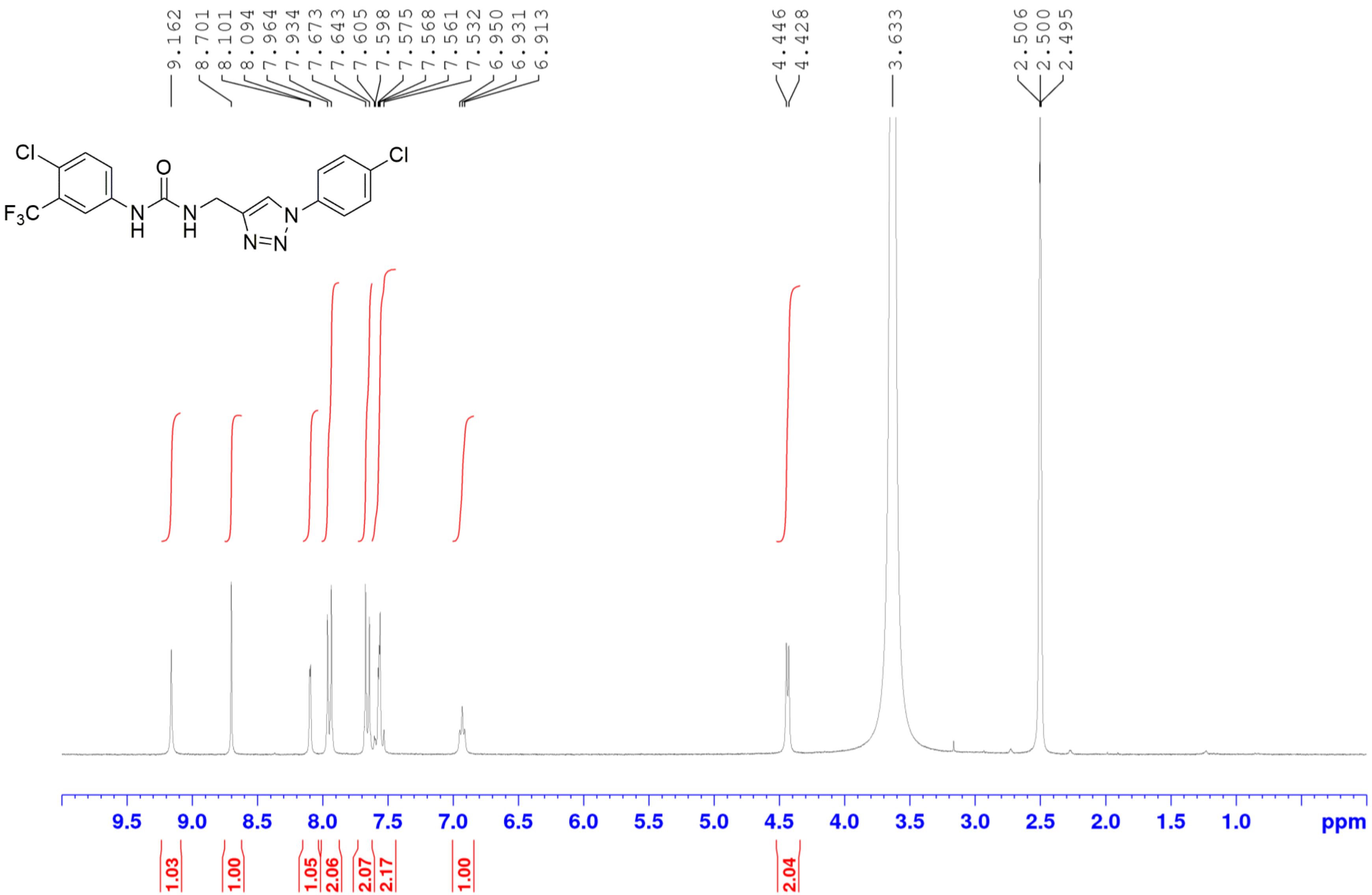

^{13}C NMR of compound **2g** (75 MHz, DMSO-d_6)

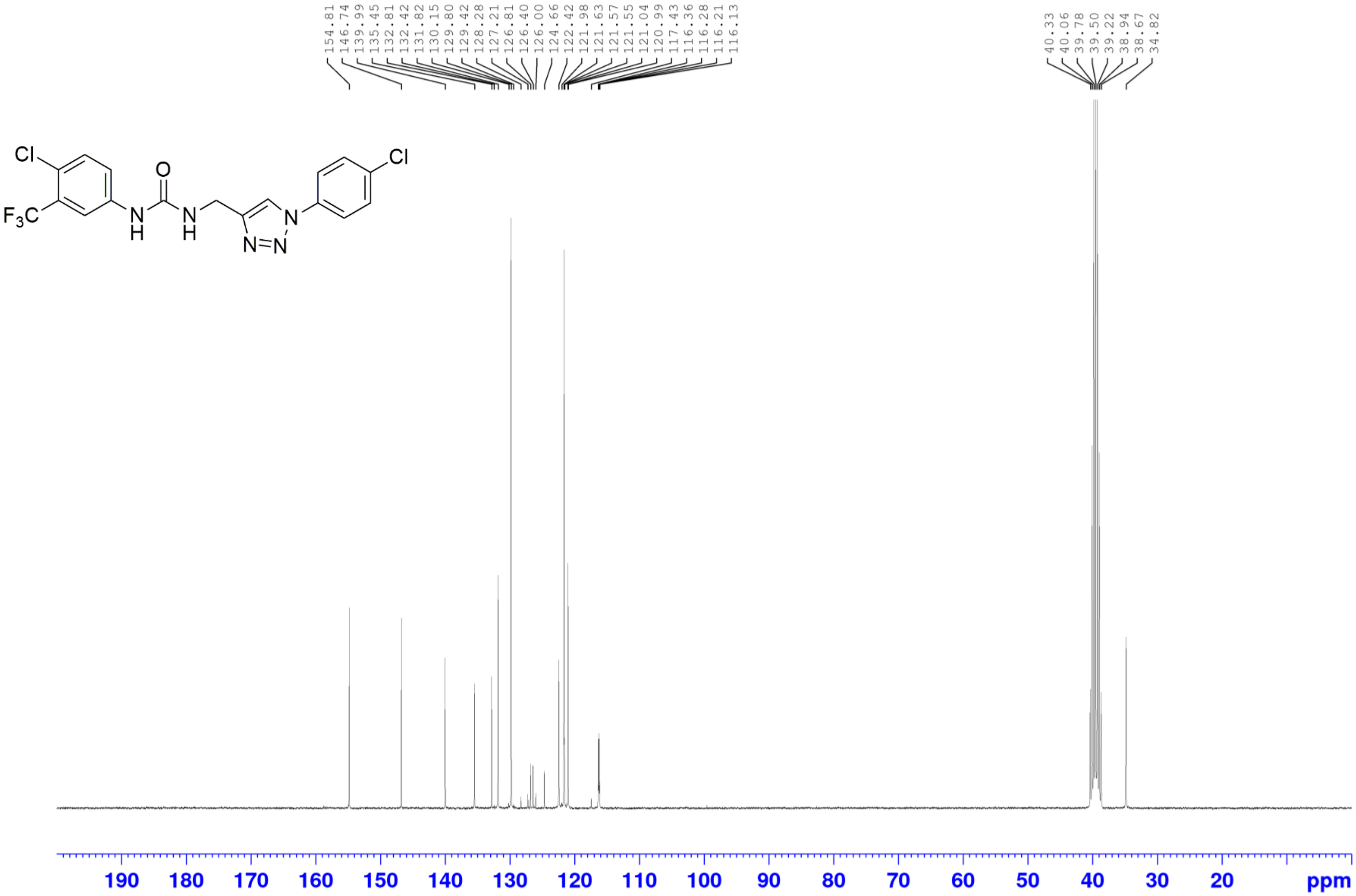

^{19}F NMR of compound **2g** (282 MHz, DMSO-d_6)

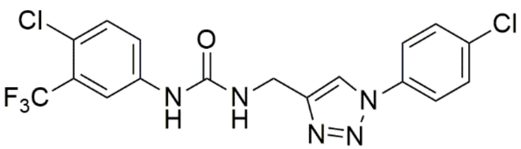

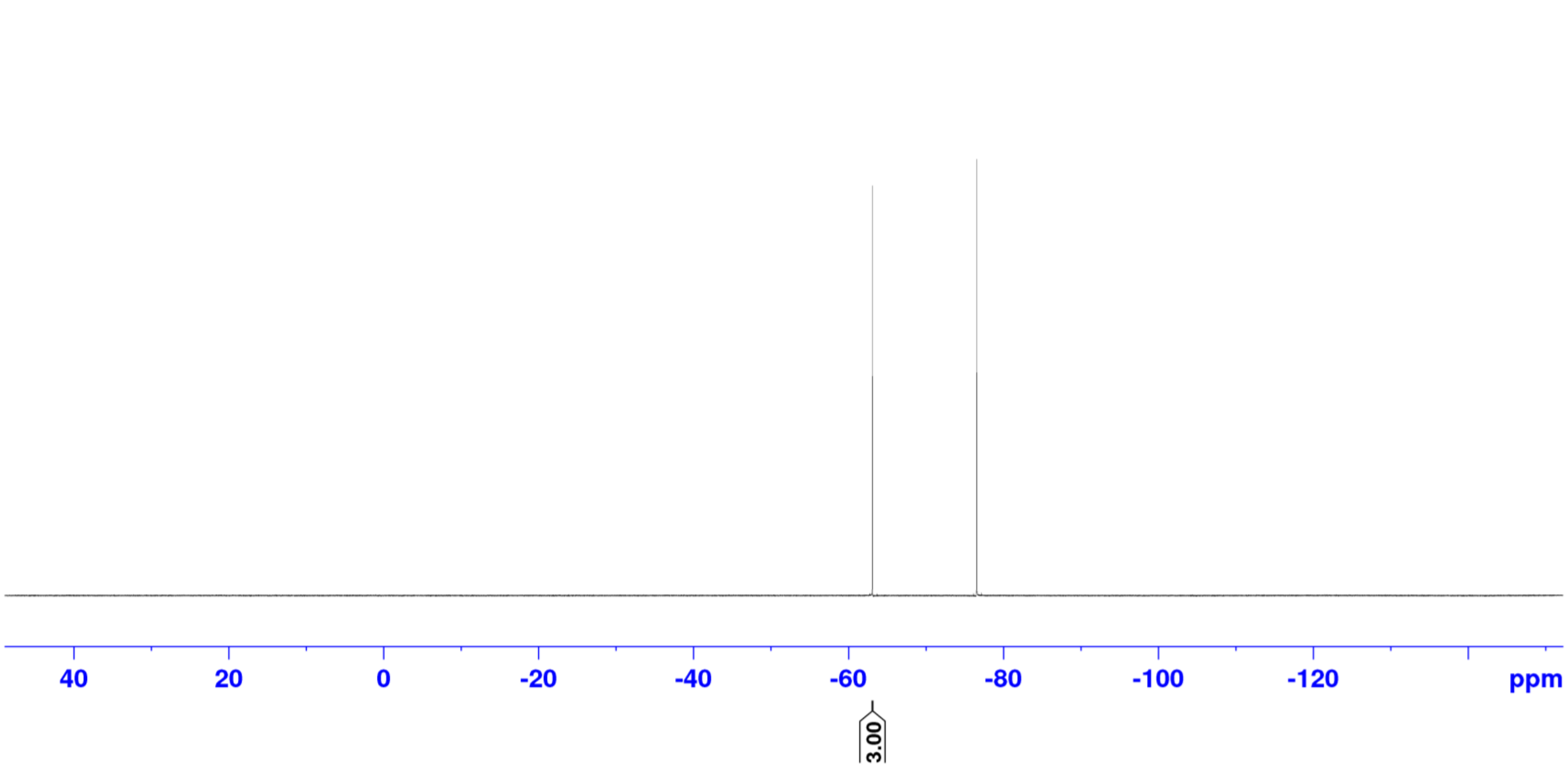

^{1}H NMR of compound **2h** (300 MHz, DMSO-d_6)

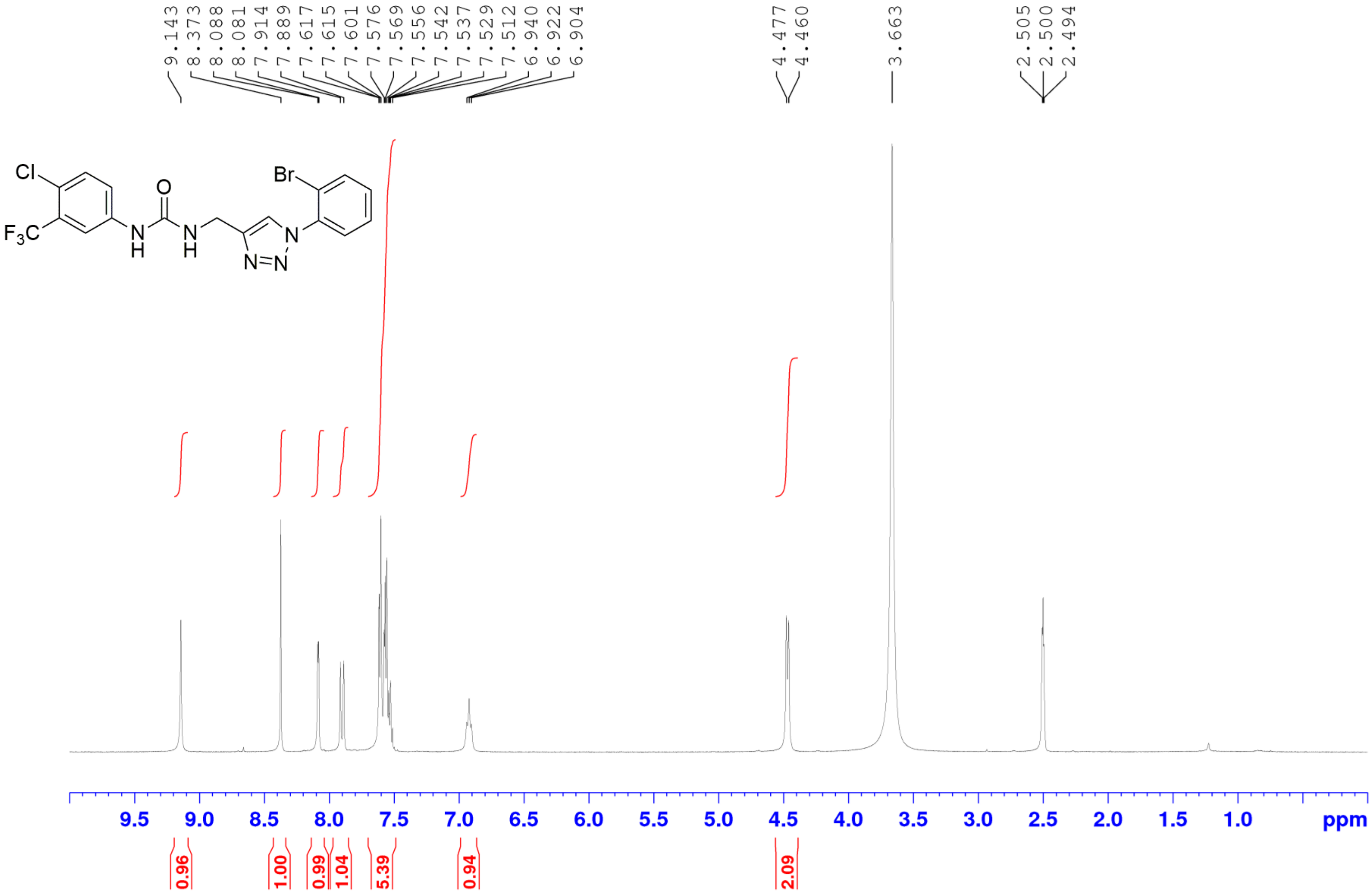

^{13}C NMR of compound **2h** (75 MHz, DMSO-d_6)

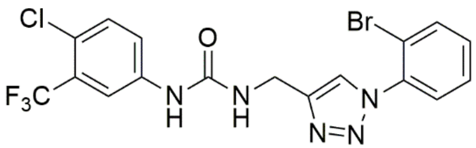

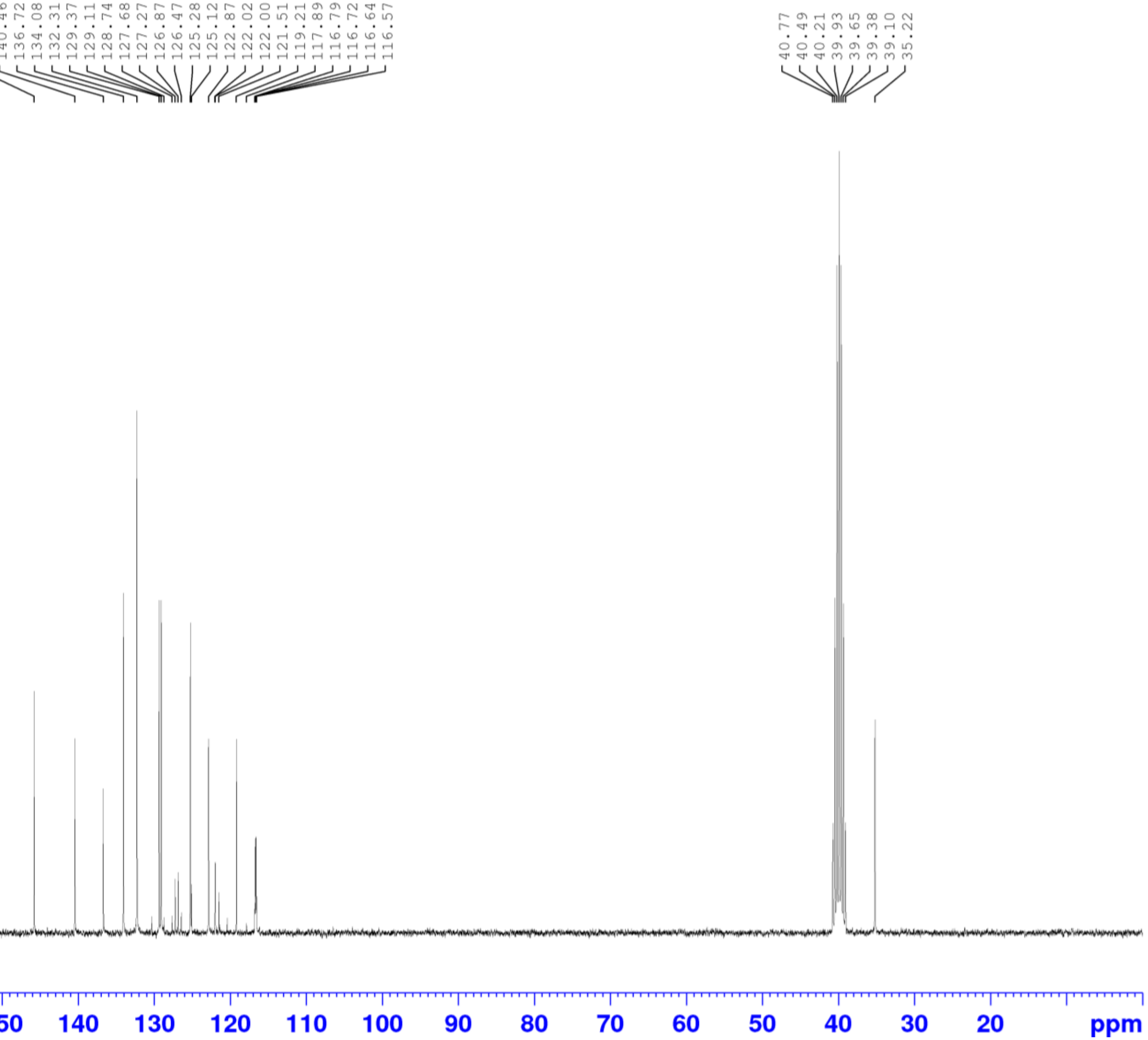

^{19}F NMR of compound **2h** (282 MHz, DMSO-d_6)

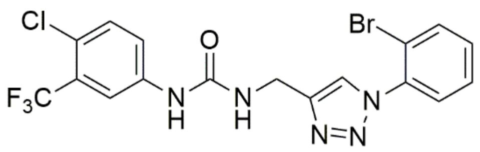

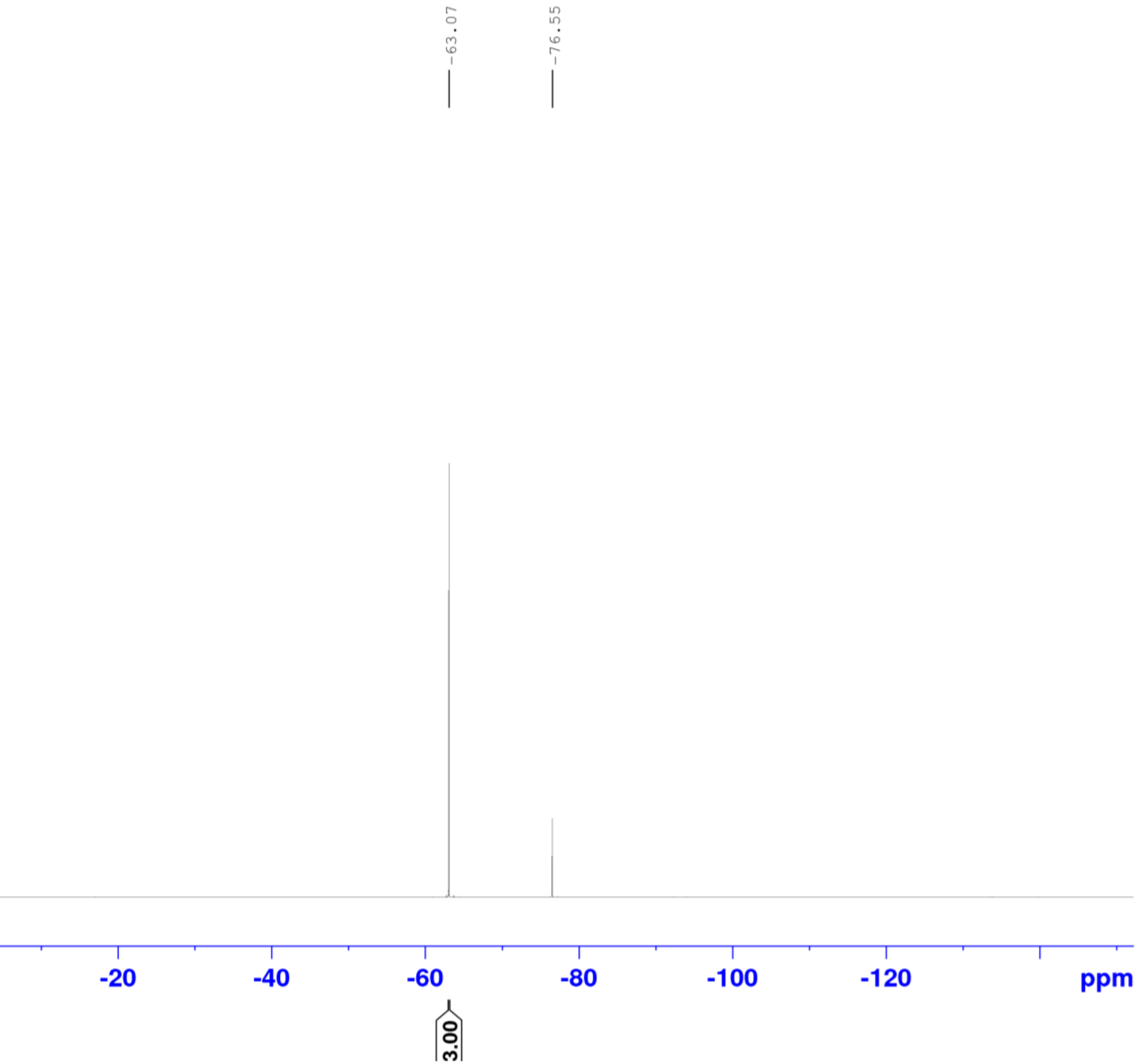

^{1}H NMR of compound **2i** (300 MHz, DMSO-*d*$_6$)

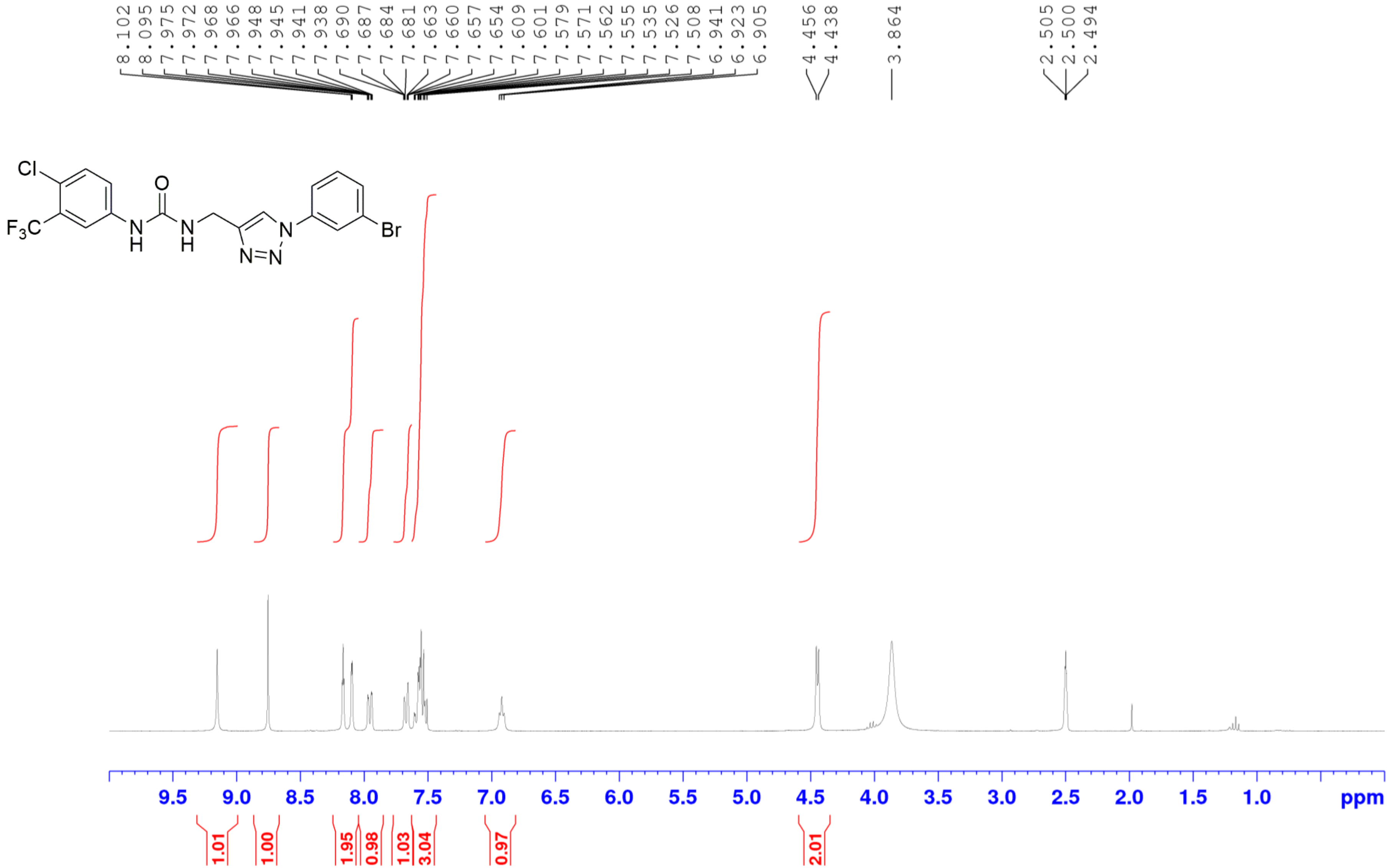

^{13}C NMR of compound **2i** (75 MHz, DMSO-d_6)

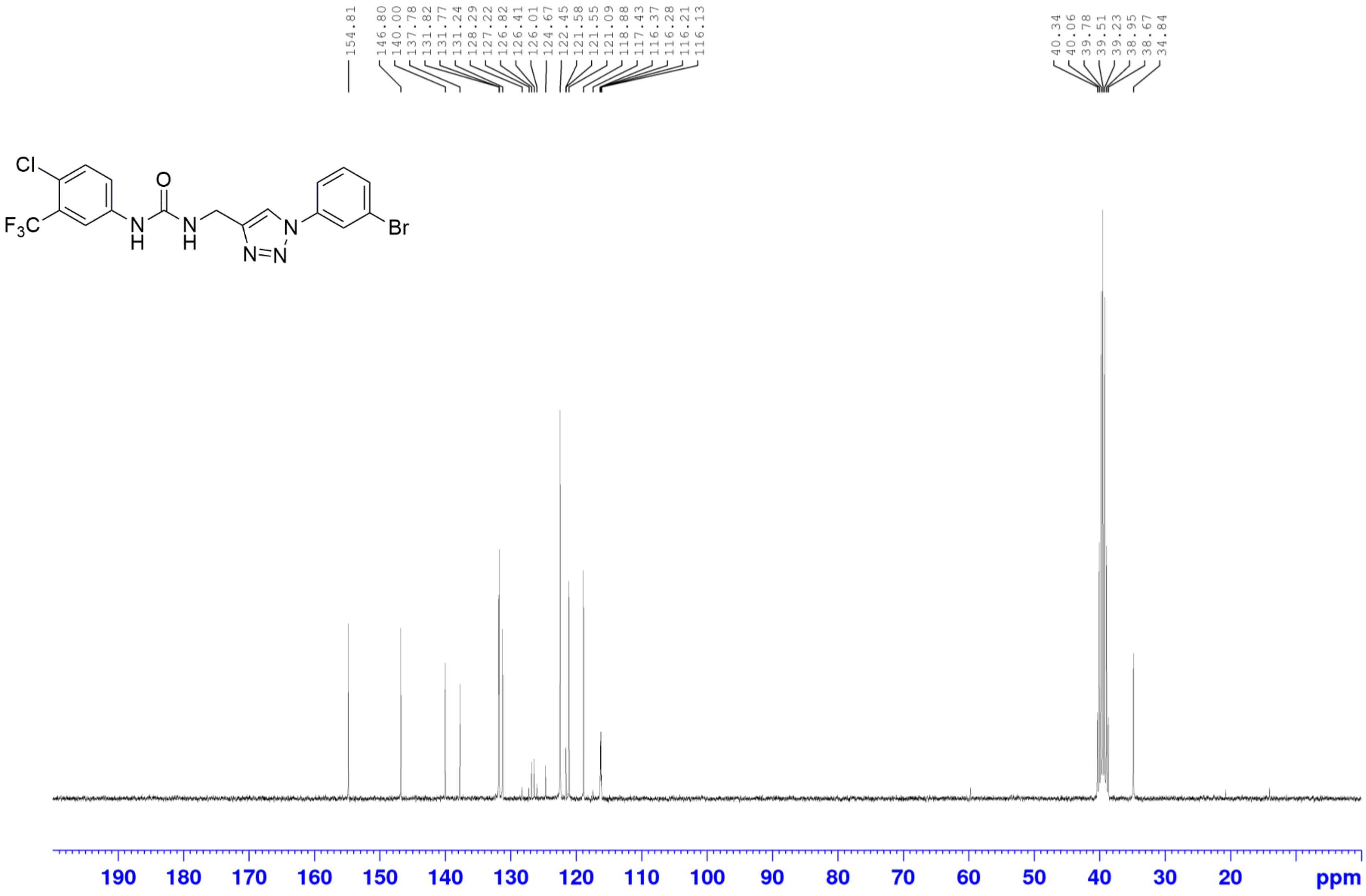

^{19}F NMR of compound **2i** (282 MHz, DMSO-d_6)

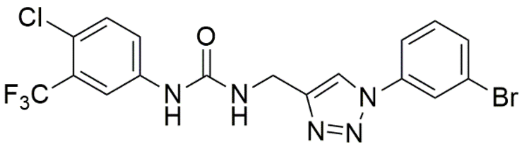

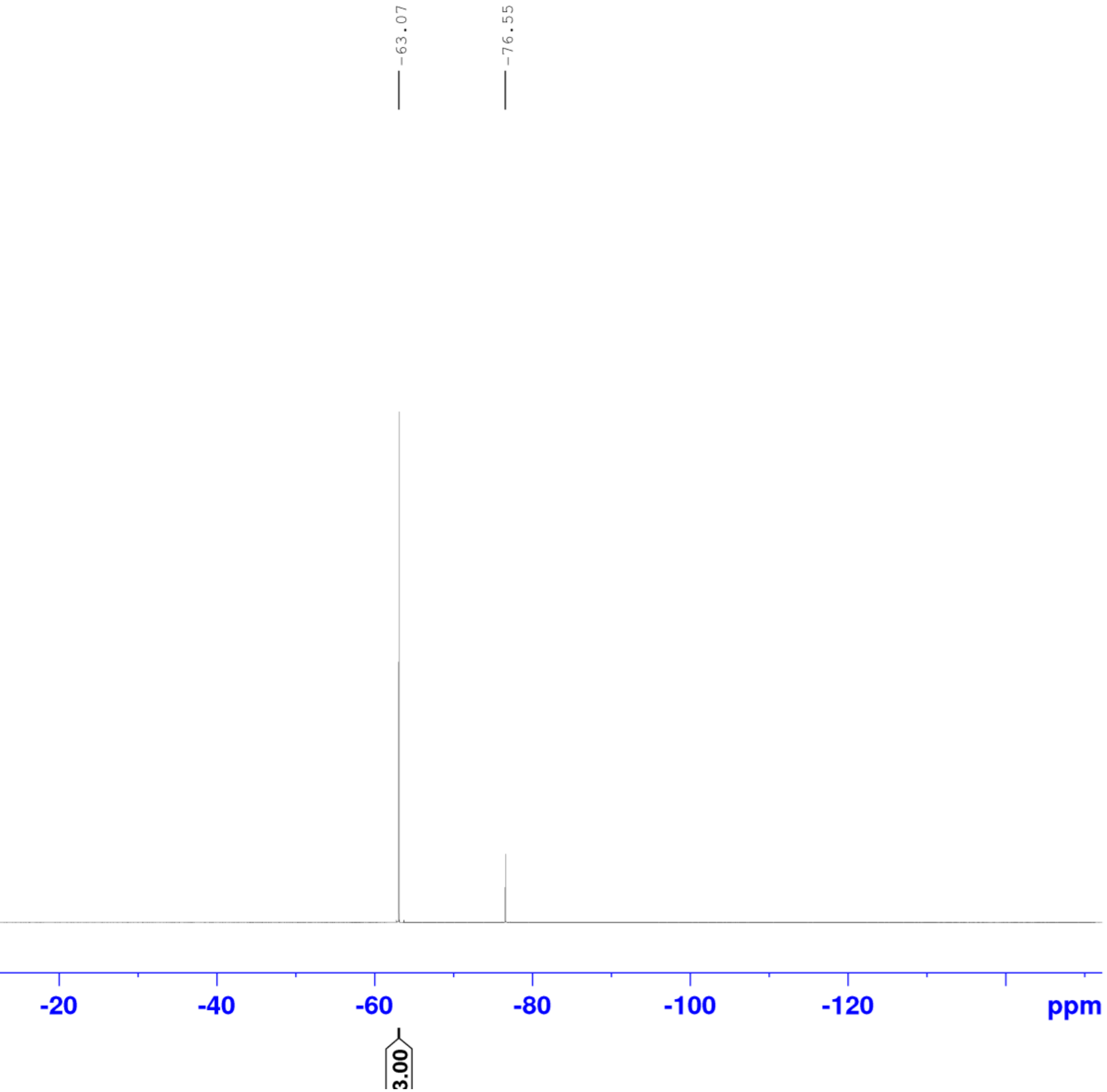

^{1}H NMR of compound **2j** (300 MHz, DMSO-d_6)

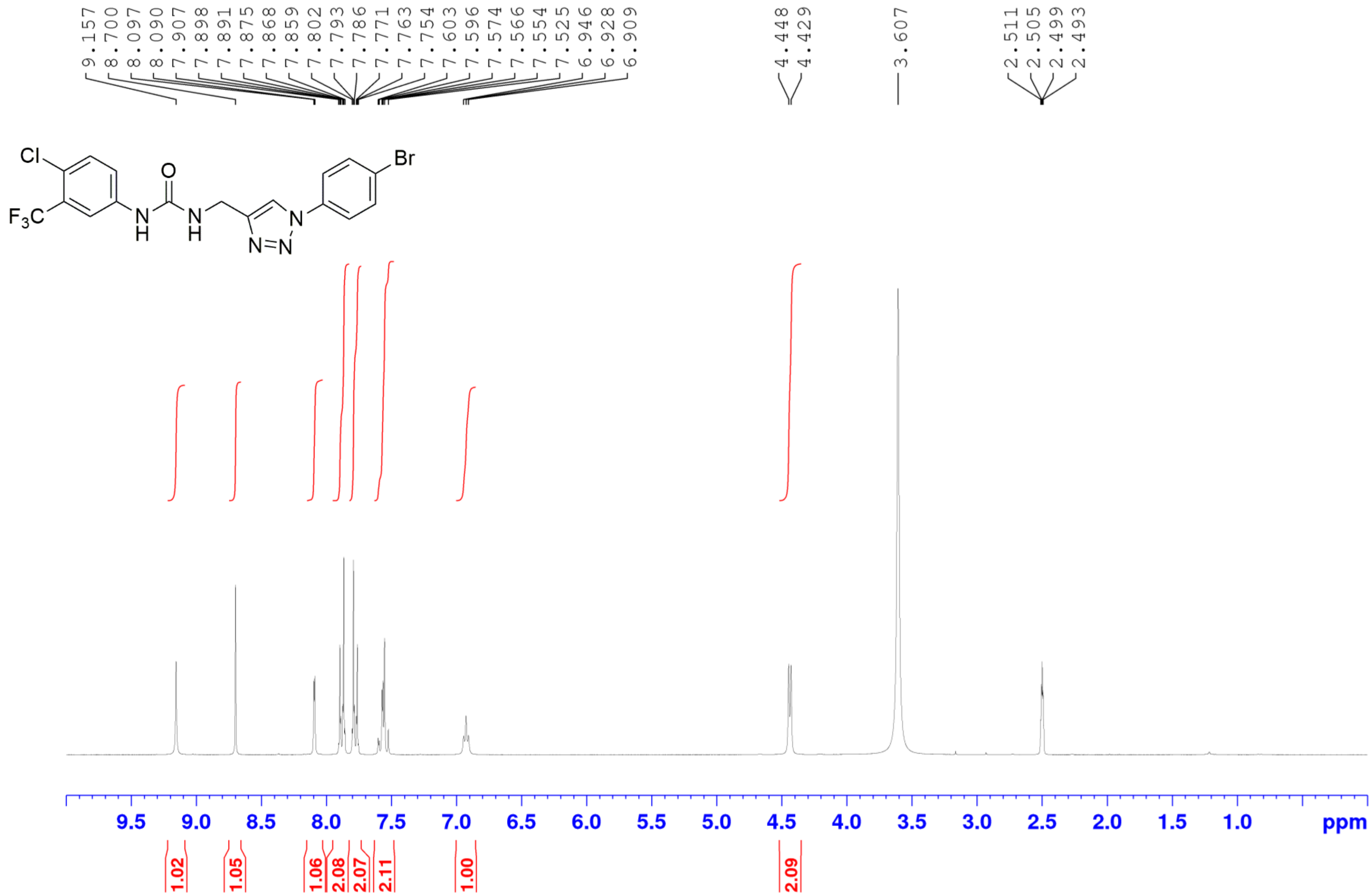

^{13}C NMR of compound **2j** (75 MHz, DMSO-d_6)

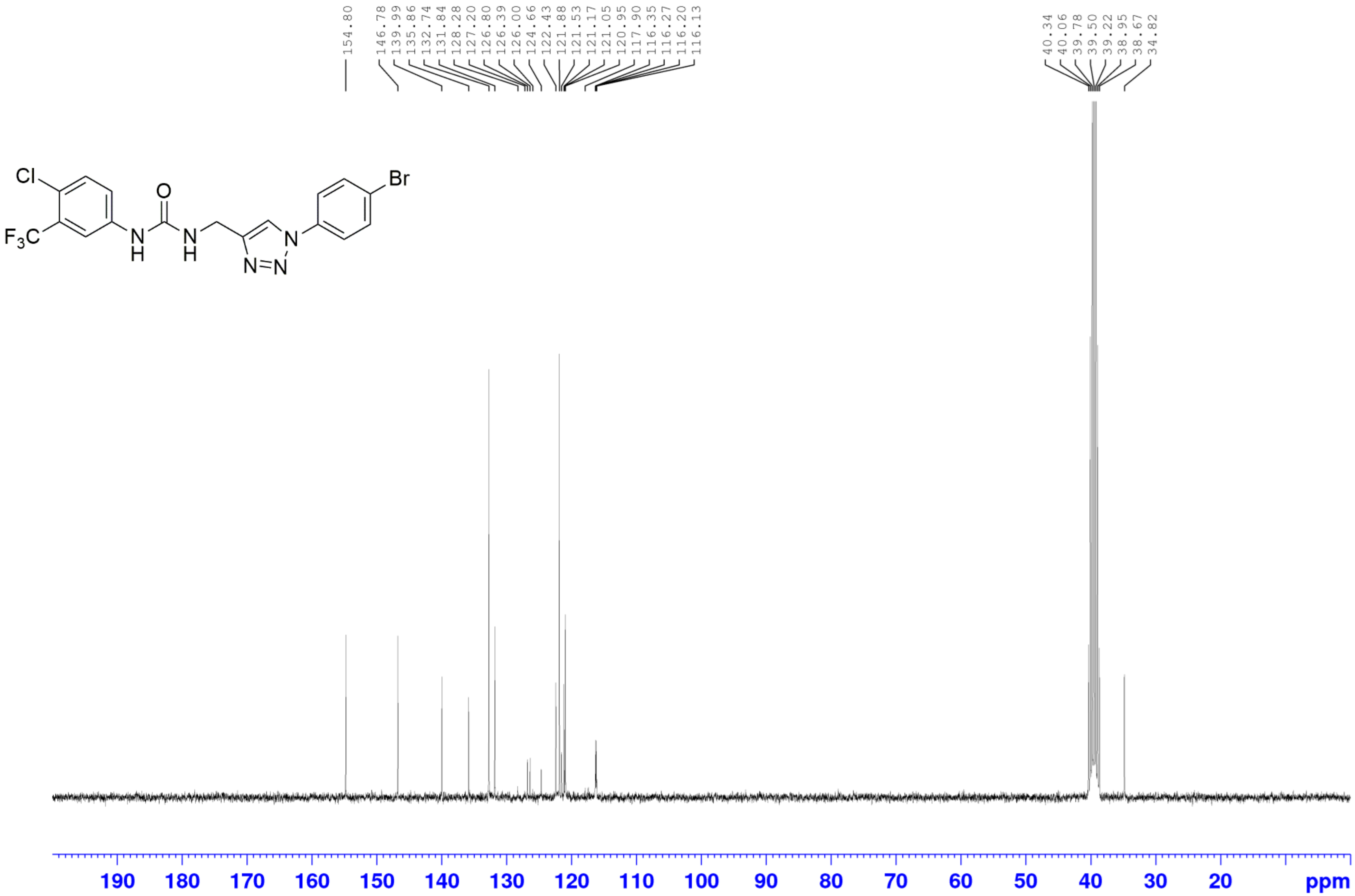

^{19}F NMR of compound **2j** (282 MHz, DMSO-*d6*)

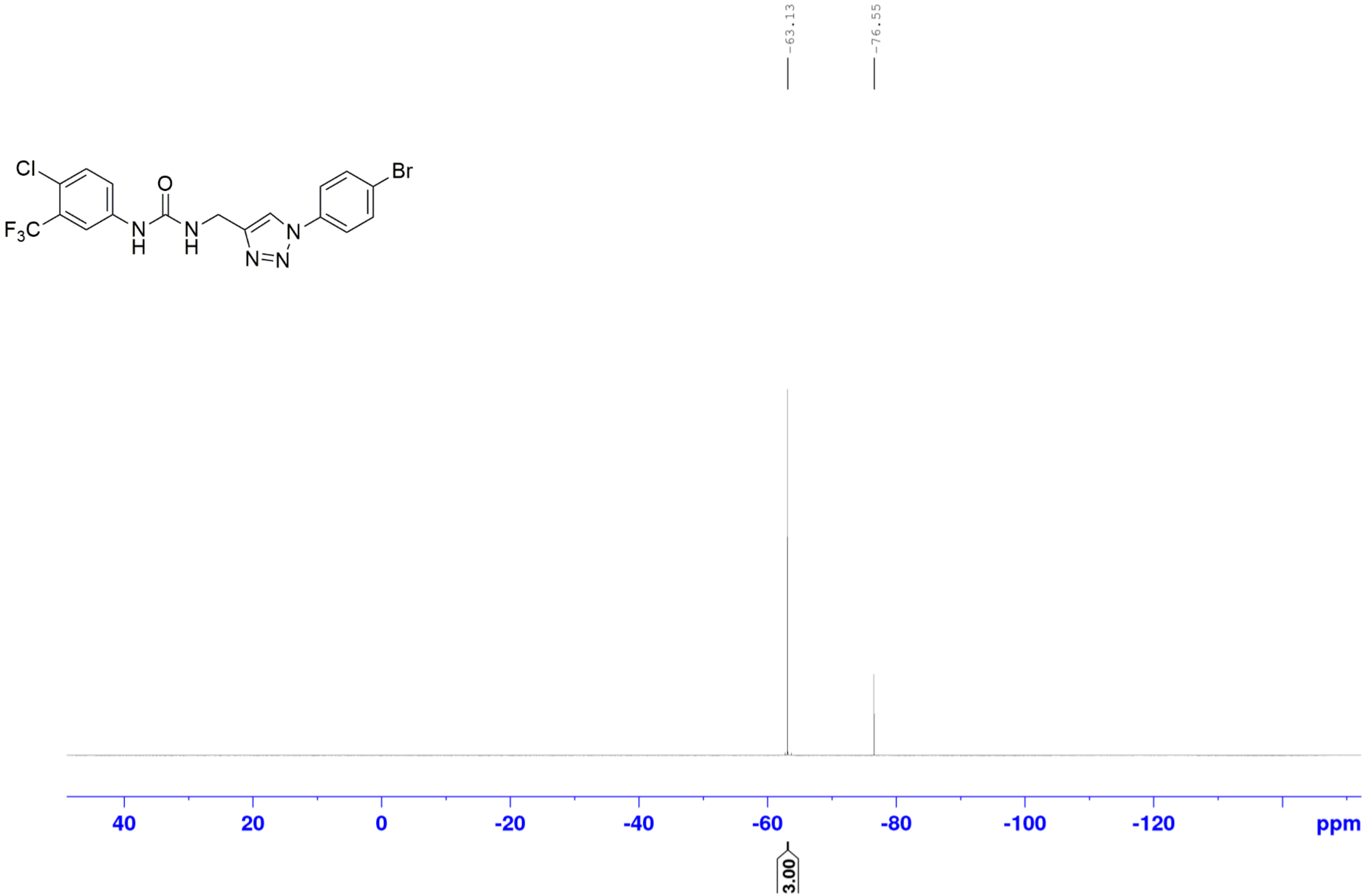

^{1}H NMR of compound **2k** (300 MHz, DMSO-d_6)

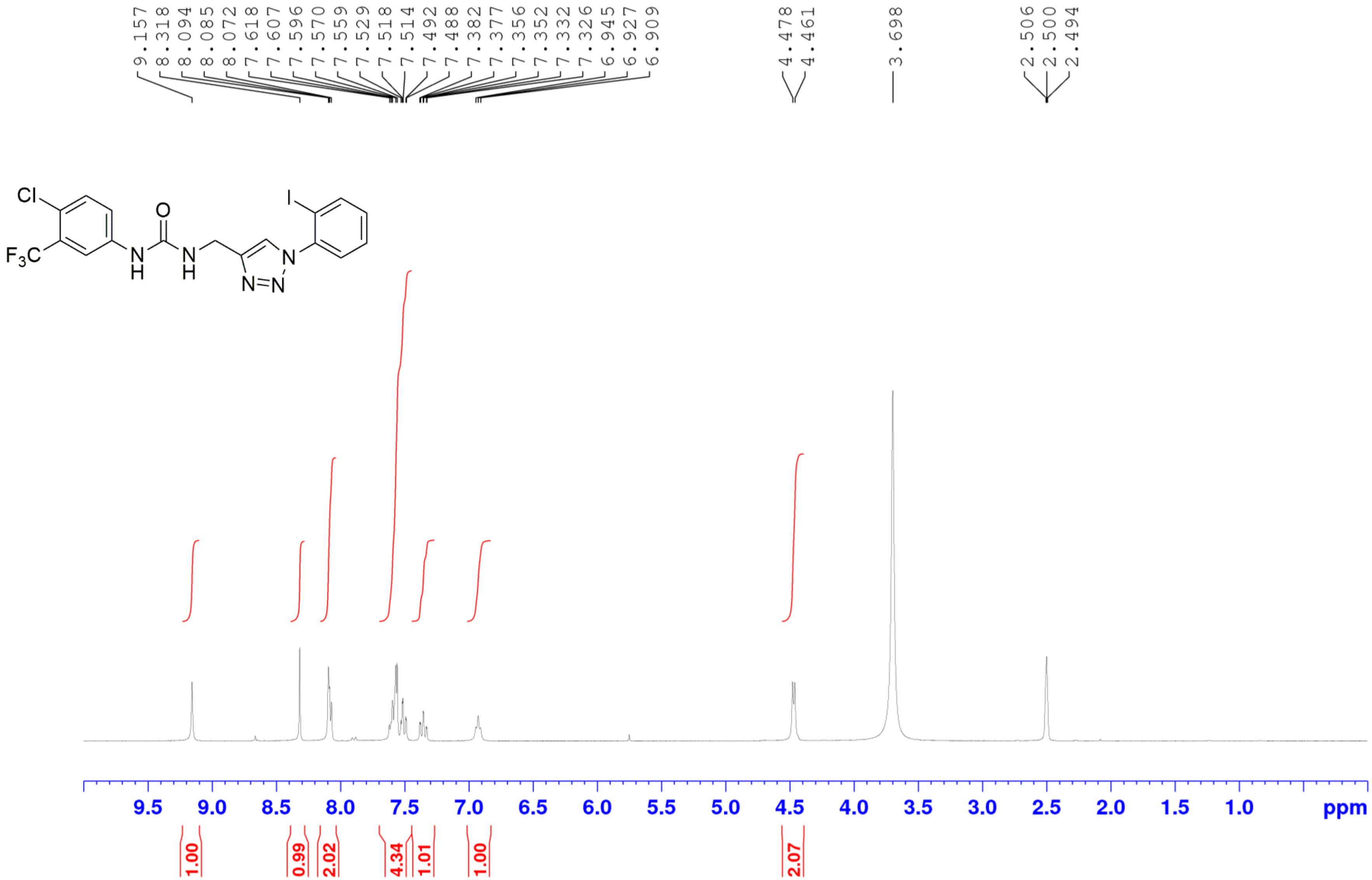

^{13}C NMR of compound **2k** (75 MHz, DMSO-d_6)

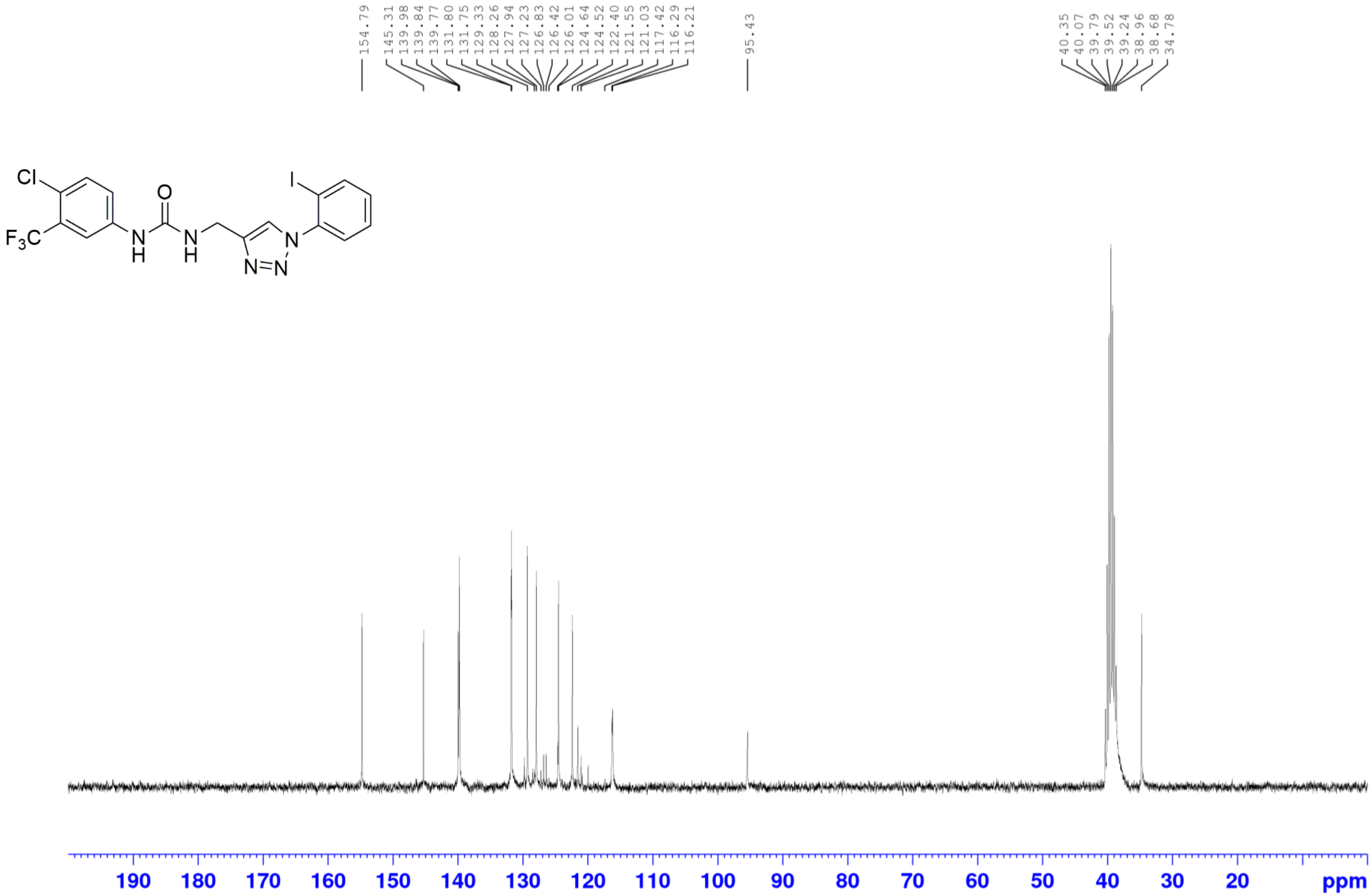

^{19}F NMR of compound **2k** (282 MHz, DMSO-d_6)

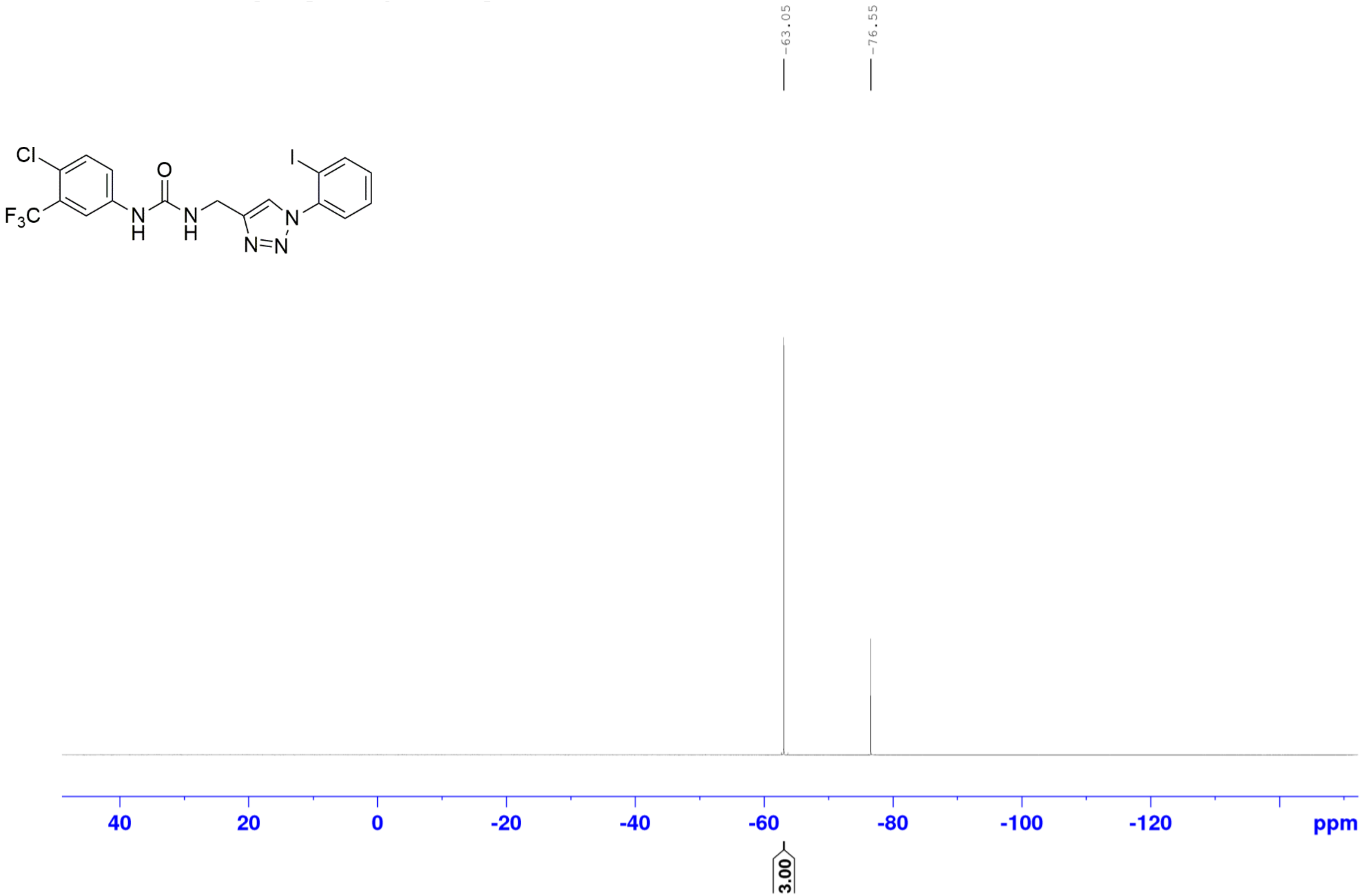

^{1}H NMR of compound **2l** (300 MHz, DMSO-d_6)

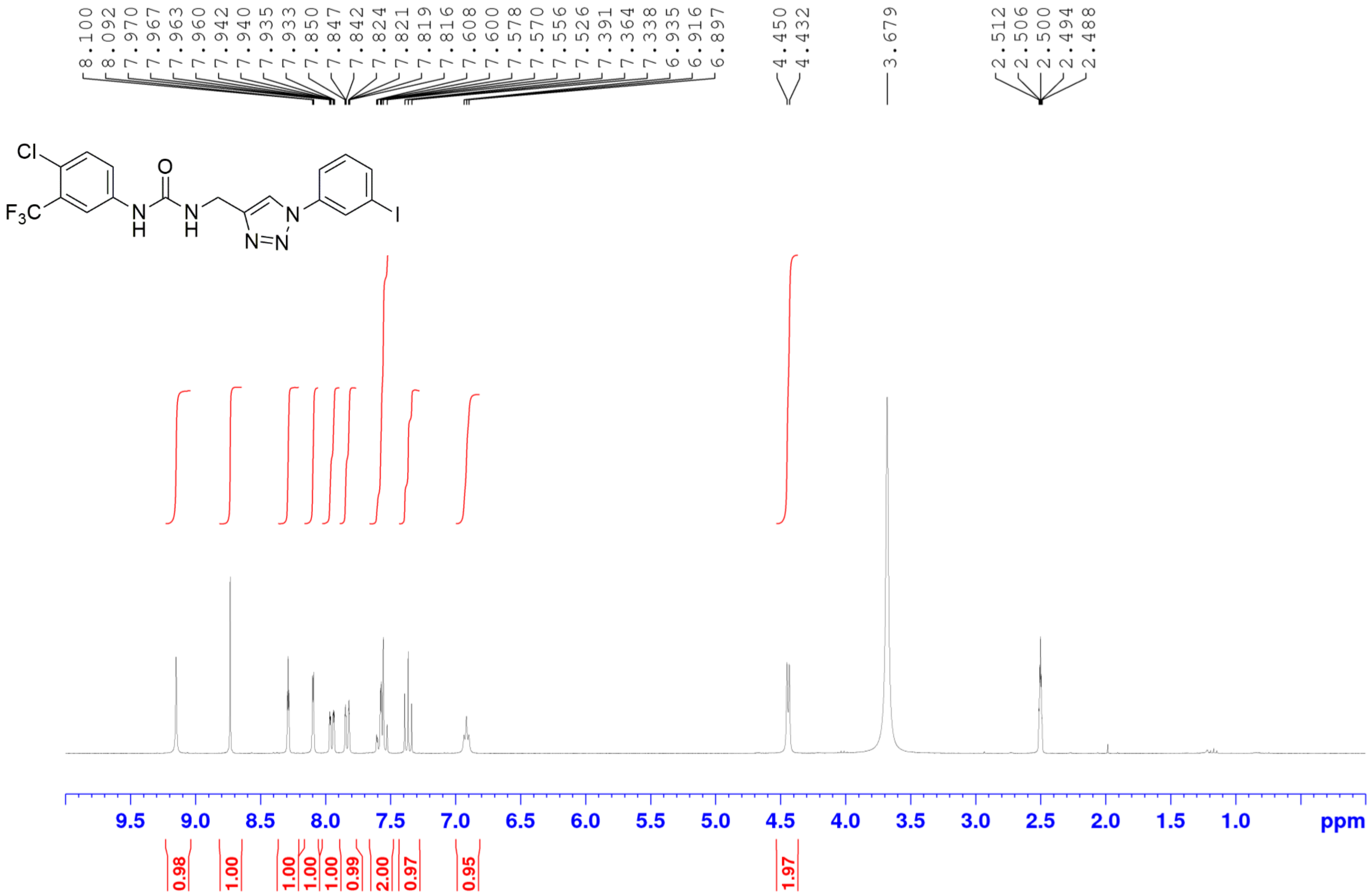

^{13}C NMR of compound **2l** (75 MHz, DMSO-d_6)

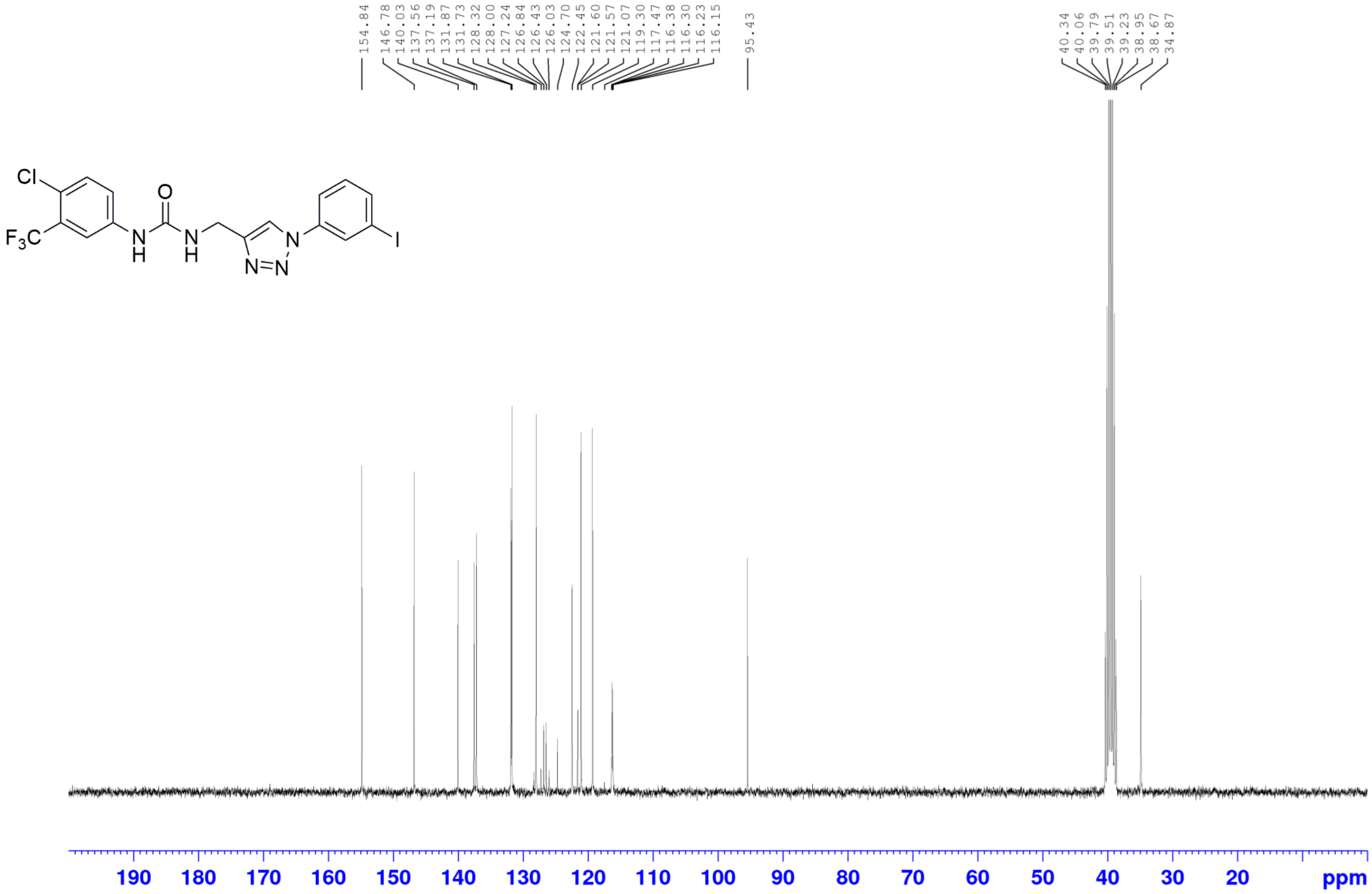

^{19}F NMR of compound **2c** (282 MHz, DMSO-d_6)

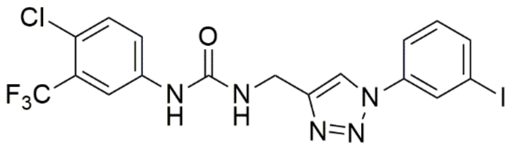

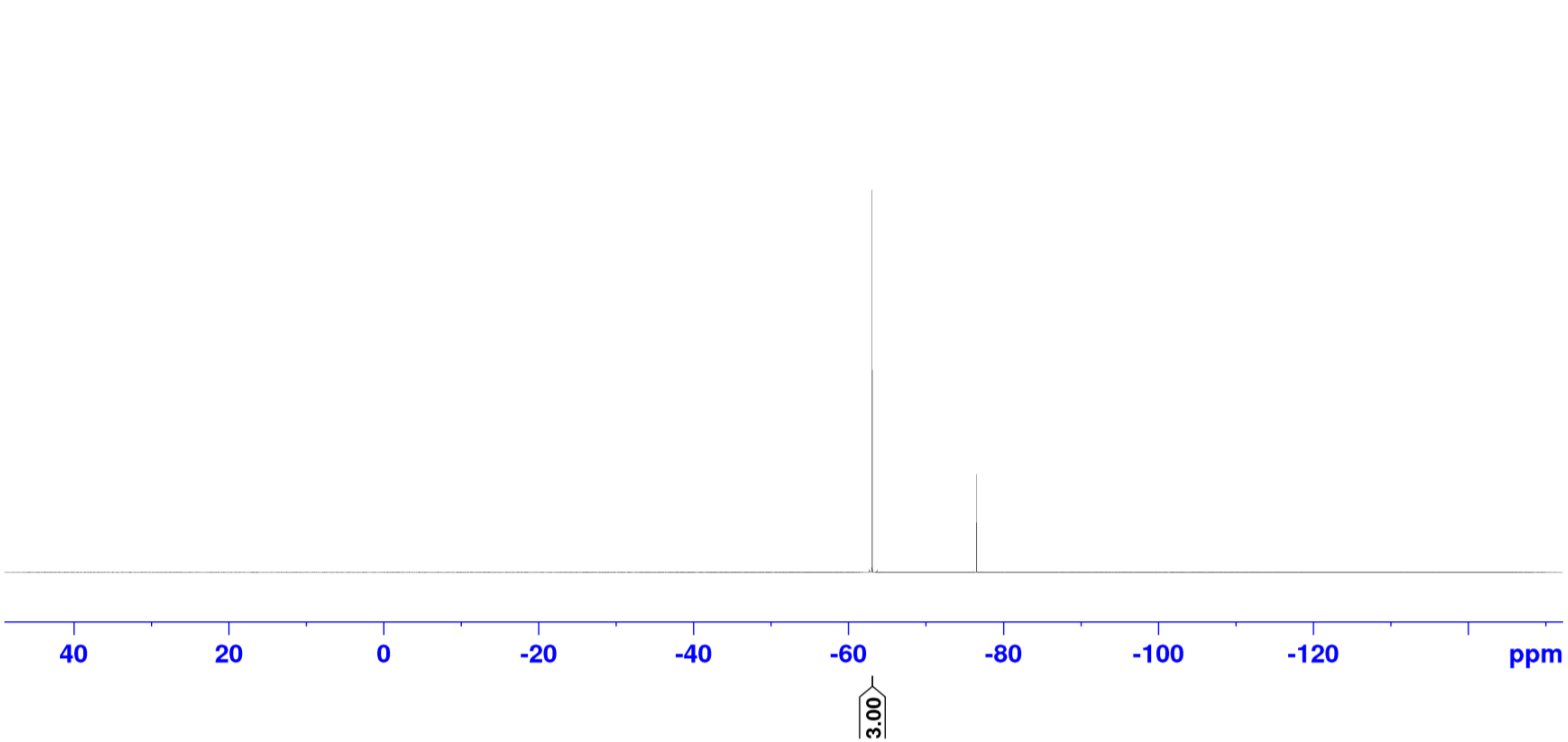

^{1}H NMR of compound **2m** (300 MHz, DMSO-d_6)

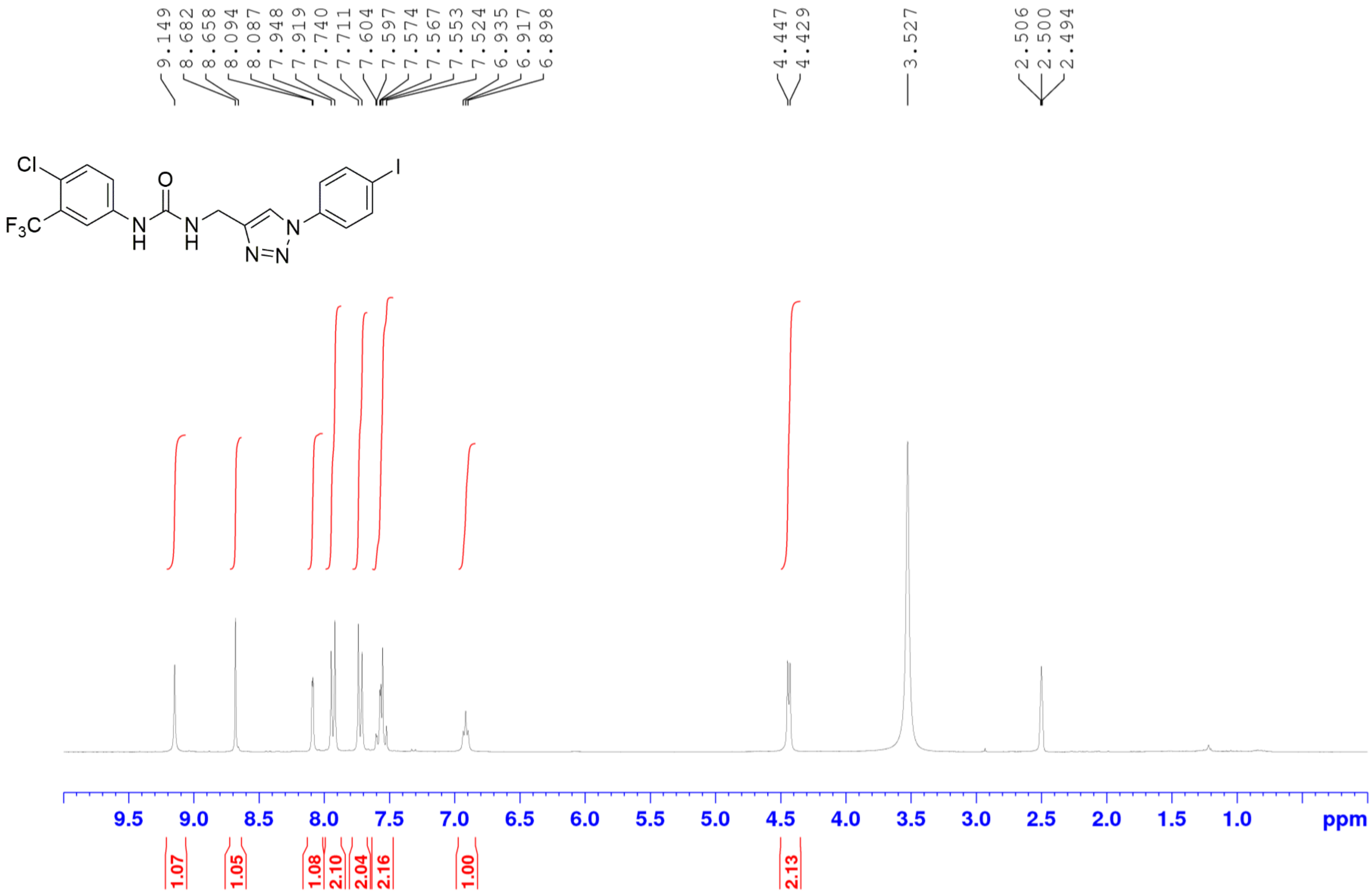

^{13}C NMR of compound **2m** (75 MHz, DMSO-*d6*)

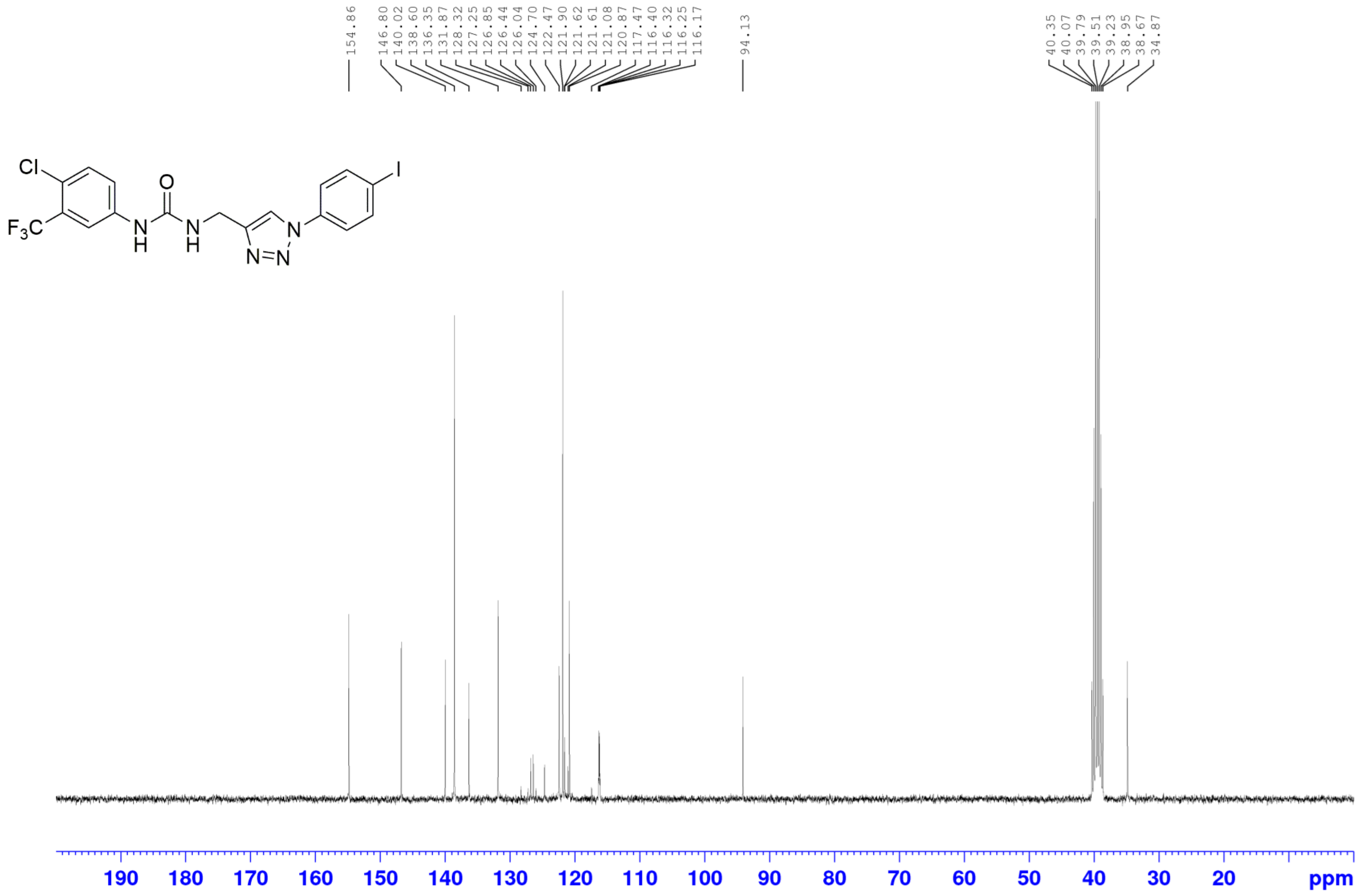

^{19}F NMR of compound **2m** (282 MHz, DMSO-d_6)

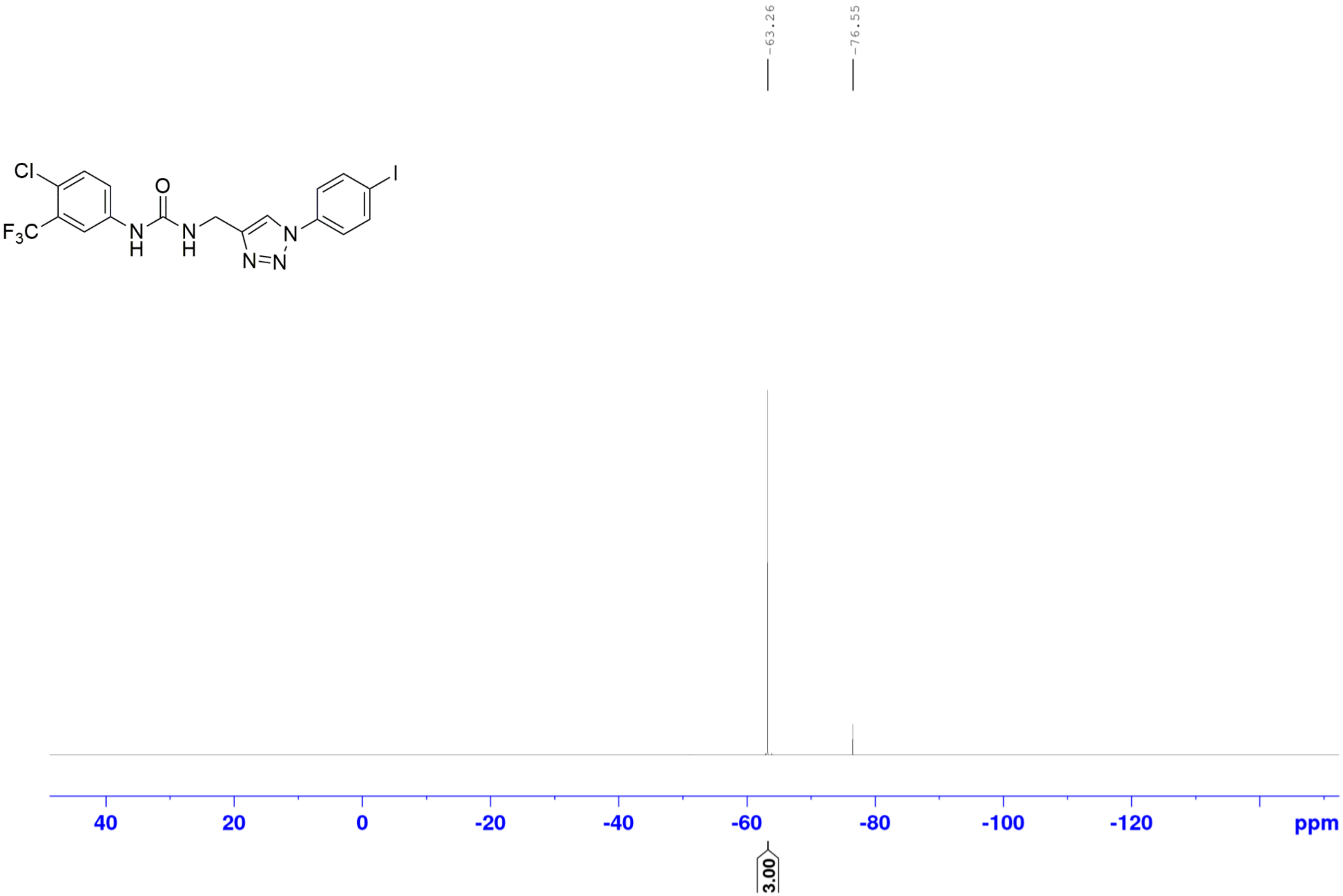

^{1}H NMR of compound **2n** (300 MHz, DMSO-d_6)

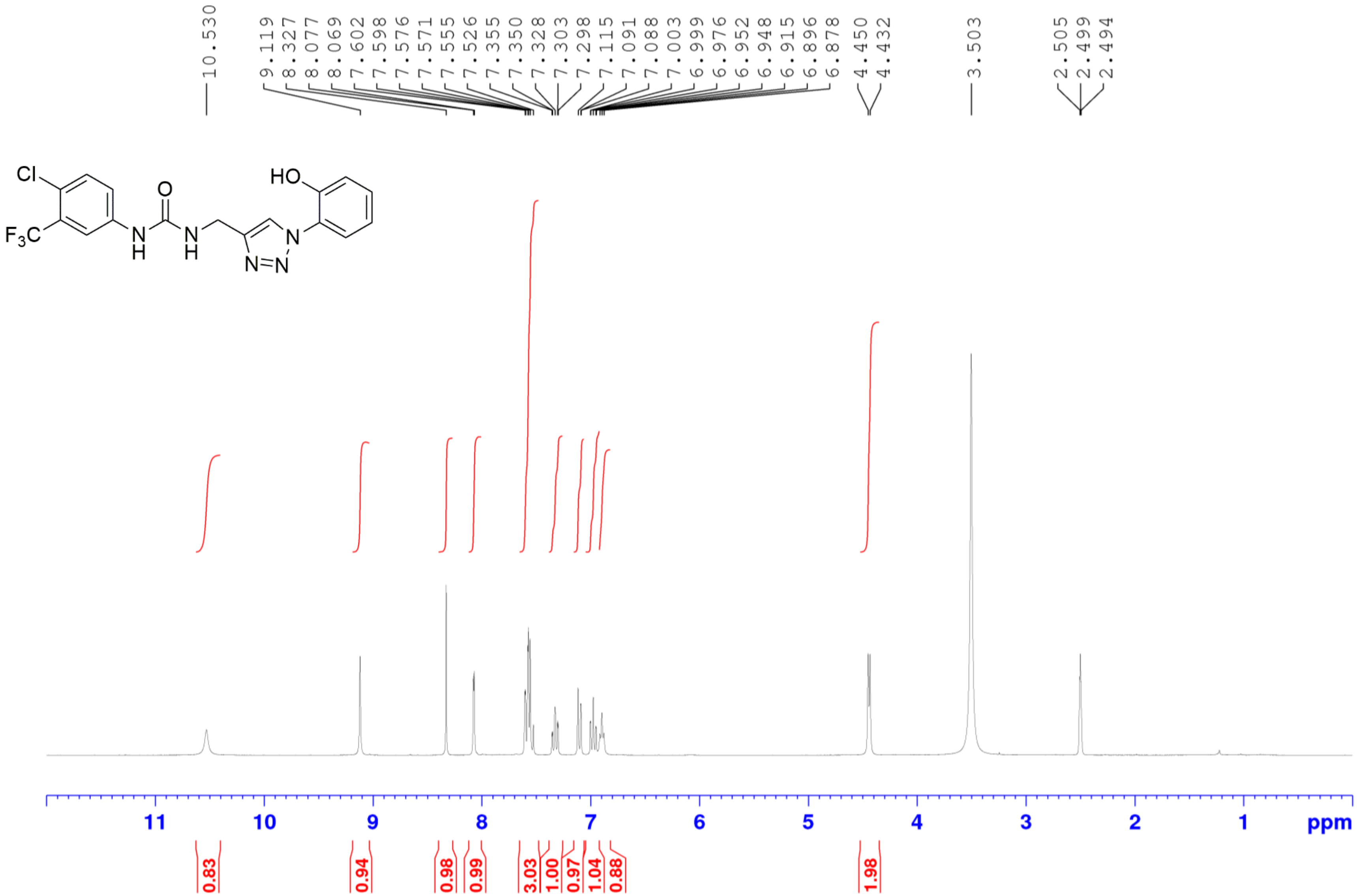

^{13}C NMR of compound **2n** (75 MHz, DMSO-d_6)

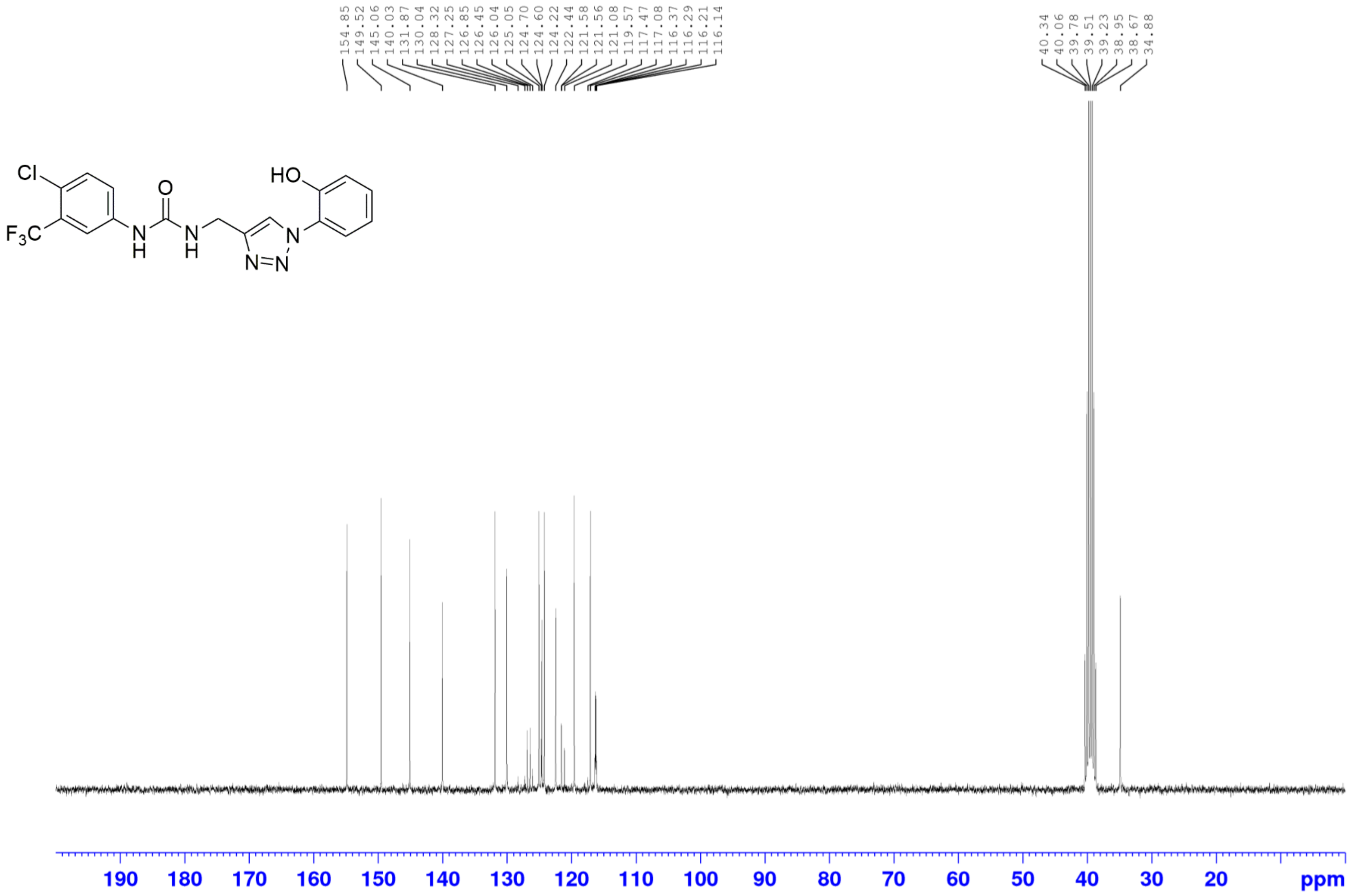

^{19}F NMR of compound **2n** (282 MHz, DMSO-d_6)

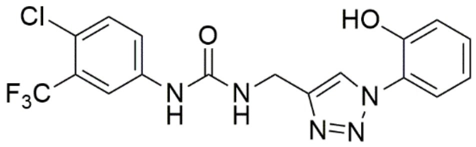

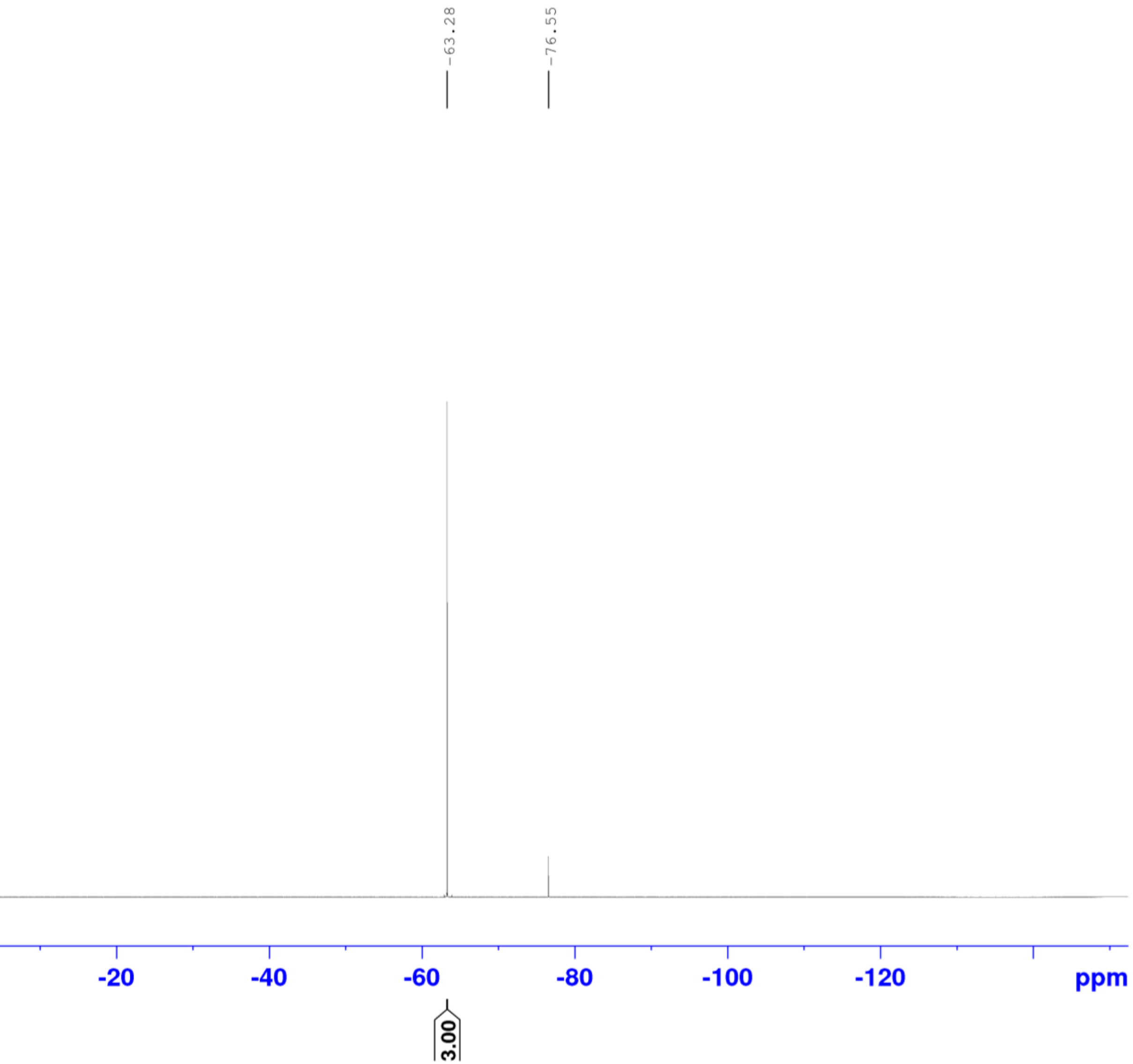

^{1}H NMR of compound **2o** (300 MHz, DMSO-d_6)

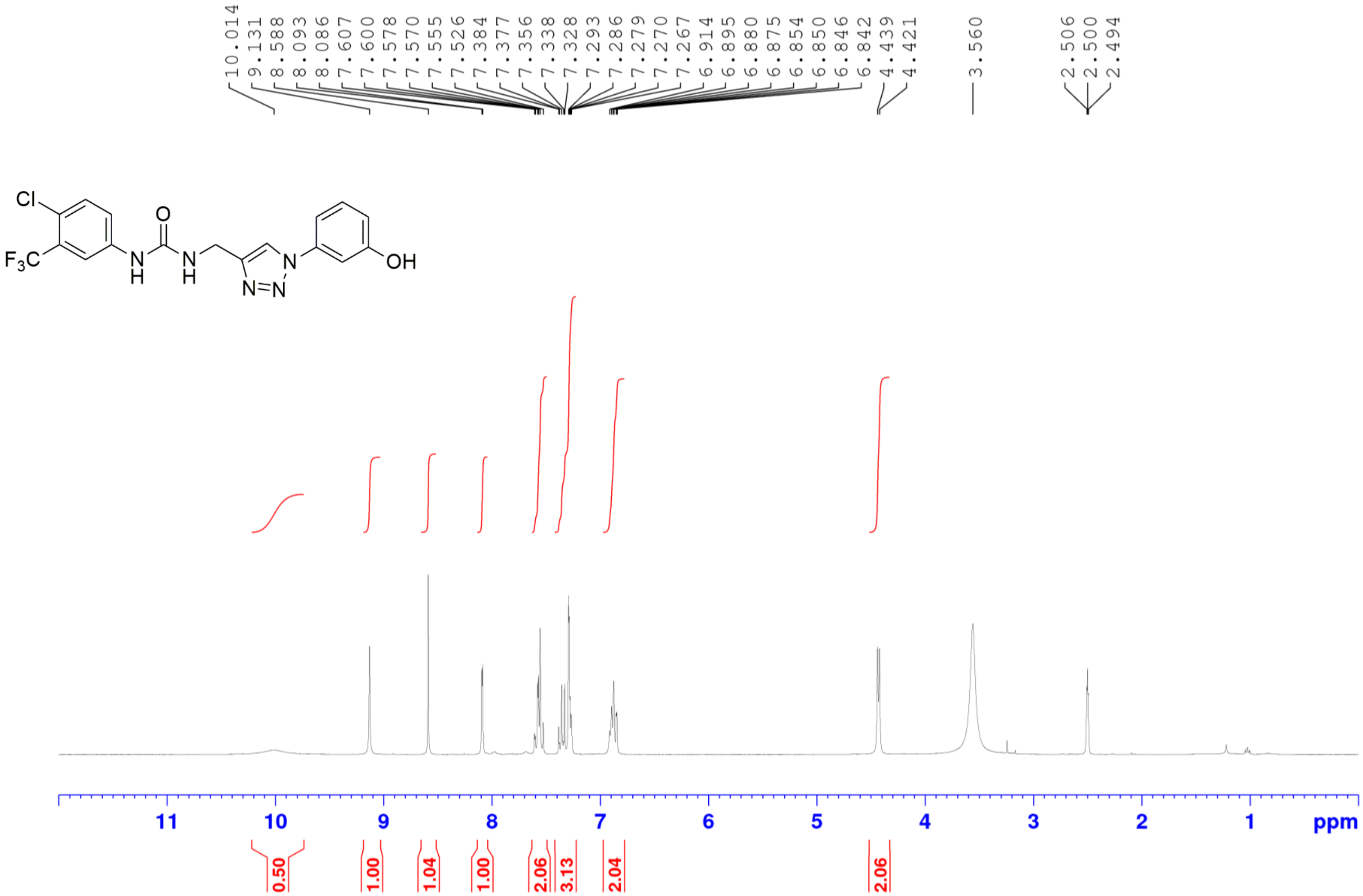

^{13}C NMR of compound **2o** (75 MHz, DMSO-d_6)

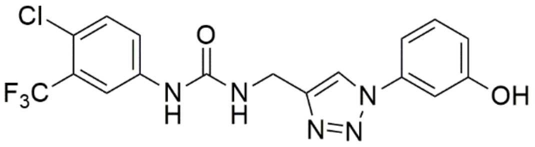

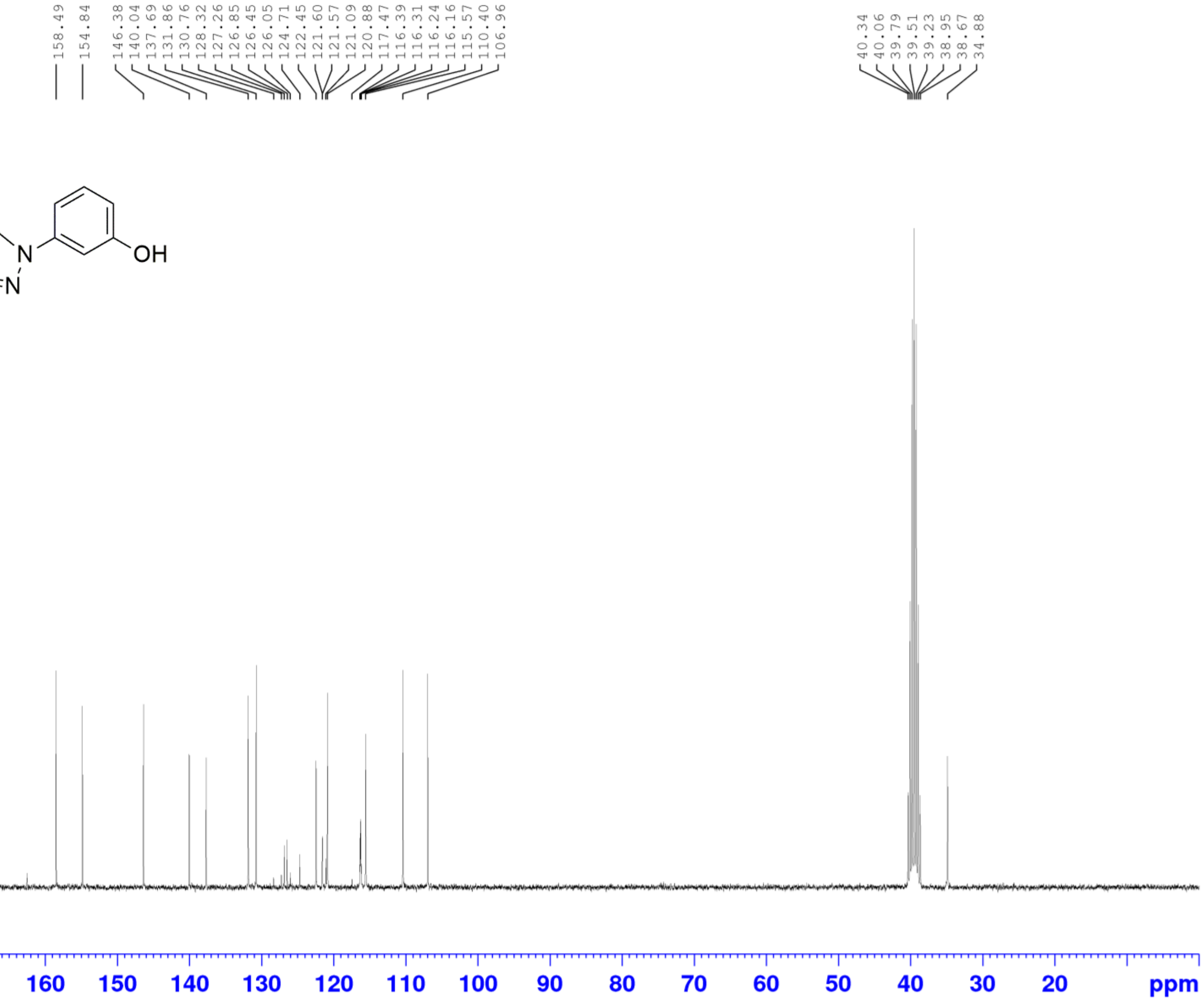

^{19}F NMR of compound **2o** (282 MHz, DMSO-d_6)

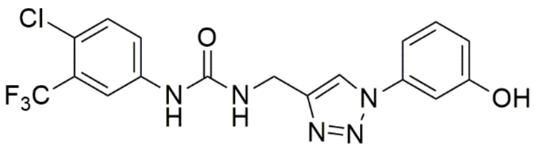

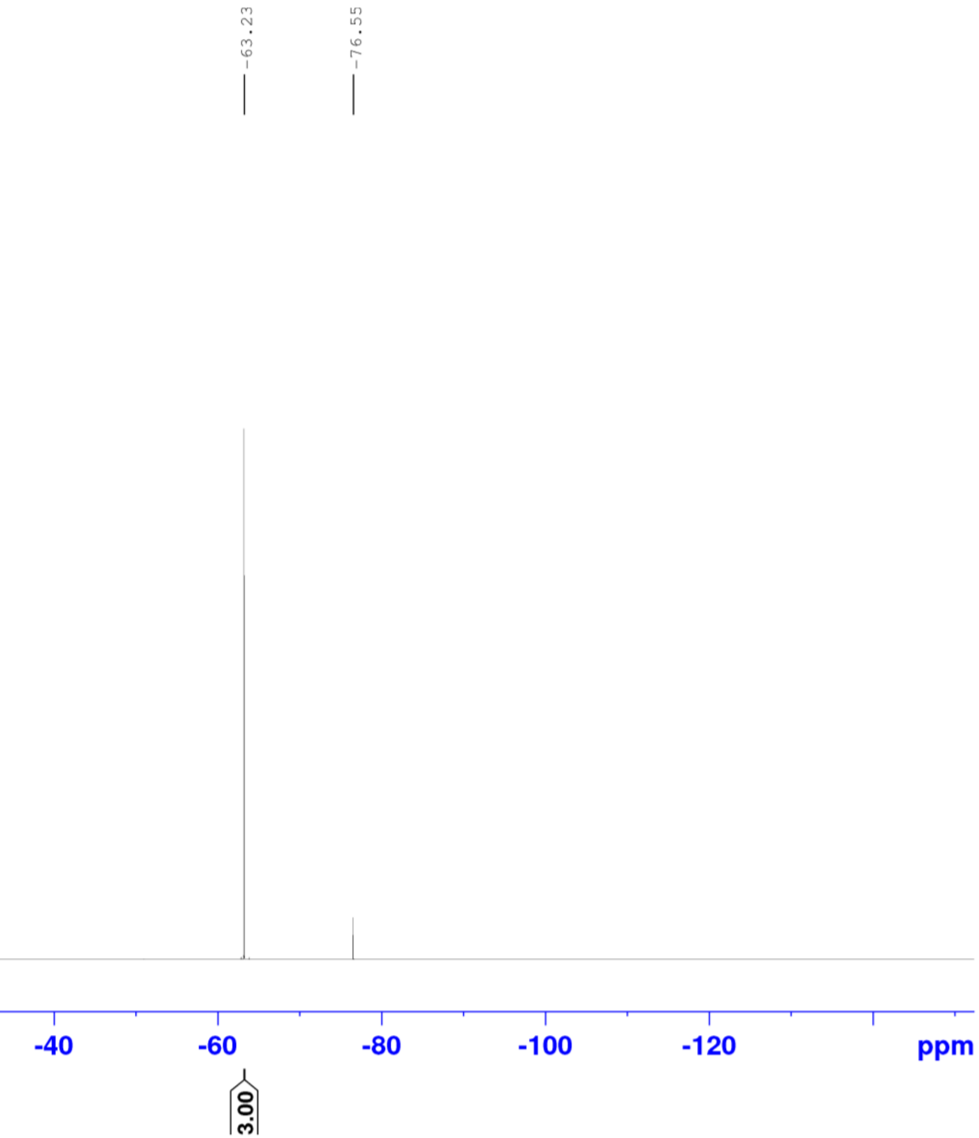

^{1}H NMR of compound **2p** (300 MHz, DMSO-d_6)

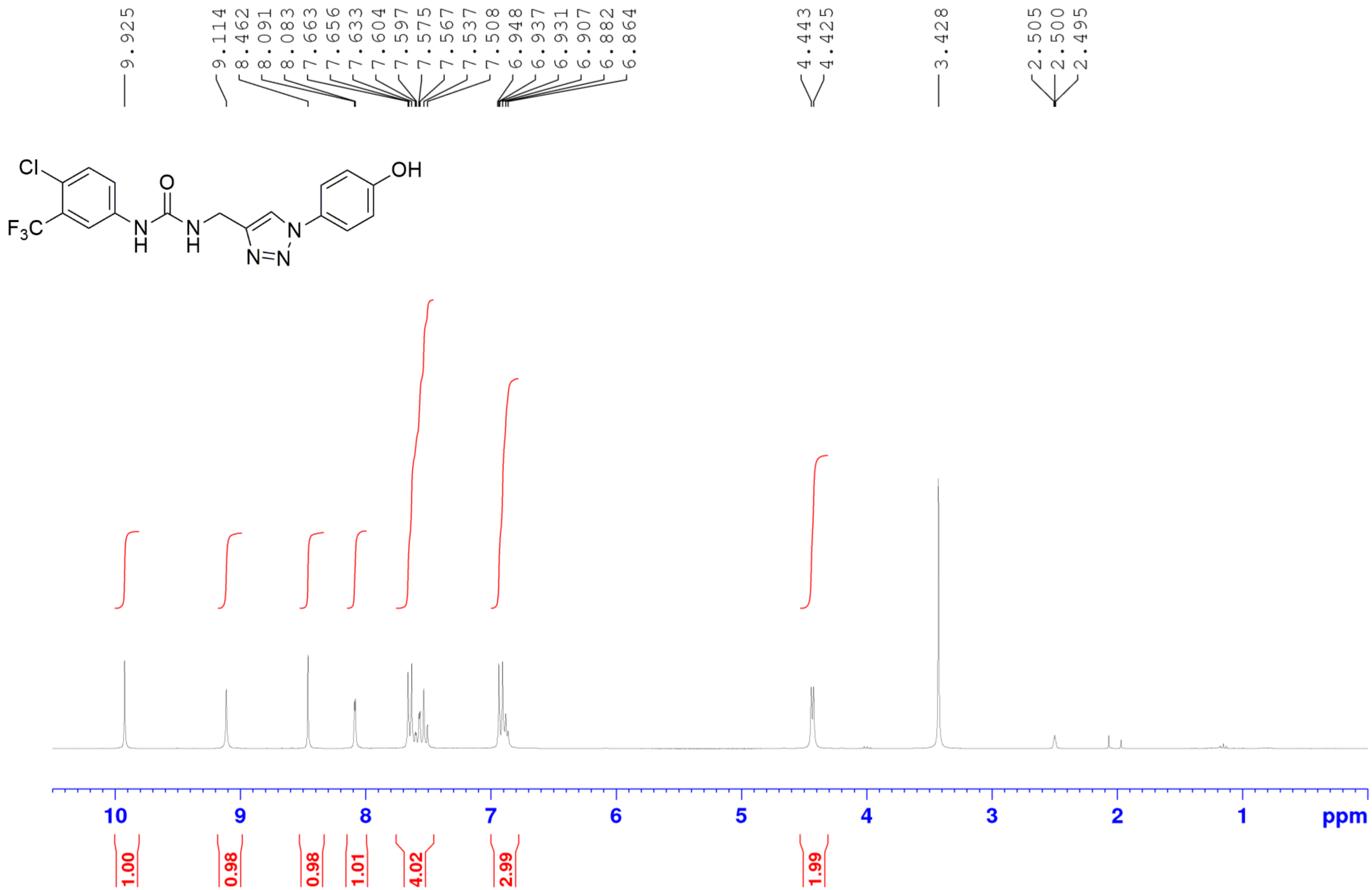

^{13}C NMR of compound **2p** (75 MHz, DMSO-d_6)

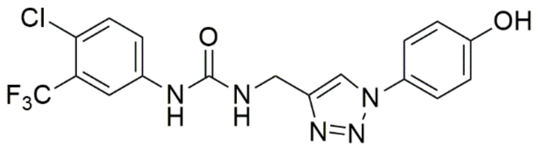

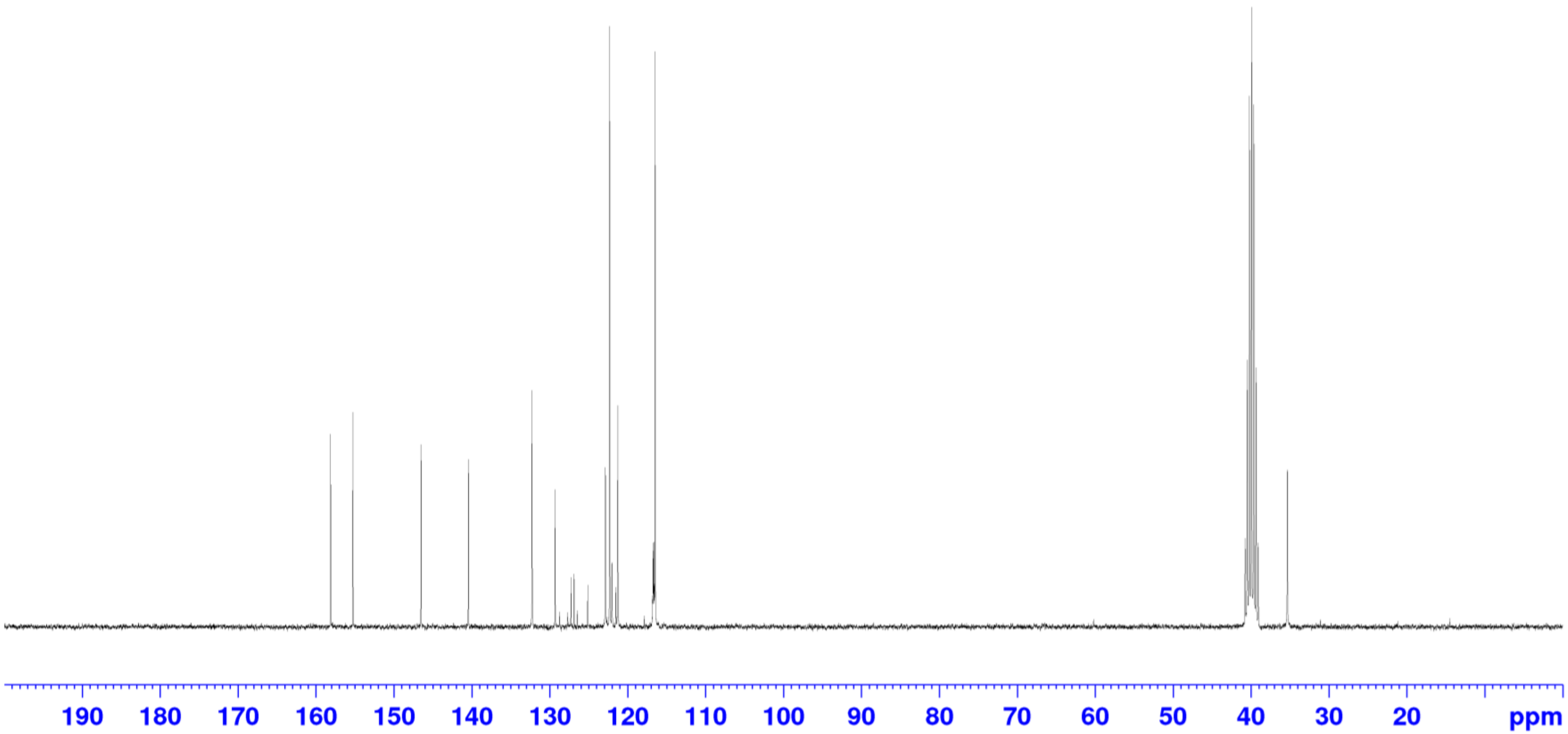

^{19}F NMR of compound **2p** (282 MHz, DMSO-d_6)

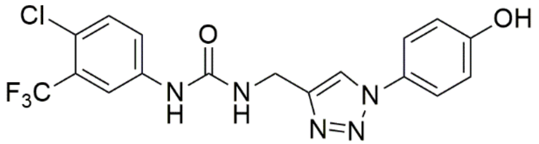

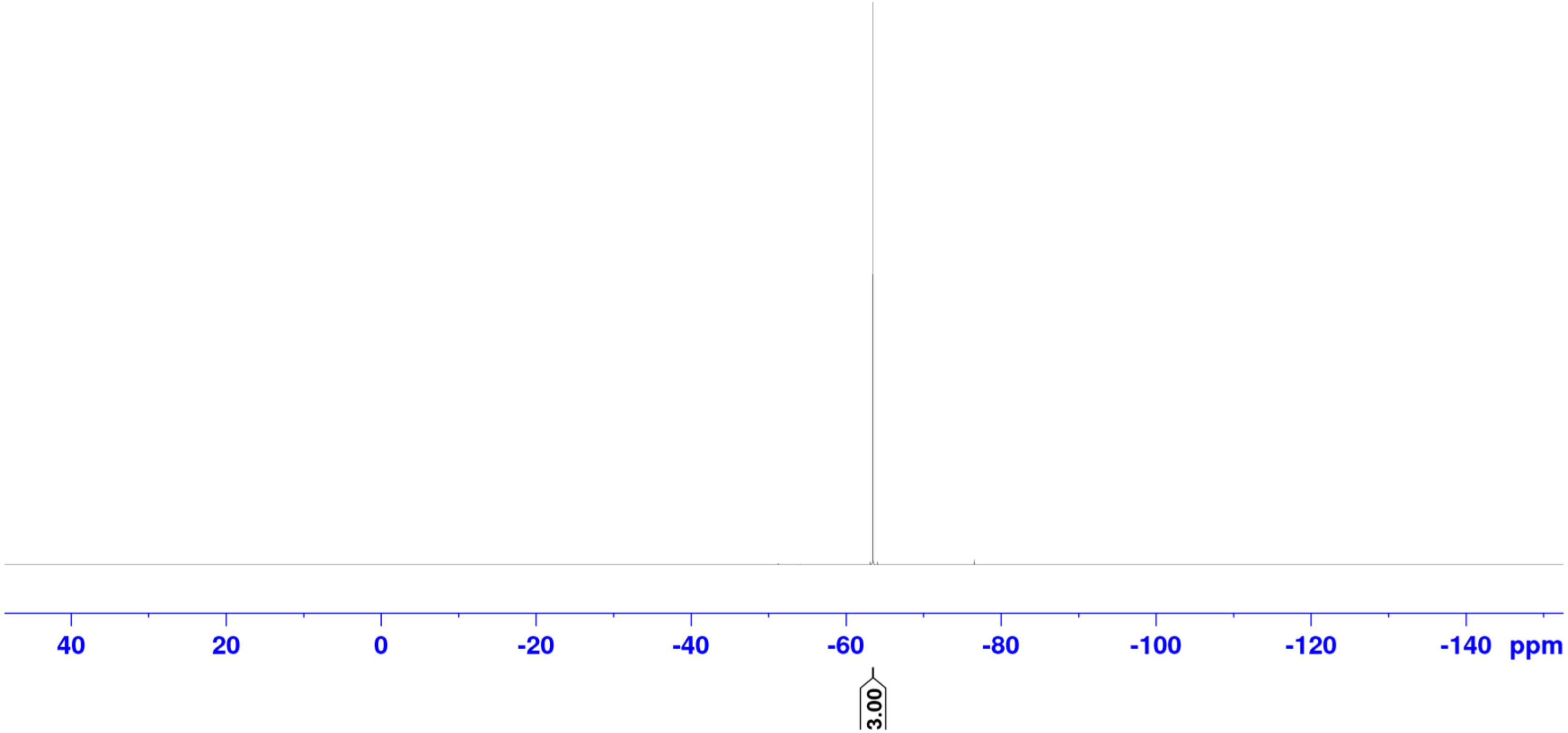

^{1}H NMR of compound **2q** (300 MHz, DMSO-d_6)

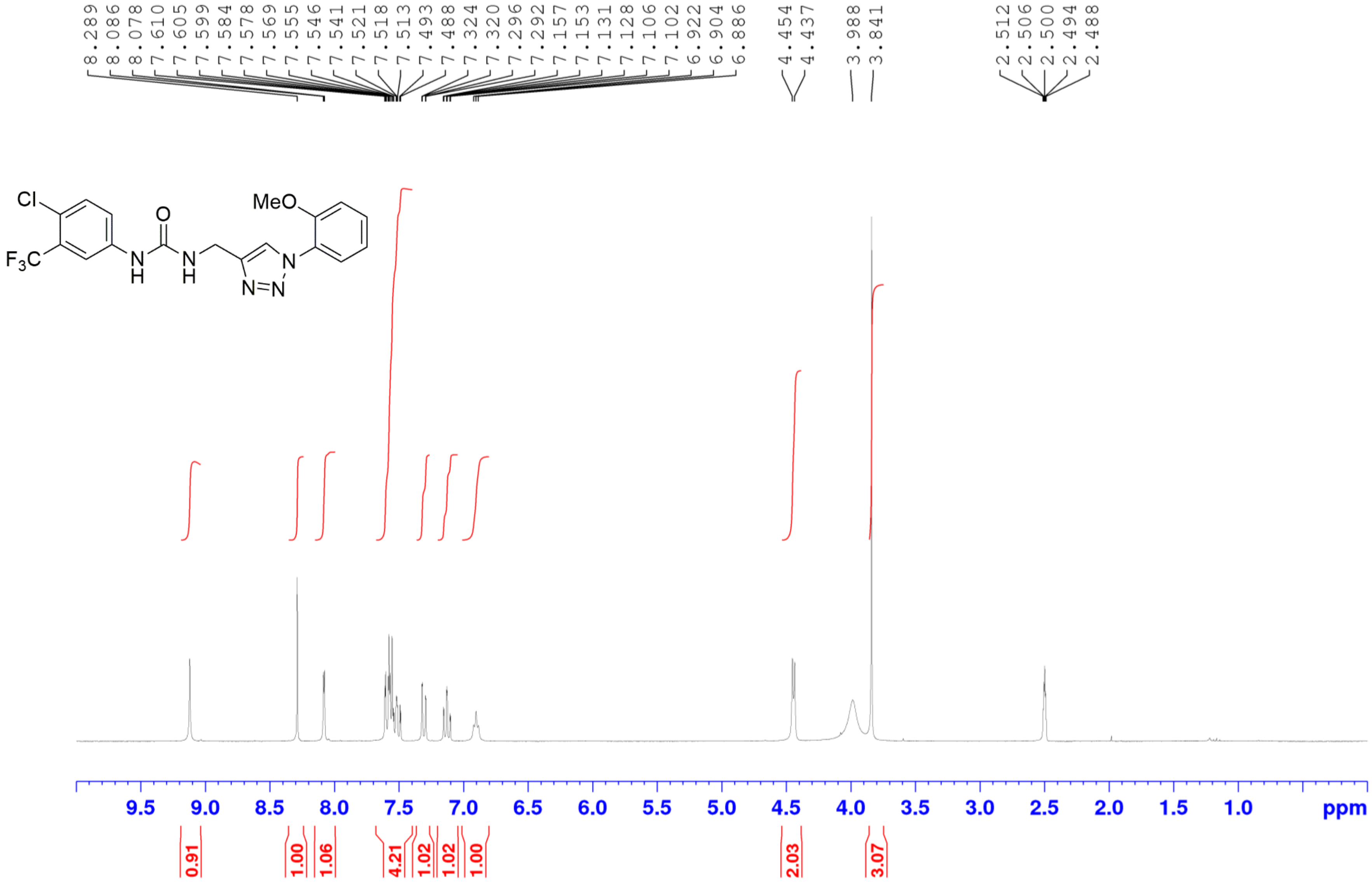

^{13}C NMR of compound **2q** (75 MHz, DMSO-d_6)

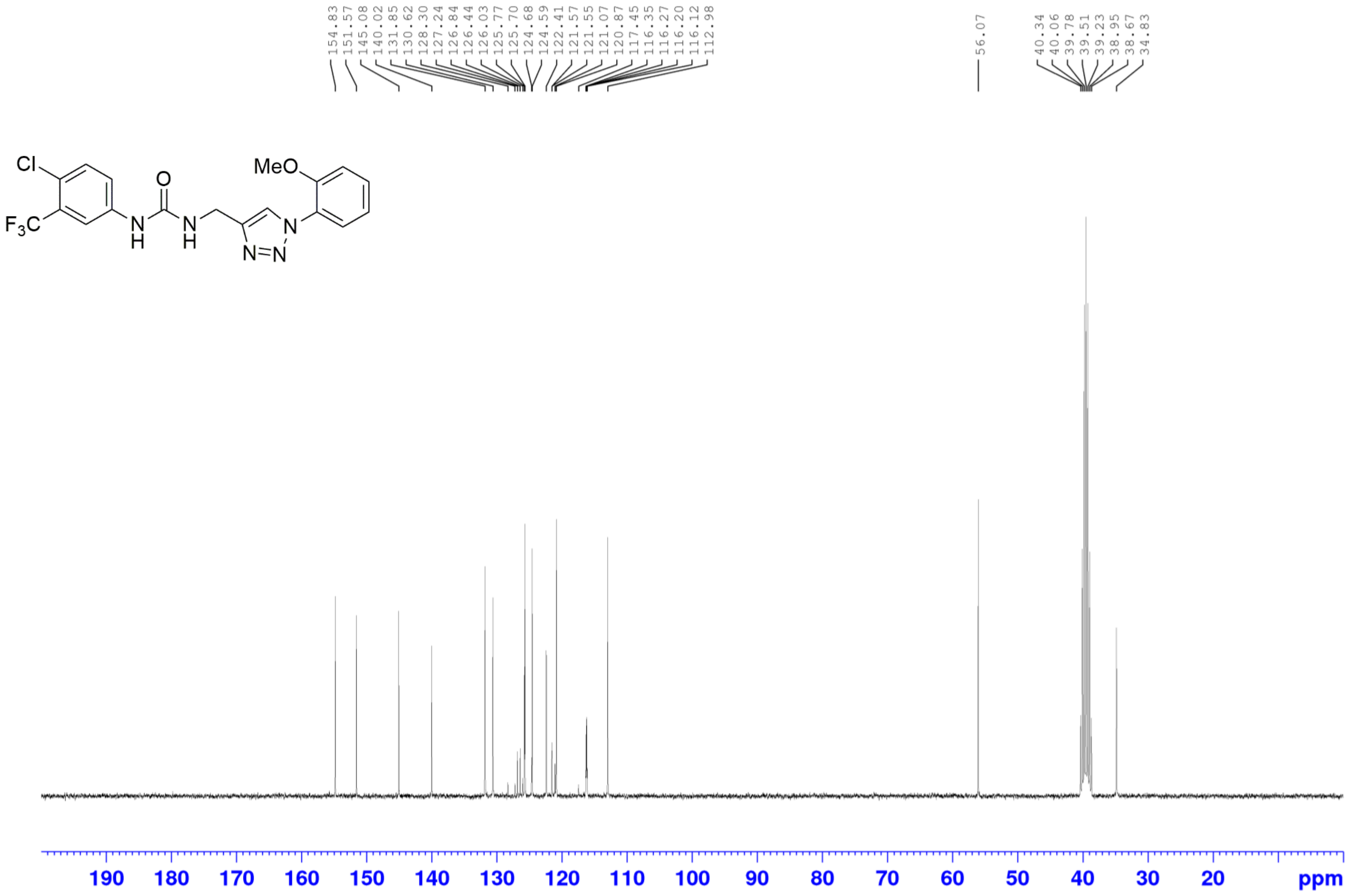

^{19}F NMR of compound **2q** (282 MHz, DMSO-d_6)

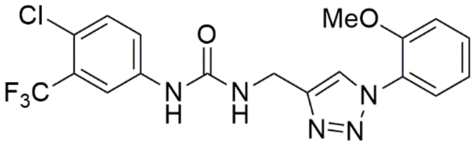

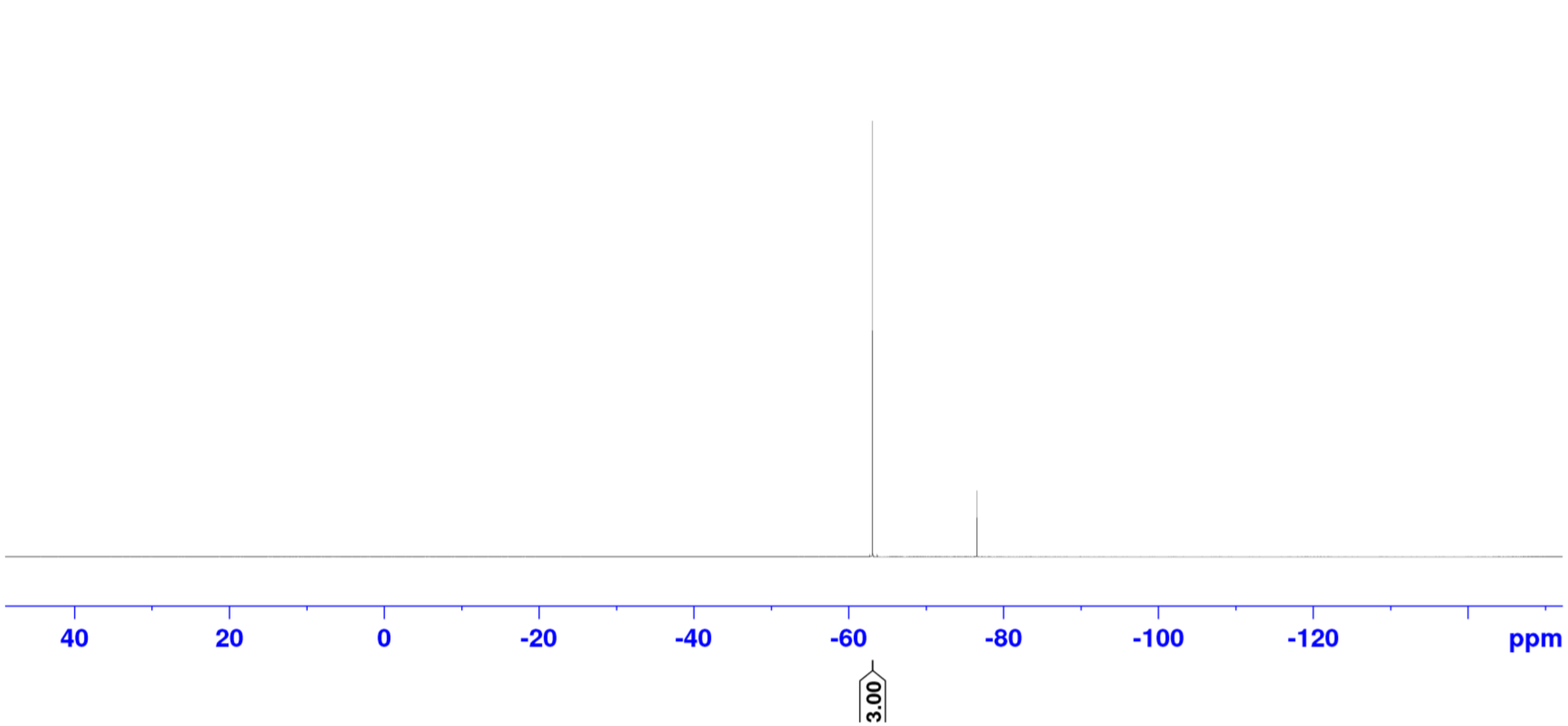

^{1}H NMR of compound **2r** (300 MHz, DMSO-d_6)

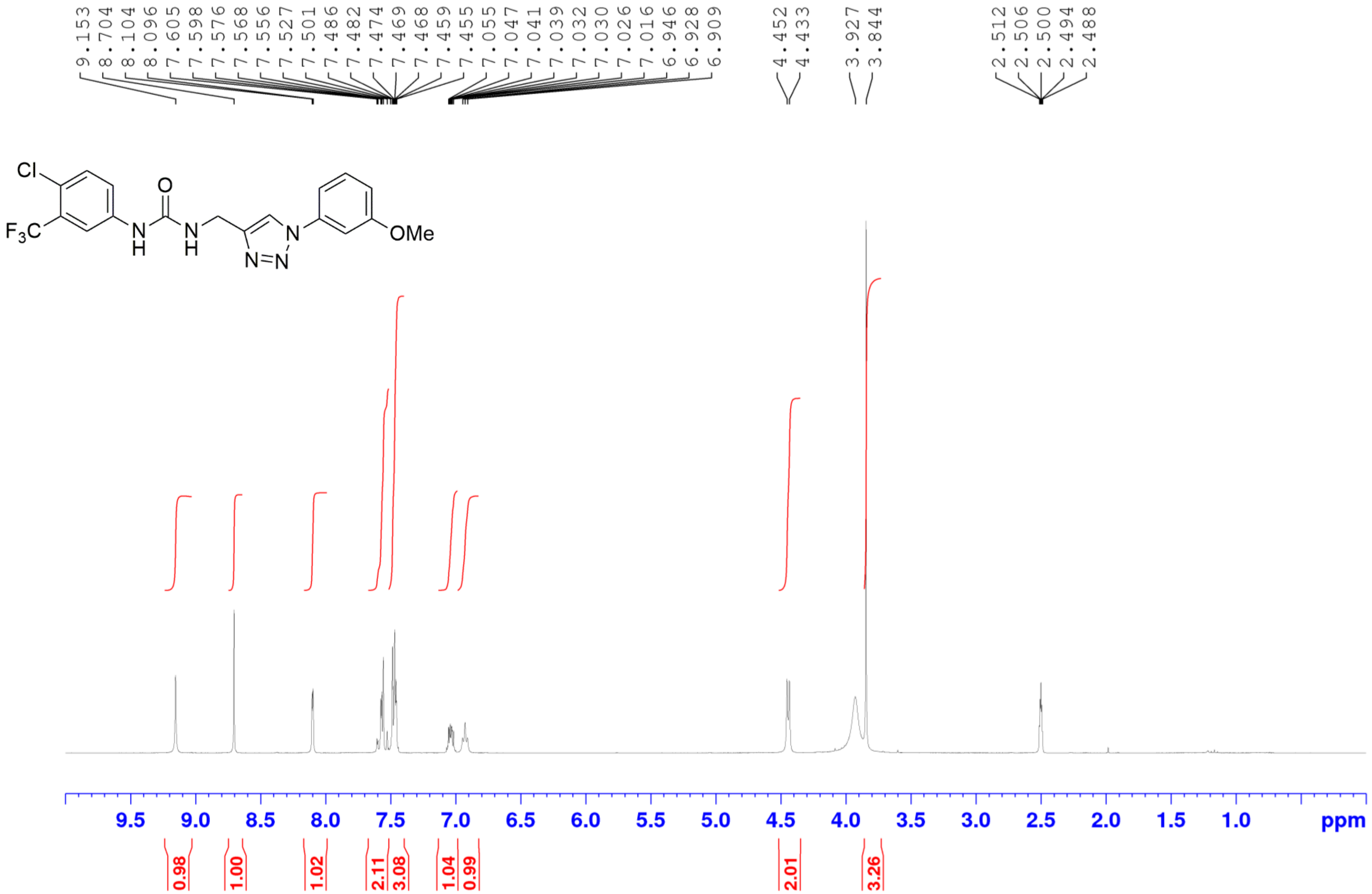

^{13}C NMR of compound **2r** (75 MHz, DMSO-d_6)

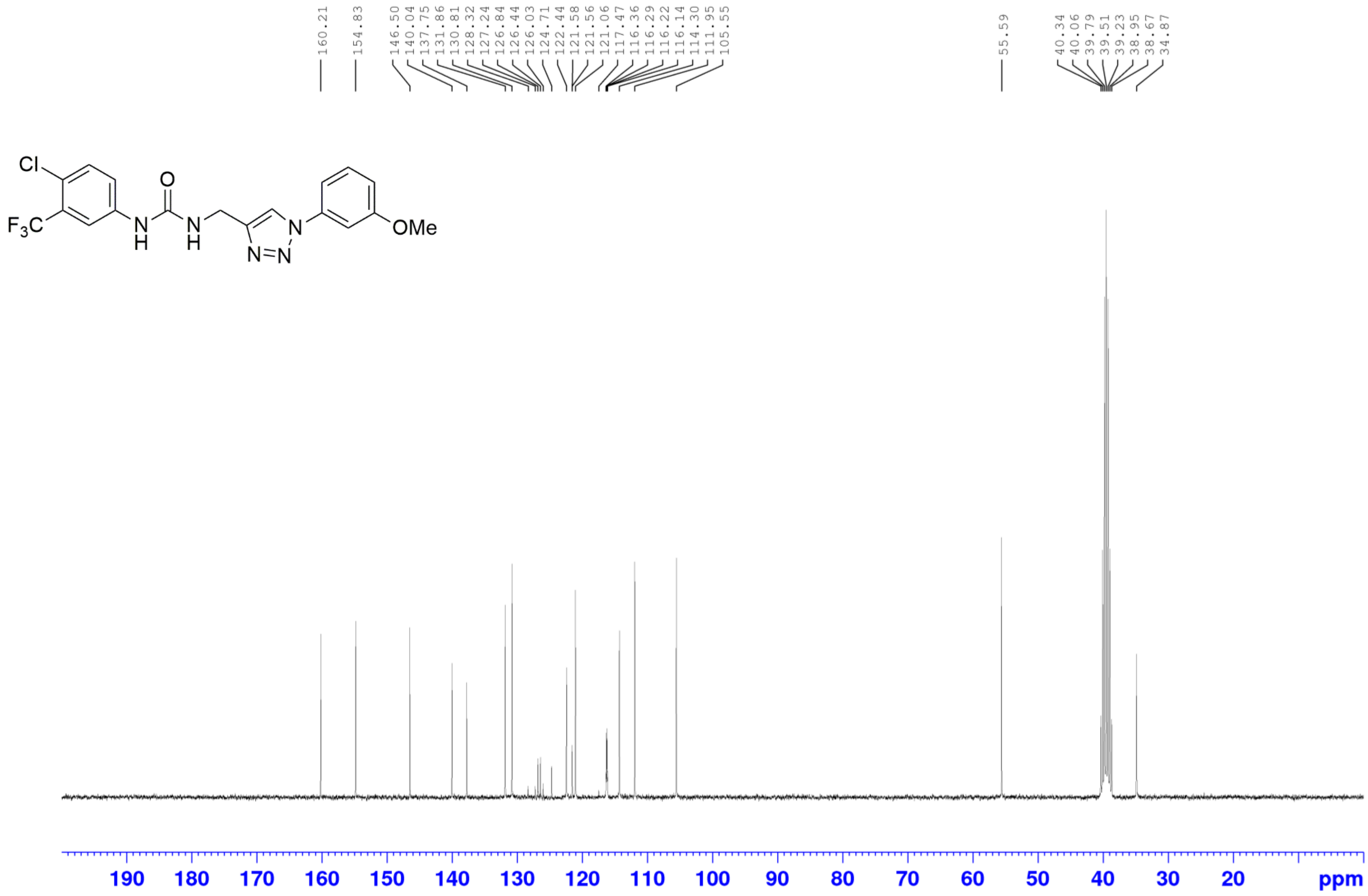

^{19}F NMR of compound **2r** (282 MHz, DMSO-d_6)

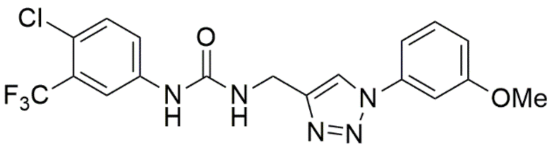

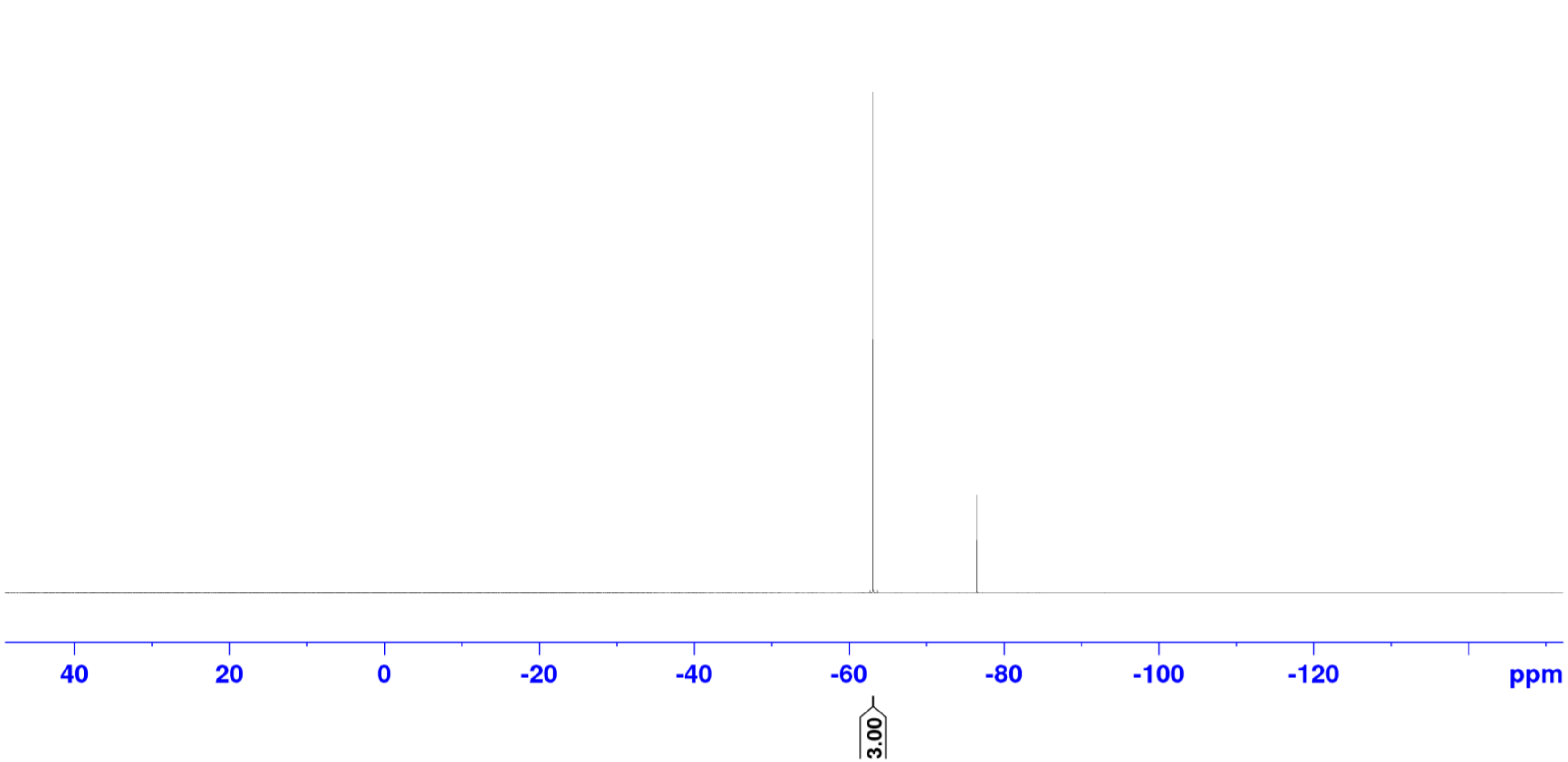

^{1}H NMR of compound **2s** (300 MHz, DMSO-d_6)

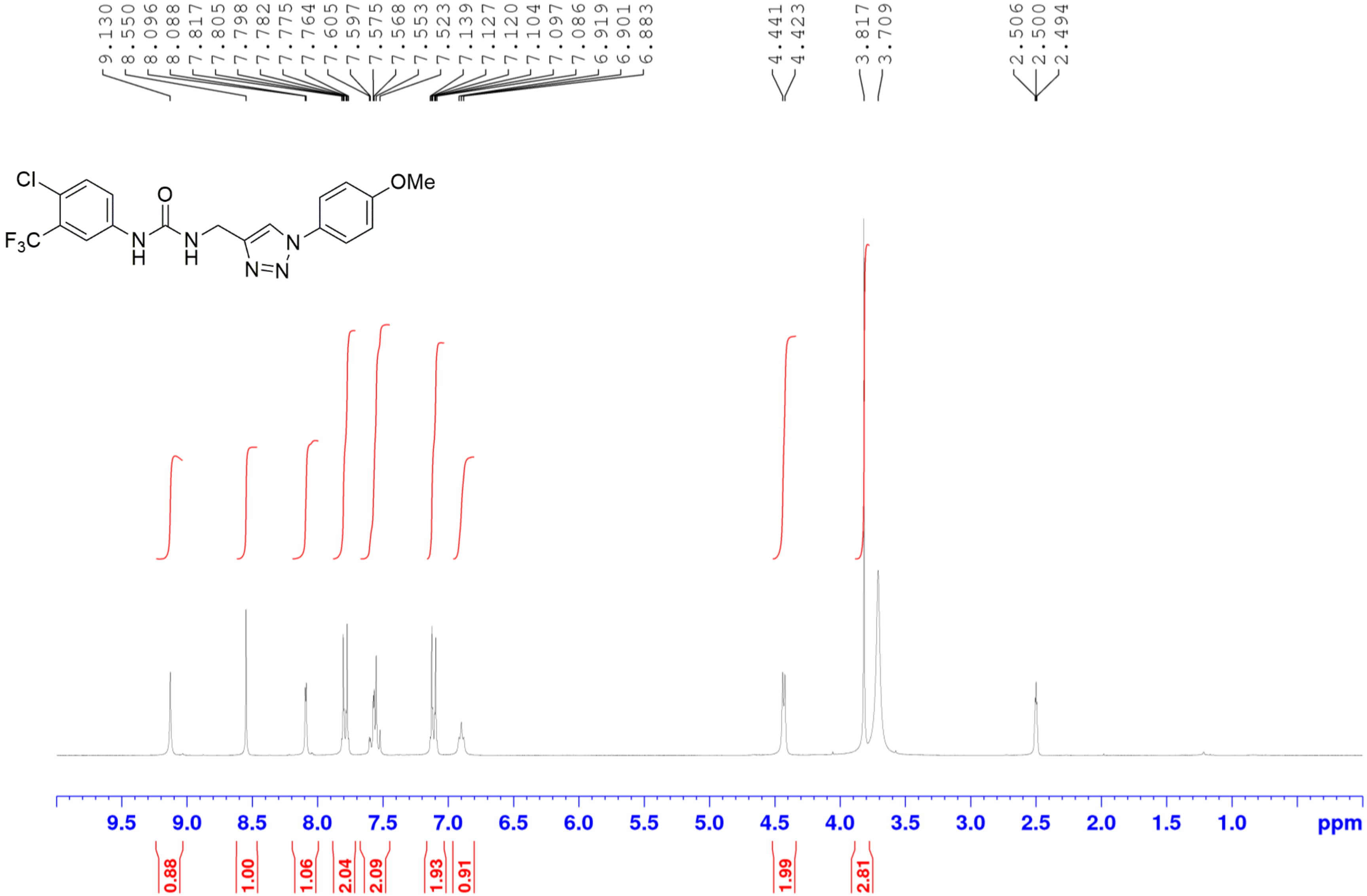

^{13}C NMR of compound **2s** (75 MHz, DMSO-d_6)

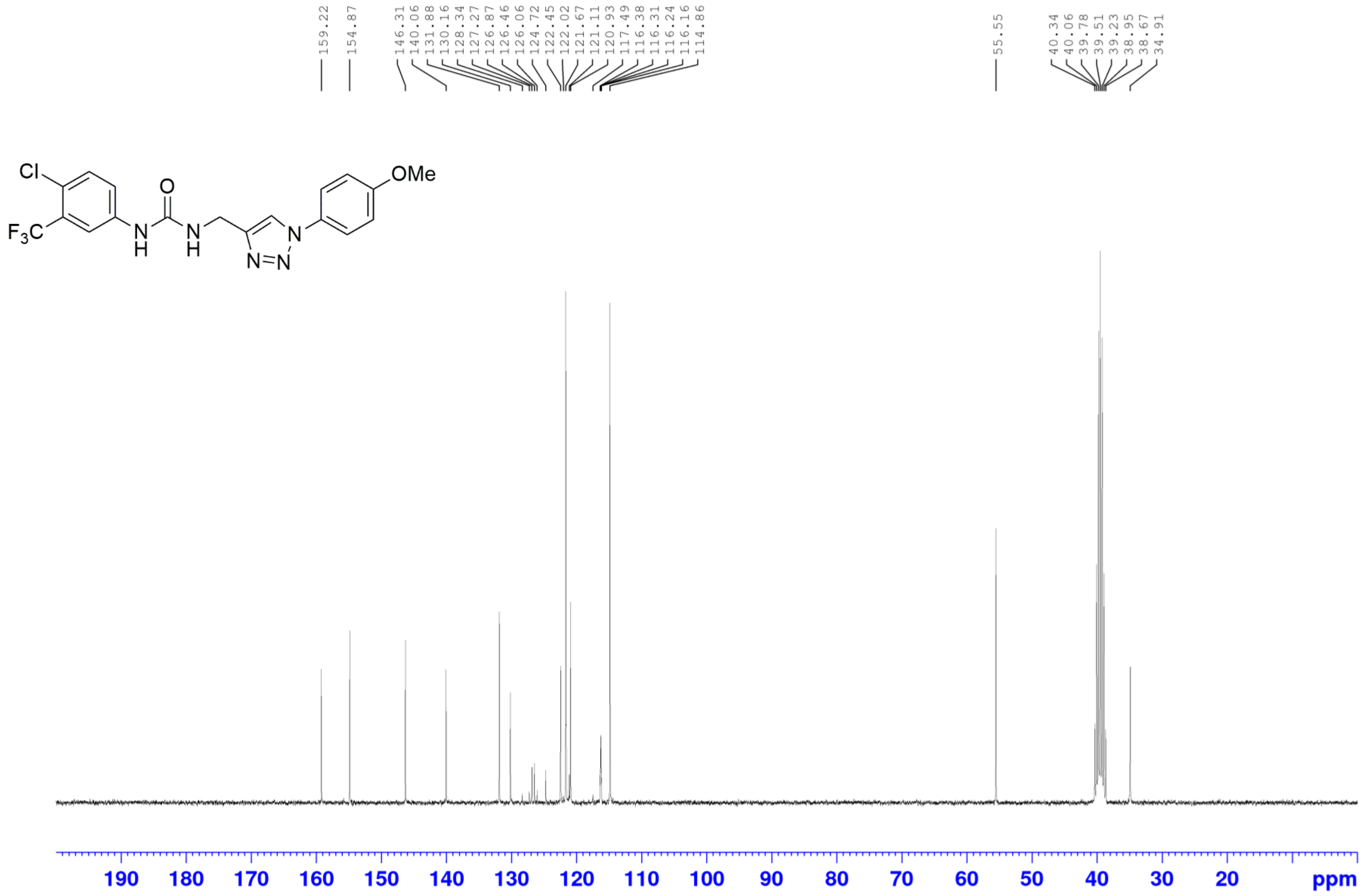

^{19}F NMR of compound **2s** (282 MHz, DMSO-d_6)

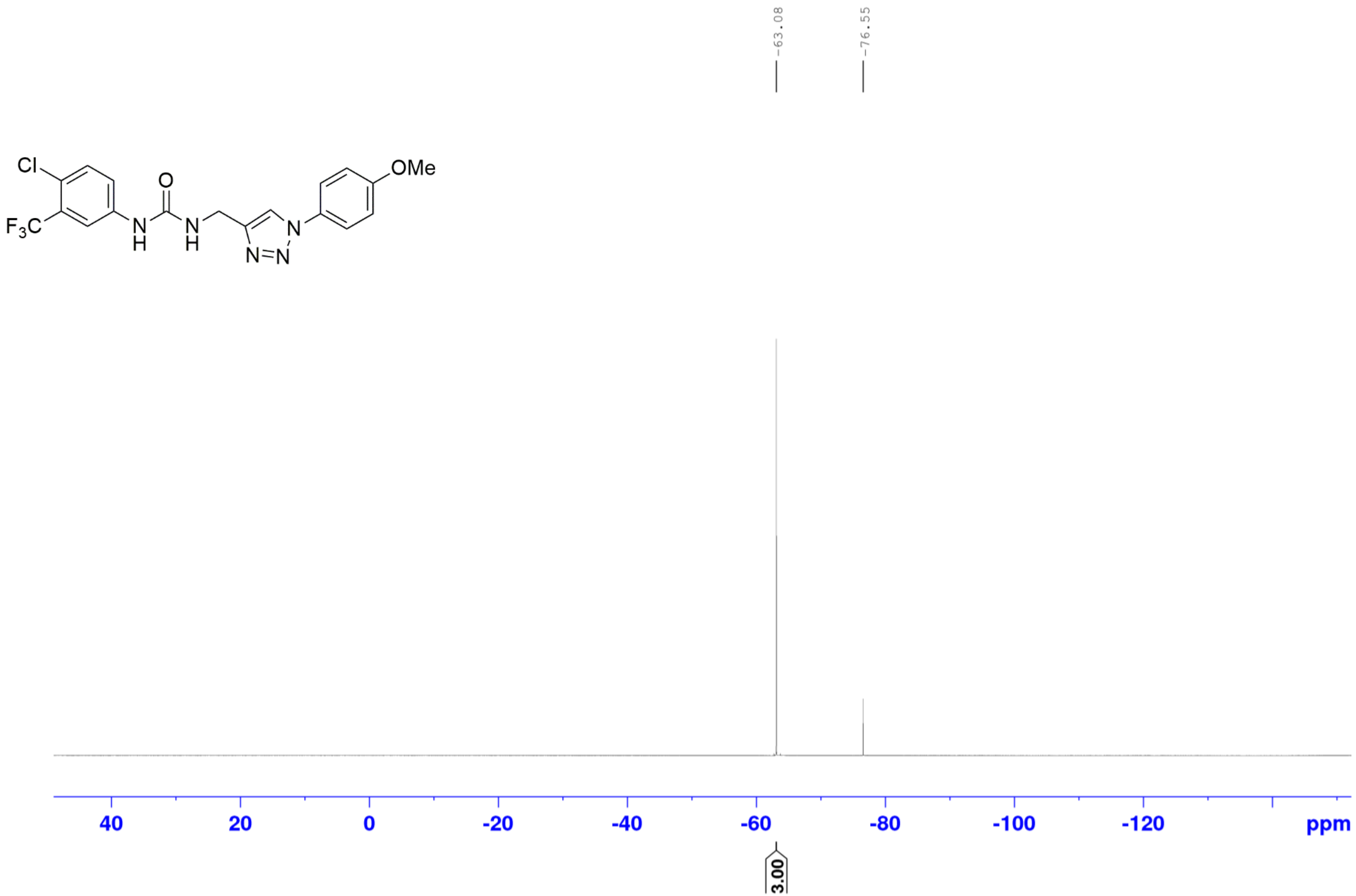

^{1}H NMR of compound **2t** (300 MHz, DMSO-d_6)

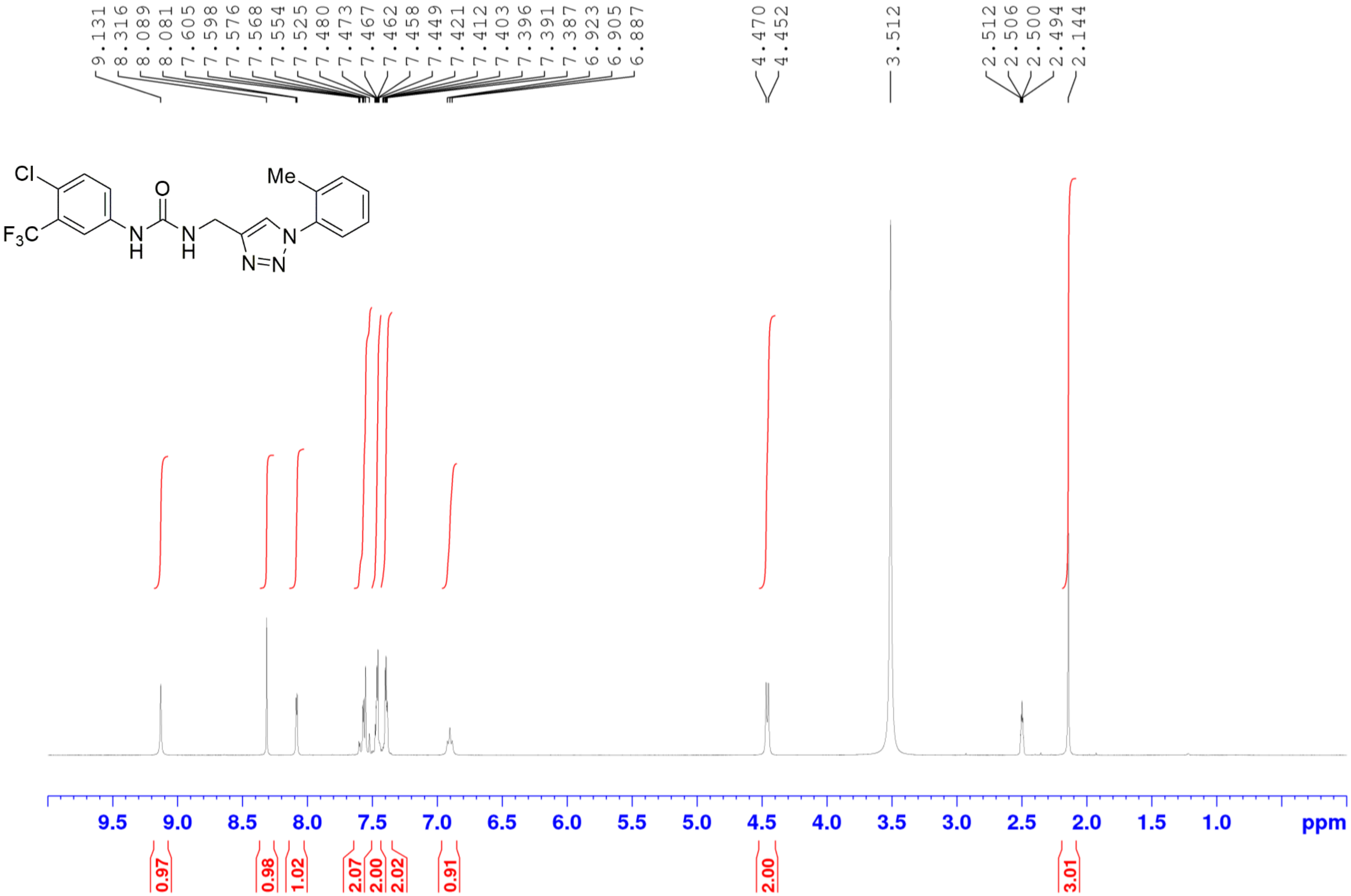

^{13}C NMR of compound **2t** (75 MHz, DMSO-*d*$_6$)

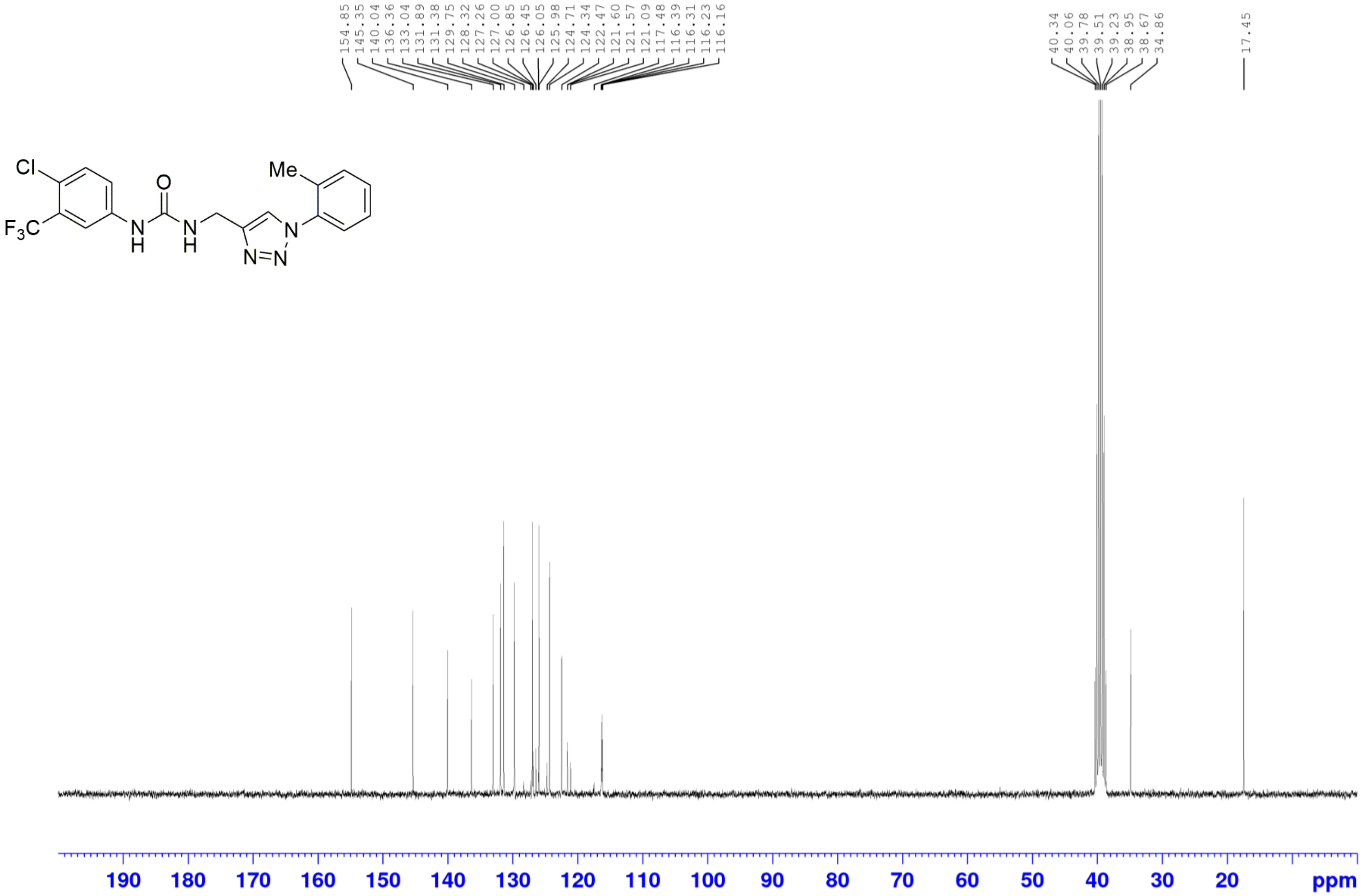

^{19}F NMR of compound **2t** (282 MHz, DMSO-d_6)

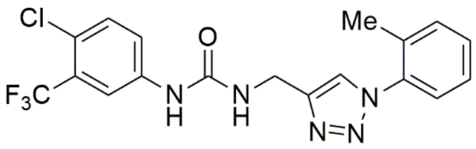

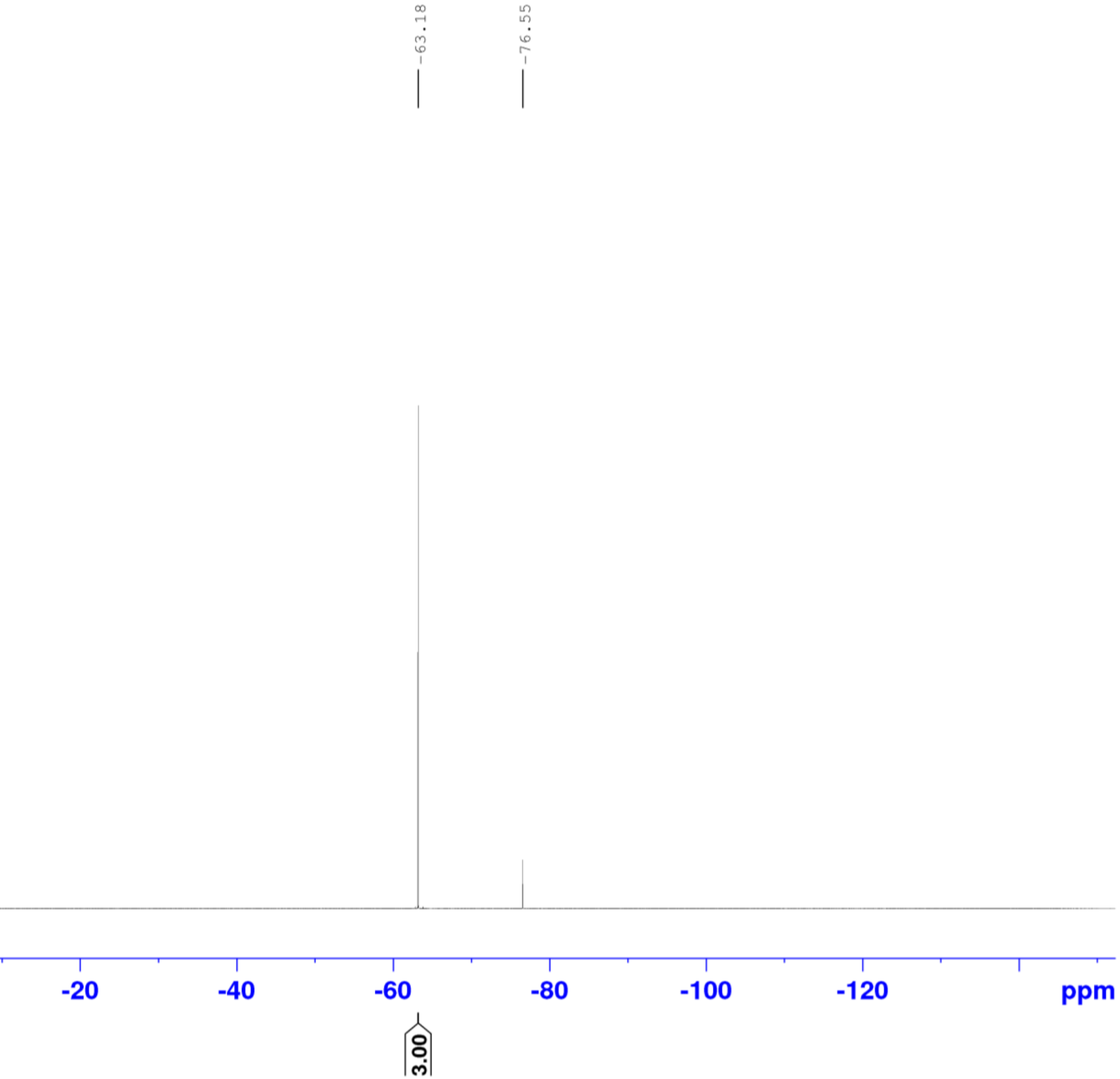

^{1}H NMR of compound **2u** (300 MHz, DMSO-d_6)

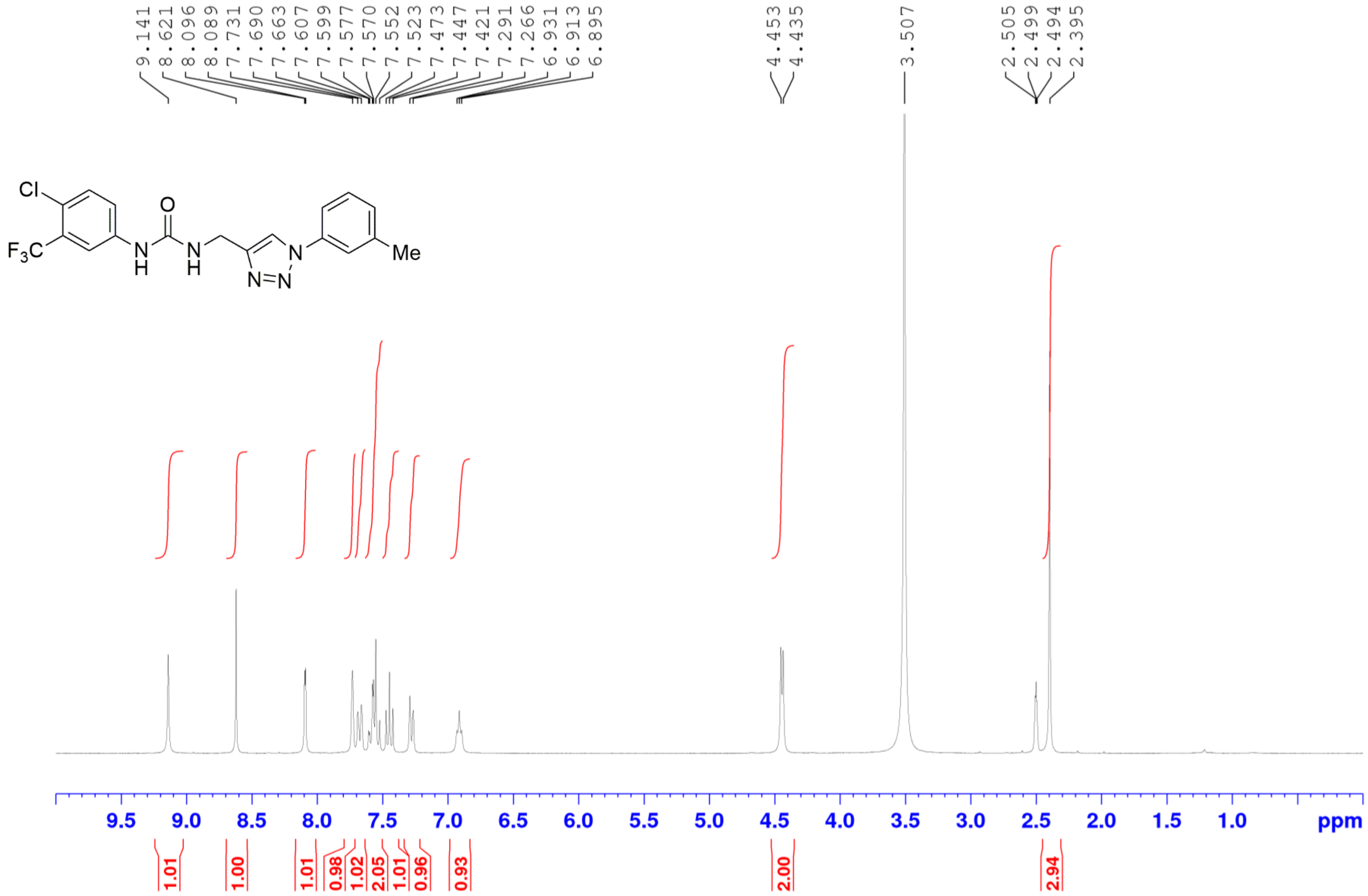

^{13}C NMR of compound **2u** (75 MHz, DMSO-d_6)

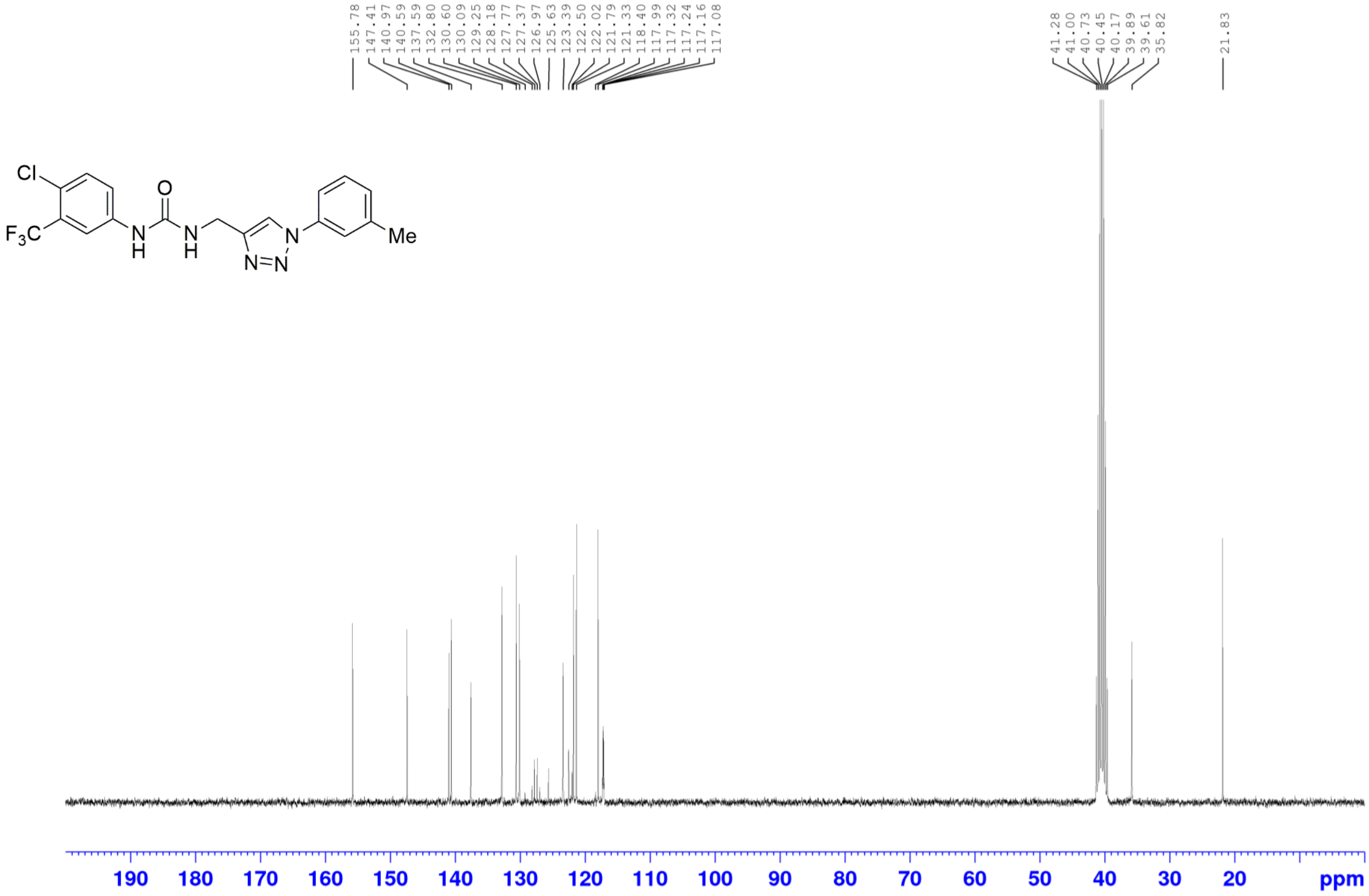

^{19}F NMR of compound **2u** (282 MHz, DMSO-d_6)

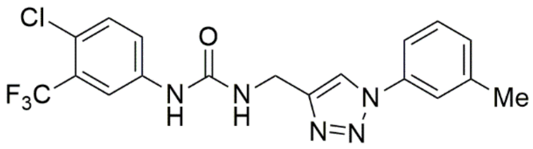

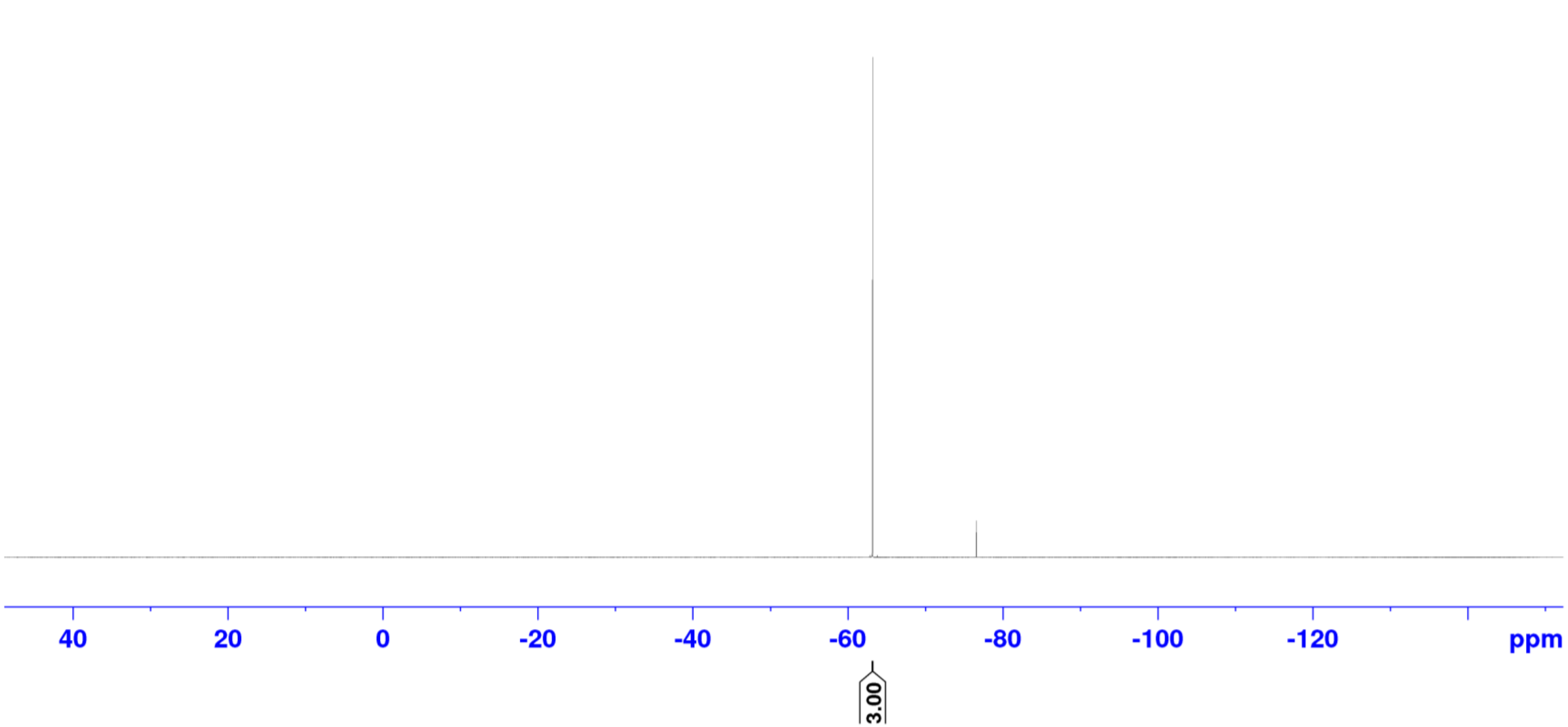

^{1}H NMR of compound **2v** (300 MHz, DMSO-d_6)

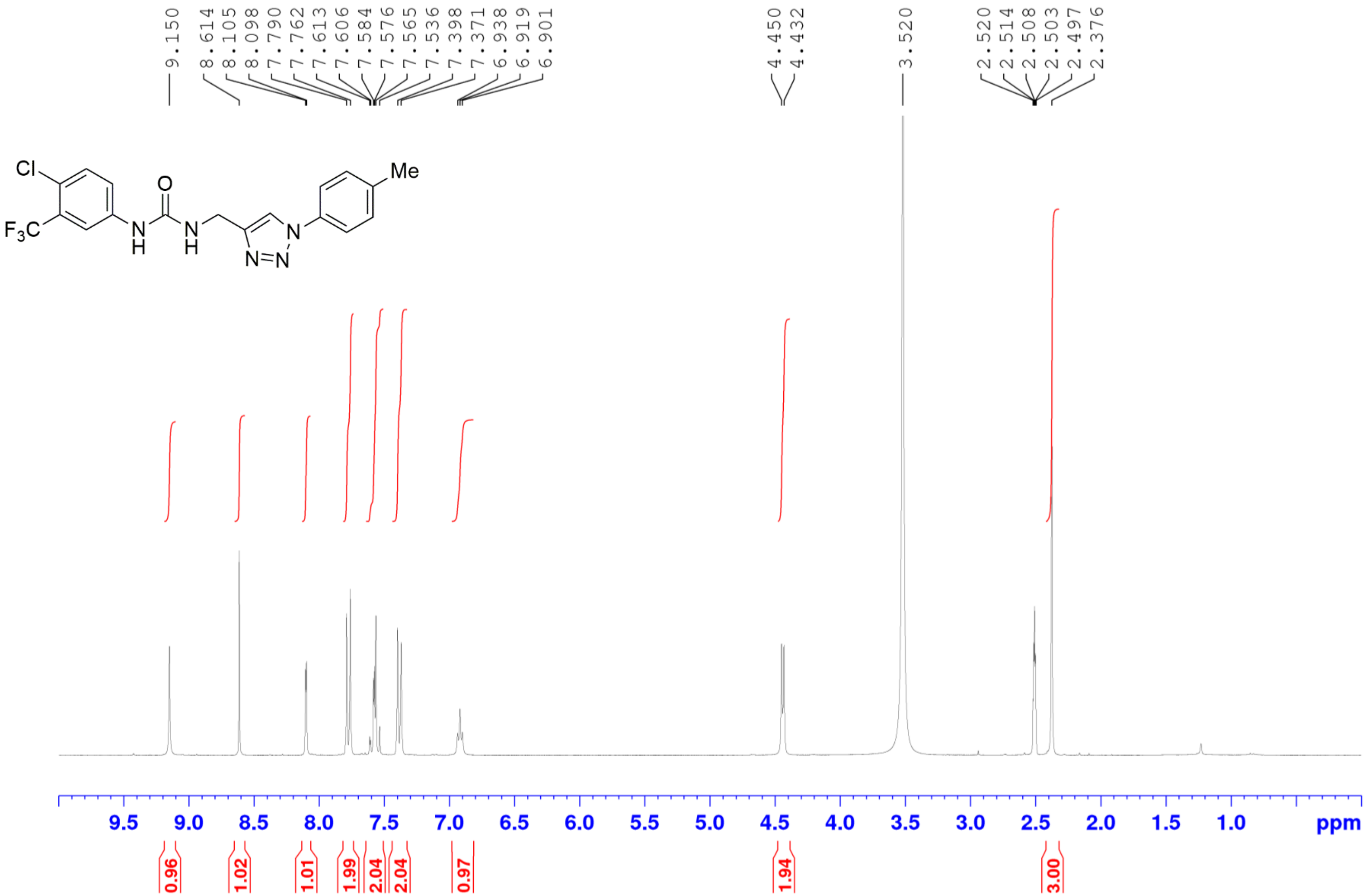

^{13}C NMR of compound **2v** (75 MHz, DMSO-d_6)

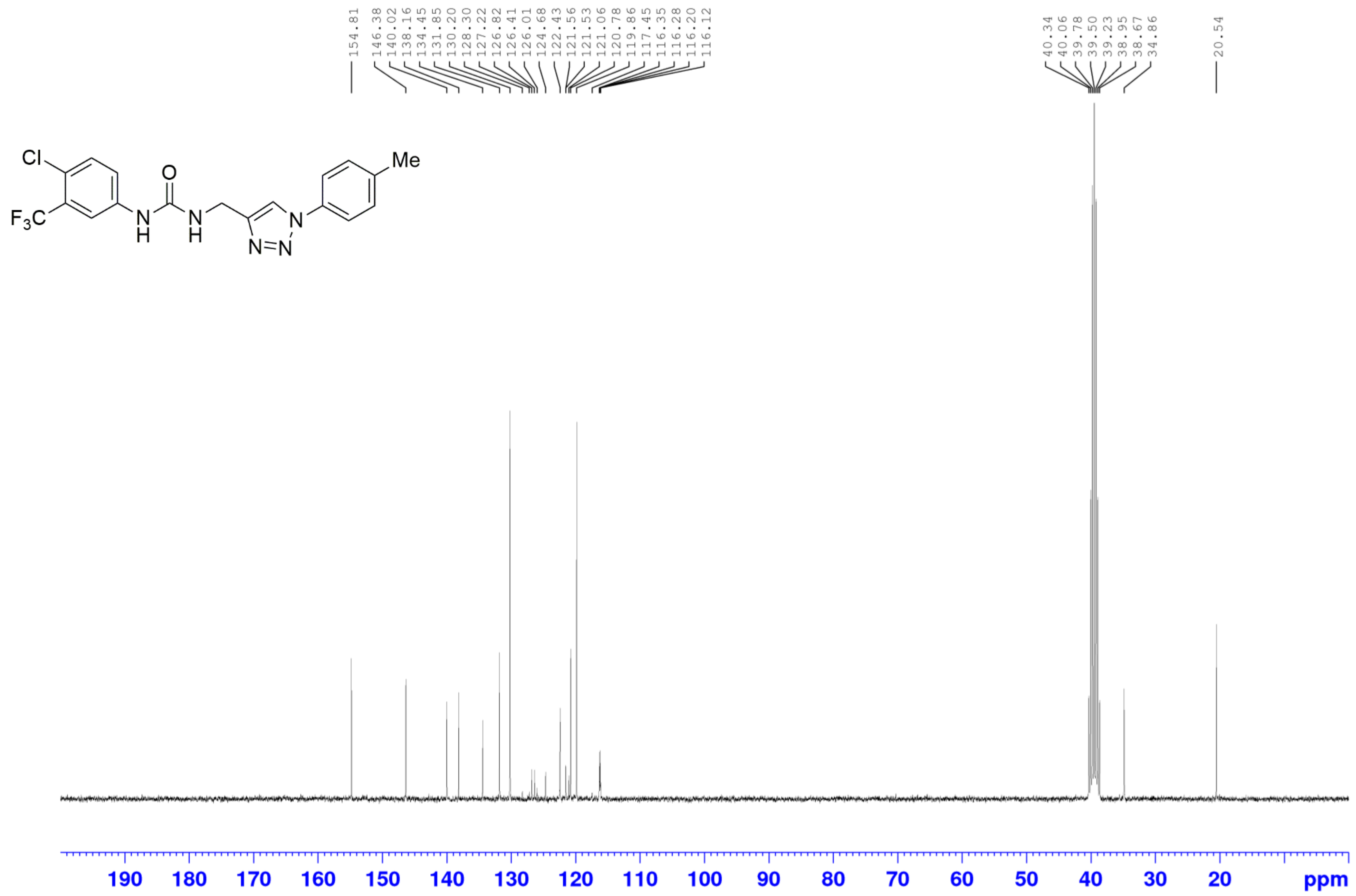

^{19}F NMR of compound **2v** (282 MHz, DMSO-d_6)

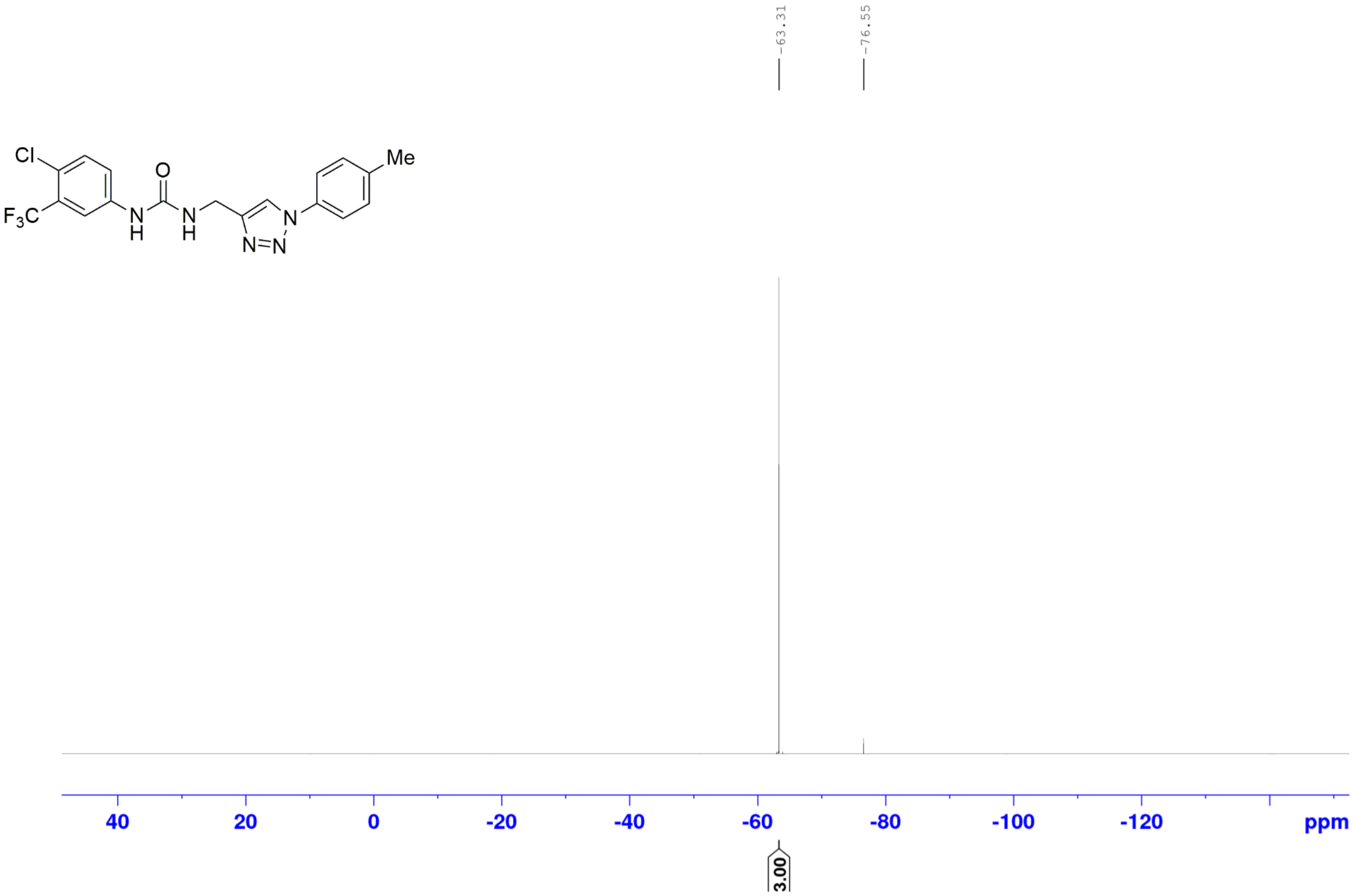

^{1}H NMR of compound **2w** (300 MHz, DMSO-d_6)

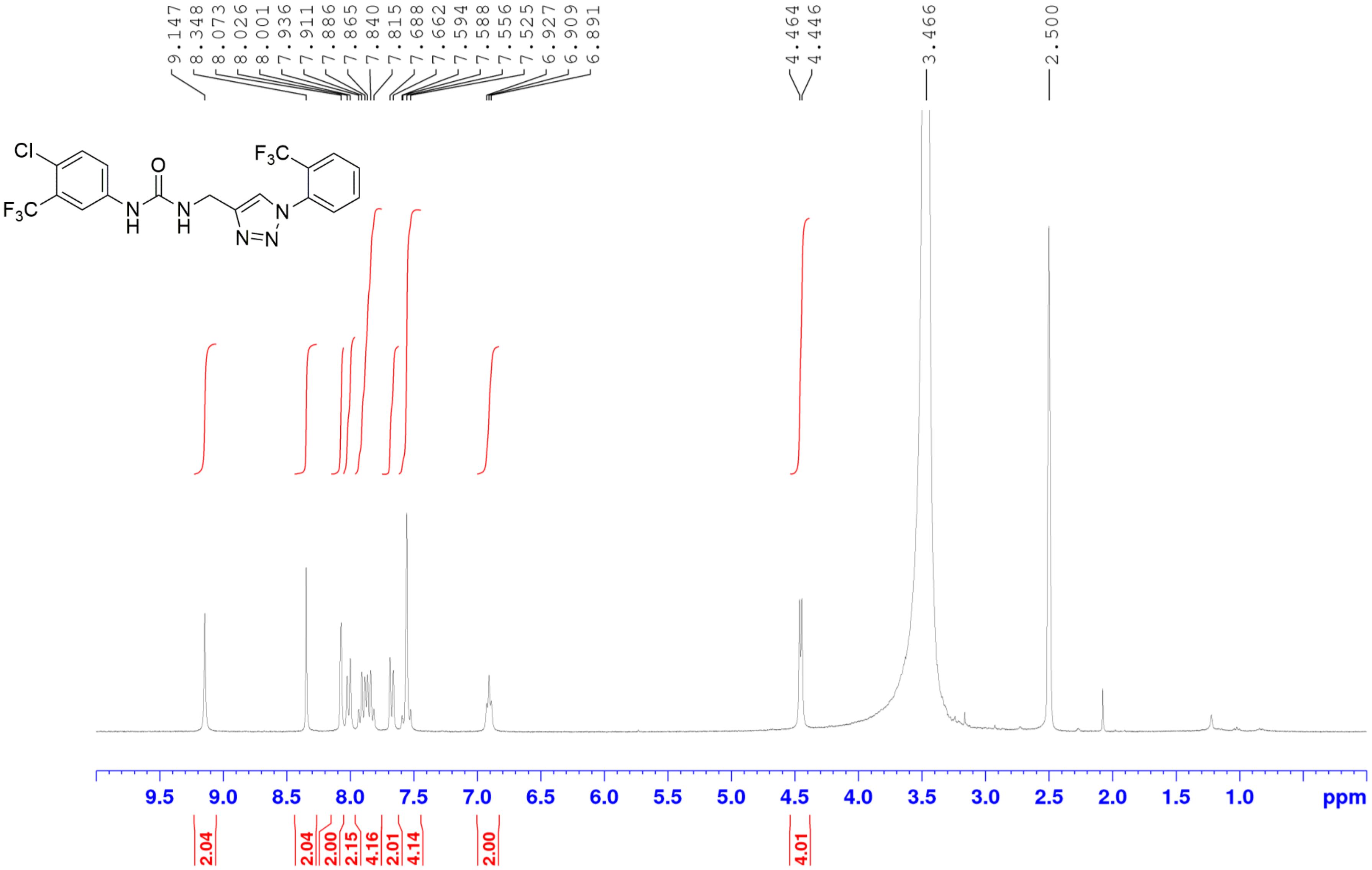

^{13}C NMR of compound **2w** (75 MHz, DMSO-d_6)

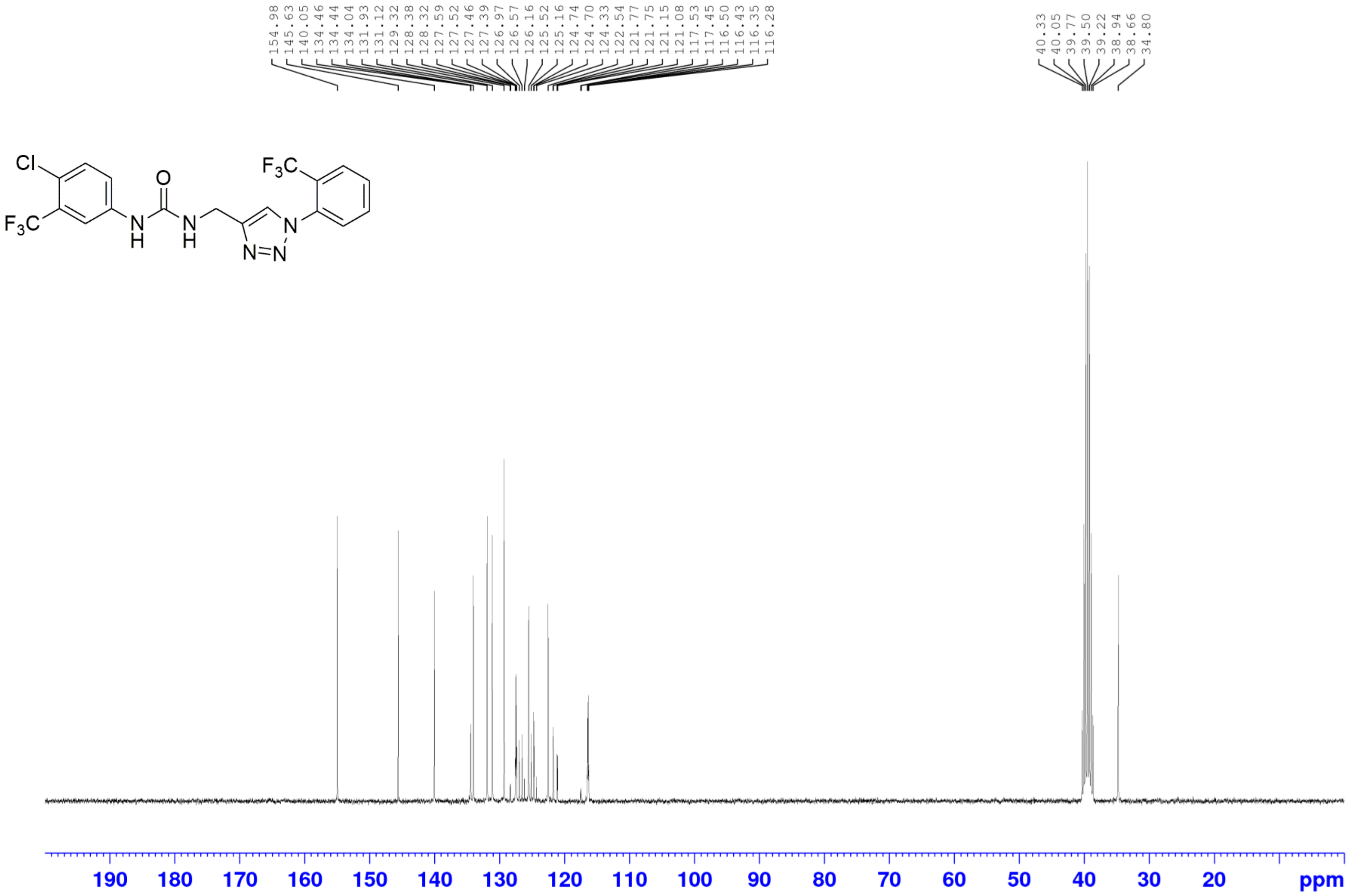

^{19}F NMR of compound **2w** (282 MHz, DMSO-d_6)

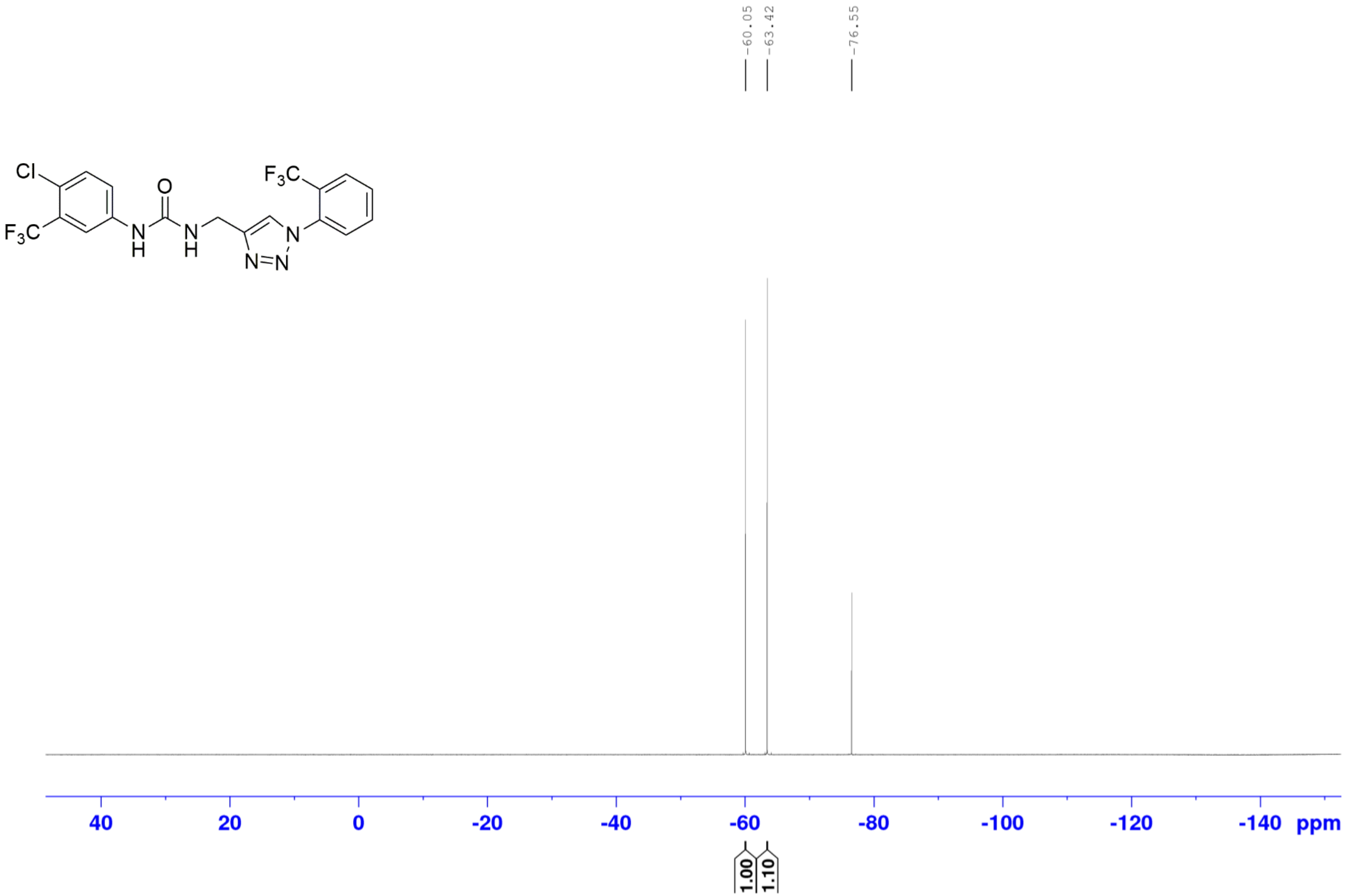

^{1}H NMR of compound **2x** (300 MHz, DMSO-d_6)

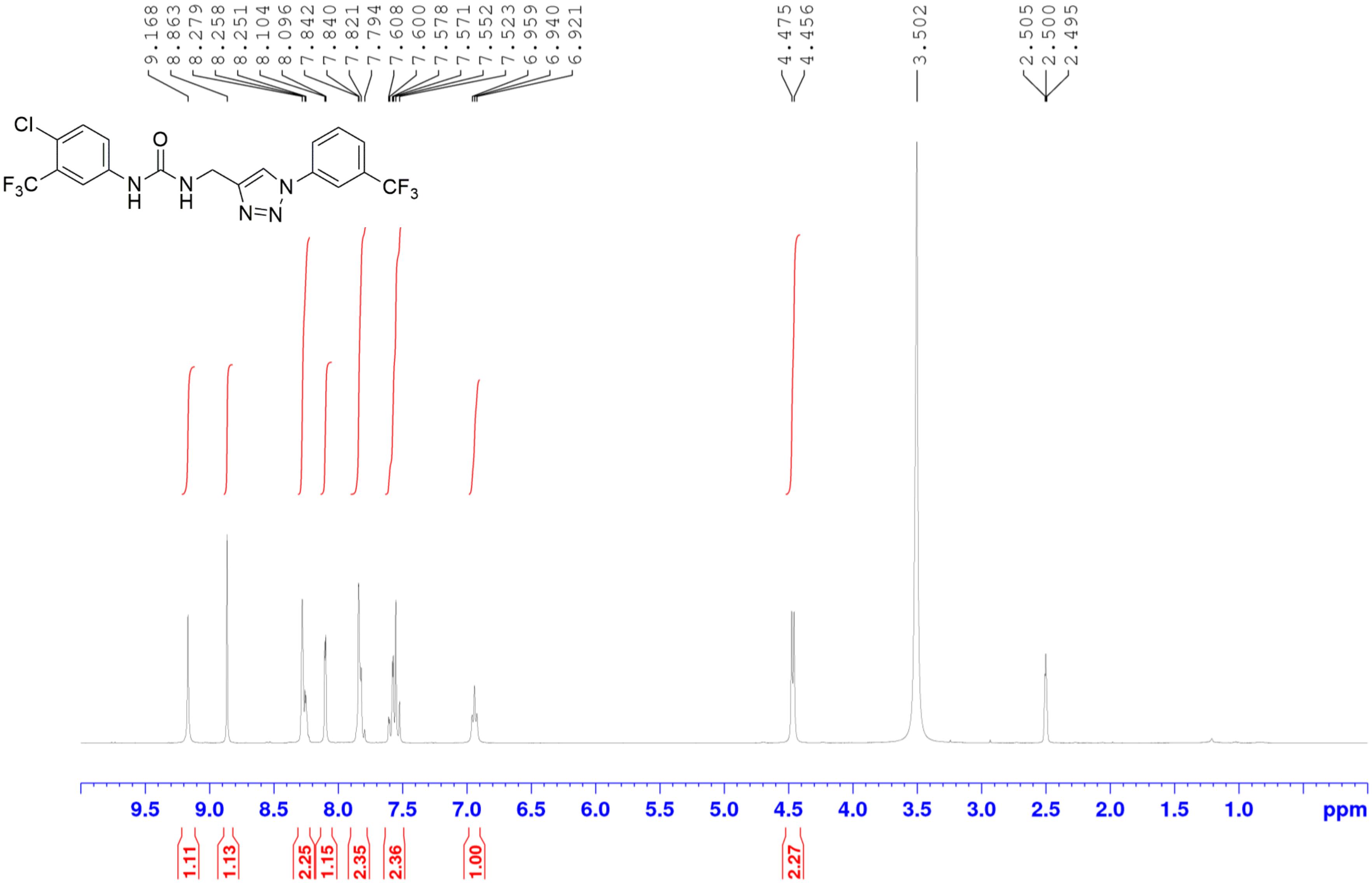

^{13}C NMR of compound **2x** (75 MHz, DMSO-d_6)

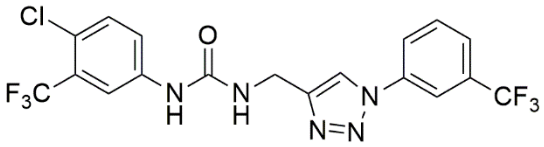

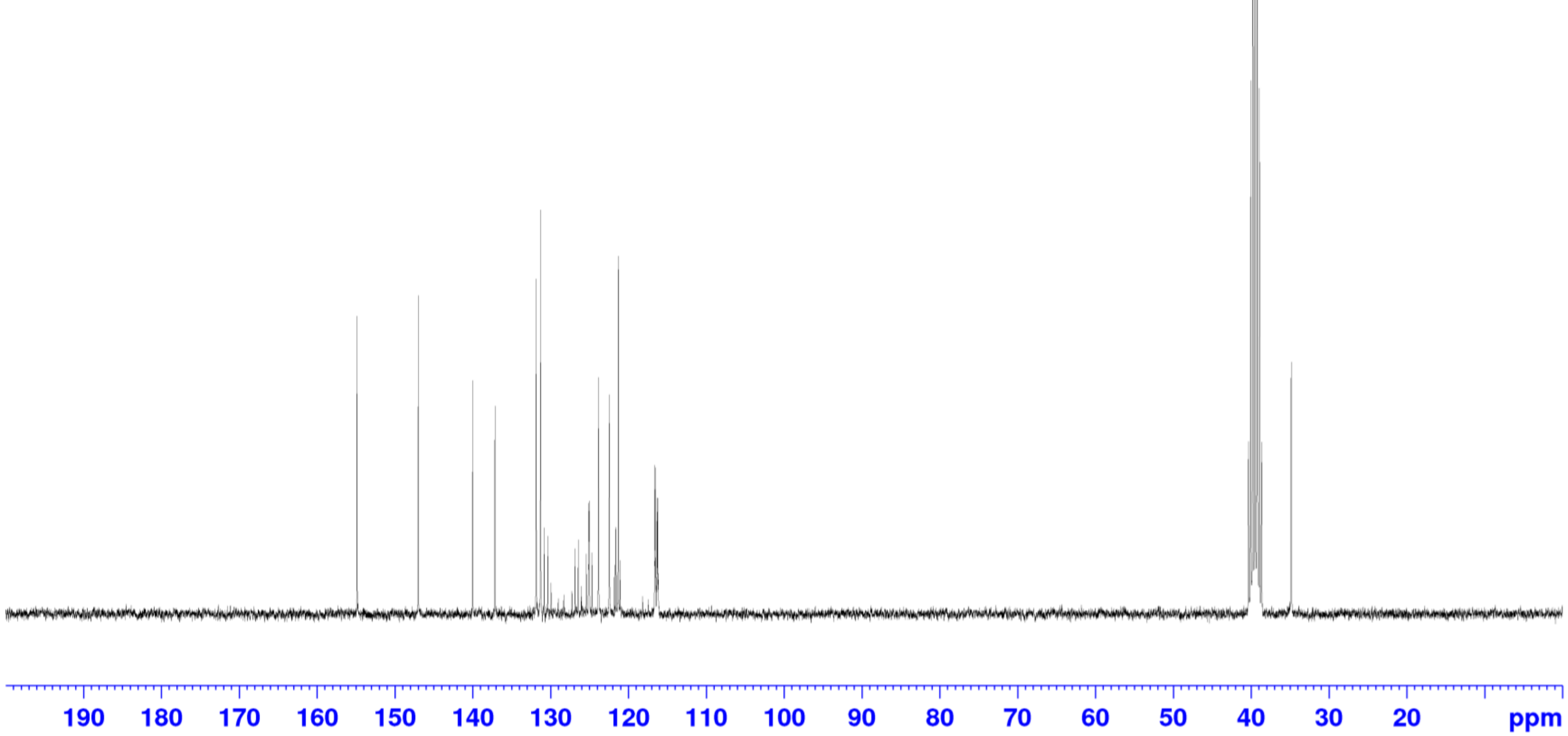

^{19}F NMR of compound **2x** (282 MHz, DMSO-d_6)

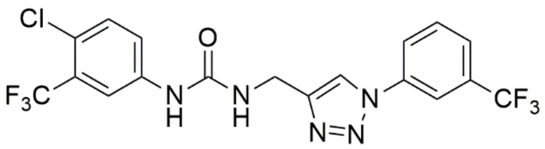

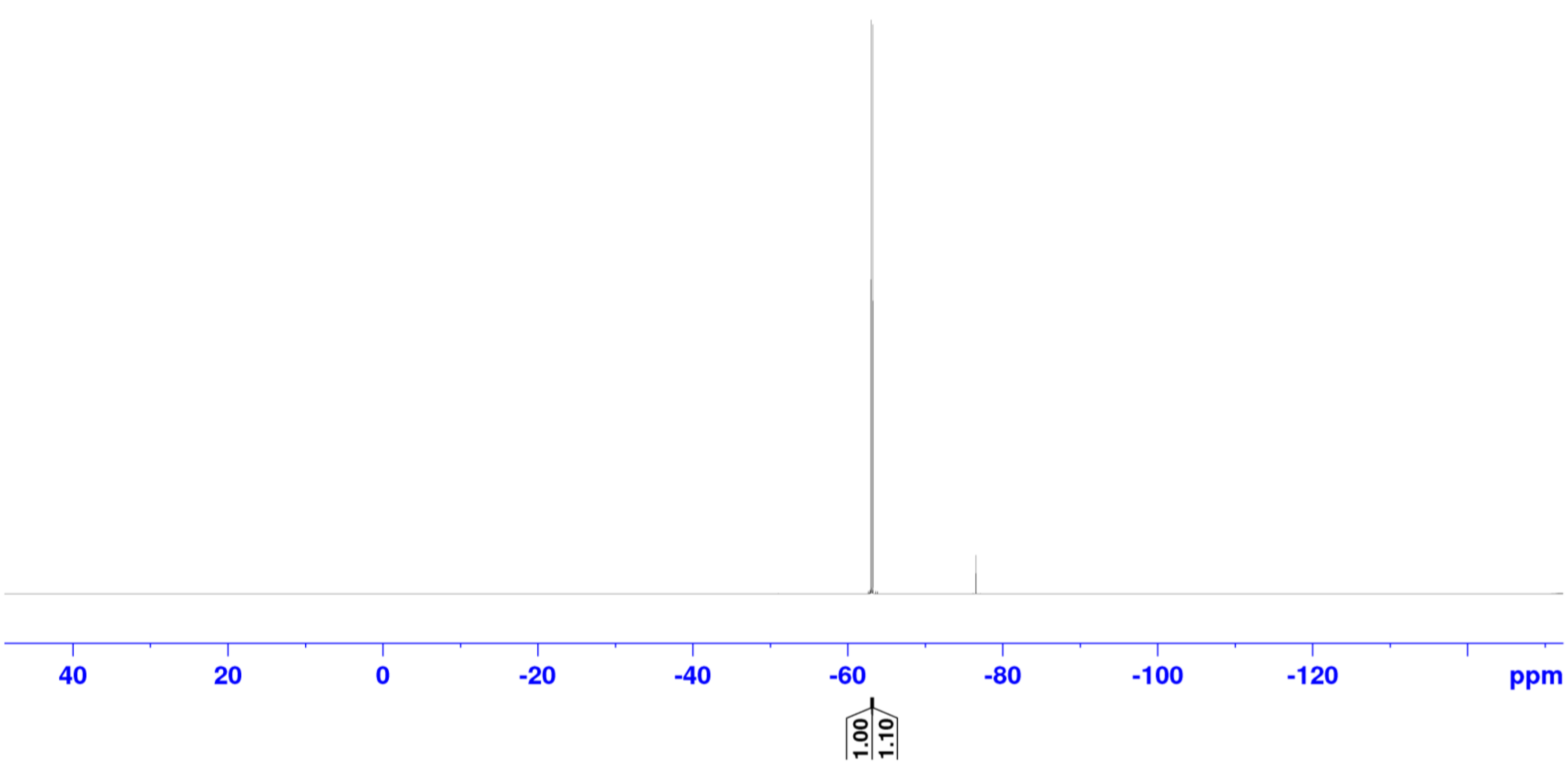

^{1}H NMR of compound **2y** (300 MHz, DMSO-d_6)

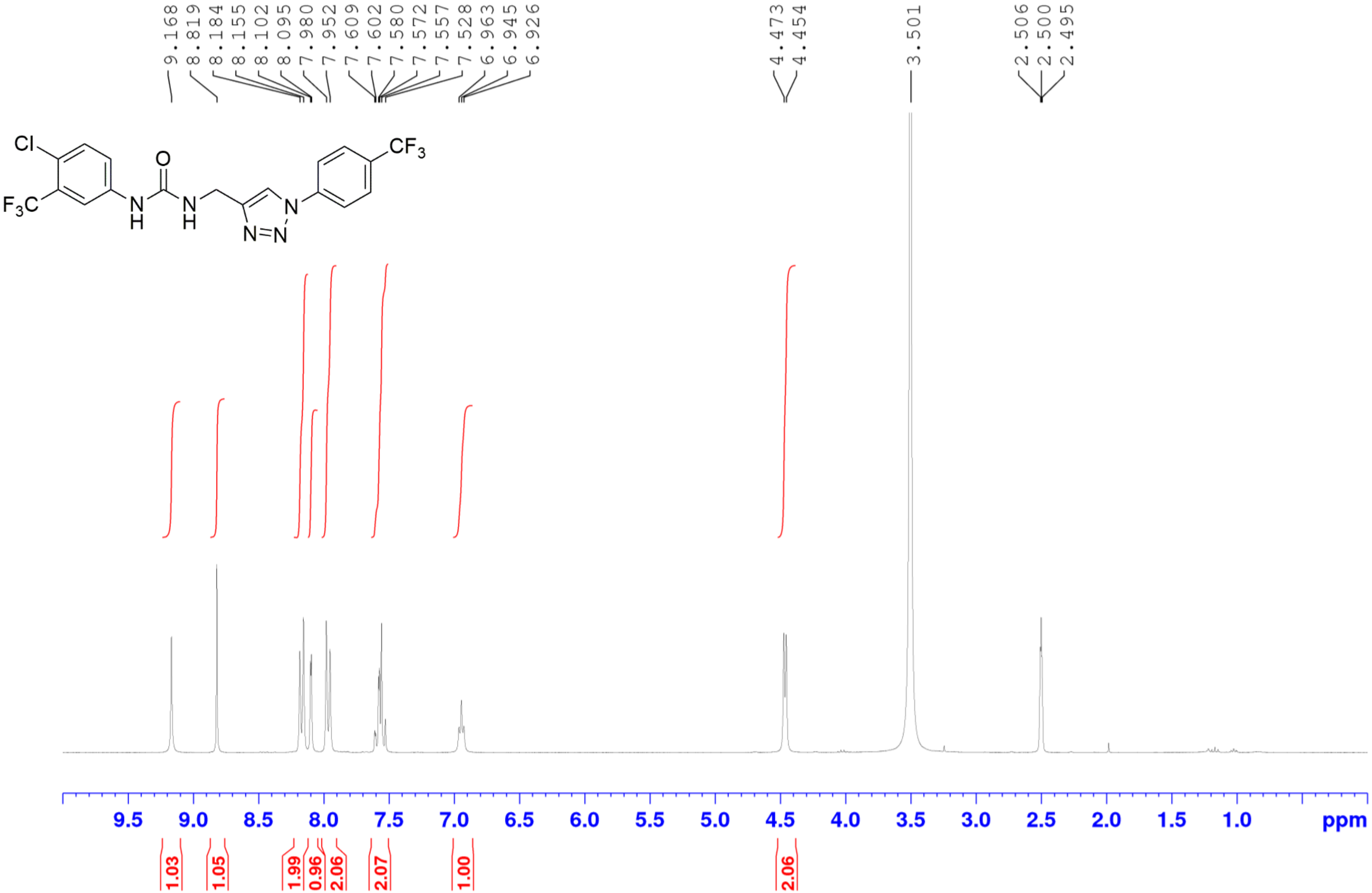

^{13}C NMR of compound **2y** (75 MHz, DMSO-d_6)

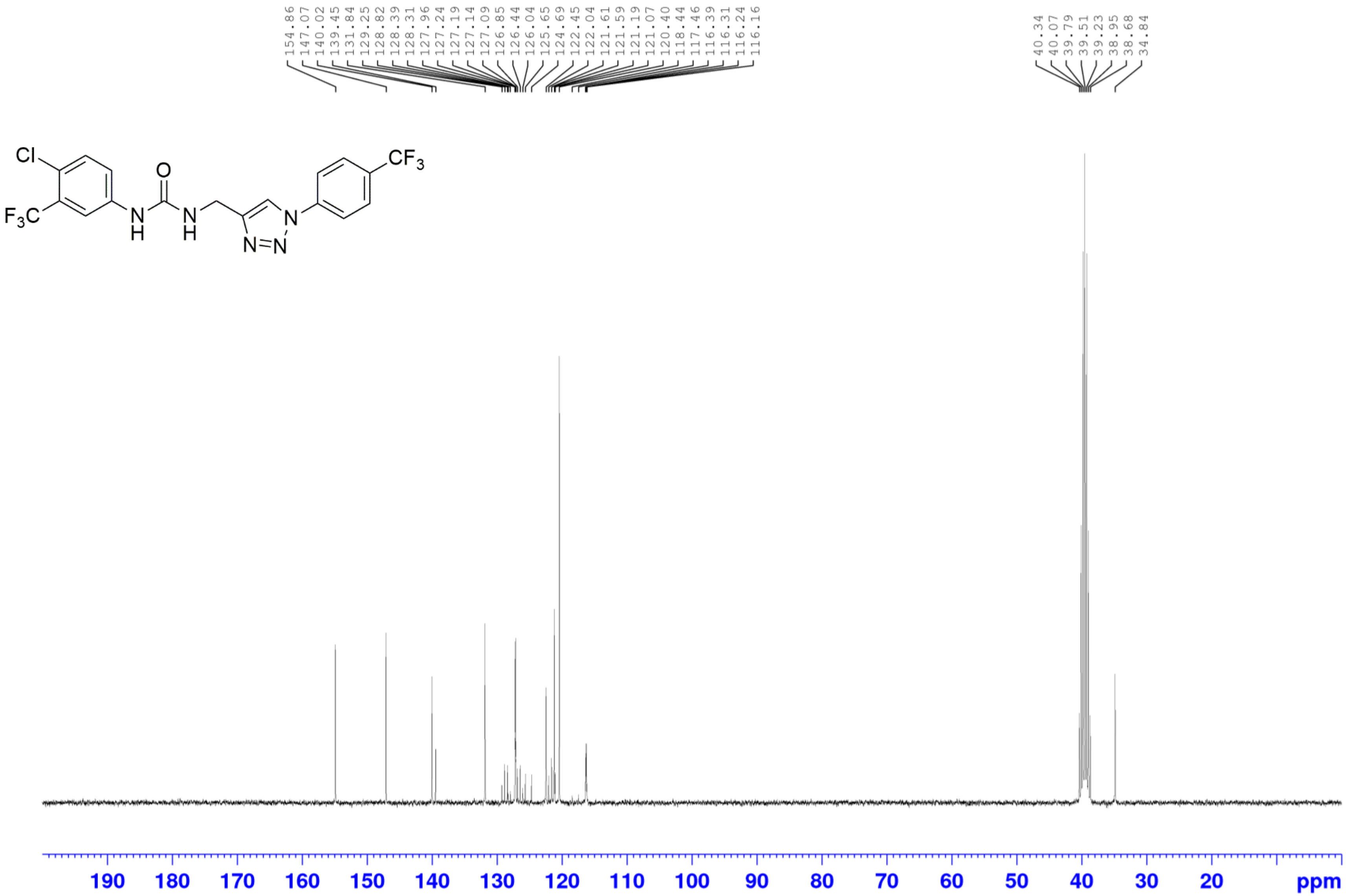

^{19}F NMR of compound **2y** (282 MHz, DMSO-d_6)

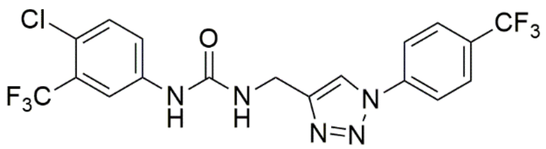

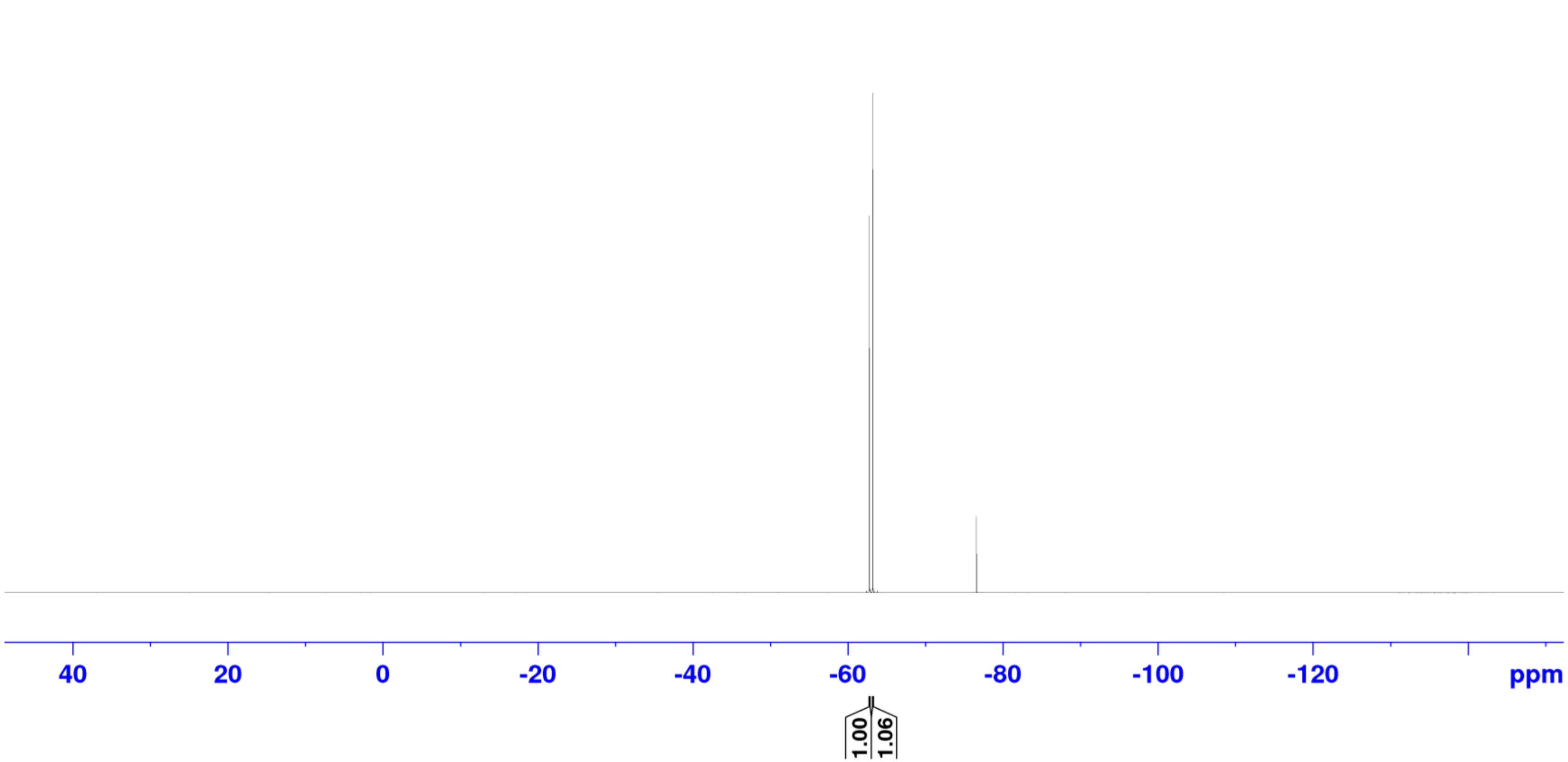

^{1}H NMR of compound **2z** (300 MHz, DMSO-d_6)

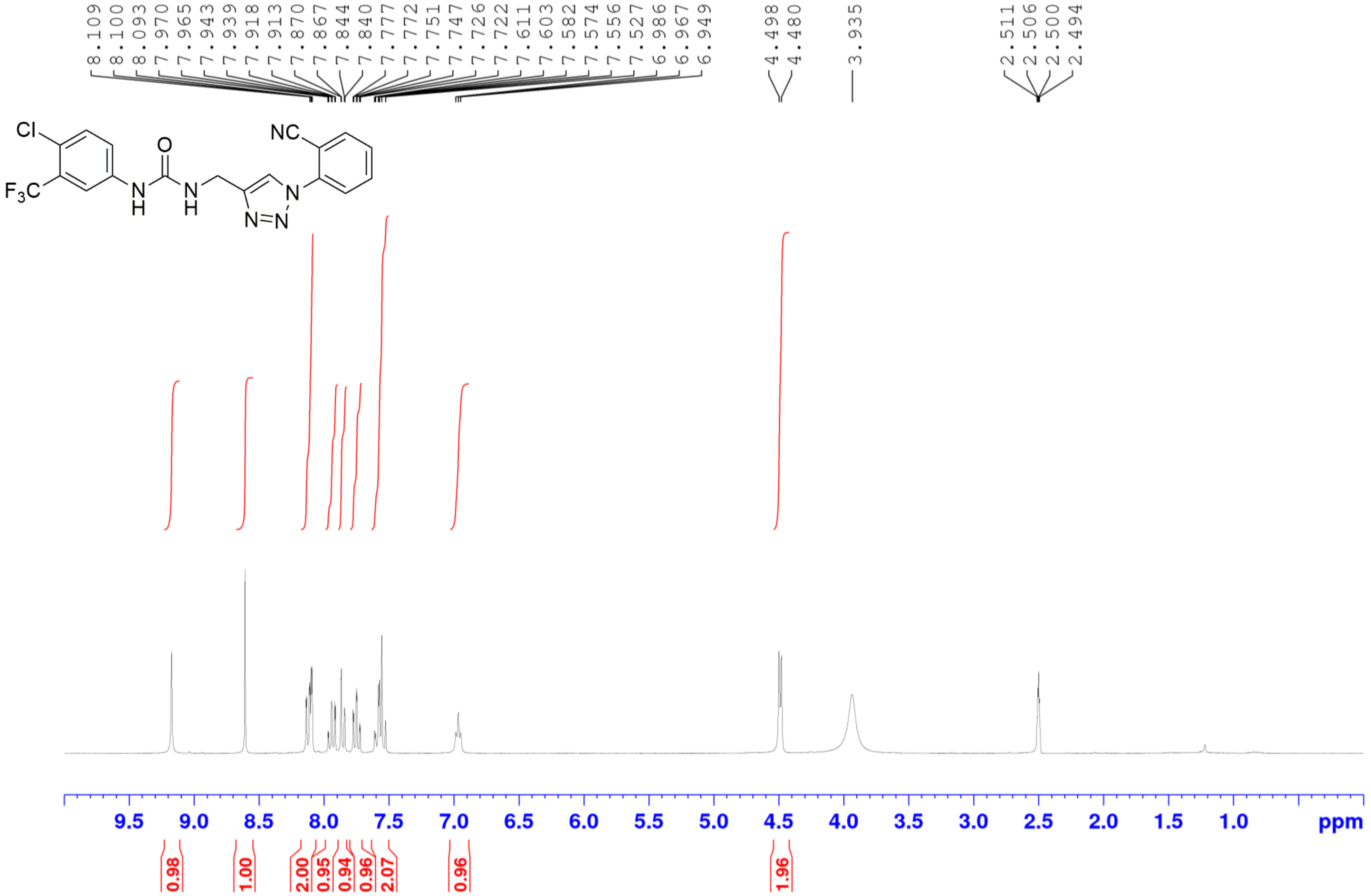

^{13}C NMR of compound **2z** (75 MHz, DMSO-d_6)

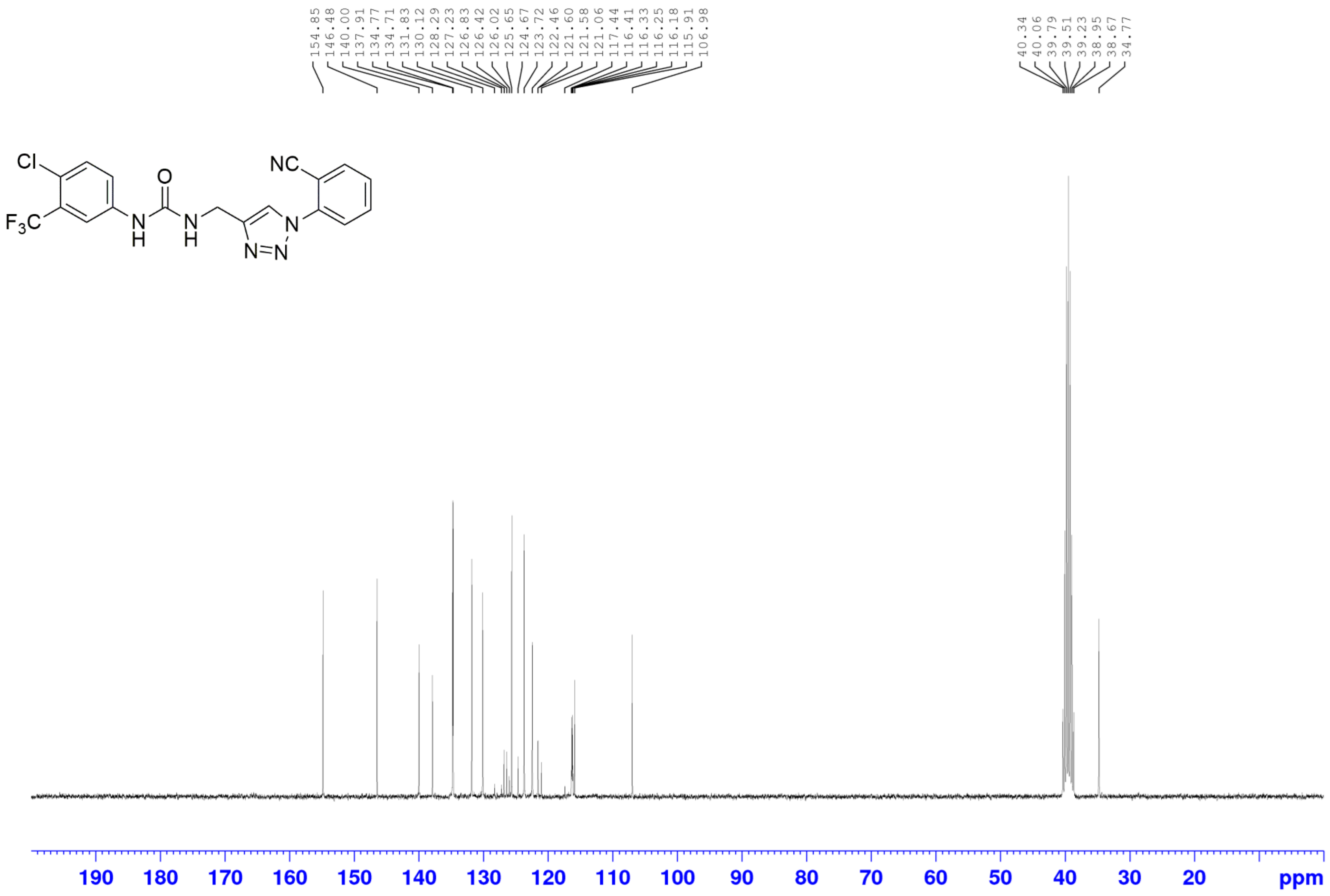

^{19}F NMR of compound **2z** (282 MHz, DMSO-d_6)

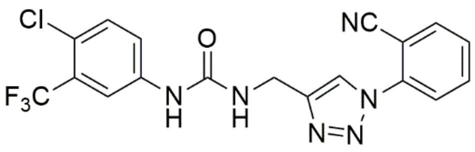

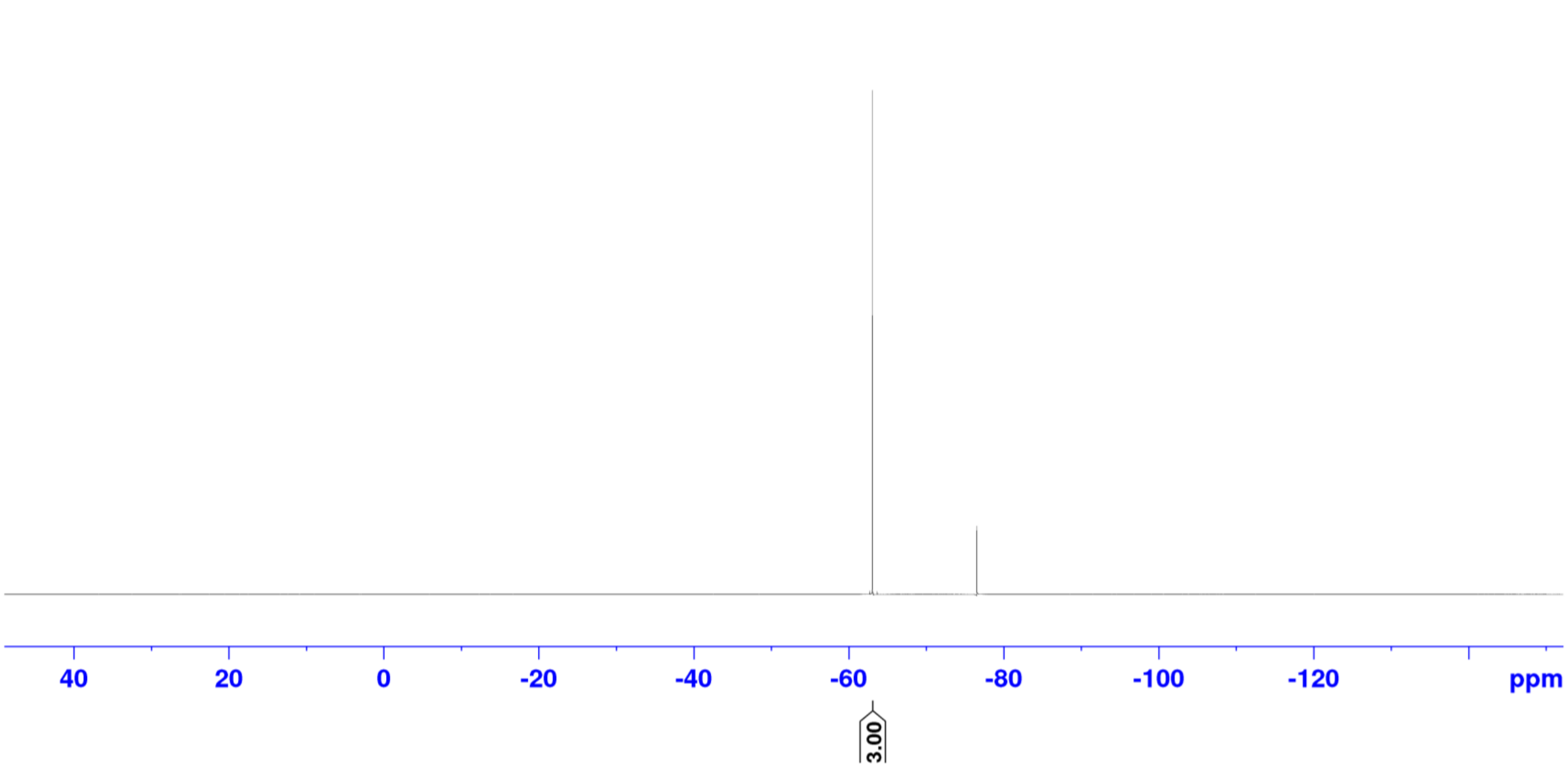

^{1}H NMR of compound **2a’** (300 MHz, DMSO-d_6)

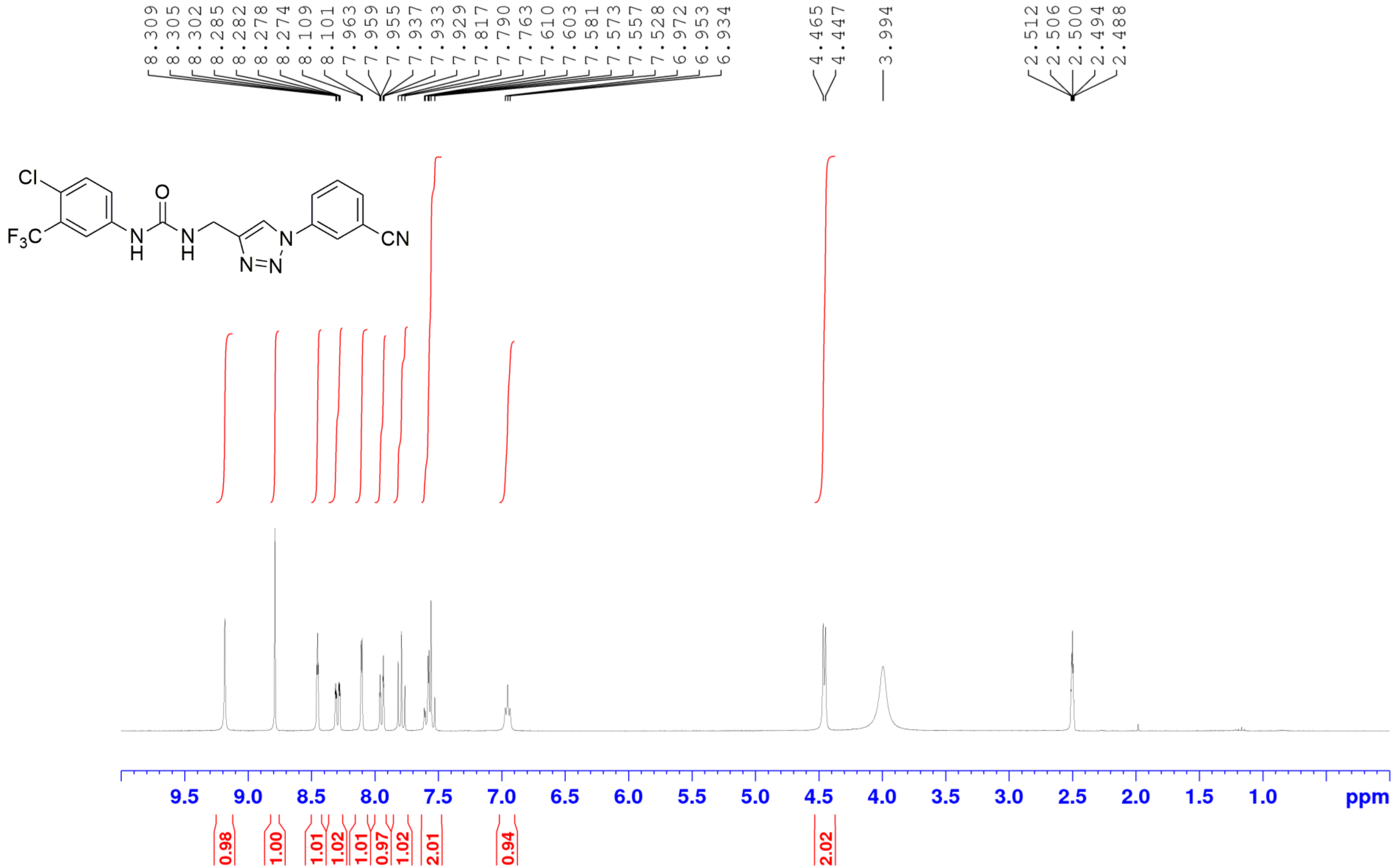

^{13}C NMR of compound **2a’** (75 MHz, DMSO-d_6)

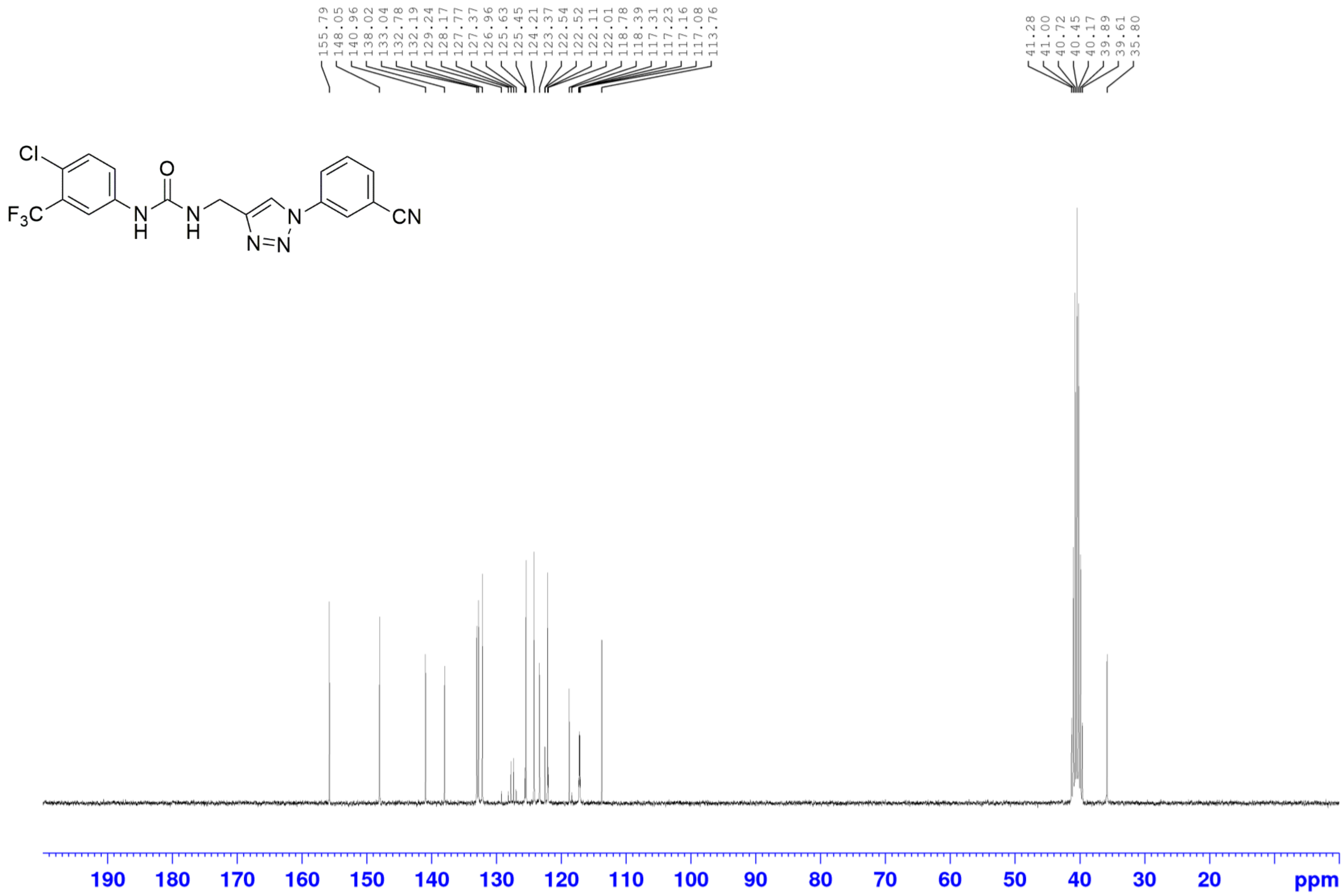

^{19}F NMR of compound **2a’** (282 MHz, DMSO-d_6)

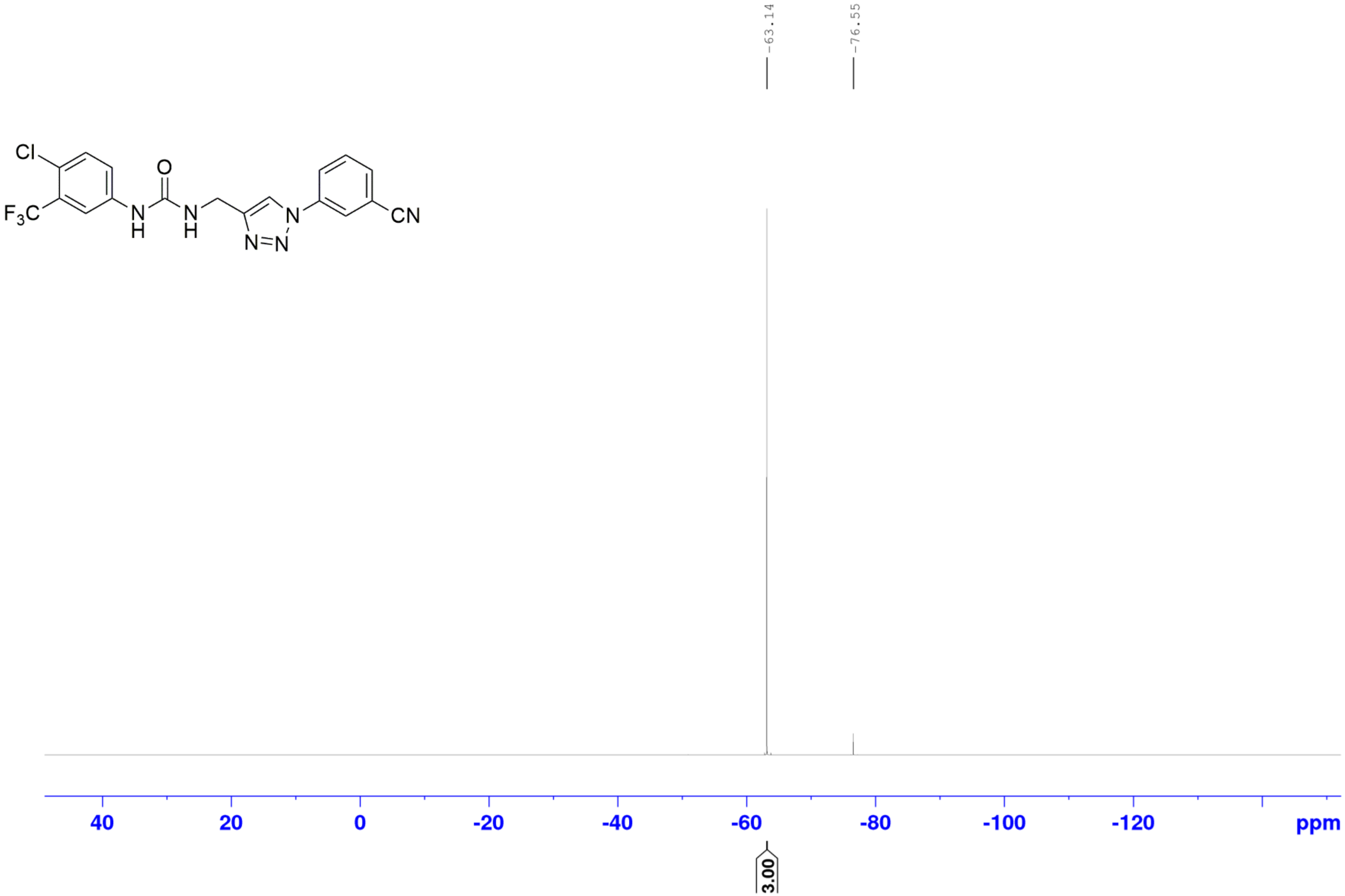

^{1}H NMR of compound **2b'** (300 MHz, DMSO-d_6)

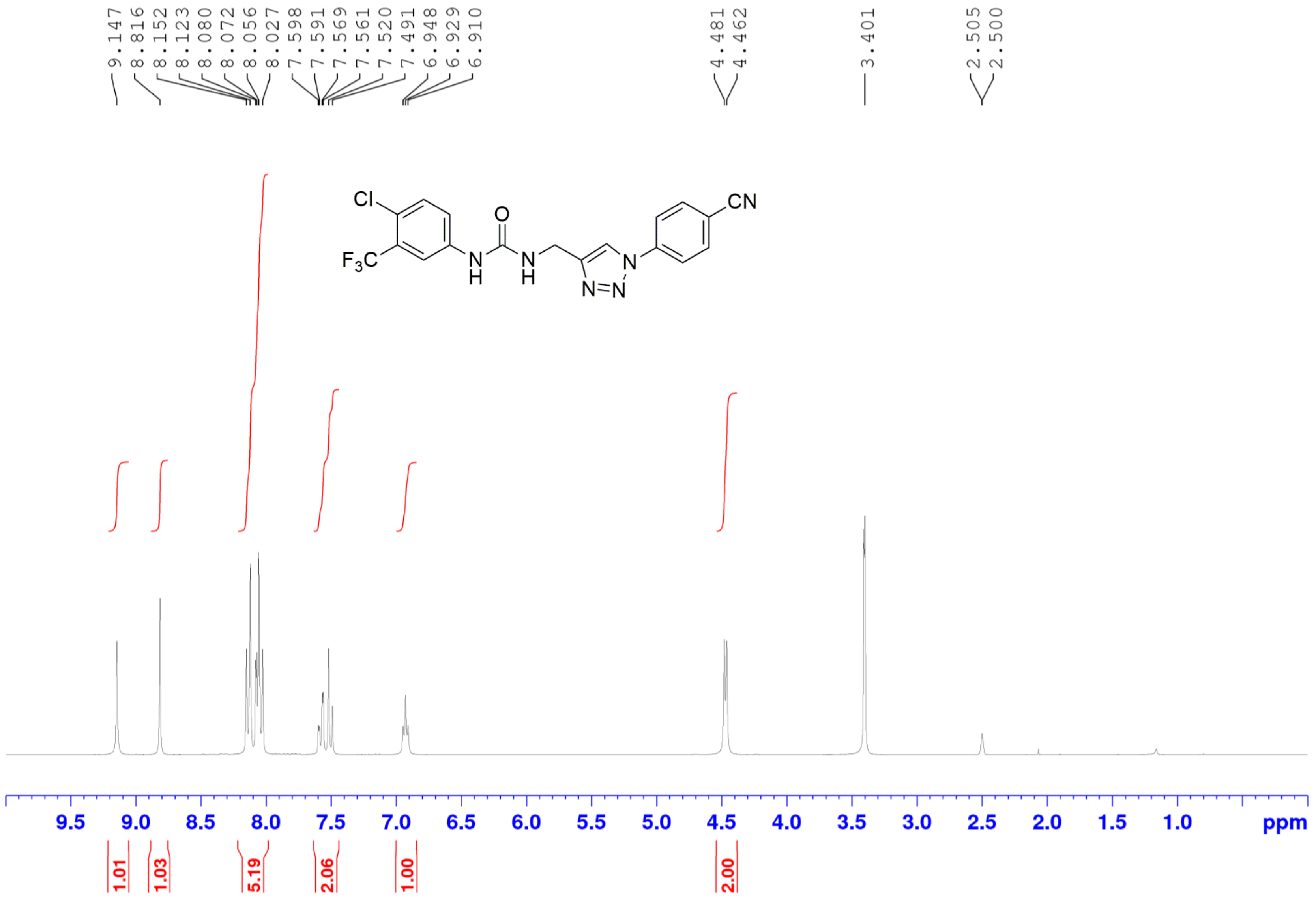

^{13}C NMR of compound **2b'** (75 MHz, DMSO-d_6)

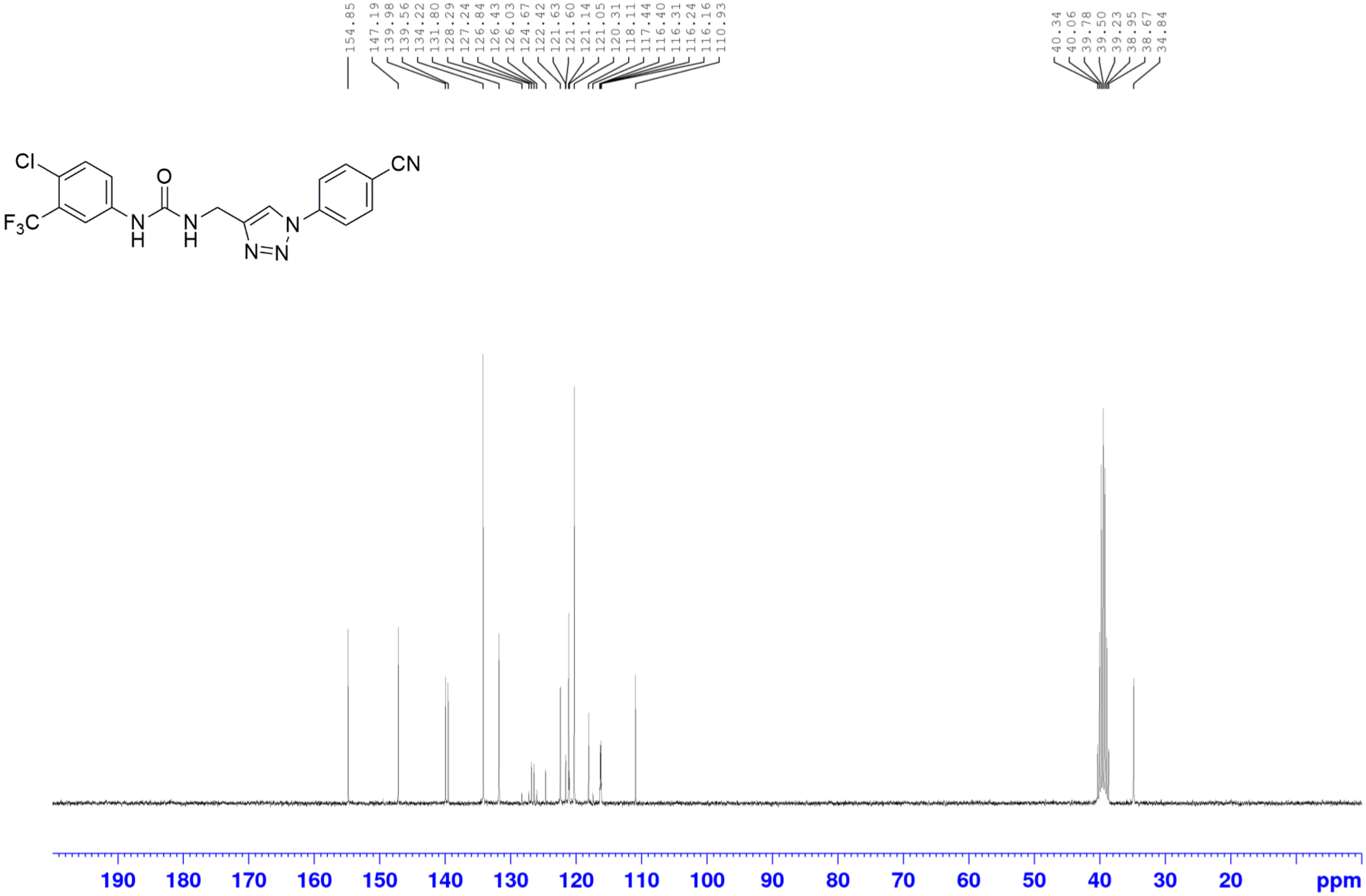

^{19}F NMR of compound **2b'** (282 MHz, DMSO-d_6)

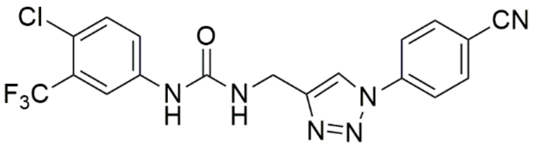

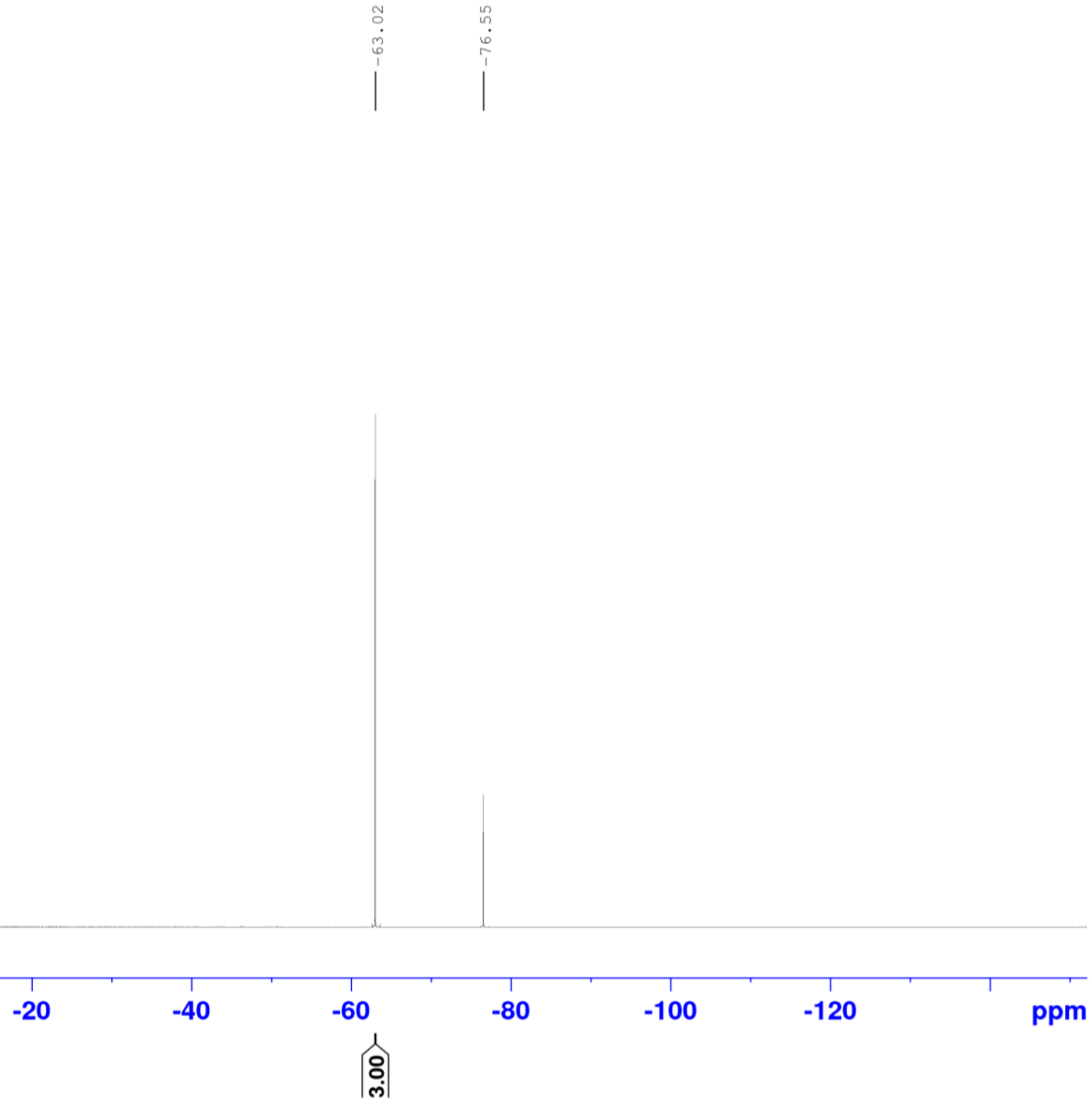

^{1}H NMR of compound **2c'** (300 MHz, DMSO-d_6)

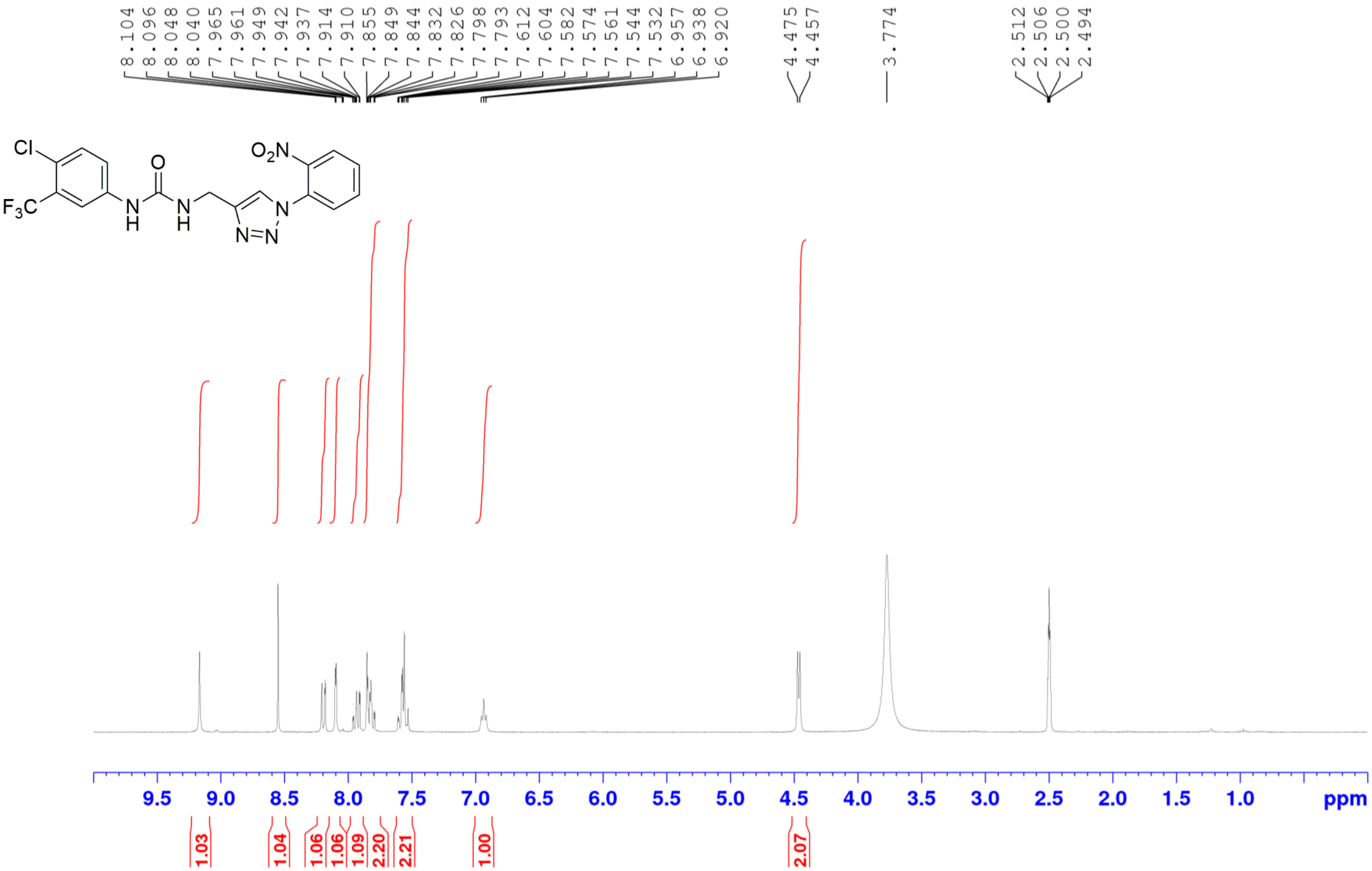

^{13}C NMR of compound **2c'** (75 MHz, DMSO-d_6)

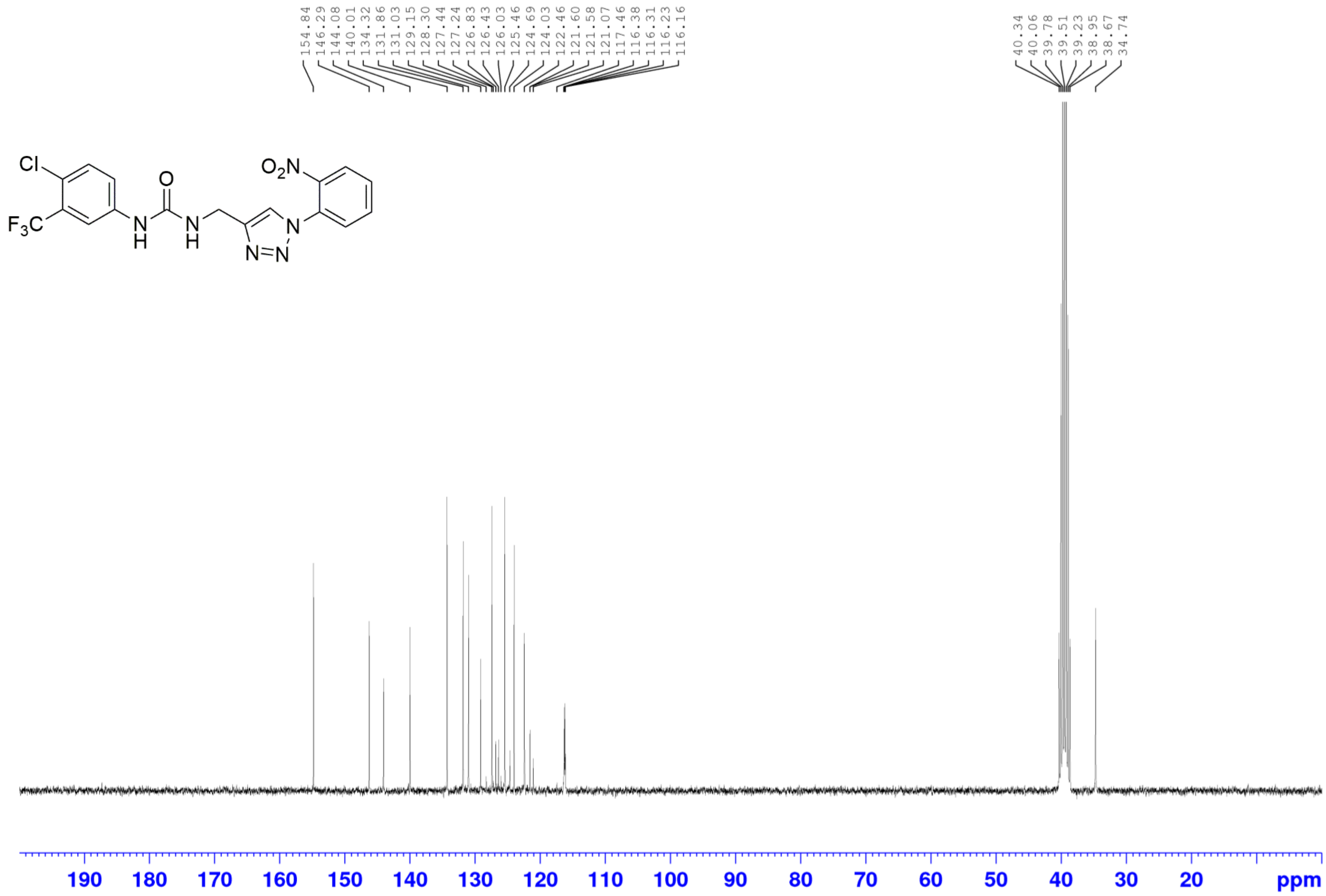

^{19}F NMR of compound **2c’** (282 MHz, DMSO-d_6)

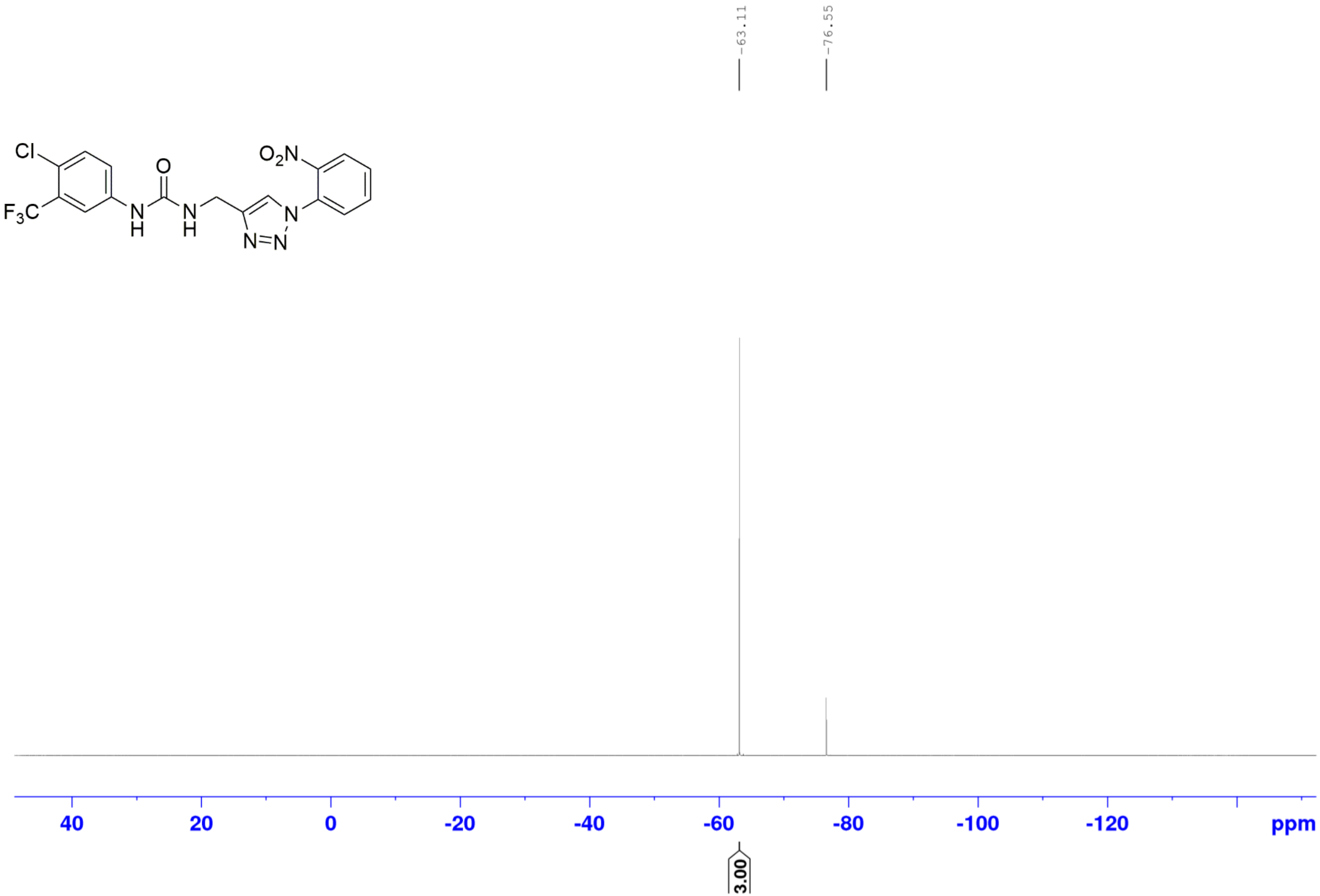

^{1}H NMR of compound **2d'** (300 MHz, DMSO-d_6)

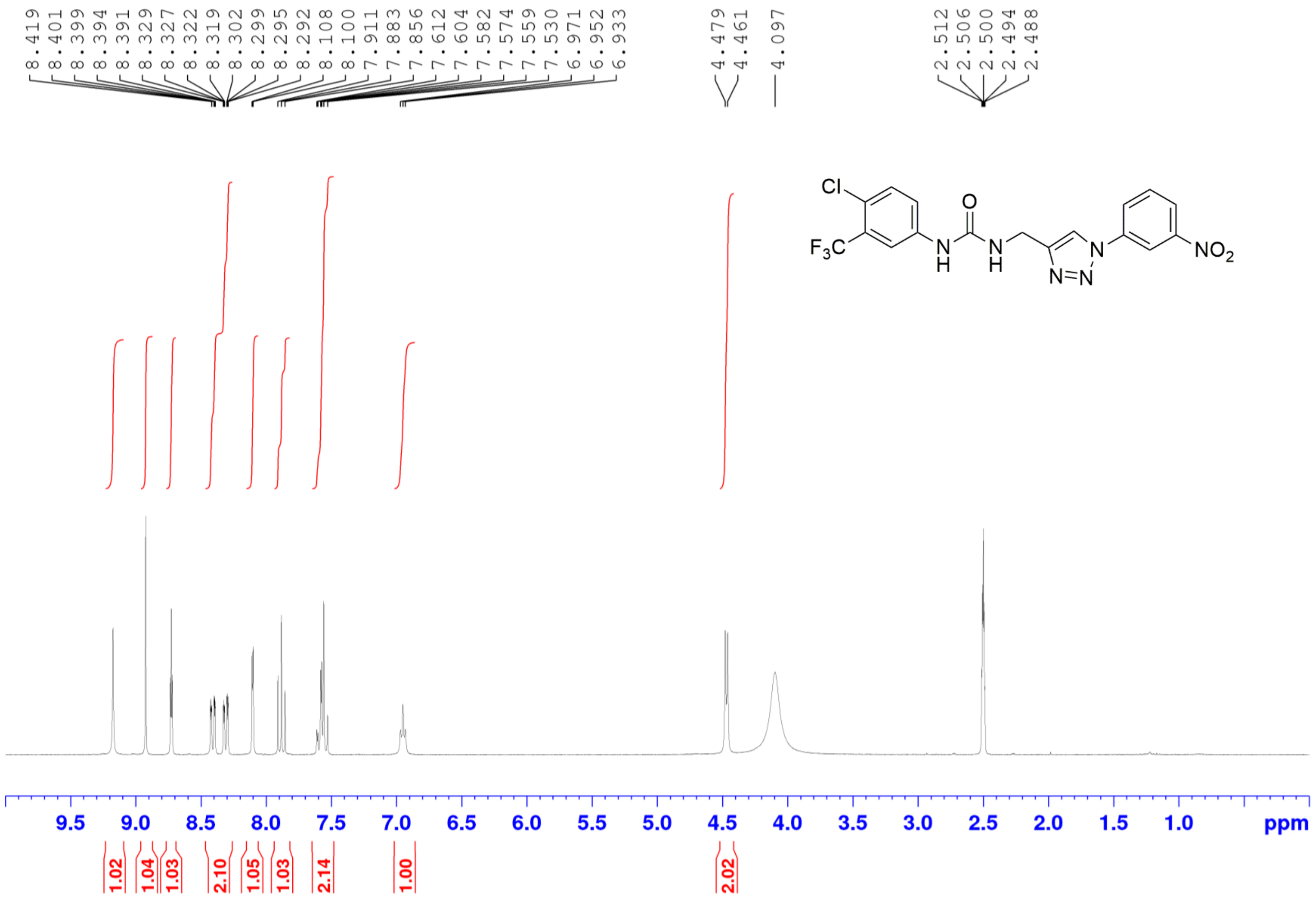

^{13}C NMR of compound **2d’** (75 MHz, DMSO-*d6*)

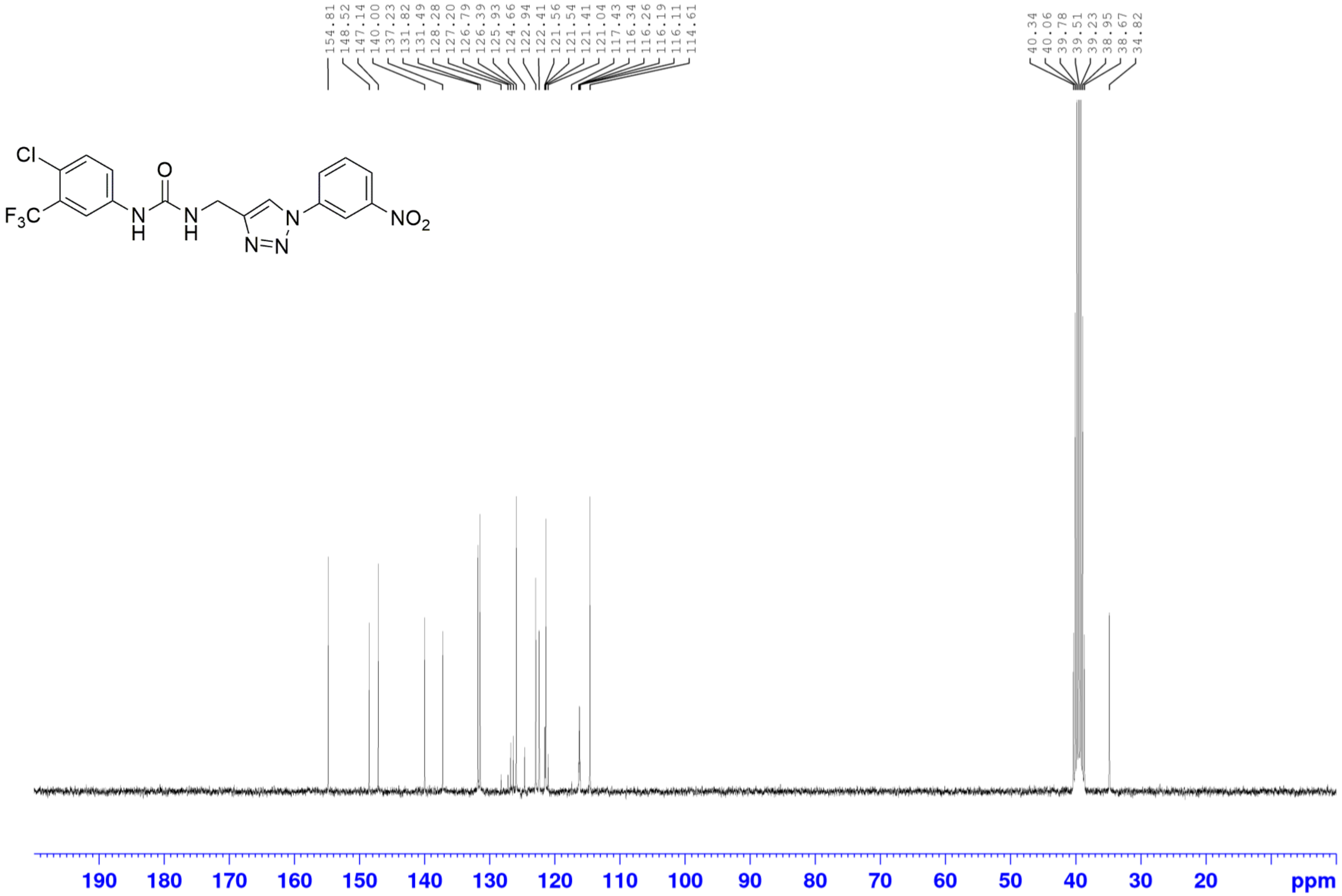

^{19}F NMR of compound **2d’** (282 MHz, DMSO-*d6*)

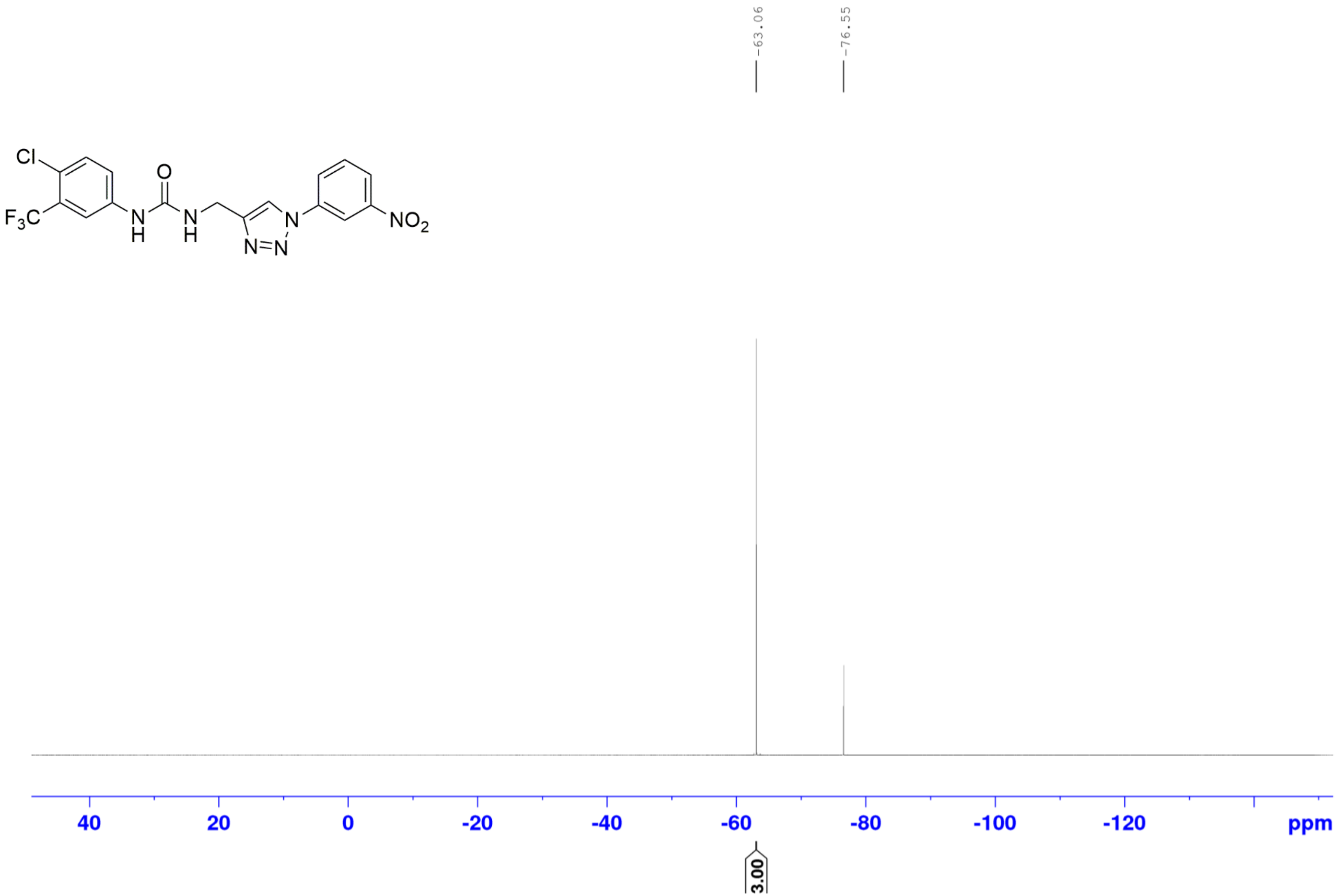

^{1}H NMR of compound **2e’** (300 MHz, DMSO-d_6)

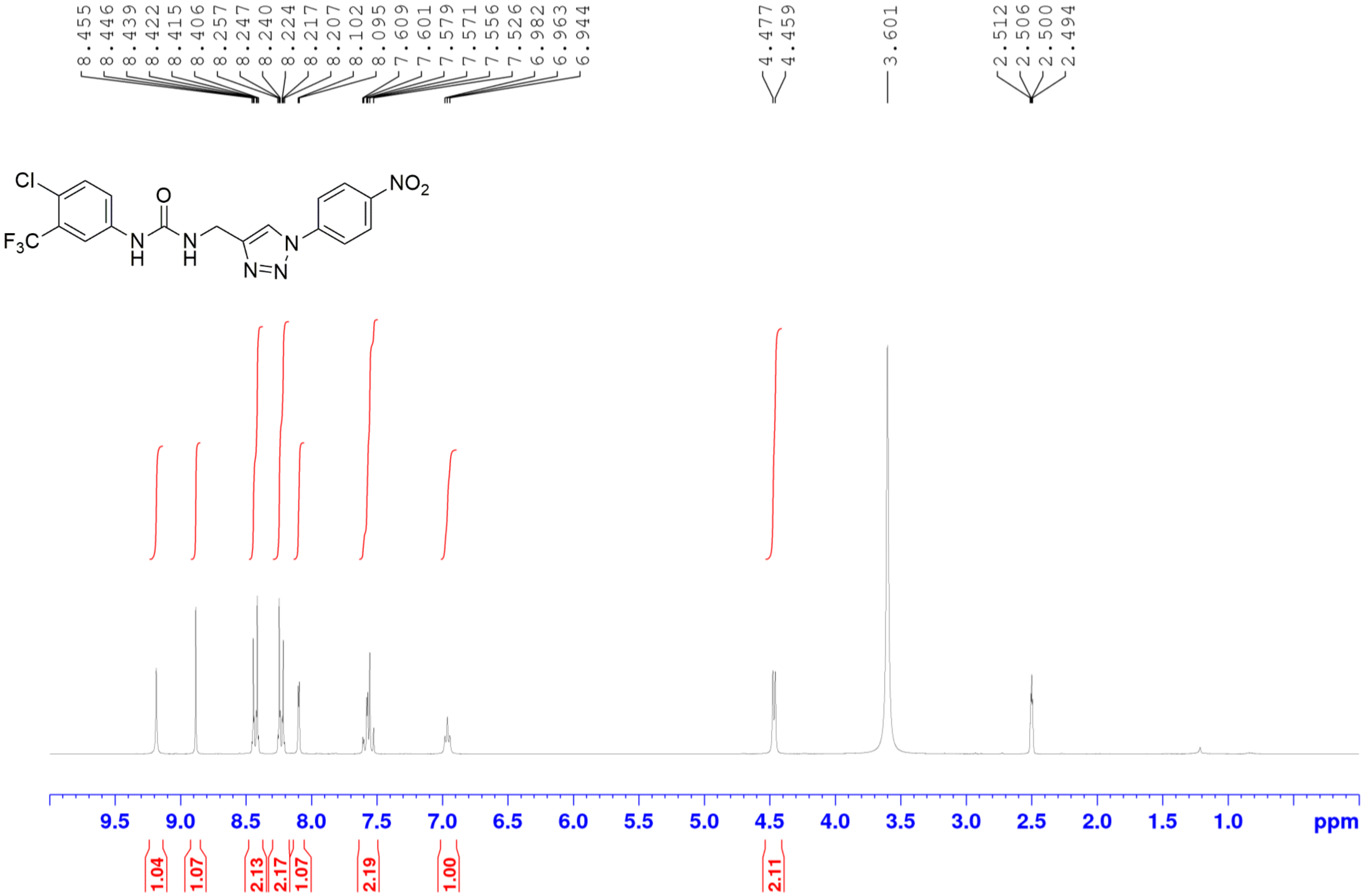

^{13}C NMR of compound **2e'** (75 MHz, DMSO-d_6)

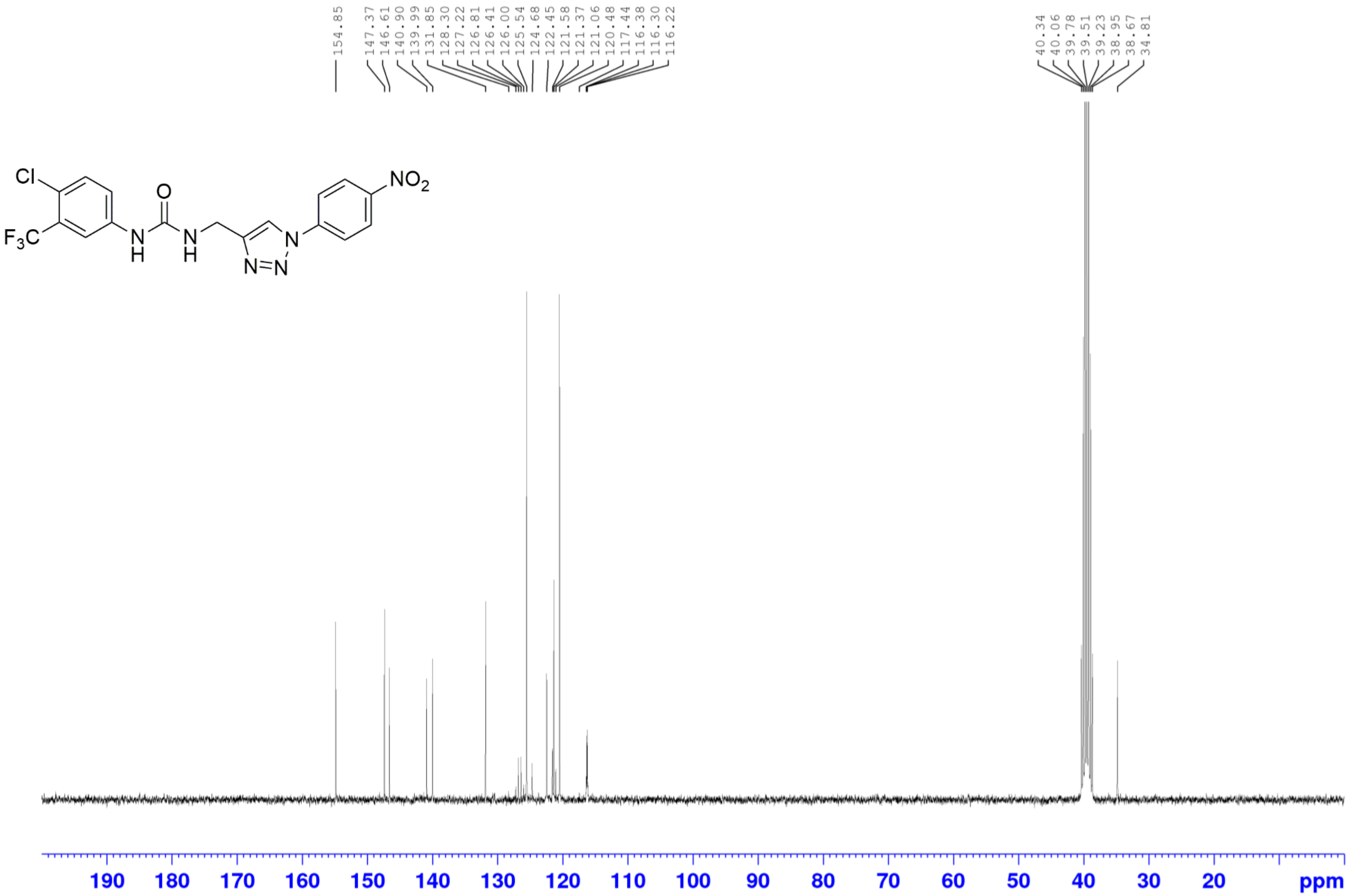

^{19}F NMR of compound **2e'** (282 MHz, DMSO-*d*$_6$)

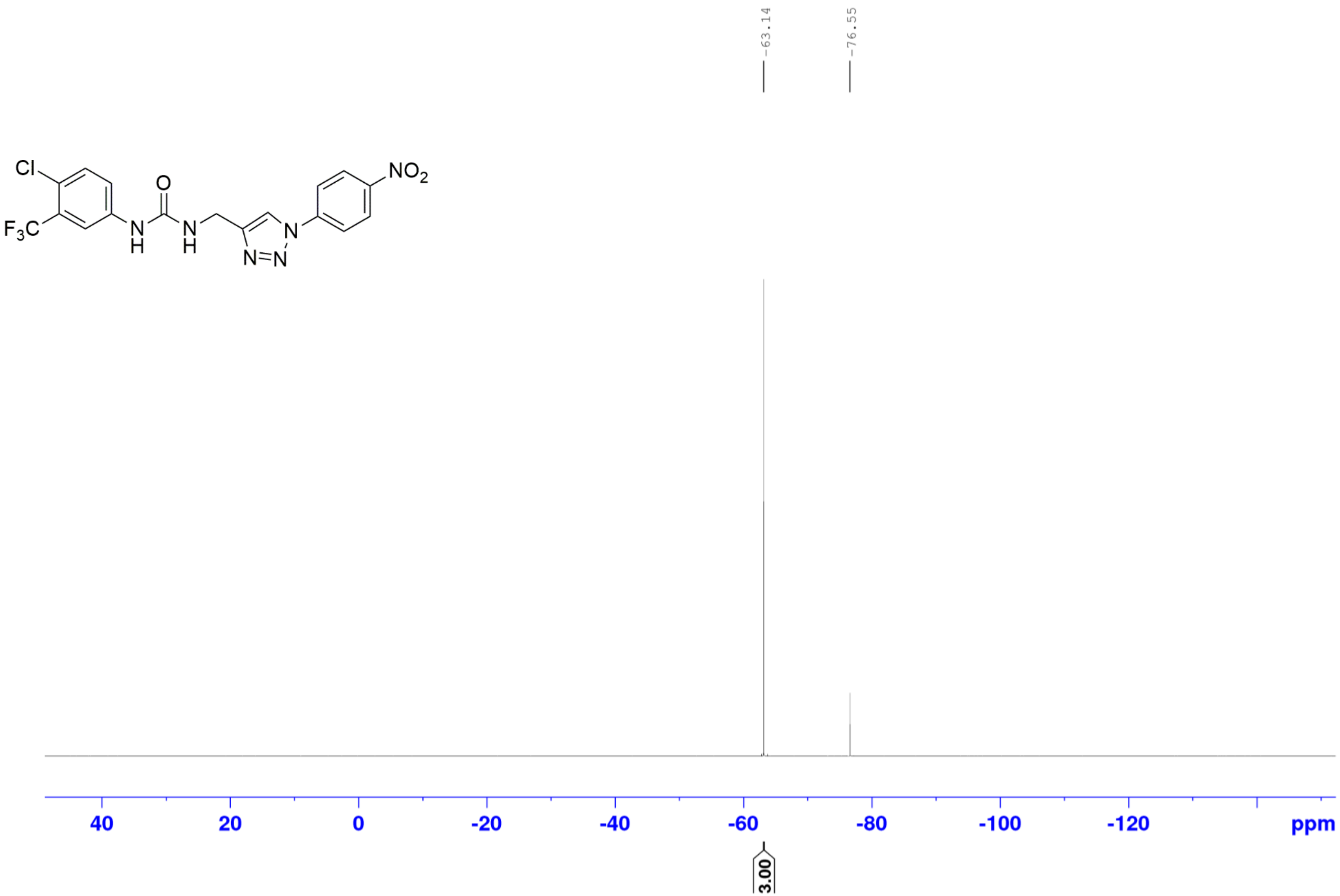

^{1}H NMR of compound **2f’** (300 MHz, DMSO-d_6)

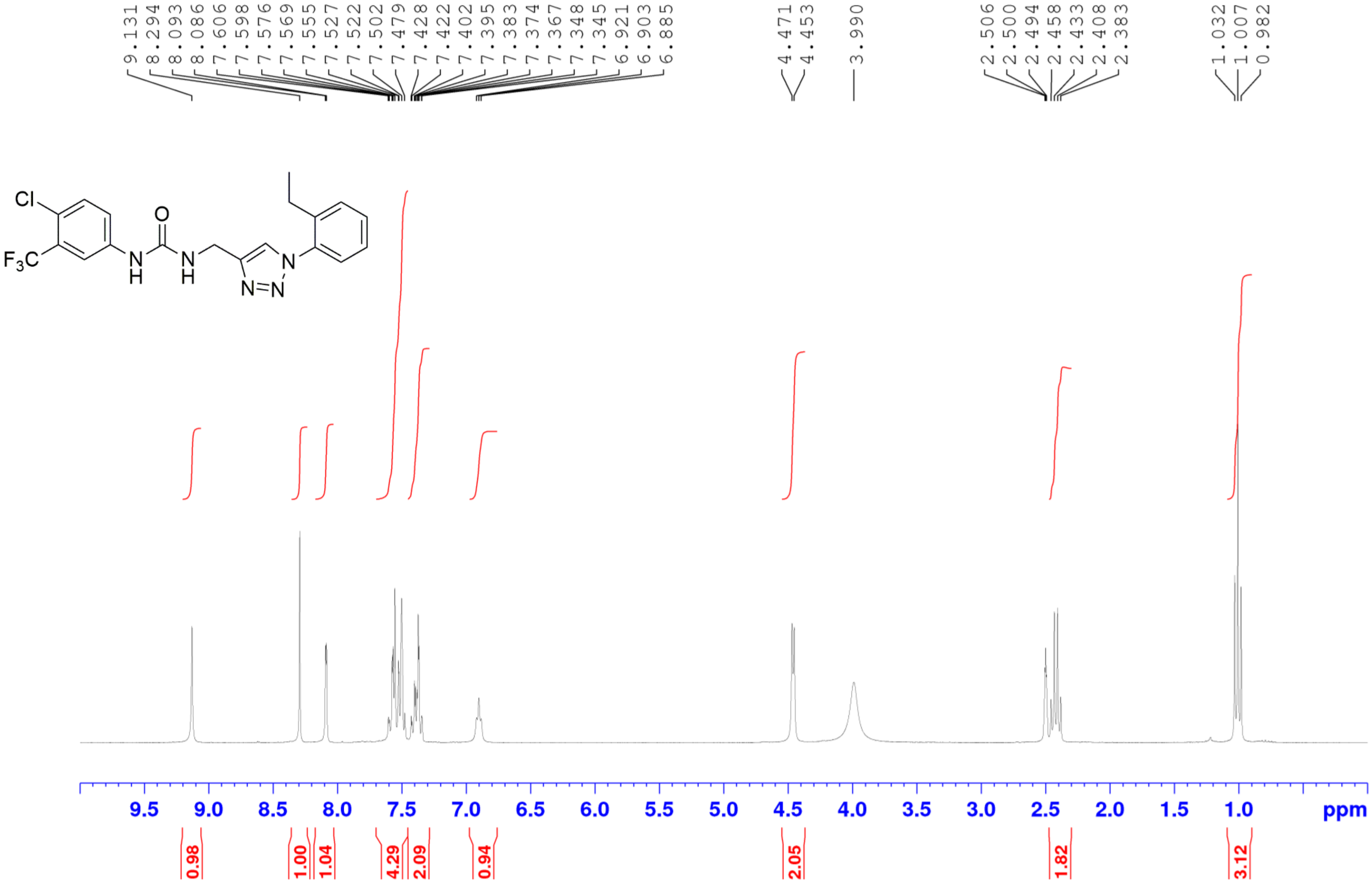

^{13}C NMR of compound **2f'** (75 MHz, DMSO-d_6)

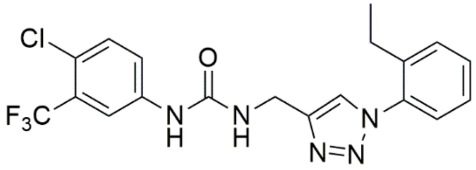

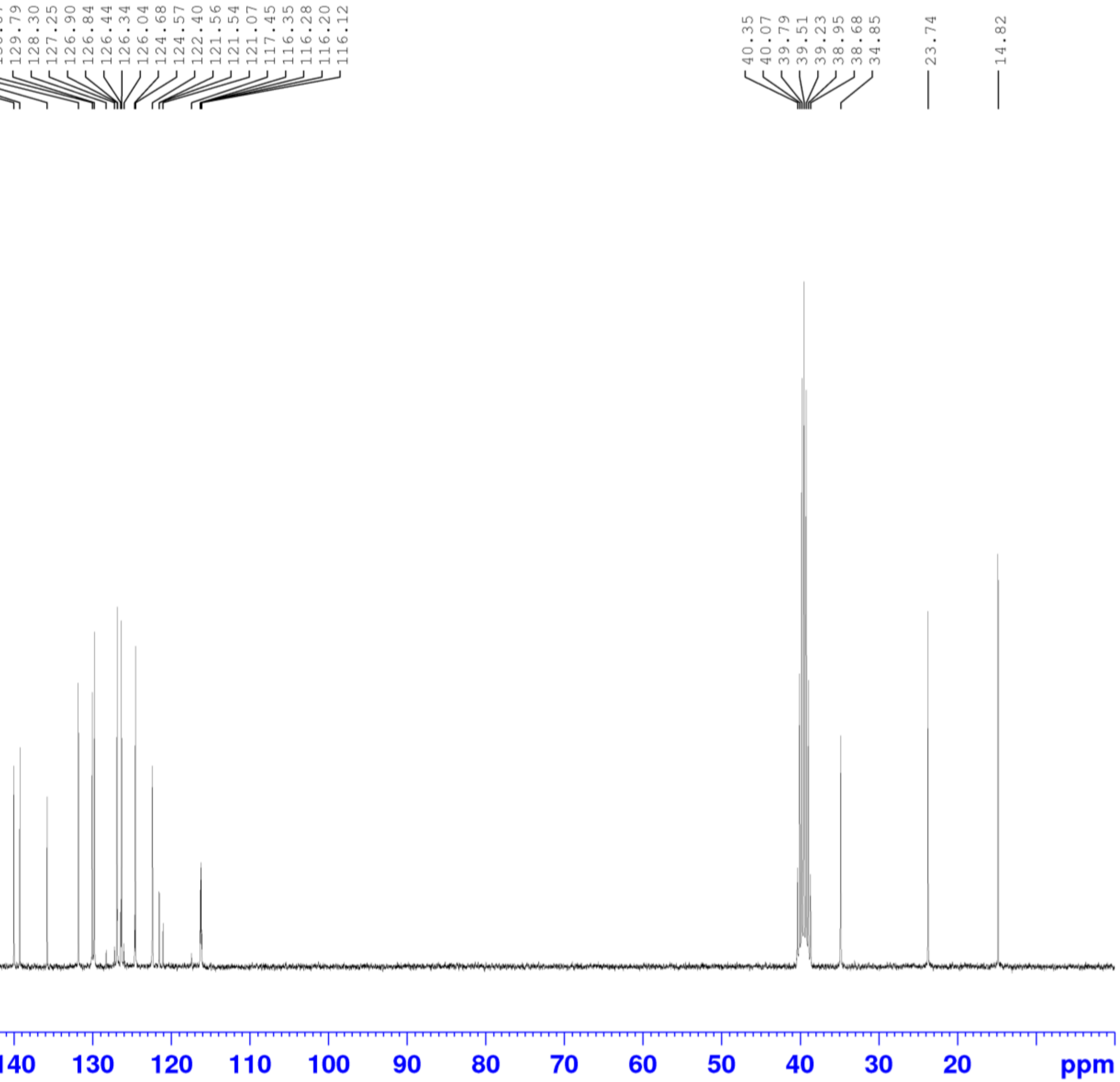

^{19}F NMR of compound **2f'** (282 MHz, DMSO-d_6)

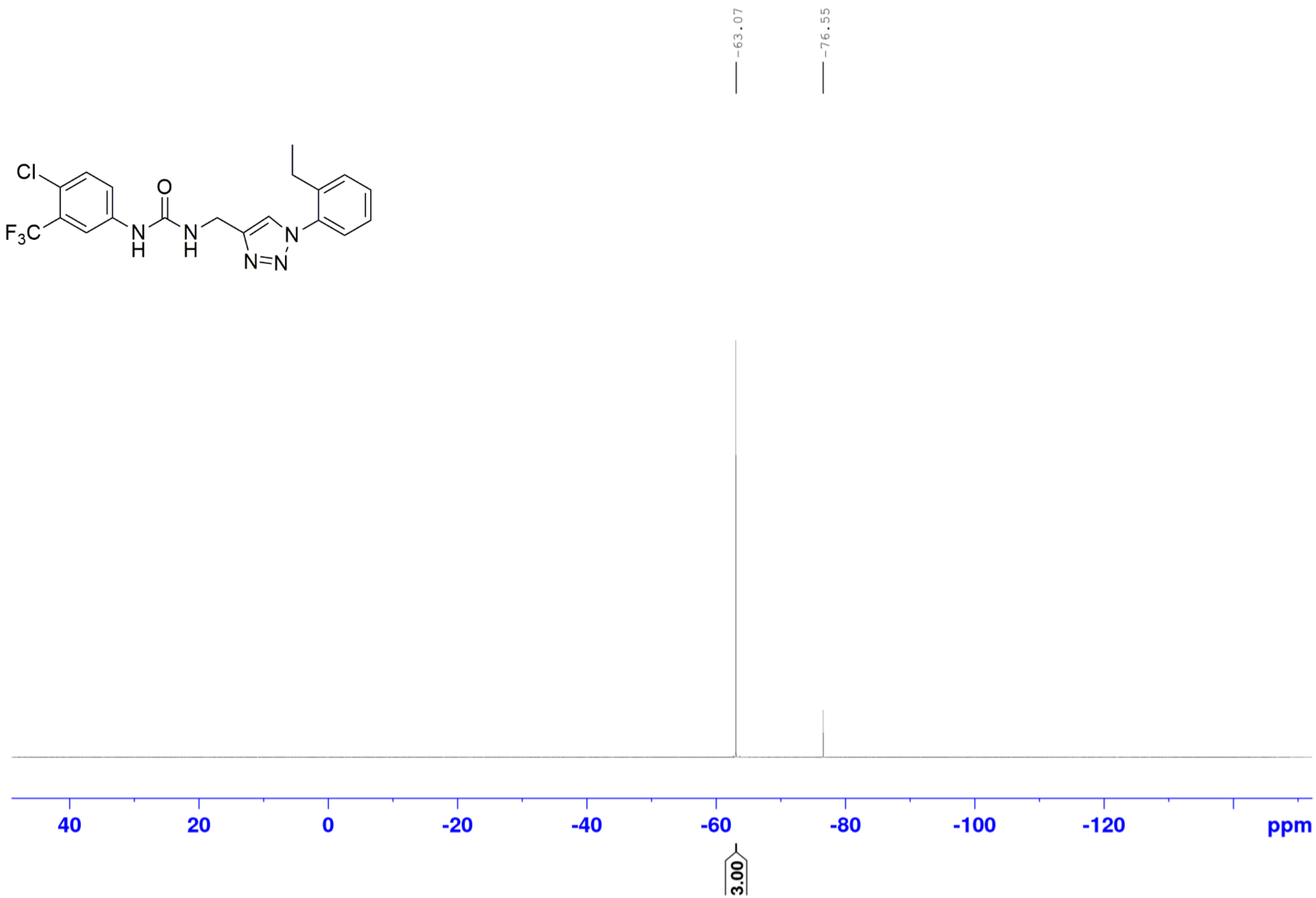

^{1}H NMR of compound **2g'** (300 MHz, DMSO-d_6)

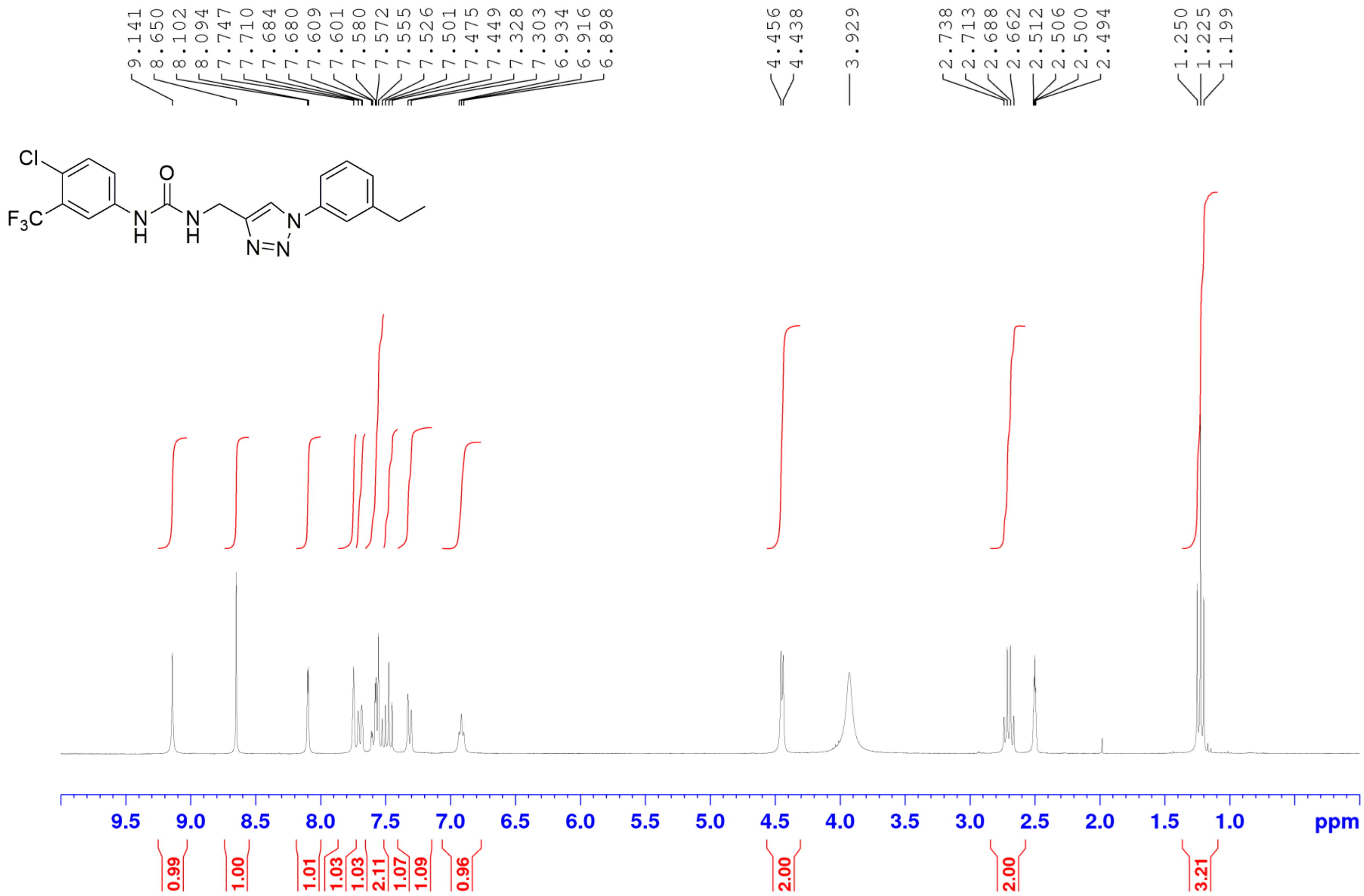

^{13}C NMR of compound **2g'** (75 MHz, DMSO-*d*$_6$)

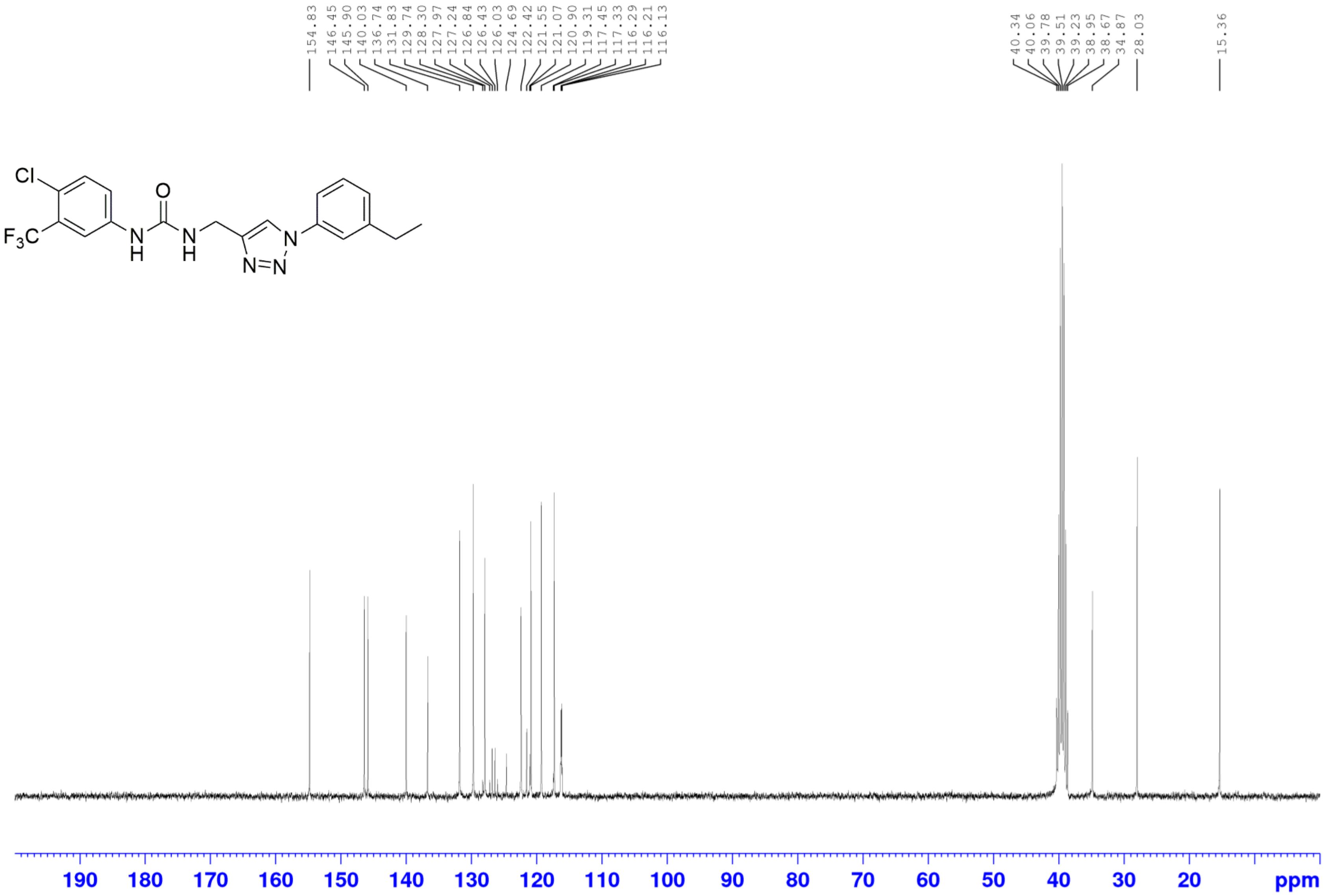

^{19}F NMR of compound **2g’** (282 MHz, DMSO-d_6)

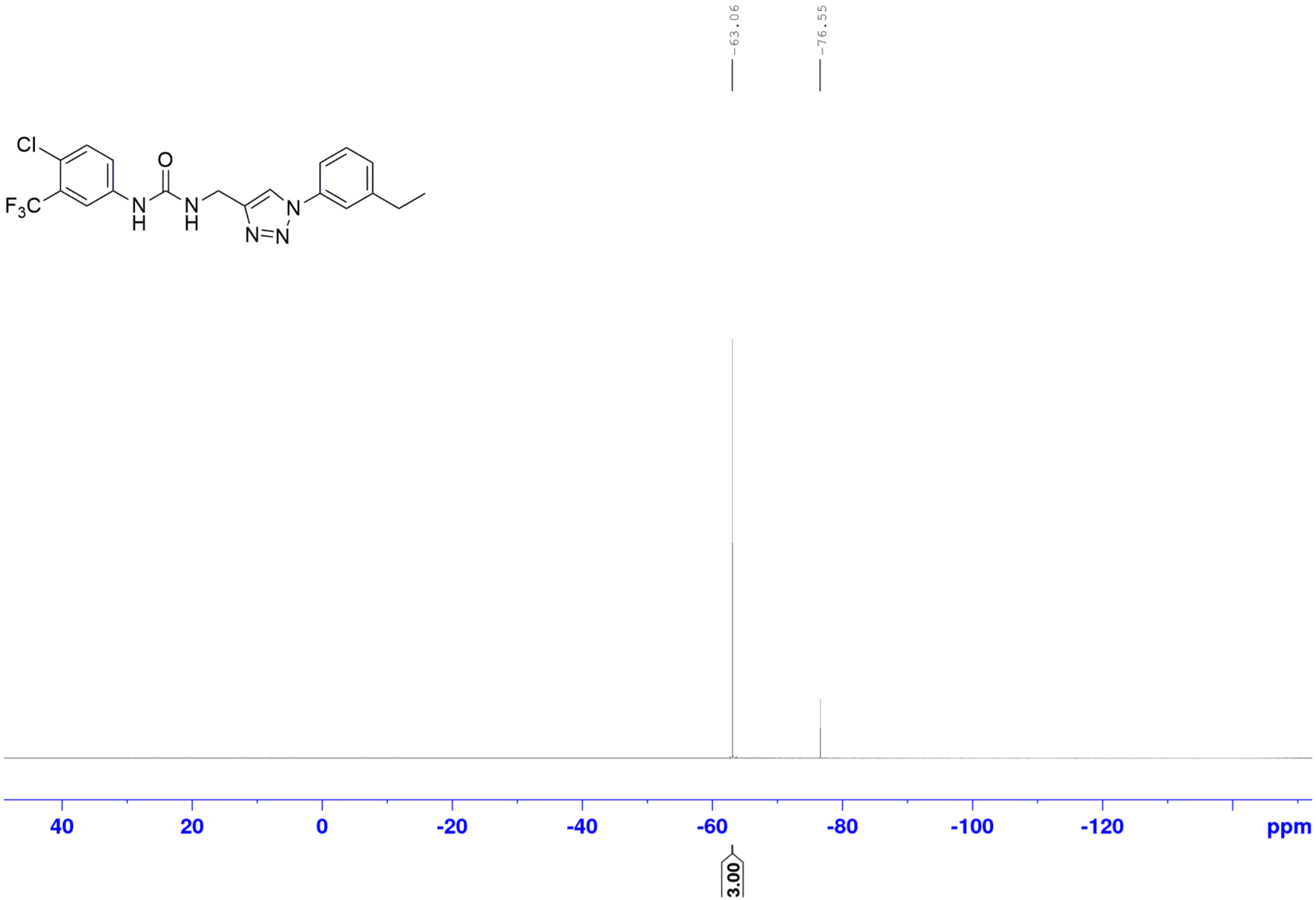

^{1}H NMR of compound **2h’** (300 MHz, DMSO-d_6)

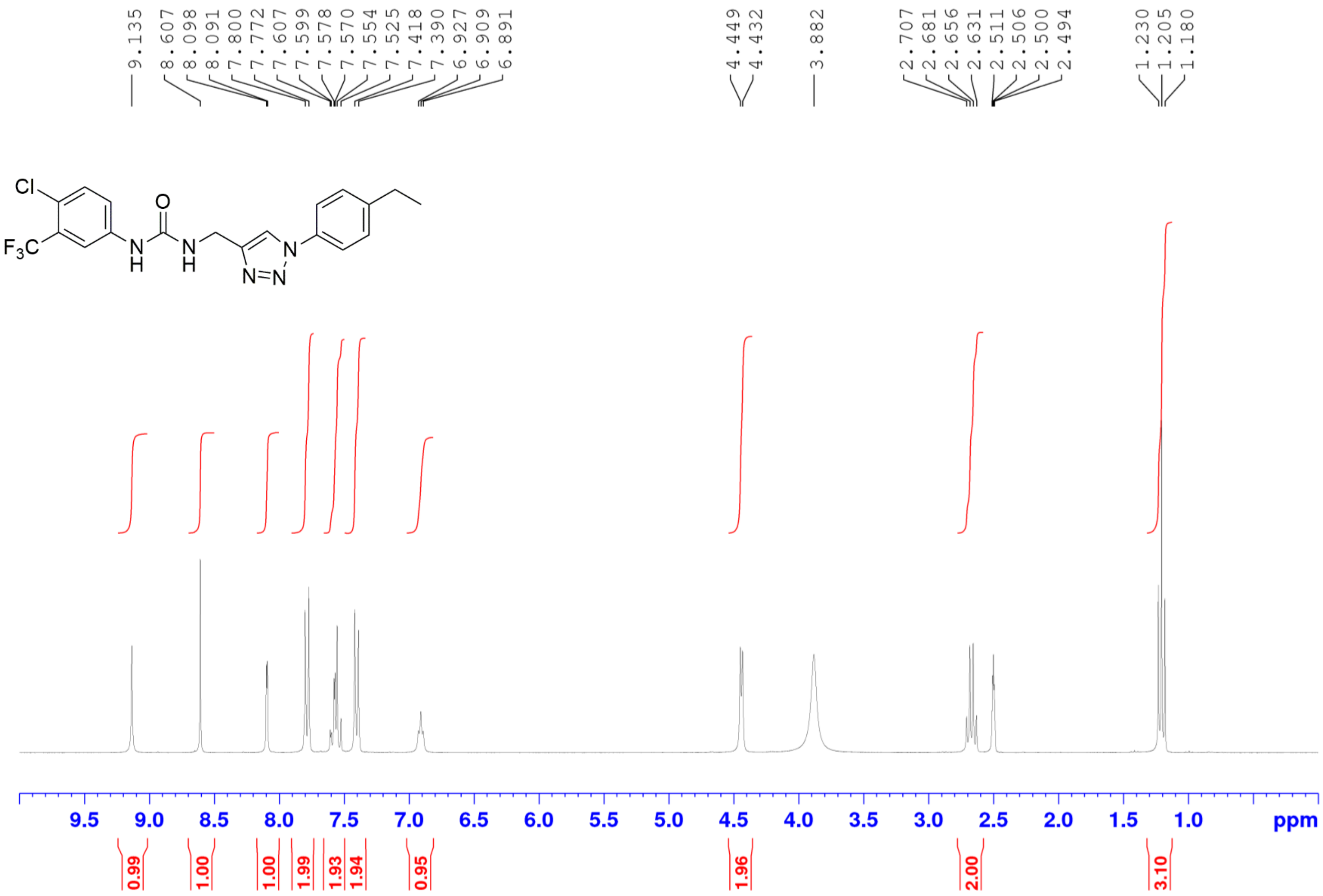

^{13}C NMR of compound **2h'** (75 MHz, DMSO-d_6)

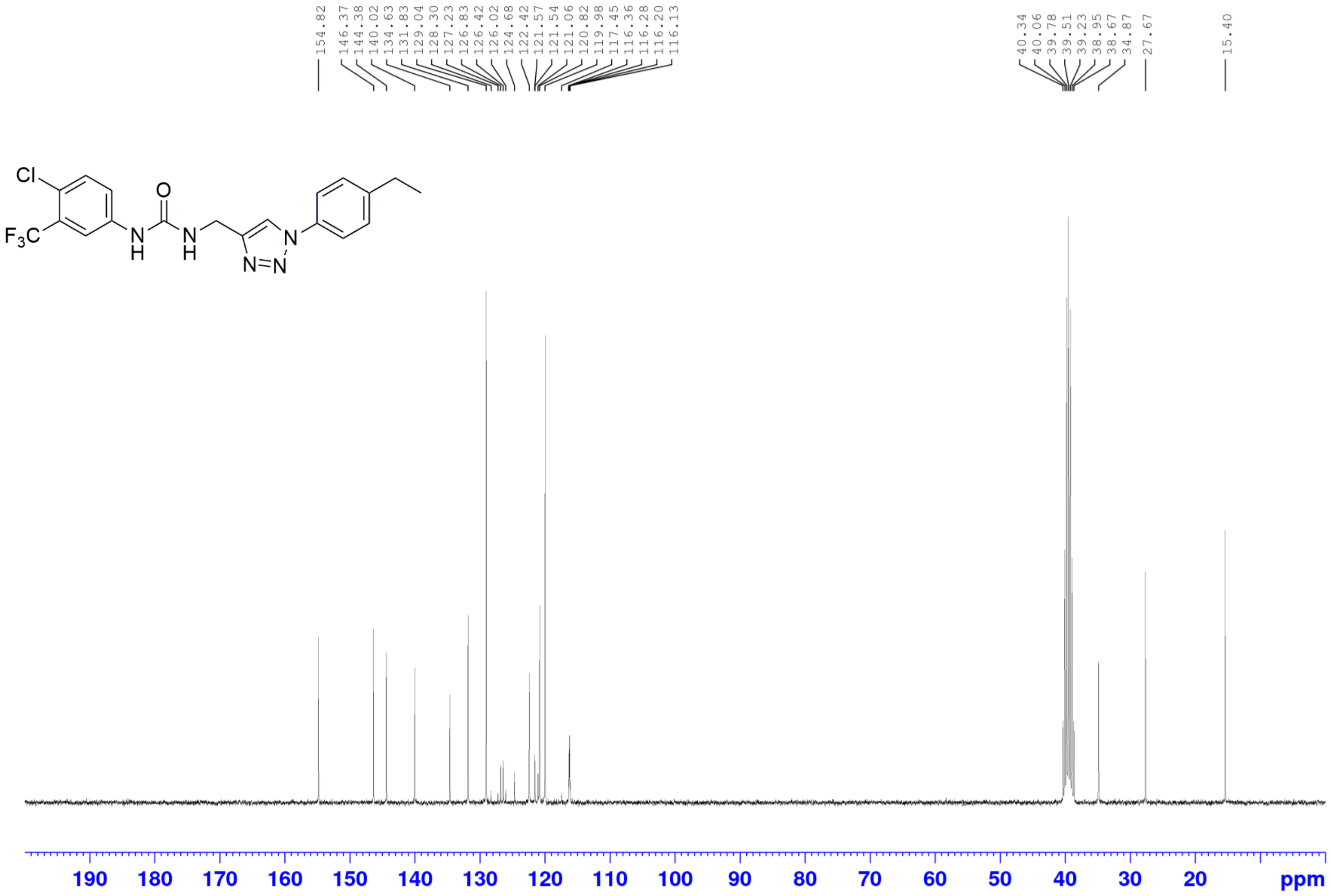

^{19}F NMR of compound **2h'** (282 MHz, DMSO-d_6)

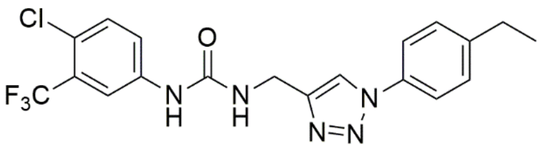

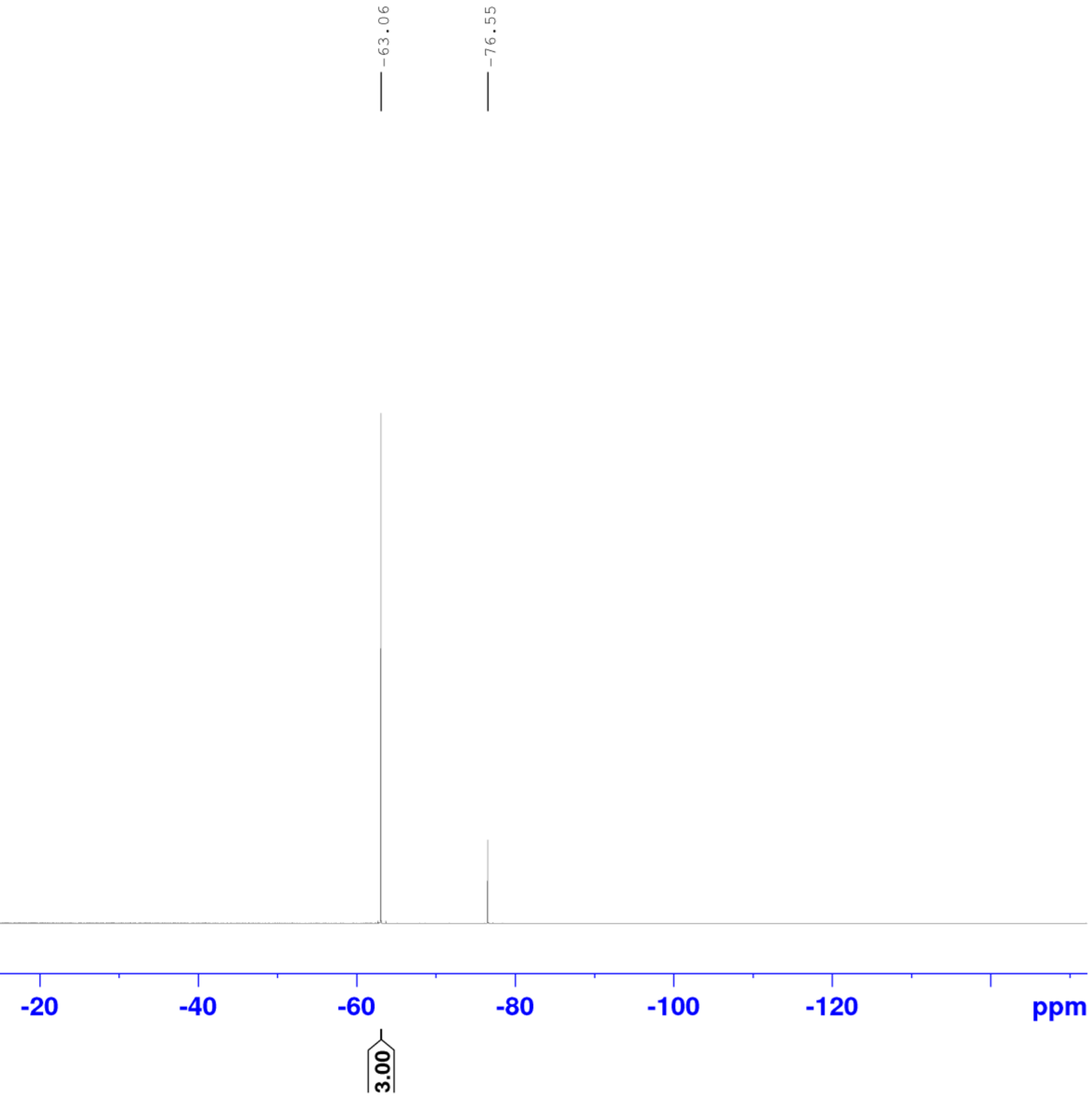

^{1}H NMR of compound **2i'** (300 MHz, DMSO-d_6)

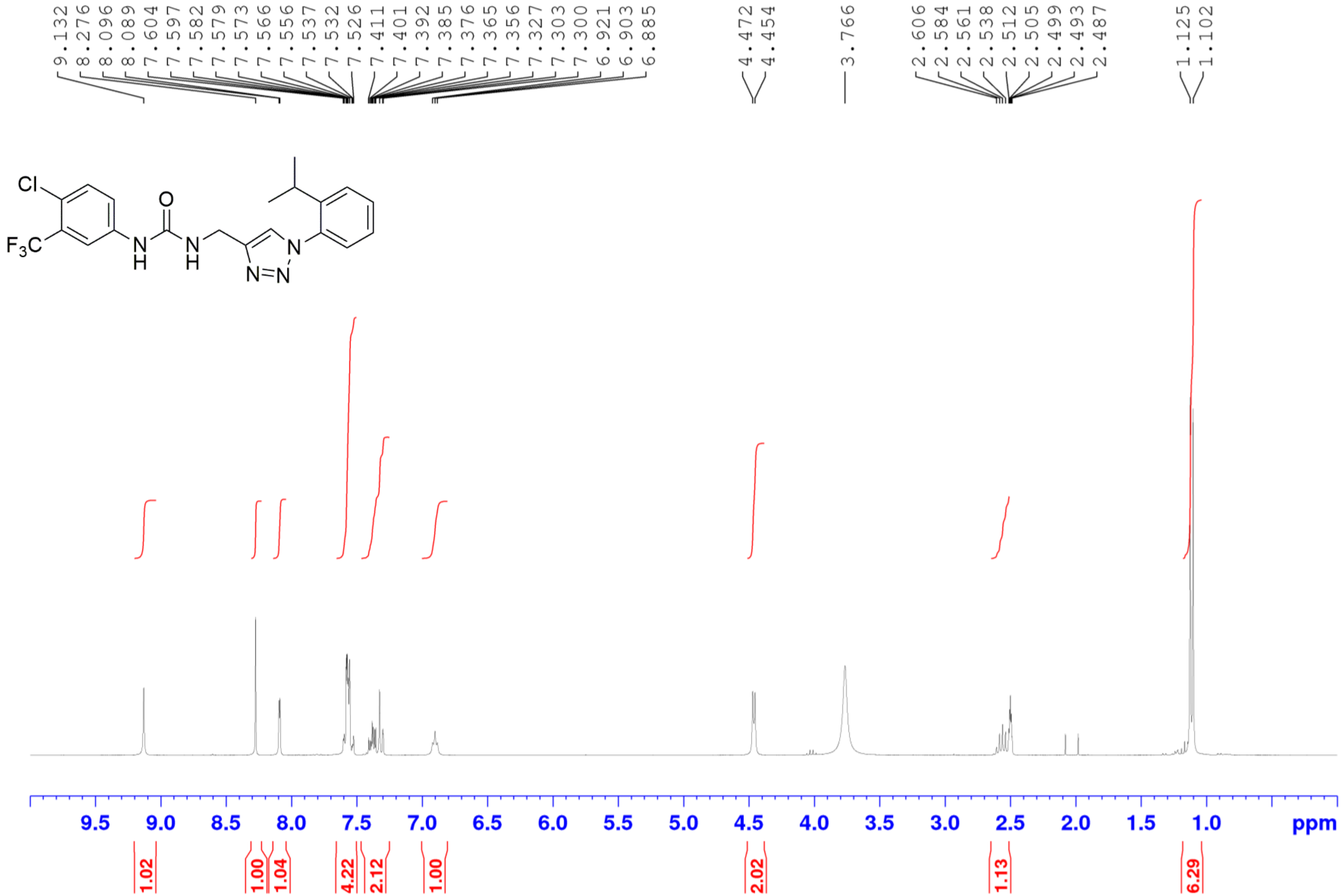

^{13}C NMR of compound **2i’** (75 MHz, DMSO-d_6)

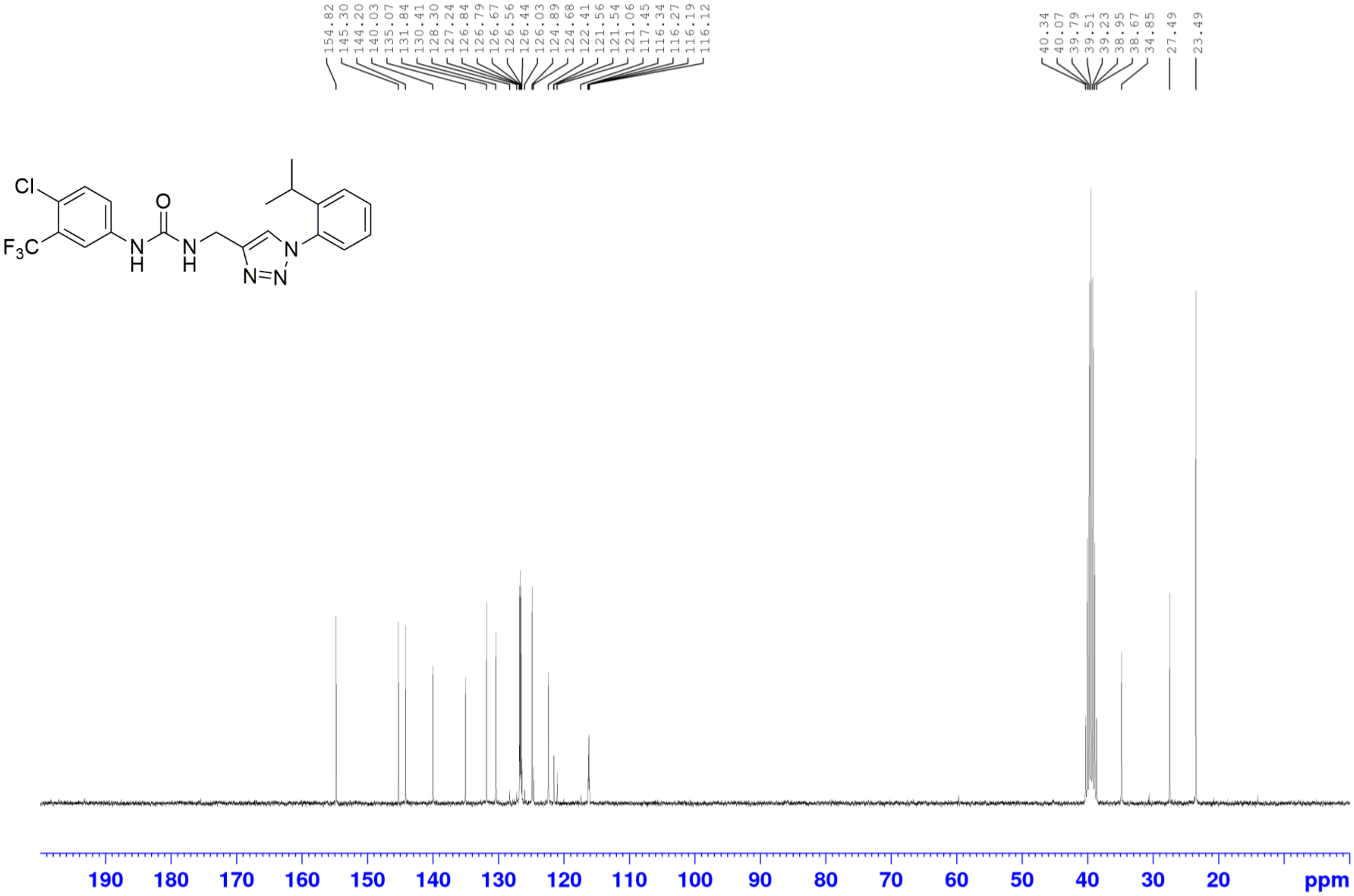

^{19}F NMR of compound **2i’** (282 MHz, DMSO-*d6*)

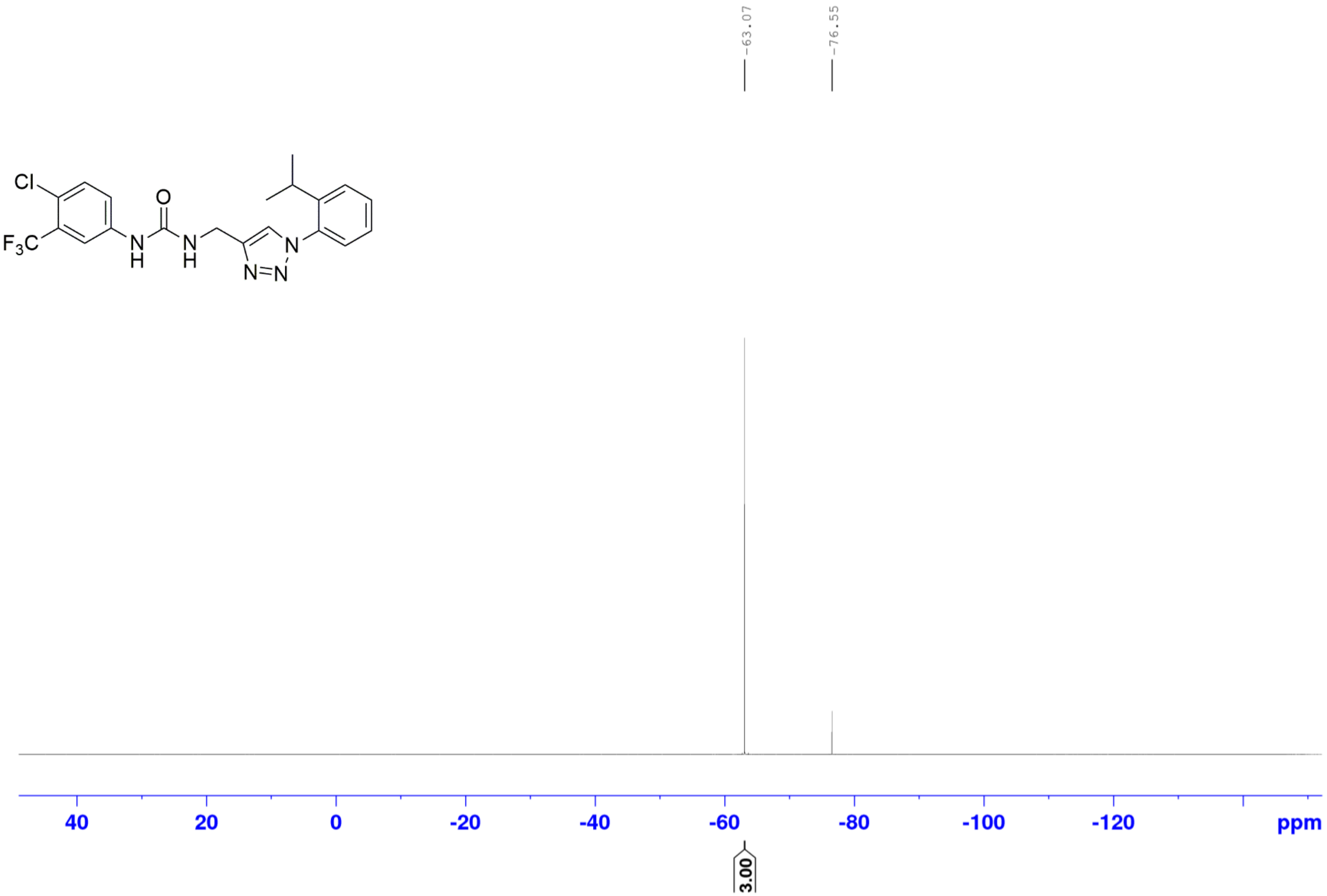

^{1}H NMR of compound **2j'** (300 MHz, DMSO-*d6*)

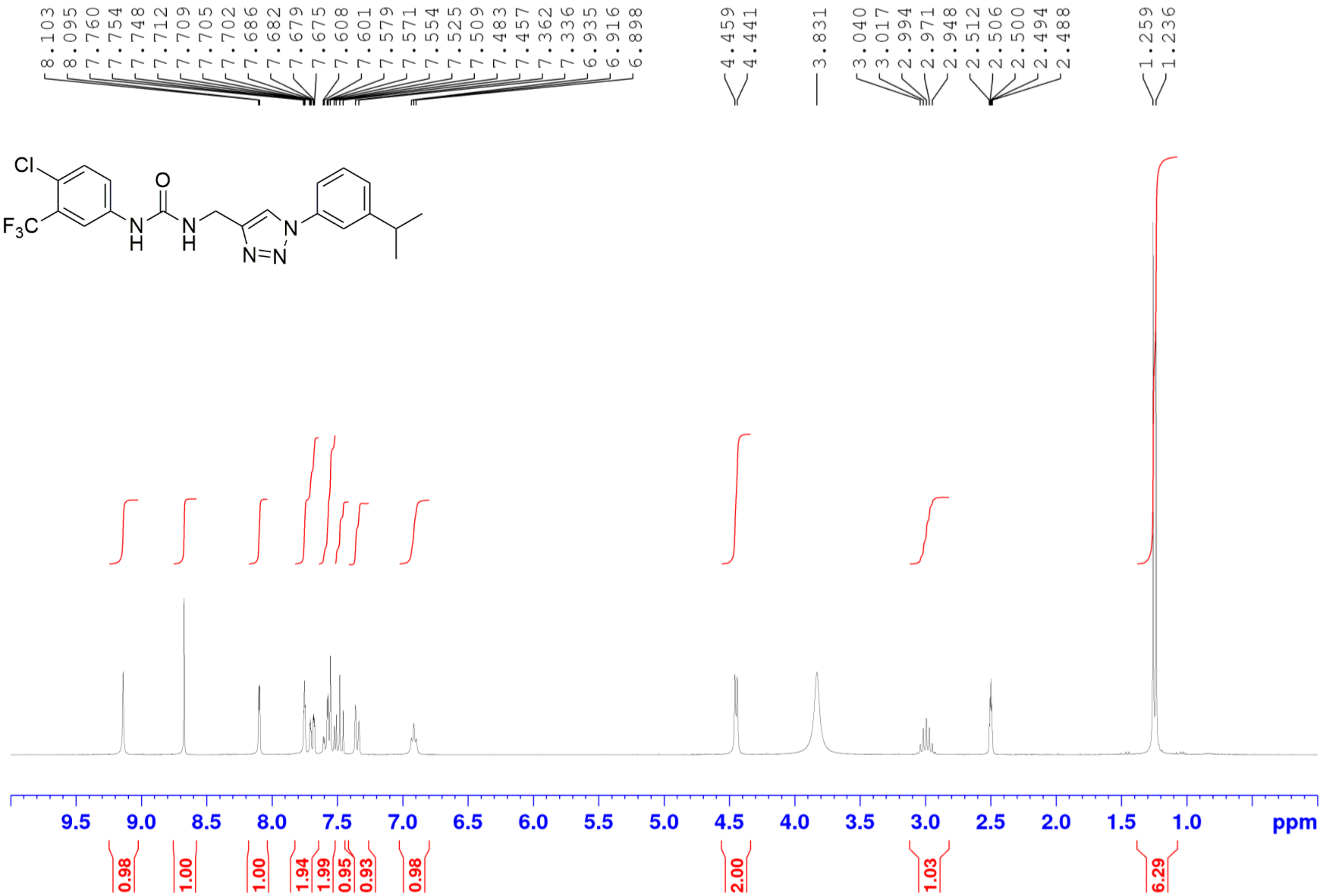

^{13}C NMR of compound **2j'** (75 MHz, DMSO-*d6*)

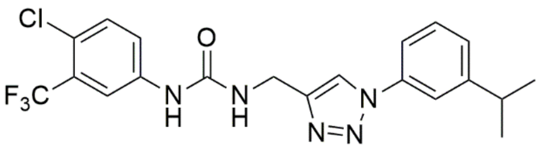

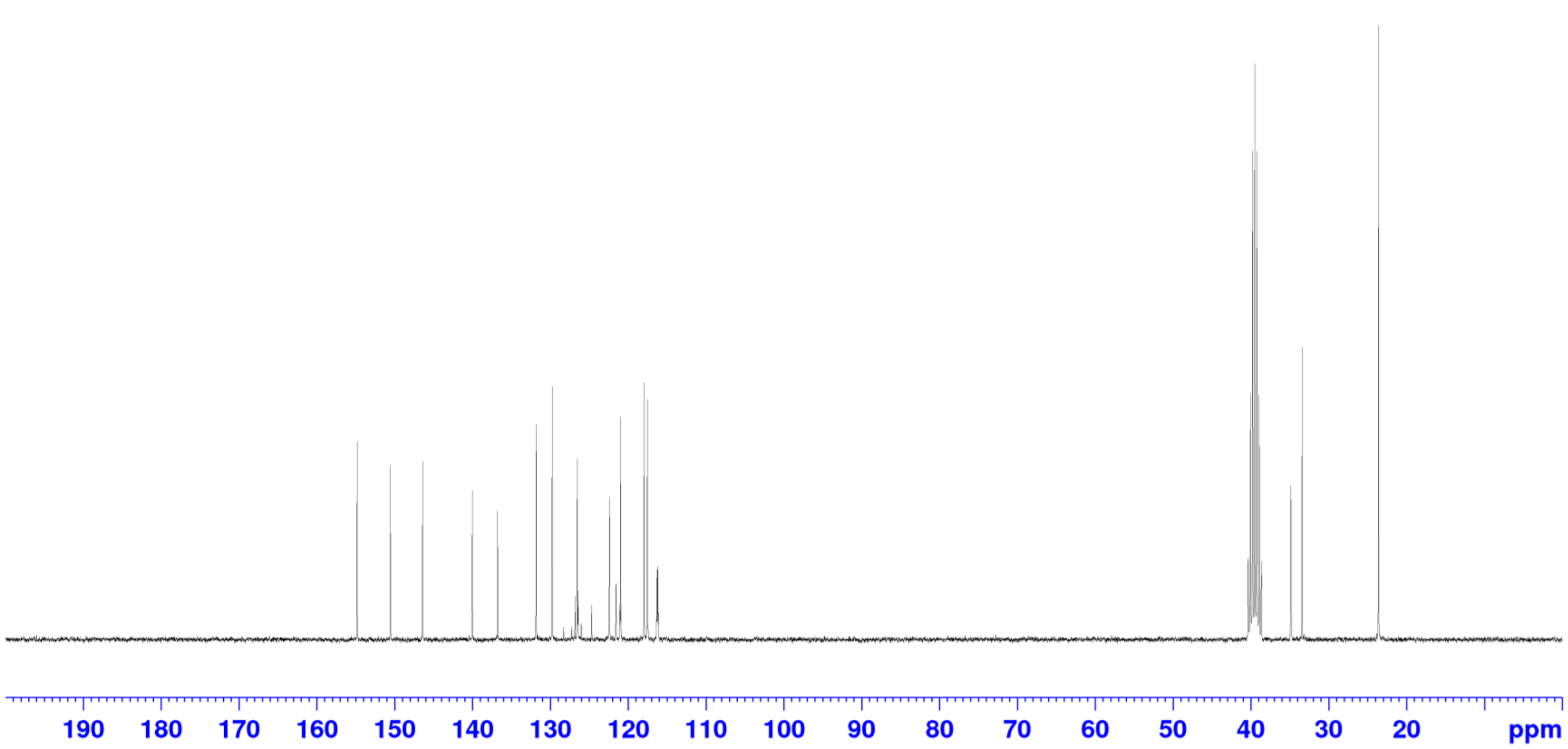

^{19}F NMR of compound **2j’** (282 MHz, DMSO-d_6)

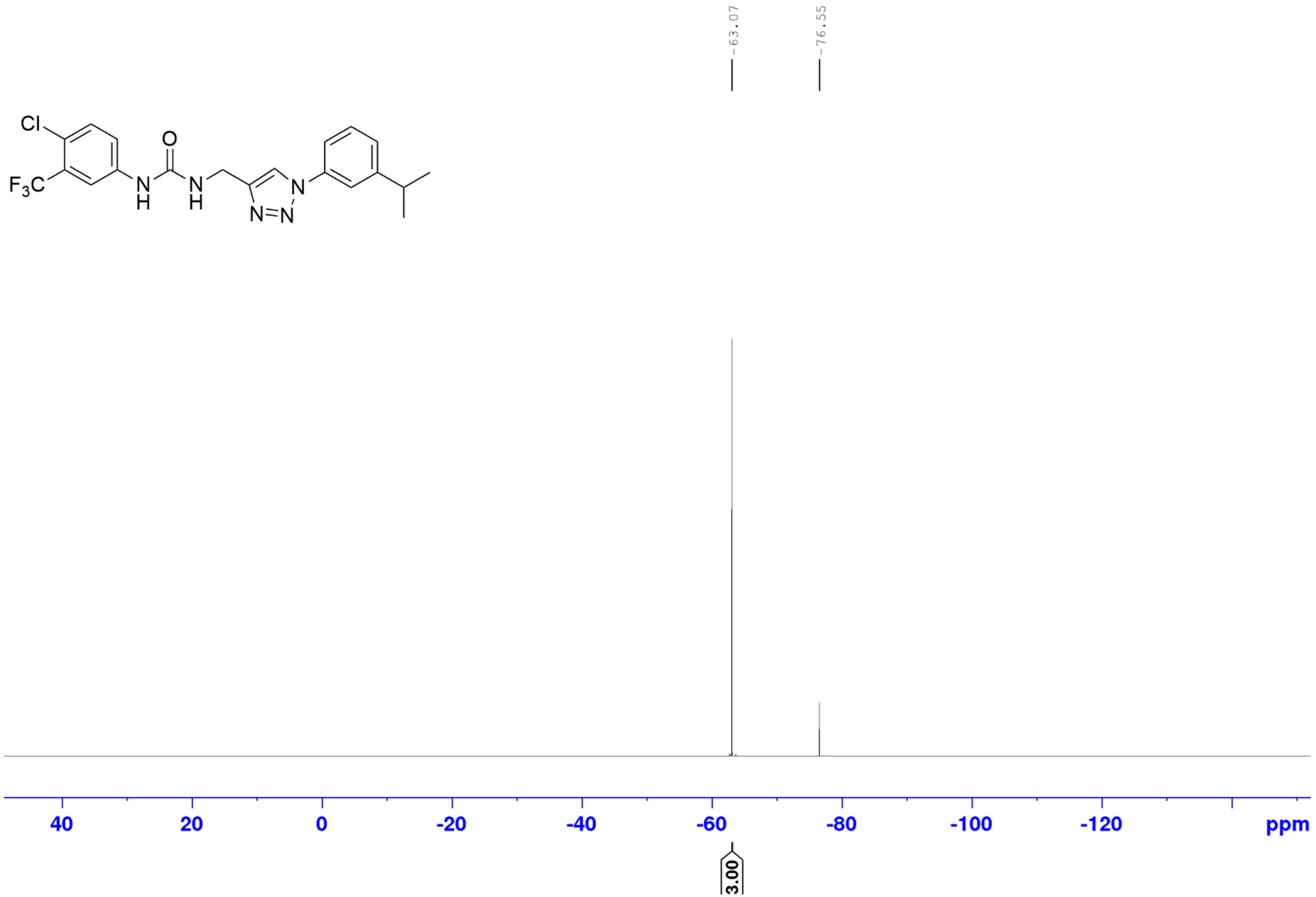

^{1}H NMR of compound **2k'** (300 MHz, DMSO-d_6)

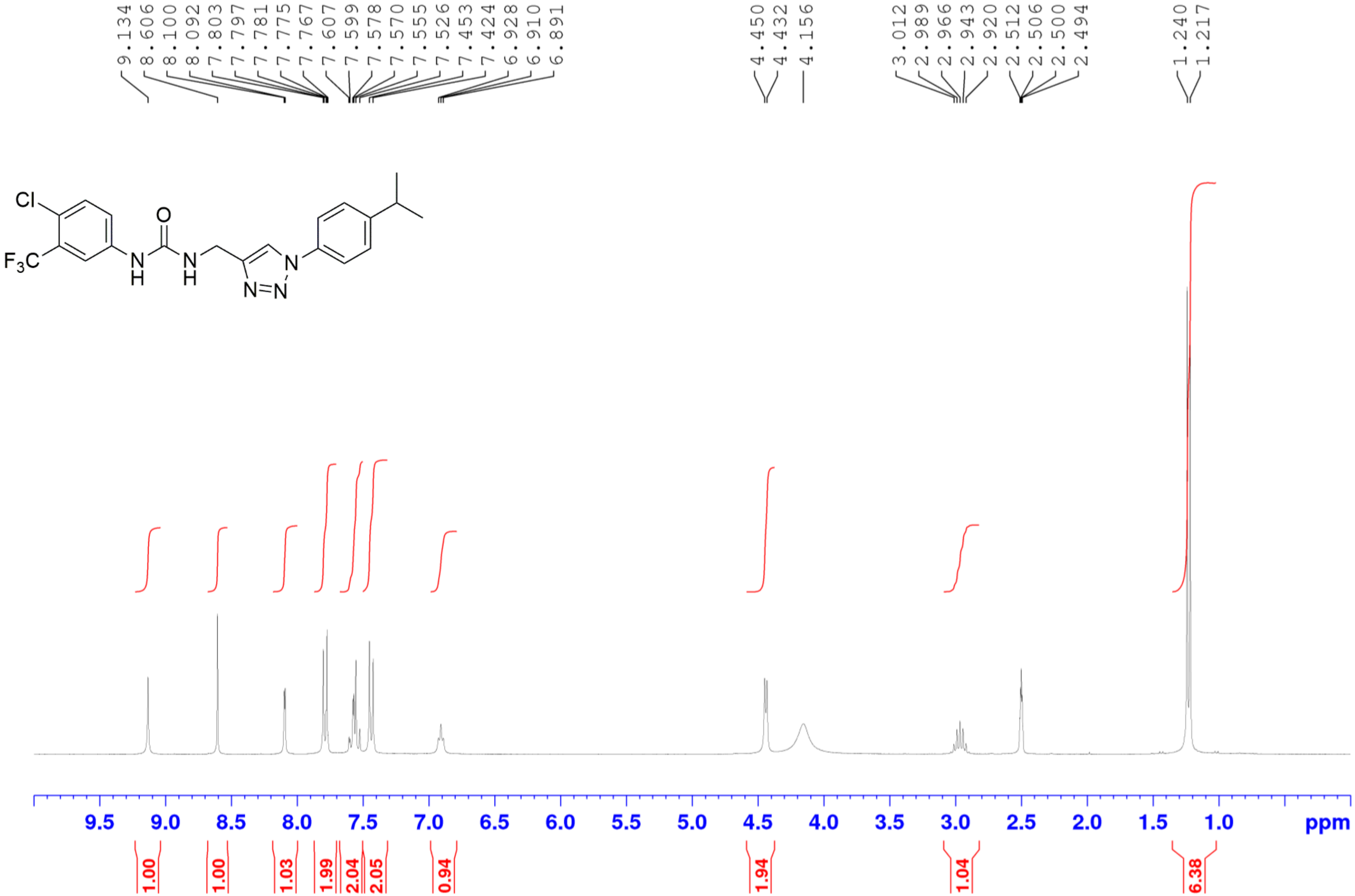

^{13}C NMR of compound **2k'** (75 MHz, DMSO-d_6)

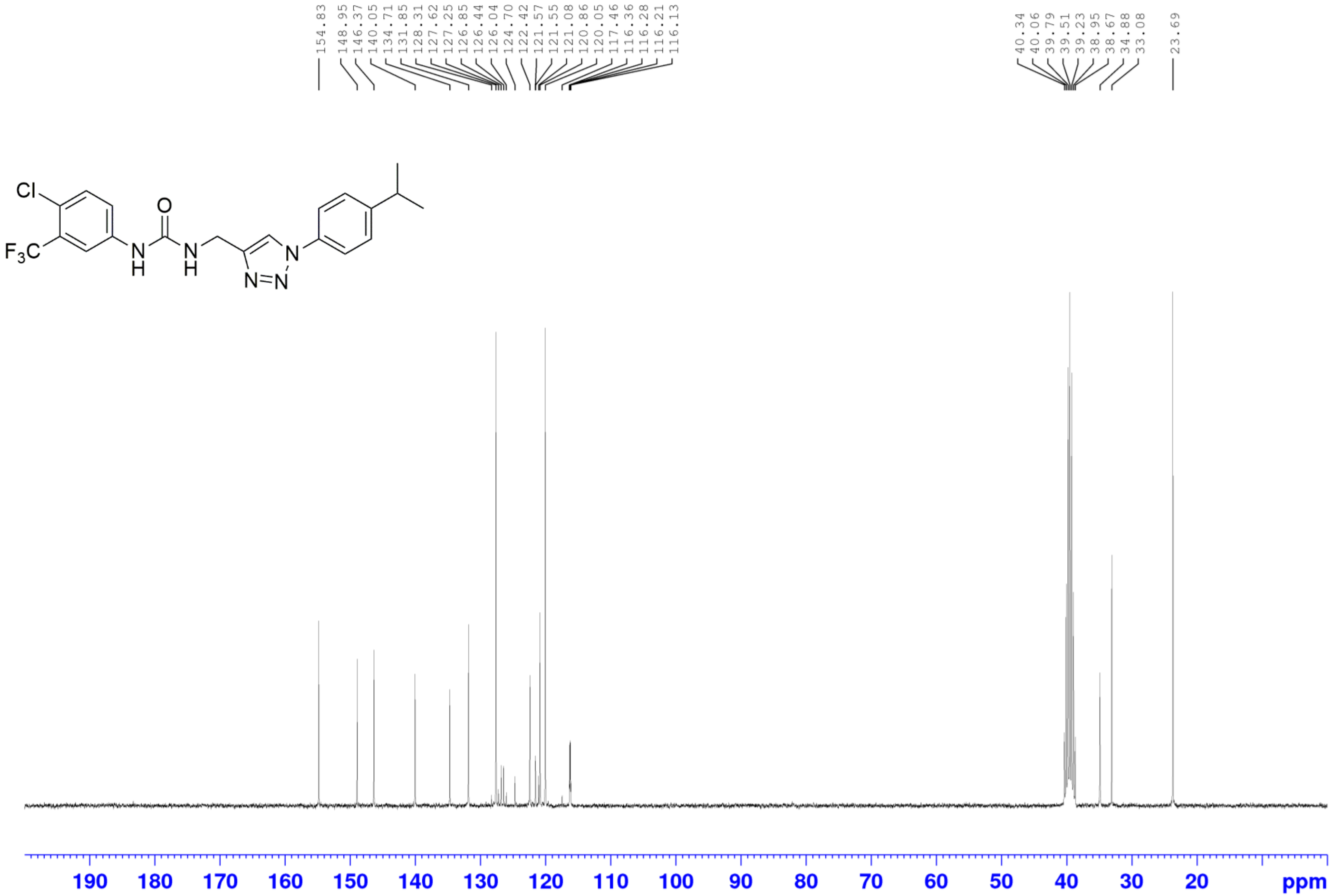

^{19}F NMR of compound **2k’** (282 MHz, DMSO-d_6)

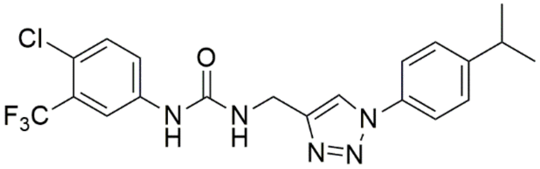

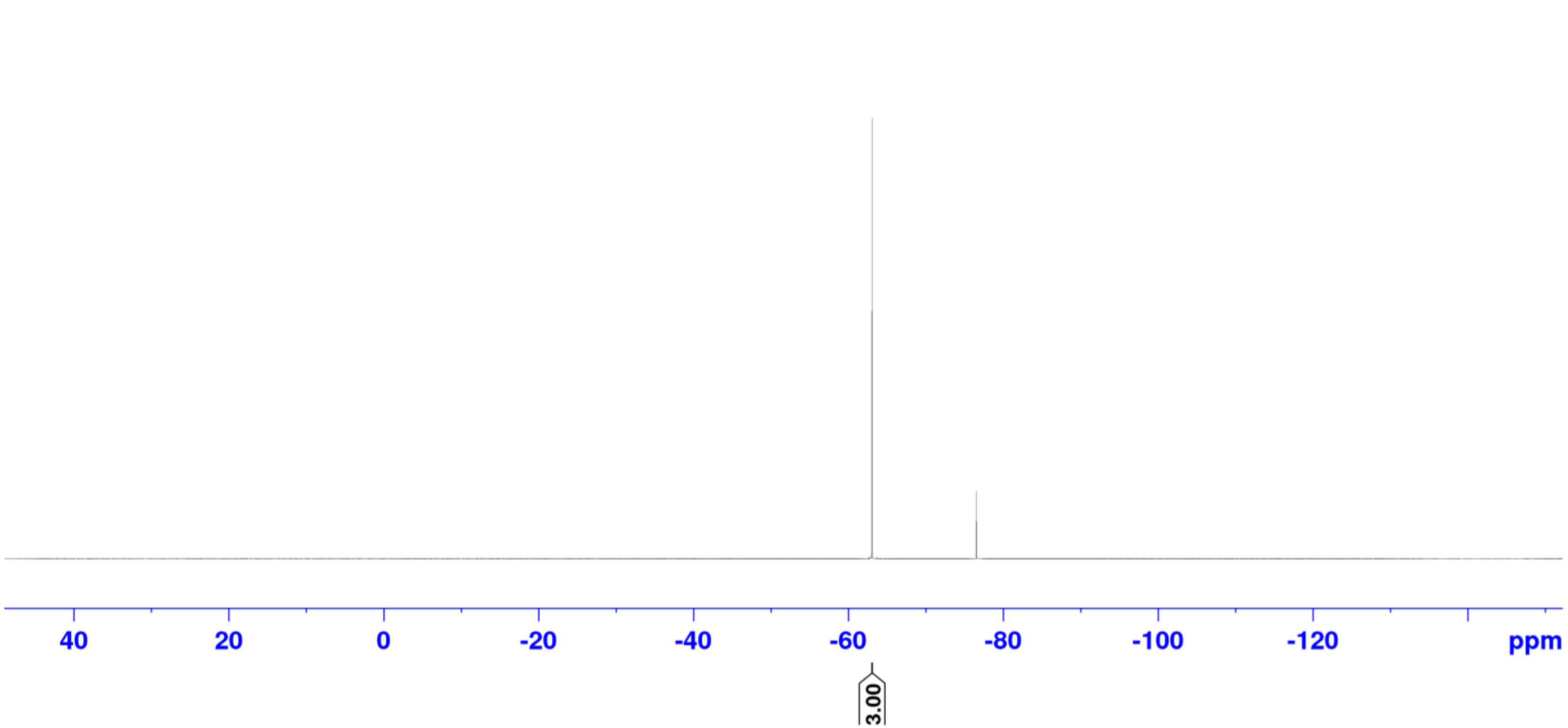

^{1}H NMR of compound **2l’** (300 MHz, DMSO-d_6)

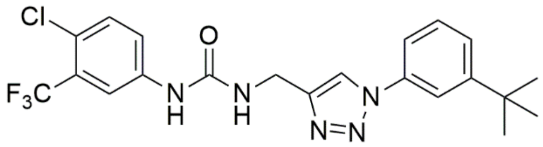

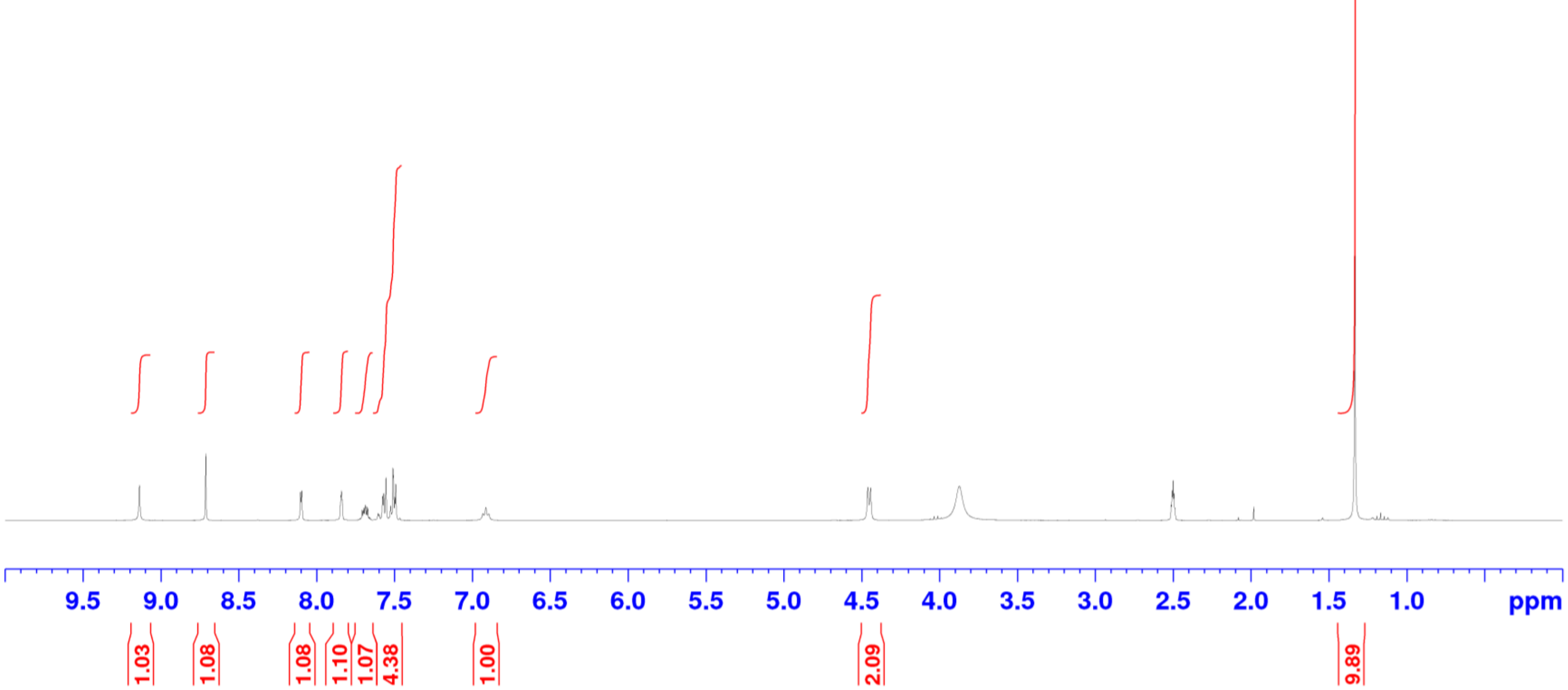

^{13}C NMR of compound **2l'** (75 MHz, DMSO-*d*$_6$)

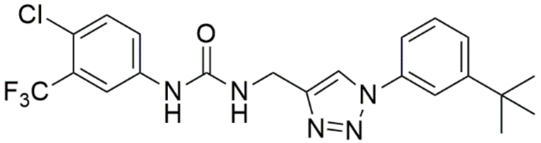

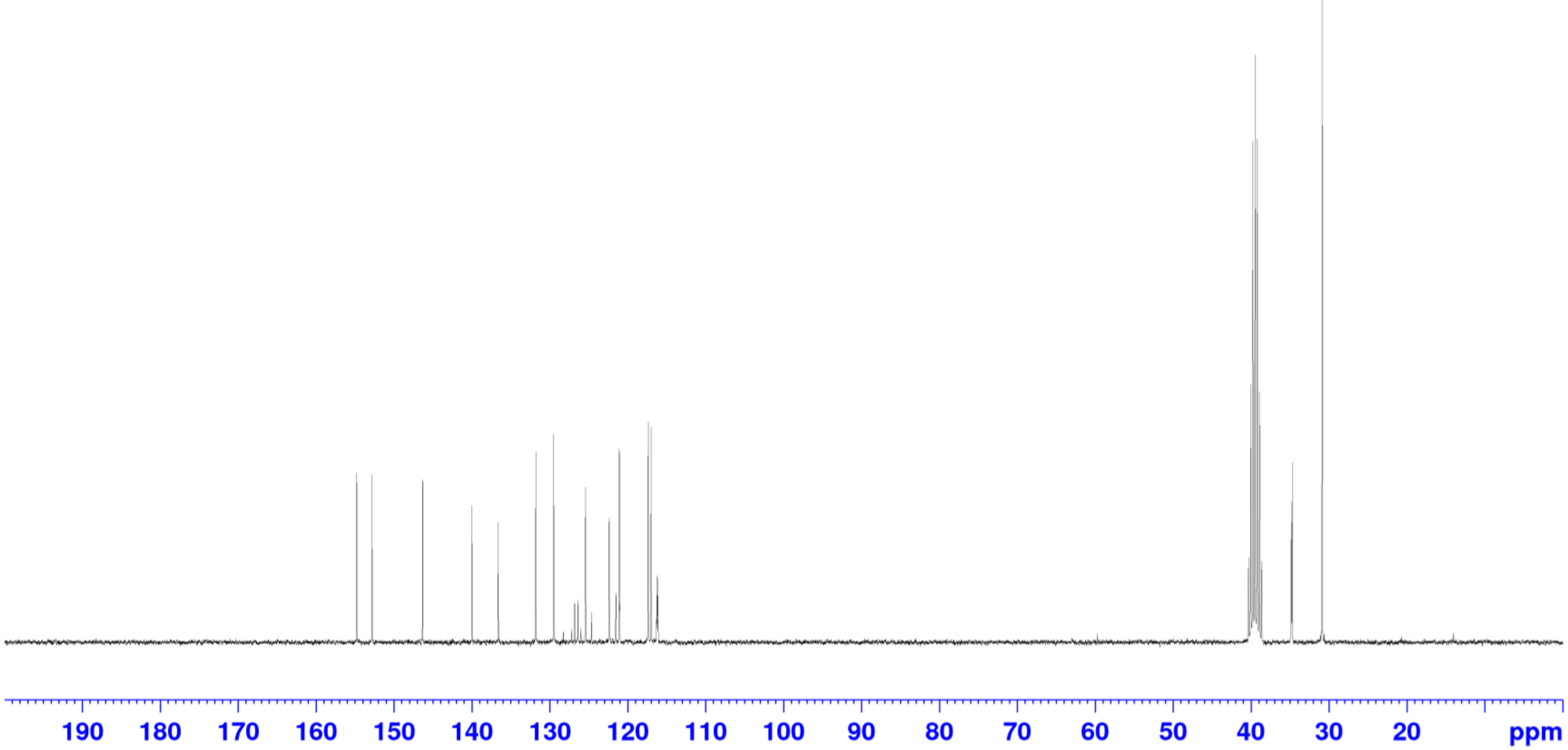

^{19}F NMR of compound **2l’** (282 MHz, DMSO-d_6)

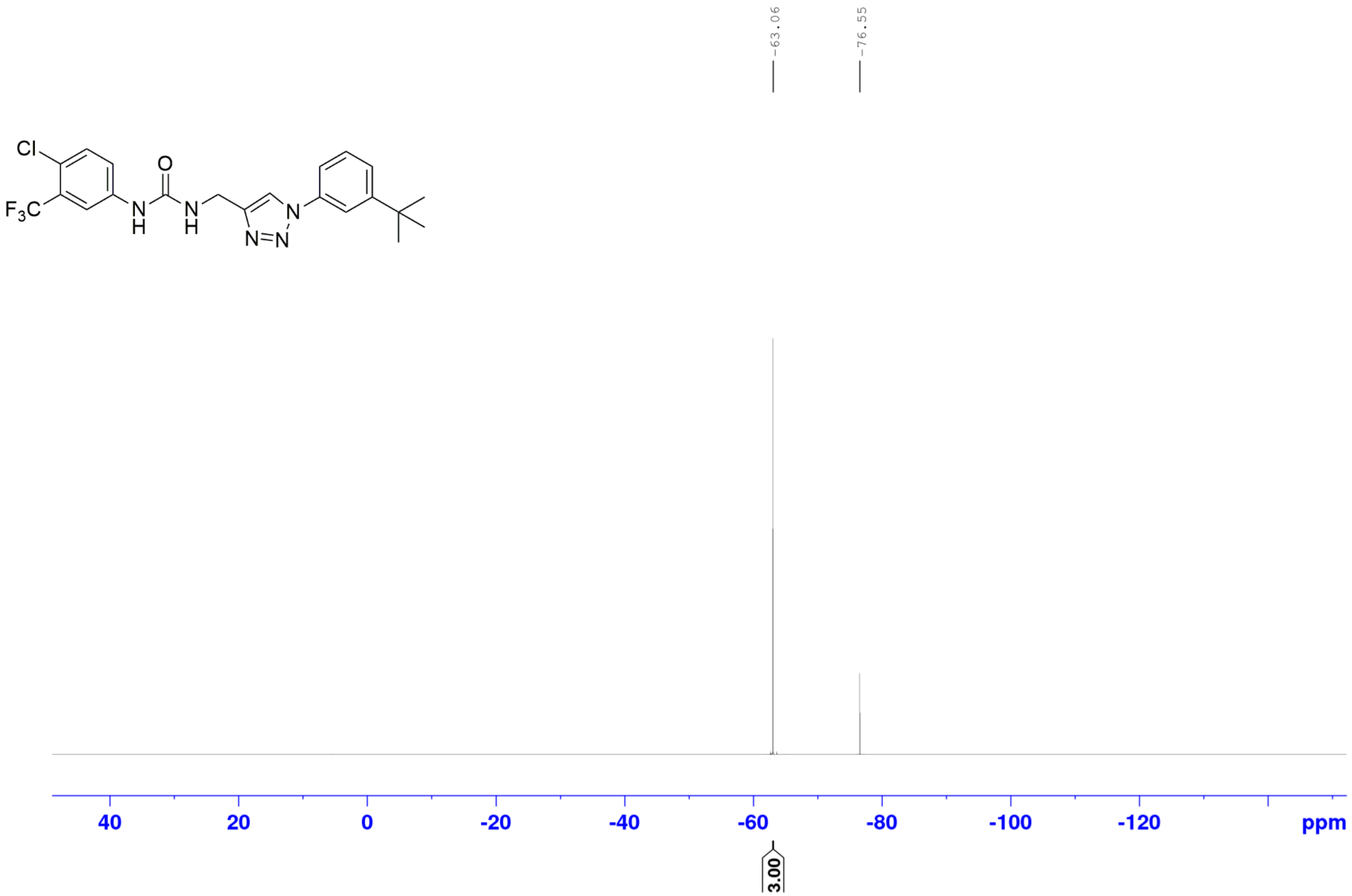

^{1}H NMR of compound **2m'** (300 MHz, DMSO-d_6)

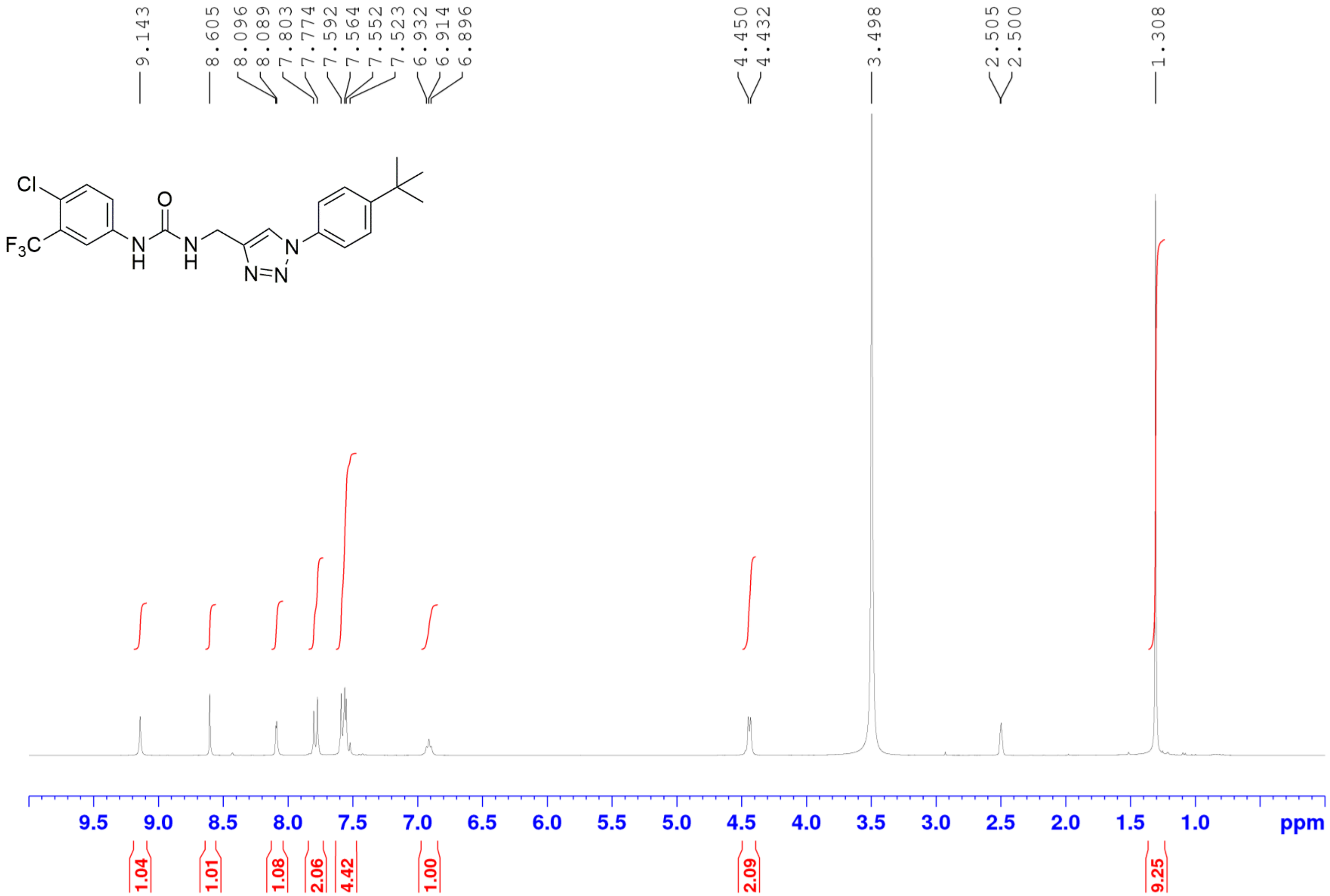

^{13}C NMR of compound **2m'** (75 MHz, DMSO-d_6)

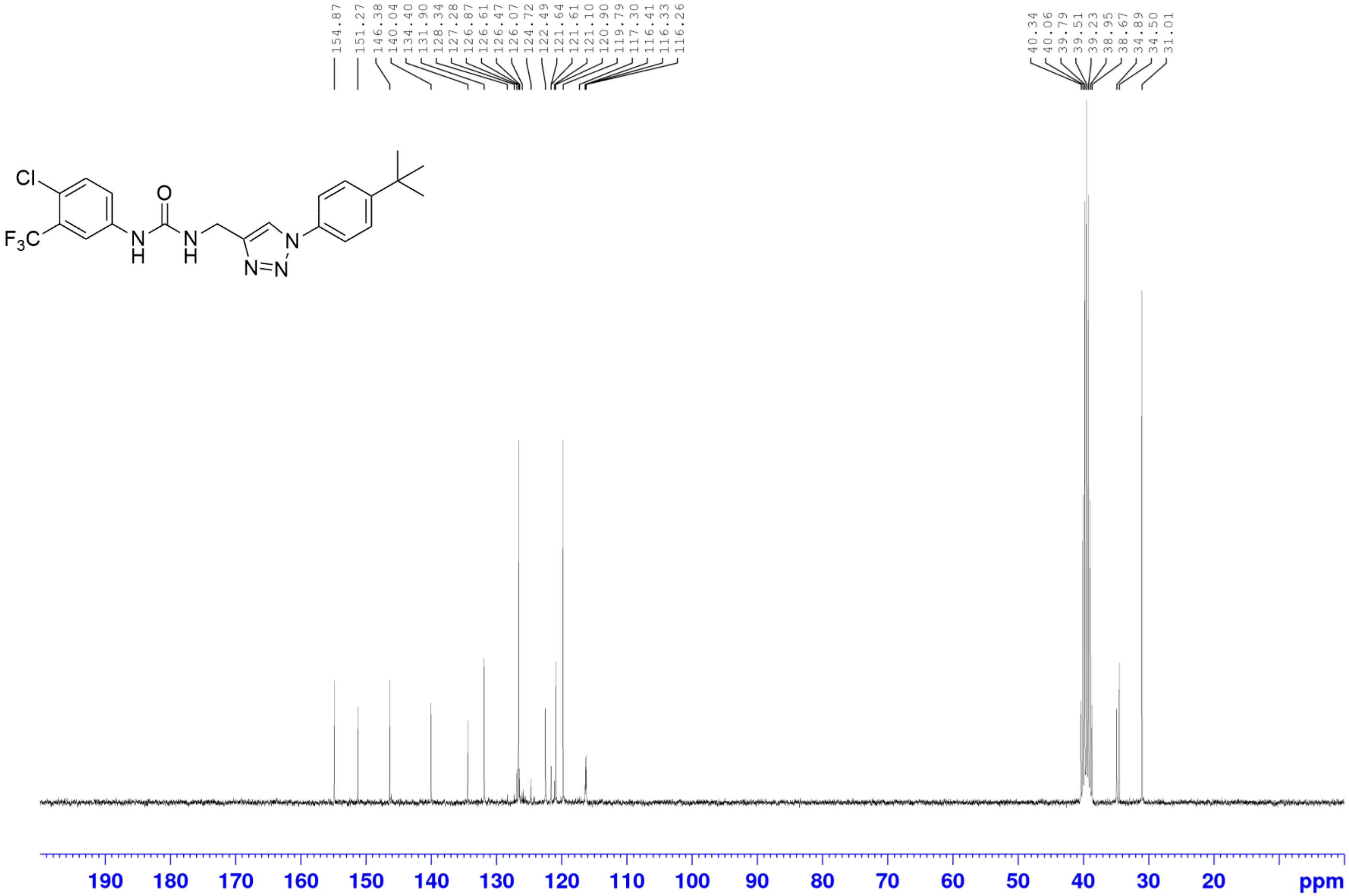

^{19}F NMR of compound **2m’** (282 MHz, DMSO-d_6)

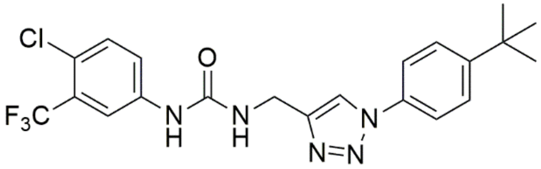

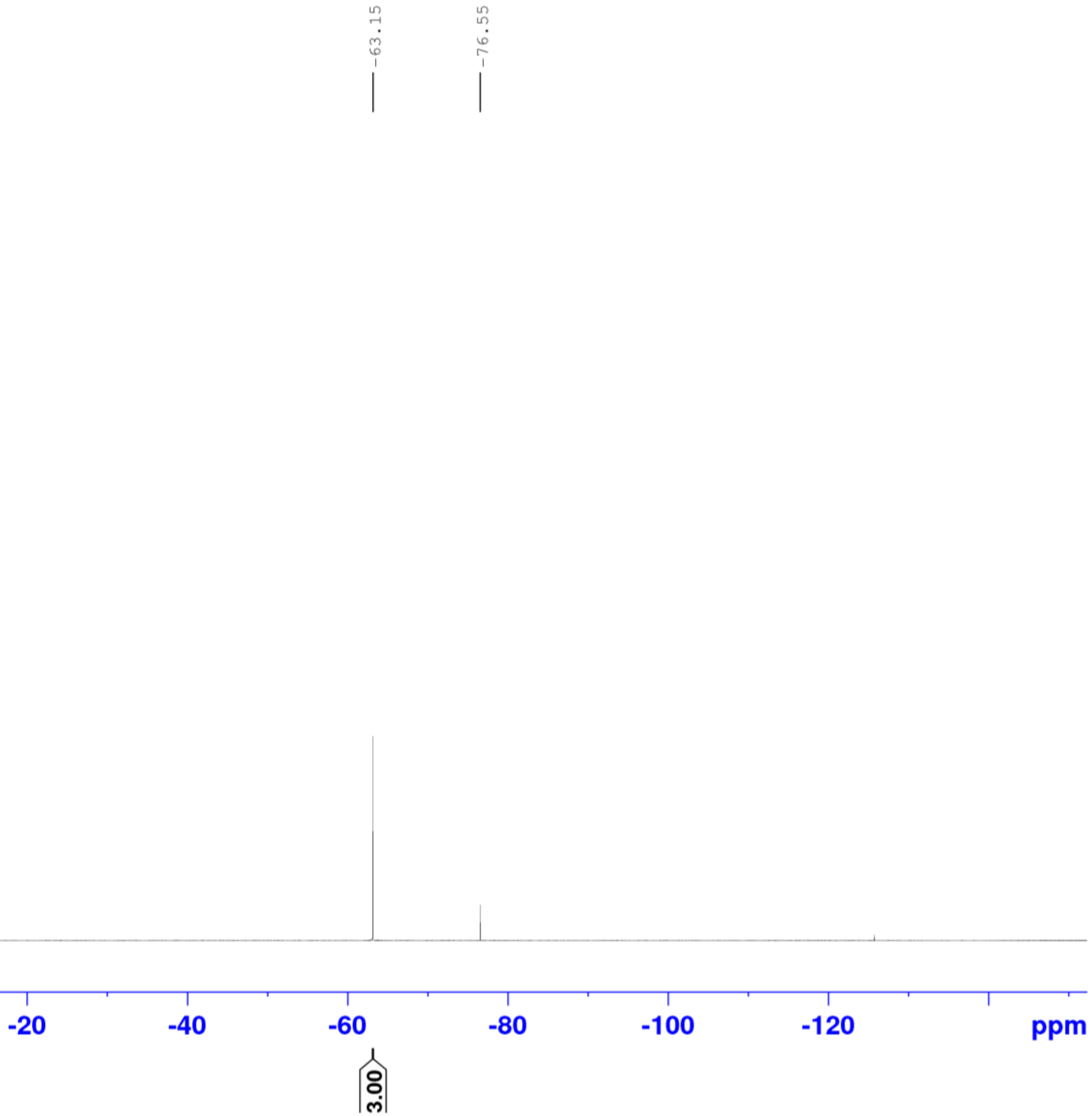

^{1}H NMR of compound **2n'** (300 MHz, DMSO-d_6)

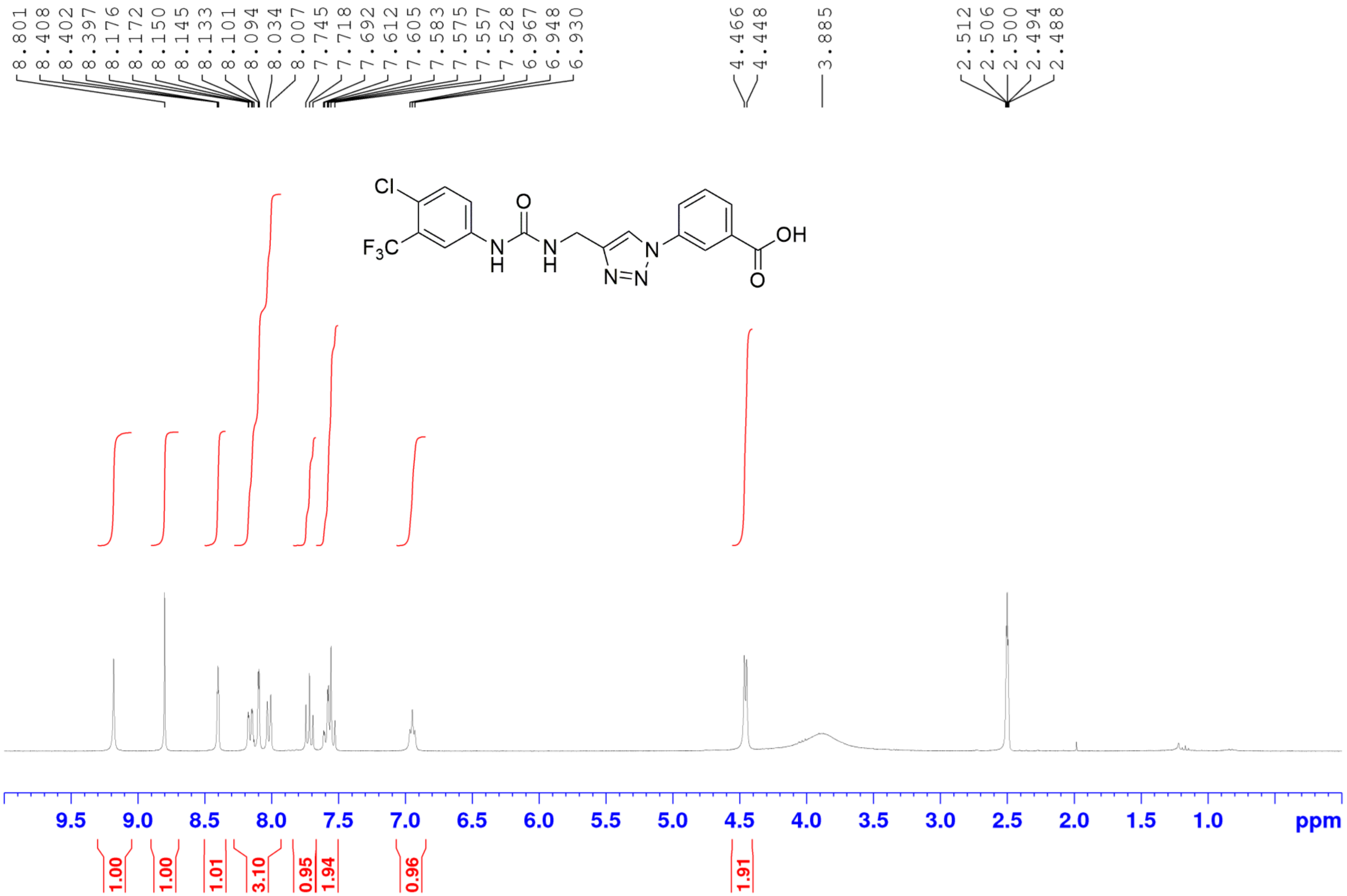

^{13}C NMR of compound **2n’** (75 MHz, DMSO-d_6)

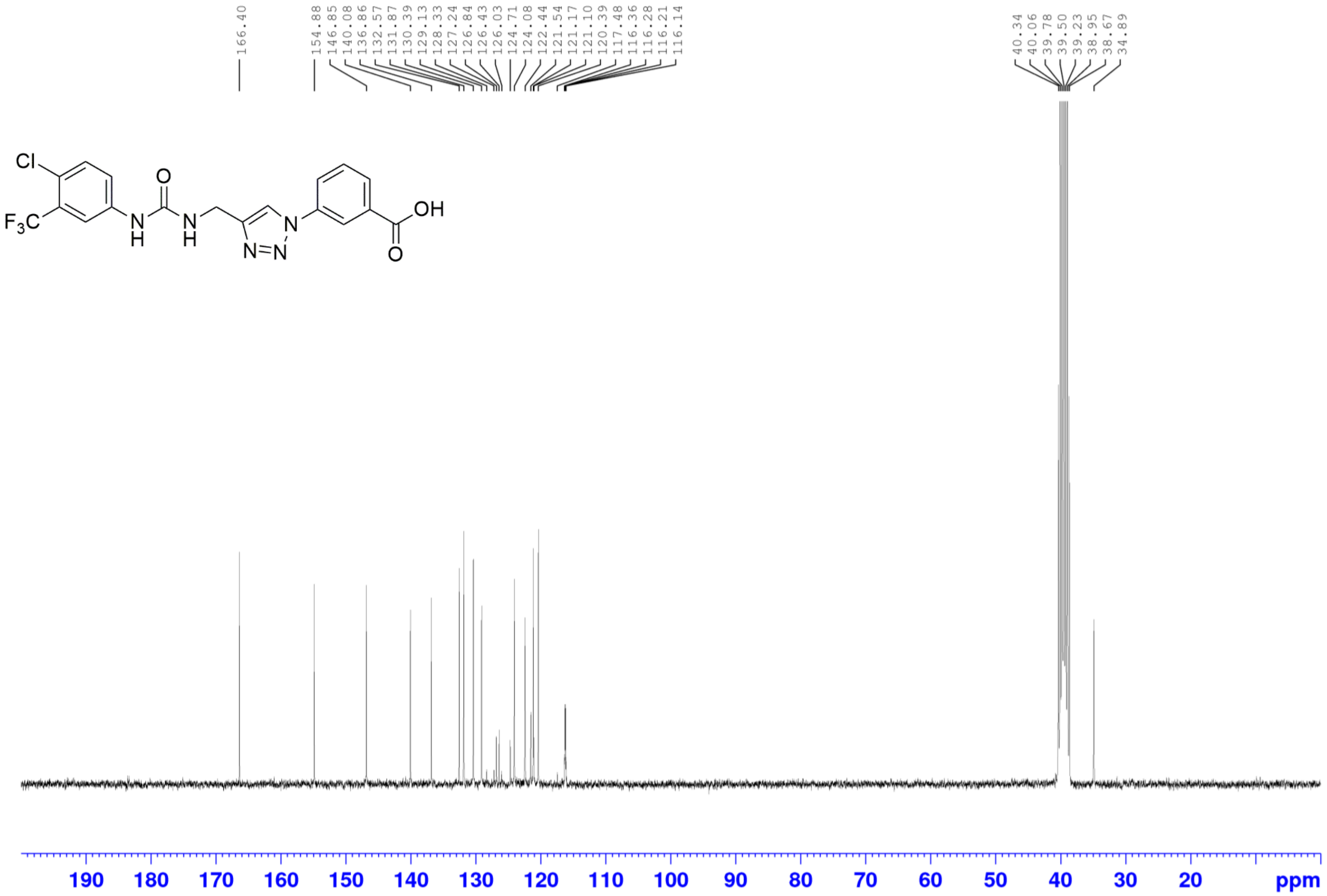

^{19}F NMR of compound **2n’** (282 MHz, DMSO-d_6)

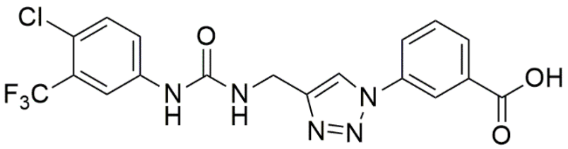

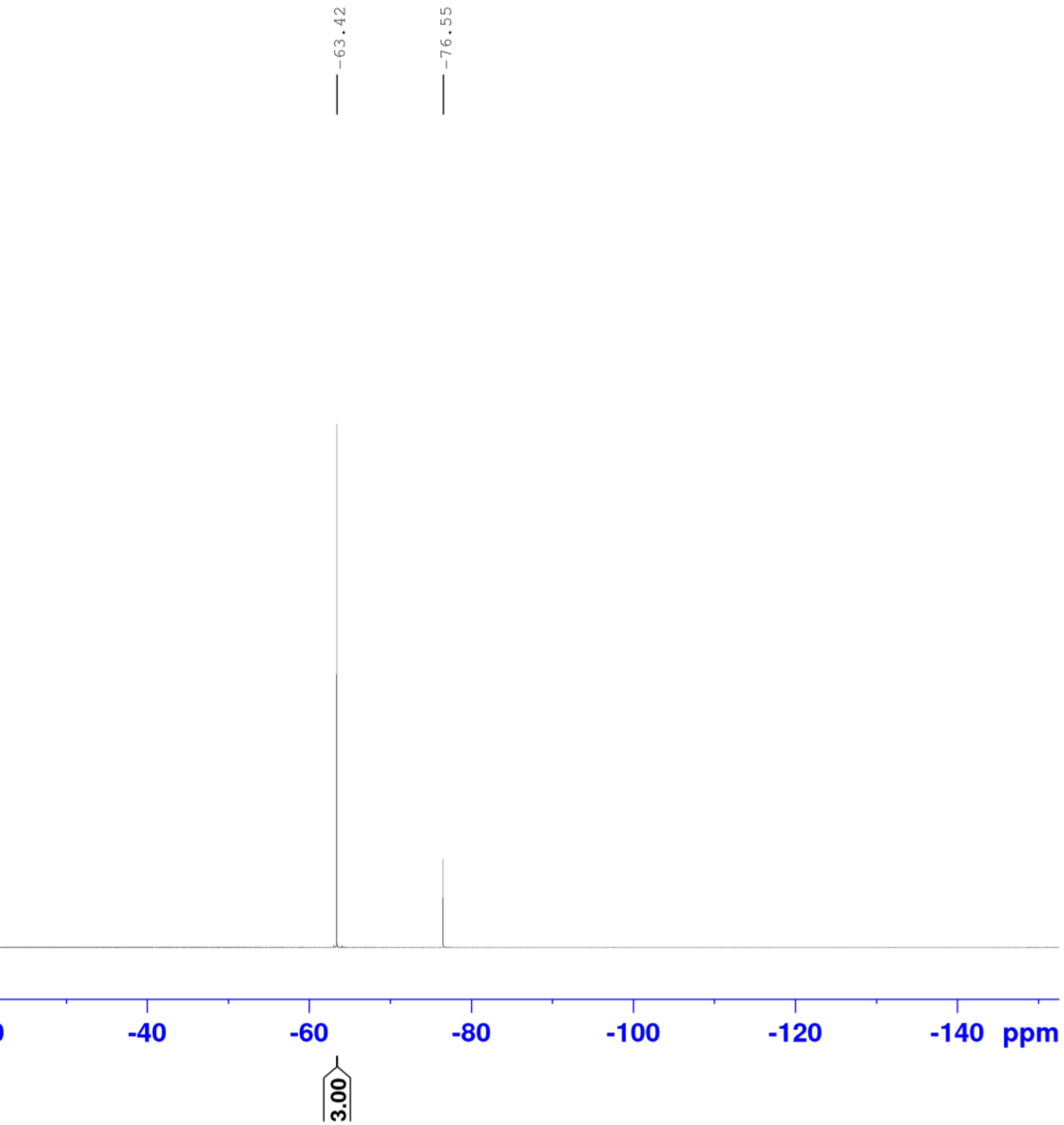

^{1}H NMR of compound **2o'** (300 MHz, DMSO-d_6)

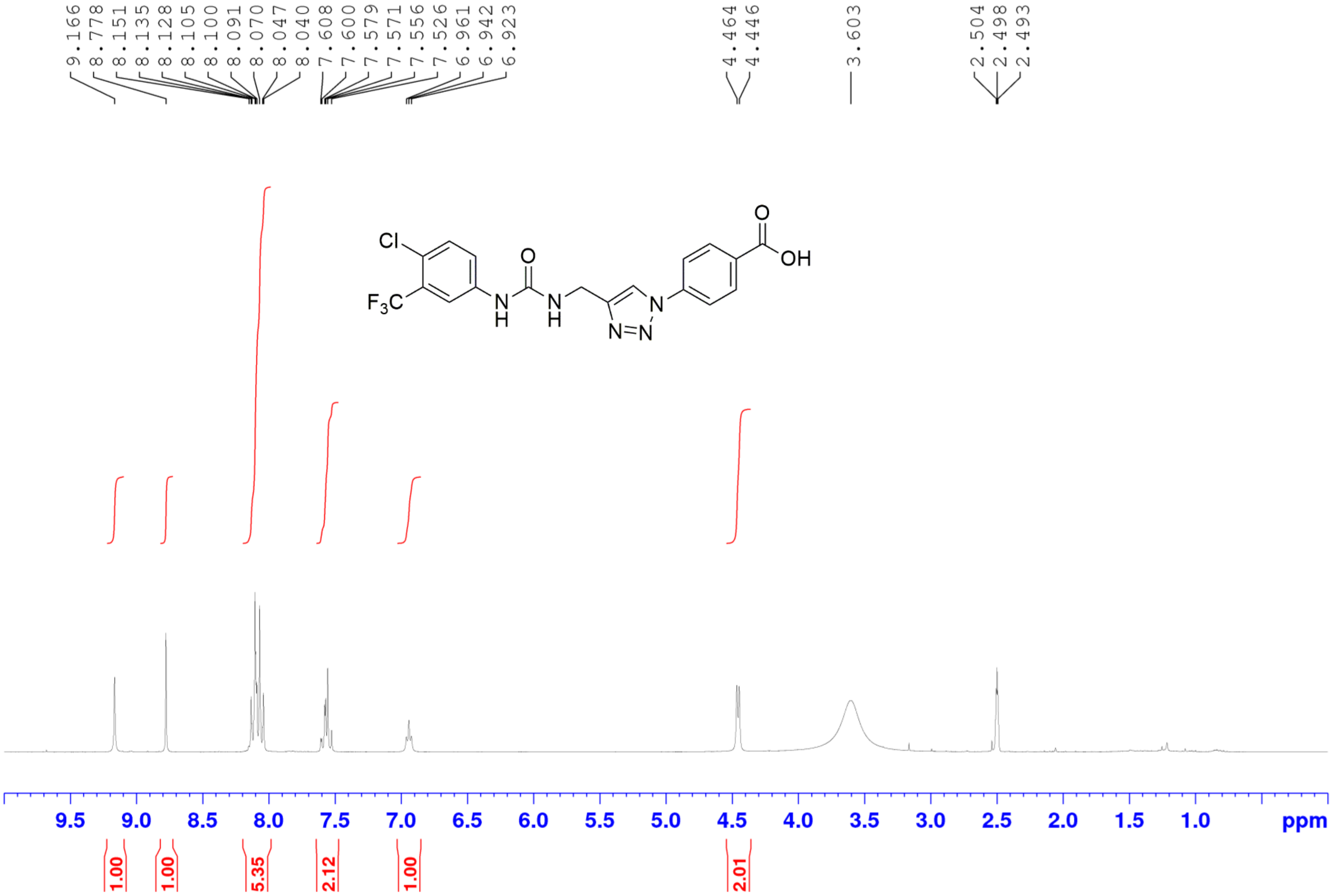

^{13}C NMR of compound **2o'** (75 MHz, DMSO-d_6)

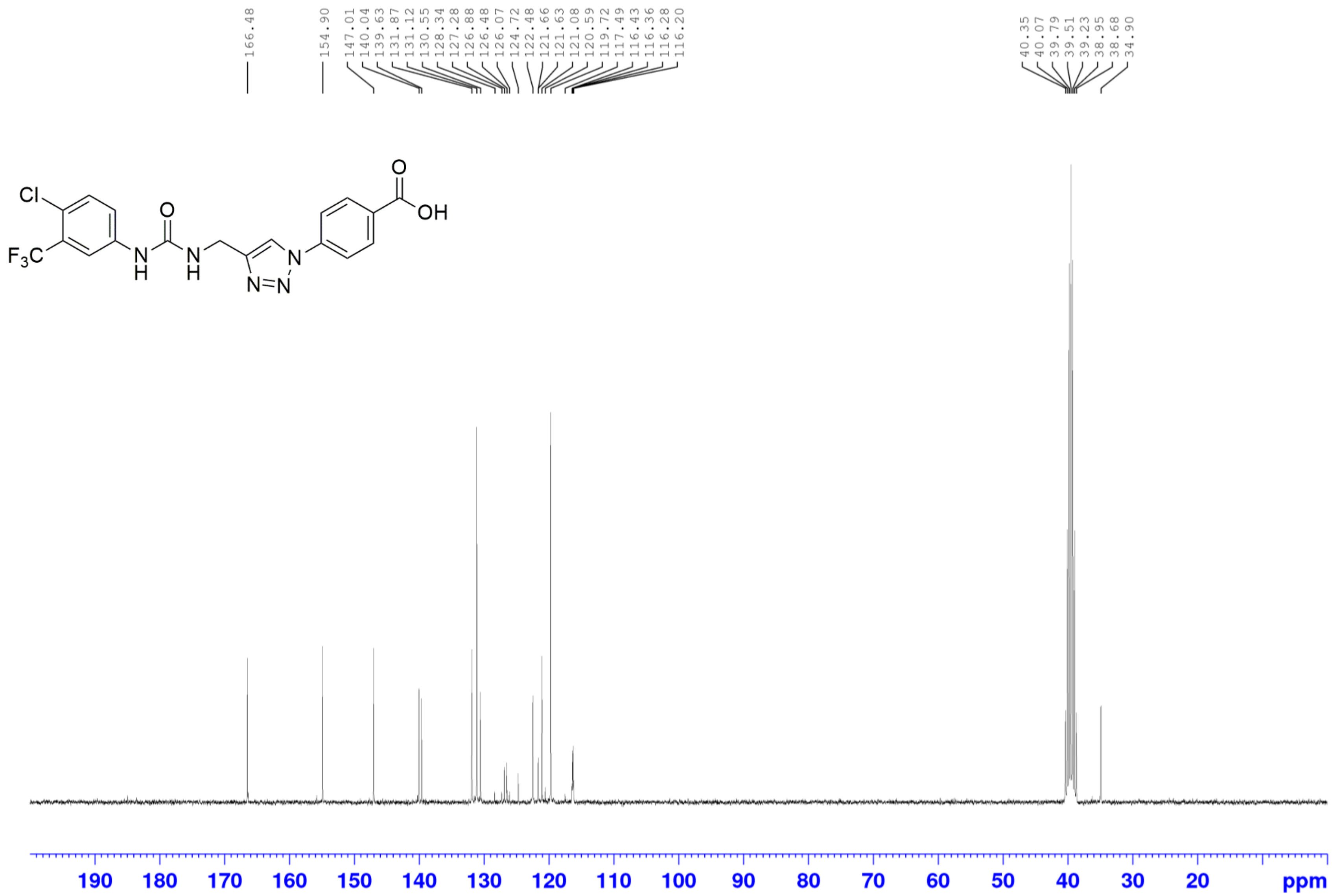

^{19}F NMR of compound **2o'** (282 MHz, DMSO-d_6)

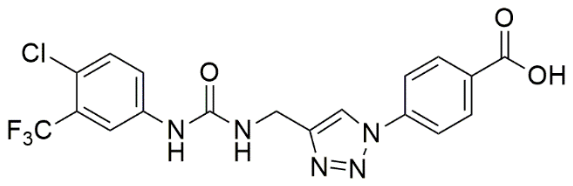

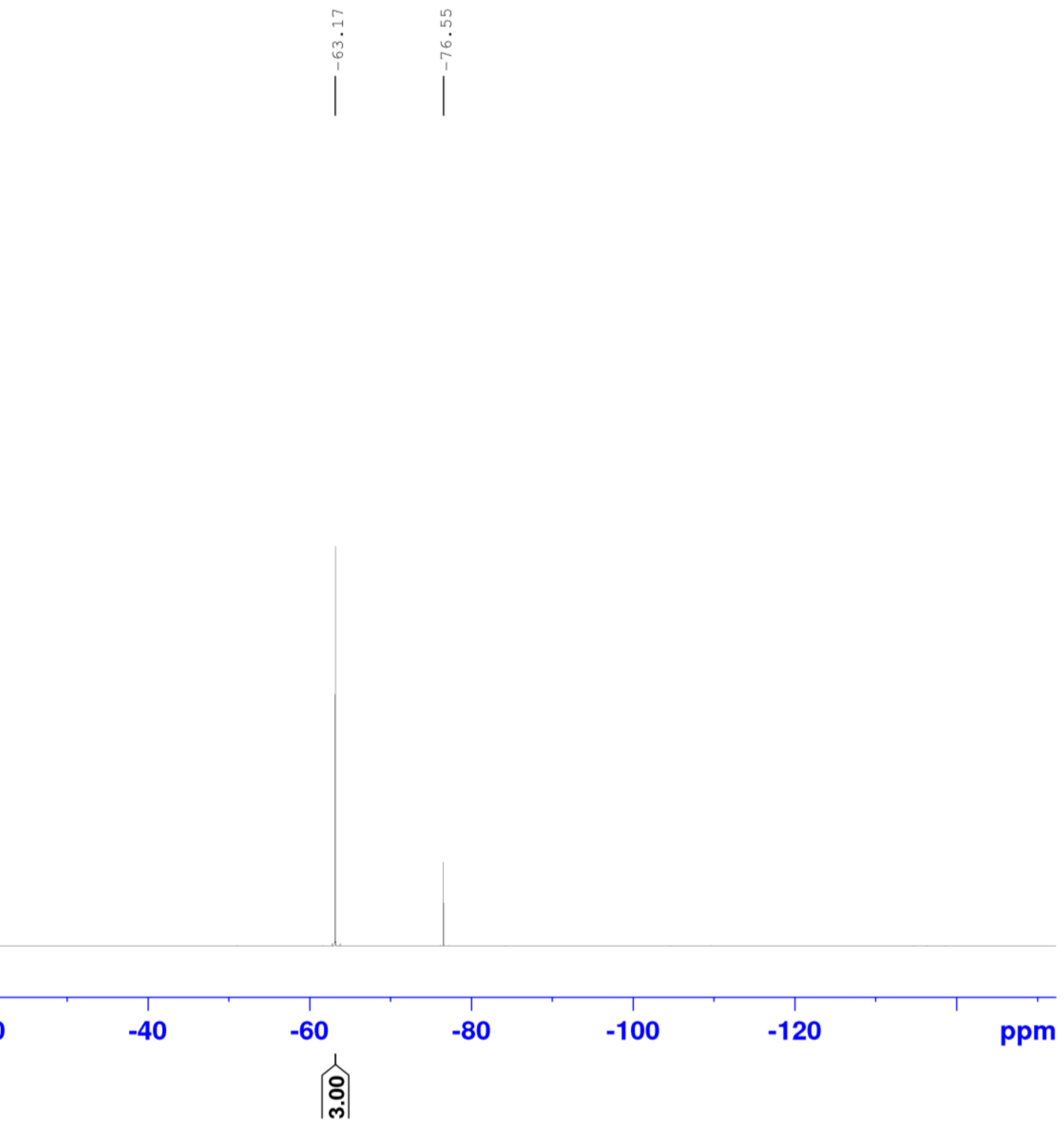

^{1}H NMR of compound **2u’** (300 MHz, DMSO-d_6)

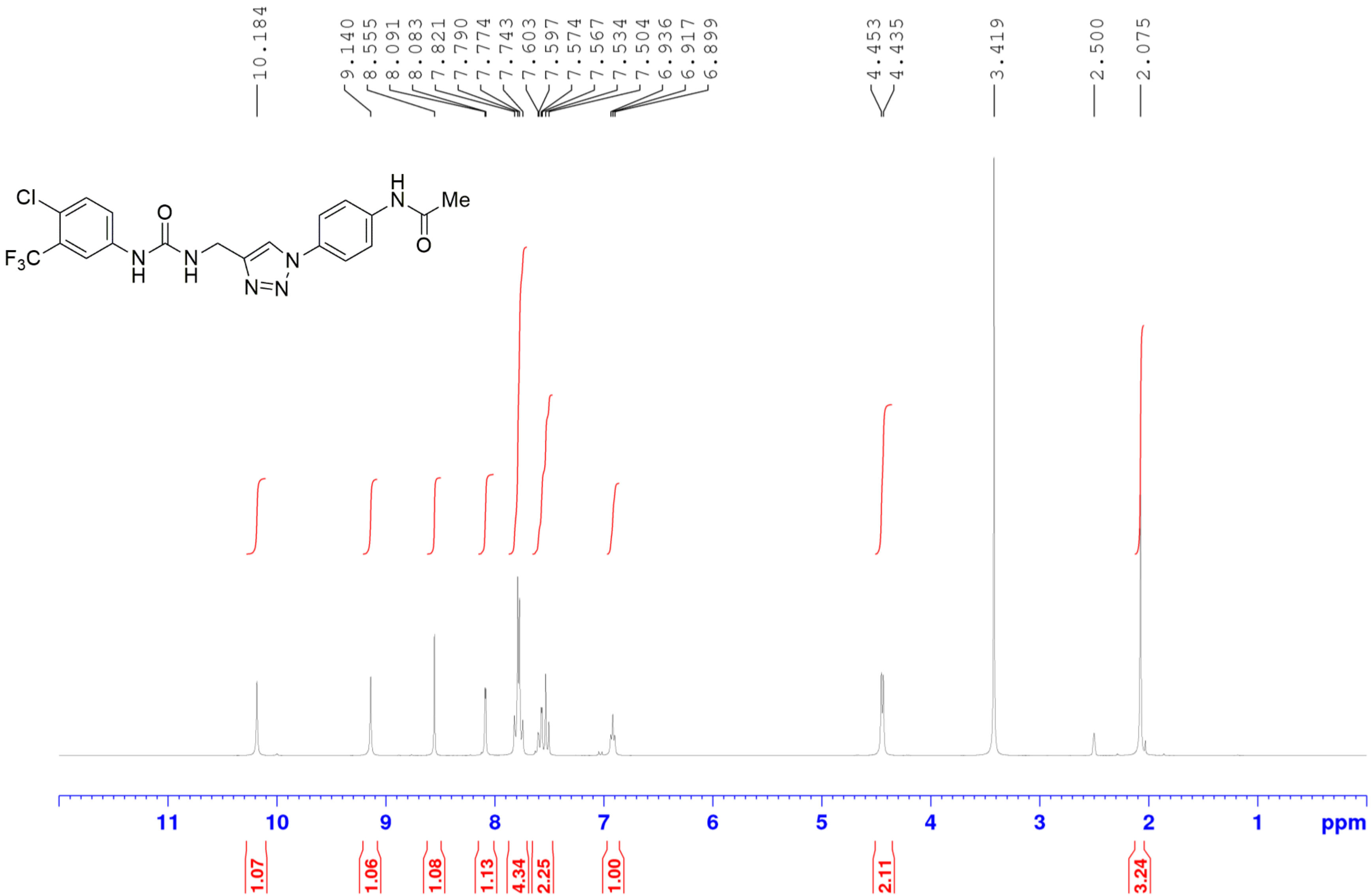

^{13}C NMR of compound **2u'** (75 MHz, DMSO-d_6)

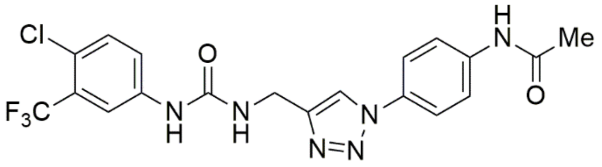

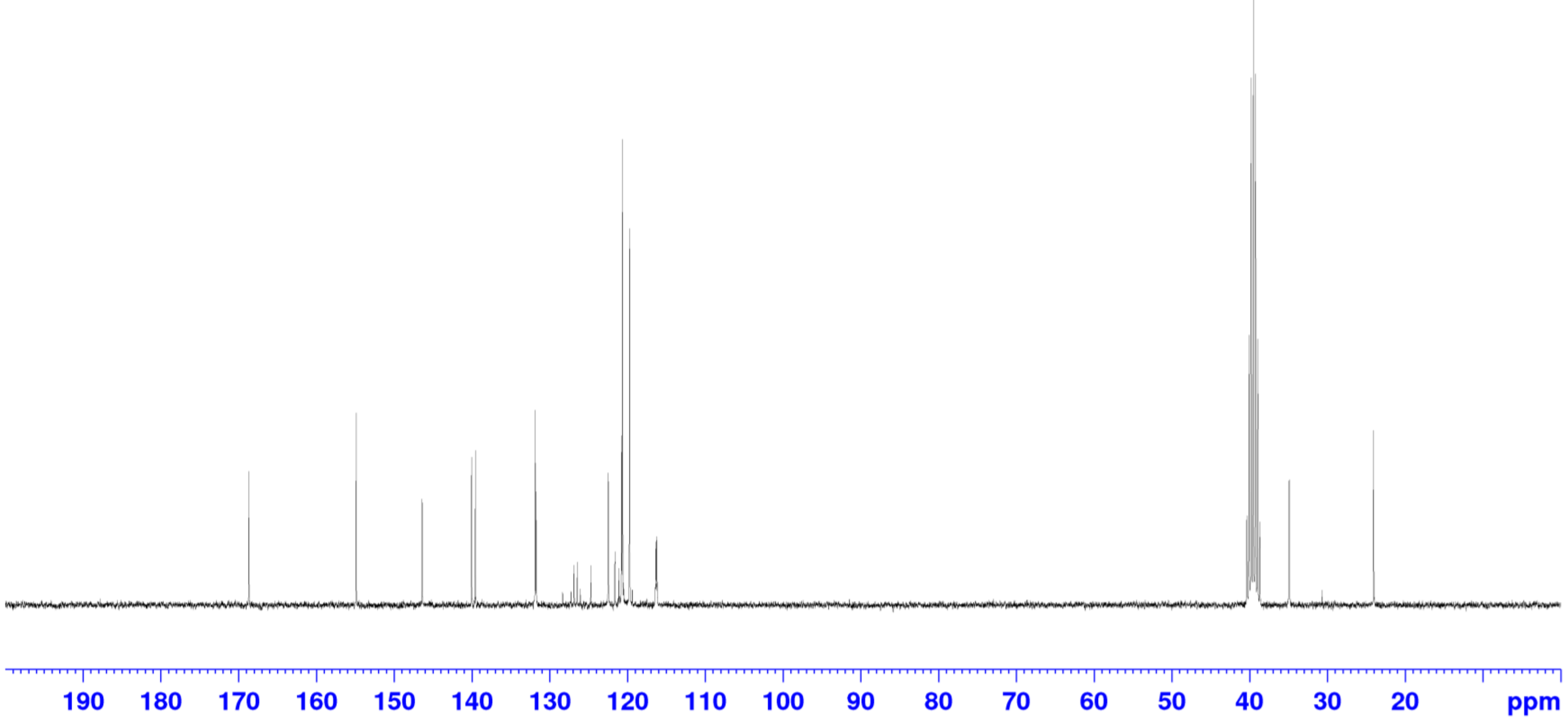

^{19}F NMR of compound **2u'** (282 MHz, DMSO-*d*$_6$)

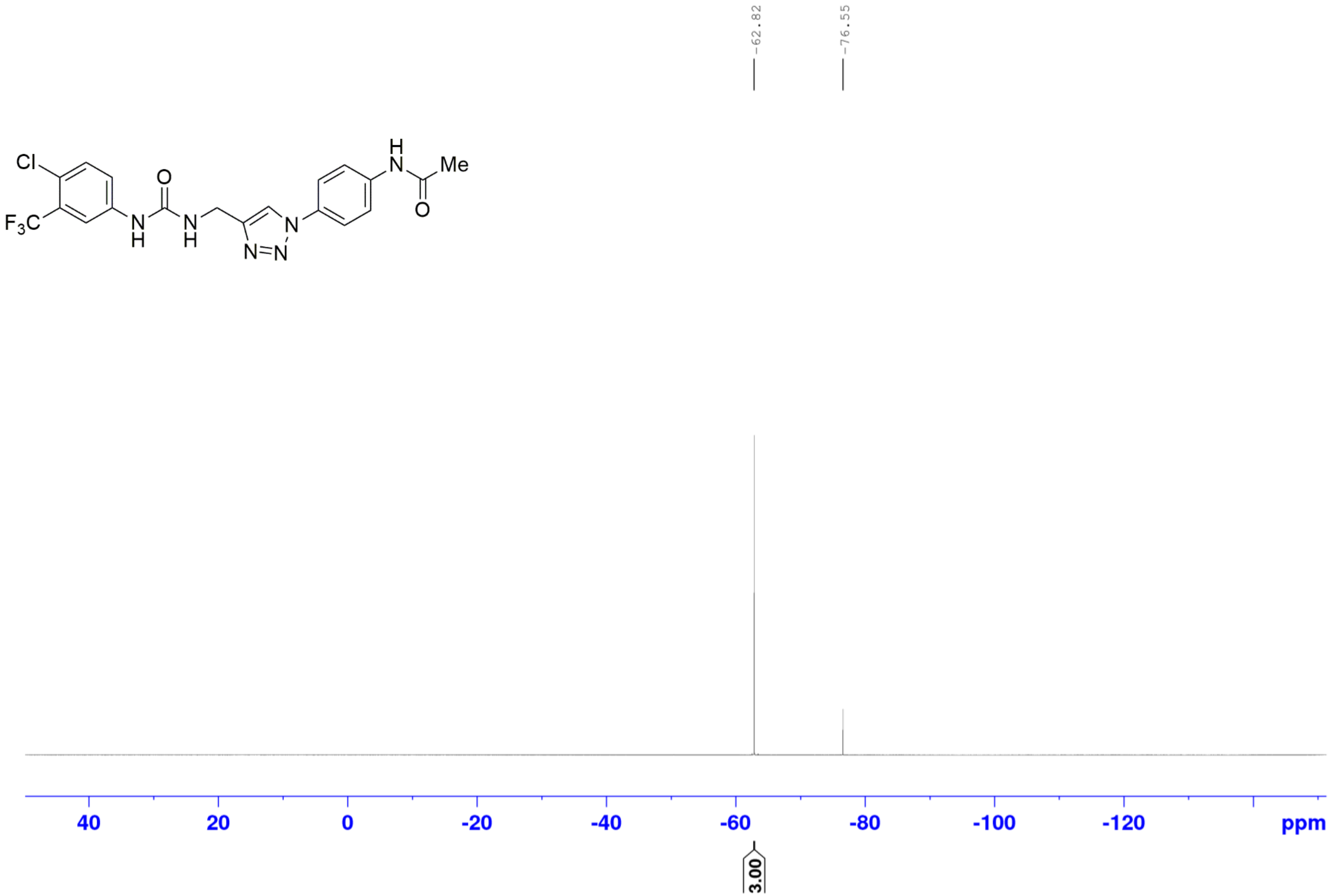

^{1}H NMR of compound **2p'** (300 MHz, DMSO-d_6)

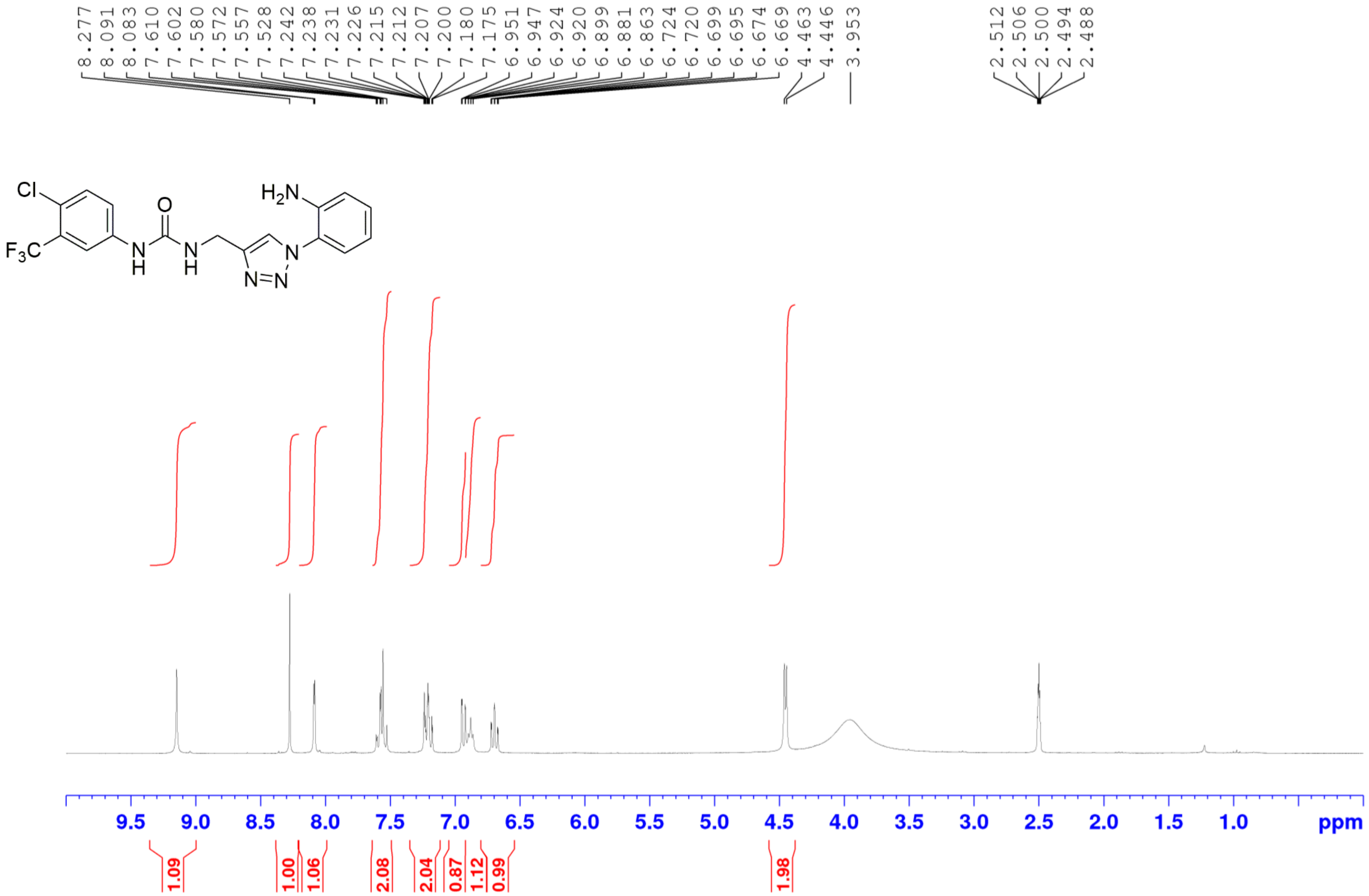

^{13}C NMR of compound **2p'** (75 MHz, DMSO-d_6)

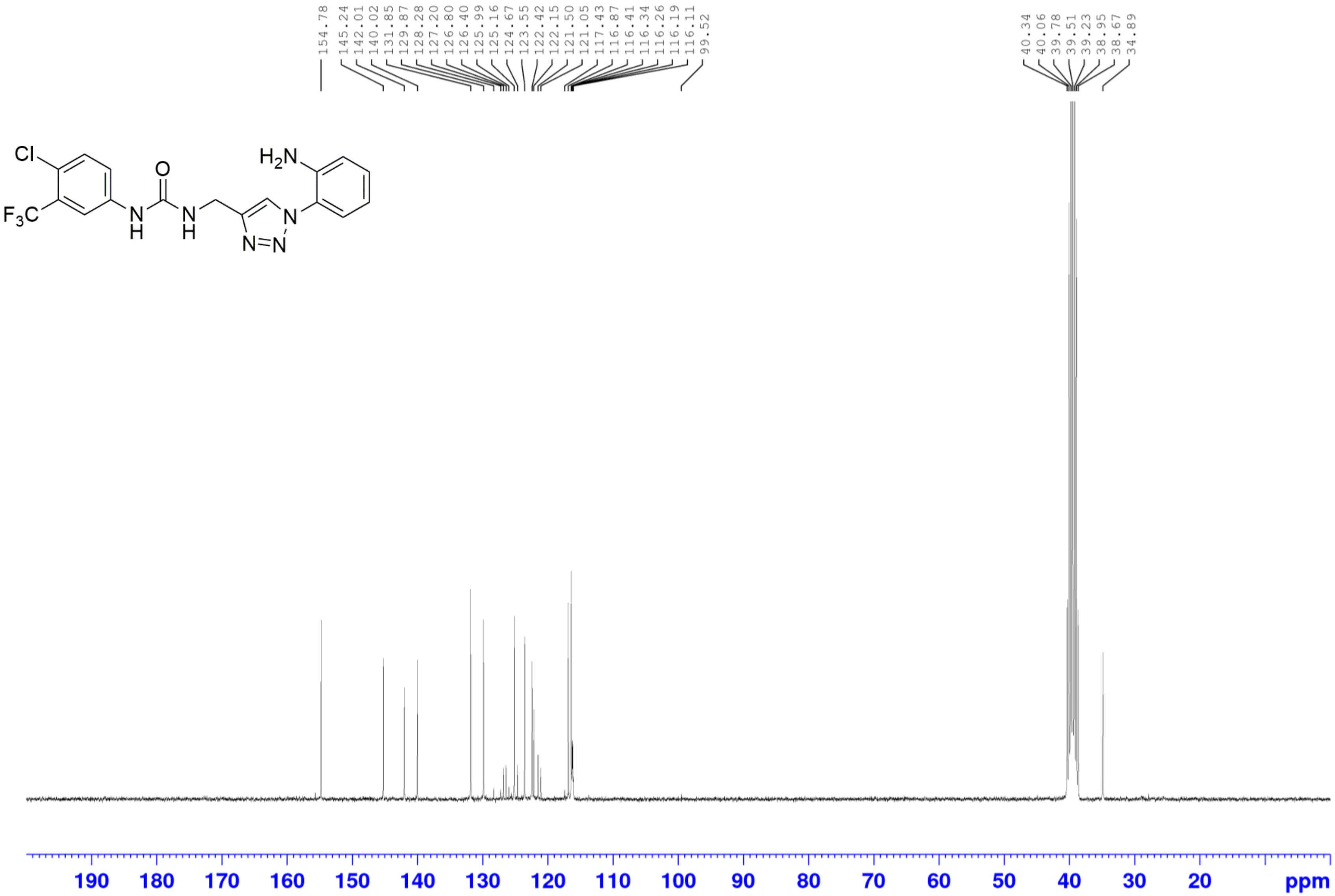

^{19}F NMR of compound **2p'** (282 MHz, DMSO-d_6)

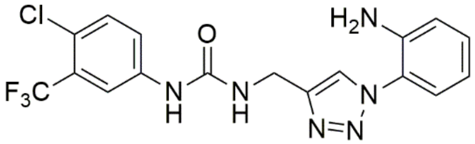

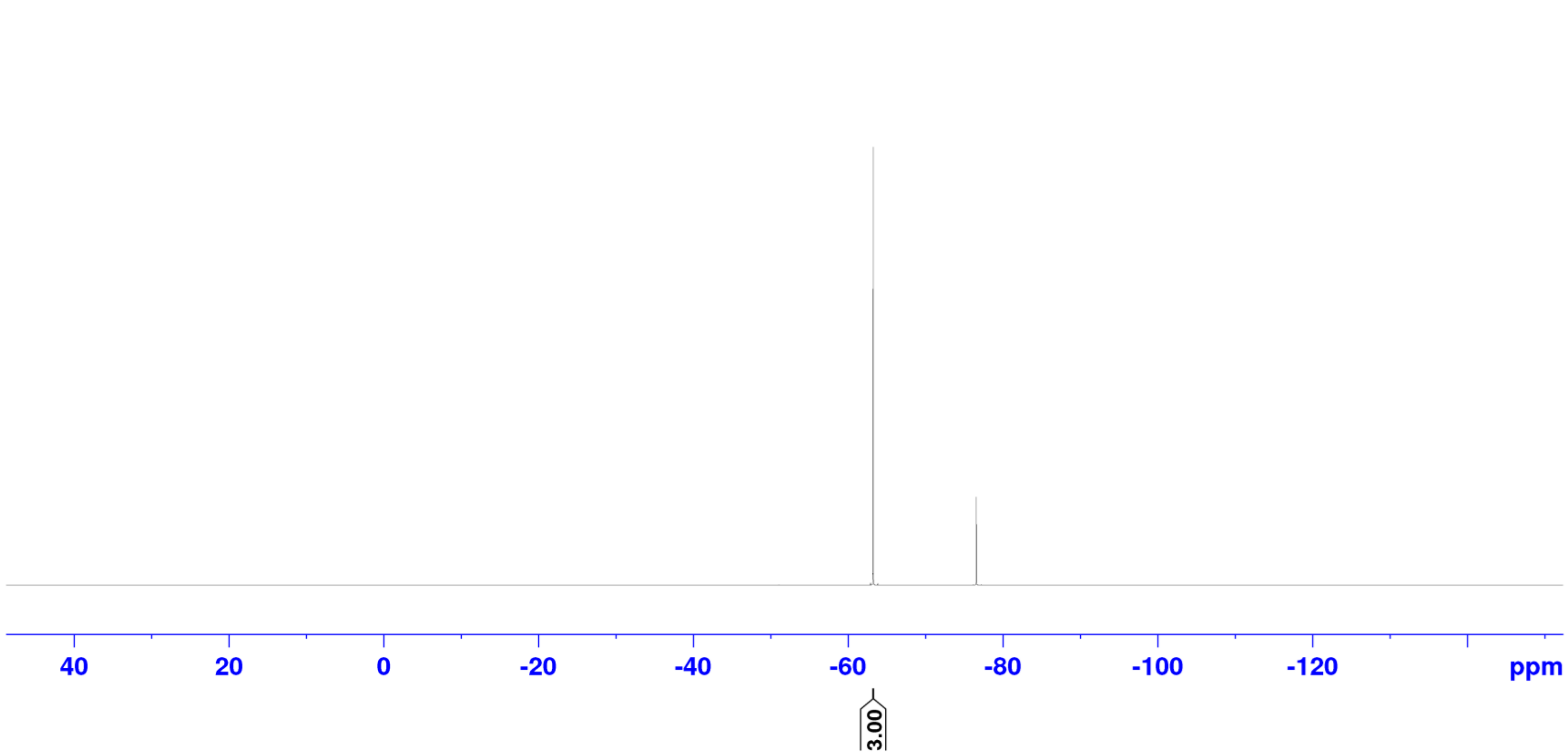

^{1}H NMR of compound **2q'** (300 MHz, DMSO-d_6)

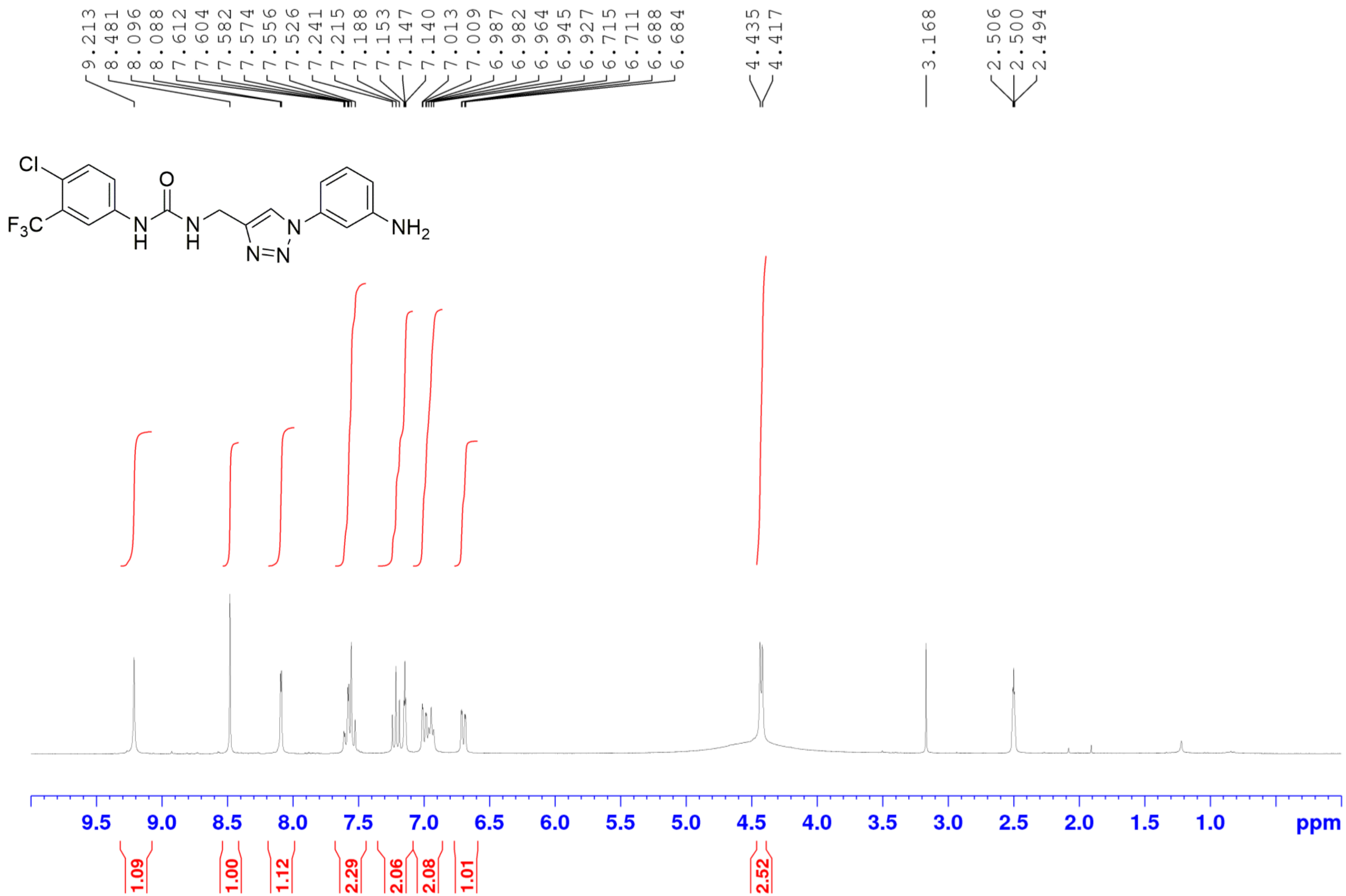

^{13}C NMR of compound **2q'** (75 MHz, DMSO-d_6)

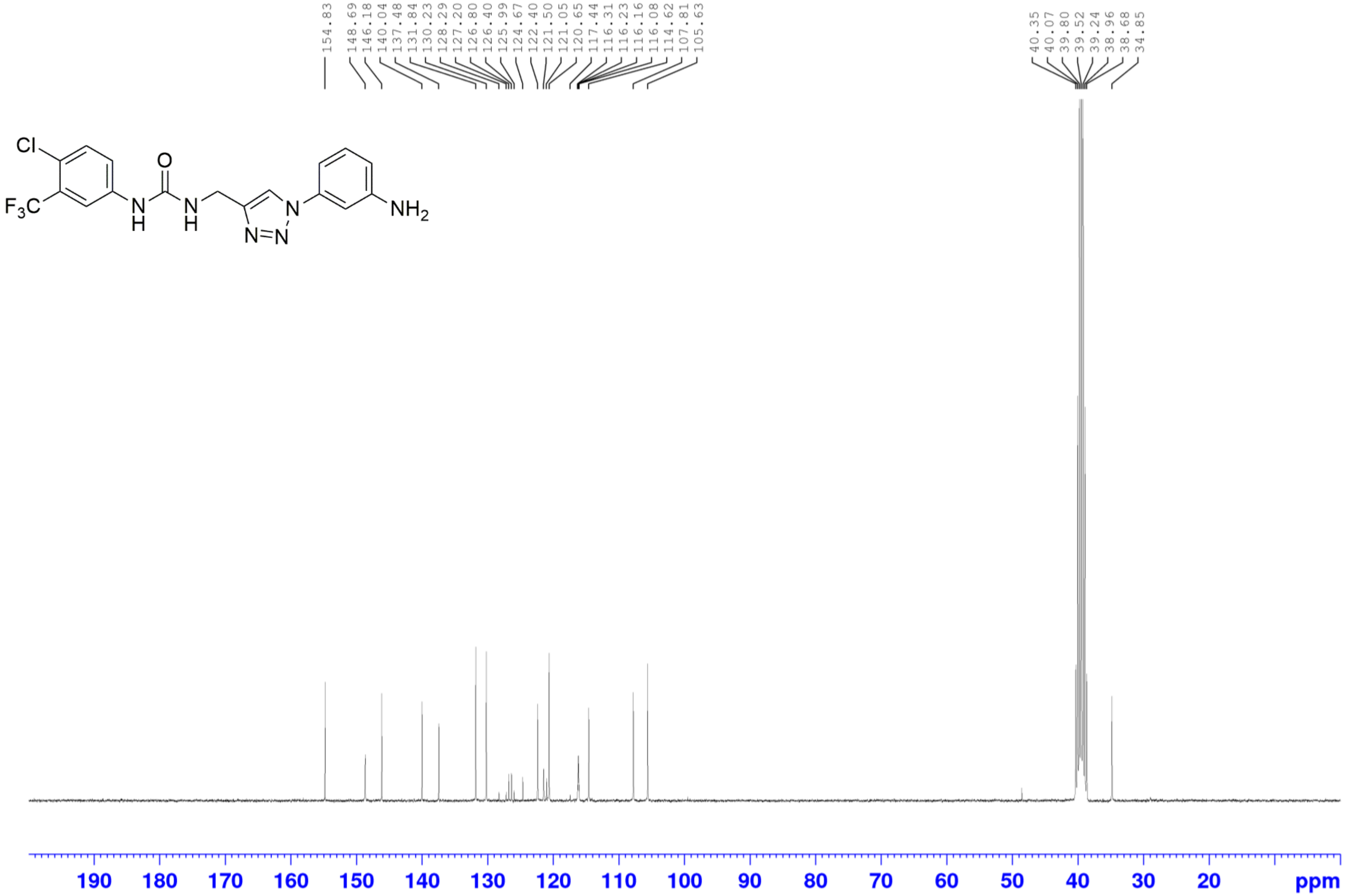

^{19}F NMR of compound **2q'** (282 MHz, DMSO-d_6)

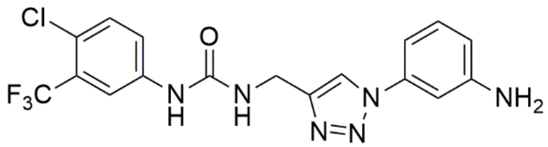

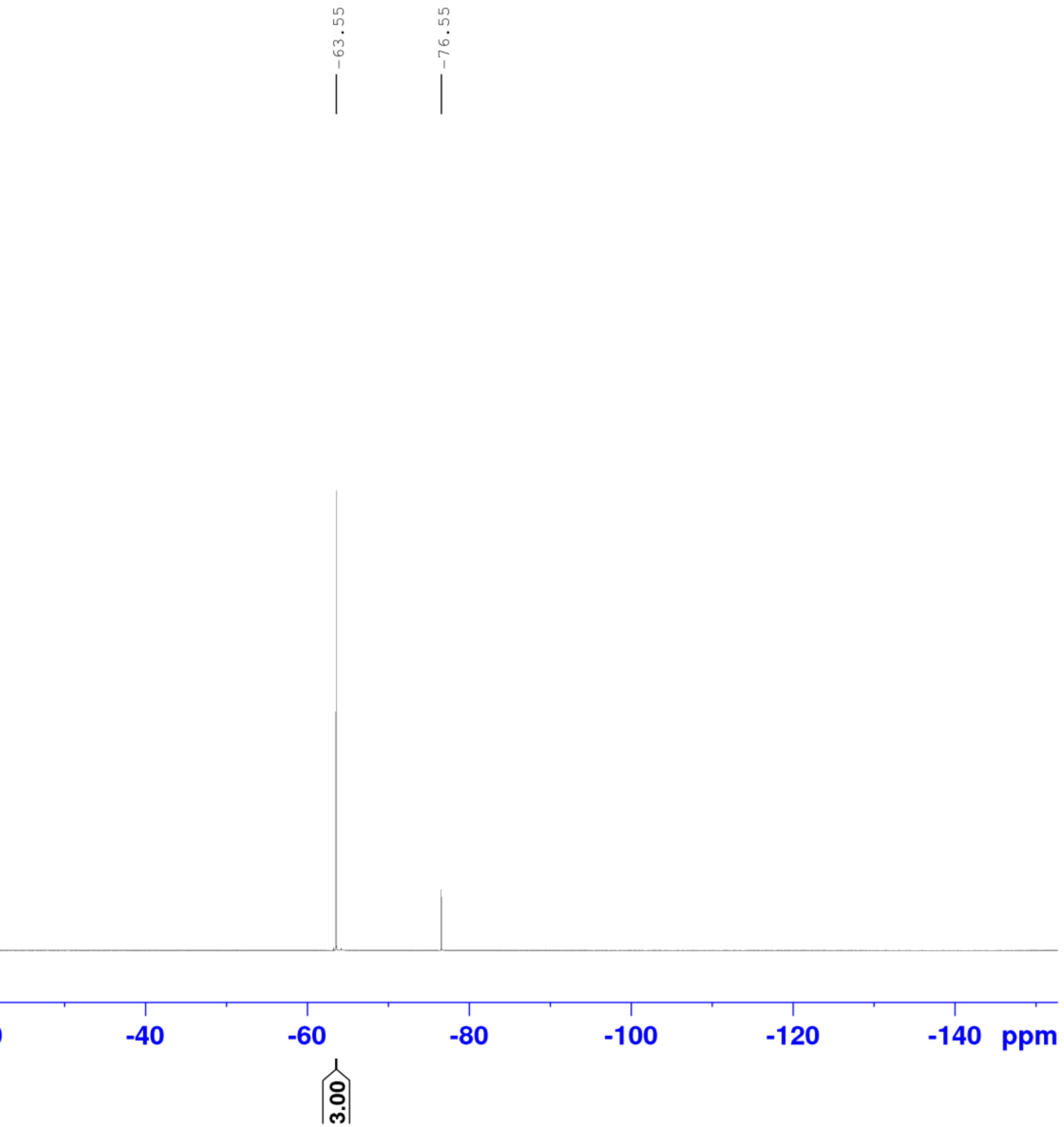

^{1}H NMR of compound **2r'** (300 MHz, DMSO-d_6)

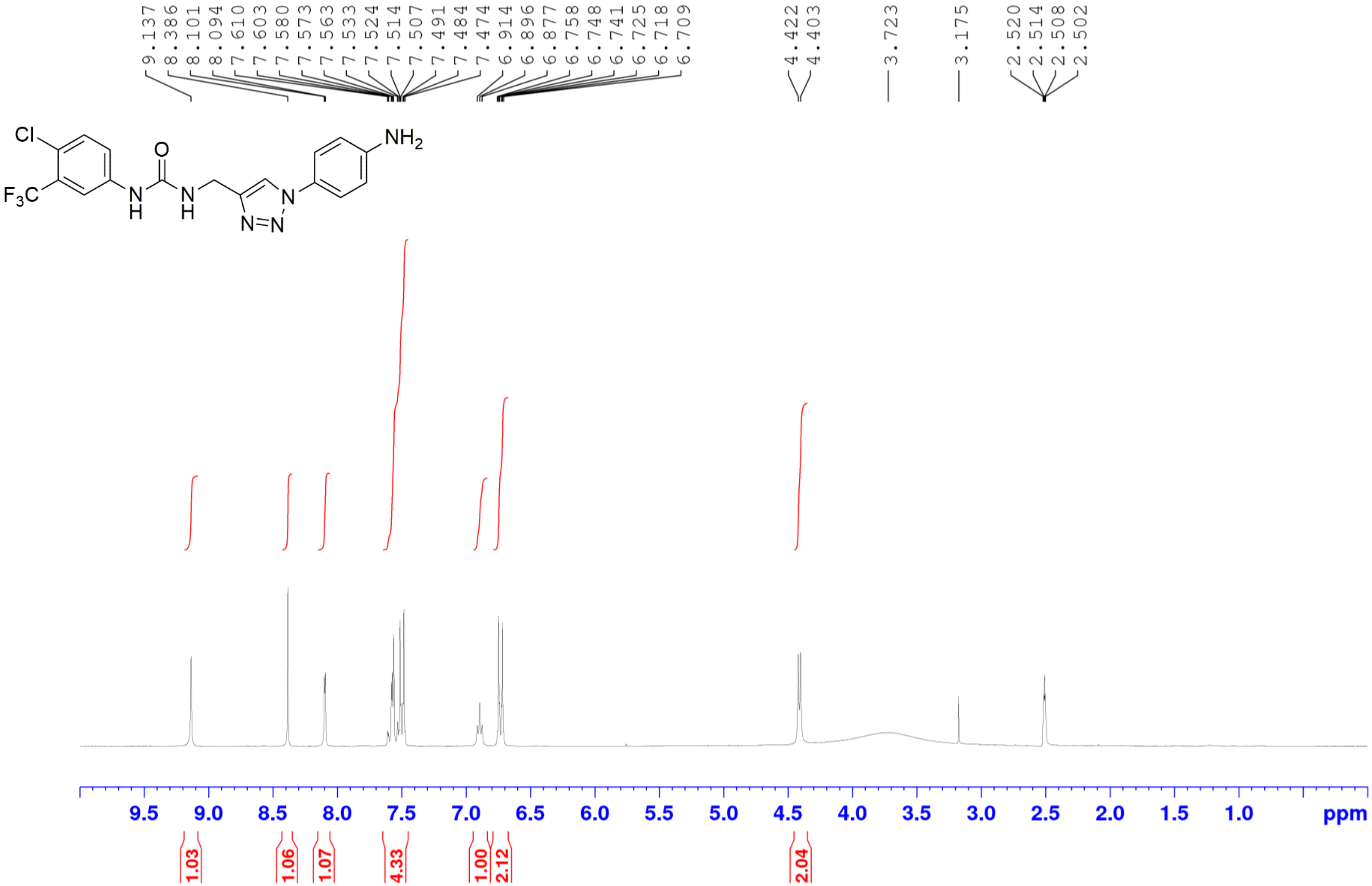

^{13}C NMR of compound **2r’** (75 MHz, DMSO-d_6)

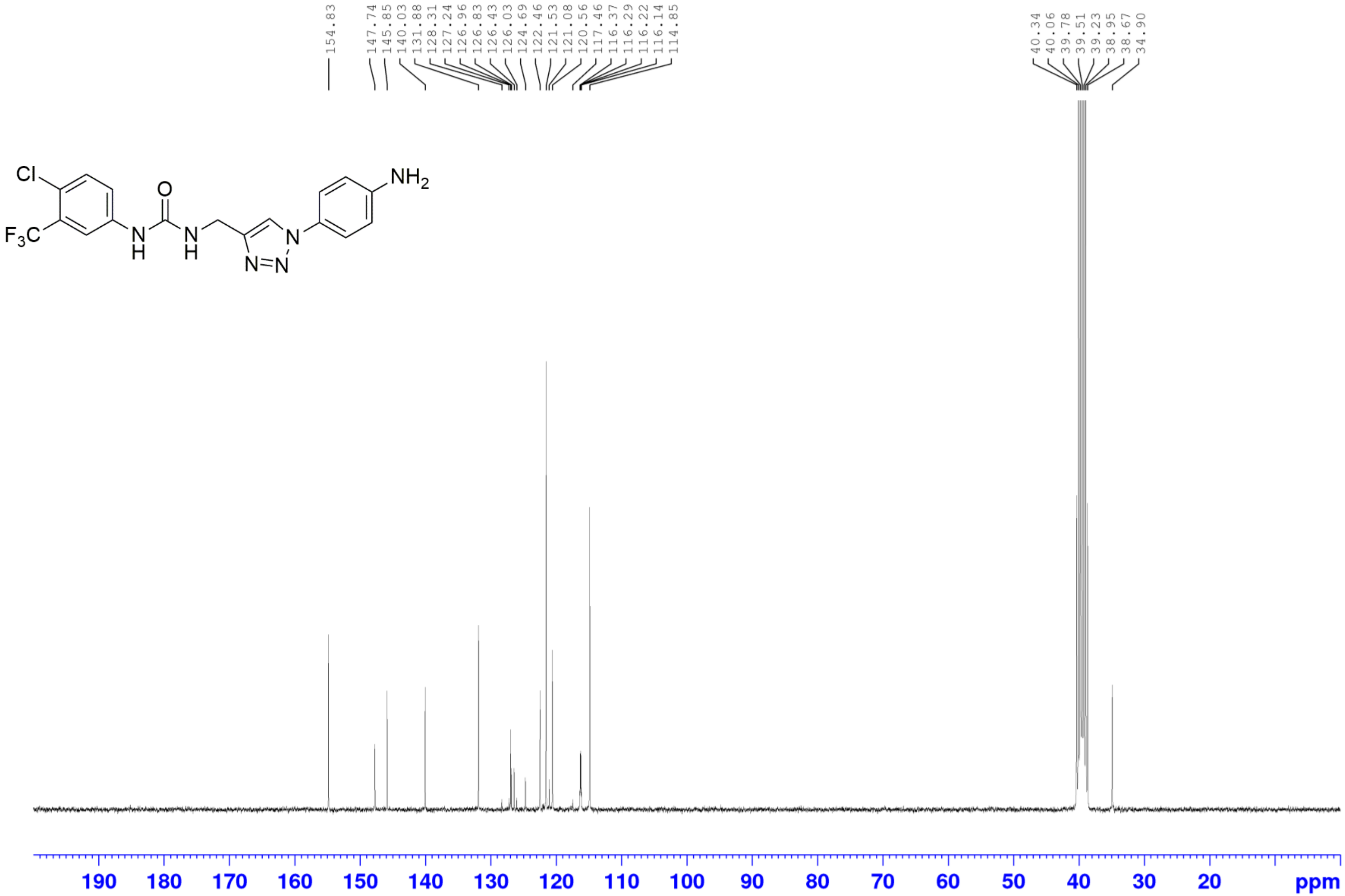

^{19}F NMR of compound **2r'** (282 MHz, DMSO-d_6)

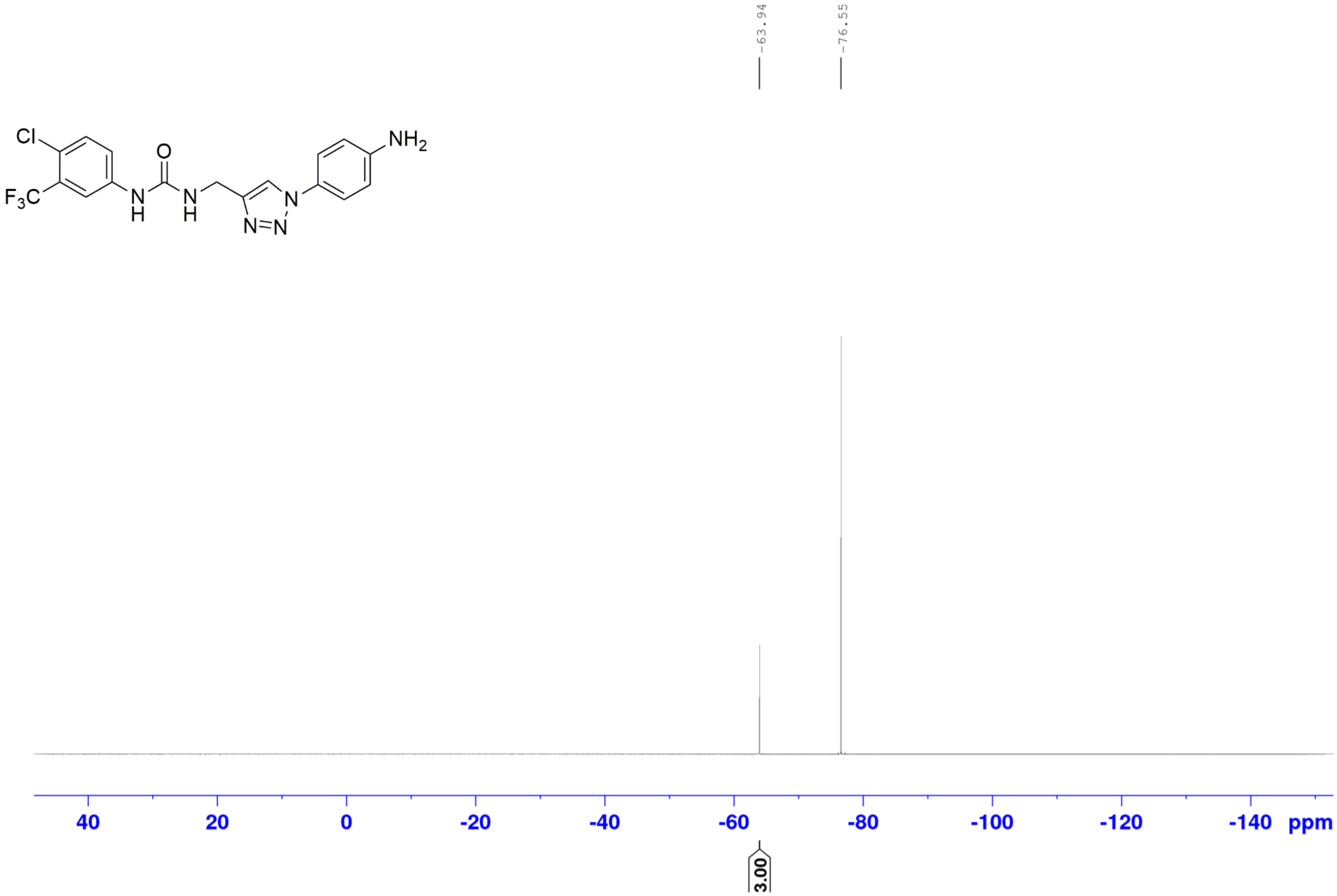

^{1}H NMR of compound **2s'** (300 MHz, DMSO-d_6)

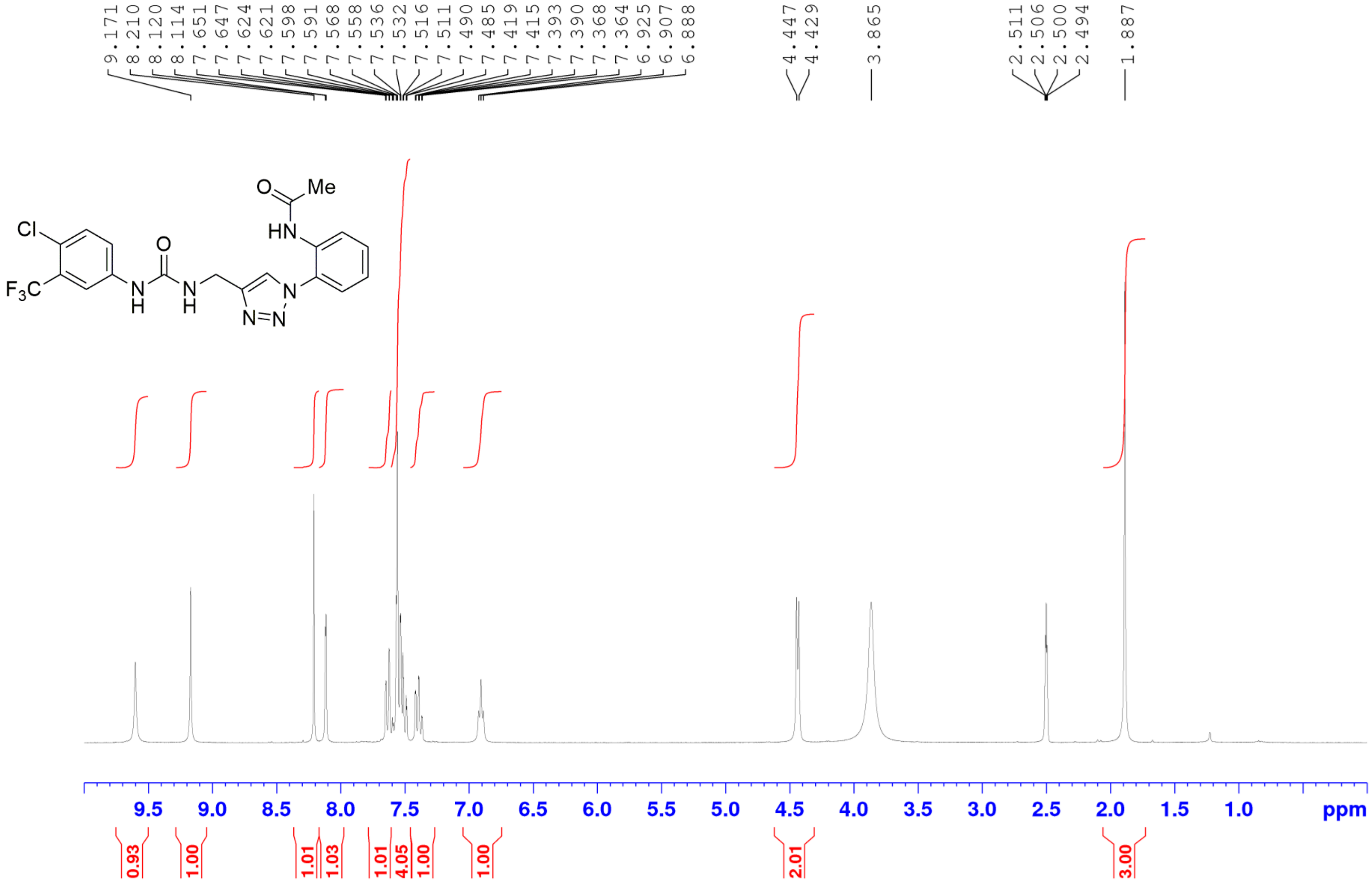

^{13}C NMR of compound **2s’** (75 MHz, DMSO-*d*$_6$)

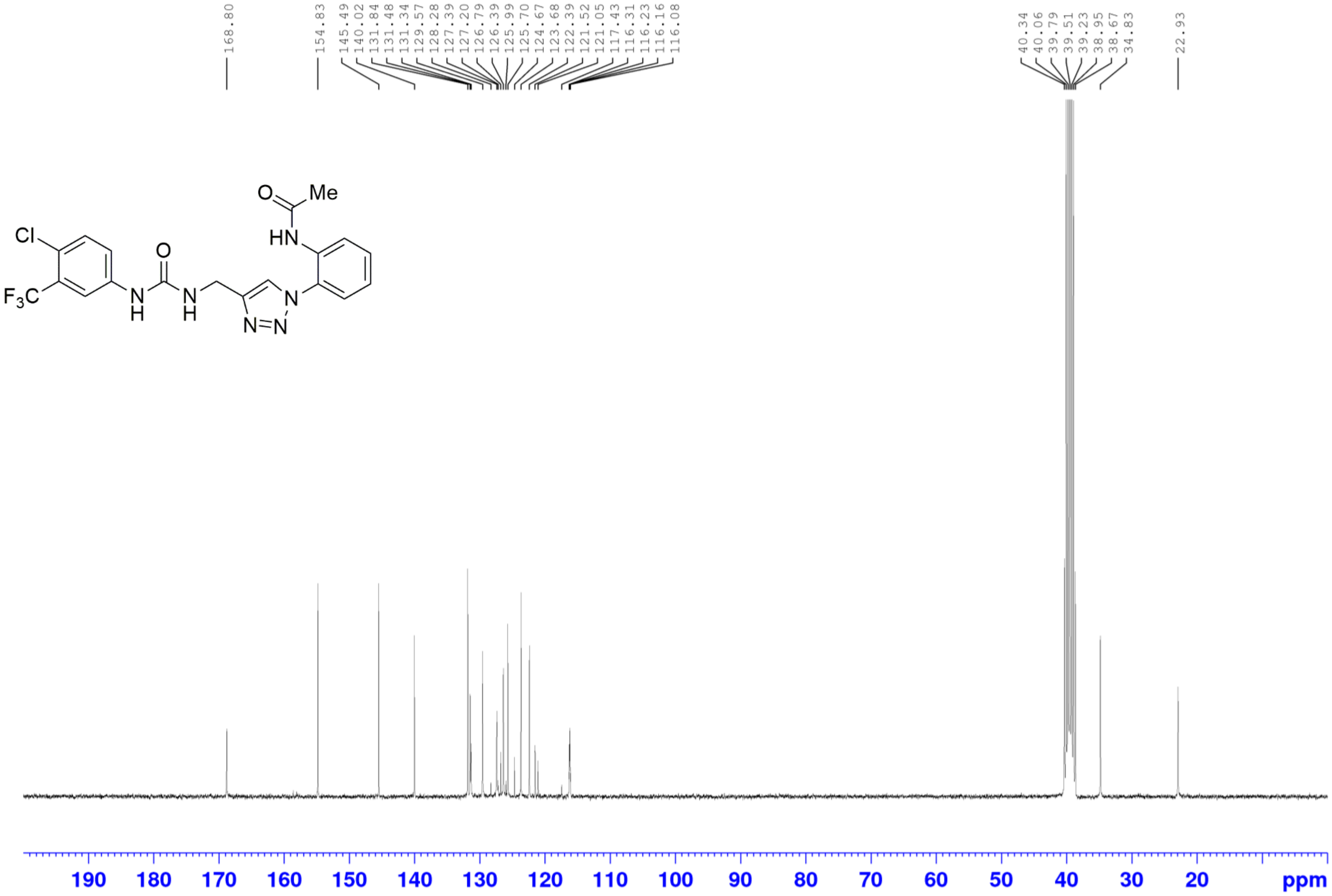

^{19}F NMR of compound **2s’** (282 MHz, DMSO-d_6)

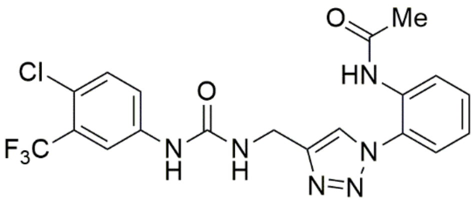

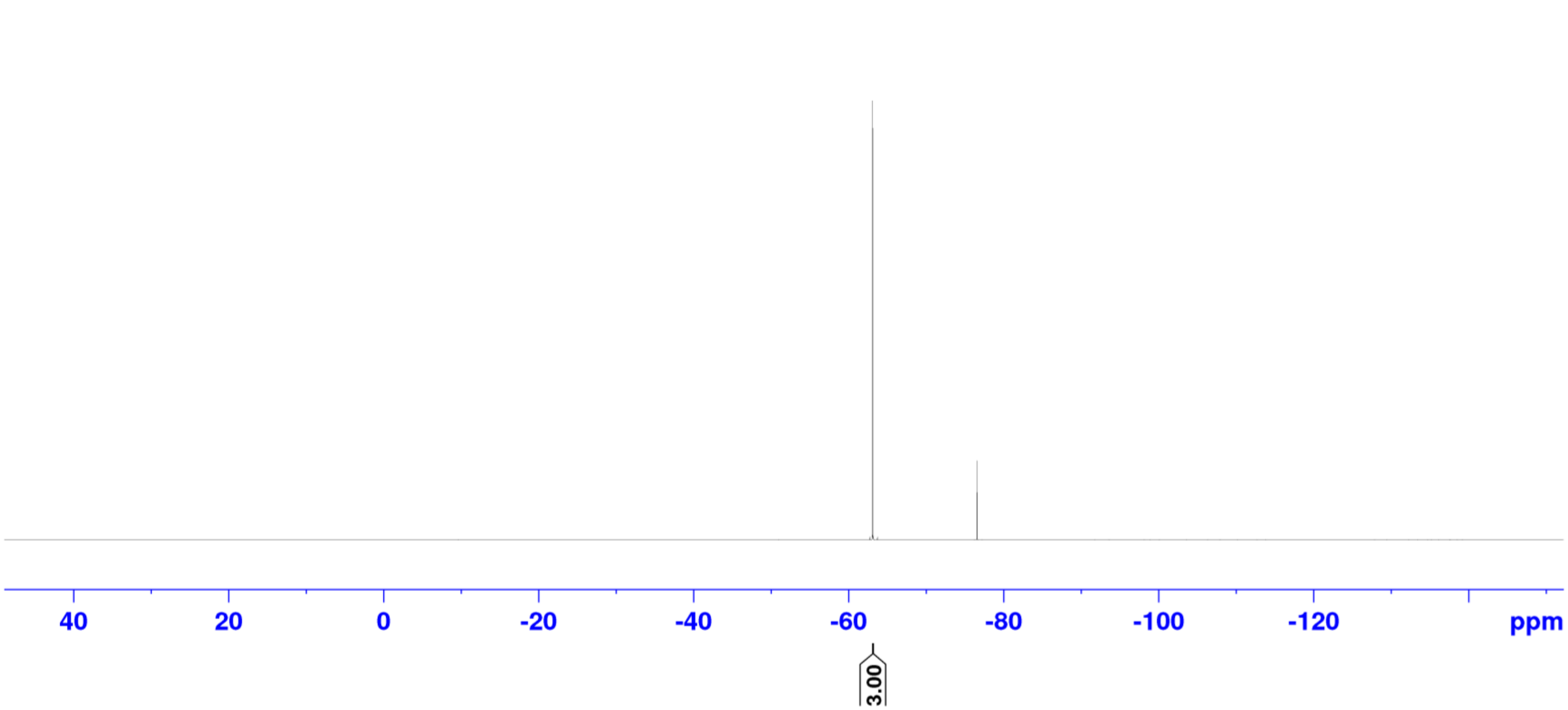

^{1}H NMR of compound **2t’** (300 MHz, DMSO-d_6)

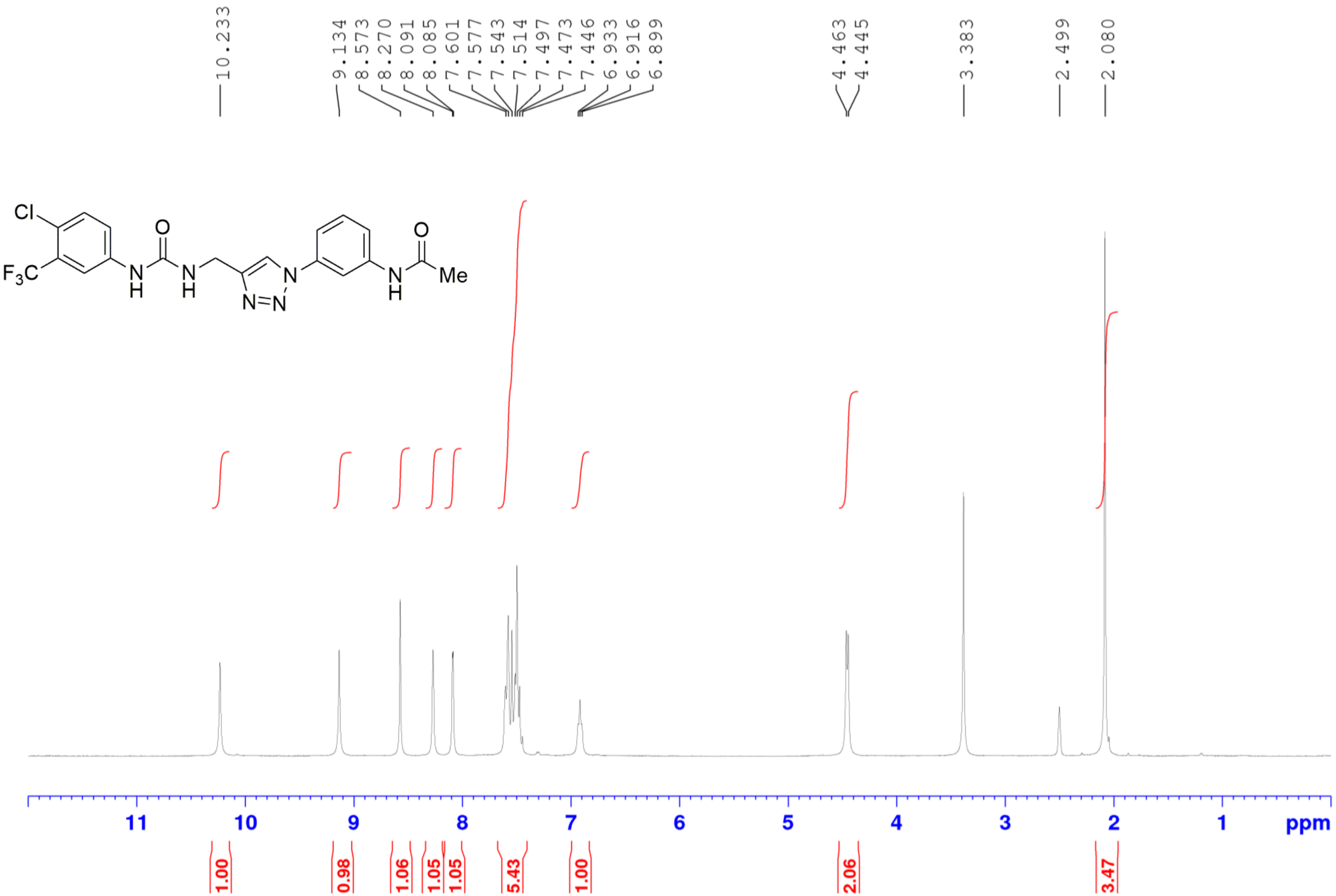

^{13}C NMR of compound **2t’** (75 MHz, DMSO-*d6*)

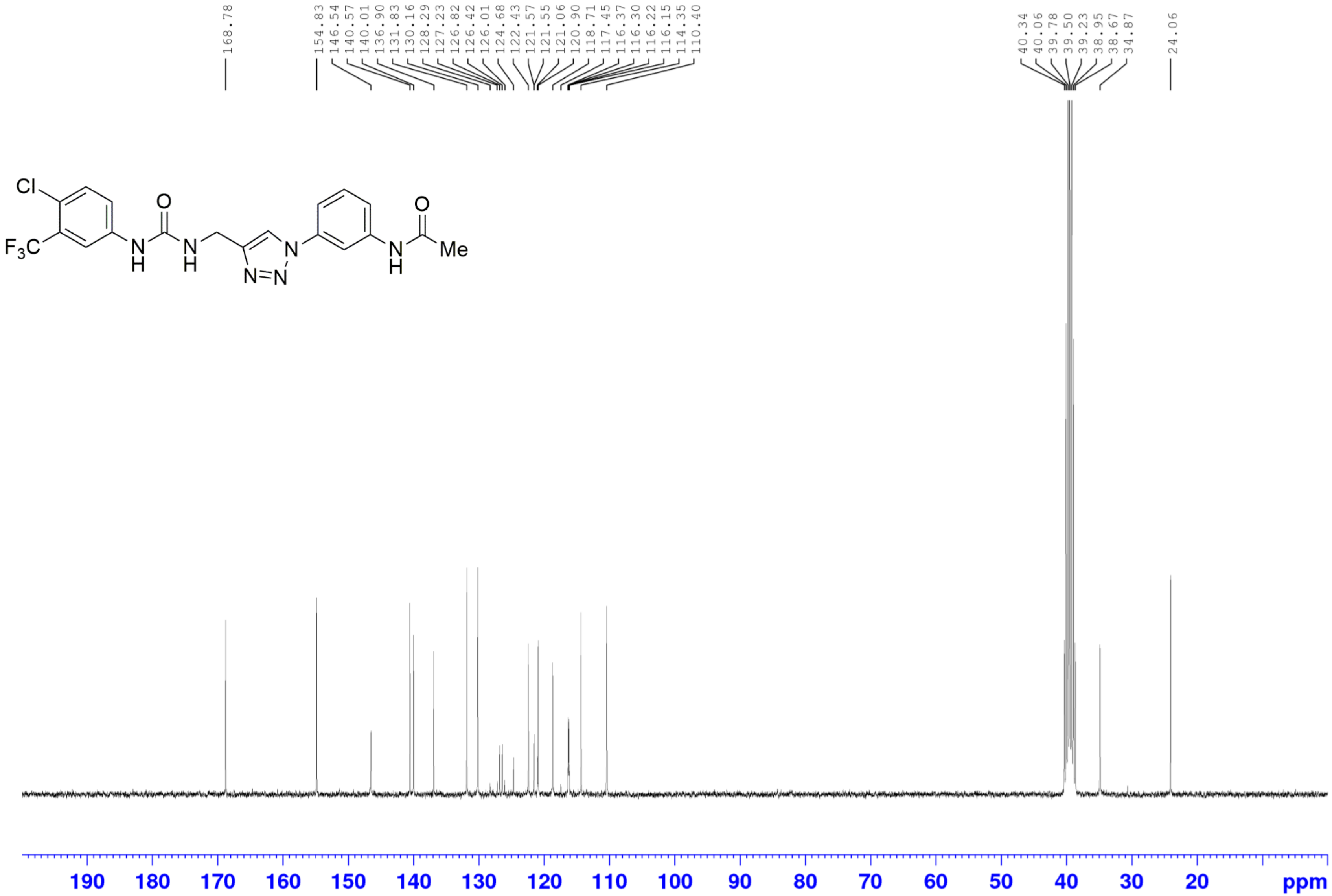

^{19}F NMR of compound **2t'** (282 MHz, DMSO-d_6)

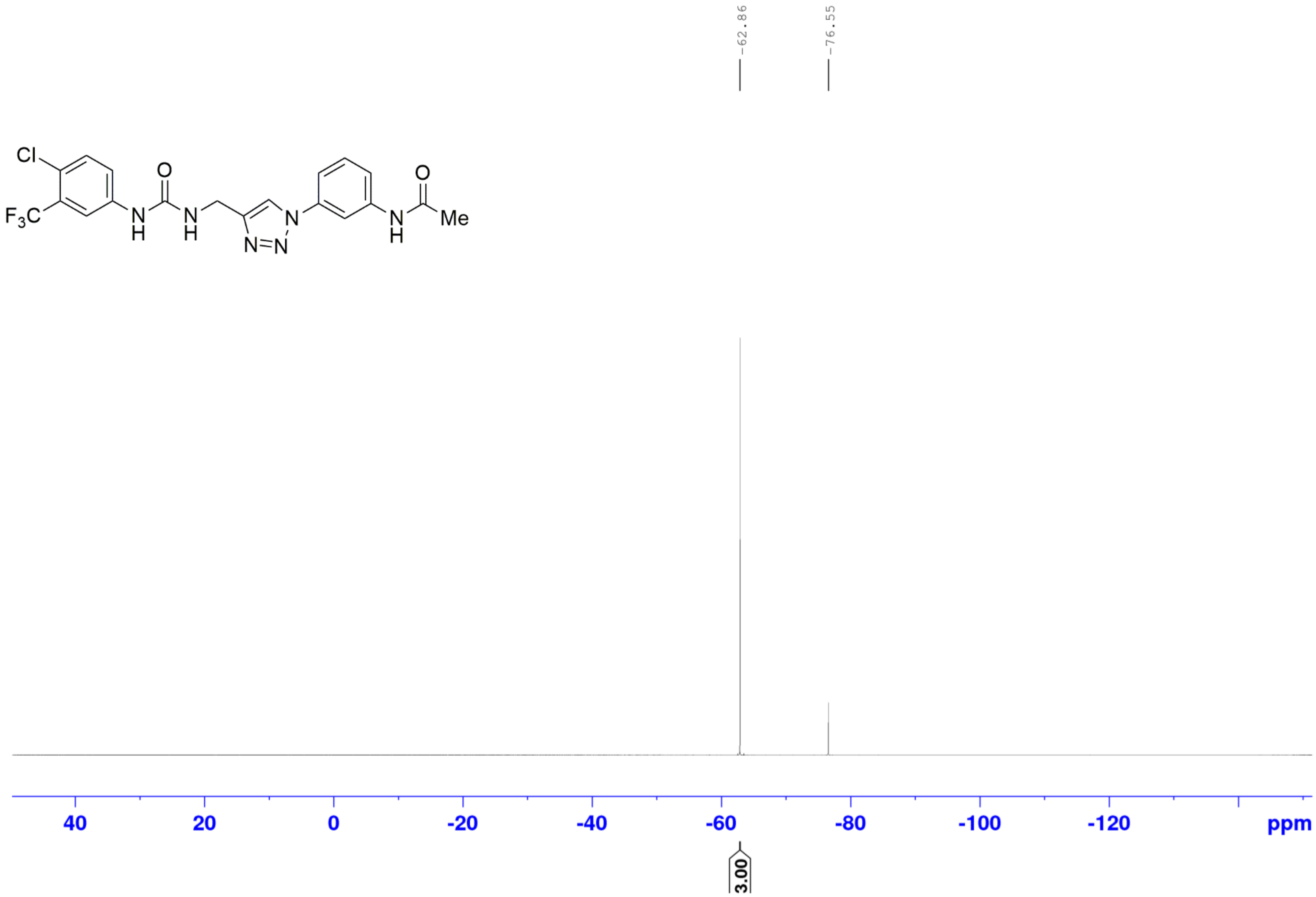

^{1}H NMR of compound **2v’** (300 MHz, DMSO-d_6)

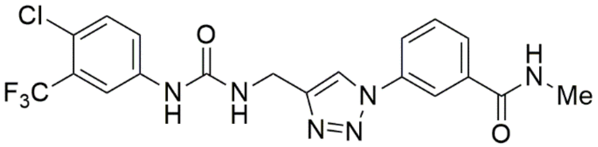

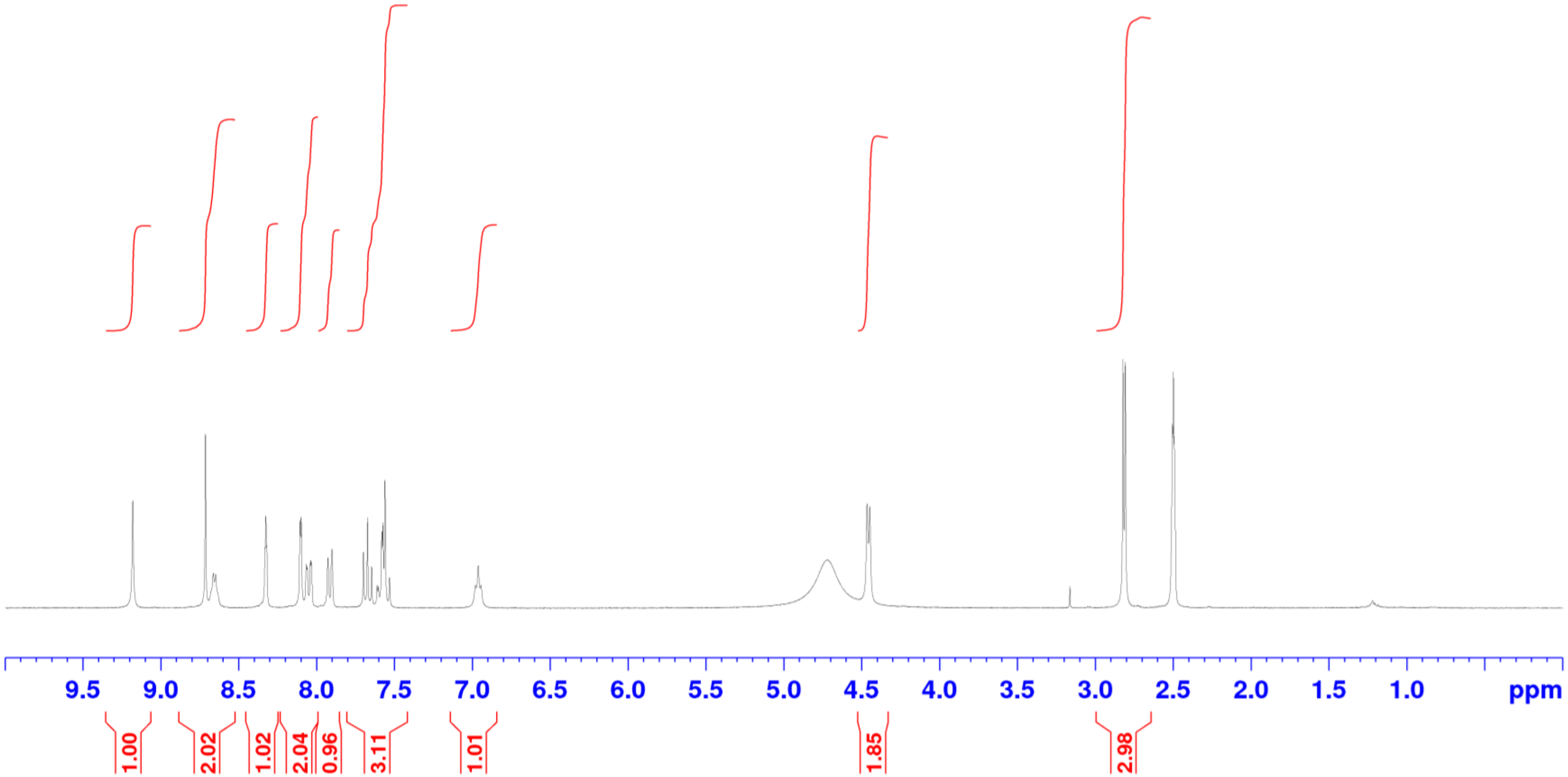

^{13}C NMR of compound **2v’** (75 MHz, DMSO-d_6)

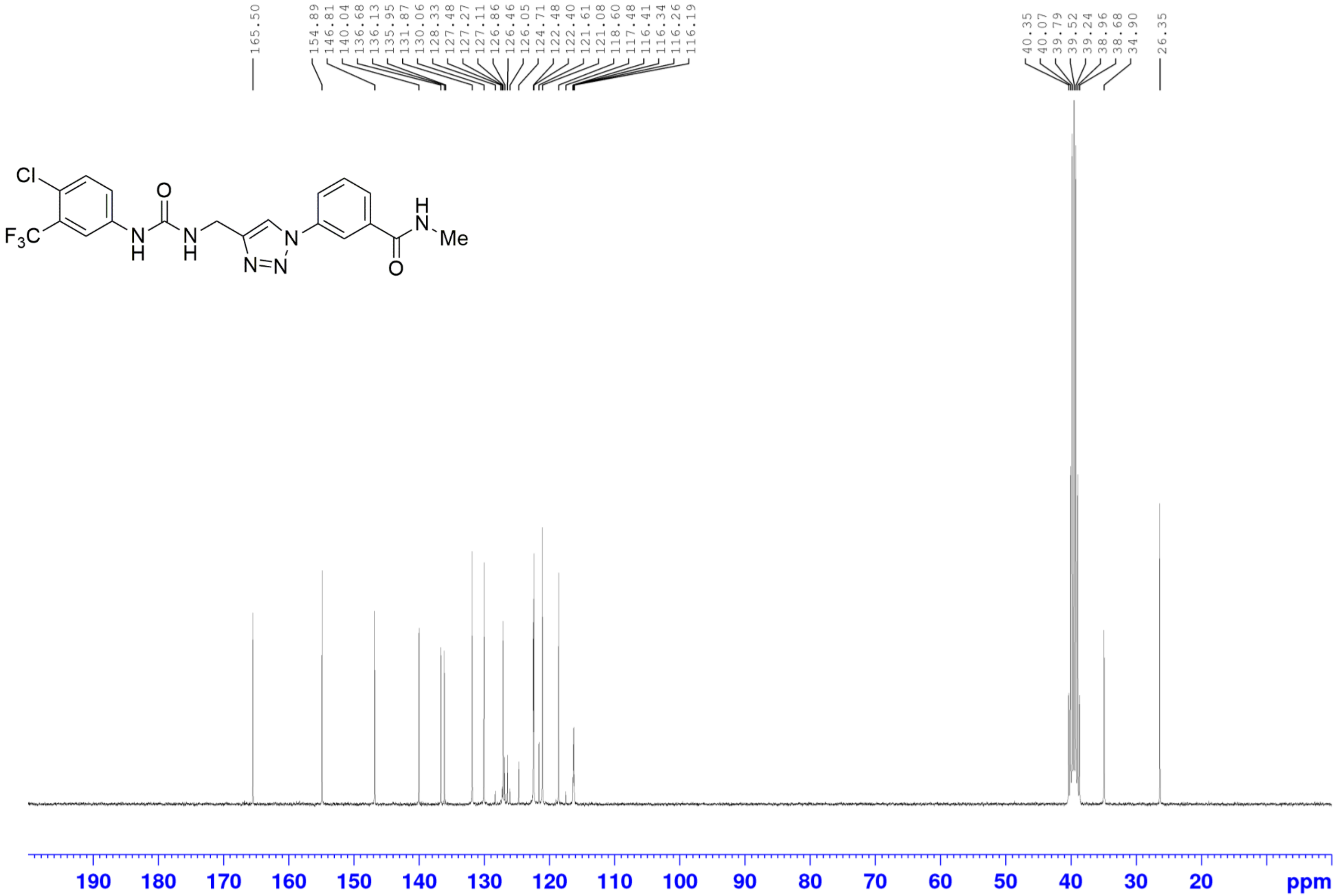

^{19}F NMR of compound **2v’** (282 MHz, DMSO-*d6*)

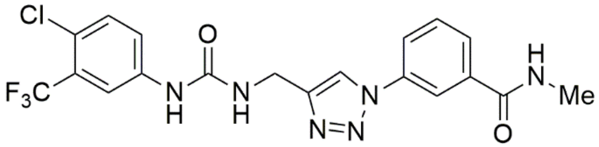

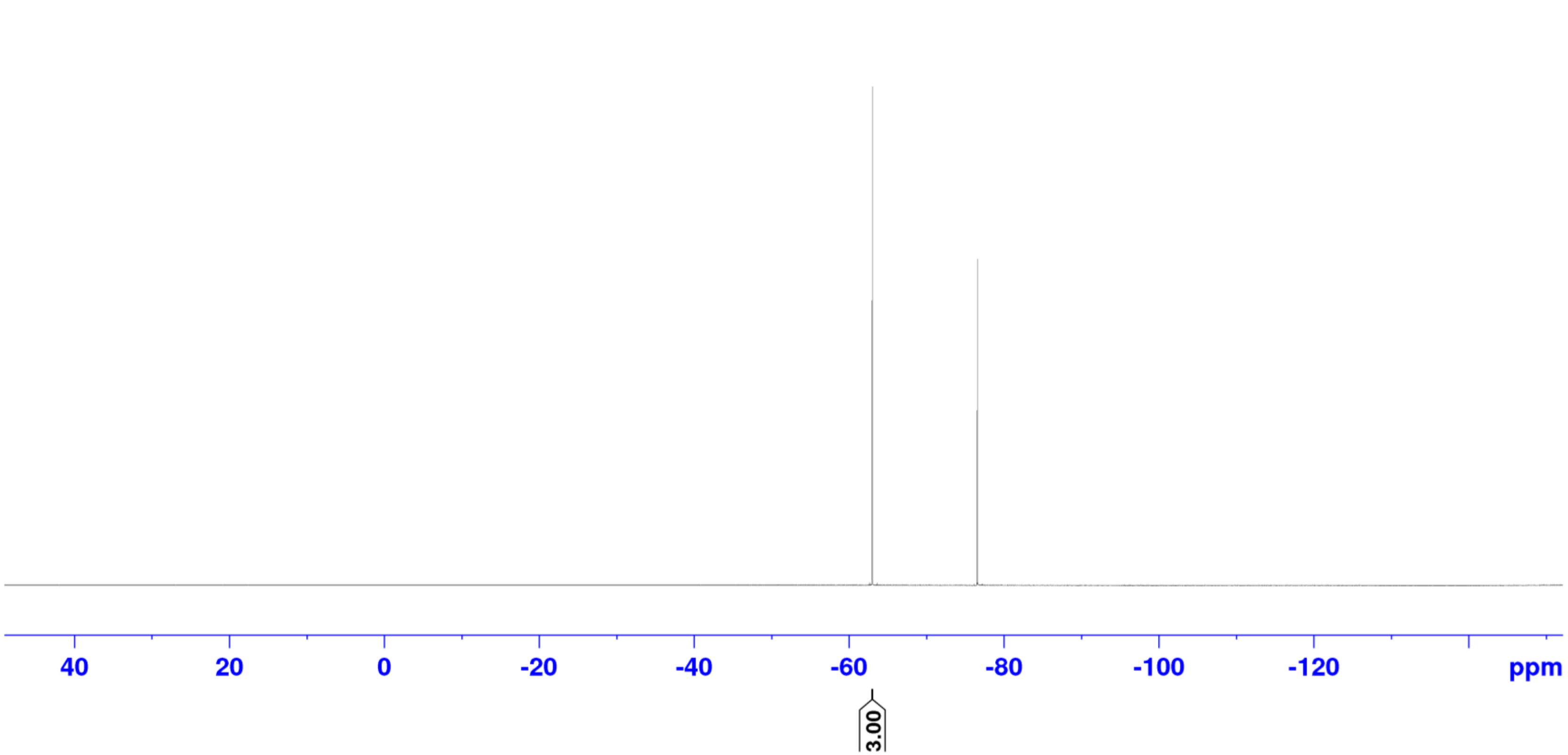

^{1}H NMR of compound **2w'** (300 MHz, DMSO-d_6)

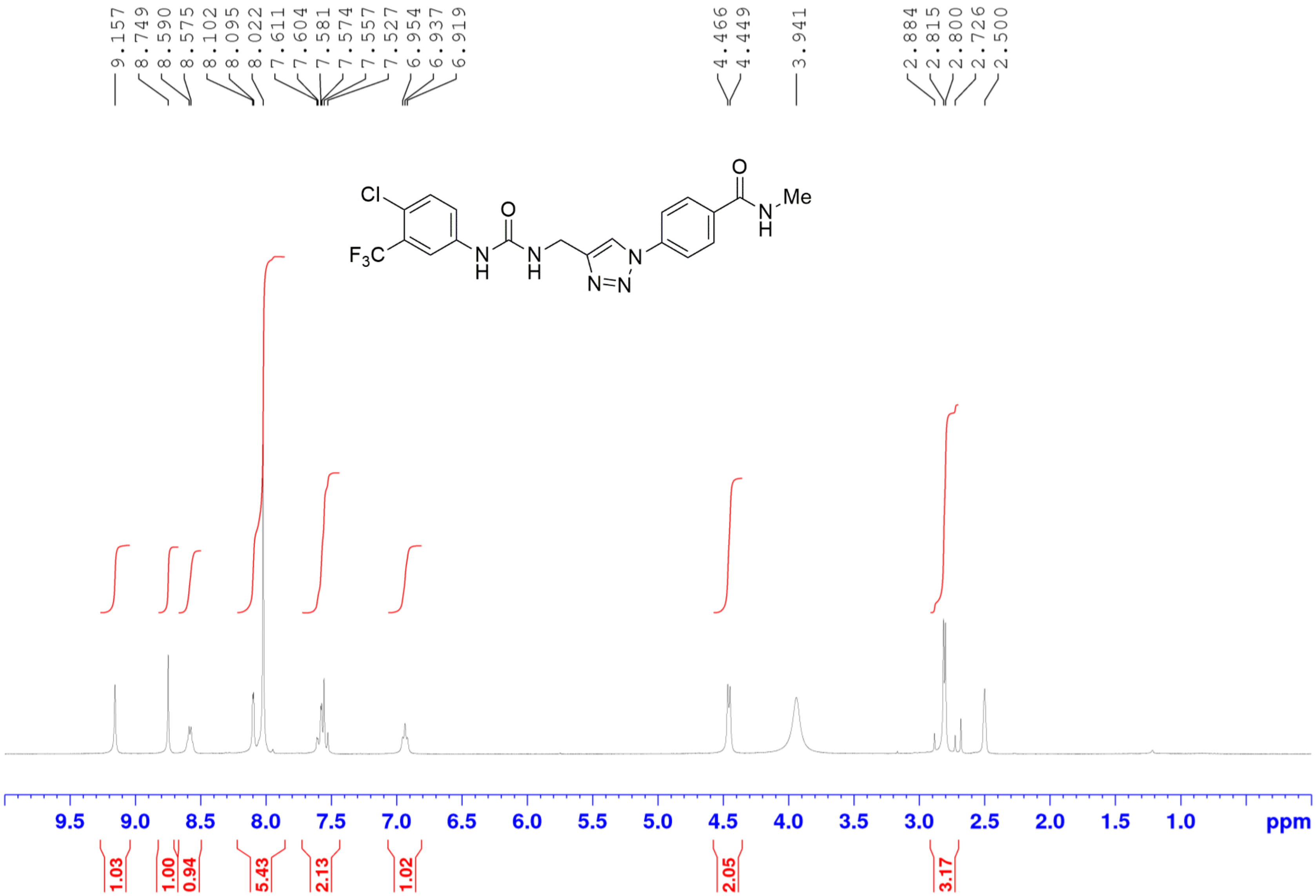

^{13}C NMR of compound **2w’** (75 MHz, DMSO-d_6)

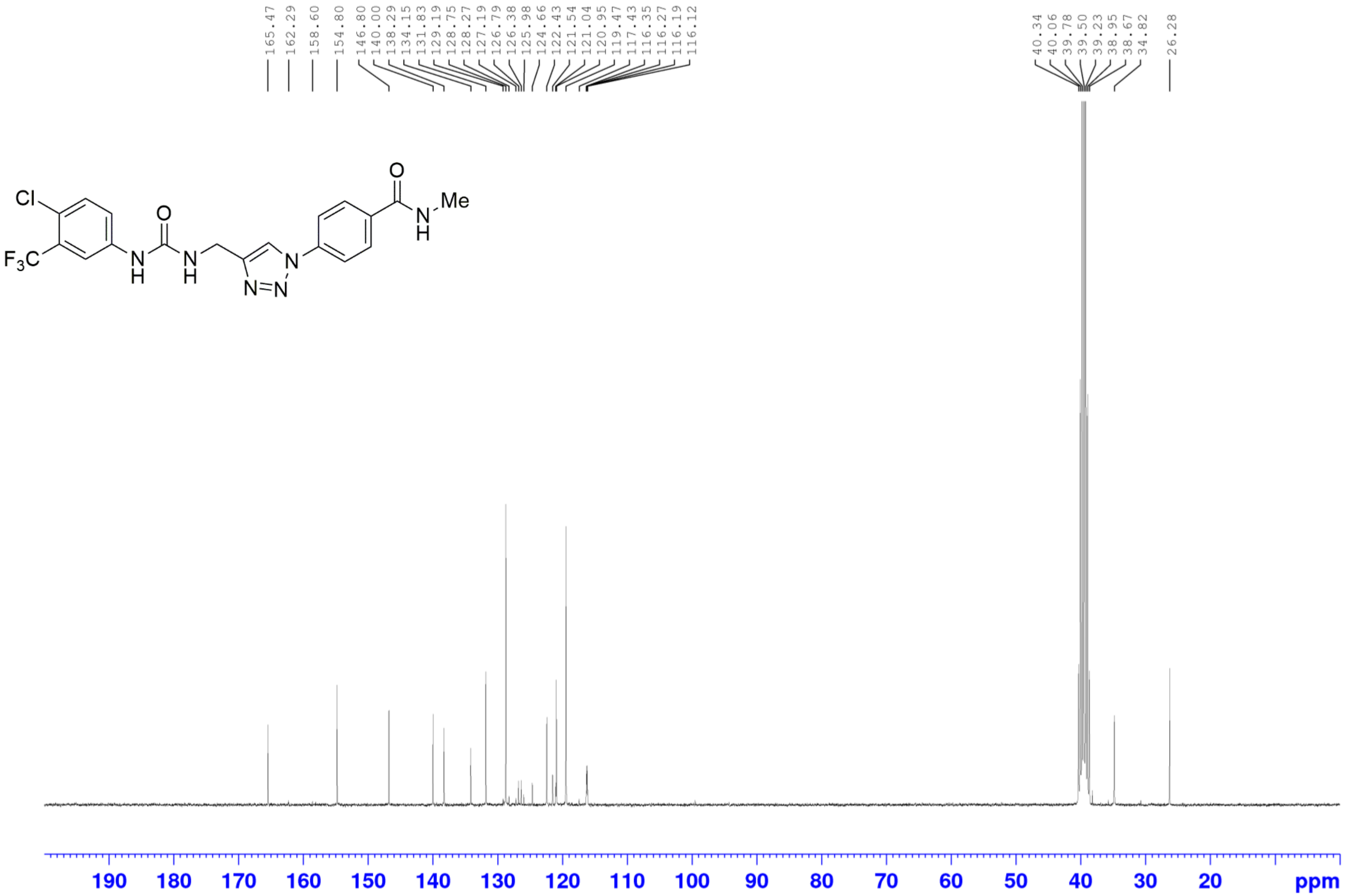

^{19}F NMR of compound **2w’** (282 MHz, DMSO-d_6)

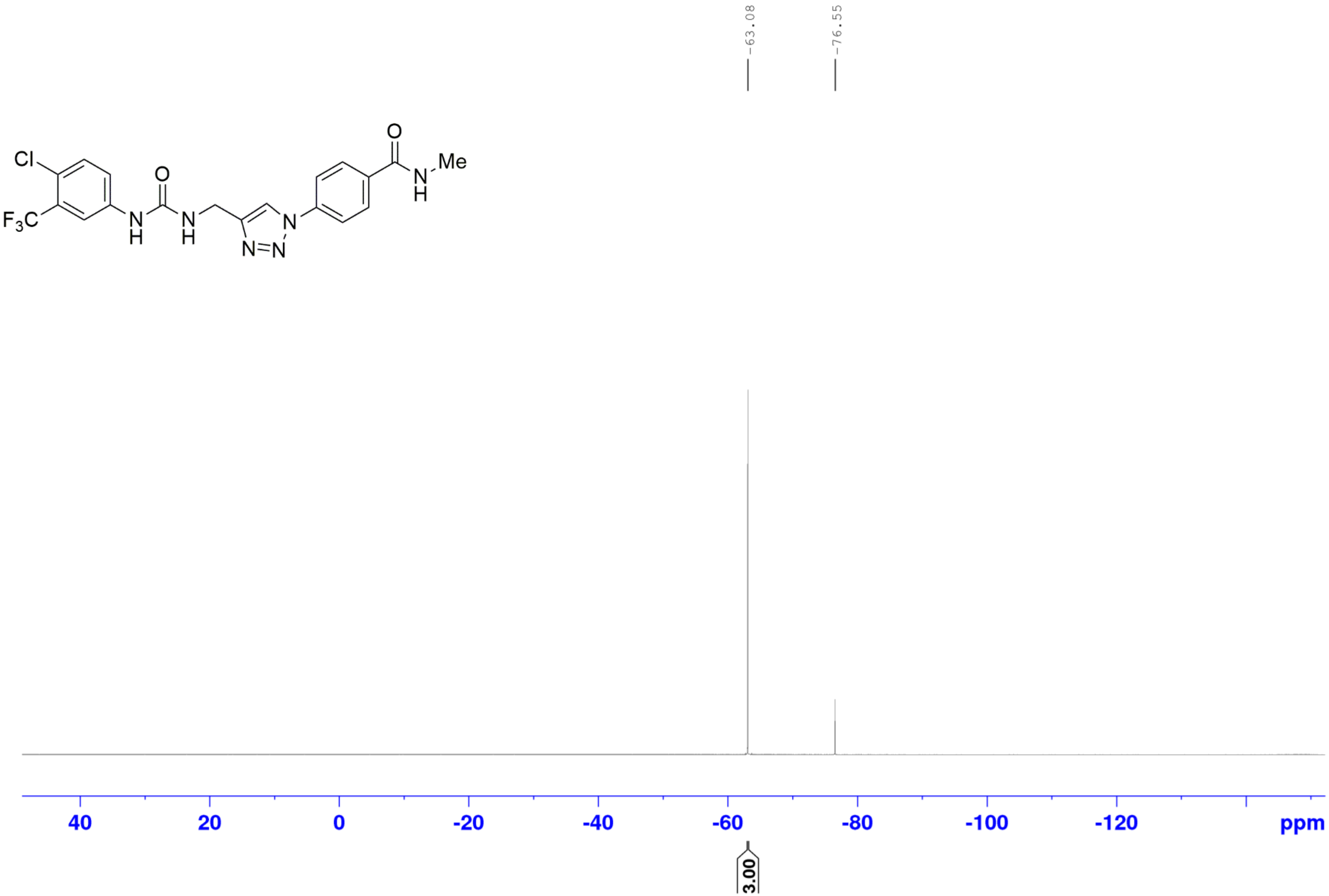

Supplement: Supplementary file 1 [file pharmaceuticals-15-00504-s001.zip › pharmaceuticals-1632951-supplementary.pdf]
